# Nickel(II)/Salox-Catalyzed Enantioselective C–H Functionalization

**DOI:** 10.1021/acscentsci.4c02049

**Published:** 2025-01-02

**Authors:** Jia-Hao Chen, Qi-Jun Yao, Ming-Yu Zhong, Tian-Yu Jiang, Fan-Rui Huang, Xiang Li, Bing-Feng Shi

**Affiliations:** † Department of Chemistry, Zhejiang University, Hangzhou 310058, China; ‡ School of Chemistry and Chemical Engineering, Henan Normal University, Xinxiang 453007, China; § College of Material Chemistry and Chemical Engineering, Key Laboratory of Organosilicon Chemistry and Material Technology, Ministry of Education, Hangzhou Normal University, Hangzhou 311121, China

## Abstract

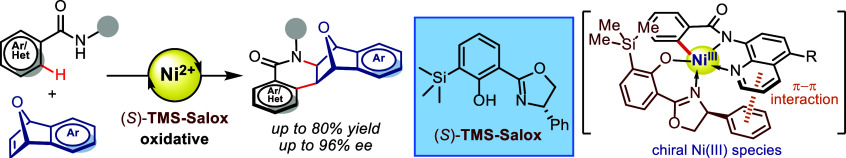

Recently, nickel catalysts have garnered considerable attention for their efficacy and versatility in asymmetric catalysis, attributed to their distinctive properties. However, the use of cost-effective and sustainable divalent nickel catalysts in C–H activation/asymmetric alkene insertion poses significant challenges due to the intricate control of stereochemistry in the transformation of the tetracoordinate C–Ni(II) intermediate. Herein, we report a Ni(II)-catalyzed enantioselective C–H/N–H annulation with oxabicyclic alkenes. This protocol offers straightforward access to chiral [2,2,1]-bridged bicyclic compounds bearing four consecutive stereocenters with high enantioselectivity (up to 96% ee). The development of a sterically hindered chiral salicyloxazoline (Salox) ligand, TMS-Salox, is key to the success of this protocol. Mechanistic investigations unveiled that a chiral Ni(III)-metalacyclic intermediate was formed through the in situ oxidation of achiral organometallic Ni(II) species and coordination of the Salox ligand. This process led to the creation of a tailored chiral pocket that guides the approach of alkenes, thereby influencing and determining the stereochemistry.

## Abstract 2

Ni(II)-catalyzed enantioselective C−H/N−H annulation with oxabicyclic alkenes using a sterically hindered chiral salicyloxazoline (Salox) ligand, TMS-Salox, was reported.

## Introduction

Over the past decade, transition-metal-catalyzed enantioselective C–H activation has opened up new opportunities for the rapid construction of chiral molecules in a straightforward and asymmetric manner.^1−8^ Owing to the earth-abundant, inexpensive, and less-toxic nature of first-row transition metals, 3d-metal-catalyzed asymmetric C–H activation reactions have emerged as a recent area of interest.^9−12^ Among the 3d metals, nickel and its complexes are particularly intriguing due to their unique properties, such as the adoption of both high- and low-spin configurations and multiple oxidation states ranging from Ni(0) to Ni(IV).^13−20^ As a consequence, the development of nickel-catalyzed enantioselective C–H activation reactions is highly desirable.

**Figure 1:**
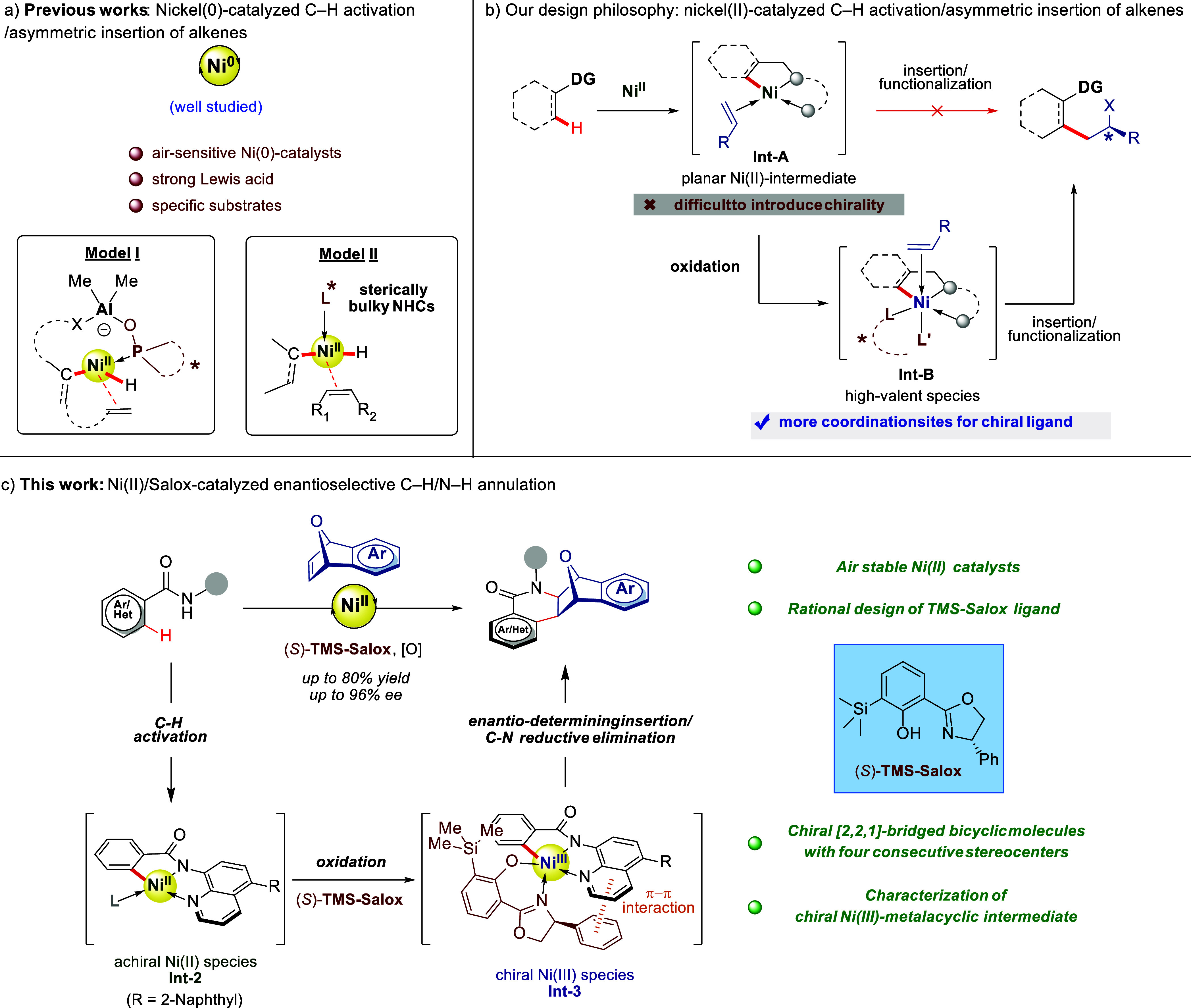
Nickel-catalyzed asymmetric C–H activation and asymmetric alkene insertion reactions. FG, functional group. SPO, secondary phosphine oxide. DG, directing group.

Meanwhile, transition-metal-catalyzed C–H activation/asymmetric alkene insertion reactions are valuable approaches to access valuable chiral skeletons.^21−34^ Nickel catalysis has made significant strides in this burgeoning area. Cramer reported the first nickel(0)-catalyzed formamide C–H activation/intramolecular enantioselective hydrocarbamoylation of alkenes through a heterobimetallic mode with nickel(0) catalyst and Lewis acid using chiral secondary phosphine oxide (SPO) type ligands.^21^ Since this seminal work, extensive studies have been conducted in this area using nickel(0) catalysts, leading to the development of two primary models for asymmetric induction during alkene insertion. These models include Ni–Al heterobimetallic catalysis using chiral SPOs and the utilization of sterically hindered chiral ligands, such as chiral N-heterocyclic carbenes (NHCs).^21−28^ Despite these advances, the current methods rely on air-sensitive Ni(COD)_2_ catalysts, necessitating specialized inert reaction conditions. Additionally, C–H activations are largely limited to acidic C–H bonds or those activated by strong Lewis acids such as AlMe_3_ (Figure 1a). Therefore, there is a growing demand for the development of more sustainable and user-friendly catalytic systems in nickel-catalyzed C–H activation/asymmetric alkene insertion reactions.

The use of commercially available and stable nickel(II) salts as catalysts in conjunction with strongly coordinating directing groups (DGs) has been demonstrated as a robust catalytic system for the functionalization of unactivated C–H bonds.^19,20,35−43^ Unfortunately, the harsh reaction conditions and the absence of suitable chiral ligands have hindered the development of enantioselective C–H functionalization with Ni(II) catalysis. Recently, we successfully achieved the nickel/BINOL-catalyzed enantioselective C–H alkynylation of planar chiral metallocenes through desymmetrization. Mechanistic studies revealed that C–H cleavage is the enantio-determining step, with the BINOL ligand dissociating after this cleavage. Consequently, this Ni(II)/BINOL catalytic system is not suitable for the C–H activation/asymmetric alkene insertion reactions.^44^ Our longstanding interest in 3d-metal-catalyzed asymmetric C–H activation^45−51^ motivated us to tackle this challenge and develop a Ni(II)-catalyzed C–H activation/asymmetric alkene insertion reaction.

Mechanistically, as depicted in Figure 1b, the reaction pathway of our targeted transformation proceeds through base-concerted divalent nickelation of C–H bonds and alkene coordination to generate **Int-A**.^52−57^ Generally, the subsequent alkene insertion and functionalization would be the key enantiocontrol step. However, the resulting tetracoordinate C–Ni(II) intermediate **Int-A** lacks additional coordination sites for chiral ligands to introduce chirality during either alkene insertion or the following functionalization steps. To overcome this inherent challenge, we envisioned that the high-valent nickel species (+III or VI) **Int-B**, generated from the *in situ* oxidation of **Int-A**, could offer more positions for chiral ligands, given their penta- or hexa-coordinated frameworks. Moreover, the specific three-dimensional nature of **Int-B** in conjunction with the elaborated chiral ligand would offer precise control over the orientation of alkene coordination and the facial selectivity of subsequent C–Ni bond migratory insertion. Nonetheless, the development of such an asymmetric transformation is exceptionally challenging due to the inherent stereochemical variability in nickel complexes.^58^

Very recently, we successfully achieved high-valent cobalt-catalyzed enantioselective C–H activation reactions, utilizing tailor-made chiral salicyloxazoline (Salox) ligands.^46−51,59−62^ The exceptional enantioselectivities observed in these protocols are attributed to the precise arrangement of Salox ligands and substrates in an octahedral configuration around trivalent cobalt ions.^46^ Inspired by these previous studies, we envisaged that the well-designed Salox ligands could effectively regulate the stereochemistry around high-valent nickel centers, leveraging the diverse stereochemical properties of multiple coordination complexes to bring our proposed concept to fruition. Furthermore, considering the distinct reactivity profiles inherent in cobalt and nickel catalysis, we anticipated that these variations might lead to divergent reaction pathways different from cobalt catalysis. Herein, we report a Ni(II)-catalyzed enantioselective C–H/N–H annulation with *meso-*oxabicyclic alkenes through a Ni(II/III/I) catalytic pathway enabled by a tailor-made sterically hindered Salox ligand, TMS-Salox. Mechanistic studies revealed that the reaction proceeded through Ni(II)-catalyzed C–H activation to form the achiral square-planar cyclometalated Ni(II) **Int-2**. Subsequent oxidation led to the formation of chiral Ni(III) species **Int-3** stabilized by the TMS-Salox ligand, which underwent an enantio-determining alkene insertion/C–N reductive elimination process to give the [4 + 2] annulation products (Figure 1c).^63^

## Results and Discusion

**Figure 2:**
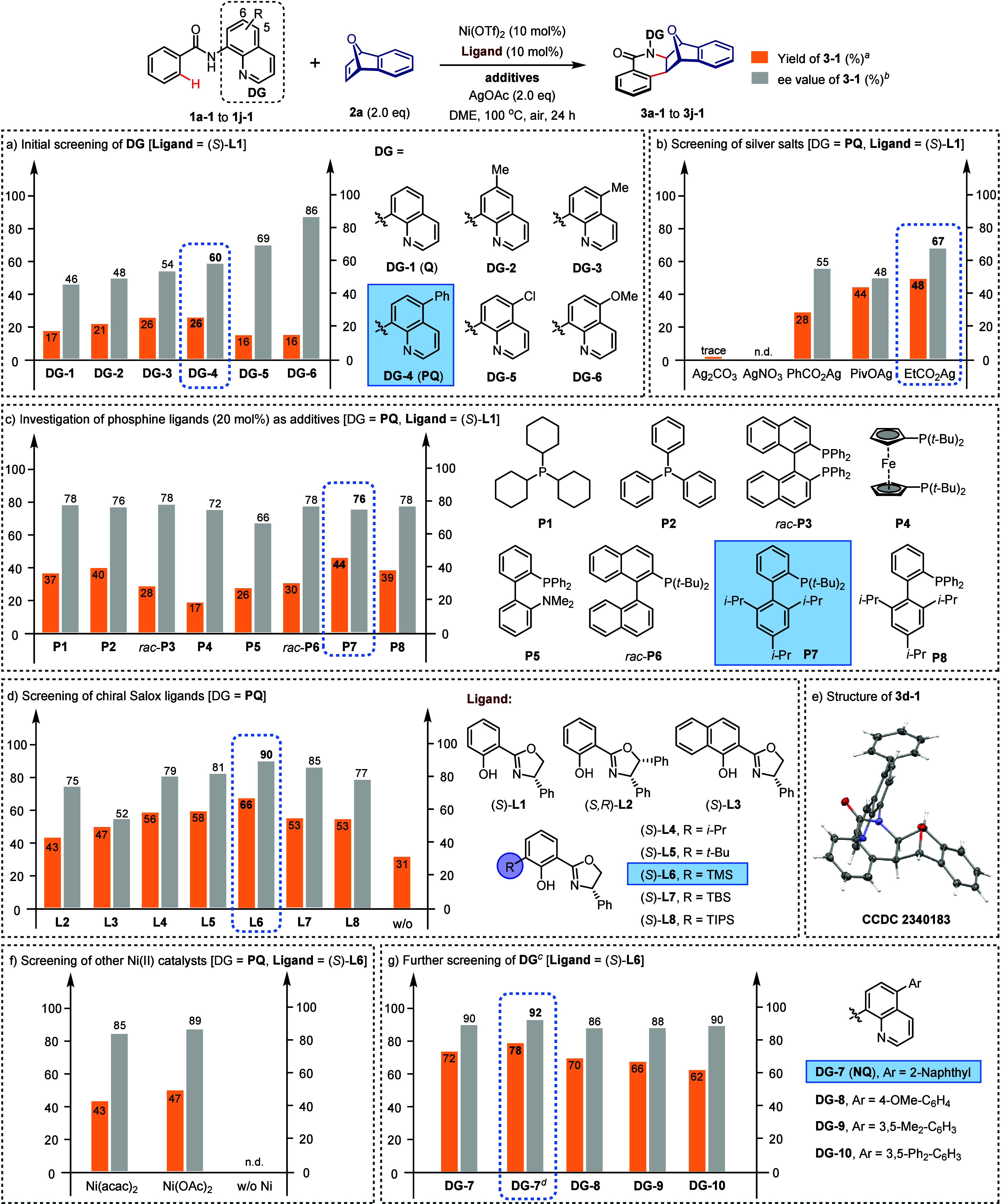
Optimization of the reaction conditions. Reaction conditions: **1**–**1** (0.10 mmol), **2a** (2.0 equiv), Ni(OTf)_2_ (10 mol %), **Ligand** (10 mol %), **additives** (20 mol %), silver salts (2.0 equiv), DME (0.5 mL), air, 100 °C, 24 h. ^*a*^The yields were determined by ^1^H NMR. ^*b*^The ee values were determined by HPLC on a stationary chiral stationary phase. ^*c*^Isolated yields. ^*d*^20 mol % (*S*)-**L6** was used.

Bridged heterobicyclic alkenes can undergo a wide variety of reactions and have demonstrated their value in the efficient and stereoselective synthesis of chiral bicyclic compounds.^64−68^ These frameworks also exhibit distinct reactivity patterns in transition-metal-catalyzed enantioselective C–H activation reactions.^69−74^ Thus, we began our studies by investigating the reaction of *N*-(quinolin-8-yl)benzamides **1**–**1** bearing substituents on the quinoline moiety with 7-oxa-benzonorbornadiene (**2a**). The desired Ni(II)-catalyzed C–H activation/asymmetric alkene insertion reaction was initially optimized with 10 mol % Ni(OTf)_2_, 10 mol % Salox ligand (*S*)-**L1**, and 2.0 equiv of AgOAc (Figure 2a). To our excitement, [4 + 2] annulation products **3a-1** to **3e-1** were obtained with moderate enantioselectivities (46% to 69% ee) albeit in low yields (16% to 26%). Although the bidentate DG (**DG-6**) with a methoxy group at the 5-position afforded **3f-1** in 86% ee, it displayed low reactivity, hindering further optimization. Consequently, we selected the quinoline framework with a phenyl group at the 5-position (**DG-4**, **PQ**) for subsequent investigations. The use of silver carboxylates as oxidants facilitated a smooth reaction, with EtCO_2_Ag giving **3d-1** in 48% yield with 67% ee. In contrast, inorganic silver salts such as Ag_2_CO_3_ and AgNO_3_ failed to deliver the desired product (Figure 2b). Building on previous studies that phosphine ligands can promote the reaction of Ni(II)-catalyzed C–H activation,^35,37,38^ we further explored the impact of various phoshines as crucial additives. We were pleased to find that the enantioselectivity was improved slightly when 20 mol % phosphine ligands (**P1** to **P8**) were investigated (Figure 2c). Phosphine **P7** was found to be optimal, giving the desired product in 44% yield with 76% ee. In our subsequent investigations focusing on Salox ligands, we observed that Salox ligands with bulky substituents at the *ortho*-position of the phenolic group, such as isopropyl (**L4**), *tert*-butyl (**L5**), and trimethylsilyl (**L6**), displayed significantly enhanced activity and enantioselectivity compared to **L1** (Figure 2d). Notably, the use of **L6** gave **3d-1** in 66% yield with an impressive 90% ee. However, attempts to further enhance the enantioselectivity by employing even bulkier *t*-butyldimethylsilyl (TBS, **L7**) or triisopropylsilyl (TIPS, **L8**) groups resulted in decreased yields and ee values. Intriguingly, racemic product **3d-1** was obtained in a substantially decreased yield of 31% in the absence of a Salox ligand, suggesting a case of ligand-accelerated catalysis. The configuration of **3d-1** was unambiguously established by X-ray analysis (CCDC 2340183), providing clear insights into the *exo*-coordination of oxabicyclic alkenes toward *exo*-selective products (Figure 2e). Additionally, a comparative evaluation of other Ni(II) catalysts, including Ni(acac)_2_ and Ni(OAc)_2_, revealed the superiority of Ni(OTf)_2_. A control experiment further confirmed that no product was formed in the absence of a Ni(II) catalyst (Figure 2f). Finally, by replacing the phenyl group on the quinoline moiety with a larger naphthyl unit and increasing the loading of **L6** to 20 mol %, a significant improvement of the yield and enantioselectivity of product **3g-1** was achieved (Figure 2g, 78%, 92% ee).

**Figure 3:**
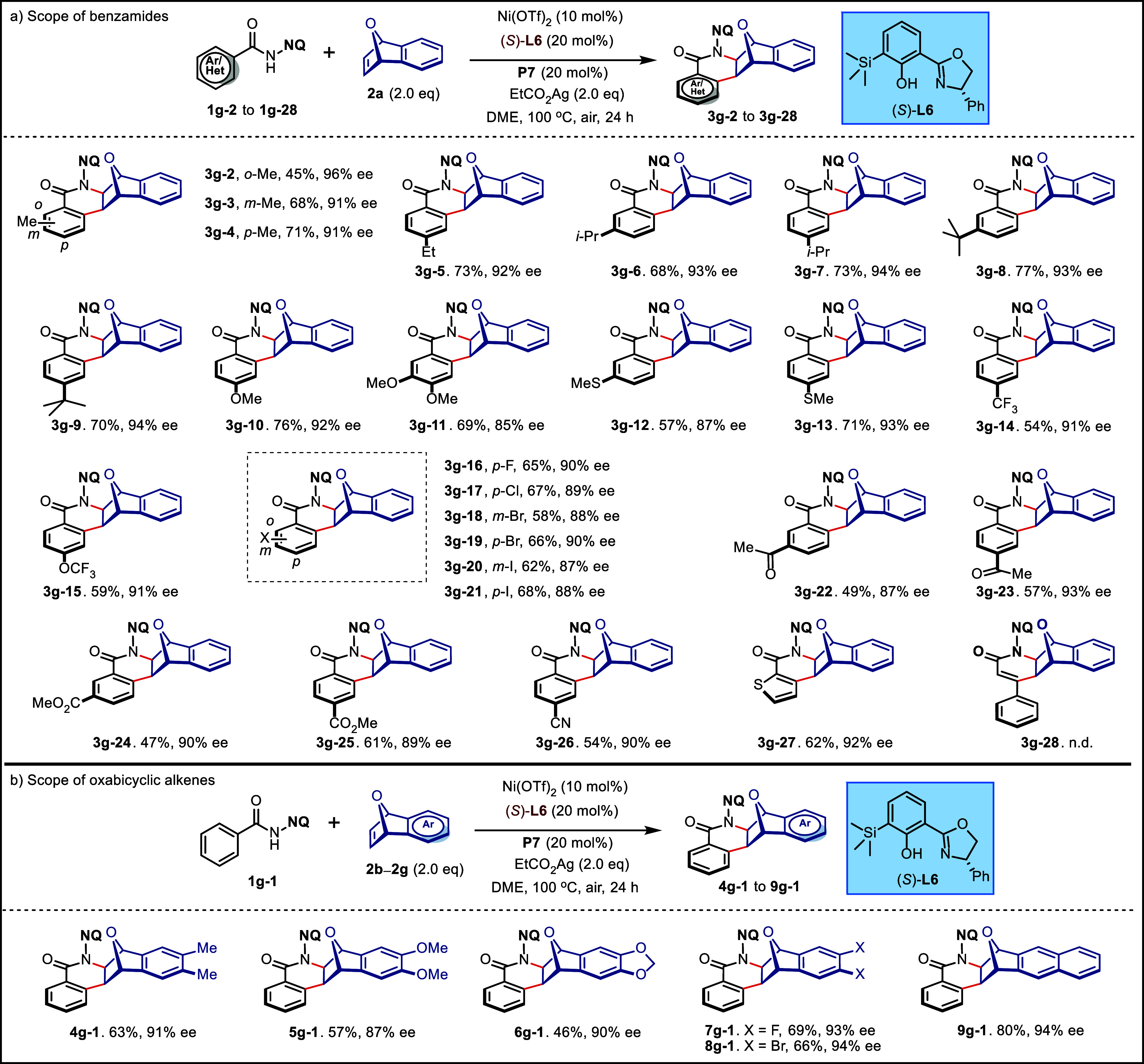
Investigation of the substrate scope. (a) Reaction conditions: **1g** (0.10 mmol), **2a** (2.0 equiv), Ni(OTf)_2_ (10 mol %), (*S*)-**L6** (20 mol %), **P7** (20 mol %), EtCO_2_Ag (2.0 equiv), DME (0.5 mL), air, 100 °C, 24 h. (b) Reaction condition: **1g-1** (0.10 mmol), **2b**–**2g** (2.0 equiv), Ni(OTf)_2_ (10 mol %), (*S*)-**L6** (20 mol %), **P7** (20 mol %), EtCO_2_Ag (2.0 equiv), DME (0.5 mL), air, 100 °C, 24 h. Isolated yield. ee values were determined by HPLC on a chiral stationary phase. n.d., not detected.

With the optimal conditions in hand, the scope of this protocol was examined. In general, benzamides bearing both electron-donating and -withdrawing groups reacted smoothly with **2a**, furnishing the desired chiral [2,2,1]-bridged bicyclic products in satisfactory yields with good enantioselectivities (Figure 3a, 45 to 77% yield, 85% to 96% ee). To our delight, diversely decorated substrates bearing valuable functional groups, such as trifluoromethoxy (**3g-15**), halos (**3g-16** to **3g-21**), acetyl (**3g-22**, **3g-23**), esters (**3g-24**, **3g-25**), and cyan group (**3g-26**), were well-tolerated. Notably, this protocol is also suitable for sulfur-containing benzamides such as **1g-12** and **1g-13**, although the sulfur atom exhibits a strong coordination capability with metals, leading to potential catalyst deactivation. Thiophene-2-carboxamide **1g-27** was compatible with this reaction, giving the annulation product **3g-27** in 62% yield with high enantioselectivity (92% ee). Unfortunately, this protocol was unfeasible for vinylic C–H functionalization, possibly due to the coordination interaction between olefins and nickel(II).

The robustness of this protocol was further demonstrated by the reaction with a range of symmetrically substituted oxabicyclic alkenes (Figure 3b, **2b** to **2f**), affording the corresponding chiral products with a high level of stereocontrol (87% to 94% ee). In addition, the chiral condensed ring framework **9g-1** could be synthesized in a good yield (80%) and excellent enantioselectivity (94% ee).

**Figure 4:**
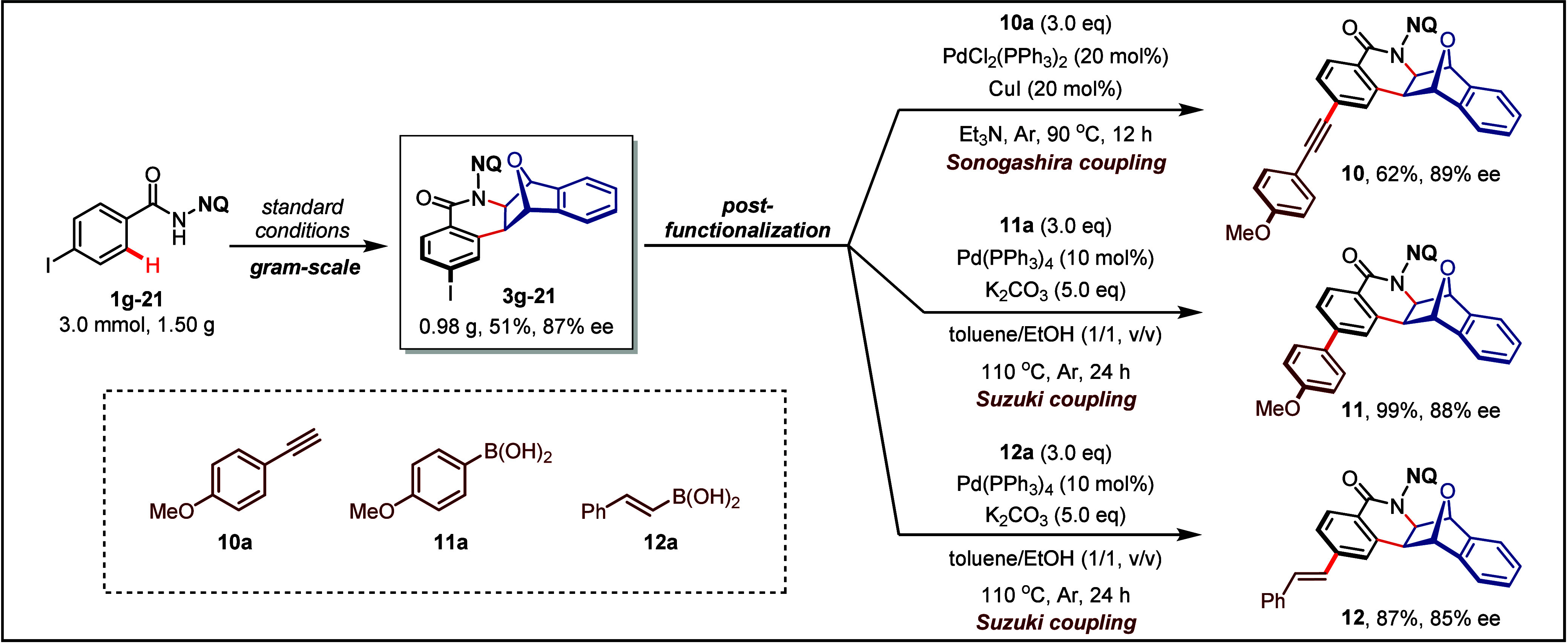
Gram-scale synthesis and postfunctionalization.

To illustrate the practicality and potential applications of this reaction, gram-scale synthesis and subsequent postfunctionalization were conducted (Figure 4). The reaction was successfully carried out on a 3.0 mmol scale, yielding compound **3g-21** in 51% yield with 87% ee. The chiral [2,2,1]-bridged bicyclic compound **3g-21**, which contains an iodide functional group, can be efficiently transformed through Sonogashira coupling (**10**, 62% yield, 89% ee) and Suzuki coupling (**11**, 99% yield, 88% ee;**12**, 87% yield, 85% ee).

**Figure 5:**
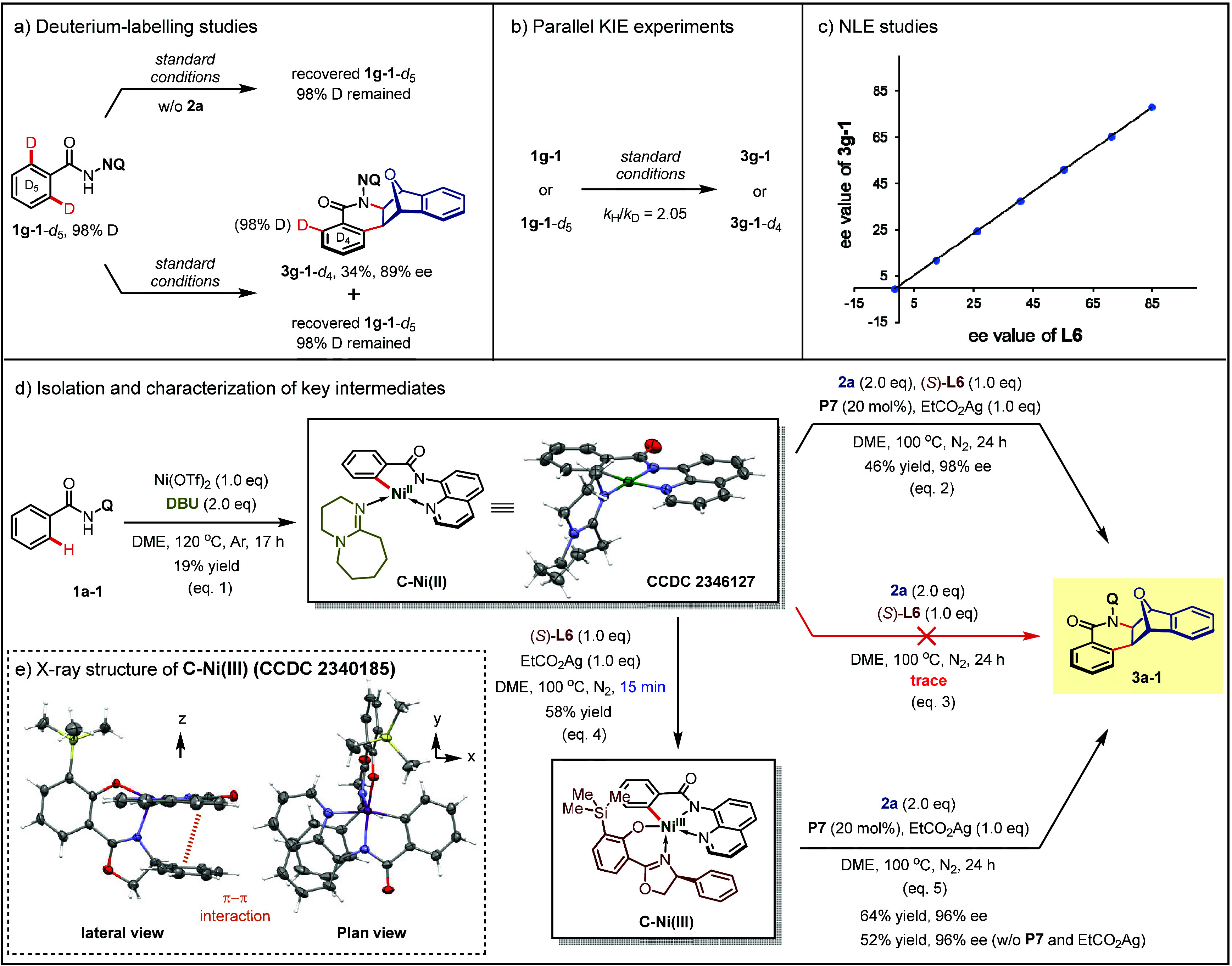
Mechanistic studies. KIE, kinetic isotope effects. NLE, nonlinear effects. DBU, 1,8-diazabicyclo[5.4.0]undec-7-ene.

To shed light on the mechanism of this transformation, a series of experiments have been conducted (Figure 5). First, the deuterium-labeling experiments of **1g-1**-*d*_5_ indicate that the C–H activation step is irreversible (Figure 5a). A parallel KIE experiment, in which the kinetic isotope effect of **1g-1** is determined as 2.05, suggests that C–H cleavage might be involved in the rate-determining step (Figure 5b).^75^

The nonlinear effects between **L6** and **3g-1** were then studied. A plot of ee_**3g-1**_ as a function of ee_**L6**_ was observed to be linear, illustrating a monomeric nickel catalyst with a single bound Salox ligand **L6** (Figure 5c).^76^ Subsequently, the isolation and characterization of nickel intermediates were conducted to elucidate the reaction mechanism (Figure 5d). A tetracoordinate divalent intermediate **C–Ni(II)** was obtained by the stoichiometric reaction of **1a-1** with equimolar Ni(OTf)_2_, using 1,8-diazabicyclo[5.4.0]undec-7-ene (DBU) as a monodentate neutral ligand to stabilize the C–H nickelation complex (eq 1). The absolute configuration of **C–Ni(II)** (CCDC 2346127) was determined by an X-ray analysis. Treatment of **C–Ni(II)** with oxabicyclic alkene **2a** and 1.0 equiv of EtCO_2_Ag as oxidant in the presence of 1.0 equiv of **L6** resulted in the formation of chiral product **3a-1** in 46% yield with 98% ee (eq 2). In contrast, only trace product was observed in the absence of EtCO_2_Ag (eq 3), suggesting the possible involvement of high-valent nickel species followed by the irreversible C–H nickelation step. Motivated by these results, we found that **C–Ni(II)** could be easily and rapidly oxidized by EtCO_2_Ag in the presence of Salox ligand **L6** to generate the trivalent cyclonickelated complex **C–Ni(III)** as a single diastereomer in 58% yield (eq 4). Furthermore, the reaction of **C–Ni(III)** and **2a** gave the desired product **3a-1** in moderate yields with excellent ee values (96% ee), irrespective of the addition of oxidants (eq 5). These results support the formation of **C–Ni(III)** during the reaction and suggest that the tetravalent nickel species might not be involved in the subsequent steps.

The absolute configuration of **C–Ni(III)** (CCDC 2340185) was determined by an X-ray analysis (Figure 5e). The central Ni(III) atom is five-coordinated, adopting a distorted trigonal-bipyramidal geometry, with the chirality of nickel center stereoselectivity determined by the coordination of ligand **L6**. From the lateral view of the crystal structure, a notable π–π stacking interaction was observed between the phenyl group of **L6** and the quinoline moiety. We propose that this secondary interaction plays a crucial role in the diastereoselective arrangement of the substrate and Salox ligand **L6** around the nickel center;^34,46^ from the plan view of the crystal structure, the massive bulk of **C–Ni(III)** is predominantly located in the first and second quadrants. We postulate that by incorporation of a naphthyl group at the 5-position of the directing group, substantial bulk could be generated in the third quadrant. This spatial arrangement would create an open access for alkene to approach the metal center exclusively in the fourth quadrant, thereby determining the stereochemistry.

**Figure 6:**
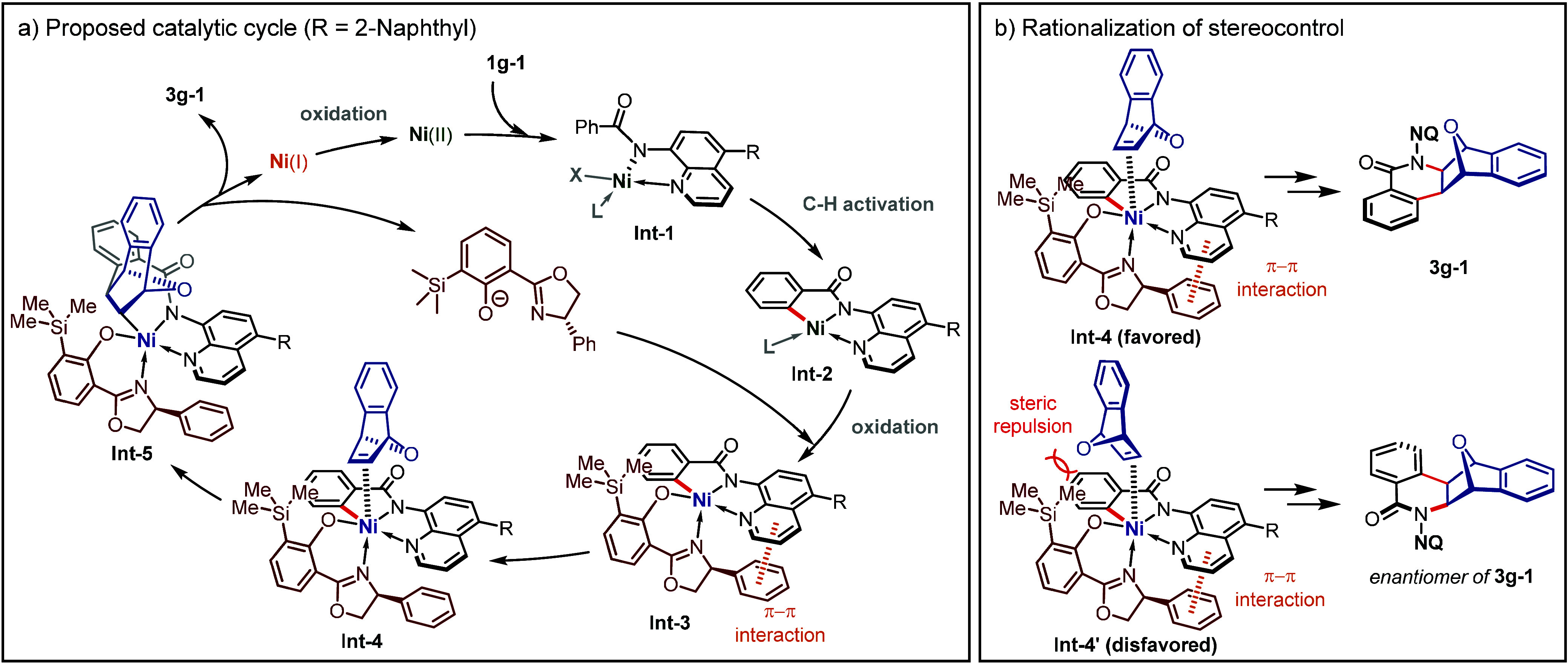
Proposed catalytic cycle and rationalization of stereocontrol.

Based on the above studies, a plausible catalytic cycle was proposed (Figure 6a). The coordination of substrate **1g-1** with a divalent nickel salt initiates the formation of **Int-1**. Subsequent C–H cleavage gives achiral planar Ni(II)-complex **Int-2**.^52−57^ Oxidation of **Int-2**, followed by **L6** coordination, results in the formation of the chiral trivalent intermediate **Int-3**, establishing a chiral binding pocket for alkene. The *exo*-coordination of 7-oxa-benzonorbornadiene **2a** with **Int-3** gives **Int-4**. Guided by the tailored chiral environment of **Int-4**, a C–Ni bond *exo*-migratory insertion leads to the formation of the seven-membered Ni-alkyl intermediate **Int-5**. The desired product **3g-1** is obtained after C–N reductive elimination. The liberated Ni(I) species can be reoxidized to divalent nickel by EtCO_2_Ag, and the Ni(II/III/I) catalytic cycle is closed. In the enantio-determining step (Figure 6b), **Int-4** is energetically favored over **Int-4’** due to the steric repulsion between the bulky TMS group and oxygen bridgehead in **2a**, elucidating the stereochemistry observed for the product.

## Conclusion

In conclusion, we have reported a nickel(II)-catalyzed enantioselective C–H/N–H annulation with oxabicyclic alkenes through a Ni(II/III/I) catalytic pathway. In this protocol, the key chiral trivalent cyclonickelated intermediate was generated through the oxidation of the achiral Ni(II) complex followed by Salox ligand coordination, leaving a tailored chiral pocket for the approach of oxabicyclic alkenes. The sterically hindered chiral Salox ligand, TMS-Salox, is crucial to the success of this transformation. This protocol provides straightforward and step-economic access to a wide variety of chiral [2,2,1]-bridged bicyclic compounds. We expect that the Salox ligands might have broad applications in 3d-transition-metal-catalyzed asymmetric C–H activations.

## Supporting Information Available

Detailed experimental procedures, characterization of new compounds, spectroscopic data and crystallographic data (PDF)

## Accession Codes

CCDC 2340183 (**3d-1**), 2346127 (**C–Ni(II)**) and 2340185 (**C–Ni(III)**) contain the supplementary crystallographic data for this paper. These data can be obtained free of charge via www.ccdc.cam.ac.uk/data_request/cif, or by emailing data_request@ccdc.cam.ac.uk, or by contacting The Cambridge Crystallographic Data Centre, 12 Union Road, Cambridge CB2 1EZ, UK; fax: + 44 1223 336033.

## References

[ref1] Newton C. G.; Wang S.-G.; Oliveira C. C.; Cramer N. Catalytic Enantioselective Transformations Involving C-H Bond Cleavage by Transition-Metal Complexes. Chem. Rev. 2017, 117, 8908–8976. 10.1021/acs.chemrev.6b00692. 28212007

[ref2] Saint-Denis T. G.; Zhu R.-Y.; Chen G.; Wu Q.-F.; Yu J.-Q. Enantioselective C(sp^3^)-H Bond Activation by Chiral Transition Metal Catalysts. Science 2018, 359, eaao4798 10.1126/science.aao4798. 29449462 PMC5862070

[ref3] Liao G.; Zhang T.; Lin Z.-K.; Shi B.-F. Transition Metal-Catalyzed Enantioselective C-H Functionalization via Chiral Transient Directing Group Strategies. Angew. Chem., Int. Ed. 2020, 59, 19773–19786. 10.1002/anie.202008437. 32687690

[ref4] Yoshino T.; Satake S.; Matsunaga S. Diverse Approaches for Enantioselective C-H Functionalization Reactions Using Group 9 Cp^x^M^III^ Catalysts. Chem.-Eur. J. 2020, 26, 7346–7357. 10.1002/chem.201905417. 31994236

[ref5] Achar T. K.; Maiti S.; Jana S.; Maiti D. Transition Metal Catalyzed Enantioselective C(sp^2^)-H Bond Functionalization. ACS Catal. 2020, 10, 13748–13793. 10.1021/acscatal.0c03743.

[ref6] Vyhivskyi O.; Kudashev A.; Miyakoshi T.; Baudoin O. Chiral Catalysts for Pd^0^-Catalyzed Enantioselective C-H Activation. Chem.Eur. J. 2021, 27, 1231–1257. 10.1002/chem.202003225. 32767709

[ref7] Liang H.; Wang J. Enantioselective C-H Bond Functionalization Involving Arene Ruthenium(II) Catalysis. Chem.-Eur. J. 2023, 29, e202202461 10.1002/chem.202202461. 36300688

[ref8] Liu C.-X.; Yin S.-Y.; Zhao F.; Yang H.; Feng Z.; Gu Q.; You S.-L. Rhodium-Catalyzed Asymmetric C-H Functionalization Reactions. Chem. Rev. 2023, 123, 10079–10134. 10.1021/acs.chemrev.3c00149. 37527349

[ref9] Wozniak L.; Cramer N. Enantioselective C-H Bond Functionalizations by 3d Transition-Metal Catalysts. Trends in Chem. 2019, 1, 471–484. 10.1016/j.trechm.2019.03.013.

[ref10] Yoshino T.; Matsunaga S. Cp*Co^III^-Catalyzed C-H Functionalization and Asymmetric Reactions Using External Chiral Sources. Synlett 2019, 30, 1384–1400. 10.1055/s-0037-1611814.

[ref11] Zheng Y.; Zheng C.; Gu Q.; You S.-L. Enantioselective C-H Functionalization Reactions Enabled by Cobalt Catalysis. Chem. Catal. 2022, 2, 2965–2985. 10.1016/j.checat.2022.08.020.

[ref12] Garai B.; Das A.; Kumar D. V.; Sundararaju B. Enantioselective C-H Bond Functionalization Under Co (III)-Catalysis. Chem. Commun. 2024, 60, 3354–3369. 10.1039/D3CC05329F. 38441168

[ref13] Tasker S. Z.; Standley E. A.; Jamison T. F. Recent Advances in Homogeneous Nickel Catalysis. Nature 2014, 509, 299–309. 10.1038/nature13274. 24828188 PMC4344729

[ref14] Diccianni J.; Lin Q.; Diao T. Mechanisms of Nickel-Catalyzed Coupling Reactions and Applications in Alkene Functionalization. Acc. Chem. Res. 2020, 53, 906–919. 10.1021/acs.accounts.0c00032. 32237734 PMC7958188

[ref15] Hu X. Nickel-Catalyzed Cross Coupling of Non-Activated Alkyl Halides: A Mechanistic Perspective. Chem. Sci. 2011, 2, 1867–1886. 10.1039/c1sc00368b.

[ref16] Netherton M. R.; Fu G. C. Nickel-Catalyzed Cross-Couplings of Unactivated Alkyl Halides and Pseudohalides with Organometallic Compounds. Adv. Synth. Catal. 2004, 346, 1525–1532. 10.1002/adsc.200404223.

[ref17] Derosa J.; Apolinar O.; Kang T.; Tran V. T.; Engle K. M. Recent Developments in Nickel-Catalyzed Intermolecular Dicarbofunctionalization of Alkenes. Chem. Sci. 2020, 11, 4287–4296. 10.1039/C9SC06006E. 34122886 PMC8152638

[ref18] Poremba K. E.; Dibrell S. E.; Reisman S. E. Nickel-Catalyzed Enantioselective Reductive Cross-Coupling Reactions. ACS Catal. 2020, 10, 8237–8246. 10.1021/acscatal.0c01842. 32905517 PMC7470226

[ref19] Khake S. M.; Chatani N. Chelation-Assisted Nickel-Catalyzed C-H Functionalizations. Trends in Chem. 2019, 1, 524–539. 10.1016/j.trechm.2019.06.002.

[ref20] Liu Y.-H.; Xia Y.-N.; Shi B.-F. Ni-Catalyzed Chelation-Assisted Direct Functionalization of Inert C-H Bonds. Chin. J. Chem. 2020, 38, 635–662. 10.1002/cjoc.201900468.

[ref21] Donets P. A.; Cramer N. Diaminophosphine Oxide Ligand Enabled Asymmetric Nickel-Catalyzed Hydrocarbamoylations of Alkenes. J. Am. Chem. Soc. 2013, 135, 11772–11775. 10.1021/ja406730t. 23909575

[ref22] Ahlin J. S. E.; Cramer N. Chiral *N*-Heterocyclic Carbene Ligand Enabled Nickel(0)-Catalyzed Enantioselective Three-Component Couplings as Direct Access to Silylated Indanols. Org. Lett. 2016, 18, 3242–3245. 10.1021/acs.orglett.6b01492. 27326716

[ref23] Diesel J.; Finogenova A. M.; Cramer N. Nickel-Catalyzed Enantioselective Pyridone C-H Functionalizations Enabled by a Bulky *N*-Heterocyclic Carbene Ligand. J. Am. Chem. Soc. 2018, 140, 4489–4493. 10.1021/jacs.8b01181. 29543449

[ref24] Wang Y.-X.; Qi S.-L.; Luan Y.-X.; Han X.-W.; Wang S.; Chen H.; Ye M. Enantioselective Ni-Al Bimetallic Catalyzed *exo*-Selective C-H Cyclization of Imidazoles with Alkenes. J. Am. Chem. Soc. 2018, 140, 5360–5364. 10.1021/jacs.8b02547. 29641189

[ref25] Diesel J.; Grosheva D.; Kodama S.; Cramer N. A Bulky Chiral *N*-Heterocyclic Carbene Nickel Catalyst Enables Enantioselective C-H Functionalizations of Indoles and Pyrroles. Angew. Chem., Int. Ed. 2019, 58, 11044–11048. 10.1002/anie.201904774. 31095857

[ref26] Zhang W.-B.; Yang X.-T.; Ma J.-B.; Su Z.-M.; Shi S.-L. Regio- and Enantioselective C-H Cyclization of Pyridines with Alkenes Enabled by a Nickel/*N*-Heterocyclic Carbene Catalysis. J. Am. Chem. Soc. 2019, 141, 5628–5634. 10.1021/jacs.9b00931. 30888167

[ref27] Loup J.; Mueller V.; Ghorai D.; Ackermann L. Enantioselective Aluminum-Free Alkene Hydroarylations through C-H Activation by a Chiral Nickel/JoSPOphos Manifold. Angew. Chem., Int. Ed. 2019, 58, 1749–1753. 10.1002/anie.201813191. 30517772

[ref28] Li J.-F.; Pan D.; Wang H.-R.; Zhang T.; Li Y.; Huang G.; Ye M. Enantioselective C2-H Alkylation of Pyridines with 1,3-Dienes via Ni-Al Bimetallic Catalysis. J. Am. Chem. Soc. 2022, 144, 18810–18816. 10.1021/jacs.2c09306. 36205623

[ref29] Hyster T. K.; Knoerr L.; Ward T. R.; Rovis T. Biotinylated Rh(III) Complexes in Engineered Streptavidin for Accelerated Asymmetric C-H Activation. Science 2012, 338, 500–503. 10.1126/science.1226132. 23112327 PMC3820005

[ref30] Ye B.; Cramer N. Chiral Cyclopentadienyl Ligands as Stereocontrolling Element in Asymmetric C-H Functionalization. Science 2012, 338, 504–506. 10.1126/science.1226938. 23112328

[ref31] Lee P.-S.; Yoshikai N. Cobalt-Catalyzed Enantioselective Directed C-H Alkylation of Indole with Styrenes. Org. Lett. 2015, 17, 22–25. 10.1021/ol503119z. 25514474

[ref32] Chen S.-S.; Wu M.-S.; Han Z.-Y. Palladium-Catalyzed Cascade sp^2^ C-H Functionalization/Intramolecular Asymmetric Allylation: From Aryl Ureas and 1,3-Dienes to Chiral Indolines. Angew. Chem., Int. Ed. 2017, 56, 6641–6645. 10.1002/anie.201702745. 28467624

[ref33] Ozols K.; Jang Y.-S.; Cramer N. Chiral Cyclopentadienyl Cobalt(III) Complexes Enable Highly Enantioselective 3d-Metal-Catalyzed C-H Functionalizations. J. Am. Chem. Soc. 2019, 141, 5675–5680. 10.1021/jacs.9b02569. 30901216

[ref34] Yao Q.-J.; Huang F.-R.; Chen J.-H.; Zhong M.-Y.; Shi B.-F. Enantio- and Regioselective Electrooxidative Cobalt-Catalyzed C-H/N-H Annulation with Alkenes. Angew. Chem., Int. Ed. 2023, 62, e202218533 10.1002/anie.202218533. 36658097

[ref35] Aihara Y.; Chatani N. Nickel-Catalyzed Direct Alkylation of C-H Bonds in Benzamides and Acrylamides with Functionalized Alkyl Halides via Bidentate-Chelation Assistance. J. Am. Chem. Soc. 2013, 135, 5308–5311. 10.1021/ja401344e. 23495861

[ref36] Aihara Y.; Chatani N. Nickel-Catalyzed Direct Arylation of C(sp^3^)-H Bonds in Aliphatic Amides via Bidentate-Chelation Assistance. J. Am. Chem. Soc. 2014, 136, 898–901. 10.1021/ja411715v. 24377655

[ref37] Wu X.; Zhao Y.; Ge H. Nickel-Catalyzed Site-Selective Alkylation of Unactivated C(sp^3^)-H Bonds. J. Am. Chem. Soc. 2014, 136, 1789–1792. 10.1021/ja413131m. 24446698

[ref38] Li M.; Dong J.; Huang X.; Li K.; Wu Q.; Song F.; You J. Nickel-Catalyzed Chelation-Assisted Direct Arylation of Unactivated C(sp^3^)-H Bonds with Aryl Halides. Chem. Commun. 2014, 50, 3944–3946. 10.1039/C4CC00716F. 24599346

[ref39] Liu Y.-J.; Liu Y.-H.; Yan S.-Y.; Shi B.-F. A Sustainable and Simple Catalytic System for Direct Alkynylation of C(sp^2^)-H Bonds with Low Nickel Loadings. Chem. Commun. 2015, 51, 6388–6391. 10.1039/C5CC01163A. 25762446

[ref40] Liu Y.-J.; Zhang Z.-Z.; Yan S.-Y.; Liu Y.-H.; Shi B.-F. Ni(II)/BINOL-Catalyzed Alkenylation of Unactivated C(sp^3^)-H bonds. Chem. Commun. 2015, 51, 7899–7902. 10.1039/C5CC02254A. 25857332

[ref41] Zaitsev V. G.; Shabashov D.; Daugulis O. Highly Regioselective Arylation of sp^3^ C-H Bonds Catalyzed by Palladium Acetate. J. Am. Chem. Soc. 2005, 127, 13154–13155. 10.1021/ja054549f. 16173737

[ref42] Daugulis O.; Roane J.; Tran L. D. Bidentate, Monoanionic Auxiliary-Directed Functionalization of Carbon-Hydrogen Bonds. Acc. Chem. Res. 2015, 48, 1053–1064. 10.1021/ar5004626. 25756616 PMC4406856

[ref43] Rej S.; Ano Y.; Chatani N. Bidentate Directing Groups: An Efficient Tool in C-H Bond Functionalization Chemistry for the Expedient Construction of C-C Bonds. Chem. Rev. 2020, 120, 1788–1887. 10.1021/acs.chemrev.9b00495. 31904219

[ref44] Yao Q.-J.; Huang F.-R.; Chen J.-H.; Shi B.-F. Nickel(II)/BINOL-catalyzed enantioselective C-H activation via desymmetrization and kinetic resolution. Nat. Commun. 2024, 15, 7135 10.1038/s41467-024-51409-3. 39164290 PMC11336223

[ref45] Yuan W.-K.; Shi B.-F. Synthesis of Chiral Spirolactams via Sequential C-H Olefination/Asymmetric [4 + 1] Spirocyclization under a Simple Co^II^/Chiral Spiro Phosphoric Acid Binary System. Angew. Chem., Int. Ed. 2021, 60, 23187–23192. 10.1002/anie.202108853. 34435722

[ref46] Yao Q.-J.; Chen J.-H.; Song H.; Huang F.-R.; Shi B.-F. Cobalt/Salox-Catalyzed Enantioselective C-H Functionalization of Arylphosphinamides. Angew. Chem., Int. Ed. 2022, 61, e202202892 10.1002/anie.202202892. 35385597

[ref47] Wang B.-J.; Xu G.-X.; Huang Z.-W.; Wu X.; Hong X.; Yao Q.-J.; Shi B.-F. Single-Step Synthesis of Atropisomers with Vicinal C-C and C-N Diaxes by Cobalt-Catalyzed Atroposelective C-H Annulation. Angew. Chem., Int. Ed. 2022, 61, e202208912 10.1002/anie.202208912. 35917381

[ref48] Wu Y.-J.; Chen J.-H.; Teng M.-Y.; Li X.; Jiang T.-Y.; Huang F.-R.; Yao Q.-J.; Shi B.-F. Cobalt-Catalyzed Enantioselective C-H Annulation of Benzylamines with Alkynes: Application to the Modular and Asymmetric Syntheses of Bioactive Molecules. J. Am. Chem. Soc. 2023, 145, 24499–24505. 10.1021/jacs.3c10714. 38104268

[ref49] Huang F.-R.; Yao Q.-J.; Zhang P.; Teng M.-Y.; Chen J.-H.; Jiang L.-C.; Shi B.-F. Cobalt-Catalyzed Domino Transformations via Enantioselective C-H Activation/Nucleophilic [3 + 2] Annulation Towards Chiral Bridged Bicycles. J. Am. Chem. Soc. 2024, 146, 15576–15586. 10.1021/jacs.4c04623. 38753821

[ref50] Teng M.-Y.; Wu Y.-J.; Chen J.-H.; Huang F.-R.; Liu D.-Y.; Yao Q.-J.; Shi B.-F. Cobalt-Catalyzed Enantioselective C-H Carbonylation towards Chiral Isoindolinones. Angew. Chem., Int. Ed. 2024, 63, e202318803 10.1002/anie.202318803. 38205884

[ref51] Qian P.-F.; Zhou G.; Hu J.-H.; Wang B.-J.; Jiang A.-L.; Zhou T.; Yuan W.-K.; Yao Q.-J.; Chen J.-H.; Kong K.-X.; Shi B.-F. Asymmetric Synthesis of Chiral Calix[4]arenes with Both Inherent and Axial Chirality via Cobalt-Catalyzed Enantioselective Intermolecular C-H Annulation. Angew. Chem. Int. Int. 2024, 63, e202412459 10.1002/anie.202412459. 39261278

[ref52] Roy P.; Bour J. R.; Kampf J. W.; Sanford M. S. Catalytically Relevant Intermediates in the Ni-Catalyzed C(sp^2^)-H and C(sp^3^)-H Functionalization of Aminoquinoline Substrates. J. Am. Chem. Soc. 2019, 141, 17382–17387. 10.1021/jacs.9b09109. 31618019

[ref53] Beattie D. D.; Grunwald A. C.; Perse T.; Schafer L. L.; Love J. A. Understanding Ni(II)-Mediated C(sp^3^)-H Activation: Tertiary Ureas as Model Substrates. J. Am. Chem. Soc. 2018, 140, 12602–12610. 10.1021/jacs.8b07708. 30185028

[ref54] Liu J.; Johnson S. A. Mechanism of 8-Aminoquinoline-Directed Ni-Catalyzed C(sp^3^)-H Functionalization: Paramagnetic Ni(II) Species and the Deleterious Effect of Carbonate as a Base. Organometallics 2021, 40, 2970–2982. 10.1021/acs.organomet.1c00265.

[ref55] Tang H.; Huang X.-R.; Yao J.; Chen H. Understanding the Effects of Bidentate Directing Groups: A Unified Rationale for sp^2^ and sp^3^ C-H Bond Activations. J. Org. Chem. 2015, 80, 4672–4682. 10.1021/acs.joc.5b00580. 25836059

[ref56] Haines B. E.; Yu J.-Q.; Musaev D. G. The Mechanism of Directed Ni(II)-Catalyzed C-H Iodination with Molecular Iodine. Chem. Sci. 2018, 9, 1144–1154. 10.1039/C7SC04604A. 29675159 PMC5883947

[ref57] Li Y.; Zou L.; Bai R.; Lan Y. Ni(I)-Ni(III) *vs*. Ni(II)-Ni(IV): Mechanistic Study of Ni-Ccatalyzed Alkylation of Benzamides with Alkyl Halides. Org. Chem. Front. 2018, 5, 615–622. 10.1039/C7QO00850C.

[ref58] Bauer E. B. Chiral-at-Metal Complexes and Their Catalytic Applications in Organic Synthesis. Chem. Soc. Rev. 2012, 41, 3153–3167. 10.1039/c2cs15234g. 22306968

[ref59] Si X.-J.; Yang D.; Sun M.-C.; Wei D.; Song M.-P.; Niu J.-L. Atroposelective Isoquinolinone Synthesis through Cobalt-Catalysed C-H Activation and Annulation. Nat. Synth. 2022, 1, 709–718. 10.1038/s44160-022-00114-4.

[ref60] Li T.; Shi L.; Wang X.; Yang C.; Yang D.; Song M.-P.; Niu J.-L. Cobalt-Catalyzed Atroposelective C-H Activation/Annulation to Access N-N Axially Chiral Frameworks. Nat. Commun. 2023, 14, 5271 10.1038/s41467-023-40978-4. 37644016 PMC10465517

[ref61] von Münchow T.; Dana S.; Xu Y.; Yuan B.; Ackermann L. Enantioselective electrochemical cobalt-catalyzed aryl C-H activation reactions. Science 2023, 379, 1036–1042. 10.1126/science.adg2866. 36893225

[ref62] Das A.; Mandal R.; Ravi Sankar H. S.; Kumaran S.; Premkumar J. R.; Borah D.; Sundararaju B. Reversal of Regioselectivity in Asymmetric C-H Bond Annulation with Bromoalkynes under Cobalt Catalysis. Angew. Chem., Int. Ed. 2024, 63, e202315005 10.1002/anie.202315005. 38095350

[ref63] Ackermann L.; Münchow T.; Pandit N.; Dana S.; Boos P.; Peters S.; Boucat J.; Liu Y.-R.; Scheremetjew A. Nickel-electrocatalyzed enantioselective C-H activations for chemo-divergence. Preprint in Research Square 2024, 10.21203/rs.3.rs-3760859/v1.

[ref64] Vivek Kumar S.; Yen A.; Lautens M.; Guiry P. J. Catalytic Asymmetric Transformations of Oxa- and Azabicyclic Alkenes. Chem. Soc. Rev. 2021, 50, 3013–3093. 10.1039/D0CS00702A. 33544110

[ref65] Khan R.; Chen J.; Fan B. Versatile Catalytic Reactions of Norbornadiene Derivatives with Alkynes. Adv. Synth. Catal. 2020, 362, 1564–1601. 10.1002/adsc.201901494.

[ref66] Wang F.; Yu S.; Li X. Transition Metal-Catalysed Couplings between Arenes and Strained or Reactive Rings: Combination of C-H Activation and Ring Scission. Chem. Soc. Rev. 2016, 45, 6462–6477. 10.1039/C6CS00371K. 27711636

[ref67] Rayabarapu D. K.; Cheng C.-H. New Catalytic Reactions of Oxa- and Azabicyclic Alkenes. Acc. Chem. Res. 2007, 40, 971–983. 10.1021/ar600021z. 17591745

[ref68] Lautens M.; Fagnou K.; Hiebert S. Transition Metal-Catalyzed Enantioselective Ring-Opening Reactions of Oxabicyclic Alkenes. Acc. Chem. Res. 2003, 36, 48–58. 10.1021/ar010112a. 12534304

[ref69] Yang X.; Zheng G.; Li X. Rhodium(III)-Catalyzed Enantioselective Coupling of Indoles and 7-Azabenzonorbornadienes by C-H Activation/Desymmetrization. Angew. Chem., Int. Ed. 2019, 58, 322–326. 10.1002/anie.201811998. 30450800

[ref70] Mi R.; Zheng G.; Qi Z.; Li X. Rhodium-Catalyzed Enantioselective Oxidative [3 + 2] Annulation of Arenes and Azabicyclic Olefins through Twofold C-H Activation. Angew. Chem., Int. Ed. 2019, 58, 17666–17670. 10.1002/anie.201911086. 31549764

[ref71] Liao G.; Chen H.-M.; Xia Y.-N.; Li B.; Yao Q.-J.; Shi B.-F. Synthesis of Chiral Aldehyde Catalysts by Pd-Catalyzed Atroposelective C-H Naphthylation. Angew. Chem., Int. Ed. 2019, 58, 11464–11468. 10.1002/anie.201906700. 31190443

[ref72] Trifonova E. A.; Ankudinov N. M.; Mikhaylov A. A.; Chusov D. A.; Nelyubina Y. V.; Perekalin D. S. A Planar-Chiral Rhodium(III) Catalyst with a Sterically Demanding Cyclopentadienyl Ligand and Its Application in the Enantioselective Synthesis of Dihydroisoquinolones. Angew. Chem., Int. Ed. 2018, 57, 7714–7718. 10.1002/anie.201801703. 29624840

[ref73] Brandes D. S.; Sirvent A.; Mercado B. Q.; Ellman J. A. Three-Component 1,2-Carboamidation of Bridged Bicyclic Alkenes via Rh^III^-Catalyzed Addition of C-H Bonds and Amidating Reagents. Org. Lett. 2021, 23, 2836–2840. 10.1021/acs.orglett.1c00851. 33739839 PMC8026749

[ref74] Zheng Y.; Zhang W.-Y.; Gu Q.; Zheng C.; You S.-L. Cobalt(III)-Catalyzed Asymmetric Ring-Opening of 7-Oxabenzonorbornadienes via Indole C-H Functionalization. Nat. Commun. 2023, 14, 1094 10.1038/s41467-023-36723-6. 36841798 PMC9968317

[ref75] Simmons E. M.; Hartwig J. F. On the Interpretation of Deuterium Kinetic Isotope Effects in C-H Bond Functionalizations by Transition-Metal Complexes. Angew. Chem., Int. Ed. 2012, 51, 3066–3072. 10.1002/anie.201107334. 22392731

[ref76] Satyanarayana T.; Abraham S.; Kagan H. B. Nonlinear Effects in Asymmetric Catalysis. Angew. Chem., Int. Ed. 2009, 48, 456–494. 10.1002/anie.200705241. 19115268

