# Supplementary material for: Nickel(II)/Salox-Catalyzed Enantioselective C–H Functionalization
Source: ACS Cent Sci. 2025 Jan 2;11(1):127–35. doi: 10.1021/acscentsci.4c02049 (PMC11758223; doi:10.1021/acscentsci.4c02049)

Supporting Information

Nickel(II)/Salox-Catalyzed Enantioselective C−H Functionalization

Jia-Hao Chen,[†,1] Qi-Jun Yao,[†,1] Ming-Yu Zhong,[1] Tian-Yu Jiang,[1] Fan-Rui Huang,[1] Xiang Li,[1] Bing-Feng Shi*,[1,2,3]

[1]Department of Chemistry, Zhejiang University, Hangzhou 310058, China

[2]School of Chemistry and Chemical Engineering, Henan Normal University, Xinxiang 453007, China

[3]College of Material Chemistry and Chemical Engineering, Key Laboratory of Organosilicon Chemistry and Material Technology, Ministry of Education, Hangzhou Normal University, Hangzhou 311121, China

[†]These authors contributed equally to this work

*Email: bfshi@zju.edu.cn

Table of Contents

1. General Information **3**

2. Synthesis of Substrates **4**

2.1 Summary of benzamides 1 and bicyclic alkenes 2 4

2.2 Synthesis and characterization of substrates 1g-1 – 1j-1 5

2.3 Synthesis and characterization of 8-aminoquinoline (NQ) 7

2.4 Synthesis and characterization of substrates 1g-2 – 1g-28 8

2.6 Synthesis and characterization of ligands (*S*)-L6 – (*S*)-L8 16

3. General Procedure for Nickel(II)/Salox-Catalyzed Enantioselective C−H Functionalization **18**

3.1 Optimization of reaction conditions 18

3.2 Reaction procedure and characterization for Nickel(II)/Salox-catalyzed C−H activation/asymmetric alkene insertion reaction 24

3.3 Gram-scale synthesis and post-functionalization 66

4. Mechanistic Studies **70**

4.1 Experiments of nonlinear effects (NLEs) 70

4.2 Deuterium-labeling studies 71

4.3 Parallel KIE experiments with 1g-1 and 1g-1-d_5 75

4.4 Synthesis and Characterization of C-Ni(II) and C-Ni(III) intermediates 76

References **78**

X-Ray Crystallographic Data **79**

NMR Spectra **84**

1. General Information

Unless otherwise noted, all the materials and solvents were purchased from commercial suppliers and used without additional purification. NMR spectra were recorded on a Bruke Avance operating for ^{1}H NMR at 400 MHz, ^{13}C NMR at 101 MHz, and ^{19}F NMR at 376 MHz, using TMS as internal standard. The peaks were internally referenced to TMS (0.00 ppm) or residual undeuterated solvent signal (77.16 ppm for ^{13}C NMR). The following abbreviations (or combinations thereof) were used to explain multiplicities: s = singlet, d = doublet, t = triplet, m = multiplet, br = broad. Mass spectroscopy data of the products were collected on an HRMS-TOF using ESI ionization. High pressure liquid chromatography (HPLC) analyses were performed on a Shimadzu instrument using a chiral stationary phase column (Daicel Co. CHIRALPAK). The chiral HPLC methods were calibrated with the corresponding racemic mixtures. Elemental analyses were performed on UNICUBE®-Elementar.

2. Synthesis of Substrates

2.1 Summary of benzamides 1 and bicyclic alkenes 2

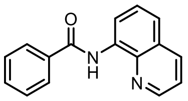

1a-1

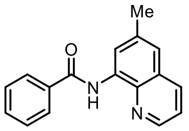

1b-1

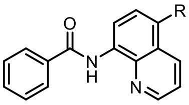

1c-1, R = Me
1d-1, R = Ph
1e-1, R = Cl
1f-1, R = OMe
1g-1, R = 2-Napth
1h-1, R = 4-OMeC_6H_4
1i-1, R = 3,5-$Me_2C_6H_3$
1j-1, R = 3,5-$Ph_2C_6H_3$

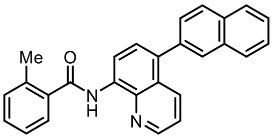

1g-2

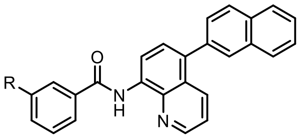

1g-3, R = Me
1g-6, R = *i*-Pr
1g-8, R = *t*-Bu
1g-18, R = Br
1g-20, R = I
1g-12, R = SMe
1g-24, R = CO_2Me
1g-22, R = Ac

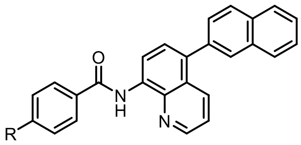

1g-4, R = Me
1g-5, R = Et
1g-7, R = *i*-Pr
1g-9, R = *t*-Bu
1g-16, R = F
1g-17, R = Cl
1g-19, R = Br
1g-21, R = I
1g-10, R = OMe
1g-13, R = SMe
1g-25, R = CO_2Me
1g-23, R = Ac
1g-14, R = CF_3
1g-15, R = OCF_3
1g-26, R = CN

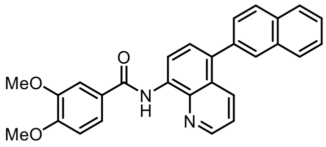

1g-11

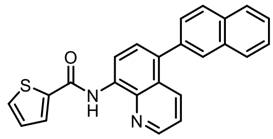

1g-27

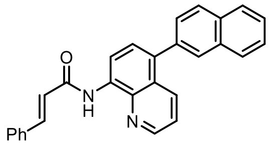

1g-28

1a-1 – **1f-1** were known compounds that were prepared following reported literatures.[1-3] **1g-1** – **1g-28** were synthesized according to following procedures.

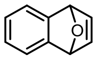

2a

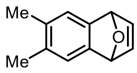

2b

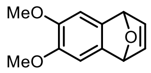

2c

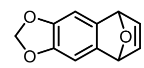

2d

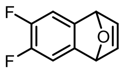

2e

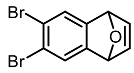

2f

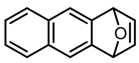

2g

2a – **2g** were known compounds that were prepared following reported literatures.[4-5]

2.2 Synthesis and characterization of substrates 1g-1 – 1j-1

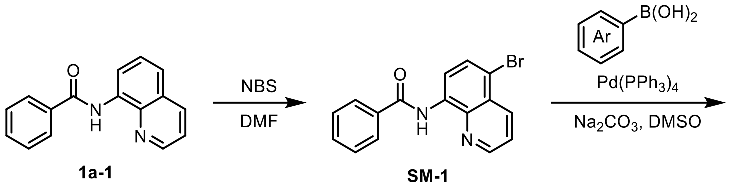

Step 1: To a 100 mL three-necked flask was added amide **1a-1** (3.72 g, 15 mmol), NBS (2.94 g, 16.5 mmol, 1.1 eq) and DMF (20 mL). Then the mixture was stirred at 50 °C for 4 hours under N_2 atmosphere. After the reaction was completed, the reaction mixture was quenched with water (100 mL) and extracted with Et_2O (3×40 mL). The combined organic layer was washed with brine, dried over anhydrous Na_2SO_4, and concentrated in vacuo. The resulting residue was purified by flash chromatography on silica gel in PE/EtOAc = 9/1 (v/v, R_f = 0.53) to afford the desired brominated amide **SM-1**.

Step 2: To a 50 mL Schlenk tube was added **SM-1** (978 mg, 3.0 mmol), aryl boronic acid (6.0 mmol, 2.0 eq), $Pd(PPh_3)_4$ (346.8 mg, 10 mol%), Na_2CO_3 (636 mg, 6.0 mmol, 2eq) and DMSO (10 mL). Then the mixture was stirred at 140 °C for 12 hours under N_2 atmosphere. After the reaction was completed, the reaction mixture was quenched with water (100 mL) and extracted with DCM (3×30 mL). The combined organic layer was washed with brine, dried over anhydrous Na_2SO_4, and concentrated in vacuo. The resulting residue was purified by flash chromatography on silica gel in PE/EtOAc to afford the desired amides **1**.

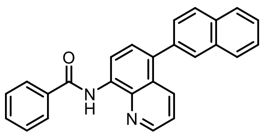

1g-1: A purification by flash chromatography in PE/EtOAc = 30/1 (v/v, R_f = 0.17) gave **1g-1** as light-yellow solid (1.06 g, 94%). **^{1}H NMR (400 MHz, CDCl$_3$)** δ 10.89 (s, 1H), 9.05 (d, *J* = 8.0 Hz, 1H), 8.88 (dd, *J* = 4.2, 1.6 Hz, 1H), 8.35 (dd, *J* = 8.5, 1.7 Hz, 1H), 8.14 (dd, *J* = 7.8, 1.8 Hz, 2H), 8.01 – 7.87 (m, 4H), 7.67 (d, *J* = 7.9 Hz, 1H), 7.62 – 7.51 (m, 6H), 7.43 (dd, *J* = 8.5, 4.1 Hz, 1H); **^{13}C NMR (101 MHz, CDCl$_3$)** δ 165.5, 148.2, 138.9, 136.7, 135.2, 134.9, 134.4, 134.2, 133.5, 132.7, 131.9, 129.0, 128.9, 128.4, 128.3, 128.1, 127.8, 127.4, 126.6, 126.3, 121.8, 116.3; **HRMS (ESI)** calcd for $C_{26}H_{18}N_2O$ $[M+H]^+$: 375.1492, Found: 375.1494.

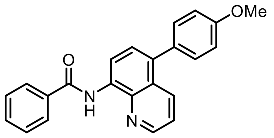

1h-1: A purification by flash chromatography in PE/DCM/EtOAc = 75/25/1 (v/v, R_f = 0.21) gave **1h-1** as light-yellow foam (1.02 g, 96%). **^{1}H NMR (400 MHz, CDCl$_3$)** δ 10.84 (s, 1H), 8.98 (d, *J* = 7.9 Hz, 1H), 8.85 (dd, *J* = 4.2, 1.6 Hz, 1H), 8.32 (dd, *J* = 8.6, 1.6 Hz, 1H), 8.11 (dd, *J* = 7.9, 1.8 Hz, 2H), 7.64 – 7.51 (m, 4H), 7.47 – 7.35 (m, 3H), 7.07 – 7.01 (m, 2H), 3.89 (s, 3H); **^{13}C NMR (101 MHz, CDCl$_3$)** δ 165.5, 159.2, 148.1, 138.9, 135.3, 135.0, 134.3, 133.8, 131.9, 131.6, 131.2, 128.9, 127.9, 127.4, 126.6, 121.6, 116.3, 114.0, 55.5; **HRMS (ESI)** calcd for $C_{23}H_{18}N_2O_2$ $[M+H]^+$: 355.1441, Found: 355.1443.

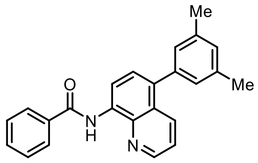

1i-1: A purification by flash chromatography in PE/EtOAc = 30/1 (v/v, R_f = 0.23) gave **1i-1** as white foam (0.77 g, 73%). **^{1}H NMR (400 MHz, CDCl$_3$)** δ 10.86 (s, 1H), 8.99 (d, *J* = 7.9 Hz, 1H), 8.86 (dd, *J* = 4.1, 1.7 Hz, 1H), 8.35 (dd, *J* = 8.5, 1.7 Hz, 1H), 8.12 (dd, *J* = 7.8, 1.8 Hz, 2H), 7.64 – 7.51 (m, 4H), 7.44 (dd, *J* = 8.5, 4.2 Hz, 1H), 7.09 (s, 3H), 2.42 (s, 6H); **^{13}C NMR (101 MHz, CDCl$_3$)** δ 165.5, 148.1, 139.2, 138.9, 138.1, 135.3, 135.1, 134.9, 133.8, 131.9, 129.2, 128.9, 128.0, 127.9, 127.4, 126.5, 121.6, 116.2, 21.5; **HRMS (ESI)** calcd for $C_{24}H_{20}N_2O$ [M+H]$^+$: 353.1648, Found: 353.1652.

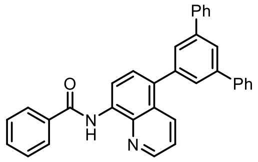

1j-1: A purification by flash chromatography in PE/DCM = 6/1 with 1% EtOAc (v/v, R_f = 0.33) gave **1j-1** as white foam (1.06 g, 74%). **^{1}H NMR (400 MHz, CDCl$_3$)** δ 10.91 (s, 1H), 9.07 (d, *J* = 7.9 Hz, 1H), 8.90 (dd, *J* = 4.2, 1.6 Hz, 1H), 8.45 (dd, *J* = 8.6, 1.7 Hz, 1H), 8.15 (dd, *J* = 7.5, 2.0 Hz, 2H), 7.92 (s, 1H), 7.78 – 7.65 (m, 7H), 7.66 – 7.54 (m, 3H), 7.54 – 7.45 (m, 5H), 7.41 (t, *J* = 7.4 Hz, 2H); **^{13}C NMR (101 MHz, CDCl$_3$)** δ 165.5, 148.3, 142.2, 140.9, 140.3, 138.9, 135.2, 134.9, 134.3, 132.0, 129.0, 128.9, 128.2, 128.0, 127.8, 127.4, 126.5, 125.4, 121.9, 116.3; **HRMS (ESI)** calcd for $C_{34}H_{24}N_2O$ [M+H]$^+$: 477.1961, Found: 477.1964.

2.3 Synthesis and characterization of 8-aminoquinoline (NQ)

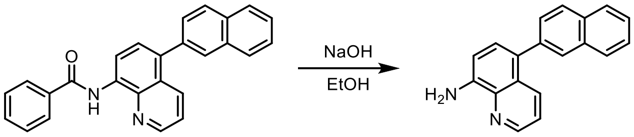

To a 10 mL Schlenk tube was added **1g-1** (74.8 mg, 0.20 mmol) and NaOH (40 mg, 1.0 mmol, 5eq), followed by addition of EtOH (0.5 mL). Then the mixture was stirred at 120 °C for 12 h. After cooling to room temperature, the reaction system was quenched with water (10 mL) and extracted with CH_2Cl_2 (3×15 mL). The combined organic layer was dried over anhydrous Na_2SO_4, filtered, and concentrated in vacuo. After concentration, the crude product was purified by preparative TLC (PE/EtOAc = 20/1, v/v, R_f = 0.09) to afford **NQ** (52.5 mg, 97% yield) as a yellow solid.

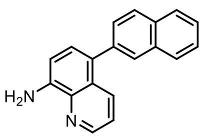

^{1}H NMR (400 MHz, CDCl$_3$) δ 8.81 (d, *J* = 4.1 Hz, 1H), 8.28 (d, *J* = 8.5 Hz, 1H), 7.95 – 7.88 (m, 4H), 7.60 (d, *J* = 8.4 Hz, 1H), 7.57 – 7.51 (m, 2H), 7.43 (d, *J* = 7.7 Hz, 1H), 7.34 (dd, *J* = 8.6, 4.1 Hz, 1H), 7.03 (d, *J* = 7.8 Hz, 1H), 5.13 (s, 2H); **^{13}C NMR (101 MHz, CDCl$_3$)** δ 147.5, 143.7, 138.5, 137.7, 134.5, 133.7, 132.5, 128.9, 128.8, 128.6, 128.1, 128.0, 127.9, 127.2, 126.4, 126.0, 121.6, 109.7; **HRMS (ESI)** calcd for $C_{19}H_{14}N_2$ $[M+H]^+$: 271.1230, Found: 271.1230.

2.4 Synthesis and characterization of substrates 1g-2 – 1g-28

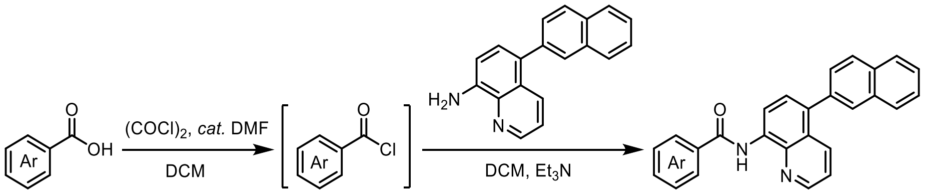

Step 1: To a mixture of benzoic acid (3.0 mmol) and DMF (*cat.*, several drops) in anhydrous DCM (15 mL) was slowly added oxalyl chloride (381 μL, 4.5 mmol, 1.5 eq) at 0 °C, and stirred at room temperature for 3 hours. After the reaction was completed, the solvent was removed in vacuo and the resulting acid chloride was used immediately without further purification.

Step 2: To an oven-dried 50 mL three-necked flask, 8-aminoquinoline (**NQ**, 405 mg, 1.5 mmol, 0.5 eq), Et_3N (835 μL, 6.0 mmol, 2.0 eq) and anhydrous DCM (15 mL) were added. A solution of above crude acid chloride or commercially available acid chloride in anhydrous DCM (10 mL) was added dropwise to the mixture at 0 °C, and the solution was stirred at room temperature for overnight. After the reaction was completed, the reaction mixture was quenched with $NaHCO_3$ (50 mL, sat. aq.) and extracted with DCM (3×20 mL). The combined organic layer was washed with brine, dried over anhydrous Na_2SO_4, and concentrated in vacuo. The resulting residue was purified by recrystallization or flash chromatography on silica gel in PE/EtOAc to afford the desired amides **1**.

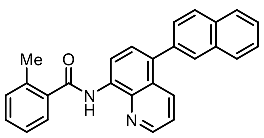

1g-2: A purification by flash chromatography in PE/DCM = 1/1 (v/v, R_f = 0.15) gave **1g-2** as white foam (0.72 g, 93%). **^{1}H NMR (400 MHz, CDCl$_3$)** δ 10.34 (s, 1H), 9.04 (d, *J* = 7.9 Hz, 1H), 8.81 (dd, *J* = 4.1, 1.7 Hz, 1H), 8.35 (dd, *J* = 8.5, 1.7 Hz, 1H), 7.98 (d, *J* = 8.4 Hz, 1H), 7.96 – 7.88 (m, 3H), 7.73 (dd, *J* = 7.5, 1.6 Hz, 1H), 7.68 (d, *J* = 7.9 Hz, 1H), 7.61 (dd, *J* = 8.4, 1.7 Hz, 1H), 7.59 – 7.53 (m, 2H), 7.47 – 7.39 (m, 2H), 7.35 (t, *J* = 7.7 Hz, 2H), 2.65 (s, 3H); **^{13}C NMR (101 MHz, CDCl$_3$)** δ 168.3, 148.2, 138.8, 136.8, 136.8, 134.9, 134.5, 134.3, 133.5, 132.7, 131.5, 130.4, 129.0, 128.3, 128.1, 127.8, 127.4, 126.6, 126.3, 126.1, 121.7, 116.2, 20.3; **HRMS (ESI)** calcd for $C_{27}H_{20}N_2O$ $[M+H]^+$: 389.1648, Found: 389.1651.

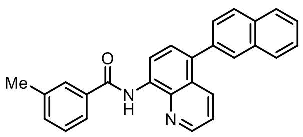

1g-3: A purification by recrystallization in DCM/EtOH gave **1g-3** as light-yellow foam (0.31 g, 40%). **^{1}H NMR (400 MHz, CDCl$_3$)** δ 10.85 (s, 1H), 9.04 (d, *J* = 8.0 Hz, 1H), 8.90 (dd, *J* = 4.2, 1.7 Hz, 1H), 8.36 (dd, *J* = 8.5, 1.7 Hz, 1H), 8.04 – 7.86 (m, 6H), 7.68 (d, *J* = 8.0 Hz, 1H), 7.61 (dd, *J* = 8.3, 1.7 Hz, 1H), 7.59 – 7.53 (m, 2H), 7.49 – 7.39 (m, 3H), 2.51 (s, 3H); **^{13}C NMR (101 MHz, CDCl$_3$)** δ 165.8, 148.2, 138.9, 138.8, 136.8, 135.3, 134.9, 134.4, 134.2, 133.5, 132.7, 129.0, 128.7, 128.4, 128.3, 128.2, 128.1, 127.8, 126.6, 126.6, 126.3, 124.3, 121.7, 116.3, 21.6; **HRMS (ESI)** calcd for $C_{27}H_{20}N_2O$ $[M+H]^+$: 389.1648, Found: 389.1652.

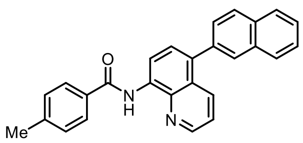

1g-4: A purification by flash chromatography in PE/DCM = 1/1 (v/v, R_f = 0.17) gave **1g-4** as white foam (0.75 g, 97%). **^{1}H NMR (400 MHz, CDCl$_3$)** δ 10.85 (s, 1H), 9.03 (d, *J* = 8.0 Hz, 1H), 8.89 (dd, *J* = 4.2, 1.6 Hz, 1H), 8.36 (dd, *J* = 8.6, 1.6 Hz, 1H), 8.03 (d, *J* = 8.2 Hz, 2H), 7.99 – 7.88 (m, 4H), 7.67 (d, *J* = 7.9 Hz, 1H), 7.61 (dd, *J* = 8.3, 1.7 Hz, 1H), 7.58 – 7.52 (m, 2H), 7.45 (dd, *J* = 8.5, 4.2 Hz, 1H), 7.37 (d, *J* = 7.9 Hz, 2H), 2.47 (s, 3H); **^{13}C NMR (101 MHz, CDCl$_3$)** δ 165.5, 148.1, 142.4, 138.9, 136.8, 134.9, 134.3, 134.3, 133.5, 132.7, 132.4, 129.5, 129.0, 128.4, 128.4, 128.1, 128.1, 127.8, 127.4, 126.6, 126.6, 126.3, 121.7, 116.2, 21.6; **HRMS (ESI)** calcd for $C_{27}H_{20}N_2O$ [M+H]$^+$: 389.1648, Found: 389.1651.

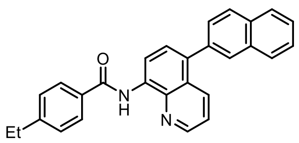

1g-5: A purification by flash chromatography in PE/DCM = 1/1 (v/v, R_f = 0.15) gave **1g-5** as light-yellow solid (0.24 g, 40%). **^{1}H NMR (400 MHz, CDCl$_3$)** δ 10.86 (s, 1H), 9.04 (d, *J* = 8.0 Hz, 1H), 8.88 (dd, *J* = 4.2, 1.6 Hz, 1H), 8.35 (dd, *J* = 8.6, 1.6 Hz, 1H), 8.06 (d, *J* = 8.0 Hz, 2H), 7.98 – 7.90 (m, 4H), 7.68 (d, *J* = 7.9 Hz, 1H), 7.61 (dd, *J* = 8.3, 1.7 Hz, 1H), 7.58 – 7.51 (m, 2H), 7.44 (dd, *J* = 8.6, 4.2 Hz, 1H), 7.40 (d, *J* = 7.9 Hz, 2H), 2.77 (q, *J* = 7.6 Hz, 2H), 1.32 (t, *J* = 7.6 Hz, 3H); **^{13}C NMR (101 MHz, CDCl$_3$)** δ 165.5, 148.6, 148.1, 138.9, 136.8, 134.9, 134.2, 134.2, 133.4, 132.6, 128.9, 128.4, 128.4, 128.3, 128.1, 128.1, 127.8, 127.5, 126.5, 126.3, 121.7, 116.1, 28.9, 15.4; **HRMS (ESI)** calcd for $C_{28}H_{22}N_2O$ [M+H]$^+$: 403.1805, Found: 403.1808.

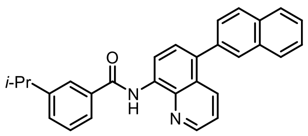

1g-6: A purification by flash chromatography in PE/DCM = 1/1 (v/v, R_f = 0.20) gave **1g-6** as light-yellow solid (0.29 g, 46%). **^{1}H NMR (400 MHz, CDCl$_3$)** δ 10.88 (s, 1H), 9.05 (d, *J* = 7.9 Hz, 1H), 8.89 (dd, *J* = 4.2, 1.7 Hz, 1H), 8.36 (dd, *J* = 8.5, 1.7 Hz, 1H), 8.02 (s, 1H), 8.00 – 7.90 (m, 5H), 7.68 (d, *J* = 8.0 Hz, 1H), 7.61 (dd, *J* = 8.4, 1.7 Hz, 1H), 7.59 – 7.53 (m, 2H), 7.53 – 7.47 (m, 2H), 7.44 (dd, *J* = 8.5, 4.2 Hz, 1H), 3.11 – 3.04 (m, 1H), 1.36 (d, *J* = 6.9 Hz, 6H); **^{13}C NMR (101 MHz, CDCl$_3$)** δ 165.9, 149.8, 148.2, 138.9, 136.7, 135.3, 134.9, 134.3, 134.2, 133.4, 132.6, 130.1, 129.0, 128.8, 128.4, 128.3, 128.1, 127.8, 126.6, 126.5, 126.3, 126.0, 124.5, 121.7, 116.2, 34.2, 24.0; **HRMS (ESI)** calcd for $C_{29}H_{24}N_2O$ [M+H]$^+$: 417.1961, Found: 417.1963.

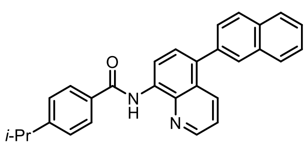

1g-7: A purification by flash chromatography in PE/DCM = 1/1 (v/v, R_f = 0.10) gave **1g-7** as light-yellow foam (0.27 g, 43%). **^{1}H NMR (400 MHz, CDCl$_3$)** δ 10.86 (s, 1H), 9.04 (d, *J* = 7.9 Hz, 1H), 8.88 (dd, *J* = 4.2, 1.7 Hz, 1H), 8.35 (dd, *J* = 8.5, 1.7 Hz, 1H), 8.07 (d, *J* = 7.9 Hz, 2H), 7.98 – 7.90 (m, 4H), 7.67 (d, *J* = 7.9 Hz, 1H), 7.61 (dd, *J* = 8.4, 1.7 Hz, 1H), 7.58 – 7.52 (m, 2H), 7.44 (dd, *J* = 8.4, 4.0 Hz, 3H), 3.07 – 3.00 (m, 1H), 1.33 (d, *J* = 6.9 Hz, 6H); **^{13}C NMR (101 MHz, CDCl$_3$)** δ 165.6, 153.2, 148.1, 138.9, 136.8, 134.9, 134.3, 134.2, 133.4, 132.8, 132.6, 128.9, 128.4, 128.3, 128.1, 128.1, 127.8, 127.5, 127.0, 126.5, 126.5, 126.3, 121.7, 116.1, 34.2, 23.9; **HRMS (ESI)** calcd for $C_{29}H_{24}N_2O$ [M+H]$^+$: 417.1961, Found: 417.1965.

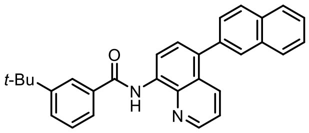

1g-8: A purification by flash chromatography in PE/DCM = 1/1 (v/v, R_f = 0.12) gave **1g-8** as white foam (0.40 g, 62%). **^{1}H NMR (400 MHz, $CDCl_3$)** δ 10.90 (s, 1H), 9.06 (d, *J* = 7.9 Hz, 1H), 8.88 (dd, *J* = 4.3, 1.6 Hz, 1H), 8.36 (dd, *J* = 8.5, 1.6 Hz, 1H), 8.21 (s, 1H), 8.00 – 7.89 (m, 5H), 7.72 – 7.59 (m, 3H), 7.58 – 7.48 (m, 3H), 7.44 (dd, *J* = 8.5, 4.1 Hz, 1H), 1.44 (s, 9H); **^{13}C NMR (101 MHz, $CDCl_3$)** δ 166.1, 152.1, 148.2, 138.9, 136.7, 135.0, 134.9, 134.3, 134.2, 133.5, 132.6, 129.1, 129.0, 128.5, 128.4, 128.3, 128.1, 127.8, 126.6, 126.5, 126.3, 124.9, 124.0, 121.7, 116.1, 35.0, 31.4; **HRMS (ESI)** calcd for $C_{30}H_{26}N_2O$ $[M+H]^+$: 431.2118, Found: 431.2121.

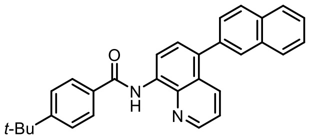

1g-9: A purification by flash chromatography in PE/DCM = 2/1 (v/v, R_f = 0.06) gave **1g-9** as white foam (0.85 g, 99%). **^{1}H NMR (400 MHz, $CDCl_3$)** δ 10.87 (s, 1H), 9.04 (d, *J* = 8.0 Hz, 1H), 8.88 (dd, *J* = 4.2, 1.6 Hz, 1H), 8.36 (dd, *J* = 8.5, 1.7 Hz, 1H), 8.08 (d, *J* = 8.5 Hz, 2H), 8.00 – 7.88 (m, 4H), 7.68 (d, *J* = 8.0 Hz, 1H), 7.64 – 7.52 (m, 5H), 7.44 (dd, *J* = 8.6, 4.1 Hz, 1H), 1.40 (s, 9H); **^{13}C NMR (101 MHz, $CDCl_3$)** δ 165.5, 155.5, 148.1, 138.9, 136.8, 134.9, 134.3, 134.3, 133.5, 132.7, 132.4, 129.0, 128.4, 128.4, 128.1, 128.1, 127.8, 127.3, 126.6, 126.6, 126.3, 125.8, 121.7, 116.2, 35.1, 31.3; **HRMS (ESI)** calcd for $C_{30}H_{26}N_2O$ $[M+H]^+$: 431.2118, Found: 431.2120.

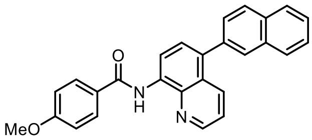

1g-10: A purification by recrystallization in DCM/EtOH gave **1g-10** as light-yellow powder (0.57 g, 71%). **^{1}H NMR (400 MHz, $CDCl_3$)** δ 10.82 (s, 1H), 9.02 (d, *J* = 7.9 Hz, 1H), 8.88 (d, *J* = 4.1 Hz, 1H), 8.36 (d, *J* = 8.5 Hz, 1H), 8.11 (d, *J* = 8.3 Hz, 2H), 7.99 – 7.90 (m, 4H), 7.67 (d, *J* = 7.9 Hz, 1H), 7.61 (d, *J* = 8.5 Hz, 1H), 7.58 – 7.54 (m, 2H), 7.45 (dd, *J* = 8.7, 4.1 Hz, 1H), 7.07 (d, *J* = 8.3 Hz, 2H), 3.92 (s, 3H); **^{13}C NMR (101 MHz, $CDCl_3$)** δ 165.1, 162.6, 148.1, 138.9, 136.8, 135.0, 134.3, 134.1, 133.5, 132.6, 129.3, 129.0, 128.5, 128.4, 128.1, 127.8, 127.5, 126.6, 126.3, 121.8, 116.1, 114.1, 55.6; **HRMS (ESI)** calcd for $C_{27}H_{20}N_2O_2$ $[M+H]^+$: 405.1598, Found: 405.1601.

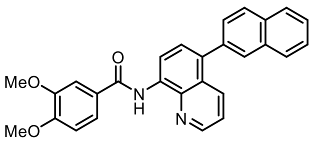

1g-11: A purification by flash chromatography in PE/DCM = 1/1 with 2% EtOAc (v/v, R_f = 0.13) gave **1g-11** as white solid (0.85 g, 98%). **^{1}H NMR (400 MHz, $CDCl_3$)** δ 10.84 (s, 1H), 9.01 (d, *J* = 8.0 Hz, 1H), 8.88 (dd, *J* = 4.1, 1.6 Hz, 1H), 8.36 (dd, *J* = 8.5, 1.6 Hz, 1H), 7.97 (d, *J* = 8.4 Hz, 1H), 7.96 – 7.89 (m, 3H), 7.74 – 7.70 (m, 2H), 7.68 (d, *J* = 8.0 Hz, 1H), 7.61 (dd, *J* = 8.3, 1.7 Hz, 1H), 7.58 – 7.52 (m, 2H), 7.45 (dd, *J* = 8.5, 4.2 Hz, 1H), 7.02 (d, *J* = 8.9 Hz, 1H), 4.03 (s, 3H), 3.99 (s, 3H); **^{13}C NMR (101 MHz, $CDCl_3$)** δ 165.1, 152.2, 149.3, 148.1, 138.9, 136.8, 135.0, 134.3, 134.2, 133.5, 132.7, 129.0, 128.5, 128.4, 128.1, 127.9, 127.8, 126.6, 126.6,

126.3, 121.7, 119.9, 116.0, 111.0, 110.5, 56.2, 56.2; **HRMS (ESI)** calcd for $C_{28}H_{22}N_2O_3$ $[M+H]^+$: 435.1703, Found: 435.1706.

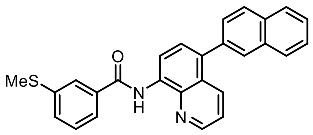

1g-12: A purification by flash chromatography in PE/DCM = 1/1 (v/v, R_f = 0.05) gave **1g-12** as light-yellow foam (0.41 g, 65%). **^{1}H NMR (400 MHz, CDCl$_3$)** δ 10.85 (s, 1H), 9.02 (d, *J* = 7.9 Hz, 1H), 8.89 (dd, *J* = 4.2, 1.7 Hz, 1H), 8.36 (dd, *J* = 8.5, 1.6 Hz, 1H), 8.00 (s, 1H), 7.99 – 7.89 (m, 4H), 7.86 – 7.83 (m, 1H), 7.68 (d, *J* = 8.0 Hz, 1H), 7.61 (dd, *J* = 8.4, 1.7 Hz, 1H), 7.58 – 7.53 (m, 2H), 7.50 – 7.41 (m, 3H), 2.60 (s, 3H); **^{13}C NMR (101 MHz, CDCl$_3$)** δ 165.1, 148.2, 140.0, 138.8, 136.7, 135.9, 134.9, 134.5, 134.0, 133.4, 132.6, 129.7, 129.1, 129.0, 128.3, 128.3, 128.1, 127.8, 126.6, 126.5, 126.3, 125.1, 123.4, 116.3, 15.7; **HRMS (ESI)** calcd for $C_{27}H_{20}N_2OS$ $[M+H]^+$: 421.1369, Found: 421.1372.

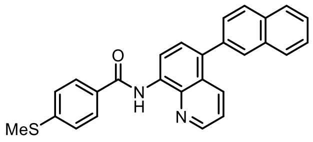

1g-13: A purification by flash chromatography in PE/DCM = 1/1 with 1% EtOAc (v/v, R_f = 0.39) gave **1g-13** as light-yellow solid (0.18 g, 29%). **^{1}H NMR (400 MHz, CDCl$_3$)** δ 10.84 (s, 1H), 9.02 (d, *J* = 8.0 Hz, 1H), 8.88 (dd, *J* = 4.2, 1.6 Hz, 1H), 8.35 (dd, *J* = 8.6, 1.7 Hz, 1H), 8.05 (d, *J* = 8.5 Hz, 2H), 7.99 – 7.88 (m, 4H), 7.67 (d, *J* = 8.0 Hz, 1H), 7.60 (dd, *J* = 8.4, 1.7 Hz, 1H), 7.58 – 7.52 (m, 2H), 7.44 (dd, *J* = 8.5, 4.1 Hz, 1H), 7.39 (d, *J* = 8.5 Hz, 2H), 2.56 (s, 3H); **^{13}C NMR (101 MHz, CDCl$_3$)** δ 164.9, 148.1, 144.1, 138.8, 136.7, 134.9, 134.3, 134.1, 133.4, 132.6, 131.2, 128.9, 128.4, 128.3, 128.1, 127.8, 127.8, 126.5, 126.5, 126.3, 125.5, 121.7, 116.1, 15.1; **HRMS (ESI)** calcd for $C_{27}H_{20}N_2OS$ $[M+H]^+$: 421.1369, Found: 421.1371.

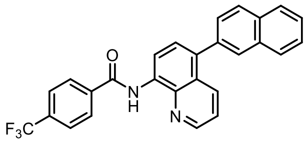

1g-14: A purification by recrystallization in DCM/EtOH gave **1g-14** as light-yellow powder (0.70 g, 79%). **^{1}H NMR (400 MHz, CDCl$_3$)** δ 10.92 (s, 1H), 9.01 (d, *J* = 8.0 Hz, 1H), 8.89 (dd, *J* = 4.2, 1.6 Hz, 1H), 8.38 (dd, *J* = 8.5, 1.6 Hz, 1H), 8.23 (d, *J* = 8.1 Hz, 2H), 7.98 (d, *J* = 8.5 Hz, 1H), 7.96 – 7.89 (m, 3H), 7.85 (d, *J* = 8.1 Hz, 2H), 7.69 (d, *J* = 8.0 Hz, 1H), 7.61 (dd, *J* = 8.4, 1.7 Hz, 1H), 7.59 – 7.53 (m, 2H), 7.47 (dd, *J* = 8.6, 4.2 Hz, 1H); **^{19}F NMR (376 MHz, CDCl$_3$)** δ -62.84; **^{13}C NMR (101 MHz, CDCl$_3$)** δ 164.1, 148.3, 138.8, 138.4, 136.5, 135.0, 134.9, 133.6, 133.5 (q, *J* = 32.8 Hz), 133.4, 132.7, 129.0, 128.3, 128.2, 128.1, 128.1, 127.8, 126.6, 126.6, 126.4, 125.9 (q, *J* = 3.7 Hz), 123.8 (q, *J* = 273.5 Hz), 121.9, 116.4; **HRMS (ESI)** calcd for $C_{27}H_{17}F_3N_2O$ $[M+H]^+$: 443.1366, Found: 443.1366.

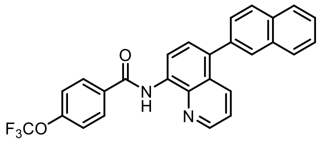

1g-15: A purification by flash chromatography in PE/DCM = 1/1 (v/v, R_f = 0.15) gave **1g-15** as light-yellow solid (0.49 g, 71%). **^{1}H NMR (400 MHz, CDCl$_3$)** δ 10.86 (s, 1H), 9.00 (d, *J* = 7.9 Hz, 1H), 8.88 (dd, *J* = 4.2, 1.6 Hz, 1H), 8.37 (dd, *J* = 8.6, 1.6 Hz, 1H), 8.17 (d, *J* = 8.8 Hz, 2H), 8.02 – 7.85 (m, 4H), 7.68 (d, *J* = 7.9 Hz, 1H), 7.60 (dd, *J* = 8.4, 1.7 Hz, 1H), 7.59

– 7.53 (m, 2H), 7.46 (dd, J = 8.6, 4.2 Hz, 1H), 7.41 (d, J = 8.3 Hz, 2H); **^{19}F NMR (376 MHz, CDCl$_3$)** δ -57.58; **^{13}C NMR (101 MHz, CDCl$_3$)** δ 164.1, 151.8 (m), 148.2, 138.8, 136.6, 135.0, 134.7, 133.8, 133.6, 133.4, 132.6, 129.3, 129.0, 128.3, 128.2, 128.1, 128.1, 127.8, 126.6, 126.5, 126.3, 121.8, 120.9, 120.4 (q, J = 258.4 Hz), 116.3; **HRMS (ESI)** calcd for $C_{27}H_{17}F_3N_2O_2$ $[M+H]^+$: 459.1315, Found: 459.1318.

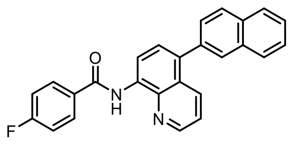

1g-16: A purification by recrystallization in DCM/EtOH gave **1g-16** as white powder (0.55 g, 70%). **^{1}H NMR (400 MHz, CDCl$_3$)** δ 10.88 (s, 1H), 9.04 (d, J = 7.9 Hz, 1H), 8.93 (d, J = 4.4 Hz, 1H), 8.41 (d, J = 8.6 Hz, 1H), 8.23 – 8.13 (m, 2H), 8.07 – 7.89 (m, 4H), 7.72 (d, J = 7.9 Hz, 1H), 7.65 (d, J = 8.3 Hz, 1H), 7.61 – 7.59 (m, 2H), 7.50 (dd, J = 8.6, 4.1 Hz, 1H), 7.35 – 7.18 (m, 2H); **^{19}F NMR (376 MHz, CDCl$_3$)** δ -107.57; **^{13}C NMR (101 MHz, CDCl$_3$)** δ 165.0 (d, J = 252.5 Hz), 164.4, 148.2, 138.8, 136.6, 135.0, 134.5, 134.0, 133.4, 132.6, 131.4 (d, J = 3.3 Hz), 129.7 (d, J = 9.0 Hz), 129.0, 128.4, 128.3, 128.1, 128.1, 127.8, 126.6, 126.6, 126.3, 121.8, 116.2, 115.9 (d, J = 21.9 Hz); **HRMS (ESI)** calcd for $C_{26}H_{17}FN_2O$ $[M+H]^+$: 393.1398, Found: 393.1400.

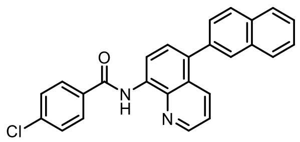

1g-17: A purification by recrystallization in DCM/EtOH gave **1g-17** as white foam (0.56 g, 71%). **^{1}H NMR (400 MHz, CDCl$_3$)** δ 10.85 (s, 1H), 9.00 (d, J = 7.9 Hz, 1H), 8.89 (d, J = 4.1 Hz, 1H), 8.36 (d, J = 9.1 Hz, 1H), 8.07 (d, J = 8.3 Hz, 2H), 7.98 (d, J = 8.4 Hz, 1H), 7.96 – 7.88 (m, 3H), 7.68 (d, J = 7.9 Hz, 1H), 7.61 (dd, J = 8.3, 1.7 Hz, 1H), 7.58 – 7.54 (m, 4H), 7.46 (dd, J = 8.6, 4.1 Hz, 1H); **^{13}C NMR (101 MHz, CDCl$_3$)** δ 164.4, 148.3, 138.9, 138.2, 136.7, 135.0, 134.7, 133.9, 133.6, 133.5, 132.7, 129.2, 129.0, 128.8, 128.4, 128.3, 128.1, 128.1, 127.8, 126.6, 126.4, 121.8, 116.3; **HRMS (ESI)** calcd for $C_{26}H_{17}ClN_2O$ $[M+H]^+$: 409.1102, Found: 409.1105.

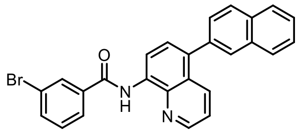

1g-18: A purification by flash chromatography in PE/DCM = 1/1 with 1% EtOAc (v/v, R_f = 0.36) gave **1g-18** as white foam (0.63 g, 93%). **^{1}H NMR (400 MHz, CDCl$_3$)** δ 10.82 (s, 1H), 8.99 (d, J = 7.9 Hz, 1H), 8.92 – 8.87 (m, 1H), 8.36 (dd, J = 8.5, 1.7 Hz, 1H), 8.26 (s, 1H), 8.03 (d, J = 7.8 Hz, 1H), 8.00 – 7.88 (m, 4H), 7.73 (dd, J = 8.0, 2.0 Hz, 1H), 7.67 (d, J = 8.0 Hz, 1H), 7.62 – 7.54 (m, 3H), 7.49 – 7.40 (m, 2H); **^{13}C NMR (101 MHz, CDCl$_3$)** δ 163.9, 148.3, 138.8, 137.1, 136.6, 135.0, 134.9, 134.8, 133.7, 133.4, 132.6, 130.7, 130.4, 129.0, 128.3, 128.2, 128.1, 128.1, 127.8, 126.6, 126.5, 126.3, 125.8, 123.1, 121.8, 116.4; **HRMS (ESI)** calcd for $C_{26}H_{17}BrN_2O$ $[M+H]^+$: 453.0597, Found: 453.0599.

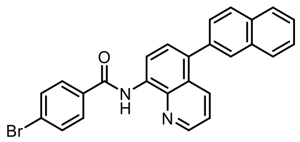

1g-19: A purification by recrystallization in DCM/EtOH gave **1g-19** as pink powder (0.54 g, 60%). **^{1}H NMR (400 MHz, CDCl$_3$)** δ 10.84 (s, 1H), 8.99 (d, J = 8.0 Hz, 1H), 8.88 (dd, J = 4.1, 1.7 Hz, 1H), 8.36 (dd, J = 8.5, 1.7 Hz, 1H), 7.97 (d, J = 8.4 Hz, 1H), 7.96 – 7.89 (m, 5H), 7.84

(d, J = 8.5 Hz, 2H), 7.67 (d, J = 7.9 Hz, 1H), 7.60 (dd, J = 8.4, 1.7 Hz, 1H), 7.58 – 7.53 (m, 2H), 7.46 (dd, J = 8.6, 4.2 Hz, 1H); **^{13}C NMR (101 MHz, CDCl$_3$)** δ 164.7, 148.2, 138.9, 138.1, 136.7, 135.0, 134.7, 134.7, 133.9, 133.5, 132.7, 129.0, 128.4, 128.3, 128.1, 128.1, 127.8, 126.6, 126.4, 121.8, 116.3, 99.0; **HRMS (ESI)** calcd for $C_{26}H_{17}BrN_2O$ [M+H]$^+$: 453.0597, Found: 453.0601.

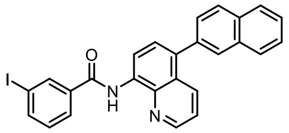

1g-20: A purification by flash chromatography in PE/DCM = 1/1 with 1% EtOAc (v/v, R_f = 0.48) gave **1g-20** as light-yellow foam (0.61 g, 81%). **^{1}H NMR (400 MHz, CDCl$_3$)** δ 10.79 (s, 1H), 8.99 (d, J = 7.9 Hz, 1H), 8.89 (dd, J = 4.2, 1.6 Hz, 1H), 8.45 (s, 1H), 8.36 (dd, J = 8.6, 1.6 Hz, 1H), 8.06 (d, J = 7.7 Hz, 1H), 7.99 – 7.87 (m, 5H), 7.66 (d, J = 7.9 Hz, 1H), 7.61 – 7.55 (m, 3H), 7.45 (dd, J = 8.5, 4.1 Hz, 1H), 7.30 (t, J = 7.8 Hz, 1H); **^{13}C NMR (101 MHz, CDCl$_3$)** δ 163.8, 148.3, 140.8, 138.8, 137.1, 136.6, 136.6, 135.0, 134.7, 133.7, 133.4, 132.6, 130.4, 129.0, 128.3, 128.3, 128.1, 128.1, 127.8, 126.6, 126.5, 126.3, 121.8, 116.4, 94.7; **HRMS (ESI)** calcd for $C_{26}H_{17}IN_2O$ [M+H]$^+$: 501.0458, Found: 501.0460.

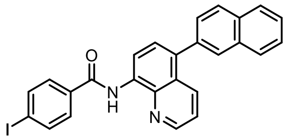

1g-21: A purification by recrystallization in DCM/EtOH gave **1g-21** as white powder (0.76 g, 76%). **^{1}H NMR (400 MHz, CDCl$_3$)** δ 10.85 (s, 1H), 9.00 (d, J = 7.9 Hz, 1H), 8.89 (dd, J = 4.1, 1.7 Hz, 1H), 8.37 (dd, J = 8.5, 1.6 Hz, 1H), 8.03 – 7.87 (m, 6H), 7.73 – 7.69 (m, 2H), 7.68 (d, J = 8.0 Hz, 1H), 7.61 (dd, J = 8.4, 1.7 Hz, 1H), 7.58 – 7.52 (m, 2H), 7.46 (dd, J = 8.5, 4.2 Hz, 1H); **^{13}C NMR (101 MHz, CDCl$_3$)** δ 164.5, 148.3, 138.9, 136.7, 135.0, 134.7, 134.1, 133.9, 133.5, 132.7, 132.1, 129.0, 128.4, 128.3, 128.1, 128.1, 127.8, 126.7, 126.6, 126.4, 121.8, 116.3; **HRMS (ESI)** calcd for $C_{26}H_{17}IN_2O$ [M+H]$^+$: 501.0458, Found: 501.0461.

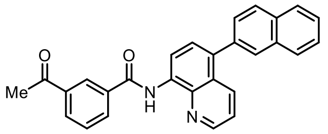

1g-22: A purification by flash chromatography in PE/DCM = 1/1 with 5% EtOAc (v/v, R_f = 0.31) gave **1g-22** as yellow powder (0.38 g, 61%). **^{1}H NMR (400 MHz, CDCl$_3$)** δ 10.93 (s, 1H), 9.02 (d, J = 7.9 Hz, 1H), 8.90 (dd, J = 4.2, 1.6 Hz, 1H), 8.70 (s, 1H), 8.36 (dd, J = 8.5, 1.6 Hz, 1H), 8.31 (d, J = 7.8 Hz, 1H), 8.19 (d, J = 7.8 Hz, 1H), 8.00 – 7.87 (m, 4H), 7.70 – 7.67 (m, 2H), 7.61 (dd, J = 8.4, 1.7 Hz, 1H), 7.58 – 7.53 (m, 2H), 7.46 (dd, J = 8.5, 4.1 Hz, 1H), 2.73 (s, 3H); **^{13}C NMR (101 MHz, CDCl$_3$)** δ 197.5, 164.5, 148.3, 138.8, 137.6, 136.6, 135.7, 135.0, 134.8, 133.8, 133.4, 132.6, 131.5, 131.4, 129.3, 129.0, 128.3, 128.2, 128.1, 128.1, 127.8, 127.4, 126.6, 126.5, 126.3, 121.9, 116.4, 26.9; **HRMS (ESI)** calcd for $C_{28}H_{20}N_2O_2$ [M+H]$^+$: 417.1598, Found: 417.1599.

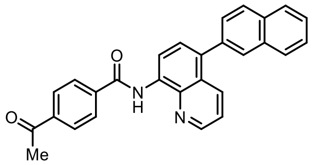

1g-23: A purification by flash chromatography in PE/DCM = 1/1 with 1% EtOAc (v/v, R_f = 0.08) gave **1g-23** as light-yellow solid (0.75 g, 90%). **^{1}H NMR (400 MHz, CDCl$_3$)** δ 10.94 (s, 1H), 9.02 (d, J = 8.0 Hz, 1H), 8.90 (dd, J = 4.1, 1.6 Hz, 1H), 8.37 (dd, J = 8.5, 1.6 Hz, 1H), 8.21 (d, J = 8.5 Hz, 2H), 8.15 (d, J = 8.5 Hz, 2H), 8.00 – 7.88 (m, 4H), 7.69 (d, J = 7.9 Hz, 1H),

7.61 (dd, *J* = 8.4, 1.7 Hz, 1H), 7.58 – 7.52 (m, 2H), 7.47 (dd, *J* = 8.6, 4.2 Hz, 1H), 2.70 (s, 3H); **^{13}C NMR (101 MHz, CDCl$_3$)** δ 197.5, 164.4, 148.3, 139.5, 139.0, 138.9, 136.6, 135.1, 134.9, 133.8, 133.5, 132.7, 129.0, 128.8, 128.3, 128.3, 128.2, 128.1, 127.8, 127.7, 126.6, 126.4, 121.9, 116.4, 27.0; **HRMS (ESI)** calcd for $C_{28}H_{20}N_2O_2$ $[M+H]^+$: 417.1598, Found: 417.1599.

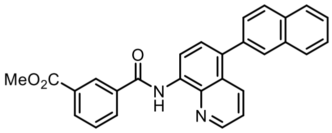

1g-24: A purification by flash chromatography in PE/DCM = 1/1 with 5% EtOAc (v/v, R_f = 0.67) gave **1g-24** as light-yellow foam (0.37 g, 57%). **^{1}H NMR (400 MHz, CDCl$_3$)** δ 10.91 (s, 1H), 9.02 (d, *J* = 8.0 Hz, 1H), 8.90 (dd, *J* = 4.2, 1.6 Hz, 1H), 8.78 (s, 1H), 8.36 (dd, *J* = 8.6, 1.6 Hz, 1H), 8.31 (d, *J* = 7.9 Hz, 1H), 8.27 (d, *J* = 7.9 Hz, 1H), 8.00 – 7.87 (m, 4H), 7.71 – 7.63 (m, 2H), 7.61 (dd, *J* = 8.3, 1.7 Hz, 1H), 7.58 – 7.52 (m, 2H), 7.45 (dd, *J* = 8.5, 4.2 Hz, 1H), 4.01 (s, 3H); **^{13}C NMR (101 MHz, CDCl$_3$)** δ 166.4, 164.5, 148.3, 138.8, 136.6, 135.6, 134.9, 134.7, 133.8, 133.4, 132.8, 132.6, 131.8, 130.9, 129.1, 129.0, 128.4, 128.3, 128.3, 128.1, 128.1, 127.8, 126.6, 126.5, 126.3, 121.8, 116.4, 52.5; **HRMS (ESI)** calcd for $C_{28}H_{20}N_2O_3$ $[M+H]^+$: 433.1547, Found: 433.1549.

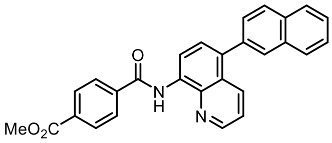

1g-25: A purification by flash chromatography in PE/DCM = 1/1 with 1% EtOAc (v/v, R_f = 0.13) gave **1g-25** as light-yellow powder (0.63 g, 73%). **^{1}H NMR (400 MHz, CDCl$_3$)** δ 10.93 (s, 1H), 9.02 (d, *J* = 7.9 Hz, 1H), 8.90 (dd, *J* = 4.2, 1.6 Hz, 1H), 8.37 (dd, *J* = 8.5, 1.6 Hz, 1H), 8.24 (d, *J* = 8.6 Hz, 2H), 8.18 (d, *J* = 8.5 Hz, 2H), 7.98 (d, *J* = 8.4 Hz, 1H), 7.96 – 7.88 (m, 3H), 7.69 (d, *J* = 7.9 Hz, 1H), 7.61 (dd, *J* = 8.4, 1.7 Hz, 1H), 7.59 – 7.52 (m, 2H), 7.47 (dd, *J* = 8.6, 4.1 Hz, 1H), 3.99 (s, 3H); **^{13}C NMR (101 MHz, CDCl$_3$)** δ 166.4, 164.5, 148.3, 139.1, 138.9, 136.6, 135.0, 134.9, 133.8, 133.5, 133.1, 132.7, 130.1, 129.0, 128.3, 128.3, 128.1, 128.1, 127.8, 127.4, 126.6, 126.4, 121.9, 116.4, 52.5; **HRMS (ESI)** calcd for $C_{28}H_{20}N_2O_3$ $[M+H]^+$: 433.1547, Found: 433.1550.

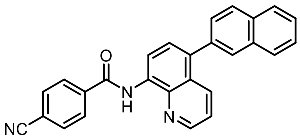

1g-26: A purification by flash chromatography in PE/DCM = 1/1 (v/v, R_f = 0.42) gave **1g-26** as light-yellow foam (0.53 g, 66%). **^{1}H NMR (400 MHz, CDCl$_3$)** δ 10.93 (s, 1H), 8.99 (d, *J* = 7.9 Hz, 1H), 8.89 (dd, *J* = 4.2, 1.6 Hz, 1H), 8.38 (dd, *J* = 8.6, 1.6 Hz, 1H), 8.22 (d, *J* = 8.4 Hz, 2H), 7.98 (d, *J* = 8.4 Hz, 1H), 7.96 – 7.90 (m, 3H), 7.88 (d, *J* = 8.4 Hz, 2H), 7.69 (d, *J* = 7.9 Hz, 1H), 7.60 (dd, *J* = 8.4, 1.7 Hz, 1H), 7.59 – 7.54 (m, 2H), 7.48 (dd, *J* = 8.6, 4.2 Hz, 1H); **^{13}C NMR (101 MHz, CDCl$_3$)** δ 163.5, 148.4, 139.1, 138.8, 136.5, 135.2, 135.1, 133.5, 133.5, 132.8, 132.7, 129.0, 128.3, 128.2, 128.1, 128.1, 127.8, 126.6, 126.4, 122.0, 118.1, 116.5, 115.4; **HRMS (ESI)** calcd for $C_{27}H_{17}N_3O$ $[M+H]^+$: 400.1444, Found: 400.1447.

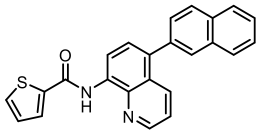

1g-27: A purification by flash chromatography in PE/DCM = 2/1 (v/v, R_f = 0.10) gave **1g-27** as light-yellow solid (0.71 g, 93%). **^{1}H NMR (400 MHz, CDCl$_3$)** δ 10.73 (s, 1H), 8.94 (d, *J* = 8.0 Hz, 1H), 8.89 (dd, *J* = 4.1, 1.6 Hz, 1H), 8.35 (dd, *J* = 8.5, 1.6 Hz, 1H), 7.97 (d, *J* = 8.4 Hz, 1H), 7.95 – 7.86 (m, 4H), 7.66 (d, *J* = 8.0 Hz, 1H), 7.62 – 7.59 (m, 2H), 7.58 – 7.53 (m, 2H), 7.45 (dd, *J* = 8.6, 4.1 Hz, 1H), 7.21 (dd, *J* = 5.0, 3.7 Hz, 1H); **^{13}C NMR (101 MHz, CDCl$_3$)** δ 160.1, 148.2, 140.2, 138.7, 136.7, 135.0, 134.4, 133.9, 133.5, 132.7, 131.0, 129.0, 128.5, 128.4, 128.3, 128.1, 128.0, 127.8, 126.6, 126.3, 121.8, 116.2; **HRMS (ESI)** calcd for $C_{24}H_{16}N_2OS$ $[M+H]^+$: 381.1056, Found: 381.1059.

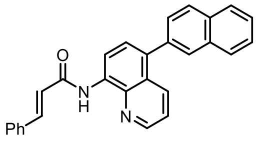

1g-28: A purification by flash chromatography in PE/DCM = 1/1 (v/v, R_f = 0.37) gave **1g-28** as light-yellow solid (0.34 g, 43%). **^{1}H NMR (400 MHz, CDCl$_3$)** δ 10.15 (s, 1H), 9.01 (d, *J* = 8.0 Hz, 1H), 8.88 (dd, *J* = 4.1, 1.6 Hz, 1H), 8.36 (dd, *J* = 8.5, 1.6 Hz, 1H), 8.00 – 7.89 (m, 4H), 7.86 (d, *J* = 15.6 Hz, 1H), 7.69 – 7.62 (m, 3H), 7.60 (dd, *J* = 8.4, 1.7 Hz, 1H), 7.58 – 7.53 (m, 2H), 7.48 – 7.36 (m, 4H), 6.86 (d, *J* = 15.5 Hz, 1H); **^{13}C NMR (101 MHz, CDCl$_3$)** δ 164.2, 148.1, 142.2, 138.6, 136.8, 135.0, 134.9, 134.4, 134.2, 133.5, 132.7, 130.0, 129.0, 129.0, 128.5, 128.3, 128.1, 128.1, 127.8, 126.6, 126.3, 121.7, 121.7, 116.6; **HRMS (ESI)** calcd for $C_{28}H_{20}N_2O$ $[M+H]^+$: 401.1648, Found: 401.1650.

2.6 Synthesis and characterization of ligands (*S*)-L6 – (*S*)-L8

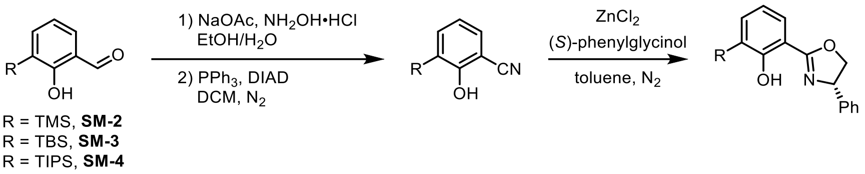

3-Silyl-2-hydroxybenzaldehyde **SM-2**, **SM-3** and **SM-4** were known compounds that were prepared following reported literature.[6]

Step 1: To a mixture of salicylaldehyde **SM-2** (10 mmol), $NH_2OH\cdot HCl$ (25 mmol, 2.5 eq) and NaOAc (40 mmol, 4.0 eq) in a mixed solvents of EtOH and H_2O (30 mL: 10 mL) was heated to reflux for overnight. After the reaction was completed, the reaction mixture was extracted with DCM (3×20 mL). Then, the DCM layer was dried over anhydrous Na_2SO_4 and concentrated in vacuo to afford the crude salicylaldoxime without further purification.

Step 2: To a solution of above crude salicylaldoxime and PPh_3 (25 mmol, 2.5 eq) in anhydrous DCM (50 mL) was slowly added DIAD (25 mmol, 2.5 eq) at room temperature under N_2 atmosphere and stirred for 2 hours. After the reaction was completed, the reaction mixture was concentrated in vacuo to remove DCM. Then, the residue was dissolved in Et_2O and filtered to separate the insoluble triphenylphosphine oxide. After concentration, the crude product was purified by flash chromatography on silica gel in PE/EtOAc to afford the salicylonitrile.

Step 3: A suspension of salicylonitrile, (*S*)-phenylglycinol (1.1 equiv) and $ZnCl_2$ (10 mol%) in toluene was heated to reflux under N_2 atmosphere for overnight. After the toluene being evaporated, the residue was purified by flash chromatography on silica gel in PE/EtOAc to give the desired Salox ligand.

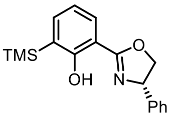

(*S*)-**L6:** A purification by flash chromatography in PE (R_f = 0.17) gave (*S*)-**L6** as pink solid (0.75 g, 19% yield starting from **Step 3**). **^{1}H NMR (400 MHz, CDCl$_3$)** δ 12.29 (s, 1H), 7.74 (dd, *J* = 7.8, 1.8 Hz, 1H), 7.51 (dd, *J* = 7.2, 1.8 Hz, 1H), 7.42 – 7.35 (m, 2H), 7.34 – 7.30 (m, 3H), 6.90 (t, *J* = 7.5 Hz, 1H), 5.46 (dd, *J* = 10.1, 8.3 Hz, 1H), 4.79 (dd, *J* = 10.1, 8.4 Hz, 1H), 4.25 (t, *J* = 8.4 Hz, 1H), 0.32 (s, 9H); **^{13}C NMR (101 MHz, CDCl$_3$)** δ 166.6, 164.8, 141.7, 139.2, 129.5, 128.9, 127.9, 127.1, 126.7, 118.4, 109.3, 74.0, 69.0, -1.1; **HRMS (ESI)** calcd for $C_{18}H_{21}NO_2Si$ $[M+H]^+$: 312.1414, Found: 312.1413.

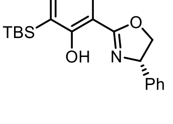

(*S*)-**L7:** A purification by flash chromatography in PE (R_f = 0.25) gave (*S*)-**L7** as colorless oil (1.96 g, 50% yield starting from **Step 3**). **^{1}H NMR (400 MHz, CDCl$_3$)** δ 12.30 (s, 1H), 7.76 (dd, *J* = 7.8, 1.8 Hz, 1H), 7.52 (dd, *J* = 7.2, 1.8 Hz, 1H), 7.42 – 7.36 (m, 2H), 7.35 – 7.28 (m, 3H), 6.90 (t, *J* = 7.5 Hz, 1H), 5.45 (dd, *J* = 10.1, 8.3 Hz, 1H), 4.79 (dd, *J* = 10.1, 8.4 Hz, 1H), 4.24 (t, *J* = 8.4 Hz, 1H), 0.93 (s, 9H), 0.34 (s, 3H), 0.33 (s, 3H); **^{13}C NMR (101 MHz, CDCl$_3$)** δ 166.7, 165.0, 141.7, 140.9, 129.6, 128.9, 127.9, 126.7, 124.9, 118.2,

109.5, 74.0, 69.0, 27.2, 17.7, -4.6, -4.7; **HRMS (ESI)** calcd for $C_{21}H_{27}NO_2Si$ [M+H]$^+$: 354.1884, Found: 354.1885.

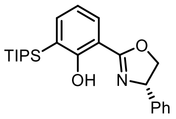

(*S*)-**L8:** A purification by flash chromatography in PE (R_f = 0.31) gave (*S*)-**L8** as colorless oil (1.45 g, 28% yield starting from **Step 3**). **^{1}H NMR (400 MHz, $CDCl_3$)** δ 12.33 (s, 1H), 7.76 (dd, *J* = 7.7, 1.8 Hz, 1H), 7.55 (dd, *J* = 7.3, 1.8 Hz, 1H), 7.41 – 7.37 (m, 2H), 7.35 – 7.30 (m, 3H), 6.91 (t, *J* = 7.5 Hz, 1H), 5.46 (dd, *J* = 10.1, 8.3 Hz, 1H), 4.79 (dd, *J* = 10.1, 8.4 Hz, 1H), 4.24 (t, *J* = 8.4 Hz, 1H), 1.60 – 1.49 (m, 3H), 1.17 – 1.04 (m, 18H); **^{13}C NMR (101 MHz, $CDCl_3$)** δ 166.8, 165.3, 141.7, 141.3, 129.3, 128.9, 127.9, 126.7, 122.8, 118.2, 109.5, 74.0, 69.0, 19.0, 11.7; **HRMS (ESI)** calcd for $C_{24}H_{33}NO_2Si$ [M+H]$^+$: 396.2353, Found: 396.2354.

3. General Procedure for Nickel(II)/Salox-Catalyzed Enantioselective C−H Functionalization

3.1 Optimization of reaction conditions

<u>**Table S1.** Initial screening of DG*ab*</u>

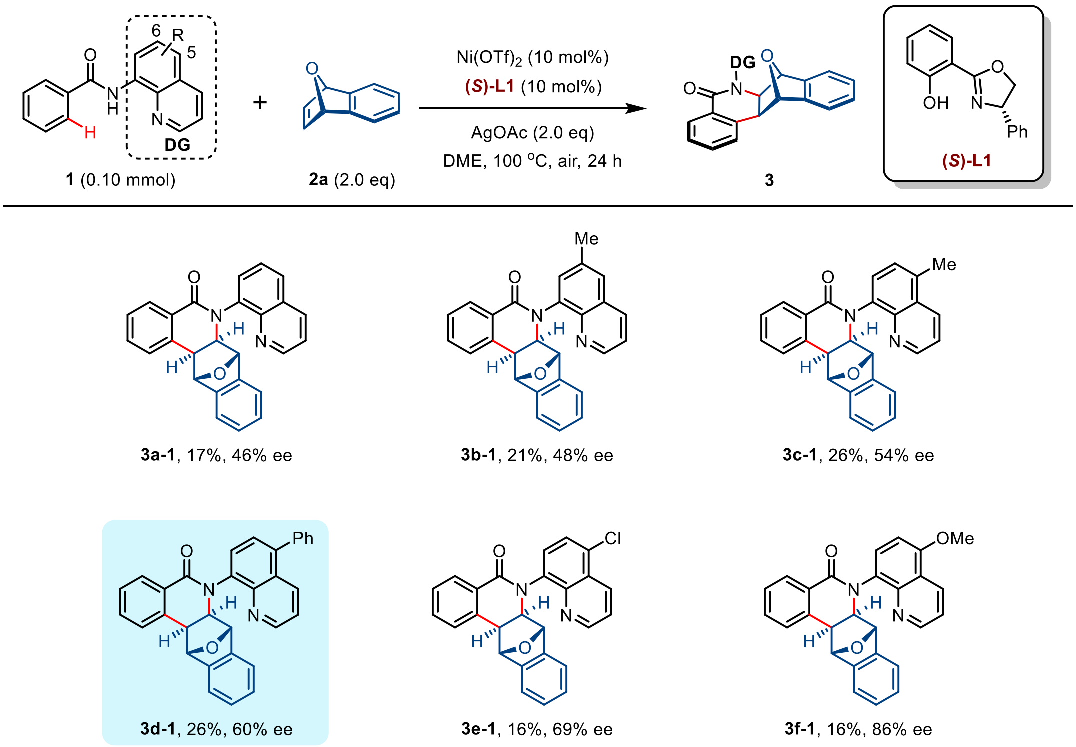

Reaction conditions: **1** (0.10 mmol), **2a** (0.20 mmol, 2.0 eq), Ni(OTf)$_2$ (10 mol%), (*S*)-**L1** (10 mol%), AgOAc (0.20 mmol, 2.0 eq), dry DME (0.5 mL), 100 °C, 24 h. [a]^{1}H NMR yield using 1,3,5-trimethoxybenzene as internal standard. [b]The value of ee was determined by HPLC.

Table S2. Screening of silver salts[a,b]

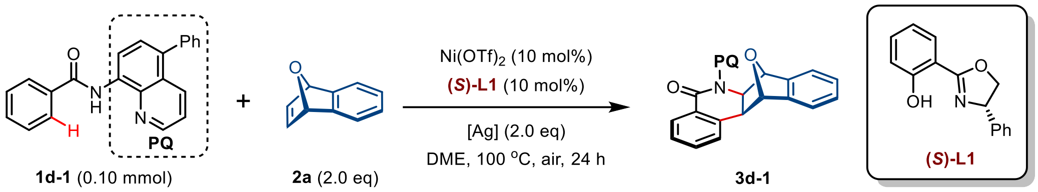

entry	[Ag]	yield of **3d-1**	ee of **3d-1**
1	Ag_2CO_3	trace	--
2	$AgNO_3$	n.d.	--
3	$PhCO_2Ag$	28	55
4	PivOAg	44	48
5	AgOAc	26	60
6	$EtCO_2Ag$	48	67

Reaction conditions: **1d-1** (0.10 mmol), **2a** (0.20 mmol, 2.0 eq), $Ni(OTf)_2$ (10 mol%), (*S*)-**L1** (10 mol%), [Ag] (0.20 mmol, 2.0 eq), dry DME (0.5 mL), 100 °C, 24 h. [a]^{1}H NMR yield using 1,3,5-trimethoxybenzene as internal standard. [b]The value of ee was determined by HPLC.

Table S3. Investigation of phosphine ligands as additives[ab]

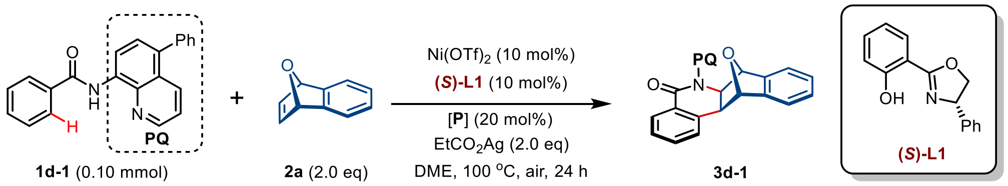

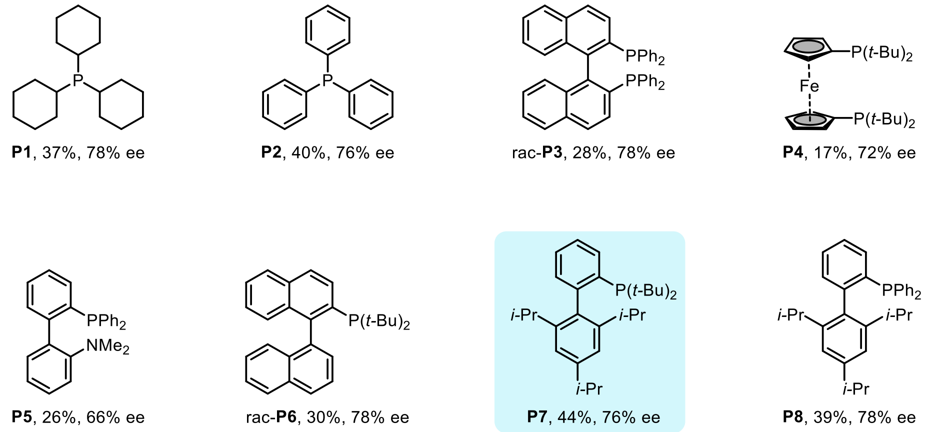

Reaction conditions: **1d-1** (0.10 mmol), **2a** (0.20 mmol, 2.0 eq), Ni(OTf)$_2$ (10 mol%), (*S*)-**L1** (10 mol%), [**P**] (20 mol%), EtCO$_2$Ag (0.20 mmol, 2.0 eq), dry DME (0.5 mL), 100 °C, 24 h. [a]^{1}H NMR yield using 1,3,5-trimethoxybenzene as internal standard. [b]The value of ee was determined by HPLC.

Table S4. Screening of chiral Salox ligands[ab]

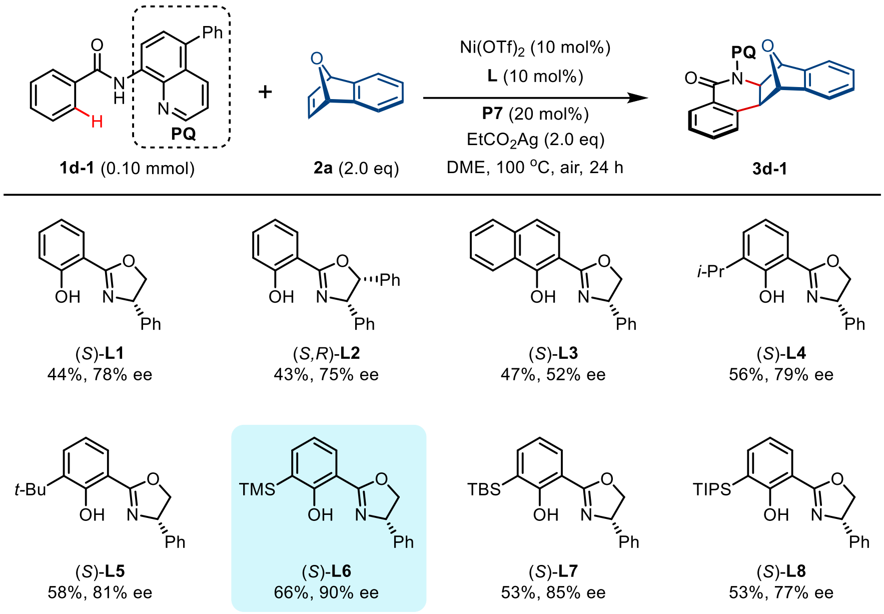

Reaction conditions: **1d-1** (0.10 mmol), **2a** (0.20 mmol, 2.0 eq), $Ni(OTf)_2$ (10 mol%), **L** (10 mol%), **P7** (20 mol%), $EtCO_2Ag$ (0.20 mmol, 2.0 eq), dry DME (0.5 mL), 100 ºC, 24 h. [a]^{1}H NMR yield using 1,3,5-trimethoxybenzene as internal standard. [b]The value of ee was determined by HPLC.

Table S5. Screening of Nickel salts[ab]

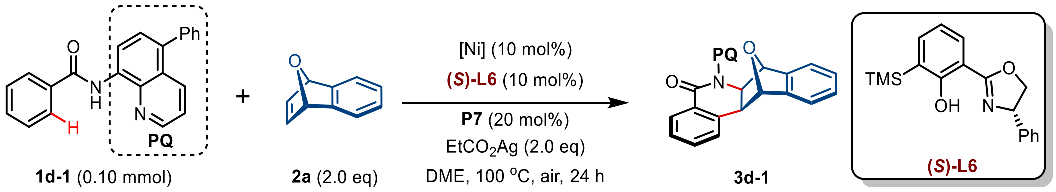

entry	[Ni]	yield of **3d-1**[a]	ee of **3d-1**[b]
1	$Ni(acac)_2$	43	85
2	$Ni(OAc)_2$	47	89
3	$Ni(OTf)_2$	66	90
4[c]	$Ni(OTf)_2$	31	--
5	w/o Ni	n.d.	--

Reaction conditions: **1d-1** (0.10 mmol), **2a** (0.20 mmol, 2.0 eq), [Ni] (10 mol%), (*S*)-**L6** (10 mol%), **P7** (20 mol%), $EtCO_2Ag$ (0.20 mmol, 2.0 eq), dry DME (0.5 mL), 100 °C, 24 h. [a]^{1}H NMR yield using 1,3,5-trimethoxybenzene as internal standard. [b]The value of ee was determined by HPLC. [c]Without (*S*)-**L6**.

<u>**Table S6.** Further screening of DG[ab]</u>

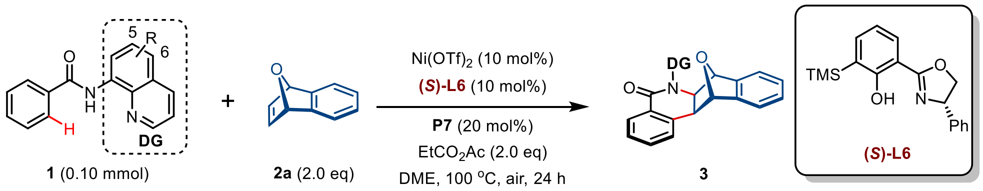

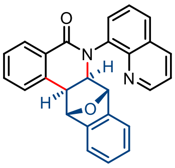

3a-1, 44%, 89% ee

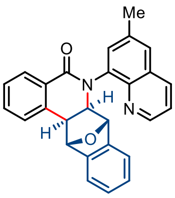

3b-1, 42%, 86% ee

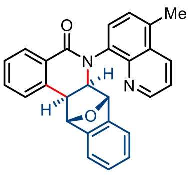

3c-1, 63%, 83% ee

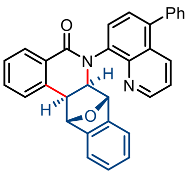

3d-1, 69%, 90% ee

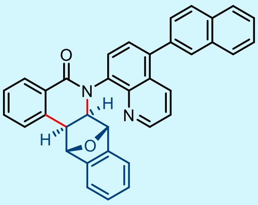

3g-1, 72%, 90% ee
78%, 92% ee[c]

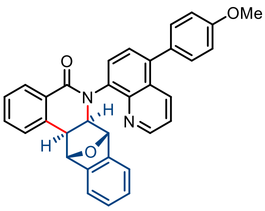

3h-1, 70%, 86% ee

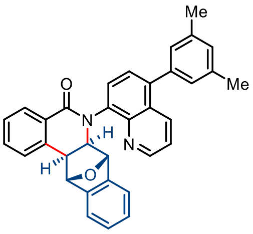

3i-1, 66%, 88% ee

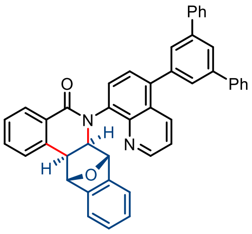

3j-1, 62%, 90% ee

Reaction conditions: **1** (0.10 mmol), **2a** (0.20 mmol, 2.0 eq), $Ni(OTf)_2$ (10 mol%), (*S*)-**L6** (10 mol%), **P7** (20 mol%), $EtCO_2Ag$ (0.20 mmol, 2.0 eq), dry DME (0.5 mL), 100 °C, 24 h. [a]Isolated yield. [b]The value of ee was determined by HPLC. [c]Using 20 mol% (*S*)-**L6**.

3.2 Reaction procedure and characterization for Nickel(II)/Salox-catalyzed C−H activation/asymmetric alkene insertion reaction

Reaction procedure and characterization of **3a-1**

To a 10 mL Schlenk tube was added **1a-1** (24.8 mg, 0.10 mmol), $Ni(OTf)_2$ (3.6 mg, 10 mol%), (*S*)-**L6** (3.1 mg, 10 mol%), $EtCO_2Ag$ (36.0 mg, 0.20 mmol, 2.0 eq), **P7** (8.5 mg, 20 mol%) and **2a** (28.8 mg, 0.20 mmol, 2.0 eq), followed by addition of anhydrous DME (0.5 mL). Then the mixture was stirred at 100 °C for 24 h. After cooling to room temperature, the reaction system was quenched with aq. $NaHCO_3$ (10 mL) and extracted with CH_2Cl_2 (3×15 mL). The combined organic layer was dried over anhydrous Na_2SO_4, filtered, and concentrated in vacuo. After concentration, the crude product was purified by preparative TLC (PE/EtOAc = 1/1, v/v, R_f = 0.29) to afford **3a-1** (17.3 mg, 44% yield, 89% ee) as a light-yellow oil.

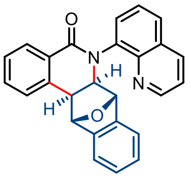

3a-1: $[\alpha]_D^{20}$ = +55.0 (c = 0.58, $CHCl_3$, 89% ee); **^{1}H NMR (400 MHz, $CDCl_3$)** δ 8.80 (brs, 1H), 8.25 (t, *J* = 8.6 Hz, 2H), 7.95 – 7.93 (m, 2H), 7.73 (brs, 1H), 7.61 (t, *J* = 7.5 Hz, 1H), 7.52 (d, *J* = 7.7 Hz, 1H), 7.42 – 7.39 (m, 3H), 7.21 (t, *J* = 7.5 Hz, 1H), 7.08 (t, *J* = 7.4 Hz, 1H), 6.95 (d, *J* = 7.3 Hz, 1H), 5.50 (s, 1H), 5.45 (s, 1H), 4.73 (brs, 1H), 3.69 (d, *J* = 8.6 Hz, 1H); **^{13}C NMR (101 MHz, $CDCl_3$)** δ 163.0, 150.7, 145.7, 143.8, 142.4, 138.2, 137.6, 136.5, 132.5, 130.0, 128.9, 128.6, 128.1, 127.6, 127.2, 127.1, 127.1, 126.5, 121.6, 120.4, 119.2, 88.8, 84.6, 63.4, 43.0; **HRMS (ESI)** calcd for $C_{26}H_{18}N_2O_2$ $[M+H]^+$: 391.1441, Found: 391.1443; **HPLC condition:** The enantiomeric excess was determined by Daicel Chiralcel IA, Hexanes/IPA = 50/50, 1.2 mL/min, λ = 254 nm, t (minor) = 15.149 min, t (major) = 22.611 min.

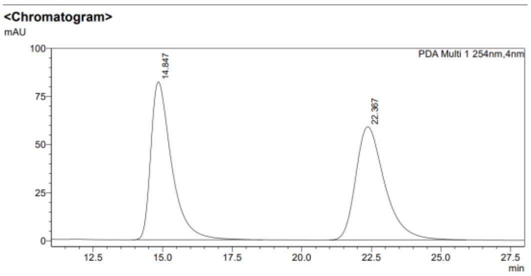

<Peak Table>
PDA Ch1 254nm

Peak#	Ret. Time	Area	Height	Area%
1	14.847	4268080	81981	50.027
2	22.367	4263409	58815	49.973
Total		8531490	140795	100.000

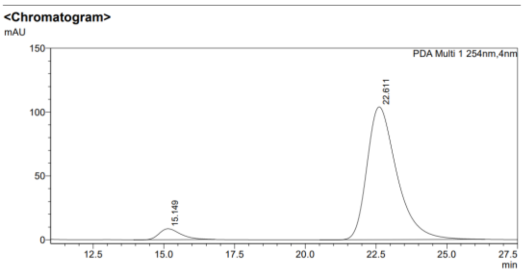

<Peak Table>
PDA Ch1 254nm

Peak#	Ret. Time	Area	Height	Area%
1	15.149	453836	8538	5.614
2	22.611	7629615	103940	94.386
Total		8083451	112478	100.000

Reaction procedure and characterization of **3b-1**

To a 10 mL Schlenk tube was added **1b-1** (26.2 mg, 0.10 mmol), $Ni(OTf)_2$ (3.6 mg, 10 mol%), (*S*)-**L6** (3.1 mg, 10 mol%), $EtCO_2Ag$ (36.0 mg, 0.20 mmol, 2.0 eq), **P7** (8.5 mg, 20 mol%) and **2a** (28.8 mg, 0.20 mmol, 2.0 eq), followed by addition of anhydrous DME (0.5 mL). Then the mixture was stirred at 100 °C for 24 h. After cooling to room temperature, the reaction system was quenched with aq. $NaHCO_3$ (10 mL) and extracted with CH_2Cl_2 (3×15 mL). The combined organic layer was dried over anhydrous Na_2SO_4, filtered, and concentrated in vacuo. After concentration, the crude product was purified by preparative TLC (PE/EtOAc = 1/1, v/v, R_f = 0.39) to afford **3b-1** (17.0 mg, 42% yield, 86% ee) as a yellow oil.

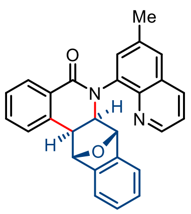

3b-1: $[\alpha]_D^{20}$ = +66.4 (c = 0.56, $CHCl_3$, 86% ee); **^{1}H NMR (400 MHz, $CDCl_3$)** δ 8.72 (brs, 1H), 8.26 (d, *J* = 7.8 Hz, 1H), 8.14 (d, *J* = 8.3 Hz, 1H), 7.81 (brs, 1H), 7.70 (s, 1H), 7.61 (t, *J* = 7.5 Hz, 1H), 7.51 (d, *J* = 7.7 Hz, 1H), 7.42 – 7.36 (m, 3H), 7.21 (t, *J* = 7.4 Hz, 1H), 7.08 (t, *J* = 7.4 Hz, 1H), 6.97 (d, *J* = 7.2 Hz, 1H), 5.51 (s, 1H), 5.45 (s, 1H), 4.76 (brs, 1H), 3.68 (d, *J* = 8.6 Hz, 1H), 2.65 (s, 3H); **^{13}C NMR (101 MHz, $CDCl_3$)** δ 163.0, 149.8, 145.8, 142.4, 137.6, 136.5, 135.8, 134.5, 132.5, 130.0, 128.9, 128.0, 127.6, 127.5, 127.2, 127.1, 127.1, 121.6, 120.5, 119.2, 88.8, 84.5, 63.2, 43.0, 21.7; **HRMS (ESI)** calcd for $C_{27}H_{20}N_2O_2$ $[M+H]^+$: 405.1598, Found: 405.1601; **HPLC condition:** The enantiomeric excess was determined by Daicel Chiralcel IC, Hexanes/IPA = 50/50, 1.2 mL/min, λ = 254 nm, t (major) = 26.450 min, t (minor) = 89.431 min.

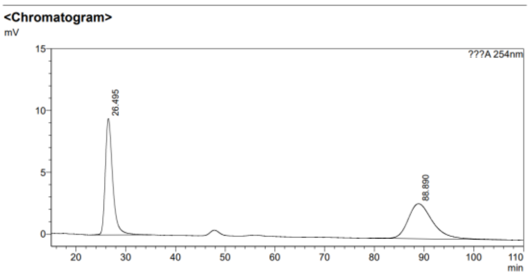

<Peak Table>

???A 254nm

Peak#	Ret. Time	Area	Height	Area%
1	26.495	977105	9422	50.052
2	88.890	975069	2837	49.948
Total		1952173	12258	100.000

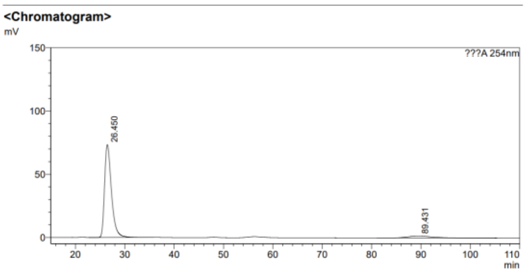

<Peak Table>

???A 254nm

Peak#	Ret. Time	Area	Height	Area%
1	26.450	7132606	73417	93.109
2	89.431	527846	1493	6.891
Total		7660452	74909	100.000

Reaction procedure and characterization of **3c-1**

To a 10 mL Schlenk tube was added **1c-1** (26.2 mg, 0.10 mmol), $Ni(OTf)_2$ (3.6 mg, 10 mol%), (*S*)-**L6** (3.1 mg, 10 mol%), $EtCO_2Ag$ (36.0 mg, 0.20 mmol, 2.0 eq), **P7** (8.5 mg, 20 mol%) and **2a** (28.8 mg, 0.20 mmol, 2.0 eq), followed by addition of anhydrous DME (0.5 mL). Then the mixture was stirred at 100 °C for 24 h. After cooling to room temperature, the reaction system was quenched with aq. $NaHCO_3$ (10 mL) and extracted with CH_2Cl_2 (3×15 mL). The combined organic layer was dried over anhydrous Na_2SO_4, filtered, and concentrated in vacuo. After concentration, the crude product was purified by preparative TLC (PE/EtOAc = 1/1, v/v, R_f = 0.31) to afford **3c-1** (25.4 mg, 63% yield, 83% ee) as a colorless oil.

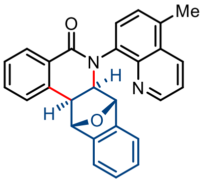

3c-1: $[\alpha]_D^{20}$ = +38.6 (c = 1.03, $CHCl_3$, 83% ee); **^{1}H NMR (400 MHz, $CDCl_3$)** δ 8.78 (brs, 1H), 8.38 (d, *J* = 8.5 Hz, 1H), 8.26 (d, *J* = 7.8 Hz, 1H), 7.85 (brs, 1H), 7.64 – 7.49 (m, 3H), 7.44 – 7.38 (m, 3H), 7.21 (t, *J* = 7.4 Hz, 1H), 7.07 (t, *J* = 7.4 Hz, 1H), 6.96 (d, *J* = 7.2 Hz, 1H), 5.52 (s, 1H), 5.45 (s, 1H), 4.72 (brs, 1H), 3.68 (d, *J* = 8.6 Hz, 1H), 2.77 (s, 3H); **^{13}C NMR (101 MHz, $CDCl_3$)** δ 163.1, 150.1, 145.8, 143.9, 142.5, 137.6, 136.2, 135.6, 133.1, 132.4, 131.9, 129.2, 128.9, 128.0, 127.6, 127.2, 127.1, 126.9, 121.1, 120.4, 119.2, 88.8, 84.6, 63.4, 43.0, 18.9; **HRMS (ESI)** calcd for $C_{27}H_{20}N_2O_2$ $[M+H]^+$: 405.1598, Found: 405.1599; **HPLC condition:** The enantiomeric excess was determined by Daicel Chiralcel IA, Hexanes/IPA = 50/50, 1.2 mL/min, λ = 254 nm, t (minor) = 20.654 min, t (major) = 33.483 min.

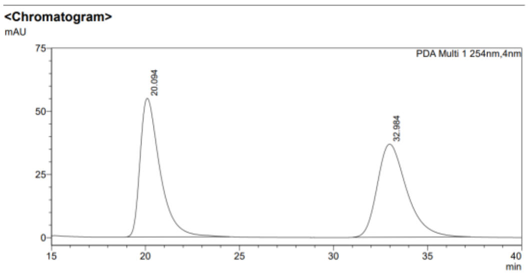

<Peak Table>
PDA Ch1 254nm

Peak#	Ret. Time	Area	Height	Area%
1	20.094	4032739	54847	50.147
2	32.984	4009031	36799	49.853
Total		8041769	91647	100.000

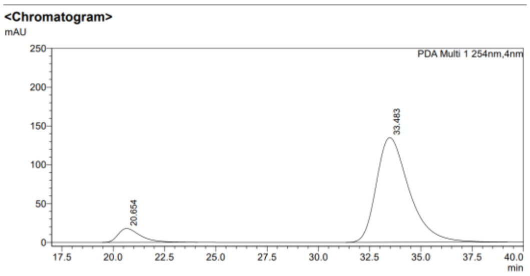

<Peak Table>
PDA Ch1 254nm

Peak#	Ret. Time	Area	Height	Area%
1	20.654	1364073	17906	8.327
2	33.483	15017084	134891	91.673
Total		16381157	152797	100.000

<u>Reaction procedure and characterization of **3d-1**</u>

To a 10 mL Schlenk tube was added **1d-1** (32.4 mg, 0.10 mmol), Ni(OTf)$_2$ (3.6 mg, 10 mol%), (*S*)-**L6** (3.1 mg, 10 mol%), EtCO$_2$Ag (36.0 mg, 0.20 mmol, 2.0 eq), **P7** (8.5 mg, 20 mol%) and **2a** (28.8 mg, 0.20 mmol, 2.0 eq), followed by addition of anhydrous DME (0.5 mL). Then the mixture was stirred at 100 °C for 24 h. After cooling to room temperature, the reaction system was quenched with aq. NaHCO$_3$ (10 mL) and extracted with CH$_2$Cl$_2$ (3×15 mL). The combined organic layer was dried over anhydrous Na$_2$SO$_4$, filtered, and concentrated in vacuo. After concentration, the crude product was purified by preparative TLC (PE/EtOAc = 2/1, v/v, R$_f$ = 0.32) to afford **3d-1** (32.0 mg, 69% yield, 90% ee) as a light-yellow oil.

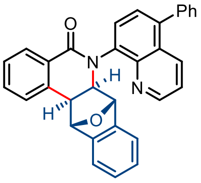

3d-1: $[\alpha]_D^{20}$ = -14.3 (c = 0.97, CHCl$_3$, 90% ee); **<u>^{1}H NMR (400 MHz, CDCl$_3$)</u>** δ 8.79 (brs, 1H), 8.32 – 8.28 (m, 2H), 8.02 (brs, 1H), 7.69 (brs, 1H), 7.62 (t, *J* = 7.5 Hz, 1H), 7.59 – 7.47 (m, 6H), 7.44 – 7.40 (m, 2H), 7.36 (brs, 1H), 7.23 (t, *J* = 7.5 Hz, 1H), 7.11 (t, *J* = 7.4 Hz, 1H), 7.04 (d, *J* = 7.2 Hz, 1H), 5.59 (s, 1H), 5.47 (s, 1H), 4.80 (brs, 1H), 3.73 (d, *J* = 8.5 Hz, 1H); **<u>^{13}C NMR (101 MHz, CDCl$_3$)</u>** δ 163.2, 150.4, 145.8, 144.0, 142.5, 141.1, 139.0, 137.7, 137.3, 135.0, 132.5, 131.8, 130.1, 129.0, 128.6, 128.4, 128.1, 128.0, 127.6, 127.2, 127.2, 127.1, 121.5, 120.5, 119.2, 88.9, 84.7, 63.4, 43.1; **<u>HRMS (ESI)</u>** calcd for C$_{32}$H$_{22}$N$_2$O$_2$ [M+H]$^+$: 467.1754, Found: 467.1756; **<u>HPLC condition:</u>** The enantiomeric excess was determined by Daicel Chiralcel IA, Hexanes/IPA = 50/50, 1.2 mL/min, λ = 254 nm, t (minor) = 21.646 min, t (major) = 39.733 min.

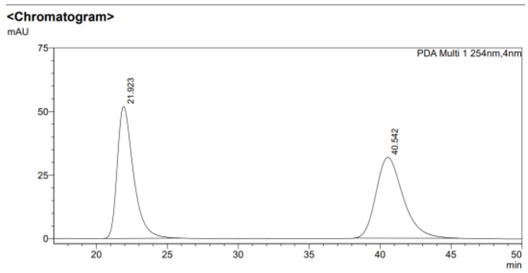

<Peak Table>

PDA Ch1 254nm

Peak#	Ret. Time	Area	Height	Area%
1	21.923	4106580	51857	49.301
2	40.542	4223037	31749	50.699
Total		8329618	83606	100.000

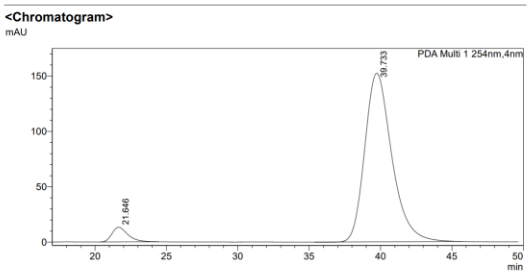

<Peak Table>

PDA Ch1 254nm

Peak#	Ret. Time	Area	Height	Area%
1	21.646	1024886	13222	4.855
2	39.733	20085479	152365	95.145
Total		21110365	165587	100.000

Reaction procedure and characterization of **3e-1**

To a 10 mL Schlenk tube was added **1e-1** (28.2 mg, 0.10 mmol), Ni(OTf)$_2$ (3.6 mg, 10 mol%), (*S*)-**L1** (2.4 mg, 10 mol%), AgOAc (33.4 mg, 0.20 mmol, 2.0 eq) and **2a** (28.8 mg, 0.20 mmol, 2.0 eq), followed by addition of anhydrous DME (0.5 mL). Then the mixture was stirred at 100 °C for 24 h. After cooling to room temperature, the reaction system was quenched with aq. $NaHCO_3$ (10 mL) and extracted with CH_2Cl_2 (3×15 mL). The combined organic layer was dried over anhydrous Na_2SO_4, filtered, and concentrated in vacuo. After concentration, the residue was analyzed by ^{1}H NMR, using 1,3,5-trimethoxybenzene as internal standard. Product **3e-1** was obtained in 16% yield with 69% ee.

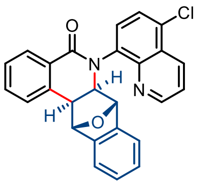

3e-1: **^{1}H NMR (400 MHz, CDCl$_3$)** δ 8.84 (brs, 1H), 8.65 (d, *J* = 8.6 Hz, 1H), 8.25 (d, *J* = 7.8 Hz, 1H), 8.01 – 7.73 (m, 2H), 7.62 (t, *J* = 7.6 Hz, 1H), 7.52 (d, *J* = 7.8 Hz, 2H), 7.42 – 7.39 (m, 2H), 7.22 (t, *J* = 7.5 Hz, 1H), 7.09 (t, *J* = 7.4 Hz, 1H), 6.98 (d, *J* = 7.3 Hz, 1H), 5.47 (s, 1H), 5.45 (s, 1H), 4.68 (brs, 1H), 3.69 (d, *J* = 8.6 Hz, 1H); **^{13}C NMR (101 MHz, CDCl$_3$)** δ 163.2, 151.3, 145.7, 144.4, 142.3, 137.7, 137.4, 133.6, 132.7, 132.3, 131.8, 128.9, 128.1, 128.0, 127.7, 127.3, 126.9, 126.7, 122.5, 120.5, 119.3, 88.8, 84.5, 63.5, 43.1; **HRMS (ESI)** calcd for $C_{26}H_{17}ClN_2O_2$ $[M+H]^+$: 425.1052, Found: 425.1051; **HPLC condition:** The enantiomeric excess was determined by Daicel Chiralcel IA, Hexanes/IPA = 50/50, 1.6 mL/min, λ = 254 nm, t (minor) = 25.339 min, t (major) = 39.527 min.

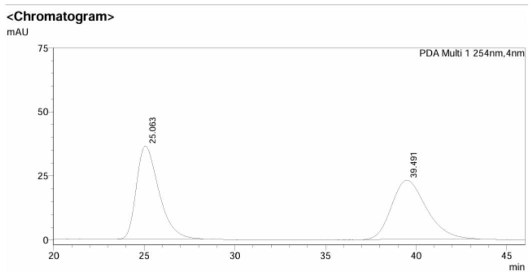

<Peak Table>

PDA Ch1 254nm

Peak#	Ret. Time	Area	Height	Area%
1	25.063	3137172	36301	50.329
2	39.491	3096140	23016	49.671
Total		6233312	59317	100.000

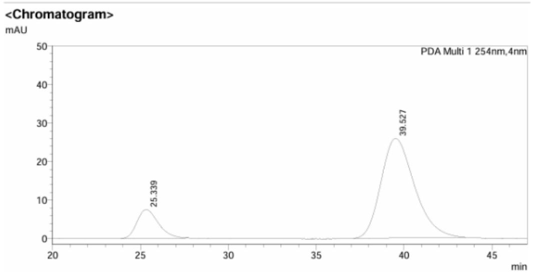

<Peak Table>

PDA Ch1 254nm

Peak#	Ret. Time	Area	Height	Area%
1	25.339	631060	7463	15.562
2	39.527	3424148	25763	84.438
Total		4055208	33226	100.000

<u>Reaction procedure and characterization of **3f-1**</u>

To a 10 mL Schlenk tube was added **1f-1** (27.8 mg, 0.10 mmol), Ni(OTf)$_2$ (3.6 mg, 10 mol%), (*S*)-**L1** (2.4 mg, 10 mol%), AgOAc (33.4 mg, 0.20 mmol, 2.0 eq) and **2a** (28.8 mg, 0.20 mmol, 2.0 eq), followed by addition of anhydrous DME (0.5 mL). Then the mixture was stirred at 100 °C for 24 h. After cooling to room temperature, the reaction system was quenched with aq. NaHCO$_3$ (10 mL) and extracted with CH$_2$Cl$_2$ (3×15 mL). The combined organic layer was dried over anhydrous Na$_2$SO$_4$, filtered, and concentrated in vacuo. After concentration, the residue was analyzed by ^{1}H NMR, using 1,3,5-trimethoxybenzene as internal standard. Product **3f-1** was obtained in 16% yield with 86% ee.

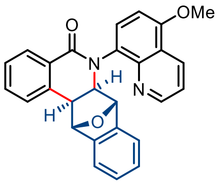

3f-1: **<u>^{1}H NMR (400 MHz, CDCl$_3$)</u>** δ 8.76 (brs, 1H), 8.64 (d, *J* = 8.5 Hz, 1H), 8.26 (d, *J* = 7.8 Hz, 1H), 7.87 (brs, 1H), 7.60 (t, *J* = 7.5 Hz, 1H), 7.51 (d, *J* = 7.8 Hz, 1H), 7.39 (t, *J* = 7.7 Hz, 3H), 7.21 (t, *J* = 7.4 Hz, 1H), 7.12 – 6.93 (m, 3H), 5.53 (s, 1H), 5.44 (s, 1H), 4.69 (brs, 1H), 4.09 (s, 3H), 3.67 (d, *J* = 8.6 Hz, 1H); **<u>^{13}C NMR (101 MHz, CDCl$_3$)</u>** δ 163.2, 155.1, 150.8, 145.7, 144.2, 142.4, 137.5, 132.3, 132.2, 131.3, 130.4, 128.9, 128.0, 127.5, 127.1, 127.0, 122.1, 120.5, 120.4, 119.1, 104.0, 88.8, 84.6, 63.2, 55.9, 42.9; **<u>HRMS (ESI)</u>** calcd for $C_{27}H_{20}N_2O_3$ [M+Na]$^+$: 443.1365, Found: 443.1366; **<u>HPLC condition:</u>** The enantiomeric excess was determined by Daicel Chiralcel IA, Hexanes/IPA = 50/50, 1.6 mL/min, λ = 254 nm, t (minor) = 18.061 min, t (major) = 22.507 min.

<Chromatogram>

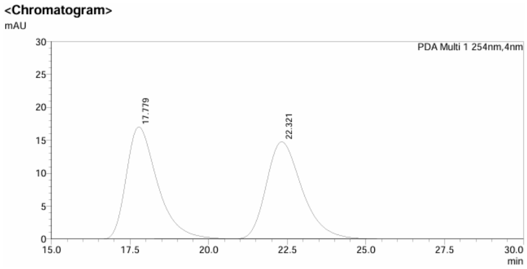

<Peak Table>

PDA Ch1 254nm

Peak#	Ret. Time	Area	Height	Area%
1	17.779	1178520	17050	50.370
2	22.321	1161202	14706	49.630
Total		2339722	31756	100.000

<Chromatogram>

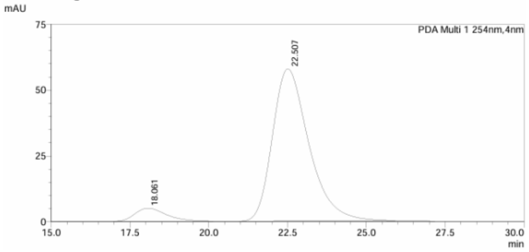

<Peak Table>

PDA Ch1 254nm

Peak#	Ret. Time	Area	Height	Area%
1	18.061	345057	4979	6.919
2	22.507	4642145	57715	93.081
Total		4987201	62694	100.000

Reaction procedure and characterization of **3g-1**

To a 10 mL Schlenk tube was added **1g-1** (37.4 mg, 0.10 mmol), $Ni(OTf)_2$ (3.6 mg, 10 mol%), (*S*)-**L6** (6.2 mg, 20 mol%), $EtCO_2Ag$ (36.0 mg, 0.20 mmol, 2.0 eq), **P7** (8.5 mg, 20 mol%) and **2a** (28.8 mg, 0.20 mmol, 2.0 eq), followed by addition of anhydrous DME (0.5 mL). Then the mixture was stirred at 100 °C for 24 h. After cooling to room temperature, the reaction system was quenched with aq. $NaHCO_3$ (10 mL) and extracted with CH_2Cl_2 (3×15 mL). The combined organic layer was dried over anhydrous Na_2SO_4, filtered, and concentrated in vacuo. After concentration, the crude product was purified by preparative TLC (PE/EtOAc = 2/1, v/v, R_f = 0.24) to afford **3g-1** (40.6 mg, 78% yield, 92% ee) as a light-yellow solid.

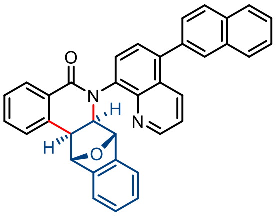

3g-1: $[\alpha]_D^{20}$ = -48.4 (c = 1.01, $CHCl_3$, 92% ee); **^{1}H NMR (400 MHz, $CDCl_3$)** δ 8.85 (brs, 1H), 8.39 – 8.34 (m, 2H), 8.15 – 7.94 (m, 5H), 7.83 (brs, 1H), 7.71 (d, *J* = 8.5 Hz, 1H), 7.69 – 7.58 (m, 4H), 7.49 – 7.45 (m, 2H), 7.40 (brs, 1H), 7.29 (d, *J* = 7.6 Hz, 1H), 7.16 (t, *J* = 7.4 Hz, 1H), 7.10 (d, *J* = 7.2 Hz, 1H), 5.66 (s, 1H), 5.53 (s, 1H), 4.87 (brs, 1H), 3.78 (d, *J* = 8.5 Hz, 1H); **^{13}C NMR (101 MHz, $CDCl_3$)** δ 163.2, 150.5, 145.8, 144.0, 142.5, 141.1, 137.7, 137.4, 136.5, 135.1, 133.4, 132.8, 132.6, 131.9, 129.1, 129.0, 128.5, 128.2, 128.1, 127.9, 127.7, 127.5, 127.2, 127.1, 126.7, 126.6, 121.6, 120.5, 119.2, 88.9, 84.7, 63.4, 43.1; **HRMS (ESI)** calcd for $C_{36}H_{24}N_2O_2$ $[M+H]^+$: 517.1911, Found: 517.1913; **HPLC condition:** The enantiomeric excess was determined by Daicel Chiralcel IA, Hexanes/IPA = 50/50, 1.2 mL/min, λ = 254 nm, t (minor) = 29.353 min, t (major) = 65.339 min.

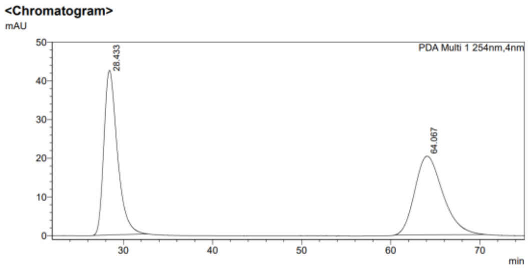

<Peak Table>

PDA Ch1 254nm

Peak#	Ret. Time	Area	Height	Area%
1	28.433	4496303	42571	50.157
2	64.067	4468181	20332	49.843
Total		8964483	62903	100.000

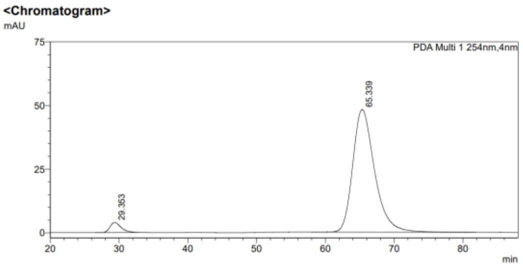

<Peak Table>

PDA Ch1 254nm

Peak#	Ret. Time	Area	Height	Area%
1	29.353	450542	3977	4.067
2	65.339	10627271	48146	95.933
Total		11077814	52123	100.000

Reaction procedure and characterization of **3h-1**

To a 10 mL Schlenk tube was added **1h-1** (35.4 mg, 0.10 mmol), Ni(OTf)$_2$ (3.6 mg, 10 mol%), (*S*)-**L6** (3.1 mg, 10 mol%), $EtCO_2Ag$ (36.0 mg, 0.20 mmol, 2.0 eq), **P7** (8.5 mg, 20 mol%) and **2a** (28.8 mg, 0.20 mmol, 2.0 eq), followed by addition of anhydrous DME (0.5 mL). Then the mixture was stirred at 100 °C for 24 h. After cooling to room temperature, the reaction system was quenched with aq. $NaHCO_3$ (10 mL) and extracted with CH_2Cl_2 (3×15 mL). The combined organic layer was dried over anhydrous Na_2SO_4, filtered, and concentrated in vacuo. After concentration, the crude product was purified by preparative TLC (PE/EtOAc = 1/1, v/v, R_f = 0.42) to afford **3h-1** (34.7 mg, 70% yield, 86% ee) as a light-yellow oil.

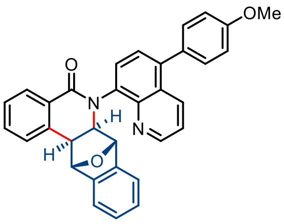

3h-1: $[\alpha]_D^{20}$ = -25.3 (c = 0.85, $CHCl_3$, 86% ee); **<u>^{1}H NMR (400 MHz, CDCl$_3$)</u>** δ 8.77 (brs, 1H), 8.34 – 8.28 (m, 2H), 8.00 (brs, 1H), 7.70 – 7.58 (m, 2H), 7.54 (d, *J* = 7.8 Hz, 1H), 7.48 (d, *J* = 8.1 Hz, 2H), 7.43 – 7.39 (m, 2H), 7.36 (brs, 1H), 7.22 (t, *J* = 7.4 Hz, 1H), 7.09 (d, *J* = 7.9 Hz, 3H), 7.03 (d, *J* = 7.3 Hz, 1H), 5.59 (s, 1H), 5.47 (s, 1H), 4.80 (brs, 1H), 3.92 (s, 3H), 3.72 (d, *J* = 8.6 Hz, 1H); **<u>^{13}C NMR (101 MHz, CDCl$_3$)</u>** δ 163.2, 159.5, 150.3, 145.8, 144.0, 142.5, 140.8, 137.7, 137.0, 135.1, 132.5, 131.8, 131.4, 131.3, 129.0, 128.5, 128.1, 127.6, 127.2, 127.1, 121.4, 120.5, 119.2, 114.1, 88.9, 84.7, 63.4, 55.5, 43.1; **<u>HRMS (ESI)</u>** calcd for $C_{33}H_{24}N_2O_3$ $[M+H]^+$: 497.1860, Found: 497.1862; **<u>HPLC condition:</u>** The enantiomeric excess was determined by Daicel Chiralcel IA, Hexanes/IPA = 50/50, 1.2 mL/min, λ = 254 nm, t (minor) = 22.380 min, t (major) = 37.525 min.

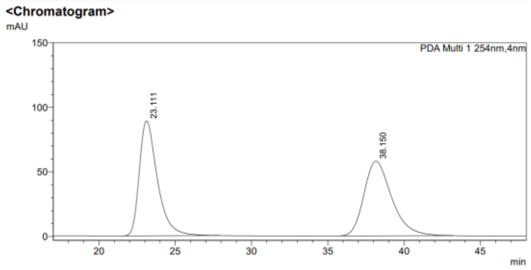

<Peak Table>

PDA Ch1 254nm

Peak#	Ret. Time	Area	Height	Area%
1	23.111	7343083	88959	50.128
2	38.150	7305702	57722	49.872
Total		14648785	146682	100.000

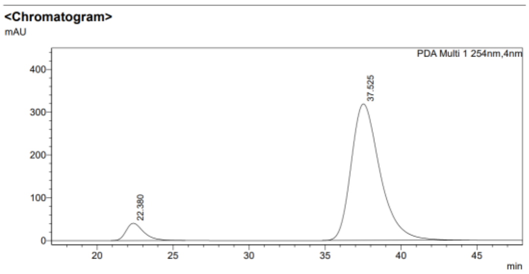

<Peak Table>

PDA Ch1 254nm

Peak#	Ret. Time	Area	Height	Area%
1	22.380	3245042	40375	7.147
2	37.525	42160791	319194	92.853
Total		45405834	359569	100.000

Reaction procedure and characterization of **3i-1**

To a 10 mL Schlenk tube was added **1i-1** (35.2 mg, 0.10 mmol), $Ni(OTf)_2$ (3.6 mg, 10 mol%), (*S*)-**L6** (3.1 mg, 10 mol%), $EtCO_2Ag$ (36.0 mg, 0.20 mmol, 2.0 eq), **P7** (8.5 mg, 20 mol%) and **2a** (28.8 mg, 0.20 mmol, 2.0 eq), followed by addition of anhydrous DME (0.5 mL). Then the mixture was stirred at 100 °C for 24 h. After cooling to room temperature, the reaction system was quenched with aq. $NaHCO_3$ (10 mL) and extracted with CH_2Cl_2 (3×15 mL). The combined organic layer was dried over anhydrous Na_2SO_4, filtered, and concentrated in vacuo. After concentration, the crude product was purified by preparative TLC (PE/EtOAc = 2/1, v/v, R_f = 0.36) to afford **3i-1** (32.8 mg, 66% yield, 88% ee) as a light-yellow oil.

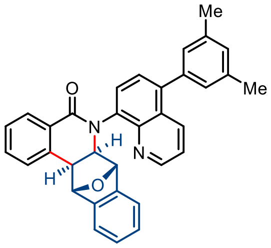

3i-1: $[\alpha]_D^{20}$ = -25.2 (c = 1.03, $CHCl_3$, 88% ee); **^{1}H NMR (400 MHz, $CDCl_3$)** δ 8.78 (brs, 1H), 8.34 (d, *J* = 8.5 Hz, 1H), 8.30 (dd, *J* = 7.9, 1.4 Hz, 1H), 8.00 (brs, 1H), 7.68 (brs, 1H), 7.62 (t, *J* = 7.5 Hz, 1H), 7.54 (d, *J* = 7.7 Hz, 1H), 7.44 – 7.40 (m, 2H), 7.36 (brs, 1H), 7.23 (t, *J* = 7.4 Hz, 1H), 7.16 (d, *J* = 7.7 Hz, 3H), 7.10 (t, *J* = 7.4 Hz, 1H), 7.03 (d, *J* = 7.3 Hz, 1H), 5.58 (s, 1H), 5.47 (s, 1H), 4.80 (brs, 1H), 3.72 (d, *J* = 8.6 Hz, 1H), 2.45 (s, 6H); **^{13}C NMR (101 MHz, $CDCl_3$)** δ 163.2, 150.3, 145.8, 143.9, 142.5, 141.4, 139.0, 138.2, 137.7, 137.1, 135.2, 132.5, 131.8, 129.6, 129.0, 128.4, 128.1, 127.9, 127.6, 127.2, 127.0, 121.4, 120.5, 119.2, 88.9, 84.7, 63.4, 43.1, 21.5; **HRMS (ESI)** calcd for $C_{34}H_{26}N_2O_2$ $[M+H]^+$: 495.2067, Found: 495.2069; **HPLC condition:** The enantiomeric excess was determined by Daicel Chiralcel IA, Hexanes/IPA = 50/50, 1.2 mL/min, λ = 254 nm, t (minor) = 17.820 min, t (major) = 33.973 min.

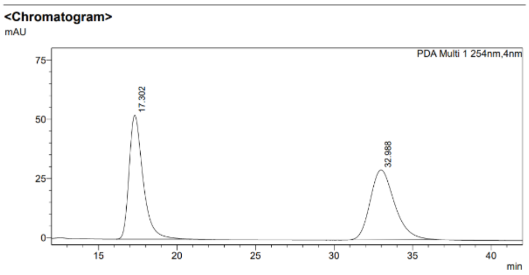

<Peak Table>
PDA Ch1 254nm

Peak#	Ret. Time	Area	Height	Area%
1	17.302	3220805	52222	50.327
2	32.988	3178891	29443	49.673
Total		6399695	81665	100.000

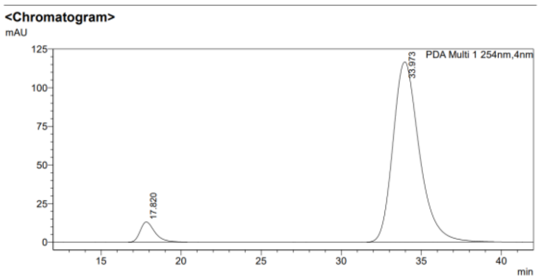

<Peak Table>
PDA Ch1 254nm

Peak#	Ret. Time	Area	Height	Area%
1	17.820	831775	13061	5.870
2	33.973	13338905	116495	94.130
Total		14170680	129556	100.000

Reaction procedure and characterization of **3j-1**

To a 10 mL Schlenk tube was added **1j-1** (47.6 mg, 0.10 mmol), $Ni(OTf)_2$ (3.6 mg, 10 mol%), (*S*)-**L6** (3.1 mg, 10 mol%), $EtCO_2Ag$ (36.0 mg, 0.20 mmol, 2.0 eq), **P7** (8.5 mg, 20 mol%) and **2a** (28.8 mg, 0.20 mmol, 2.0 eq), followed by addition of anhydrous DME (0.5 mL). Then the mixture was stirred at 100 °C for 24 h. After cooling to room temperature, the reaction system was quenched with aq. $NaHCO_3$ (10 mL) and extracted with CH_2Cl_2 (3×15 mL). The combined organic layer was dried over anhydrous Na_2SO_4, filtered, and concentrated in vacuo. After concentration, the crude product was purified by preparative TLC (PE/EtOAc = 2/1, v/v, R_f = 0.30) to afford **3j-1** (38.3 mg, 62% yield, 90% ee) as a light-yellow solid.

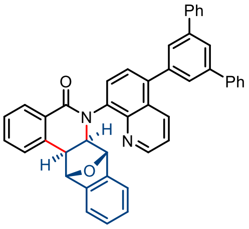

3j-1: $[\alpha]_D^{20}$ = -52.7 (c = 1.07, $CHCl_3$, 90% ee); **<u>^{1}H NMR (400 MHz, $CDCl_3$)</u>** δ 8.86 (brs, 1H), 8.47 (d, *J* = 8.6 Hz, 1H), 8.35 (d, *J* = 7.8 Hz, 1H), 8.10 (brs, 1H), 8.00 (s, 1H), 7.86 (brs, 1H), 7.83 – 7.76 (m, 6H), 7.67 (t, *J* = 7.5 Hz, 1H), 7.60 – 7.54 (m, 5H), 7.49 – 7.42 (m, 5H), 7.29 (d, *J* = 7.8 Hz, 1H), 7.15 (t, *J* = 7.4 Hz, 1H), 7.09 (d, *J* = 7.3 Hz, 1H), 5.66 (s, 1H), 5.53 (s, 1H), 4.87 (brs, 1H), 3.78 (d, *J* = 8.5 Hz, 1H); **<u>^{13}C NMR (101 MHz, $CDCl_3$)</u>** δ 163.2, 150.5, 145.8, 144.0, 142.5, 142.2, 140.9, 140.7, 140.1, 137.7, 135.1, 132.6, 131.9, 129.0, 129.0, 128.5, 128.1, 127.9, 127.8, 127.7, 127.4, 127.3, 127.2, 127.1, 125.7, 121.7, 120.5, 119.2, 88.9, 84.7, 63.4, 43.1; **<u>HRMS (ESI)</u>** calcd for $C_{44}H_{30}N_2O_2$ $[M+H]^+$: 619.2380, Found: 619.2384; **<u>HPLC condition:</u>** The enantiomeric excess was determined by Daicel Chiralcel IA, Hexanes/IPA = 50/50, 1.2 mL/min, λ = 254 nm, t (minor) = 40.146 min, t (major) = 77.949 min.

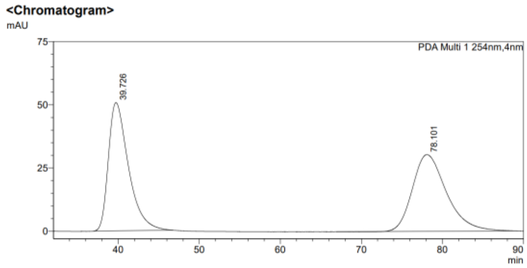

<Peak Table>
PDA Ch1 254nm

Peak#	Ret. Time	Area	Height	Area%
1	39.726	8731474	50693	49.555
2	78.101	8888150	30357	50.445
Total		17619623	81050	100.000

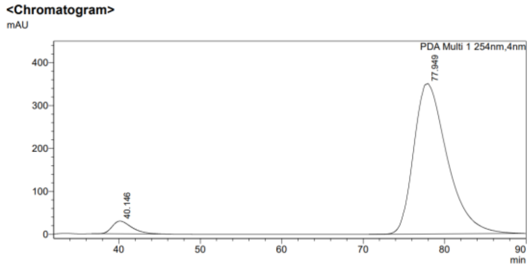

<Peak Table>
PDA Ch1 254nm

Peak#	Ret. Time	Area	Height	Area%
1	40.146	5049971	29799	4.789
2	77.949	100392877	350569	95.211
Total		105442848	380368	100.000

Reaction procedure and characterization of **3g-2**

To a 10 mL Schlenk tube was added **1g-2** (38.8 mg, 0.10 mmol), Ni(OTf)$_2$ (3.6 mg, 10 mol%), (*S*)-**L6** (6.2 mg, 20 mol%), EtCO$_2$Ag (36.0 mg, 0.20 mmol, 2.0 eq), **P7** (8.5 mg, 20 mol%) and **2a** (28.8 mg, 0.20 mmol, 2.0 eq), followed by addition of anhydrous DME (0.5 mL). Then the mixture was stirred at 100 °C for 24 h. After cooling to room temperature, the reaction system was quenched with aq. NaHCO$_3$ (10 mL) and extracted with CH$_2$Cl$_2$ (3×15 mL). The combined organic layer was dried over anhydrous Na$_2$SO$_4$, filtered, and concentrated in vacuo. After concentration, the crude product was purified by preparative TLC (PE/EtOAc = 2/1, v/v, R$_f$ = 0.58) to afford **3g-2** (24.0 mg, 45% yield, 96% ee) as a light-yellow oil.

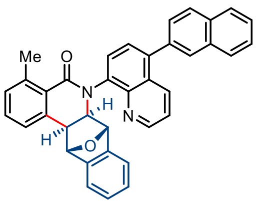

3g-2: $[\alpha]_D^{20}$ = -64.5 (c = 0.88, CHCl$_3$, 96% ee); **^{1}H NMR (400 MHz, CDCl$_3$)** δ 8.83 (brs, 1H), 8.34 (dd, *J* = 8.6, 1.7 Hz, 1H), 8.11 – 7.90 (m, 5H), 7.79 (brs, 1H), 7.67 (dd, *J* = 8.4, 1.7 Hz, 1H), 7.63 – 7.55 (m, 2H), 7.48 (t, *J* = 7.6 Hz, 1H), 7.45 – 7.33 (m, 3H), 7.26 – 7.19 (m, 2H), 7.11 (t, *J* = 7.3 Hz, 1H), 7.07 (d, *J* = 7.2 Hz, 1H), 5.61 (s, 1H), 5.45 (s, 1H), 4.71 (brs, 1H), 3.74 (d, *J* = 8.7 Hz, 1H), 2.79 (s, 3H); **^{13}C NMR (101 MHz, CDCl$_3$)** δ 164.4, 150.6, 145.9, 144.1, 142.8, 142.5, 140.9, 139.6, 138.3, 136.7, 135.1, 133.4, 132.8, 132.0, 131.8, 131.6, 129.1, 128.6, 128.2, 128.2, 127.9, 127.6, 127.2, 126.8, 126.7, 126.6, 125.0, 121.6, 120.6, 119.2, 89.5, 85.0, 62.9, 43.7, 24.4; **HRMS (ESI)** calcd for C$_{37}$H$_{26}$N$_2$O$_2$ [M+H]$^+$: 531.2067, Found: 531.2069; **HPLC condition:** The enantiomeric excess was determined by Daicel Chiralcel IA, Hexanes/IPA = 50/50, 1.2 mL/min, λ = 254 nm, t (minor) = 12.296 min, t (major) = 15.818 min.

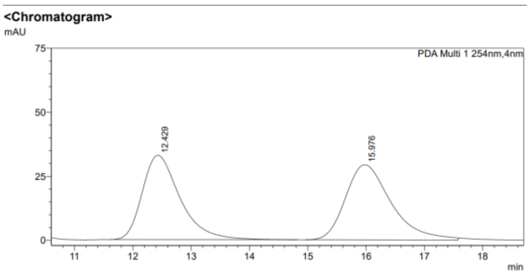

<Peak Table>
PDA Ch1 254nm

Peak#	Ret. Time	Area	Height	Area%
1	12.429	1483011	32888	48.154
2	15.976	1596721	29340	51.846
Total		3079732	62228	100.000

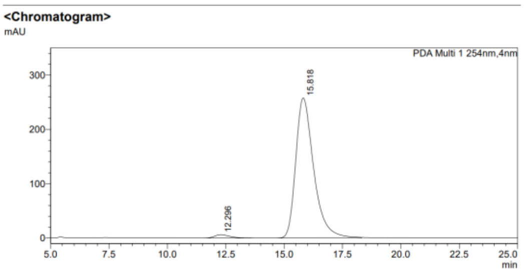

<Peak Table>
PDA Ch1 254nm

Peak#	Ret. Time	Area	Height	Area%
1	12.296	276424	6284	1.966
2	15.818	13783124	257592	98.034
Total		14059548	263875	100.000

Reaction procedure and characterization of **3g-3**

To a 10 mL Schlenk tube was added **1g-3** (38.8 mg, 0.10 mmol), $Ni(OTf)_2$ (3.6 mg, 10 mol%), (*S*)-**L6** (6.2 mg, 20 mol%), $EtCO_2Ag$ (36.0 mg, 0.20 mmol, 2.0 eq), **P7** (8.5 mg, 20 mol%) and **2a** (28.8 mg, 0.20 mmol, 2.0 eq), followed by addition of anhydrous DME (0.5 mL). Then the mixture was stirred at 100 °C for 24 h. After cooling to room temperature, the reaction system was quenched with aq. $NaHCO_3$ (10 mL) and extracted with CH_2Cl_2 (3×15 mL). The combined organic layer was dried over anhydrous Na_2SO_4, filtered, and concentrated in vacuo. After concentration, the crude product was purified by preparative TLC (PE/EtOAc = 2/1, v/v, R_f = 0.34) to afford **3g-3** (36.2 mg, 68% yield, 91% ee) as a light-yellow solid.

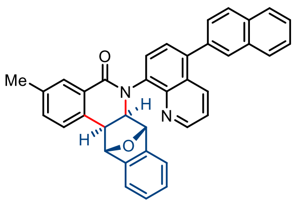

3g-3: $[\alpha]_D^{20}$ = -60.5 (c = 0.86, $CHCl_3$, 91% ee); **^{1}H NMR (400 MHz, CDCl$_3$)** δ 8.80 (brs, 1H), 8.34 (dd, *J* = 8.6, 1.7 Hz, 1H), 8.12 (s, 1H), 8.06 – 7.91 (m, 5H), 7.79 (brs, 1H), 7.68 (dd, *J* = 8.4, 1.7 Hz, 1H), 7.63 – 7.55 (m, 2H), 7.47 – 7.41 (m, 3H), 7.35 (brs, 1H), 7.23 (t, *J* = 7.6 Hz, 1H), 7.11 (t, *J* = 7.3 Hz, 1H), 7.05 (d, *J* = 7.2 Hz, 1H), 5.61 (s, 1H), 5.46 (s, 1H), 4.79 (brs, 1H), 3.70 (d, *J* = 8.6 Hz, 1H), 2.44 (s, 3H); **^{13}C NMR (101 MHz, CDCl$_3$)** δ 163.4, 150.5, 145.9, 144.1, 142.6, 141.0, 137.8, 136.9, 136.6, 135.1, 134.8, 133.5, 133.4, 132.9, 131.9, 129.2, 128.6, 128.2, 128.2, 128.1, 127.9, 127.6, 127.5, 127.2, 126.9, 126.7, 126.6, 121.6, 120.5, 119.3, 88.9, 84.7, 63.5, 42.8, 21.2; **HRMS (ESI)** calcd for $C_{37}H_{26}N_2O_2$ $[M+H]^+$: 531.2067, Found: 531.2070; **HPLC condition:** The enantiomeric excess was determined by Daicel Chiralcel IA, Hexanes/IPA = 50/50, 1.2 mL/min, λ = 254 nm, t (minor) = 51.432 min, t (major) = 80.931 min.

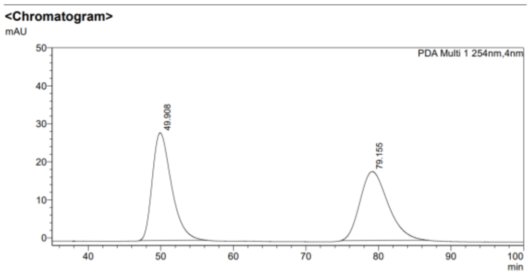

<Peak Table>
PDA Ch1 254nm

Peak#	Ret. Time	Area	Height	Area%
1	49.908	5199860	28321	51.520
2	79.155	4893111	18129	48.480
Total		10092970	46450	100.000

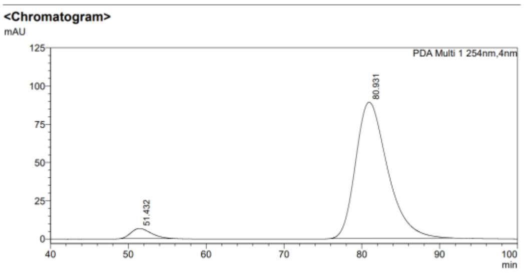

<Peak Table>
PDA Ch1 254nm

Peak#	Ret. Time	Area	Height	Area%
1	51.432	1215088	6664	4.562
2	80.931	25422698	89139	95.438
Total		26637786	95803	100.000

Reaction procedure and characterization of **3g-4**

To a 10 mL Schlenk tube was added **1g-4** (38.8 mg, 0.10 mmol), Ni(OTf)$_2$ (3.6 mg, 10 mol%), (*S*)-**L6** (6.2 mg, 20 mol%), EtCO$_2$Ag (36.0 mg, 0.20 mmol, 2.0 eq), **P7** (8.5 mg, 20 mol%) and **2a** (28.8 mg, 0.20 mmol, 2.0 eq), followed by addition of anhydrous DME (0.5 mL). Then the mixture was stirred at 100 °C for 24 h. After cooling to room temperature, the reaction system was quenched with aq. NaHCO$_3$ (10 mL) and extracted with CH$_2$Cl$_2$ (3×15 mL). The combined organic layer was dried over anhydrous Na$_2$SO$_4$, filtered, and concentrated in vacuo. After concentration, the crude product was purified by preparative TLC (PE/EtOAc = 2/1, v/v, R$_f$ = 0.26) to afford **3g-4** (37.5 mg, 71% yield, 91% ee) as a light-yellow oil.

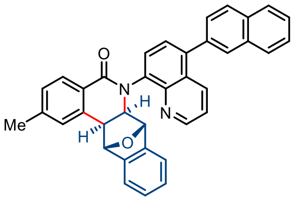

3g-4: $[\alpha]_D^{20}$ = -42.0 (c = 0.91, CHCl$_3$, 91% ee); **^{1}H NMR (400 MHz, CDCl$_3$)** δ 8.78 (brs, 1H), 8.31 (dd, *J* = 8.6, 1.7 Hz, 1H), 8.17 (d, *J* = 7.9 Hz, 1H), 8.07 – 7.87 (m, 5H), 7.76 (s, 1H), 7.65 (dd, *J* = 8.5, 1.7 Hz, 1H), 7.60 – 7.51 (m, 2H), 7.42 (d, *J* = 7.2 Hz, 1H), 7.32 – 7.30 (m, 2H), 7.24 – 7.20 (m, 2H), 7.09 (t, *J* = 7.4 Hz, 1H), 7.03 (d, *J* = 7.2 Hz, 1H), 5.59 (s, 1H), 5.47 (s, 1H), 4.79 (brs, 1H), 3.66 (d, *J* = 8.6 Hz, 1H), 2.49 (s, 3H); **^{13}C NMR (101 MHz, CDCl$_3$)** δ 163.4, 150.5, 145.9, 144.1, 143.0, 142.6, 141.0, 137.7, 136.6, 135.1, 133.4, 132.9, 132.0, 129.2, 129.1, 128.5, 128.2, 128.2, 127.9, 127.6, 127.5, 127.2, 126.7, 126.6, 124.7, 121.6, 120.5, 119.2, 88.9, 84.8, 63.5, 43.1, 21.8; **HRMS (ESI)** calcd for $C_{37}H_{26}N_2O_2$ $[M+H]^+$: 531.2067, Found: 531.2069; **HPLC condition:** The enantiomeric excess was determined by Daicel Chiralcel IA, Hexanes/IPA = 50/50, 1.2 mL/min, λ = 254 nm, t (minor) = 22.303 min, t (major) = 112.457 min.

<Chromatogram>

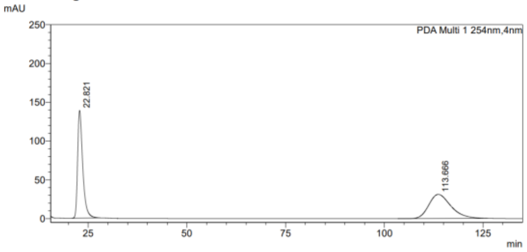

<Peak Table>

PDA Ch1 254nm

Peak#	Ret. Time	Area	Height	Area%
1	22.821	12495766	138912	50.754
2	113.666	12124647	31128	49.246
Total		24620413	170040	100.000

<Chromatogram>

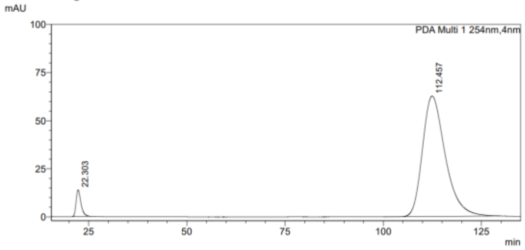

<Peak Table>

PDA Ch1 254nm

Peak#	Ret. Time	Area	Height	Area%
1	22.303	1198679	13837	4.535
2	112.457	25235917	62538	95.465
Total		26434596	76375	100.000

Reaction procedure and characterization of **3g-5**

To a 10 mL Schlenk tube was added **1g-5** (40.2 mg, 0.10 mmol), $Ni(OTf)_2$ (3.6 mg, 10 mol%), (*S*)-**L6** (6.2 mg, 20 mol%), $EtCO_2Ag$ (36.0 mg, 0.20 mmol, 2.0 eq), **P7** (8.5 mg, 20 mol%) and **2a** (28.8 mg, 0.20 mmol, 2.0 eq), followed by addition of anhydrous DME (0.5 mL). Then the mixture was stirred at 100 °C for 24 h. After cooling to room temperature, the reaction system was quenched with aq. $NaHCO_3$ (10 mL) and extracted with CH_2Cl_2 (3×15 mL). The combined organic layer was dried over anhydrous Na_2SO_4, filtered, and concentrated in vacuo. After concentration, the crude product was purified by preparative TLC (PE/acetone = 2/1, v/v, R_f = 0.53) to afford **3g-5** (39.7 mg, 73% yield, 92% ee) as a light-yellow foam.

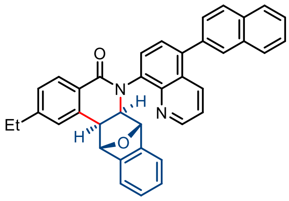

3g-5: $[\alpha]_D^{20}$ = -40.0 (c = 0.97, $CHCl_3$, 92% ee); **^{1}H NMR (400 MHz, $CDCl_3$)** δ 8.80 (brs, 1H), 8.34 (dd, *J* = 8.6, 1.7 Hz, 1H), 8.22 (d, *J* = 8.0 Hz, 1H), 8.12 – 7.90 (m, 5H), 7.79 (brs, 1H), 7.68 (dd, *J* = 8.4, 1.7 Hz, 1H), 7.63 – 7.54 (m, 2H), 7.46 (d, *J* = 7.3 Hz, 1H), 7.36 – 7.34 (m, 2H), 7.29 – 7.22 (m, 2H), 7.12 (t, *J* = 7.4 Hz, 1H), 7.06 (d, *J* = 7.2 Hz, 1H), 5.62 (s, 1H), 5.50 (s, 1H), 4.82 (brs, 1H), 3.71 (d, *J* = 8.6 Hz, 1H), 2.81 (q, *J* = 7.6 Hz, 2H), 1.36 (t, *J* = 7.6 Hz, 3H); **^{13}C NMR (101 MHz, $CDCl_3$)** δ 163.4, 150.4, 149.3, 145.9, 144.0, 142.6, 141.0, 137.7, 137.6, 136.6, 135.1, 133.4, 132.8, 131.9, 129.1, 129.1, 128.5, 128.2, 128.2, 128.1, 127.9, 127.6, 127.5, 127.3, 127.1, 127.0, 126.7, 126.5, 124.9, 121.6, 120.5, 119.3, 88.8, 84.8, 63.5, 43.1, 29.1, 15.6; **HRMS (ESI)** calcd for $C_{38}H_{28}N_2O_2$ $[M+H]^+$: 545.2224, Found: 545.2226; **HPLC condition:** The enantiomeric excess was determined by Daicel Chiralcel IA, Hexanes/IPA = 50/50, 1.7 mL/min, λ = 254 nm, t (minor) = 20.717 min, t (major) = 112.676 min.

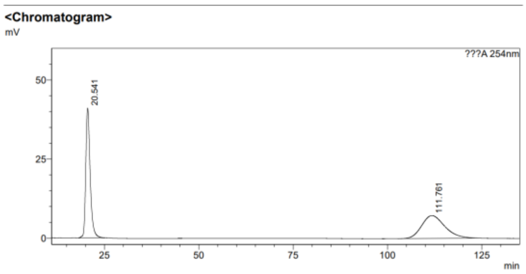

<Peak Table>
???A 254nm

Peak#	Ret. Time	Area	Height	Area%
1	20.541	3338050	41055	51.999
2	111.761	3081450	7214	48.001
Total		6419500	48270	100.000

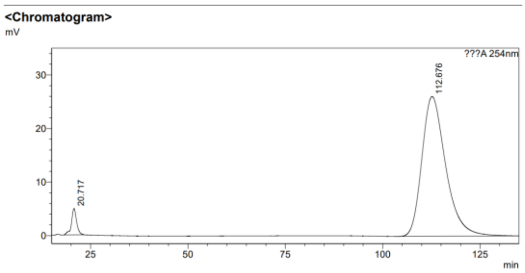

<Peak Table>
???A 254nm

Peak#	Ret. Time	Area	Height	Area%
1	20.717	449464	4919	3.894
2	112.676	11093703	26090	96.106
Total		11543167	31009	100.000

Reaction procedure and characterization of **3g-6**

To a 10 mL Schlenk tube was added **1g-6** (41.6 mg, 0.10 mmol), $Ni(OTf)_2$ (3.6 mg, 10 mol%), (*S*)-**L6** (6.2 mg, 20 mol%), $EtCO_2Ag$ (36.0 mg, 0.20 mmol, 2.0 eq), **P7** (8.5 mg, 20 mol%) and **2a** (28.8 mg, 0.20 mmol, 2.0 eq), followed by addition of anhydrous DME (0.5 mL). Then the mixture was stirred at 100 °C for 24 h. After cooling to room temperature, the reaction system was quenched with aq. $NaHCO_3$ (10 mL) and extracted with CH_2Cl_2 (3×15 mL). The combined organic layer was dried over anhydrous Na_2SO_4, filtered, and concentrated in vacuo. After concentration, the crude product was purified by preparative TLC (PE/acetone = 2/1, v/v, R_f = 0.63) to afford **3g-6** (38.0 mg, 68% yield, 93% ee) as a light-yellow foam.

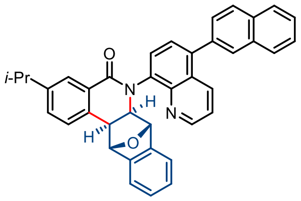

3g-6: $[\alpha]_D^{20}$ = -69.6 (c = 0.93, $CHCl_3$, 93% ee); **^{1}H NMR (400 MHz, $CDCl_3$)** δ 8.80 (brs, 1H), 8.35 (dd, *J* = 8.6, 1.7 Hz, 1H), 8.20 (d, *J* = 1.7 Hz, 1H), 8.10 – 7.92 (m, 5H), 7.80 (brs, 1H), 7.68 (dd, *J* = 8.4, 1.7 Hz, 1H), 7.63 – 7.57 (m, 2H), 7.53 – 7.46 (m, 2H), 7.44 (d, *J* = 7.3 Hz, 1H), 7.35 (brs, 1H), 7.23 (t, *J* = 7.4 Hz, 1H), 7.11 (t, *J* = 7.3 Hz, 1H), 7.05 (d, *J* = 7.2 Hz, 1H), 5.62 (s, 1H), 5.49 (d, *J* = 1.9 Hz, 1H), 4.81 (brs, 1H), 3.72 (d, *J* = 8.6 Hz, 1H), 3.07 – 2.97 (m, 1H), 1.32 – 1.30 (m, 6H); **^{13}C NMR (101 MHz, $CDCl_3$)** δ 163.6, 150.5, 147.9, 145.8, 144.0, 142.5, 141.0, 137.7, 136.6, 135.1, 133.4, 132.8, 131.9, 131.0, 129.1, 128.5, 128.2, 128.2, 127.9, 127.6, 127.5, 127.1, 126.8, 126.7, 126.6, 121.6, 120.5, 119.3, 88.8, 84.7, 63.5, 42.8, 34.0, 24.0, 23.9; **HRMS (ESI)** calcd for $C_{39}H_{30}N_2O_2$ $[M+H]^+$: 559.2380, Found: 559.2384; **HPLC condition:** The enantiomeric excess was determined by Daicel Chiralcel IA, Hexanes/IPA = 50/50, 1.2 mL/min, λ = 254 nm, t (major) = 36.399 min, t (minor) = 51.115 min.

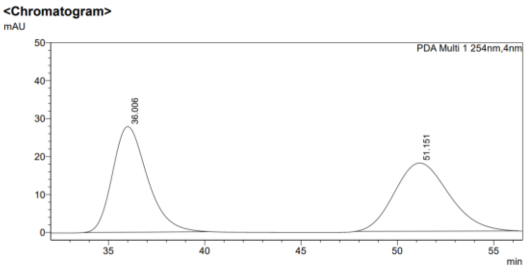

<Peak Table>
PDA Ch1 254nm

Peak#	Ret. Time	Area	Height	Area%
1	36.006	3500880	27820	49.766
2	51.151	3533842	17973	50.234
Total		7034723	45794	100.000

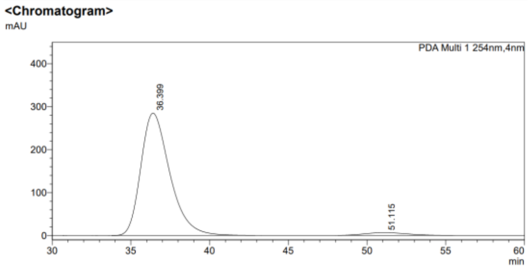

<Peak Table>
PDA Ch1 254nm

Peak#	Ret. Time	Area	Height	Area%
1	36.399	36535828	284972	96.728
2	51.115	1236071	6561	3.272
Total		37771899	291533	100.000

Reaction procedure and characterization of **3g-7**

To a 10 mL Schlenk tube was added **1g-7** (41.6 mg, 0.10 mmol), Ni(OTf)$_2$ (3.6 mg, 10 mol%), (*S*)-**L6** (6.2 mg, 20 mol%), EtCO$_2$Ag (36.0 mg, 0.20 mmol, 2.0 eq), **P7** (8.5 mg, 20 mol%) and **2a** (28.8 mg, 0.20 mmol, 2.0 eq), followed by addition of anhydrous DME (0.5 mL). Then the mixture was stirred at 100 °C for 24 h. After cooling to room temperature, the reaction system was quenched with aq. NaHCO$_3$ (10 mL) and extracted with CH$_2$Cl$_2$ (3×15 mL). The combined organic layer was dried over anhydrous Na$_2$SO$_4$, filtered, and concentrated in vacuo. After concentration, the crude product was purified by preparative TLC (PE/acetone = 2/1, v/v, R_f = 0.55) to afford **3g-7** (41.0 mg, 73% yield, 94% ee) as a light-yellow oil.

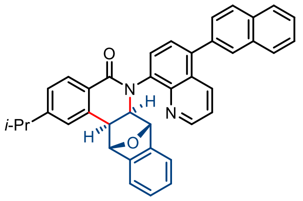

3g-7: $[\alpha]_D^{20}$ = -40.4 (c = 0.95, CHCl$_3$, 94% ee); **^{1}H NMR (400 MHz, CDCl$_3$)** δ 8.80 (brs, 1H), 8.34 (dd, *J* = 8.6, 1.7 Hz, 1H), 8.23 (d, *J* = 8.1 Hz, 1H), 8.10 – 7.92 (m, 5H), 7.79 (brs, 1H), 7.68 (dd, *J* = 8.4, 1.7 Hz, 1H), 7.62 – 7.56 (m, 2H), 7.48 (d, *J* = 7.2 Hz, 1H), 7.37 – 7.34 (m, 2H), 7.30 (dd, *J* = 8.1, 1.7 Hz, 1H), 7.28 – 7.22 (m, 1H), 7.12 (t, *J* = 7.3 Hz, 1H), 7.06 (d, *J* = 7.3 Hz, 1H), 5.62 (s, 1H), 5.50 (s, 1H), 4.83 (brs, 1H), 3.72 (d, *J* = 8.6 Hz, 1H), 3.12 – 3.02 (m, 1H), 1.38 (d, *J* = 6.9 Hz, 6H); **^{13}C NMR (101 MHz, CDCl$_3$)** δ 163.3, 153.9, 150.4, 145.8, 144.1, 142.6, 141.0, 137.7, 137.6, 136.6, 135.1, 133.4, 132.8, 132.0, 129.2, 129.1, 128.5, 128.2, 128.2, 128.1, 127.9, 127.6, 127.5, 127.1, 126.7, 126.5, 126.0, 125.6, 125.0, 121.5, 120.5, 119.3, 88.9, 84.8, 63.5, 43.2, 34.5, 24.1, 23.9; **HRMS (ESI)** calcd for $C_{39}H_{30}N_2O_2$ $[M+H]^+$: 559.2380, Found: 559.2382; **HPLC condition:** The enantiomeric excess was determined by Daicel Chiralcel IA, Hexanes/IPA = 50/50, 1.7 mL/min, λ = 254 nm, t (minor) = 25.605 min, t (major) = 92.577 min.

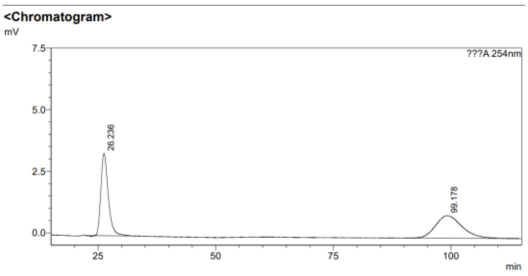

<Peak Table>

???A 254nm

Peak#	Ret. Time	Area	Height	Area%
1	26.236	362069	3338	50.086
2	99.178	360831	916	49.914
Total		722900	4254	100.000

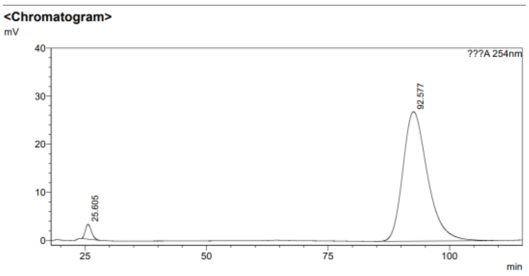

<Peak Table>

???A 254nm

Peak#	Ret. Time	Area	Height	Area%
1	25.605	271429	3085	2.778
2	92.577	9497503	26928	97.222
Total		9768932	30013	100.000

Reaction procedure and characterization of **3g-8**

To a 10 mL Schlenk tube was added **1g-8** (43.0 mg, 0.10 mmol), Ni(OTf)$_2$ (3.6 mg, 10 mol%), (*S*)-**L6** (6.2 mg, 20 mol%), EtCO$_2$Ag (36.0 mg, 0.20 mmol, 2.0 eq), **P7** (8.5 mg, 20 mol%) and **2a** (28.8 mg, 0.20 mmol, 2.0 eq), followed by addition of anhydrous DME (0.5 mL). Then the mixture was stirred at 100 °C for 24 h. After cooling to room temperature, the reaction system was quenched with aq. NaHCO$_3$ (10 mL) and extracted with CH$_2$Cl$_2$ (3×15 mL). The combined organic layer was dried over anhydrous Na$_2$SO$_4$, filtered, and concentrated in vacuo. After concentration, the crude product was purified by preparative TLC (PE/acetone = 2/1, v/v, R$_f$ = 0.59) to afford **3g-8** (44.3 mg, 77% yield, 93% ee) as a light-yellow oil.

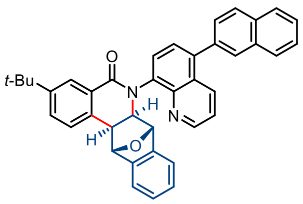

3g-8: $[\alpha]_D^{20}$ = -70.7 (c = 1.08, CHCl$_3$, 93% ee); **^{1}H NMR (400 MHz, CDCl$_3$)** δ 8.80 (brs, 1H), 8.40 – 8.31 (m, 2H), 8.11 – 7.91 (m, 5H), 7.79 (brs, 1H), 7.70 – 7.66 (m, 2H), 7.63 – 7.56 (m, 2H), 7.49 (d, *J* = 8.1 Hz, 1H), 7.45 (d, *J* = 7.3 Hz, 1H), 7.35 (brs, 1H), 7.24 (t, *J* = 7.3 Hz, 1H), 7.11 (t, *J* = 7.4 Hz, 1H), 7.06 (d, *J* = 7.2 Hz, 1H), 5.62 (s, 1H), 5.49 (s, 1H), 4.82 (brs, 1H), 3.72 (d, *J* = 8.6 Hz, 1H), 1.39 (s, 9H); **^{13}C NMR (101 MHz, CDCl$_3$)** δ 163.7, 150.5, 150.2, 145.8, 144.0, 142.5, 141.0, 137.7, 136.6, 135.1, 134.7, 133.4, 132.8, 131.9, 129.9, 129.1, 128.5, 128.2, 128.2, 127.9, 127.9, 127.6, 127.5, 127.1, 126.7, 126.6, 126.5, 125.7, 121.6, 120.5, 119.3, 88.8, 84.7, 63.4, 42.7, 34.8, 31.3; **HRMS (ESI)** calcd for $C_{40}H_{32}N_2O_2$ $[M+H]^+$: 573.2537, Found: 573.2540; **HPLC condition:** The enantiomeric excess was determined by Daicel Chiralcel IA, Hexanes/IPA = 50/50, 1.5 mL/min, λ = 254 nm, t (major) = 12.505 min, t (minor) = 32.034 min.

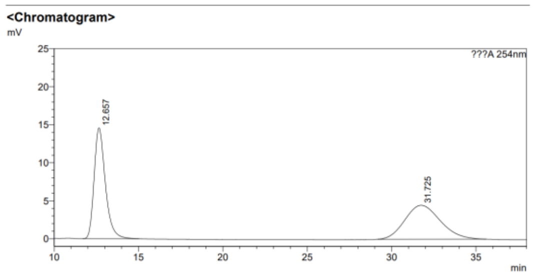

<Peak Table>
???A 254nm

Peak#	Ret. Time	Area	Height	Area%
1	12.657	673768	14574	50.753
2	31.725	653787	4463	49.247
Total		1327554	19038	100.000

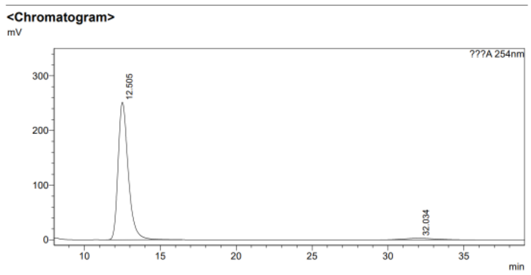

<Peak Table>
???A 254nm

Peak#	Ret. Time	Area	Height	Area%
1	12.505	11653556	251478	96.550
2	32.034	416463	2815	3.450
Total		12070019	254292	100.000

Reaction procedure and characterization of **3g-9**

To a 10 mL Schlenk tube was added **1g-9** (43.0 mg, 0.10 mmol), $Ni(OTf)_2$ (3.6 mg, 10 mol%), (*S*)-**L6** (6.2 mg, 20 mol%), $EtCO_2Ag$ (36.0 mg, 0.20 mmol, 2.0 eq), **P7** (8.5 mg, 20 mol%) and **2a** (28.8 mg, 0.20 mmol, 2.0 eq), followed by addition of anhydrous DME (0.5 mL). Then the mixture was stirred at 100 °C for 24 h. After cooling to room temperature, the reaction system was quenched with aq. $NaHCO_3$ (10 mL) and extracted with CH_2Cl_2 (3×15 mL). The combined organic layer was dried over anhydrous Na_2SO_4, filtered, and concentrated in vacuo. After concentration, the crude product was purified by preparative TLC (PE/EtOAc = 2/1, v/v, R_f = 0.55) to afford **3g-9** (39.9 mg, 70% yield, 94% ee) as a light-yellow oil.

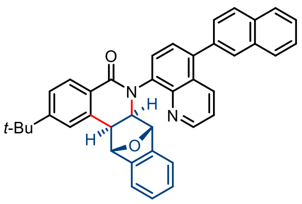

3g-9: $[\alpha]_D^{20}$ = -34.3 (c = 1.03, $CHCl_3$, 94% ee); **^{1}H NMR (400 MHz, $CDCl_3$)** δ 8.80 (brs, 1H), 8.34 (dd, *J* = 8.7, 1.7 Hz, 1H), 8.24 (d, *J* = 8.2 Hz, 1H), 8.11 – 7.91 (m, 5H), 7.78 (brs, 1H), 7.68 (dd, *J* = 8.4, 1.7 Hz, 1H), 7.62 – 7.55 (m, 2H), 7.52 – 7.42 (m, 3H), 7.35 (brs, 1H), 7.25 (t, *J* = 7.4 Hz, 1H), 7.12 (t, *J* = 7.4 Hz, 1H), 7.06 (d, *J* = 7.2 Hz, 1H), 5.62 (s, 1H), 5.50 (s, 1H), 4.84 (brs, 1H), 3.73 (d, *J* = 8.6 Hz, 1H), 1.46 (s, 9H); **^{13}C NMR (101 MHz, $CDCl_3$)** δ 163.2, 156.1, 150.5, 145.9, 144.1, 142.7, 141.0, 137.6, 137.4, 136.6, 135.1, 133.4, 132.9, 132.0, 129.1, 128.9, 128.6, 128.2, 128.2, 127.9, 127.6, 127.5, 127.2, 126.7, 126.6, 124.7, 124.7, 124.7, 121.6, 120.5, 119.4, 88.9, 84.8, 63.6, 43.4, 35.2, 31.4; **HRMS (ESI)** calcd for $C_{40}H_{32}N_2O_2$ $[M+H]^+$: 573.2537, Found: 573.2540; **HPLC condition:** The enantiomeric excess was determined by Daicel Chiralcel IA, Hexanes/IPA = 50/50, 1.2 mL/min, λ = 254 nm, t (major) = 42.413 min, t (minor) = 53.566 min.

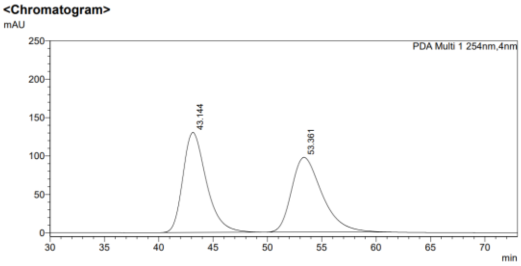

<Peak Table>

PDA Ch1 254nm

Peak#	Ret. Time	Area	Height	Area%
1	43.144	19566902	130473	50.625
2	53.361	19083774	97468	49.375
Total		38650676	227941	100.000

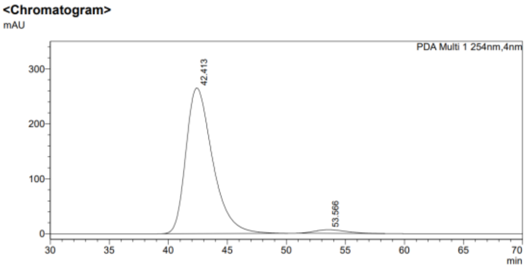

<Peak Table>

PDA Ch1 254nm

Peak#	Ret. Time	Area	Height	Area%
1	42.413	41712434	264905	97.085
2	53.566	1252579	6731	2.915
Total		42965013	271636	100.000

Reaction procedure and characterization of **3g-10**

To a 10 mL Schlenk tube was added **1g-10** (40.4 mg, 0.10 mmol), Ni(OTf)$_2$ (3.6 mg, 10 mol%), (*S*)-**L6** (6.2 mg, 20 mol%), $EtCO_2Ag$ (36.0 mg, 0.20 mmol, 2.0 eq), **P7** (8.5 mg, 20 mol%) and **2a** (28.8 mg, 0.20 mmol, 2.0 eq), followed by addition of anhydrous DME (0.5 mL). Then the mixture was stirred at 100 °C for 24 h. After cooling to room temperature, the reaction system was quenched with aq. $NaHCO_3$ (10 mL) and extracted with CH_2Cl_2 (3×15 mL). The combined organic layer was dried over anhydrous Na_2SO_4, filtered, and concentrated in vacuo. After concentration, the crude product was purified by preparative TLC (PE/EtOAc = 2/1, v/v, R_f = 0.14) to afford **3g-10** (41.3 mg, 76% yield, 92% ee) as a light-yellow oil.

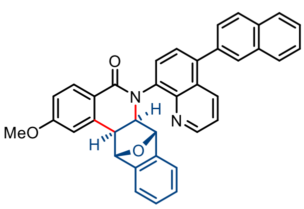

3g-10: $[\alpha]_D^{20}$ = -30.7 (c = 0.96, $CHCl_3$, 92% ee); **^{1}H NMR (400 MHz, $CDCl_3$)** δ 8.80 (brs, 1H), 8.34 (dd, *J* = 8.5, 1.7 Hz, 1H), 8.26 (d, *J* = 8.7 Hz, 1H), 8.11 – 7.90 (m, 5H), 7.79 (brs, 1H), 7.67 (dd, *J* = 8.4, 1.7 Hz, 1H), 7.63 – 7.55 (m, 2H), 7.44 (d, *J* = 7.3 Hz, 1H), 7.34 (brs, 1H), 7.23 (t, *J* = 7.7 Hz, 1H), 7.11 (t, *J* = 7.4 Hz, 1H), 7.05 (d, *J* = 7.2 Hz, 1H), 7.00 (d, *J* = 2.4 Hz, 1H), 6.95 (dd, *J* = 8.7, 2.5 Hz, 1H), 5.61 (s, 1H), 5.50 (s, 1H), 4.81 (brs, 1H), 3.95 (s, 3H), 3.68 (d, *J* = 8.5 Hz, 1H); **^{13}C NMR (101 MHz, $CDCl_3$)** δ 163.0, 150.4, 145.8, 144.1, 142.5, 140.9, 139.7, 137.6, 136.6, 135.1, 133.4, 132.8, 132.0, 131.3, 129.1, 128.5, 128.2, 128.2, 128.2, 127.9, 127.6, 127.5, 127.2, 126.7, 126.5, 121.5, 120.5, 120.3, 119.2, 112.9, 112.8, 88.8, 84.8, 63.5, 55.6, 43.3; **HRMS (ESI)** calcd for $C_{37}H_{26}N_2O_3$ $[M+H]^+$: 547.2016, Found: 547.2017; **HPLC condition:** The enantiomeric excess was determined by Daicel Chiralcel IA, Hexanes/IPA = 50/50, 1.2 mL/min, λ = 254 nm, t (minor) = 33.288 min, t (major) = 185.188 min.

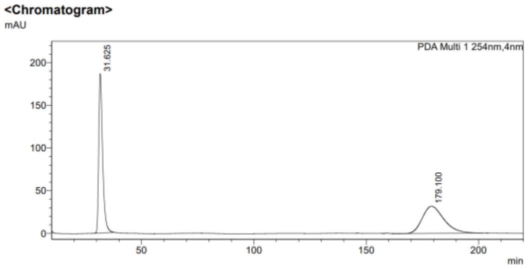

<Peak Table>

PDA Ch1 254nm

Peak#	Ret. Time	Area	Height	Area%
1	31.625	22437440	186666	51.238
2	179.100	21352926	31778	48.762
Total		43790366	218444	100.000

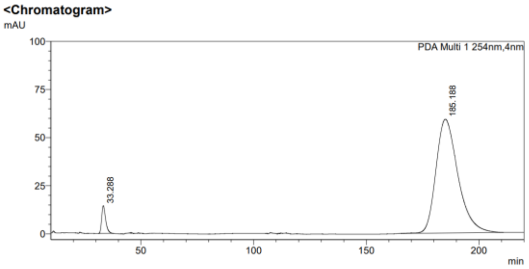

<Peak Table>

PDA Ch1 254nm

Peak#	Ret. Time	Area	Height	Area%
1	33.288	1760827	14396	4.212
2	185.188	40041406	59082	95.788
Total		41802233	73478	100.000

Reaction procedure and characterization of **3g-11**

To a 10 mL Schlenk tube was added **1g-11** (43.4 mg, 0.10 mmol), $Ni(OTf)_2$ (3.6 mg, 10 mol%), (*S*)-**L6** (6.2 mg, 20 mol%), $EtCO_2Ag$ (36.0 mg, 0.20 mmol, 2.0 eq), **P7** (8.5 mg, 20 mol%) and **2a** (28.8 mg, 0.20 mmol, 2.0 eq), followed by addition of anhydrous DME (0.5 mL). Then the mixture was stirred at 100 °C for 24 h. After cooling to room temperature, the reaction system was quenched with aq. $NaHCO_3$ (10 mL) and extracted with CH_2Cl_2 (3×15 mL). The combined organic layer was dried over anhydrous Na_2SO_4, filtered, and concentrated in vacuo. After concentration, the crude product was purified by preparative TLC (PE/EtOAc = 1/1, v/v, R_f = 0.26) to afford **3g-11** (39.9 mg, 69% yield, 85% ee) as a light-yellow foam.

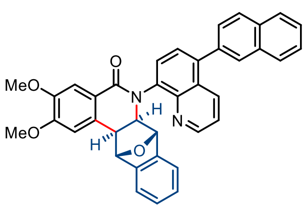

3g-11: $[\alpha]_D^{20}$ = -38.8 (c = 0.85, $CHCl_3$, 85% ee); **^{1}H NMR (400 MHz, $CDCl_3$)** δ 8.78 (brs, 1H), 8.33 (dd, *J* = 8.5, 1.7 Hz, 1H), 8.07 – 7.90 (m, 5H), 7.78 – 7.76 (m, 2H), 7.66 (dd, *J* = 8.4, 1.6 Hz, 1H), 7.61 – 7.53 (m, 2H), 7.44 (d, *J* = 7.2 Hz, 1H), 7.33 (brs, 1H), 7.22 (t, *J* = 7.4 Hz, 1H), 7.10 (t, *J* = 7.4 Hz, 1H), 7.04 (d, *J* = 7.2 Hz, 1H), 6.91 (s, 1H), 5.60 (s, 1H), 5.47 (s, 1H), 4.77 (brs, 1H), 4.05 (s, 3H), 3.93 (s, 3H), 3.65 (d, *J* = 8.7 Hz, 1H); **^{13}C NMR (101 MHz, $CDCl_3$)** δ 163.3, 152.9, 150.5, 148.3, 145.7, 144.1, 142.5, 141.0, 137.7, 136.5, 135.1, 133.4, 132.8, 131.9, 131.1, 129.1, 128.5, 128.2, 128.1, 127.9, 127.6, 127.5, 127.2, 126.7, 126.5, 121.5, 120.5, 120.2, 119.3, 110.6, 109.6, 88.4, 84.8, 63.6, 56.4, 56.1, 43.0; **HRMS (ESI)** calcd for $C_{38}H_{28}N_2O_4$ $[M+H]^+$: 577.2122, Found: 577.2125; **HPLC condition:** The enantiomeric excess was determined by Daicel Chiralcel IA, Hexanes/IPA = 50/50, 1.5 mL/min, λ = 254 nm, t (minor) = 11.075 min, t (major) = 63.167 min.

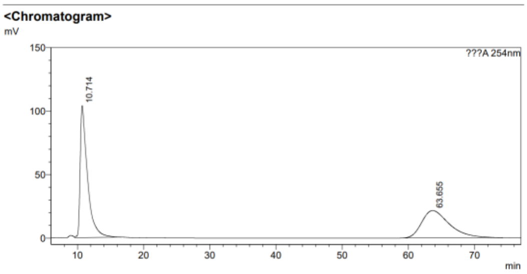

<Peak Table>
???A 254nm

Peak#	Ret. Time	Area	Height	Area%
1	10.714	7905726	103828	55.589
2	63.655	6316040	21587	44.411
Total		14221766	125415	100.000

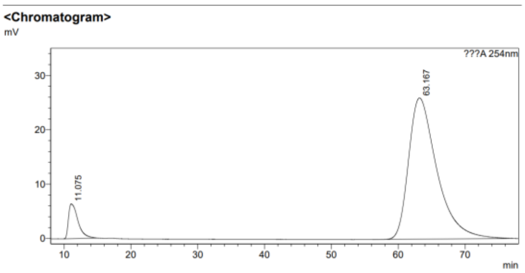

<Peak Table>
???A 254nm

Peak#	Ret. Time	Area	Height	Area%
1	11.075	630565	6387	7.604
2	63.167	7661623	25976	92.396
Total		8292187	32363	100.000

Reaction procedure and characterization of **3g-12**

To a 10 mL Schlenk tube was added **1g-12** (42.0 mg, 0.10 mmol), $Ni(OTf)_2$ (3.6 mg, 10 mol%), (*S*)-**L6** (6.2 mg, 20 mol%), $EtCO_2Ag$ (36.0 mg, 0.20 mmol, 2.0 eq), **P7** (8.5 mg, 20 mol%) and **2a** (28.8 mg, 0.20 mmol, 2.0 eq), followed by addition of anhydrous DME (0.5 mL). Then the mixture was stirred at 100 °C for 24 h. After cooling to room temperature, the reaction system was quenched with aq. $NaHCO_3$ (10 mL) and extracted with CH_2Cl_2 (3×15 mL). The combined organic layer was dried over anhydrous Na_2SO_4, filtered, and concentrated in vacuo. After concentration, the crude product was purified by preparative TLC (PE/acetone = 2/1, v/v, R_f = 0.47) to afford **3g-12** (31.8 mg, 57% yield, 87% ee) as a light-yellow oil..

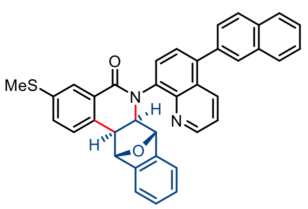

3g-12: $[\alpha]_D^{20}$ = -78.9 (c = 0.94, $CHCl_3$, 87% ee); **^{1}H NMR (400 MHz, $CDCl_3$)** δ 8.80 (brs, 1H), 8.35 (dd, *J* = 8.5, 1.7 Hz, 1H), 8.17 (d, *J* = 2.1 Hz, 1H), 8.09 – 7.91 (m, 5H), 7.79 (brs, 1H), 7.68 (dd, *J* = 8.4, 1.6 Hz, 1H), 7.63 – 7.57 (m, 2H), 7.53 (dd, *J* = 8.1, 2.2 Hz, 1H), 7.48 – 7.39 (m, 2H), 7.36 (brs, 1H), 7.23 (t, *J* = 7.4 Hz, 1H), 7.11 (t, *J* = 7.4 Hz, 1H), 7.05 (d, *J* = 7.2 Hz, 1H), 5.61 (s, 1H), 5.45 (s, 1H), 4.79 (brs, 1H), 3.69 (d, *J* = 8.6 Hz, 1H), 2.53 (s, 3H); **^{13}C NMR (101 MHz, $CDCl_3$)** δ 163.0, 150.5, 145.7, 144.0, 142.4, 141.1, 137.9, 137.4, 136.5, 135.1, 134.3, 133.4, 132.8, 131.8, 131.1, 129.1, 128.6, 128.2, 128.1, 127.9, 127.7, 127.6, 127.5, 127.2, 126.7, 126.6, 125.8, 121.6, 120.5, 119.3, 88.7, 84.7, 63.5, 42.8, 15.8; **HRMS (ESI)** calcd for $C_{37}H_{26}N_2O_2S$ $[M+H]^+$: 563.1788, Found: 563.1792; **HPLC condition:** The enantiomeric excess was determined by Daicel Chiralcel IA, Hexanes/IPA = 50/50, 1.6 mL/min, λ = 254 nm, t (minor) = 85.766 min, t (major) = 140.318 min.

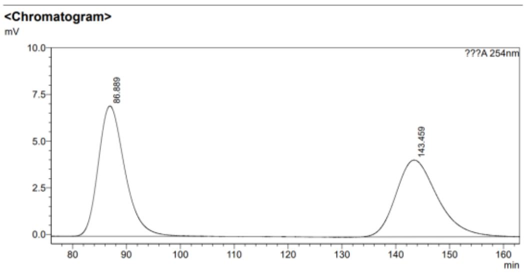

<Peak Table>
???A 254nm

Peak#	Ret. Time	Area	Height	Area%
1	86.889	2392693	6980	51.974
2	143.459	2210944	4118	48.026
Total		4603637	11097	100.000

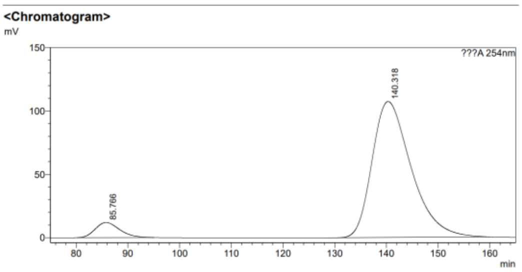

<Peak Table>
???A 254nm

Peak#	Ret. Time	Area	Height	Area%
1	85.766	3907534	11917	6.483
2	140.318	56362445	107286	93.517
Total		60269979	119203	100.000

Reaction procedure and characterization of **3g-13**

To a 10 mL Schlenk tube was added **1g-13** (42.0 mg, 0.10 mmol), Ni(OTf)$_2$ (3.6 mg, 10 mol%), (*S*)-**L6** (6.2 mg, 20 mol%), EtCO$_2$Ag (36.0 mg, 0.20 mmol, 2.0 eq), **P7** (8.5 mg, 20 mol%) and **2a** (28.8 mg, 0.20 mmol, 2.0 eq), followed by addition of anhydrous DME (0.5 mL). Then the mixture was stirred at 100 °C for 24 h. After cooling to room temperature, the reaction system was quenched with aq. NaHCO$_3$ (10 mL) and extracted with CH$_2$Cl$_2$ (3×15 mL). The combined organic layer was dried over anhydrous Na$_2$SO$_4$, filtered, and concentrated in vacuo. After concentration, the crude product was purified by preparative TLC (PE/acetone = 2/1, v/v, R$_f$ = 0.42) to afford **3g-13** (40.1 mg, 71% yield, 93% ee) as a light-yellow foam.

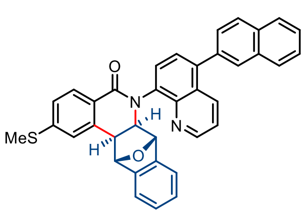

3g-13: $[\alpha]_D^{20}$ = -9.3 (c = 0.96, CHCl$_3$, 93% ee); **^{1}H NMR (400 MHz, CDCl$_3$)** δ 8.79 (brs, 1H), 8.33 (dd, *J* = 8.5, 1.7 Hz, 1H), 8.21 (d, *J* = 8.3 Hz, 1H), 8.07 – 7.90 (m, 5H), 7.78 (brs, 1H), 7.66 (dd, *J* = 8.4, 1.6 Hz, 1H), 7.62 – 7.54 (m, 2H), 7.44 (d, *J* = 7.3 Hz, 1H), 7.36 – 7.32 (m, 2H), 7.26 – 7.21 (m, 2H), 7.11 (t, *J* = 7.4 Hz, 1H), 7.05 (d, *J* = 7.2 Hz, 1H), 5.60 (s, 1H), 5.48 (s, 1H), 4.80 (brs, 1H), 3.66 (d, *J* = 8.5 Hz, 1H), 2.60 (s, 3H); **^{13}C NMR (101 MHz, CDCl$_3$)** δ 163.1, 150.5, 145.7, 144.5, 144.0, 142.5, 141.0, 138.2, 137.4, 136.5, 135.1, 133.4, 132.8, 131.9, 129.5, 129.1, 128.5, 128.2, 128.1, 127.9, 127.7, 127.5, 127.2, 126.7, 126.6, 124.9, 124.1, 123.9, 121.6, 120.5, 119.3, 88.7, 84.7, 63.5, 43.0, 15.2; **HRMS (ESI)** calcd for $C_{37}H_{26}N_2O_2S$ $[M+H]^+$: 563.1788, Found: 563.1792; **HPLC condition:** The enantiomeric excess was determined by Daicel Chiralcel IA, Hexanes/IPA = 50/50, 1.7 mL/min, λ = 254 nm, t (minor) = 32.449 min, t (major) = 177.042 min.

<Chromatogram>

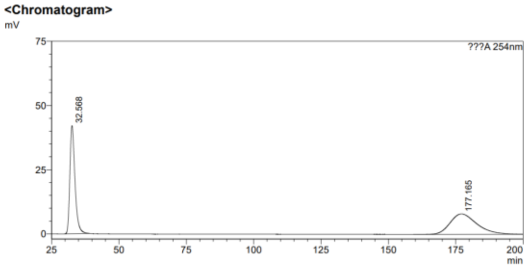

<Peak Table>

???A 254nm

Peak#	Ret. Time	Area	Height	Area%
1	32.568	5529843	42118	50.144
2	177.165	5498023	7944	49.856
Total		11027865	50062	100.000

<Chromatogram>

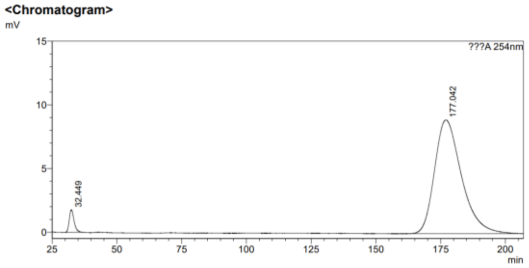

<Peak Table>

???A 254nm

Peak#	Ret. Time	Area	Height	Area%
1	32.449	230288	1787	3.444
2	177.042	6457024	8931	96.556
Total		6687311	10718	100.000

Reaction procedure and characterization of **3g-14**

To a 10 mL Schlenk tube was added **1g-14** (44.2 mg, 0.10 mmol), Ni(OTf)$_2$ (3.6 mg, 10 mol%), (*S*)-**L6** (6.2 mg, 20 mol%), EtCO$_2$Ag (36.0 mg, 0.20 mmol, 2.0 eq), **P7** (8.5 mg, 20 mol%) and **2a** (28.8 mg, 0.20 mmol, 2.0 eq), followed by addition of anhydrous DME (0.5 mL). Then the mixture was stirred at 100 °C for 24 h. After cooling to room temperature, the reaction system was quenched with aq. NaHCO$_3$ (10 mL) and extracted with CH$_2$Cl$_2$ (3×15 mL). The combined organic layer was dried over anhydrous Na$_2$SO$_4$, filtered, and concentrated in vacuo. After concentration, the crude product was purified by preparative TLC (PE/EtOAc = 2/1, v/v, R$_f$ = 0.66) to afford **3g-14** (31.8 mg, 54% yield, 89% ee) as a light-yellow oil.

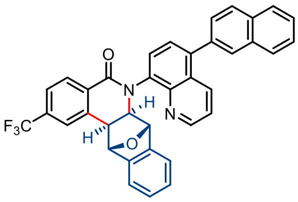

3g-14: $[\alpha]_D^{20}$ = -38.4 (c = 1.01, CHCl$_3$, 89% ee); **^{1}H NMR (400 MHz, CDCl$_3$)** δ 8.80 (brs, 1H), 8.43 (d, *J* = 8.1 Hz, 1H), 8.36 (dd, *J* = 8.6, 1.7 Hz, 1H), 8.13 – 7.91 (m, 5H), 7.82 – 7.80 (m, 2H), 7.69 – 7.66 (m, 2H), 7.62 – 7.58 (m, 2H), 7.49 (d, *J* = 7.3 Hz, 1H), 7.38 (brs, 1H), 7.27 (t, *J* = 7.4 Hz, 1H), 7.14 (t, *J* = 7.4 Hz, 1H), 7.08 (d, *J* = 7.2 Hz, 1H), 5.64 (s, 1H), 5.49 (s, 1H), 4.84 (brs, 1H), 3.78 (d, *J* = 8.5 Hz, 1H); **^{19}F NMR (376 MHz, CDCl$_3$)** δ -62.71; **^{13}C NMR (101 MHz, CDCl$_3$)** δ 162.0, 150.6, 145.3, 143.7, 142.2, 141.4, 138.5, 137.0, 136.4, 135.2, 134.1 (q, *J* = 32.5 Hz), 133.3, 132.8, 131.6, 130.1, 129.8, 129.1, 128.6, 128.3, 128.2, 128.0, 127.9, 127.5, 127.4, 126.7, 126.6, 125.2 (q, *J* = 3.7 Hz), 124.0 (q, *J* = 3.7 Hz), 123.8 (q, *J* = 273.6 Hz), 121.7, 120.6, 119.4, 88.7, 84.7, 63.6, 43.1; **HRMS (ESI)** calcd for $C_{37}H_{23}F_3N_2O_2$ $[M+H]^+$: 585.1784, Found: 585.1787; **HPLC condition:** The enantiomeric excess was determined by Daicel Chiralcel IA, Hexanes/IPA = 50/50, 1.2 mL/min, λ = 254 nm, t (minor) = 11.320 min, t (major) = 39.454 min.

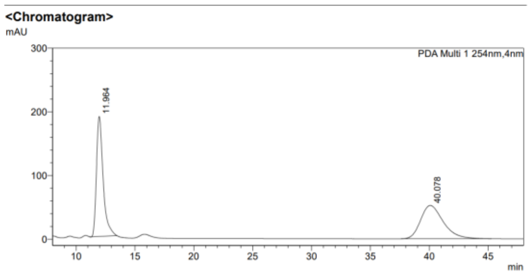

<Peak Table>
PDA Ch1 254nm

Peak#	Ret. Time	Area	Height	Area%
1	11.964	7409194	188514	51.425
2	40.078	6998648	52325	48.575
Total		14407842	240839	100.000

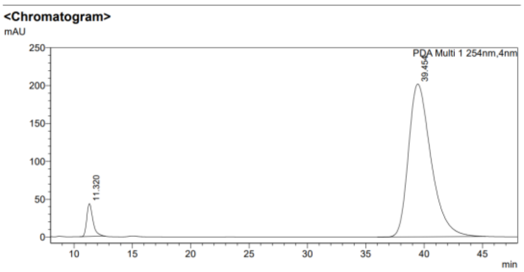

<Peak Table>
PDA Ch1 254nm

Peak#	Ret. Time	Area	Height	Area%
1	11.320	1591624	43213	5.549
2	39.454	27092225	201587	94.451
Total		28683849	244800	100.000

Reaction procedure and characterization of **3g-15**

To a 10 mL Schlenk tube was added **1g-15** (45.8 mg, 0.10 mmol), Ni(OTf)$_2$ (3.6 mg, 10 mol%), (*S*)-**L6** (6.2 mg, 20 mol%), EtCO$_2$Ag (36.0 mg, 0.20 mmol, 2.0 eq), **P7** (8.5 mg, 20 mol%) and **2a** (28.8 mg, 0.20 mmol, 2.0 eq), followed by addition of anhydrous DME (0.5 mL). Then the mixture was stirred at 100 °C for 24 h. After cooling to room temperature, the reaction system was quenched with aq. NaHCO$_3$ (10 mL) and extracted with CH_2Cl_2 (3×15 mL). The combined organic layer was dried over anhydrous Na_2SO_4, filtered, and concentrated in vacuo. After concentration, the crude product was purified by preparative TLC (PE/acetone = 2/1, v/v, R_f = 0.61) to afford **3g-15** (35.3 mg, 59% yield, 91% ee) as a light-yellow oil.

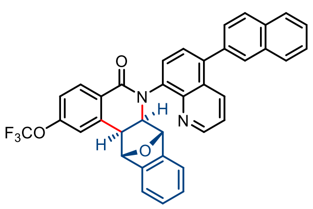

3g-15: $[\alpha]_D^{20}$ = -41.5 (c = 0.92, CHCl$_3$, 91% ee); **^{1}H NMR (400 MHz, CDCl$_3$)** δ 8.81 (brs, 1H), 8.37 – 8.34 (m, 2H), 8.10 – 7.91 (m, 5H), 7.80 (brs, 1H), 7.67 (dd, *J* = 8.4, 1.7 Hz, 1H), 7.62 – 7.58 (m, 2H), 7.46 (d, *J* = 7.3 Hz, 1H), 7.39 – 7.36 (m, 2H), 7.31 – 7.21 (m, 2H), 7.13 (t, *J* = 7.4 Hz, 1H), 7.07 (d, *J* = 7.3 Hz, 1H), 5.63 (s, 1H), 5.48 (s, 1H), 4.83 (brs, 1H), 3.73 (d, *J* = 8.5 Hz, 1H); **^{19}F NMR (376 MHz, CDCl$_3$)** δ -57.36; **^{13}C NMR (101 MHz, CDCl$_3$)** δ 162.2, 152.3 (m), 150.5, 145.3, 143.9, 142.3, 141.3, 140.0, 137.1, 136.4, 135.2, 133.4, 132.8, 131.7, 131.5, 129.1, 128.6, 128.2, 128.2, 128.1, 127.9, 127.8, 127.5, 127.4, 126.7, 126.6, 125.8, 121.7, 120.6, 120.4 (q, *J* = 258.5 Hz), 119.8, 119.5, 119.4, 88.7, 84.7, 63.7, 43.2; **HRMS (ESI)** calcd for $C_{37}H_{23}F_3N_2O_3$ $[M+H]^+$: 601.1734, Found: 601.1736; **HPLC condition:** The enantiomeric excess was determined by Daicel Chiralcel IA, Hexanes/IPA = 50/50, 1.5 mL/min, λ = 254 nm, t (minor) = 10.196 min, t (major) = 74.324 min.

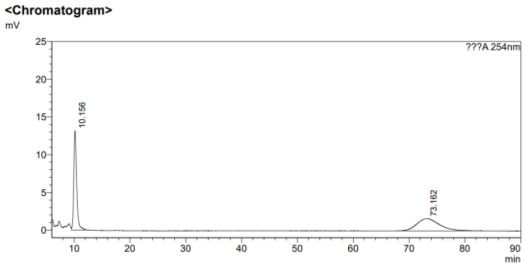

<Peak Table>
???A 254nm

Peak#	Ret. Time	Area	Height	Area%
1	10.156	470520	13140	50.884
2	73.162	454173	1608	49.116
Total		924693	14748	100.000

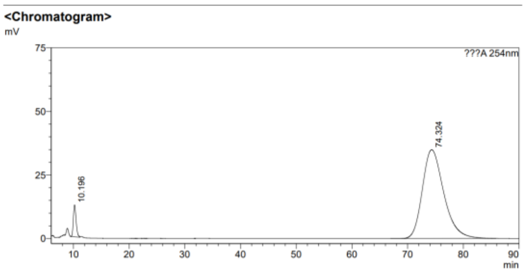

<Peak Table>
???A 254nm

Peak#	Ret. Time	Area	Height	Area%
1	10.196	430343	12511	4.401
2	74.324	9348452	34920	95.599
Total		9778794	47430	100.000

Reaction procedure and characterization of **3g-16**

To a 10 mL Schlenk tube was added **1g-16** (39.2 mg, 0.10 mmol), $Ni(OTf)_2$ (3.6 mg, 10 mol%), (*S*)-**L6** (6.2 mg, 20 mol%), $EtCO_2Ag$ (36.0 mg, 0.20 mmol, 2.0 eq), **P7** (8.5 mg, 20 mol%) and **2a** (28.8 mg, 0.20 mmol, 2.0 eq), followed by addition of anhydrous DME (0.5 mL). Then the mixture was stirred at 100 °C for 24 h. After cooling to room temperature, the reaction system was quenched with aq. $NaHCO_3$ (10 mL) and extracted with CH_2Cl_2 (3×15 mL). The combined organic layer was dried over anhydrous Na_2SO_4, filtered, and concentrated in vacuo. After concentration, the crude product was purified by preparative TLC (PE/EtOAc = 2/1, v/v, R_f = 0.32) to afford **3g-16** (34.9 mg, 65% yield, 90% ee) as a light-yellow foam.

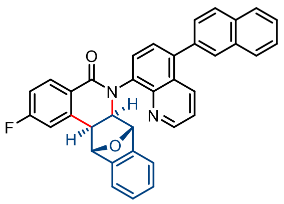

3g-16: $[\alpha]_D^{20}$ = -49.0 (c = 0.89, $CHCl_3$, 90% ee); **<u>^{1}H NMR (400 MHz, CDCl$_3$)</u>** δ 8.79 (brs, 1H), 8.39 – 8.27 (m, 2H), 8.10 – 7.88 (m, 5H), 7.78 (brs, 1H), 7.65 (dd, *J* = 8.4, 1.7 Hz, 1H), 7.62 – 7.51 (m, 2H), 7.42 (d, *J* = 7.3 Hz, 1H), 7.34 (brs, 1H), 7.28 – 7.18 (m, 2H), 7.13 – 7.07 (m, 2H), 7.04 (d, *J* = 7.2 Hz, 1H), 5.60 (s, 1H), 5.47 (s, 1H), 4.80 (brs, 1H), 3.69 (d, *J* = 8.5 Hz, 1H); **<u>^{19}F NMR (376 MHz, CDCl$_3$)</u>** δ -107.12; **<u>^{13}C NMR (101 MHz, CDCl$_3$)</u>** δ 165.5 (d, *J* = 252.8 Hz), 162.5, 150.5, 145.5, 143.9, 142.3, 141.2, 140.5 (d, *J* = 8.3 Hz), 137.3, 136.5, 135.2, 133.4, 132.8, 132.0 (d, *J* = 9.4 Hz), 129.1, 128.6, 128.2, 128.2, 128.1, 127.9, 127.8, 127.5, 127.3, 126.7, 126.6, 123.6 (d, *J* = 2.6 Hz), 121.6, 120.6, 119.3, 114.8 (d, *J* = 21.7 Hz), 114.4 (d, *J* = 22.0 Hz), 88.7, 84.8, 63.6, 43.2; **<u>HRMS (ESI)</u>** calcd for $C_{36}H_{23}FN_2O_2$ $[M+H]^+$: 535.1816, Found: 535.1818; **<u>HPLC condition:</u>** The enantiomeric excess was determined by Daicel Chiralcel IA, Hexanes/IPA = 50/50, 1.2 mL/min, λ = 254 nm, t (minor) = 14.882 min, t (major) = 49.030 min.

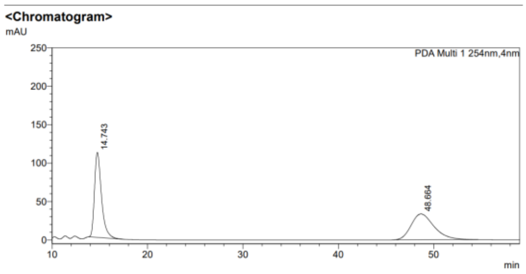

<Peak Table>
PDA Ch1 254nm

Peak#	Ret. Time	Area	Height	Area%
1	14.743	5620642	110708	49.865
2	48.664	5651044	33654	50.135
Total		11271687	144362	100.000

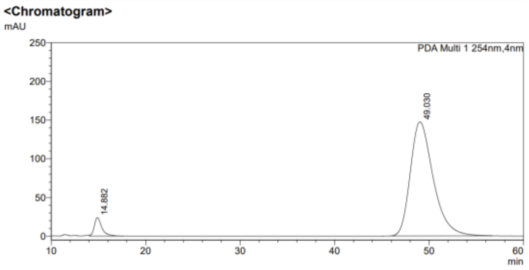

<Peak Table>
PDA Ch1 254nm

Peak#	Ret. Time	Area	Height	Area%
1	14.882	1327619	23942	5.068
2	49.030	24869295	147388	94.932
Total		26196915	171329	100.000

Reaction procedure and characterization of **3g-17**

To a 10 mL Schlenk tube was added **1g-17** (40.8 mg, 0.10 mmol), Ni(OTf)$_2$ (3.6 mg, 10 mol%), (*S*)-**L6** (6.2 mg, 20 mol%), EtCO$_2$Ag (36.0 mg, 0.20 mmol, 2.0 eq), **P7** (8.5 mg, 20 mol%) and **2a** (28.8 mg, 0.20 mmol, 2.0 eq), followed by addition of anhydrous DME (0.5 mL). Then the mixture was stirred at 100 °C for 24 h. After cooling to room temperature, the reaction system was quenched with aq. NaHCO$_3$ (10 mL) and extracted with CH$_2$Cl$_2$ (3×15 mL). The combined organic layer was dried over anhydrous Na$_2$SO$_4$, filtered, and concentrated in vacuo. After concentration, the crude product was purified by preparative TLC (PE/EtOAc = 2/1, v/v, R$_f$ = 0.54) to afford **3g-17** (37.1 mg, 67% yield, 89% ee) as a light-yellow oil.

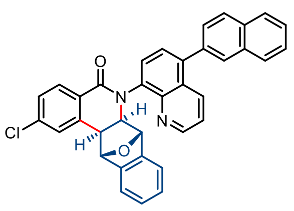

3g-17: $[\alpha]_D^{20}$ = -27.1 (c = 0.80, CHCl$_3$, 89% ee); **^{1}H NMR (400 MHz, CDCl$_3$)** δ 8.80 (brs, 1H), 8.35 (dd, *J* = 8.6, 1.6 Hz, 1H), 8.24 (d, *J* = 8.4 Hz, 1H), 8.09 – 7.91 (m, 5H), 7.79 (brs, 1H), 7.67 (dd, *J* = 8.3, 1.7 Hz, 1H), 7.62 – 7.55 (m, 2H), 7.55 (d, *J* = 2.0 Hz, 1H), 7.44 (d, *J* = 7.3 Hz, 1H), 7.40 – 7.35 (m, 2H), 7.25 (t, *J* = 7.4 Hz, 1H), 7.13 (t, *J* = 7.4 Hz, 1H), 7.06 (d, *J* = 7.2 Hz, 1H), 5.61 (s, 1H), 5.49 (s, 1H), 4.81 (brs, 1H), 3.69 (d, *J* = 8.5 Hz, 1H); **^{13}C NMR (101 MHz, CDCl$_3$)** δ 162.4, 150.5, 145.5, 143.9, 142.3, 141.2, 139.5, 138.6, 137.2, 136.4, 135.2, 133.4, 132.8, 131.7, 130.8, 129.1, 128.5, 128.2, 128.2, 128.1, 127.9, 127.9, 127.8, 127.7, 127.5, 127.3, 126.7, 126.6, 125.7, 121.7, 120.6, 119.3, 88.7, 84.7, 63.6, 42.9; **HRMS (ESI)** calcd for $C_{36}H_{23}ClN_2O_2$ $[M+H]^+$: 551.1521, Found: 551.1522; **HPLC condition:** The enantiomeric excess was determined by Daicel Chiralcel IA, Hexanes/IPA = 50/50, 1.2 mL/min, λ = 254 nm, t (minor) = 19.920 min, t (major) = 105.674 min.

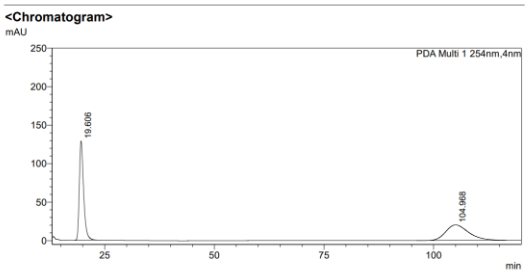

<Peak Table>

PDA Ch1 254nm

Peak#	Ret. Time	Area	Height	Area%
1	19.606	8801339	128854	54.509
2	104.968	7345388	20142	45.491
Total		16146726	148996	100.000

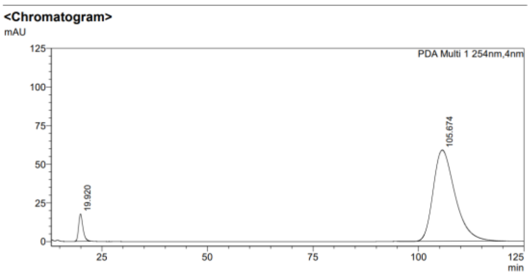

<Peak Table>

PDA Ch1 254nm

Peak#	Ret. Time	Area	Height	Area%
1	19.920	1254032	17700	5.563
2	105.674	21287104	59085	94.437
Total		22541136	76785	100.000

Reaction procedure and characterization of **3g-18**

To a 10 mL Schlenk tube was added **1g-18** (45.2 mg, 0.10 mmol), $Ni(OTf)_2$ (3.6 mg, 10 mol%), (*S*)-**L6** (6.2 mg, 20 mol%), $EtCO_2Ag$ (36.0 mg, 0.20 mmol, 2.0 eq), **P7** (8.5 mg, 20 mol%) and **2a** (28.8 mg, 0.20 mmol, 2.0 eq), followed by addition of anhydrous DME (0.5 mL). Then the mixture was stirred at 100 °C for 24 h. After cooling to room temperature, the reaction system was quenched with aq. $NaHCO_3$ (10 mL) and extracted with CH_2Cl_2 (3×15 mL). The combined organic layer was dried over anhydrous Na_2SO_4, filtered, and concentrated in vacuo. After concentration, the crude product was purified by preparative TLC (PE/acetone = 2/1, v/v, R_f = 0.57) to afford **3g-18** (34.5 mg, 58% yield, 88% ee) as a light-yellow foam.

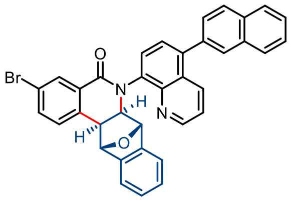

3g-18: $[\alpha]_D^{20}$ = -64.8 (c = 0.83, $CHCl_3$, 88% ee); **^{1}H NMR (400 MHz, $CDCl_3$)** δ 8.80 (brs, 1H), 8.43 (d, *J* = 2.2 Hz, 1H), 8.35 (dd, *J* = 8.6, 1.7 Hz, 1H), 8.11 – 7.92 (m, 5H), 7.79 (brs, 1H), 7.74 (dd, *J* = 8.2, 2.2 Hz, 1H), 7.67 (dd, *J* = 8.5, 1.6 Hz, 1H), 7.62 – 7.56 (m, 2H), 7.46 – 7.41 (m, 2H), 7.37 (brs, 1H), 7.26 – 7.21 (m, 1H), 7.12 (t, *J* = 7.4 Hz, 1H), 7.06 (d, *J* = 7.2 Hz, 1H), 5.61 (s, 1H), 5.43 (s, 1H), 4.79 (brs, 1H), 3.67 (d, *J* = 8.6 Hz, 1H); **^{13}C NMR (101 MHz, $CDCl_3$)** δ 162.0, 150.5, 145.5, 143.8, 142.3, 141.3, 137.1, 136.6, 136.4, 135.5, 135.2, 133.4, 132.8, 131.8, 131.7, 129.9, 129.1, 128.9, 128.6, 128.2, 128.2, 128.1, 127.9, 127.8, 127.5, 127.3, 126.7, 126.6, 121.7, 121.2, 120.6, 119.3, 88.7, 84.7, 63.6, 42.8; **HRMS (ESI)** calcd for $C_{36}H_{23}BrN_2O_2$ $[M+H]^+$: 595.1016, Found: 595.1016; **HPLC condition:** The enantiomeric excess was determined by Daicel Chiralcel IA, Hexanes/IPA = 50/50, 1.5 mL/min, λ = 254 nm, t (major) = 59.537 min, t (minor) = 79.061 min.

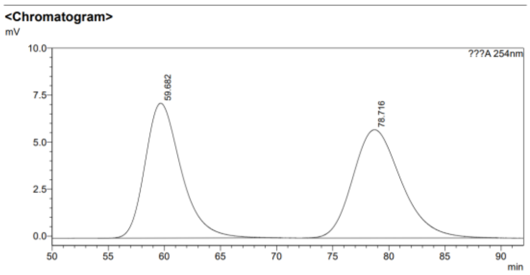

<Peak Table>
???A 254nm

Peak#	Ret. Time	Area	Height	Area%
1	59.682	1601483	7172	48.069
2	78.716	1730135	5758	51.931
Total		3331618	12930	100.000

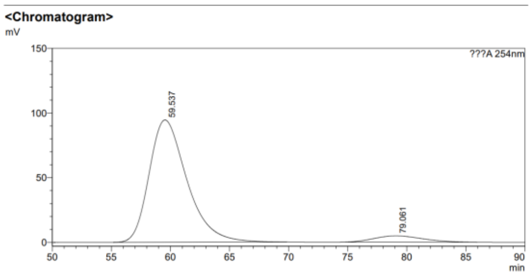

<Peak Table>
???A 254nm

Peak#	Ret. Time	Area	Height	Area%
1	59.537	21059637	94539	93.768
2	79.061	1399590	4868	6.232
Total		22459228	99408	100.000

Reaction procedure and characterization of **3g-19**

To a 10 mL Schlenk tube was added **1g-19** (45.2 mg, 0.10 mmol), $Ni(OTf)_2$ (3.6 mg, 10 mol%), (*S*)-**L6** (6.2 mg, 20 mol%), $EtCO_2Ag$ (36.0 mg, 0.20 mmol, 2.0 eq), **P7** (8.5 mg, 20 mol%) and **2a** (28.8 mg, 0.20 mmol, 2.0 eq), followed by addition of anhydrous DME (0.5 mL). Then the mixture was stirred at 100 °C for 24 h. After cooling to room temperature, the reaction system was quenched with aq. $NaHCO_3$ (10 mL) and extracted with CH_2Cl_2 (3×15 mL). The combined organic layer was dried over anhydrous Na_2SO_4, filtered, and concentrated in vacuo. After concentration, the crude product was purified by preparative TLC (PE/EtOAc = 2/1, v/v, R_f = 0.57) to afford **3g-19** (39.2 mg, 66% yield, 90% ee) as a light-yellow oil.

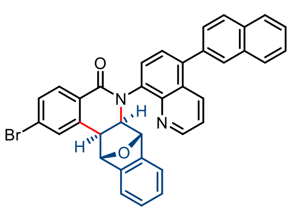

3g-19: $[\alpha]_D^{20}$ = -16.6 (c = 0.83, $CHCl_3$, 90% ee); **^{1}H NMR (400 MHz, CDCl$_3$)** δ 8.80 (brs, 1H), 8.34 (dd, *J* = 8.6, 1.7 Hz, 1H), 8.16 (d, *J* = 8.4 Hz, 1H), 8.07 – 7.90 (m, 5H), 7.79 (brs, 1H), 7.71 (d, *J* = 1.9 Hz, 1H), 7.67 (dd, *J* = 8.4, 1.7 Hz, 1H), 7.62 – 7.57 (m, 2H), 7.55 (dd, *J* = 8.4, 1.9 Hz, 1H), 7.44 (d, *J* = 7.3 Hz, 1H), 7.36 (brs, 1H), 7.27 – 7.23 (m, 1H), 7.13 (t, *J* = 7.4 Hz, 1H), 7.06 (d, *J* = 7.2 Hz, 1H), 5.61 (s, 1H), 5.48 (s, 1H), 4.80 (brs, 1H), 3.69 (d, *J* = 8.5 Hz, 1H); **^{13}C NMR (101 MHz, CDCl$_3$)** δ 162.6, 150.5, 145.4, 143.8, 142.3, 141.2, 139.7, 137.1, 136.4, 135.2, 133.4, 132.8, 131.7, 130.9, 130.9, 130.6, 129.1, 128.5, 128.2, 128.2, 128.1, 127.9, 127.8, 127.5, 127.3, 127.2, 126.7, 126.6, 126.2, 121.7, 120.6, 119.3, 88.6, 84.7, 63.6, 42.9; **HRMS (ESI)** calcd for $C_{36}H_{23}BrN_2O_2$ $[M+H]^+$: 595.1016, Found: 595.1018; **HPLC condition:** The enantiomeric excess was determined by Daicel Chiralcel IA, Hexanes/IPA = 50/50, 1.2 mL/min, λ = 254 nm, t (minor) = 23.023 min, t (major) = 137.591 min.

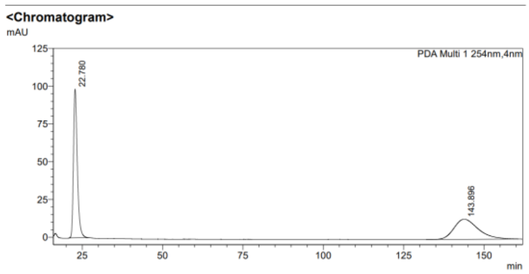

<Peak Table>
PDA Ch1 254nm

Peak#	Ret. Time	Area	Height	Area%
1	22.780	8186324	98452	54.155
2	143.896	6930040	13250	45.845
Total		15116364	111702	100.000

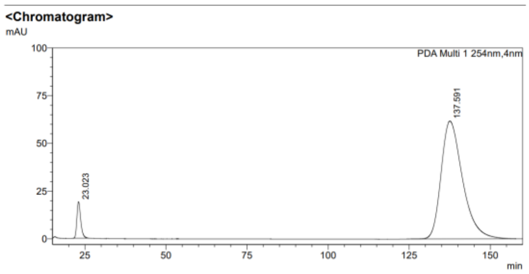

<Peak Table>
PDA Ch1 254nm

Peak#	Ret. Time	Area	Height	Area%
1	23.023	1564236	19076	5.226
2	137.591	28366223	61725	94.774
Total		29930460	80801	100.000

Reaction procedure and characterization of **3g-20**

To a 10 mL Schlenk tube was added **1g-20** (50.0 mg, 0.10 mmol), $Ni(OTf)_2$ (3.6 mg, 10 mol%), (*S*)-**L6** (6.2 mg, 20 mol%), $EtCO_2Ag$ (36.0 mg, 0.20 mmol, 2.0 eq), **P7** (8.5 mg, 20 mol%) and **2a** (28.8 mg, 0.20 mmol, 2.0 eq), followed by addition of anhydrous DME (0.5 mL). Then the mixture was stirred at 100 °C for 24 h. After cooling to room temperature, the reaction system was quenched with aq. $NaHCO_3$ (10 mL) and extracted with CH_2Cl_2 (3×15 mL). The combined organic layer was dried over anhydrous Na_2SO_4, filtered, and concentrated in vacuo. After concentration, the crude product was purified by preparative TLC (PE/acetone = 2/1, v/v, R_f = 0.54) to afford **3g-20** (39.8 mg, 62% yield, 87% ee) as a light-yellow foam.

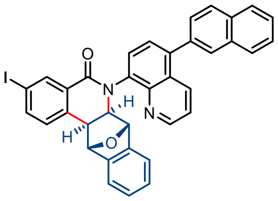

3g-20: $[\alpha]_D^{20}$ = -75.6 (c = 0.94, $CHCl_3$, 87% ee); **^{1}H NMR (400 MHz, $CDCl_3$)** δ 8.80 (brs, 1H), 8.63 (d, *J* = 1.9 Hz, 1H), 8.35 (dd, *J* = 8.6, 1.7 Hz, 1H), 8.09 – 7.89 (m, 6H), 7.79 (brs, 1H), 7.67 (dd, *J* = 8.4, 1.6 Hz, 1H), 7.62 – 7.58 (m, 2H), 7.42 (d, *J* = 7.3 Hz, 1H), 7.36 (brs, 1H), 7.30 (d, *J* = 8.2 Hz, 1H), 7.24 (t, *J* = 7.4 Hz, 1H), 7.12 (t, *J* = 7.3 Hz, 1H), 7.05 (d, *J* = 7.2 Hz, 1H), 5.60 (s, 1H), 5.43 (s, 1H), 4.78 (brs, 1H), 3.66 (d, *J* = 8.5 Hz, 1H); **^{13}C NMR (101 MHz, $CDCl_3$)** δ 161.7, 150.5, 145.5, 143.8, 142.3, 141.2, 137.8, 137.3, 136.4, 135.2, 133.4, 132.8, 131.7, 130.0, 129.1, 128.9, 128.6, 128.2, 128.2, 128.1, 127.9, 127.8, 127.5, 127.3, 126.7, 126.6, 121.7, 120.6, 119.3, 92.2, 88.7, 84.7, 63.5, 42.9; **HRMS (ESI)** calcd for $C_{36}H_{23}IN_2O_2$ $[M+H]^+$: 643.0877, Found: 643.0880; **HPLC condition:** The enantiomeric excess was determined by Daicel Chiralcel IA, Hexanes/IPA = 50/50, 1.5 mL/min, λ = 254 nm, t (major) = 59.444 min, t (minor) = 112.244 min.

<Chromatogram>

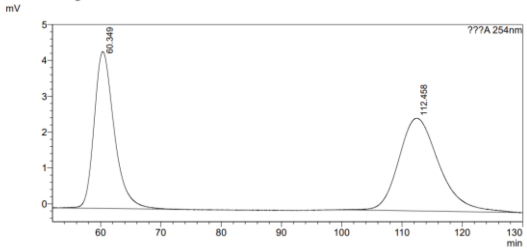

<Peak Table>

???A 254nm

Peak#	Ret. Time	Area	Height	Area%
1	60.349	1013438	4373	45.689
2	112.458	1204686	2587	54.311
Total		2218124	6960	100.000

<Chromatogram>

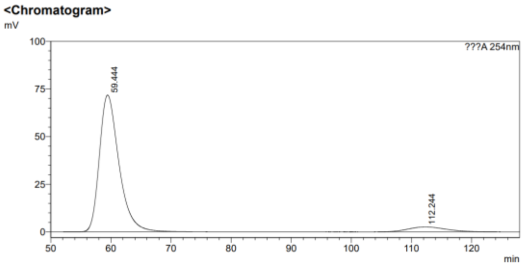

<Peak Table>

???A 254nm

Peak#	Ret. Time	Area	Height	Area%
1	59.444	16220946	71805	93.389
2	112.244	1148273	2606	6.611
Total		17369219	74411	100.000

Reaction procedure and characterization of **3g-21**

To a 10 mL Schlenk tube was added **1g-21** (50.0 mg, 0.10 mmol), $Ni(OTf)_2$ (3.6 mg, 10 mol%), (*S*)-**L6** (6.2 mg, 20 mol%), $EtCO_2Ag$ (36.0 mg, 0.20 mmol, 2.0 eq), **P7** (8.5 mg, 20 mol%) and **2a** (28.8 mg, 0.20 mmol, 2.0 eq), followed by addition of anhydrous DME (0.5 mL). Then the mixture was stirred at 100 °C for 24 h. After cooling to room temperature, the reaction system was quenched with aq. $NaHCO_3$ (10 mL) and extracted with CH_2Cl_2 (3×15 mL). The combined organic layer was dried over anhydrous Na_2SO_4, filtered, and concentrated in vacuo. After concentration, the crude product was purified by preparative TLC (PE/EtOAc = 2/1, v/v, R_f = 0.57) to afford **3g-21** (41.8 mg, 65% yield, 88% ee) as a light-yellow oil.

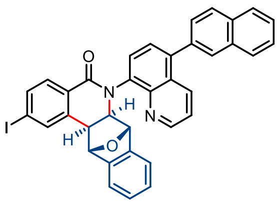

3g-21: $[\alpha]_D^{20}$ = -5.0 (c = 1.00, $CHCl_3$, 88% ee); **^{1}H NMR (400 MHz, $CDCl_3$)** δ 8.79 (brs, 1H), 8.34 (dd, *J* = 8.6, 1.7 Hz, 1H), 8.08 – 7.90 (m, 7H), 7.79 – 7.75 (m, 2H), 7.66 (dd, *J* = 8.4, 1.7 Hz, 1H), 7.62 – 7.57 (m, 2H), 7.45 (d, *J* = 7.3 Hz, 1H), 7.36 (brs, 1H), 7.27 – 7.23 (m, 1H), 7.12 (t, *J* = 7.6 Hz, 1H), 7.06 (d, *J* = 7.3 Hz, 1H), 5.60 (s, 1H), 5.48 (s, 1H), 4.79 (brs, 1H), 3.66 (d, *J* = 8.5 Hz, 1H); **^{13}C NMR (101 MHz, $CDCl_3$)** δ 162.8, 150.5, 145.5, 143.9, 142.3, 141.2, 139.6, 137.2, 137.0, 136.5, 136.4, 135.1, 133.3, 132.8, 131.7, 130.7, 129.1, 128.5, 128.2, 128.2, 128.1, 127.9, 127.8, 127.5, 127.3, 126.7, 126.6, 121.7, 120.6, 119.3, 99.7, 88.6, 84.7, 63.6, 42.6; **HRMS (ESI)** calcd for $C_{36}H_{23}IN_2O_2$ $[M+H]^+$: 643.0877, Found: 643.0879; **HPLC condition:** The enantiomeric excess was determined by Daicel Chiralcel IA, Hexanes/IPA = 50/50, 1.2 mL/min, λ = 254 nm, t (minor) = 25.484 min, t (major) = 166.490 min.

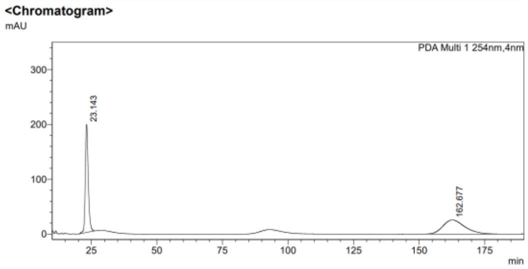

<Peak Table>

PDA Ch1 254nm

Peak#	Ret. Time	Area	Height	Area%
1	23.143	15358538	197269	50.610
2	162.677	14988362	25727	49.390
Total		30346901	222996	100.000

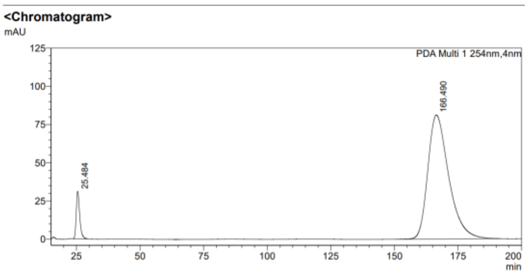

<Peak Table>

PDA Ch1 254nm

Peak#	Ret. Time	Area	Height	Area%
1	25.484	2914117	31075	5.912
2	166.490	46375320	81175	94.088
Total		49289437	112250	100.000

Reaction procedure and characterization of **3g-22**

To a 10 mL Schlenk tube was added **1g-22** (41.6 mg, 0.10 mmol), Ni(OTf)$_2$ (3.6 mg, 10 mol%), (*S*)-**L6** (6.2 mg, 20 mol%), EtCO$_2$Ag (36.0 mg, 0.20 mmol, 2.0 eq), **P7** (8.5 mg, 20 mol%) and **2a** (28.8 mg, 0.20 mmol, 2.0 eq), followed by addition of anhydrous DME (0.5 mL). Then the mixture was stirred at 100 °C for 24 h. After cooling to room temperature, the reaction system was quenched with aq. NaHCO$_3$ (10 mL) and extracted with CH$_2$Cl$_2$ (3×15 mL). The combined organic layer was dried over anhydrous Na$_2$SO$_4$, filtered, and concentrated in vacuo. After concentration, the crude product was purified by preparative TLC (PE/acetone = 2/1, v/v, R$_f$ = 0.32) to afford **3g-22** (27.5 mg, 49% yield, 87% ee) as a light-yellow foam.

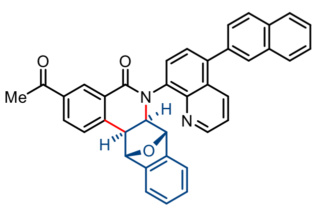

3g-22: $[\alpha]_D^{20}$ = -65.4 (c = 0.99, CHCl$_3$, 87% ee); **^{1}H NMR (400 MHz, CDCl$_3$)** δ 8.85 (d, *J* = 1.9 Hz, 1H), 8.80 (brs, 1H), 8.36 (d, *J* = 8.6 Hz, 1H), 8.27 (dd, *J* = 8.1, 2.0 Hz, 1H), 8.08 – 7.91 (m, 5H), 7.80 (brs, 1H), 7.67 (d, *J* = 7.9 Hz, 2H), 7.62 – 7.57 (m, 2H), 7.45 (d, *J* = 7.3 Hz, 1H), 7.38 (brs, 1H), 7.25 (t, *J* = 7.4 Hz, 1H), 7.13 (t, *J* = 7.4 Hz, 1H), 7.08 (d, *J* = 7.2 Hz, 1H), 5.64 (s, 1H), 5.49 (s, 1H), 4.82 (brs, 1H), 3.78 (d, *J* = 8.5 Hz, 1H), 2.67 (s, 3H); **^{13}C NMR (101 MHz, CDCl$_3$)** δ 197.7, 162.5, 150.6, 145.5, 143.9, 142.8, 142.3, 141.3, 137.1, 136.4, 136.1, 135.2, 133.4, 132.8, 131.6, 131.3, 129.8, 129.1, 128.8, 128.5, 128.3, 128.2, 128.1, 127.9, 127.8, 127.5, 127.4, 127.3, 126.7, 126.6, 121.7, 120.6, 119.3, 88.6, 84.7, 63.6, 43.4, 26.8; **HRMS (ESI)** calcd for $C_{38}H_{26}N_2O_3$ $[M+H]^+$: 559.2016, Found: 559.2018; **HPLC condition:** The enantiomeric excess was determined by Daicel Chiralcel IA, Hexanes/IPA = 50/50, 1.6 mL/min, λ = 254 nm, t (major) = 71.059 min, t (minor) = 97.412 min.

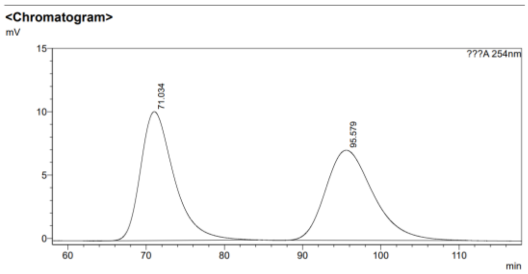

<Peak Table>
???A 254nm

Peak#	Ret. Time	Area	Height	Area%
1	71.034	3016529	10174	50.099
2	95.579	3004665	7116	49.901
Total		6021194	17290	100.000

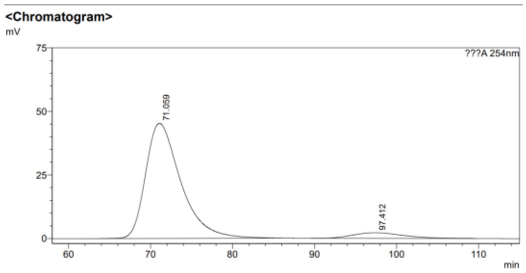

<Peak Table>
???A 254nm

Peak#	Ret. Time	Area	Height	Area%
1	71.059	13509332	45269	93.347
2	97.412	962801	2228	6.653
Total		14472133	47497	100.000

Reaction procedure and characterization of **3g-23**

To a 10 mL Schlenk tube was added **1g-23** (41.6 mg, 0.10 mmol), Ni(OTf)$_2$ (3.6 mg, 10 mol%), (*S*)-**L6** (6.2 mg, 20 mol%), EtCO$_2$Ag (36.0 mg, 0.20 mmol, 2.0 eq), **P7** (8.5 mg, 20 mol%) and **2a** (28.8 mg, 0.20 mmol, 2.0 eq), followed by addition of anhydrous DME (0.5 mL). Then the mixture was stirred at 100 °C for 24 h. After cooling to room temperature, the reaction system was quenched with aq. NaHCO$_3$ (10 mL) and extracted with CH$_2$Cl$_2$ (3×15 mL). The combined organic layer was dried over anhydrous Na$_2$SO$_4$, filtered, and concentrated in vacuo. After concentration, the crude product was purified by preparative TLC (PE/EtOAc = 2/1, v/v, R$_f$ = 0.09) to afford **3g-23** (31.9 mg, 57% yield, 93% ee) as a light-yellow foam.

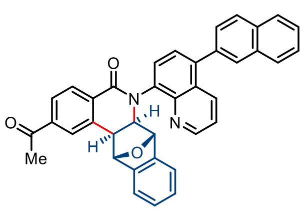

3g-23: $[\alpha]_D^{20}$ = -18.5 (c = 1.01, CHCl$_3$, 93% ee); **^{1}H NMR (400 MHz, CDCl$_3$)** δ 8.79 (brs, 1H), 8.40 (d, *J* = 8.1 Hz, 1H), 8.35 (d, *J* = 8.4 Hz, 1H), 8.19 (s, 1H), 8.08 – 7.91 (m, 6H), 7.80 (brs, 1H), 7.67 (dd, *J* = 8.3, 1.7 Hz, 1H), 7.61 – 7.57 (m, 2H), 7.49 (d, *J* = 7.3 Hz, 1H), 7.37 (brs, 1H), 7.29 – 7.23 (m, 1H), 7.13 (t, *J* = 7.4 Hz, 1H), 7.06 (d, *J* = 7.3 Hz, 1H), 5.62 (s, 1H), 5.51 (s, 1H), 4.83 (brs, 1H), 3.79 (d, *J* = 8.6 Hz, 1H), 2.73 (s, 3H); **^{13}C NMR (101 MHz, CDCl$_3$)** δ 198.0, 162.3, 150.6, 145.4, 143.8, 142.3, 141.3, 139.9, 138.3, 137.1, 136.4, 135.2, 133.3, 132.8, 131.6, 130.8, 129.5, 129.1, 128.5, 128.3, 128.2, 128.1, 127.9, 127.8, 127.5, 127.3, 127.2, 126.7, 126.6, 121.7, 120.5, 119.5, 88.8, 84.7, 63.7, 43.0, 27.1; **HRMS (ESI)** calcd for $C_{38}H_{26}N_2O_3$ $[M+H]^+$: 559.2016, Found: 559.2019; **HPLC condition:** The enantiomeric excess was determined by Daicel Chiralcel IA, Hexanes/IPA = 50/50, 1.2 mL/min, λ = 254 nm, t (minor) = 19.042 min, t (major) = 70.511 min.

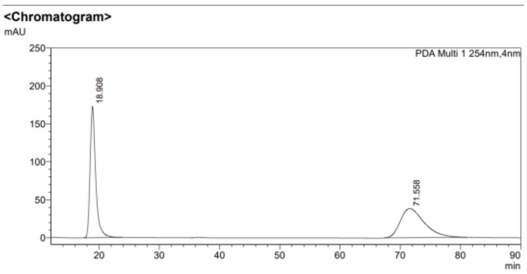

<Peak Table>
PDA Ch1 254nm

Peak#	Ret. Time	Area	Height	Area%
1	18.908	11175844	173263	51.085
2	71.558	10701092	38399	48.915
Total		21876936	211662	100.000

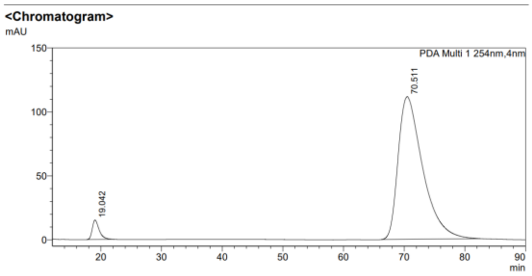

<Peak Table>
PDA Ch1 254nm

Peak#	Ret. Time	Area	Height	Area%
1	19.042	1195593	15147	3.672
2	70.511	31359961	111744	96.328
Total		32555554	126891	100.000

Reaction procedure and characterization of **3g-24**

To a 10 mL Schlenk tube was added **1g-24** (43.2 mg, 0.10 mmol), $Ni(OTf)_2$ (3.6 mg, 10 mol%), (*S*)-**L6** (6.2 mg, 20 mol%), $EtCO_2Ag$ (36.0 mg, 0.20 mmol, 2.0 eq), **P7** (8.5 mg, 20 mol%) and **2a** (28.8 mg, 0.20 mmol, 2.0 eq), followed by addition of anhydrous DME (0.5 mL). Then the mixture was stirred at 100 °C for 24 h. After cooling to room temperature, the reaction system was quenched with aq. $NaHCO_3$ (10 mL) and extracted with CH_2Cl_2 (3×15 mL). The combined organic layer was dried over anhydrous Na_2SO_4, filtered, and concentrated in vacuo. After concentration, the crude product was purified by preparative TLC (PE/acetone = 2/1, v/v, R_f = 0.41) to afford **3g-24** (26.8 mg, 47% yield, 90% ee) as a light-yellow foam.

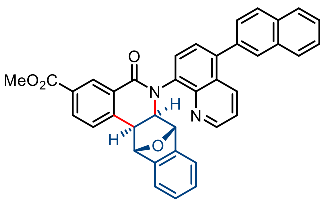

3g-24: $[\alpha]_D^{20}$ = -68.0 (c = 0.90, $CHCl_3$, 90% ee); **^{1}H NMR (400 MHz, $CDCl_3$)** δ 8.94 (d, *J* = 1.8 Hz, 1H), 8.79 (brs, 1H), 8.34 (dd, *J* = 8.6, 1.7 Hz, 1H), 8.28 (dd, *J* = 8.1, 1.9 Hz, 1H), 8.08 – 7.90 (m, 5H), 7.79 (brs, 1H), 7.70 – 7.62 (m, 2H), 7.61 – 7.55 (m, 2H), 7.43 (d, *J* = 7.2 Hz, 1H), 7.36 (brs, 1H), 7.25 – 7.21 (m, 1H), 7.12 (t, *J* = 7.4 Hz, 1H), 7.06 (d, *J* = 7.2 Hz, 1H), 5.61 (s, 1H), 5.47 (s, 1H), 4.82 (brs, 1H), 3.93 (s, 3H), 3.76 (d, *J* = 8.5 Hz, 1H); **^{13}C NMR (101 MHz, $CDCl_3$)** δ 166.5, 162.3, 150.5, 145.5, 143.8, 142.5, 142.3, 141.2, 137.2, 136.4, 135.2, 133.4, 133.2, 132.8, 131.7, 130.5, 129.4, 129.1, 128.5, 128.2, 128.2, 128.1, 127.9, 127.8, 127.5, 127.5, 127.4, 126.7, 126.6, 121.7, 120.6, 119.3, 88.7, 84.7, 63.5, 52.3, 43.4; **HRMS (ESI)** calcd for $C_{38}H_{26}N_2O_4$ $[M+H]^+$: 575.1965, Found: 575.1966; **HPLC condition:** The enantiomeric excess was determined by Daicel Chiralcel IA, Hexanes/IPA = 50/50, 1.5 mL/min, λ = 254 nm, t (major) = 80.308 min, t (minor) = 120.190 min.

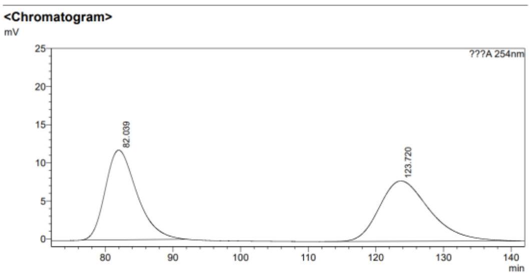

<Peak Table>
???A 254nm

Peak#	Ret. Time	Area	Height	Area%
1	82.039	3827387	11755	48.236
2	123.720	4107269	7918	51.764
Total		7934657	19674	100.000

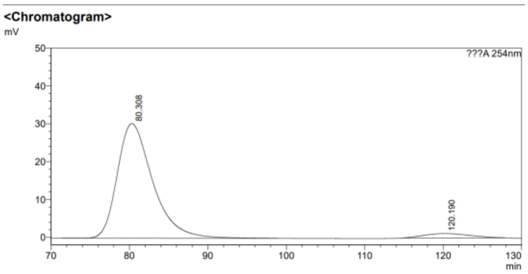

<Peak Table>
???A 254nm

Peak#	Ret. Time	Area	Height	Area%
1	80.308	9492917	30279	95.226
2	120.190	475877	1164	4.774
Total		9968795	31444	100.000

Reaction procedure and characterization of **3g-25**

To a 10 mL Schlenk tube was added **1g-25** (43.2 mg, 0.10 mmol), $Ni(OTf)_2$ (3.6 mg, 10 mol%), (*S*)-**L6** (6.2 mg, 20 mol%), $EtCO_2Ag$ (36.0 mg, 0.20 mmol, 2.0 eq), **P7** (8.5 mg, 20 mol%) and **2a** (28.8 mg, 0.20 mmol, 2.0 eq), followed by addition of anhydrous DME (0.5 mL). Then the mixture was stirred at 100 °C for 24 h. After cooling to room temperature, the reaction system was quenched with aq. $NaHCO_3$ (10 mL) and extracted with CH_2Cl_2 (3×15 mL). The combined organic layer was dried over anhydrous Na_2SO_4, filtered, and concentrated in vacuo. After concentration, the crude product was purified by preparative TLC (PE/EtOAc = 2/1, v/v, R_f = 0.20) to afford **3g-25** (34.9 mg, 61% yield, 89% ee) as a light-yellow oil.

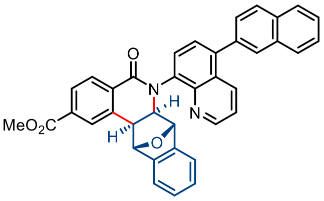

3g-25: $[\alpha]_D^{20}$ = -23.8 (c = 1.07, $CHCl_3$, 89% ee); **^{1}H NMR (400 MHz, $CDCl_3$)** δ 8.80 (brs, 1H), 8.41 – 8.32 (m, 2H), 8.26 (s, 1H), 8.11 – 7.91 (m, 5H), 7.80 (brs, 1H), 7.67 (dd, *J* = 8.4, 1.6 Hz, 1H), 7.63 – 7.56 (m, 2H), 7.48 (d, *J* = 7.3 Hz, 1H), 7.37 (brs, 1H), 7.29 – 7.22 (m, 1H), 7.13 (t, *J* = 7.4 Hz, 1H), 7.06 (d, *J* = 7.2 Hz, 1H), 5.62 (s, 1H), 5.52 (s, 1H), 4.84 (brs, 1H), 4.03 (s, 3H), 3.78 (d, *J* = 8.6 Hz, 1H); **^{13}C NMR (101 MHz, $CDCl_3$)** δ 166.6, 162.3, 150.6, 145.5, 143.8, 142.3, 141.3, 137.9, 137.1, 136.4, 135.2, 133.5, 133.4, 132.8, 131.7, 130.7, 129.7, 129.3, 129.1, 128.6, 128.2, 128.2, 128.1, 128.0, 127.9, 127.8, 127.5, 127.3, 126.7, 126.6, 121.7, 120.5, 119.5, 88.8, 84.6, 63.6, 52.6, 43.0; **HRMS (ESI)** calcd for $C_{38}H_{26}N_2O_4$ $[M+H]^+$: 575.1965, Found: 575.1963; **HPLC condition:** The enantiomeric excess was determined by Daicel Chiralcel IA, Hexanes/IPA = 50/50, 1.2 mL/min, λ = 254 nm, t (minor) = 18.780 min, t (major) = 66.333 min.

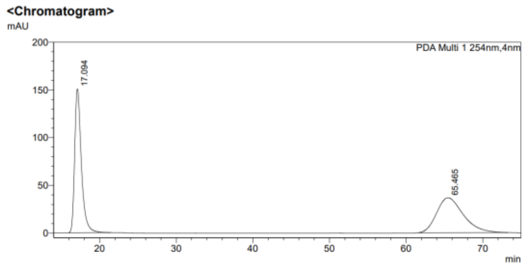

<Peak Table>

PDA Ch1 254nm

Peak#	Ret. Time	Area	Height	Area%
1	17.094	9197393	150807	50.837
2	65.465	8894644	36696	49.163
Total		18092038	187503	100.000

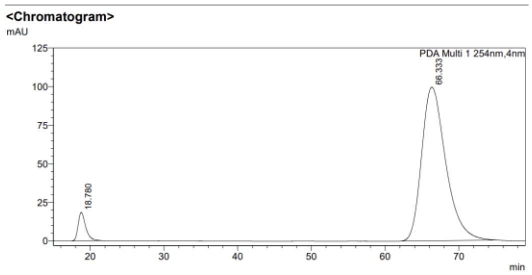

<Peak Table>

PDA Ch1 254nm

Peak#	Ret. Time	Area	Height	Area%
1	18.780	1317123	18471	5.497
2	66.333	22645255	99536	94.503
Total		23962379	118007	100.000

Reaction procedure and characterization of **3g-26**

To a 10 mL Schlenk tube was added **1g-26** (39.9 mg, 0.10 mmol), Ni(OTf)$_2$ (3.6 mg, 10 mol%), (*S*)-**L6** (6.2 mg, 20 mol%), EtCO$_2$Ag (36.0 mg, 0.20 mmol, 2.0 eq), **P7** (8.5 mg, 20 mol%) and **2a** (28.8 mg, 0.20 mmol, 2.0 eq), followed by addition of anhydrous DME (0.5 mL). Then the mixture was stirred at 100 °C for 24 h. After cooling to room temperature, the reaction system was quenched with aq. NaHCO$_3$ (10 mL) and extracted with CH$_2$Cl$_2$ (3×15 mL). The combined organic layer was dried over anhydrous Na$_2$SO$_4$, filtered, and concentrated in vacuo. After concentration, the crude product was purified by preparative TLC (PE/EtOAc = 2/1, v/v, R$_f$ = 0.20) to afford **3g-26** (29.3 mg, 54% yield, 90% ee) as a light-yellow oil.

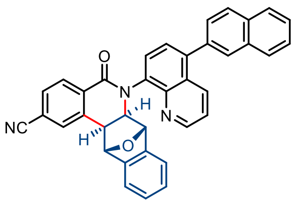

3g-26: $[\alpha]_D^{20}$ = -15.8 (c = 0.66, CHCl$_3$, 90% ee); **^{1}H NMR (400 MHz, CDCl$_3$)** δ 8.80 (brs, 1H), 8.41 (d, *J* = 8.1 Hz, 1H), 8.36 (dd, *J* = 8.7, 1.6 Hz, 1H), 8.09 – 7.92 (m, 5H), 7.90 (s, 1H), 7.80 (brs, 1H), 7.71 – 7.65 (m, 2H), 7.62 – 7.58 (m, 2H), 7.47 (d, *J* = 7.3 Hz, 1H), 7.38 (s, 1H), 7.27 (t, *J* = 7.4 Hz, 1H), 7.15 (t, *J* = 7.4 Hz, 1H), 7.08 (d, *J* = 7.3 Hz, 1H), 5.63 (s, 1H), 5.46 (s, 1H), 4.83 (brs, 1H), 3.75 (d, *J* = 8.5 Hz, 1H); **^{13}C NMR (101 MHz, CDCl$_3$)** δ 161.6, 150.6, 145.1, 143.7, 142.1, 141.5, 138.9, 136.7, 136.3, 135.2, 133.3, 132.8, 132.2, 131.5, 130.8, 130.5, 129.9, 129.1, 128.6, 128.3, 128.2, 128.0, 128.0, 127.9, 127.5, 127.5, 126.8, 126.7, 121.8, 120.6, 119.5, 118.3, 115.9, 88.7, 84.6, 63.6, 42.8; **HRMS (ESI)** calcd for $C_{37}H_{23}N_3O_2$ $[M+H]^+$: 542.1863, Found: 542.1867; **HPLC condition:** The enantiomeric excess was determined by Daicel Chiralcel IA, Hexanes/IPA = 50/50, 1.5 mL/min, λ = 254 nm, t (minor) = 8.744 min, t (major) = 70.918 min.

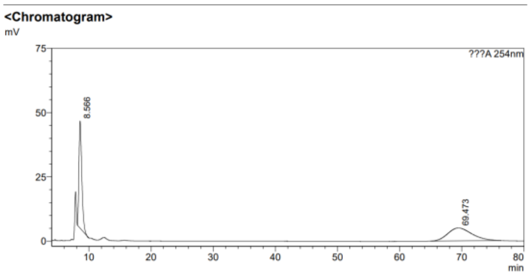

<Peak Table>

???A 254nm

Peak#	Ret. Time	Area	Height	Area%
1	8.566	1323364	41592	49.281
2	69.473	1361969	5025	50.719
Total		2685333	46617	100.000

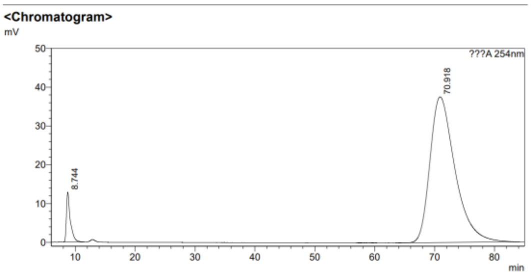

<Peak Table>

???A 254nm

Peak#	Ret. Time	Area	Height	Area%
1	8.744	577830	12884	5.029
2	70.918	10912968	37536	94.971
Total		11490798	50420	100.000

Reaction procedure and characterization of **3g-27**

To a 10 mL Schlenk tube was added **1g-27** (38.0 mg, 0.10 mmol), Ni(OTf)$_2$ (3.6 mg, 10 mol%), (*S*)-**L6** (6.2 mg, 20 mol%), $EtCO_2Ag$ (36.0 mg, 0.20 mmol, 2.0 eq), **P7** (8.5 mg, 20 mol%) and **2a** (28.8 mg, 0.20 mmol, 2.0 eq), followed by addition of anhydrous DME (0.5 mL). Then the mixture was stirred at 100 °C for 24 h. After cooling to room temperature, the reaction system was quenched with aq. $NaHCO_3$ (10 mL) and extracted with CH_2Cl_2 (3×15 mL). The combined organic layer was dried over anhydrous Na_2SO_4, filtered, and concentrated in vacuo. After concentration, the crude product was purified by preparative TLC (PE/EtOAc = 2/1, v/v, R_f = 0.09) to afford **3g-27** (32.6 mg, 62% yield, 92% ee) as a light-yellow oil.

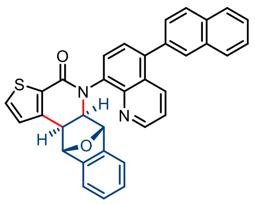

3g-27: $[\alpha]_D^{20}$ = -81.5 (c = 0.53, $CHCl_3$, 92% ee); **^{1}H NMR (400 MHz, $CDCl_3$)** δ 8.83 (brs, 1H), 8.34 (dd, *J* = 8.6, 1.7 Hz, 1H), 8.11 – 7.91 (m, 5H), 7.77 (brs, 1H), 7.67 (dd, *J* = 8.5, 1.6 Hz, 1H), 7.65 – 7.54 (m, 3H), 7.42 – 7.35 (m, 2H), 7.25 – 7.17 (m, 2H), 7.10 (t, *J* = 7.4 Hz, 1H), 7.02 (d, *J* = 7.3 Hz, 1H), 5.60 (s, 1H), 5.48 (s, 1H), 4.91 (brs, 1H), 3.74 (d, *J* = 8.6 Hz, 1H); **^{13}C NMR (101 MHz, $CDCl_3$)** δ 160.2, 150.4, 145.3, 144.1, 142.6, 142.0, 141.1, 136.6, 136.5, 135.2, 133.3, 132.8, 132.2, 132.1, 131.7, 129.1, 128.5, 128.2, 128.1, 127.9, 127.7, 127.5, 127.2, 126.7, 126.7, 126.6, 121.6, 120.4, 119.4, 86.5, 84.8, 66.0, 42.1; **HRMS (ESI)** calcd for $C_{34}H_{22}N_2O_2S$ $[M+H]^+$: 523.1475, Found: 523.1478; **HPLC condition:** The enantiomeric excess was determined by Daicel Chiralcel IA, Hexanes/IPA = 50/50, 1.5 mL/min, λ = 254 nm, t (minor) = 11.075 min, t (major) = 63.167 min.

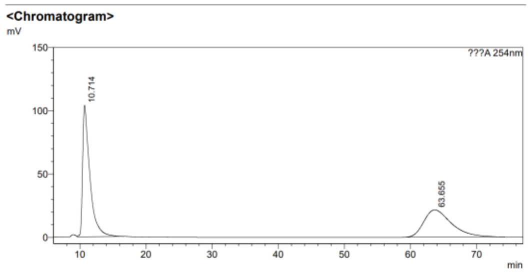

<Peak Table>
???A 254nm

Peak#	Ret. Time	Area	Height	Area%
1	10.714	7905726	103828	55.589
2	63.655	6316040	21587	44.411
Total		14221766	125415	100.000

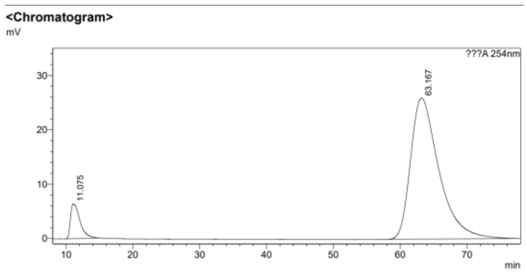

<Peak Table>
???A 254nm

Peak#	Ret. Time	Area	Height	Area%
1	11.075	630565	6387	7.604
2	63.167	7661623	25976	92.396
Total		8292187	32363	100.000

Reaction procedure and characterization of **4g-1**

To a 10 mL Schlenk tube was added **1g-1** (37.4 mg, 0.10 mmol), Ni(OTf)$_2$ (3.6 mg, 10 mol%), (*S*)-**L6** (6.2 mg, 20 mol%), EtCO$_2$Ag (36.0 mg, 0.20 mmol, 2.0 eq), **P7** (8.5 mg, 20 mol%) and **2b** (34.4 mg, 0.20 mmol, 2.0 eq), followed by addition of anhydrous DME (0.5 mL). Then the mixture was stirred at 100 °C for 24 h. After cooling to room temperature, the reaction system was quenched with aq. NaHCO$_3$ (10 mL) and extracted with CH$_2$Cl$_2$ (3×15 mL). The combined organic layer was dried over anhydrous Na$_2$SO$_4$, filtered, and concentrated in vacuo. After concentration, the crude product was purified by preparative TLC (PE/EtOAc = 2/1, v/v, R$_f$ = 0.24) to afford **4g-1** (34.3 mg, 63% yield, 91% ee) as a light-yellow oil.

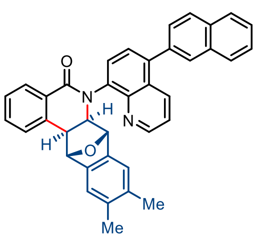

4g-1: $[\alpha]_D^{20}$ = -64.2 (c = 1.03, CHCl$_3$, 91% ee); **<u>^{1}H NMR (400 MHz, CDCl$_3$)</u>** δ 8.81 (brs, 1H), 8.36 – 8.30 (m, 2H), 8.10 – 7.92 (m, 5H), 7.79 (brs, 1H), 7.68 (dd, *J* = 8.4, 1.7 Hz, 1H), 7.65 – 7.57 (m, 3H), 7.53 (d, *J* = 7.7 Hz, 1H), 7.42 (t, *J* = 7.6 Hz, 1H), 7.35 (brs, 1H), 7.23 (s, 1H), 6.84 (s, 1H), 5.56 (s, 1H), 5.42 (s, 1H), 4.81 (brs, 1H), 3.71 (d, *J* = 8.5 Hz, 1H), 2.28 (s, 3H), 2.16 (s, 3H); **<u>^{13}C NMR (101 MHz, CDCl$_3$)</u>** δ 163.3, 150.5, 144.1, 143.7, 141.0, 140.3, 138.0, 137.6, 136.6, 135.9, 135.4, 135.1, 133.4, 132.9, 132.5, 131.9, 129.1, 129.0, 128.6, 128.2, 128.2, 128.1, 127.9, 127.5, 127.2, 127.1, 126.7, 126.6, 121.8, 121.6, 120.6, 88.9, 84.6, 63.8, 43.6, 20.1, 19.9; **<u>HRMS (ESI)</u>** calcd for $C_{38}H_{28}N_2O_2$ $[M+H]^+$: 545.2224, Found: 545.2225; **<u>HPLC condition:</u>** The enantiomeric excess was determined by Daicel Chiralcel IA, Hexanes/IPA = 50/50, 1.2 mL/min, λ = 254 nm, t (minor) = 22.780 min, t (major) = 83.052 min.

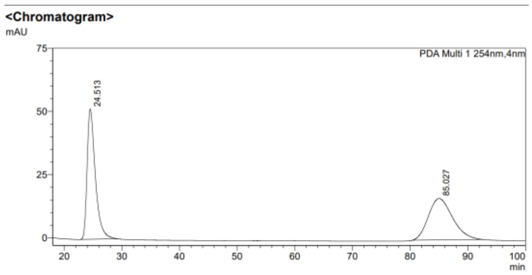

<Peak Table>

PDA Ch1 254nm

Peak#	Ret. Time	Area	Height	Area%
1	24.513	5111091	51485	51.533
2	85.027	4806916	16411	48.467
Total		9918007	67895	100.000

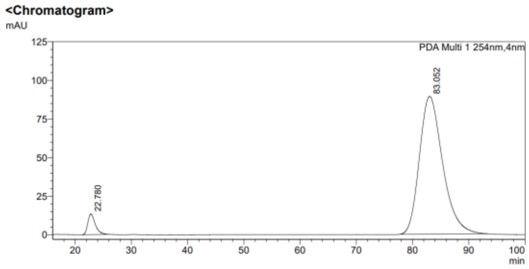

<Peak Table>

PDA Ch1 254nm

Peak#	Ret. Time	Area	Height	Area%
1	22.780	1234572	13396	4.599
2	83.052	25609336	89191	95.401
Total		26843907	102587	100.000

Reaction procedure and characterization of **5g-1**

To a 10 mL Schlenk tube was added **1g-1** (37.4 mg, 0.10 mmol), Ni(OTf)$_2$ (3.6 mg, 10 mol%), (*S*)-**L6** (6.2 mg, 20 mol%), $EtCO_2Ag$ (36.0 mg, 0.20 mmol, 2.0 eq), **P7** (8.5 mg, 20 mol%) and **2c** (40.8 mg, 0.20 mmol, 2.0 eq), followed by addition of anhydrous DME (0.5 mL). Then the mixture was stirred at 100 °C for 24 h. After cooling to room temperature, the reaction system was quenched with aq. $NaHCO_3$ (10 mL) and extracted with CH_2Cl_2 (3×15 mL). The combined organic layer was dried over anhydrous Na_2SO_4, filtered, and concentrated in vacuo. After concentration, the crude product was purified by preparative TLC (PE/EtOAc = 2/1, v/v, R_f = 0.09) to afford **5g-1** (33.0 mg, 57% yield, 87% ee) as a light-yellow foam.

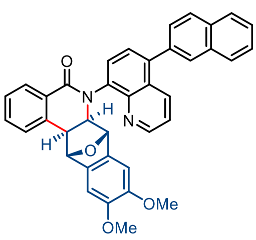

5g-1: $[\alpha]_D^{20}$ = -66.1 (c = 0.85, $CHCl_3$, 87% ee); **^{1}H NMR (400 MHz, $CDCl_3$)** δ 8.82 (brs, 1H), 8.36 (dd, *J* = 8.7, 1.7 Hz, 1H), 8.29 (dd, *J* = 7.9, 1.4 Hz, 1H), 8.11 – 7.90 (m, 5H), 7.80 (brs, 1H), 7.67 (dd, *J* = 8.4, 1.6 Hz, 1H), 7.65 – 7.56 (m, 3H), 7.54 (d, *J* = 7.6 Hz, 1H), 7.42 (t, *J* = 7.6 Hz, 1H), 7.37 (brs, 1H), 7.04 (s, 1H), 6.65 (s, 1H), 5.56 (s, 1H), 5.42 (s, 1H), 4.76 (brs, 1H), 3.93 (s, 3H), 3.75 (s, 3H), 3.68 (d, *J* = 8.5 Hz, 1H); **^{13}C NMR (101 MHz, $CDCl_3$)** δ 163.3, 150.5, 148.7, 148.3, 144.1, 141.0, 138.2, 137.8, 137.5, 136.5, 135.1, 134.4, 133.4, 132.8, 132.5, 131.9, 129.1, 129.0, 128.5, 128.3, 128.2, 128.1, 127.9, 127.5, 127.1, 127.1, 126.7, 126.6, 121.6, 105.0, 103.7, 89.2, 84.9, 63.7, 56.3, 56.3, 43.6; **HRMS (ESI)** calcd for $C_{38}H_{28}N_2O_4$ $[M+H]^+$: 577.2122, Found: 577.2123; **HPLC condition:** The enantiomeric excess was determined by Daicel Chiralcel IA, Hexanes/IPA = 50/50, 1.2 mL/min, λ = 254 nm, t (minor) = 20.321 min, t (major) = 75.715 min.

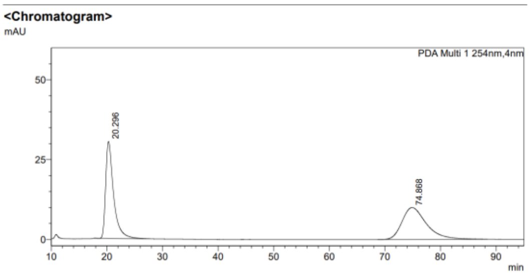

<Peak Table>
PDA Ch1 254nm

Peak#	Ret. Time	Area	Height	Area%
1	20.296	2996091	30340	49.525
2	74.868	3053528	9987	50.475
Total		6049619	40327	100.000

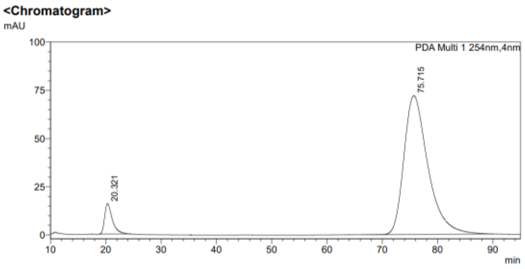

<Peak Table>
PDA Ch1 254nm

Peak#	Ret. Time	Area	Height	Area%
1	20.321	1545669	15807	6.647
2	75.715	21706935	71960	93.353
Total		23252605	87767	100.000

Reaction procedure and characterization of **6g-1**

To a 10 mL Schlenk tube was added **1g-1** (37.4 mg, 0.10 mmol), Ni(OTf)$_2$ (3.6 mg, 10 mol%), (*S*)-**L6** (6.2 mg, 20 mol%), EtCO$_2$Ag (36.0 mg, 0.20 mmol, 2.0 eq), **P7** (8.5 mg, 20 mol%) and **2d** (37.6 mg, 0.20 mmol, 2.0 eq), followed by addition of anhydrous DME (0.5 mL). Then the mixture was stirred at 100 °C for 24 h. After cooling to room temperature, the reaction system was quenched with aq. NaHCO$_3$ (10 mL) and extracted with CH$_2$Cl$_2$ (3×15 mL). The combined organic layer was dried over anhydrous Na$_2$SO$_4$, filtered, and concentrated in vacuo. After concentration, the crude product was purified by preparative TLC (PE/EtOAc = 2/1, v/v, R$_f$ = 0.15) to afford **6g-1** (25.7 mg, 46% yield, 90% ee) as a light-yellow oil.

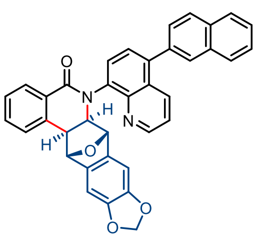

6g-1: $[\alpha]_D^{20}$ = -68.6 (c = 0.44, CHCl$_3$, 90% ee); **<u>^{1}H NMR (400 MHz, CDCl$_3$)</u>** δ 8.82 (brs, 1H), 8.34 (dd, *J* = 8.6, 1.7 Hz, 1H), 8.29 (dd, *J* = 7.9, 1.4 Hz, 1H), 8.08 – 7.90 (m, 5H), 7.78 (brs, 1H), 7.67 (dd, *J* = 8.4, 1.7 Hz, 1H), 7.64 – 7.55 (m, 3H), 7.51 (d, *J* = 7.7 Hz, 1H), 7.42 (t, *J* = 7.5 Hz, 1H), 7.36 (brs, 1H), 6.93 (s, 1H), 6.55 (s, 1H), 5.95 – 5.91 (m, 2H), 5.50 (s, 1H), 5.38 (s, 1H), 4.76 (brs, 1H), 3.67 (d, *J* = 8.5 Hz, 1H); **<u>^{13}C NMR (101 MHz, CDCl$_3$)</u>** δ 163.3, 150.4, 147.2, 146.8, 143.9, 141.1, 139.5, 137.7, 136.5, 136.1, 135.2, 133.4, 132.8, 132.6, 131.9, 129.1, 129.0, 128.5, 128.2, 128.1, 128.1, 127.9, 127.5, 127.2, 127.1, 126.7, 126.6, 121.6, 102.6, 101.5, 101.4, 88.9, 84.7, 63.5, 43.3; **<u>HRMS (ESI)</u>** calcd for $C_{37}H_{24}N_2O_4$ $[M+H]^+$: 561.1809, Found: 561.1812; **<u>HPLC condition:</u>** The enantiomeric excess was determined by Daicel Chiralcel IA, Hexanes/IPA = 50/50, 1.2 mL/min, λ = 254 nm, t (minor) = 40.480 min, t (major) = 83.328 min.

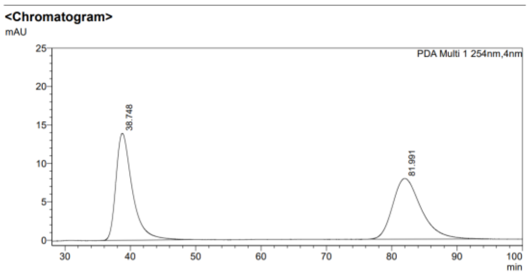

<Peak Table>
PDA Ch1 254nm

Peak#	Ret. Time	Area	Height	Conc.	Area%
1	38.748	2424196	13908	0.000	50.217
2	81.991	2403280	7900	0.000	49.783
Total		4827476	21808		100.000

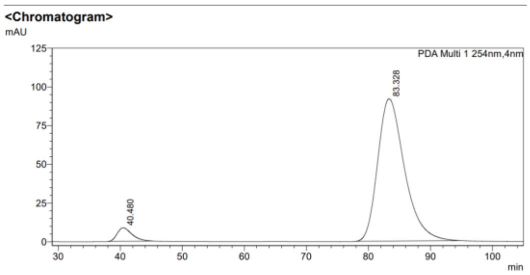

<Peak Table>
PDA Ch1 254nm

Peak#	Ret. Time	Area	Height	Area%
1	40.480	1449622	8741	4.991
2	83.328	27594417	91851	95.009
Total		29044039	100593	100.000

Reaction procedure and characterization of **7g-1**

To a 10 mL Schlenk tube was added **1g-1** (37.4 mg, 0.10 mmol), Ni(OTf)$_2$ (3.6 mg, 10 mol%), (*S*)-**L6** (6.2 mg, 20 mol%), EtCO$_2$Ag (36.0 mg, 0.20 mmol, 2.0 eq), **P7** (8.5 mg, 20 mol%) and **2e** (36.0 mg, 0.20 mmol, 2.0 eq), followed by addition of anhydrous DME (0.5 mL). Then the mixture was stirred at 100 °C for 24 h. After cooling to room temperature, the reaction system was quenched with aq. NaHCO$_3$ (10 mL) and extracted with CH$_2$Cl$_2$ (3×15 mL). The combined organic layer was dried over anhydrous Na$_2$SO$_4$, filtered, and concentrated in vacuo. After concentration, the crude product was purified by preparative TLC (PE/EtOAc = 2/1, v/v, R$_f$ = 0.18) to afford **7g-1** (38.1 mg, 69% yield, 93% ee) as a light-yellow oil.

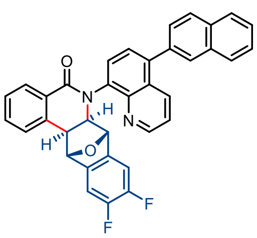

7g-1: $[\alpha]_D^{20}$ = -60.5 (c = 0.84, CHCl$_3$, 93% ee); **^{1}H NMR (400 MHz, CDCl$_3$)** δ 8.82 (brs, 1H), 8.35 (dd, *J* = 8.6, 1.7 Hz, 1H), 8.30 (dd, *J* = 7.9, 1.4 Hz, 1H), 8.08 – 7.91 (m, 5H), 7.78 (brs, 1H), 7.69 – 7.57 (m, 4H), 7.51 (d, *J* = 7.7 Hz, 1H), 7.44 (t, *J* = 7.6 Hz, 1H), 7.38 (brs, 1H), 7.27 (dd, *J* = 8.6, 6.7 Hz, 1H), 6.90 (dd, *J* = 8.8, 6.7 Hz, 1H), 5.59 (s, 1H), 5.45 (s, 1H), 4.82 (brs, 1H), 3.71 (d, *J* = 8.5 Hz, 1H); **^{19}F NMR (376 MHz, CDCl$_3$)** δ -137.74 (d, *J* = 19.1 Hz), -138.40 (d, *J* = 19.0 Hz); **^{13}C NMR (101 MHz, CDCl$_3$)** δ 163.1, 150.6, 149.8 (dd, *J* = 248.6, 13.0 Hz), 149.5 (dd, *J* = 248.0, 13.1 Hz), 143.9, 141.7 (m), 141.3, 138.4, 137.2, 137.0, 136.4, 135.2, 133.4, 132.8, 132.7, 131.8, 129.1, 129.1, 128.6, 128.2, 128.2, 128.1, 128.0, 127.9, 127.5, 127.4, 127.0, 126.7, 126.6, 121.7, 110.7 (d, *J* = 20.0 Hz), 109.4 (d, *J* = 19.8 Hz), 88.4, 84.4, 63.2, 43.0; **HRMS (ESI)** calcd for $C_{36}H_{22}F_2N_2O_2$ $[M+H]^+$: 553.1722, Found: 553.1722; **HPLC condition:** The enantiomeric excess was determined by Daicel Chiralcel IA, Hexanes/IPA = 50/50, 1.2 mL/min, λ = 254 nm, t (minor) = 23.268 min, t (major) = 83.396 min.

<Chromatogram>

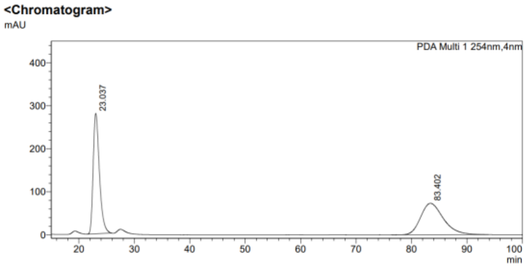

<Peak Table>

PDA Ch1 254nm

Peak#	Ret. Time	Area	Height	Area%
1	23.037	21843713	279855	51.177
2	83.402	20838775	73023	48.823
Total		42682488	352878	100.000

<Chromatogram>

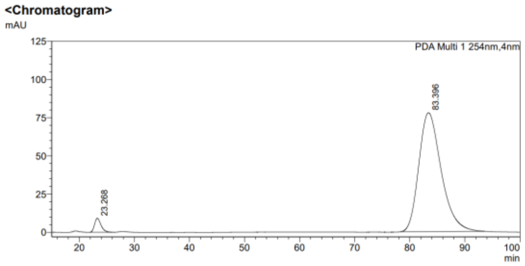

<Peak Table>

PDA Ch1 254nm

Peak#	Ret. Time	Area	Height	Area%
1	23.268	750760	9173	3.308
2	83.396	21944665	77750	96.692
Total		22695425	86922	100.000

Reaction procedure and characterization of **8g-1**

To a 10 mL Schlenk tube was added **1g-1** (37.4 mg, 0.10 mmol), Ni(OTf)$_2$ (3.6 mg, 10 mol%), (*S*)-**L6** (6.2 mg, 20 mol%), EtCO$_2$Ag (36.0 mg, 0.20 mmol, 2.0 eq), **P7** (8.5 mg, 20 mol%) and **2f** (60.0 mg, 0.20 mmol, 2.0 eq), followed by addition of anhydrous DME (0.5 mL). Then the mixture was stirred at 100 °C for 24 h. After cooling to room temperature, the reaction system was quenched with aq. NaHCO$_3$ (10 mL) and extracted with CH$_2$Cl$_2$ (3×15 mL). The combined organic layer was dried over anhydrous Na$_2$SO$_4$, filtered, and concentrated in vacuo. After concentration, the crude product was purified by preparative TLC (PE/EtOAc = 2/1, v/v, R$_f$ = 0.24) to afford **8g-1** (44.5 mg, 66% yield, 94% ee) as a light-yellow foam.

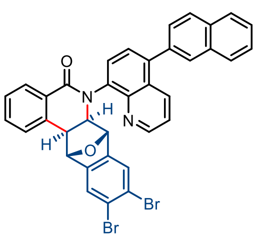

8g-1: $[\alpha]_D^{20}$ = -66.7 (c = 0.88, CHCl$_3$, 94% ee); **^{1}H NMR (400 MHz, CDCl$_3$)** δ 8.82 (brs, 1H), 8.35 (dd, *J* = 8.6, 1.7 Hz, 1H), 8.30 (dd, *J* = 7.8, 1.4 Hz, 1H), 8.08 – 7.91 (m, 5H), 7.78 (brs, 1H), 7.70 (s, 1H), 7.69 – 7.56 (m, 4H), 7.50 (d, *J* = 7.7 Hz, 1H), 7.43 (t, *J* = 7.6 Hz, 1H), 7.37 (brs, 1H), 7.32 (s, 1H), 5.57 (s, 1H), 5.42 (s, 1H), 4.85 (brs, 1H), 3.73 (d, *J* = 8.6 Hz, 1H); **^{13}C NMR (101 MHz, CDCl$_3$)** δ 162.9, 150.6, 146.7, 143.7, 143.5, 141.3, 137.1, 136.9, 136.4, 135.2, 133.4, 132.8, 132.7, 131.8, 129.1, 129.1, 128.6, 128.2, 128.2, 128.1, 128.0, 127.9, 127.5, 127.0, 126.7, 126.6, 125.9, 124.7, 123.7, 123.2, 121.7, 88.1, 84.2, 63.0, 42.8; **HRMS (ESI)** calcd for $C_{36}H_{22}Br_2N_2O_2$ $[M+H]^+$: 673.0121, Found: 673.0123; **HPLC condition:** The enantiomeric excess was determined by Daicel Chiralcel IA, Hexanes/IPA = 50/50, 1.2 mL/min, λ = 254 nm, t (minor) = 24.317 min, t (major) = 97.223 min.

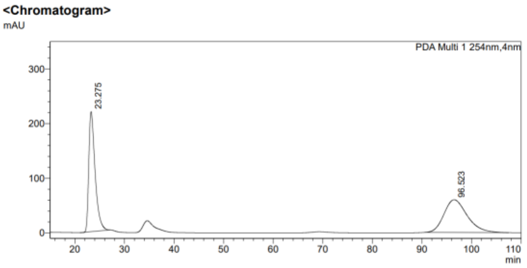

<Peak Table>

PDA Ch1 254nm

Peak#	Ret. Time	Area	Height	Area%
1	23.275	19433536	219468	49.875
2	96.523	19530663	59847	50.125
Total		38964199	279315	100.000

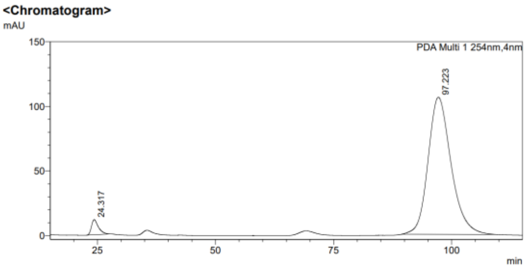

<Peak Table>

PDA Ch1 254nm

Peak#	Ret. Time	Area	Height	Area%
1	24.317	1191718	11703	3.204
2	97.223	35997912	106087	96.796
Total		37189630	117789	100.000

Reaction procedure and characterization of **9g-1**

To a 10 mL Schlenk tube was added **1g-1** (37.4 mg, 0.10 mmol), Ni(OTf)$_2$ (3.6 mg, 10 mol%), (*S*)-**L6** (6.2 mg, 20 mol%), EtCO$_2$Ag (36.0 mg, 0.20 mmol, 2.0 eq), **P7** (8.5 mg, 20 mol%) and **2g** (38.8 mg, 0.20 mmol, 2.0 eq), followed by addition of anhydrous DME (0.5 mL). Then the mixture was stirred at 100 °C for 24 h. After cooling to room temperature, the reaction system was quenched with aq. NaHCO$_3$ (10 mL) and extracted with CH$_2$Cl$_2$ (3×15 mL). The combined organic layer was dried over anhydrous Na$_2$SO$_4$, filtered, and concentrated in vacuo. After concentration, the crude product was purified by preparative TLC (PE/EtOAc = 2/1, v/v, R$_f$ = 0.21) to afford **9g-1** (45.0 mg, 80% yield, 94% ee) as a light-yellow solid.

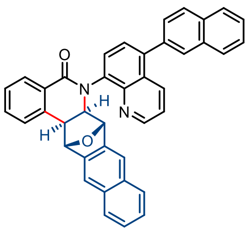

9g-1: $[\alpha]_D^{20}$ = -85.3 (c = 0.89, CHCl$_3$, 94% ee); **^{1}H NMR (400 MHz, CDCl$_3$)** δ 8.79 (brs, 1H), 8.41 – 8.30 (m, 2H), 8.10 (brs, 1H), 8.07 – 7.94 (m, 4H), 7.90 – 7.79 (m, 3H), 7.73 – 7.56 (m, 6H), 7.51 – 7.42 (m, 4H), 7.35 (brs, 1H), 5.74 (s, 1H), 5.62 (s, 1H), 4.99 (brs, 1H), 3.86 (d, *J* = 8.6 Hz, 1H); **^{13}C NMR (101 MHz, CDCl$_3$)** δ 163.2, 150.5, 144.0, 143.3, 141.1, 140.1, 137.5, 137.4, 136.5, 135.1, 133.4, 133.1, 132.8, 132.8, 132.6, 131.9, 129.1, 129.0, 128.6, 128.3, 128.2, 128.2, 128.1, 128.1, 127.9, 127.5, 127.3, 127.1, 126.7, 126.6, 126.4, 126.2, 121.6, 119.2, 117.6, 88.8, 84.6, 64.1, 44.2; **HRMS (ESI)** calcd for $C_{40}H_{26}N_2O_2$ $[M+H]^+$: 567.2067, Found: 567.2070; **HPLC condition:** The enantiomeric excess was determined by Daicel Chiralcel IA, Hexanes/IPA = 50/50, 1.2 mL/min, λ = 254 nm, t (minor) = 40.997 min, t (major) = 108.280 min.

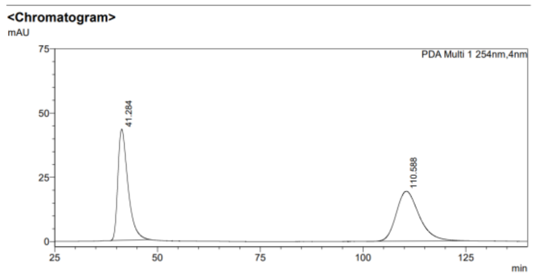

<Peak Table>
PDA Ch1 254nm

Peak#	Ret. Time	Area	Height	Area%
1	41.284	7383891	43259	49.398
2	110.588	7563971	19418	50.602
Total		14947862	62677	100.000

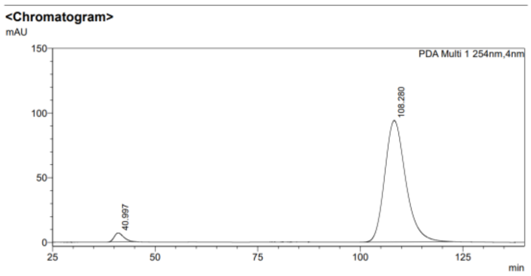

<Peak Table>
PDA Ch1 254nm

Peak#	Ret. Time	Area	Height	Area%
1	40.997	1114435	6890	3.230
2	108.280	33383430	93976	96.770
Total		34497865	100867	100.000

3.3 Gram-scale synthesis and post-functionalization

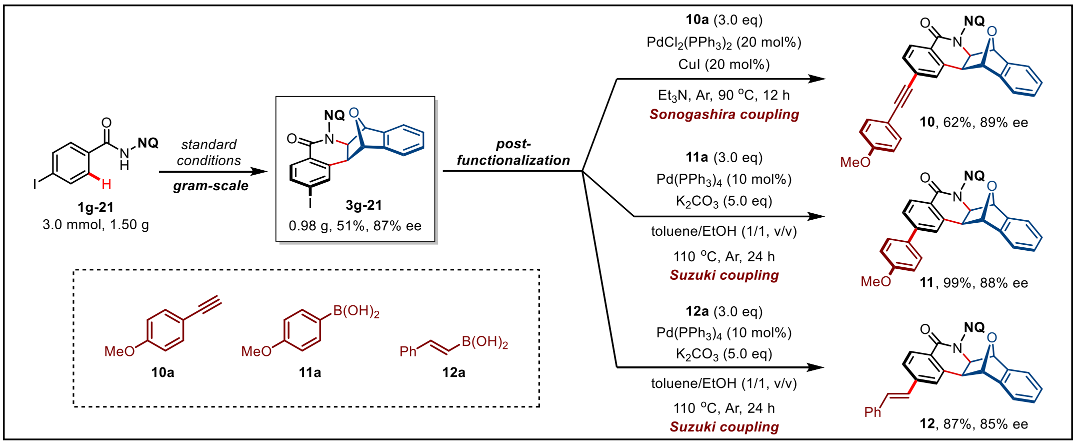

<u>Gram-scale synthesis of **3g-21**</u>

To a 250 mL Schlenk tube was added **1g-21** (1.50 g, 3.0 mmol), $Ni(OTf)_2$ (107.1 mg, 10 mol%), (*S*)-**L6** (186.6 mg, 20 mol%), $EtCO_2Ag$ (1.08 g, 6.0 mmol, 2.0 eq), **P7** (255 mg, 20 mol%) and **2a** (864 mg, 6.0 mmol, 2.0 eq), followed by addition of anhydrous DME (15 mL). Then the mixture was stirred at 100 °C for 24 h. After cooling to room temperature, the reaction system was quenched with aq. $NaHCO_3$ (100 mL) and extracted with CH_2Cl_2 (3×40 mL). The combined organic layer was dried over anhydrous Na_2SO_4, filtered, and concentrated in vacuo. After concentration, the crude product was purified by flash chromatography on silica gel in PE/EtOAc = 2/1 to afford **3g-21** (0.98 g, 51% yield, 87% ee).

Reaction procedure and characterization of **10**

To a 10 mL Schlenk tube was added **3g-21** (64.2 mg, 0.10 mmol), $PdCl_2(PPh_3)_2$ (14.0 mg, 20 mol%), CuI (3.8 mg, 20 mol%) and 4-ethynylanisole (39.6 mg, 0.30 mmol, 3.0 eq), followed by addition of Et_3N (0.5 mL). Then the mixture was stirred at 90 °C for 12 h under Ar atmosphere. After cooling to room temperature, the reaction system was quenched with aq. water (10 mL) and extracted with EtOAc (3×15 mL). The combined organic layer was dried over anhydrous Na_2SO_4, filtered, and concentrated in vacuo. After concentration, the crude product was purified by preparative TLC (PE/DCM/acetone = 50/50/1, v/v, R_f = 0.39) to afford **10** (40.0 mg, 62% yield, 89% ee) as a light-yellow oil.

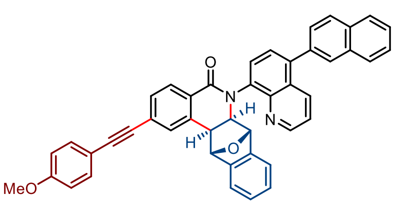

10: $[\alpha]_D^{20}$ = 52.4 (c = 1.06, $CHCl_3$, 89% ee); **^{1}H NMR (400 MHz, $CDCl_3$)** δ 8.82 (brs, 1H), 8.35 (dd, *J* = 8.6, 1.7 Hz, 1H), 8.28 (d, *J* = 8.1 Hz, 1H), 8.09 – 7.90 (m, 5H), 7.80 (brs, 1H), 7.73 – 7.64 (m, 2H), 7.63 – 7.52 (m, 5H), 7.45 (d, *J* = 7.3 Hz, 1H), 7.36 (brs, 1H), 7.29 – 7.21 (m, 1H), 7.13 (t, *J* = 7.4 Hz, 1H), 7.06 (d, *J* = 7.3 Hz, 1H), 6.94 (d, *J* = 8.8 Hz, 2H), 5.63 (s, 1H), 5.53 (s, 1H), 4.85 (brs, 1H), 3.86 (s, 3H), 3.72 (d, *J* = 8.5 Hz, 1H); **^{13}C NMR (101 MHz, $CDCl_3$)** δ 162.8, 160.0, 150.5, 145.7, 143.9, 142.4, 141.1, 137.8, 137.3, 136.5, 135.1, 133.4, 133.3, 132.8, 131.8, 130.9, 130.1, 129.1, 129.0, 128.5, 128.2, 128.1, 127.9, 127.8, 127.7, 127.5, 127.3, 126.7, 126.6, 126.3, 121.6, 120.6, 119.3, 114.9, 114.2, 92.2, 88.8, 87.8, 84.7, 63.5, 55.4, 42.9; **HRMS (ESI)** calcd for $C_{45}H_{30}N_2O_3$ $[M+H]^+$: 647.2329, Found: 647.2329; **HPLC condition:** The enantiomeric excess was determined by Daicel Chiralcel IA, Hexanes/IPA = 50/50, 1.7 mL/min, λ = 254 nm, t (minor) = 12.202 min, t (major) = 69.681 min.

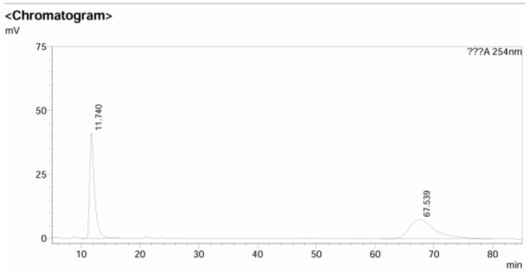

<Peak Table>
???A 254nm

Peak#	Ret. Time	Area	Height	Area%
1	11.740	2347376	40942	50.578
2	67.539	2293690	7535	49.422
Total		4641066	48477	100.000

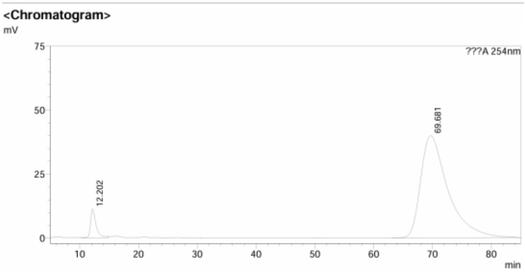

<Peak Table>
???A 254nm

Peak#	Ret. Time	Area	Height	Area%
1	12.202	738764	11201	5.391
2	69.681	12965268	39883	94.609
Total		13704033	51084	100.000

Reaction procedure and characterization of **11**

To a 10 mL Schlenk tube was added **3g-21** (64.2 mg, 0.10 mmol), $Pd(PPh_3)_4$ (11.6 mg, 10 mol%), K_2CO_3 (69.0 mg, 0.50 mmol, 5.0 eq) and 4-methoxyphenylboronic acid (45.6 mg, 0.30 mmol, 3.0 eq), followed by addition of toluene/EtOH (0.25 mL/0.25 mL). Then the mixture was stirred at 110 °C for 24 h under Ar atmosphere. After cooling to room temperature, the reaction system was quenched with aq. water (10 mL) and extracted with EtOAc (3×15 mL). The combined organic layer was dried over anhydrous Na_2SO_4, filtered, and concentrated in vacuo. After concentration, the crude product was purified by preparative TLC (PE/DCM/acetone = 50/50/1, v/v, R_f = 0.36) to afford **11** (61.6 mg, 99% yield, 88% ee) as a colorless oil.

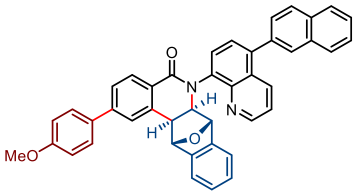

11: $[\alpha]_D^{20}$ = 25.4 (c = 0.90, $CHCl_3$, 88% ee); **^{1}H NMR (400 MHz, $CDCl_3$)** δ 8.81 (brs, 1H), 8.35 (d, *J* = 8.4 Hz, 2H), 8.14 – 7.91 (m, 5H), 7.80 (brs, 1H), 7.67 (d, *J* = 8.9 Hz, 4H), 7.62 – 7.59 (m, 3H), 7.46 (d, *J* = 7.3 Hz, 1H), 7.35 (brs, 1H), 7.28 – 7.21 (m, 1H), 7.12 (t, *J* = 7.3 Hz, 1H), 7.07 (d, *J* = 7.1 Hz, 3H), 5.64 (s, 1H), 5.54 (s, 1H), 4.85 (brs, 1H), 3.90 (s, 3H), 3.78 (d, *J* = 8.5 Hz, 1H); **^{13}C NMR (101 MHz, $CDCl_3$)** δ 163.3, 159.9, 150.5, 145.9, 145.1, 144.1, 142.6, 141.1, 138.2, 137.6, 136.6, 135.1, 133.4, 132.9, 132.8, 131.9, 129.6, 129.2, 128.5, 128.2, 128.2, 127.9, 127.7, 127.5, 127.2, 126.7, 126.6, 126.2, 125.7, 125.6, 121.6, 120.6, 119.3, 114.5, 88.9, 84.8, 63.6, 55.5, 43.3; **HRMS (ESI)** calcd for $C_{43}H_{30}N_2O_3$ $[M+H]^+$: 623.2331, Found: 623.2329; **HPLC condition:** The enantiomeric excess was determined by Daicel Chiralcel IB N-5, Hexanes/IPA = 50/50, 1.7 mL/min, λ = 254 nm, t (minor) = 20.693 min, t (major) = 47.971 min.

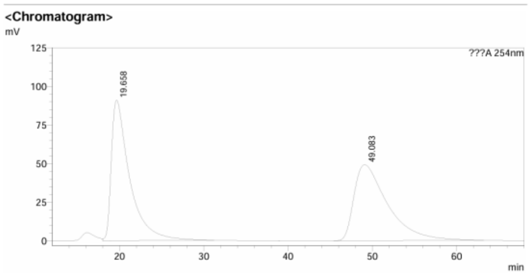

<Peak Table>

???A 254nm

Peak#	Ret. Time	Area	Height	Area%
1	19.658	13727989	90906	50.083
2	49.083	13682644	49212	49.917
Total		27410633	140118	100.000

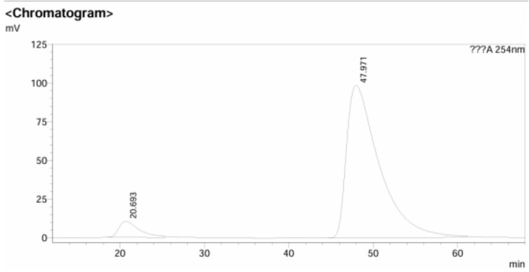

<Peak Table>

???A 254nm

Peak#	Ret. Time	Area	Height	Area%
1	20.693	1754469	10454	6.187
2	47.971	26602388	98425	93.813
Total		28356857	108879	100.000

Reaction procedure and characterization of **12**

To a 10 mL Schlenk tube was added **3g-21** (64.2 mg, 0.10 mmol), $Pd(PPh_3)_4$ (11.6 mg, 10 mol%), K_2CO_3 (69.0 mg, 0.50 mmol, 5.0 eq) and *E*-phenylethenylboronic acid (44.4 mg, 0.30 mmol, 3.0 eq), followed by addition of toluene/EtOH (0.25 mL/0.25 mL). Then the mixture was stirred at 110 °C for 24 h under Ar atmosphere. After cooling to room temperature, the reaction system was quenched with aq. water (10 mL) and extracted with EtOAc (3×15 mL). The combined organic layer was dried over anhydrous Na_2SO_4, filtered, and concentrated in vacuo. After concentration, the crude product was purified by preparative TLC (PE/DCM/acetone = 50/50/1, v/v, R_f = 0.42) to afford **12** (53.8 mg, 87% yield, 85% ee) as a white foam.

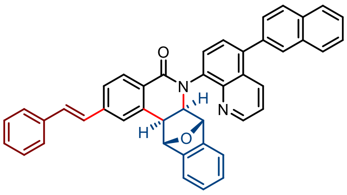

12: $[\alpha]_D^{20}$ = 45.7 (c = 1.00, $CHCl_3$, 85% ee); **^{1}H NMR (400 MHz, $CDCl_3$)** δ 8.79 (brs, 1H), 8.32 (d, *J* = 8.6 Hz, 1H), 8.27 (d, *J* = 8.1 Hz, 1H), 8.07 – 7.90 (m, 5H), 7.77 (brs, 1H), 7.65 (d, *J* = 8.5 Hz, 1H), 7.61 – 7.55 (m, 6H), 7.46 (d, *J* = 7.3 Hz, 1H), 7.41 (t, *J* = 7.5 Hz, 2H), 7.36 – 7.19 (m, 5H), 7.11 (t, *J* = 7.4 Hz, 1H), 7.04 (d, *J* = 7.3 Hz, 1H), 5.61 (s, 1H), 5.53 (s, 1H), 4.82 (brs, 1H), 3.72 (d, *J* = 8.5 Hz, 1H); **^{13}C NMR (101 MHz, $CDCl_3$)** δ 163.1, 150.5, 145.9, 144.1, 142.6, 141.5, 141.1, 138.2, 137.6, 136.9, 136.6, 135.1, 133.4, 132.9, 131.9, 131.0, 129.5, 129.2, 128.9, 128.6, 128.2, 128.2, 127.9, 127.8, 127.7, 127.6, 127.2, 126.9, 126.7, 126.6, 126.3, 125.1, 121.6, 120.6, 119.3, 88.9, 84.8, 63.6, 43.2; **HRMS (ESI)** calcd for $C_{44}H_{30}N_2O_2$ $[M+H]^+$: 619.2381, Found: 619.2380; **HPLC condition:** The enantiomeric excess was determined by Daicel Chiralcel IA, Hexanes/IPA = 50/50, 1.7 mL/min, λ = 254 nm, t (minor) = 11.379 min, t (major) = 116.710 min.

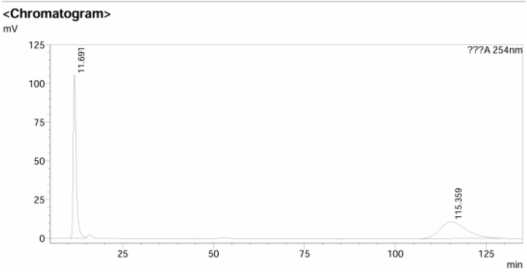

<Peak Table>
???A 254nm

Peak#	Ret. Time	Area	Height	Area%
1	11.691	5957728	105479	51.935
2	115.359	5513747	10757	48.065
Total		11471475	116237	100.000

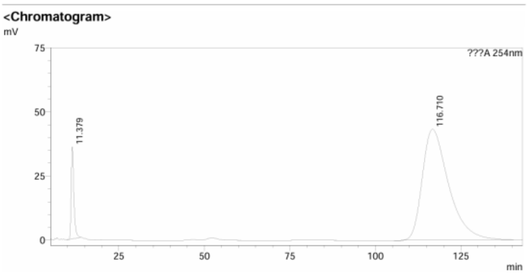

<Peak Table>
???A 254nm

Peak#	Ret. Time	Area	Height	Area%
1	11.379	1915248	35875	7.610
2	116.710	23250746	43212	92.390
Total		25165994	79087	100.000

4. Mechanistic Studies

4.1 Experiments of nonlinear effects (NLEs)

To several different 10 mL Schlenk tubes, **1g-1** (37.4 mg, 0.10 mmol), $Ni(OTf)_2$ (3.6 mg, 10 mol%), (*S*)-**L6** with different ee value (6.2 mg, 20 mol%), $EtCO_2Ag$ (36.0 mg, 0.20 mmol, 2.0 eq), **P7** (8.5 mg, 20 mol%) and **2a** (28.8 mg, 0.20 mmol, 2.0 eq) were added, followed by addition of anhydrous DME (0.5 mL). Then each tube with the mixture was stirred at 100 °C for 24 h. After cooling to room temperature, the reaction system was quenched with aq. $NaHCO_3$ (10 mL) and extracted with CH_2Cl_2 (3×15 mL). The combined organic layer was dried over anhydrous Na_2SO_4, filtered, and concentrated in vacuo. After concentration, the crude product was purified by preparative TLC (PE/EtOAc = 2/1, v/v) to afford **3g-1**. The ee values of **3g-1** were determined by chiral HPLC.

Ee value of (*S*)-**L6** (%)	Ee value of **3g-1** (%)
-1.50	-0.59
12.36	11.75
26.03	24.58
40.58	37.38
55.29	50.99
71.29	65.14
84.71	78.01

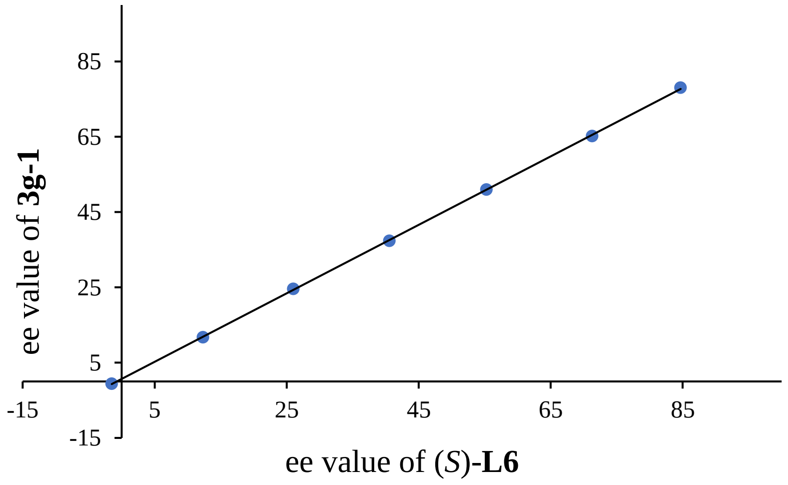

4.2 Deuterium-labeling studies

4.2.1 Synthesis of **1g-1-*d*$_5$**

To a mixture of benzoic-2,3,4,5,6-*d*$_5$ acid (3.0 mmol) and DMF (*cat.*, several drops) in anhydrous DCM (15 mL) was slowly added oxalyl chloride (381 μL, 4.5 mmol, 1.5 eq) at 0 °C, and stirred at room temperature for 3 hours. After the reaction was completed, the solvent was removed in vacuo and the resulting benzoyl-*d*$_5$ chloride was used immediately without further purification.

To an oven-dried 50 mL three-necked flask, 8-aminoquinoline (**NQ**, 405 mg, 1.5 mmol, 0.5 eq), Et_3N (835 μL, 6.0 mmol, 2.0 eq) and anhydrous DCM (15 mL) were added. A solution of above crude benzoyl-*d*$_5$ chloride in anhydrous DCM (10 mL) was added dropwise to the mixture at 0 °C, and the solution was stirred at room temperature for overnight. After the reaction was completed, the reaction mixture was quenched with $NaHCO_3$ (50 mL, sat. aq.) and extracted with DCM (3×20 mL). The combined organic layer was washed with brine, dried over anhydrous Na_2SO_4, and concentrated in vacuo. The resulting residue was purified by flash chromatography on silica gel in PE/EtOAc to afford the desired amides **1g-1-*d*$_5$** with 98% D.

1g-1-*d*$_5$: **^{1}H NMR (400 MHz, $CDCl_3$)** δ 10.89 (s, 1H), 9.04 (d, *J* = 7.9 Hz, 1H), 8.93 – 8.86 (m, 1H), 8.36 (d, *J* = 8.5 Hz, 1H), 8.14 (s, 0.04H), 7.99 – 7.90 (m, 4H), 7.68 (d, *J* = 8.0 Hz, 1H), 7.64 – 7.53 (m, 3H), 7.45 (dd, *J* = 8.6, 4.3 Hz, 1H).

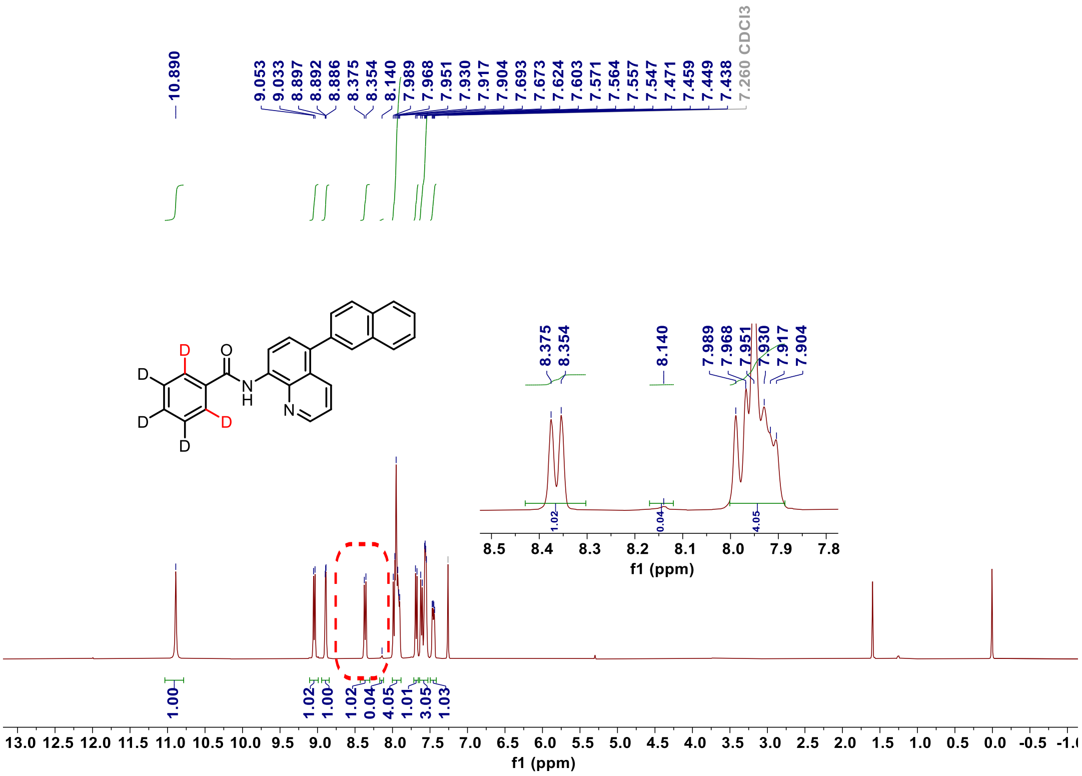

4.2.2 H/D exchange experiments with **1g-1-*d*$_5$**

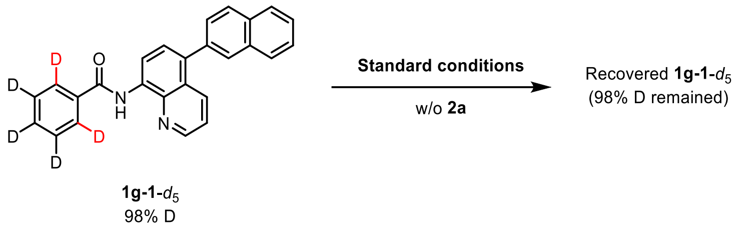

To a 10 mL Schlenk tube was added **1g-1-*d*$_5$** (37.9 mg, 0.10 mmol), $Ni(OTf)_2$ (3.6 mg, 10 mol%), (*S*)-**L6** (6.2 mg, 20 mol%), $EtCO_2Ag$ (36.0 mg, 0.20 mmol, 2.0 eq) and **P7** (8.5 mg, 20 mol%), followed by addition of anhydrous DME (0.5 mL). Then the tube with the mixture was stirred at 100 °C for 24 h. After cooling to room temperature, the reaction system was quenched with aq. $NaHCO_3$ (10 mL) and extracted with CH_2Cl_2 (3×15 mL). The combined organic layer was dried over anhydrous Na_2SO_4, filtered, and concentrated in vacuo. After concentration, the crude product was purified by preparative TLC (PE/EtOAc = 10/1, v/v) to afford the recovered starting material **1g-1-*d*$_5$**. Analysis by ^{1}H NMR, 98% D deuterated ratio of starting material **1g-1-*d*$_5$** was maintained.

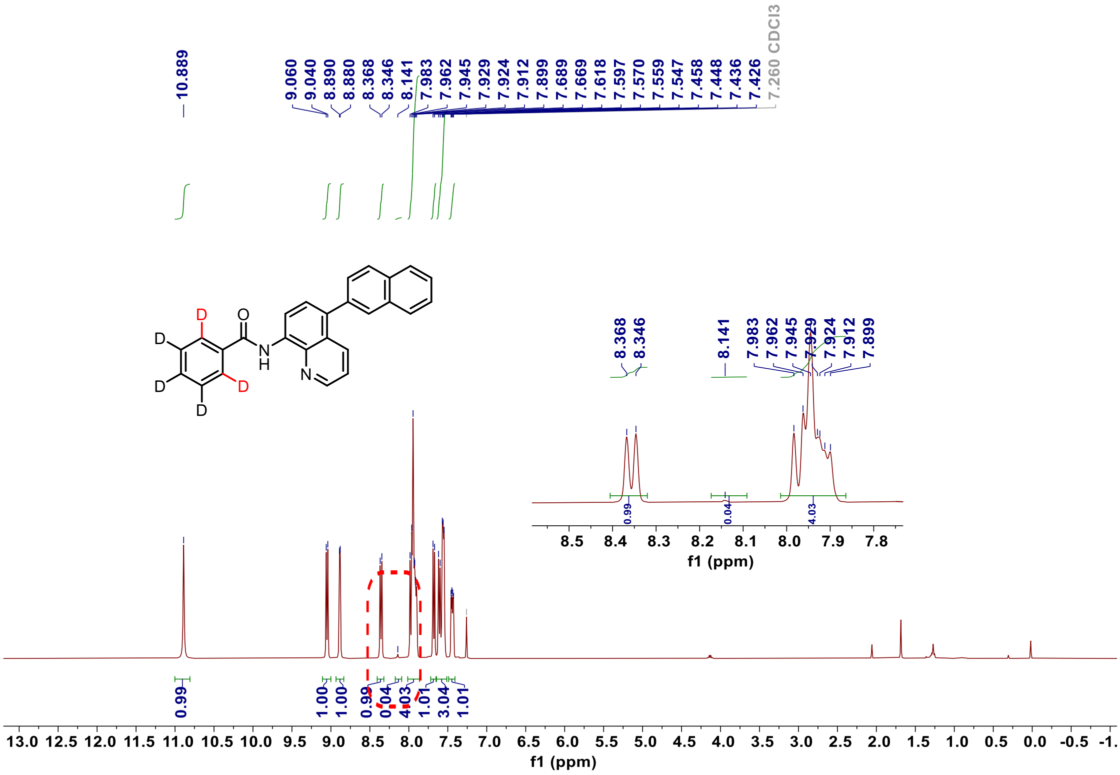

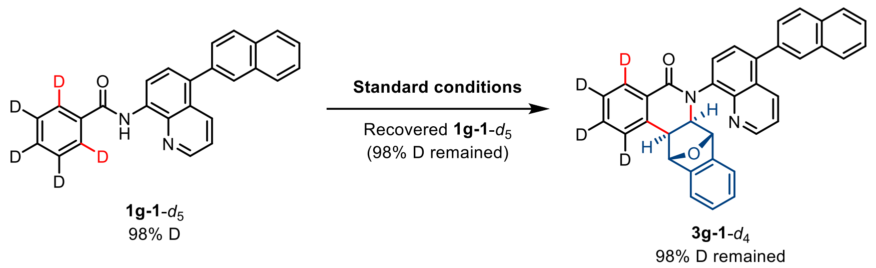

To a 10 mL Schlenk tube was added **1g-1-*d*$_5$** (37.9 mg, 0.10 mmol), Ni(OTf)$_2$ (3.6 mg, 10 mol%), (*S*)-**L6** (6.2 mg, 20 mol%), $EtCO_2Ag$ (36.0 mg, 0.20 mmol, 2.0 eq) and **P7** (8.5 mg, 20 mol%), followed by addition of anhydrous DME (0.5 mL). Then the tube with the mixture was stirred at 100 °C for 24 h. After cooling to room temperature, the reaction system was quenched with aq. $NaHCO_3$ (10 mL) and extracted with CH_2Cl_2 (3×15 mL). The combined organic layer was dried over anhydrous Na_2SO_4, filtered, and concentrated in vacuo. After concentration, the crude product was purified by preparative TLC (PE/EtOAc = 10/1 to 2/1, v/v) to afford the recovered starting material **1g-1-*d*$_5$** and product **3g-1-*d*$_4$**. Analysis by ^{1}H NMR, 98% D deuterated ratio of starting material **1g-1-*d*$_5$** and product **3g-1-*d*$_4$** were maintained.

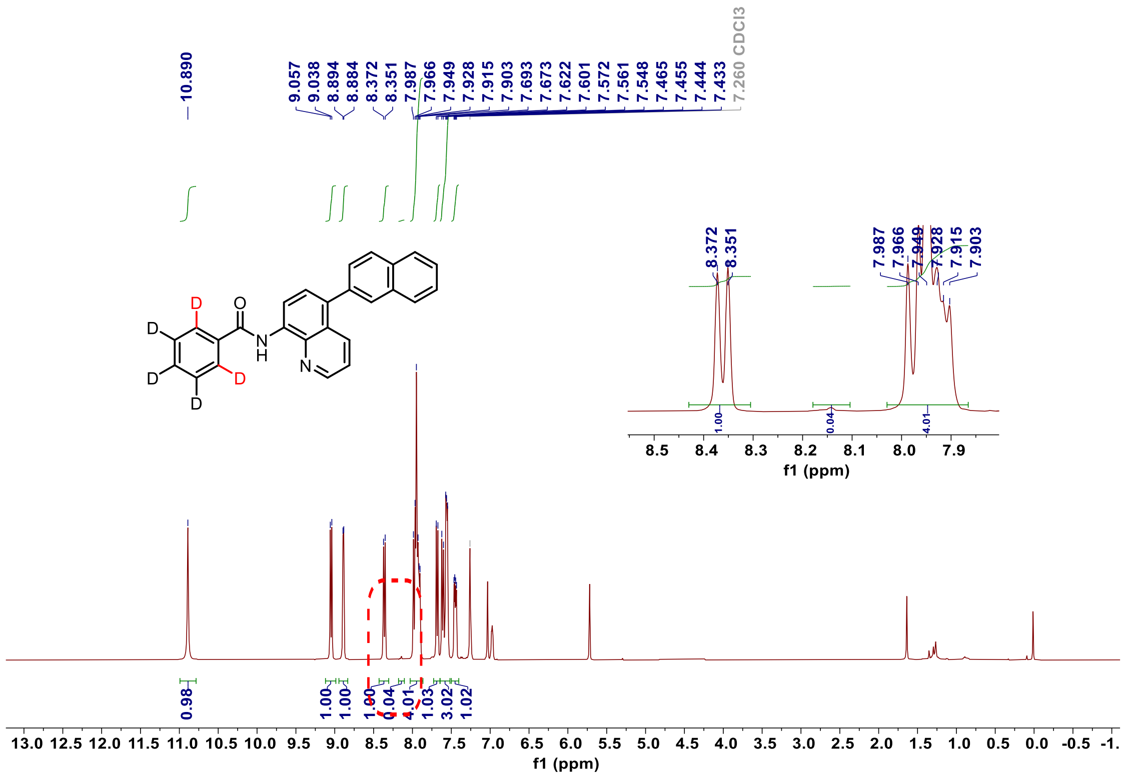

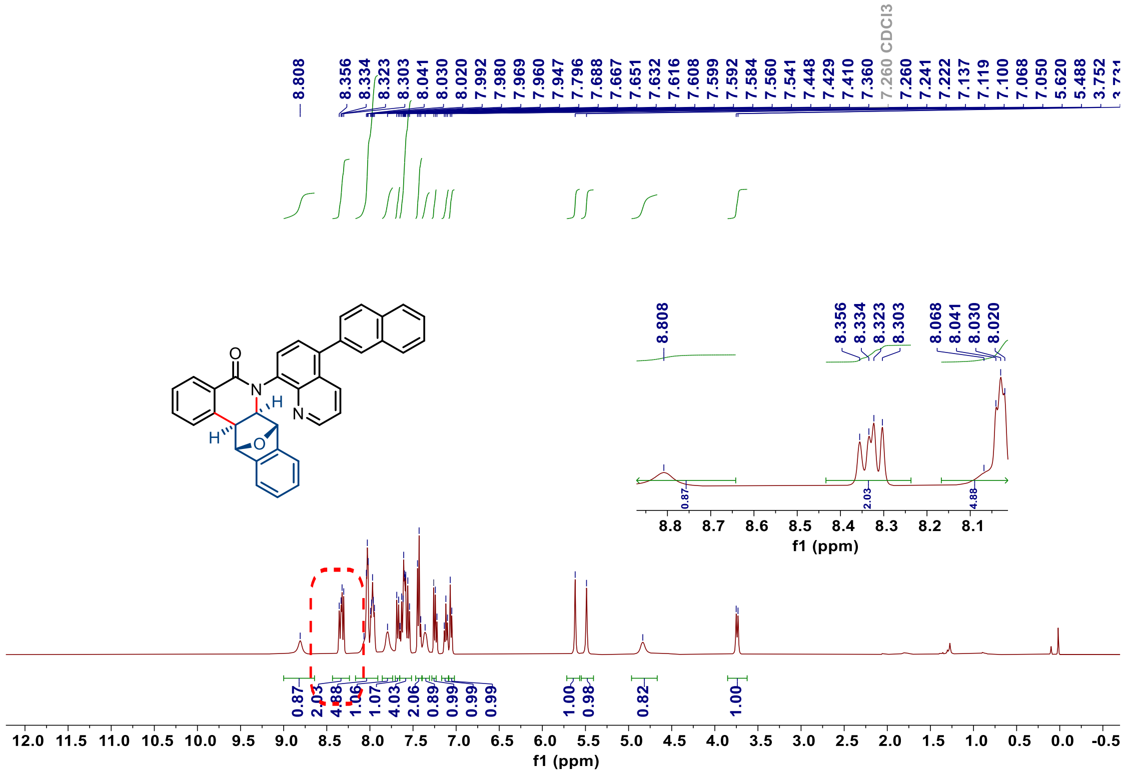
7.260 CDCl3
f1 (ppm)

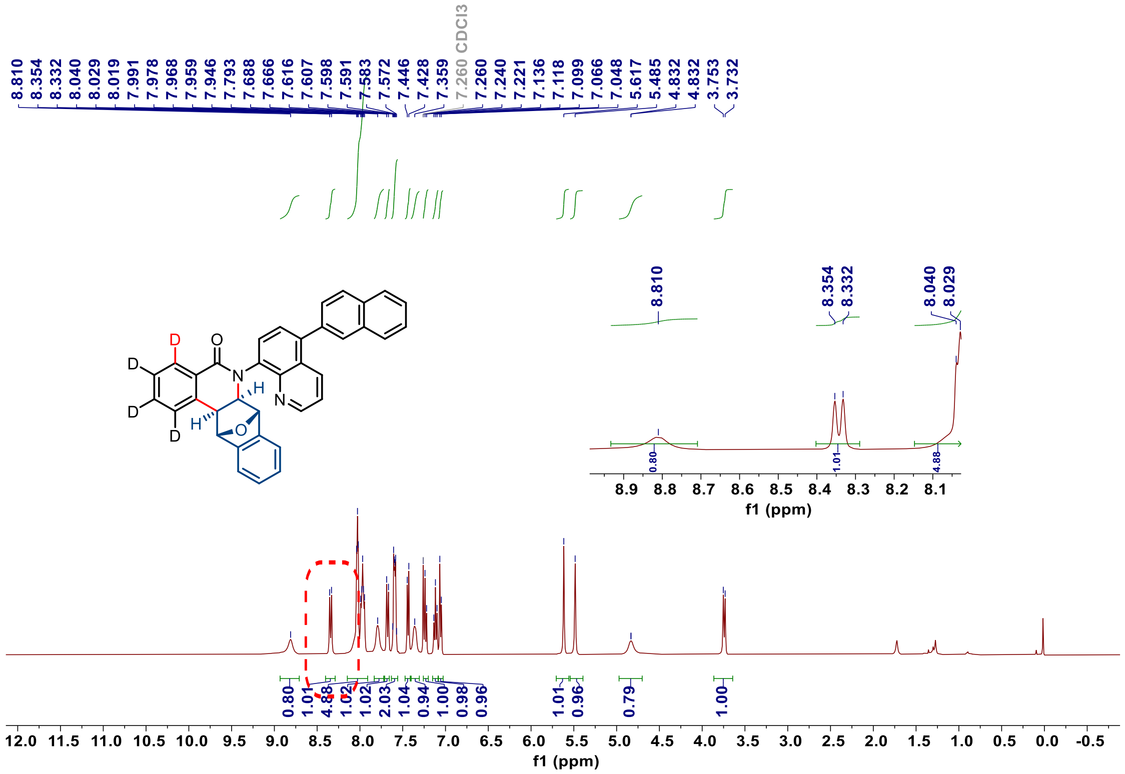
7.260 CDCl3
f1 (ppm)

4.3 Parallel KIE experiments with 1g-1 and 1g-1-d_5

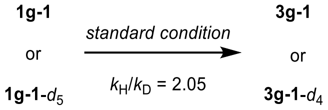

To a 10 mL Schlenk tube was added **1g-1** (37.4 mg, 0.10 mmol), $Ni(OTf)_2$ (3.6 mg, 10 mol%), (*S*)-**L6** (6.2 mg, 20 mol%), $EtCO_2Ag$ (36.0 mg, 0.20 mmol, 2.0 eq) and **P7** (8.5 mg, 20 mol%), followed by addition of anhydrous DME (0.5 mL). In another 10 mL Schlenk tube, **1g-1**-d_5 (37.9 mg, 0.10 mmol) was used instead of **1g-1**. Then each tube with the mixture was stirred at 100 °C for 1 hour. After cooling to room temperature, every reaction system was quenched with aq. $NaHCO_3$ (10 mL) and extracted with CH_2Cl_2 (3×15 mL), separately. The combined organic layer was dried over anhydrous Na_2SO_4, filtered, and concentrated in vacuo. After concentration, the two products (**3g-1**, 15.0% yield; **3g-1**-d_4, 7.33% yield) were analyzed by ^{1}H NMR, using 1,3,5-trimethoxybenzene as internal standard. The value of K_H/K_D was obtained based on yields.

4.4 Synthesis and Characterization of C-Ni(II) and C-Ni(III) intermediates

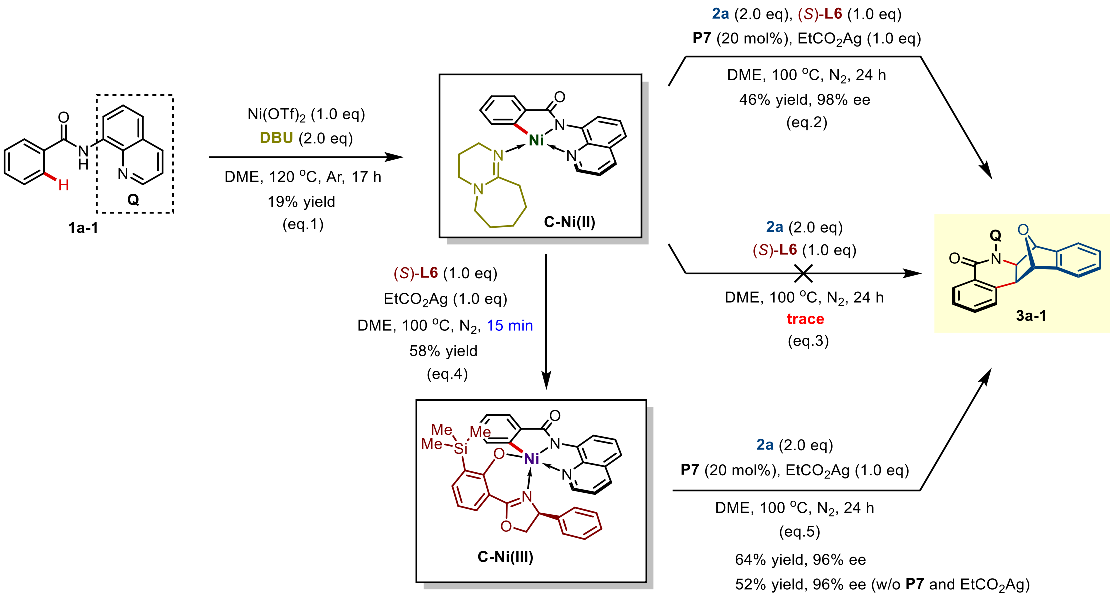

4.4.1 Synthesis and characterization of **C-Ni(II)** (eq. 1)

To an oven-dried 10 mL Schlenk tube was added **1a-1** (248.0 mg, 1.0 mmol), $Ni(OTf)_2$ (356.0 mg, 1.0 eq), 1,8-Diazabicyclo[5.4.0]undecane-7-ene (DBU, 298.8 μL, 2.0 eq) and anhydrous DME (4.0 mL). Then the mixture was stirred at 120 °C for 17 hours under Ar atmosphere. After cooling to room temperature, the reaction system was concentrated in vacuo and the residue was purified by flash chromatography on silica gel in PE/EtOAc = 4/1 to EtOAc to afford the desired **C-Ni(II)** intermediate (85.3 mg, 19% yield) as orange red foam.

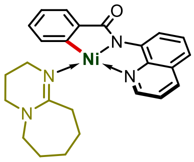

C-Ni(II): **^{1}H NMR (400 MHz, $CDCl_3$)** δ 8.90 (d, J = 7.9 Hz, 1H), 8.12 (d, J = 8.4 Hz, 1H), 7.76 (d, J = 4.8 Hz, 1H), 7.50 – 7.39 (m, 2H), 7.28 – 7.25 (m, 1H), 7.14 (d, J = 8.1 Hz, 1H), 7.07 – 6.96 (m, 2H), 6.59 (d, J = 7.0 Hz, 1H), 4.01 – 3.95 (m, 1H), 3.92 – 3.79 (m, 2H), 3.72 – 3.65 (m, 1H), 3.46 – 3.33 (m, 4H), 2.07 – 1.88 (m, 2H), 1.84 – 1.52 (m, 6H); **^{13}C NMR (101 MHz, $CDCl_3$)** δ 177.6, 164.6, 148.6, 148.1, 147.3, 145.8, 144.4, 137.4, 133.7, 129.8, 129.5, 128.9, 125.2, 124.1, 120.9, 118.6, 115.9, 54.3, 47.9, 46.9, 40.0, 29.4, 28.4, 24.7, 22.6; **HRMS (ESI)** calcd for $C_{25}H_{26}N_4NiO$ $[M+H]^+$: 457.1533, Found: 457.1535.

4.4.2 Stoichiometric reaction of **C-Ni(II)** (eq. 2 and eq. 3)

To an oven-dried 10 mL Schlenk tube was added **C-Ni(II)** (22.8 mg, 0.05 mmol), **2a** (14.4 mg, 2.0 eq), (*S*)-**L6** (15.5 mg, 1.0 eq), **P7** (4.3 mg, 20 mol%), $EtCO_2Ag$ (9.0 mg, 1.0 eq) and anhydrous DME (0.25 mL). Then the mixture was stirred at 100 °C for 24 h under N_2 atmosphere. After cooling to room temperature, the reaction system was quenched with aq. $NaHCO_3$ (10 mL) and extracted with CH_2Cl_2 (3×15 mL). The combined organic layer was dried over anhydrous Na_2SO_4, filtered, and concentrated in vacuo. After concentration, the residue was analyzed by ^1H NMR, using 1,3,5-trimethoxybenzene as internal standard. Product **3a-1** was obtained in 46% yield with 98% ee (eq. 2). However, only trace product **3a-1** was observed in the absent of $EtCO_2Ag$ (eq. 3).

4.4.3 Synthesis and characterization of **C-Ni(III)** (eq. 4)

To an oven-dried 10 mL Schlenk tube was added **C-Ni(II)** (22.8 mg, 0.05 mmol), (*S*)-**L6** (15.5 mg, 1.0 eq), $EtCO_2Ag$ (9.0 mg, 1.0 eq) and anhydrous DME (0.5 mL). Then the mixture was stirred at 100 °C for 15 minutes under N_2 atmosphere. After cooling to room temperature, the reaction system was concentrated in vacuo and the residue was purified by flash chromatography on silica gel in PE/acetone = 4/1 to afford the desired **C-Ni(III)** intermediate (17.9 mg, 58% yield) as black red solid. **HRMS (ESI)** calcd for $C_{34}H_{30}N_3NiO_3Si$ $[M+H]^+$: 615.1483, Found: 615.1486. **Elemental analysis** calcd for $C_{34}H_{30}N_3NiO_3Si$: C: 66.36, H: 4.91, N: 6.83, Found: C: 65.10, H: 4.99, N: 6.65.

4.4.4 Stoichiometric reaction of **C-Ni(III)** (eq. 5)

To an oven-dried 10 mL Schlenk tube was added **C-Ni(III)** (30.7 mg, 0.05 mmol), **2a** (14.4 mg, 2.0 eq), **P7** (4.3 mg, 20 mol%), $EtCO_2Ag$ (9.0 mg, 1.0 eq) and anhydrous DME (0.25 mL). Then the mixture was stirred at 100 °C for 24 h under N_2 atmosphere. After cooling to room temperature, the reaction system was quenched with aq. $NaHCO_3$ (10 mL) and extracted with CH_2Cl_2 (3×15 mL). The combined organic layer was dried over anhydrous Na_2SO_4, filtered, and concentrated in vacuo. After concentration, the residue was analyzed by ^1H NMR, using 1,3,5-trimethoxybenzene as internal standard. Product **3a-1** was obtained in 64% yield with 96% ee. Meanwhile, product **3a-1** obtained in 54% yield with 96% ee in the absent of **P7** and $EtCO_2Ag$.

References

[1] Whiteoak, C. J.; Planas, O.; Company, A.; Ribas, X. A First Example of Cobalt-Catalyzed Remote C-H Functionalization of 8-Aminoquinolines Operating through a Single Electron Transfer Mechanism. *Adv. Synth. Catal.* **2016**, *358*, 1679.

[2] Chen, C.; Zeng, R.; Zhang, J.; Zhao, Y. Ruthenium-Catalyzed Difluoroalkylation of 8-Aminoquinoline Amides at the C5-Position. *Eur. J. Org. Chem.* **2017**, *2017*, 6947.

[3] Li, Y.; Zhu, L.; Cao, X.; Au, C.-T.; Qiu, R.; Yin, S.-F. Metal–free C5-H Bromination of Quinolines for One-pot C−X (X=C, O, S) Bond Formations. *Adv. Synth. Catal.* **2017**, *359*, 2864.

[4] Li, Y.; Zhang, P.; Liu, Y.-J.; Yu, Z.-X.; Shi, B.-F. Remote γ-C(sp3)–H Alkylation of Aliphatic Carboxamides via an Unexpected Regiodetermining Pd Migration Process: Reaction Development and Mechanistic Study. *ACS Catal.* **2020**, *10*, 8212.

[5] Qiu, S.; Zhai, S.; Wang, H.; Chen, X.; Zhai, H. One-pot synthesis of benzo[b]fluorenones via a cobalt-catalyzed MHP-directed [3+2] annulation/ring-opening/dehydration sequence. *Chem. Commun.* **2019**, *55*, 4206.

[6] Nomura, N.; Ishii, R.; Yamamoto, Y.; Kondo, T. Stereoselective Ring-Opening Polymerization of a Racemic Lactide by Using Achiral Salen– and Homosalen–Aluminum Complexes. *Chem. Eur. J.* **2007**, *13*, 4433.

X-Ray Crystallographic Data

A single crystal of **3d-1** suitable for X-ray crystallography was obtained by crystallization via evaporation from its hexane/DCM solution.

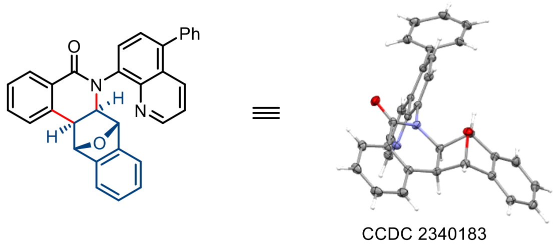

Fig. S1. X-Ray crystallographic data of **3d-1**. The ellipsoids drawn at 30% probability level

Crystal data and structure refinement for **3d-1:**

Identification code	231220_CJH_7_23_5_0m
Empirical formula	$C_{32}H_{22}N_2O_2$
Formula weight	466.51
Temperature/K	170.00
Crystal system	monoclinic
Space group	$P2_1$
a/Å	11.0401(5)
b/Å	8.2753(4)
c/Å	13.6509(7)
α/°	90
β/°	108.894(2)
γ/°	90
Volume/Å^3	1179.95(10)
Z	2
ρ_{calc}g/cm^3	1.313
μ/mm^{-1}	0.415
F(000)	488.0
Crystal size/mm^3	0.13 × 0.06 × 0.04
Radiation	GaKα (λ = 1.34139)
2Θ range for data collection/°	5.954 to 121.15
Index ranges	$-14 \le h \le 14$, $-10 \le k \le 10$, $-17 \le l \le 17$
Reflections collected	28189
Independent reflections	5388 [R_{int} = 0.0312, R_{sigma} = 0.0257]
Data/restraints/parameters	5388/1/325
Goodness-of-fit on F^2	1.047
Final R indexes [I>=2σ (I)]	R_1 = 0.0319, wR_2 = 0.0790

Final R indexes [all data]	R_1 = 0.0334, wR_2 = 0.0803
Largest diff. peak/hole / e $Å^{-3}$	0.13/-0.20
Flack parameter	0.01(7)

A single crystal of **C-Ni(II)** suitable for X-ray crystallography was obtained by crystallization via evaporation from its hexane/acetone solution.

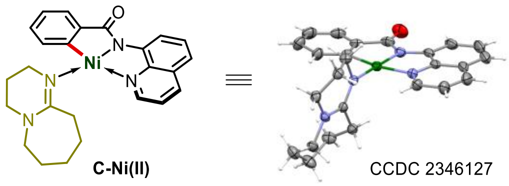

Fig. S2. X-Ray crystallographic data of **C-Ni(II)**. The ellipsoids drawn at 30% probability level

Crystal data and structure refinement for **C-Ni(II):**

Identification code	240402_JTY20240401
Empirical formula	$C_{53}H_{58}N_8Ni_2O_3$
Formula weight	972.49
Temperature/K	260.00
Crystal system	orthorhombic
Space group	Pbca
a/Å	17.5204(17)
b/Å	15.0897(15)
c/Å	35.683(3)
α/°	90
β/°	90
γ/°	90
Volume/Å^3	9433.8(15)
Z	8
ρ_{calc}g/cm^3	1.369
μ/mm^{-1}	0.851
F(000)	4096.0
Crystal size/mm^3	0.26 × 0.09 × 0.08
Radiation	MoKα (λ = 0.71073)
2Θ range for data collection/°	4.566 to 56.654
Index ranges	$-23 \leq h \leq 23, -19 \leq k \leq 20, -47 \leq l \leq 47$
Reflections collected	180226
Independent reflections	11720 [R_{int} = 0.1439, R_{sigma} = 0.0570]
Data/restraints/parameters	11720/0/597
Goodness-of-fit on F^2	1.209
Final R indexes [I>=2σ (I)]	R_1 = 0.0763, wR_2 = 0.0986
Final R indexes [all data]	R_1 = 0.1290, wR_2 = 0.1166
Largest diff. peak/hole / e Å^{-3}	0.35/-0.31

A single crystal of **C-Ni(III)** suitable for X-ray crystallography was obtained by crystallization via evaporation from its hexane/DCM solution.

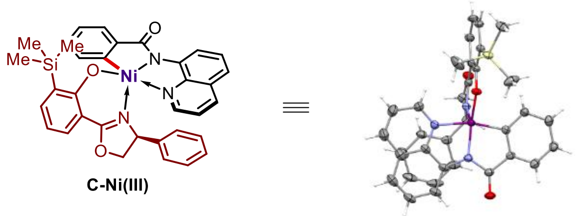

Fig. S3. X-Ray crystallographic data of **C-Ni(III)**. The ellipsoids drawn at 30% probability level

Crystal data and structure refinement for **C-Ni(III):**

Identification code	240223_CJH_7_52_1
Empirical formula	$C_{34}H_{30}N_3NiO_3Si$
Formula weight	615.41
Temperature/K	170.00
Crystal system	orthorhombic
Space group	$P2_12_12_1$
a/Å	28.2487(14)
b/Å	8.4534(4)
c/Å	12.4377(5)
α/°	90
β/°	90
γ/°	90
Volume/Å^3	2970.1(2)
Z	4
ρ_{calc}g/cm^3	1.376
μ/mm^{-1}	4.021
F(000)	1284.0
Crystal size/mm^3	0.07 × 0.04 × 0.02
Radiation	GaKα (λ = 1.34139)
2Θ range for data collection/°	6.756 to 114.892
Index ranges	$-35 \leq h \leq 35$, $-10 \leq k \leq 10$, $-15 \leq l \leq 15$
Reflections collected	50126
Independent reflections	6040 [R_{int} = 0.0834, R_{sigma} = 0.0705]
Data/restraints/parameters	6040/0/383
Goodness-of-fit on F^2	1.055
Final R indexes [I>=2σ (I)]	R_1 = 0.0405, wR_2 = 0.1013
Final R indexes [all data]	R_1 = 0.0468, wR_2 = 0.1033

Largest diff. peak/hole / e Å^{-3}	0.25/-0.57
Flack parameter	0.044(6)

NMR Spectra

^{1}H NMR of **1g-1**

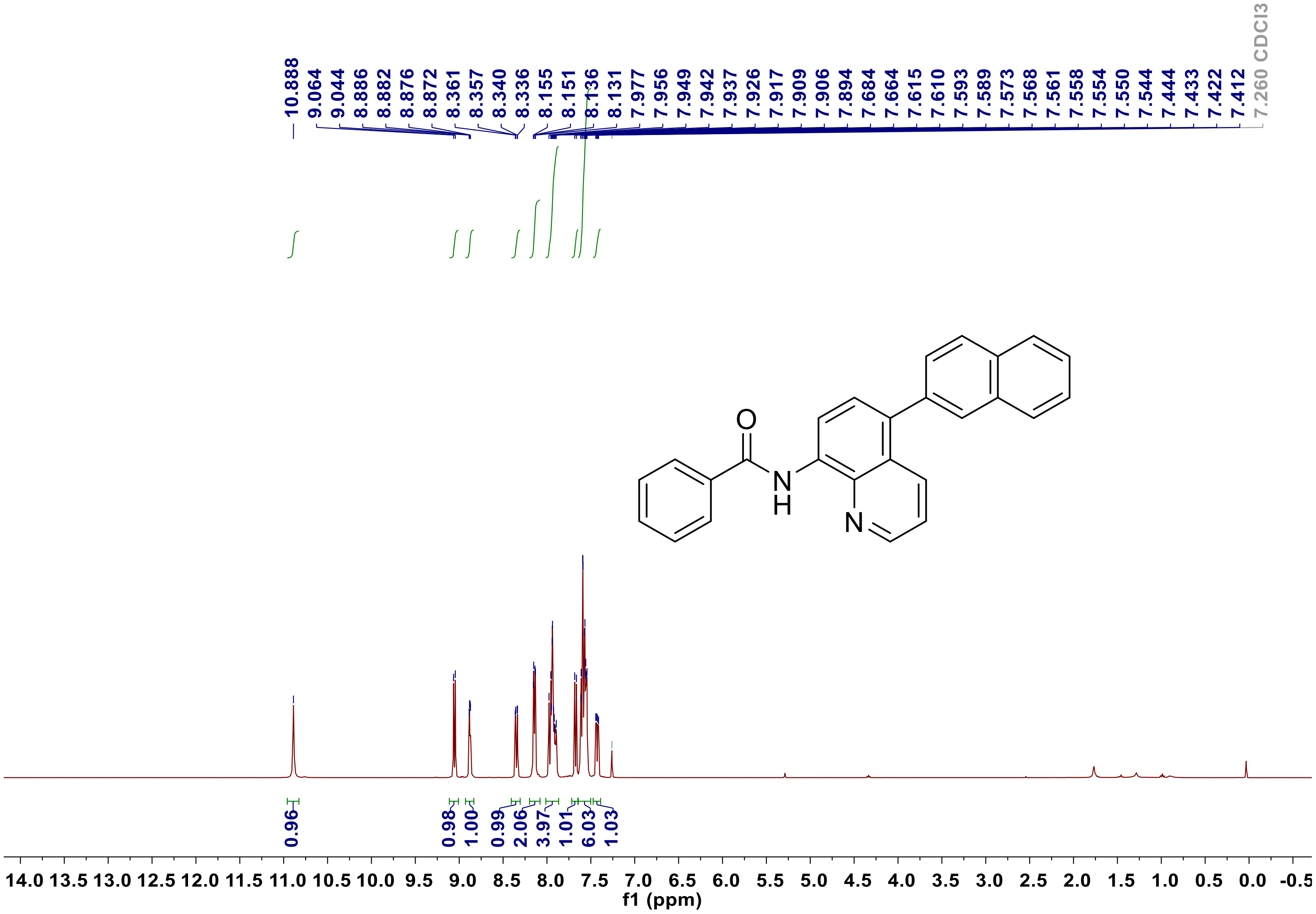
10.888
9.064
9.044
8.886
8.882
8.876
8.872
8.361
8.357
8.340
8.336
8.155
8.151
8.136
8.131
7.977
7.956
7.949
7.942
7.937
7.926
7.917
7.909
7.906
7.894
7.684
7.664
7.615
7.610
7.593
7.589
7.573
7.568
7.561
7.558
7.554
7.550
7.544
7.444
7.433
7.422
7.412
7.260 CDCl3
0.96
0.98
1.00
0.99
2.06
3.97
1.01
6.03
1.03
14.0 13.5 13.0 12.5 12.0 11.5 11.0 10.5 10.0 9.5 9.0 8.5 8.0 7.5 7.0 6.5 6.0 5.5 5.0 4.5 4.0 3.5 3.0 2.5 2.0 1.5 1.0 0.5 0.0 -0.5
f1 (ppm)
O
N
H
N

^{13}C NMR of **1g-1**

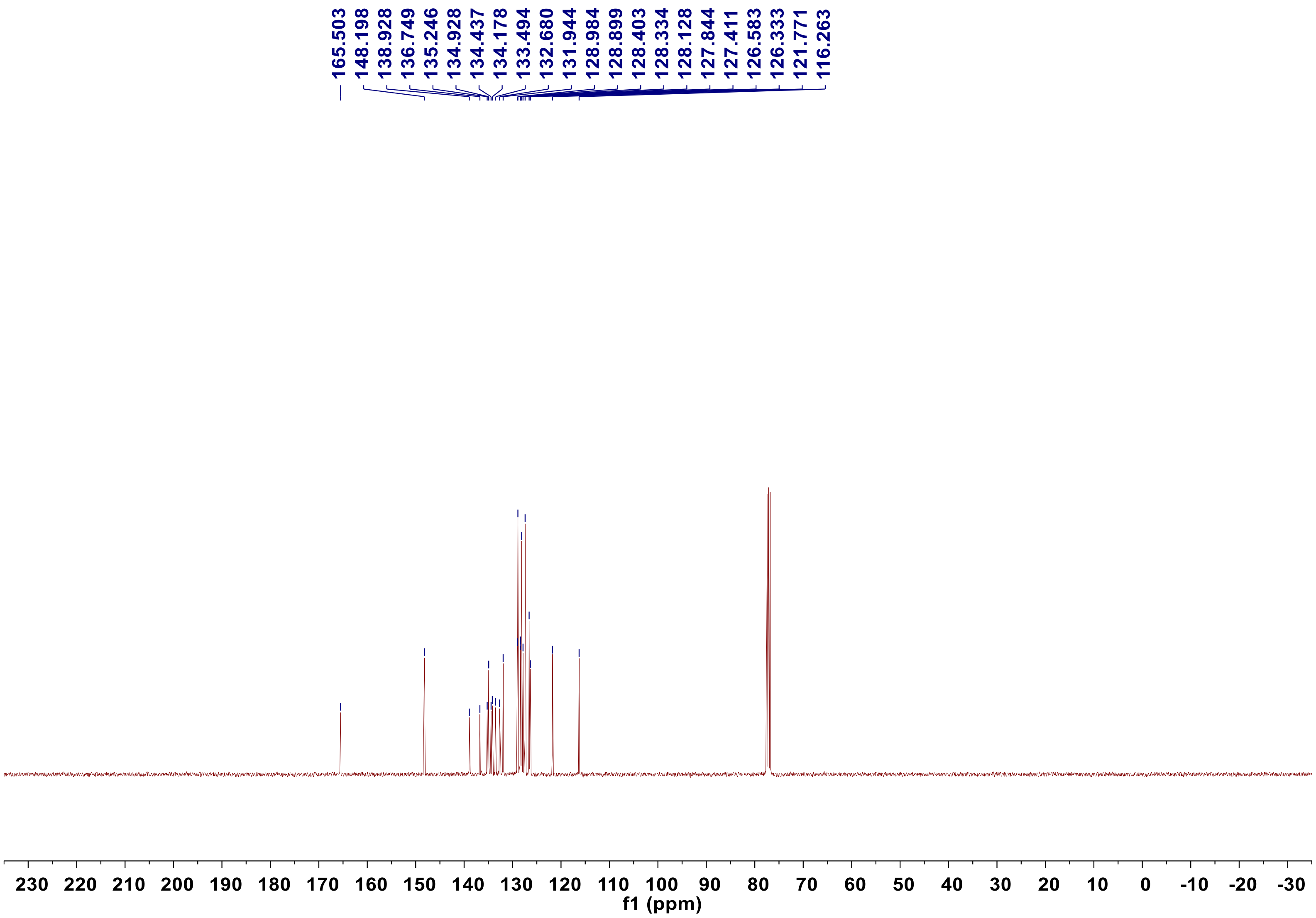

^{1}H NMR of **1h-1**

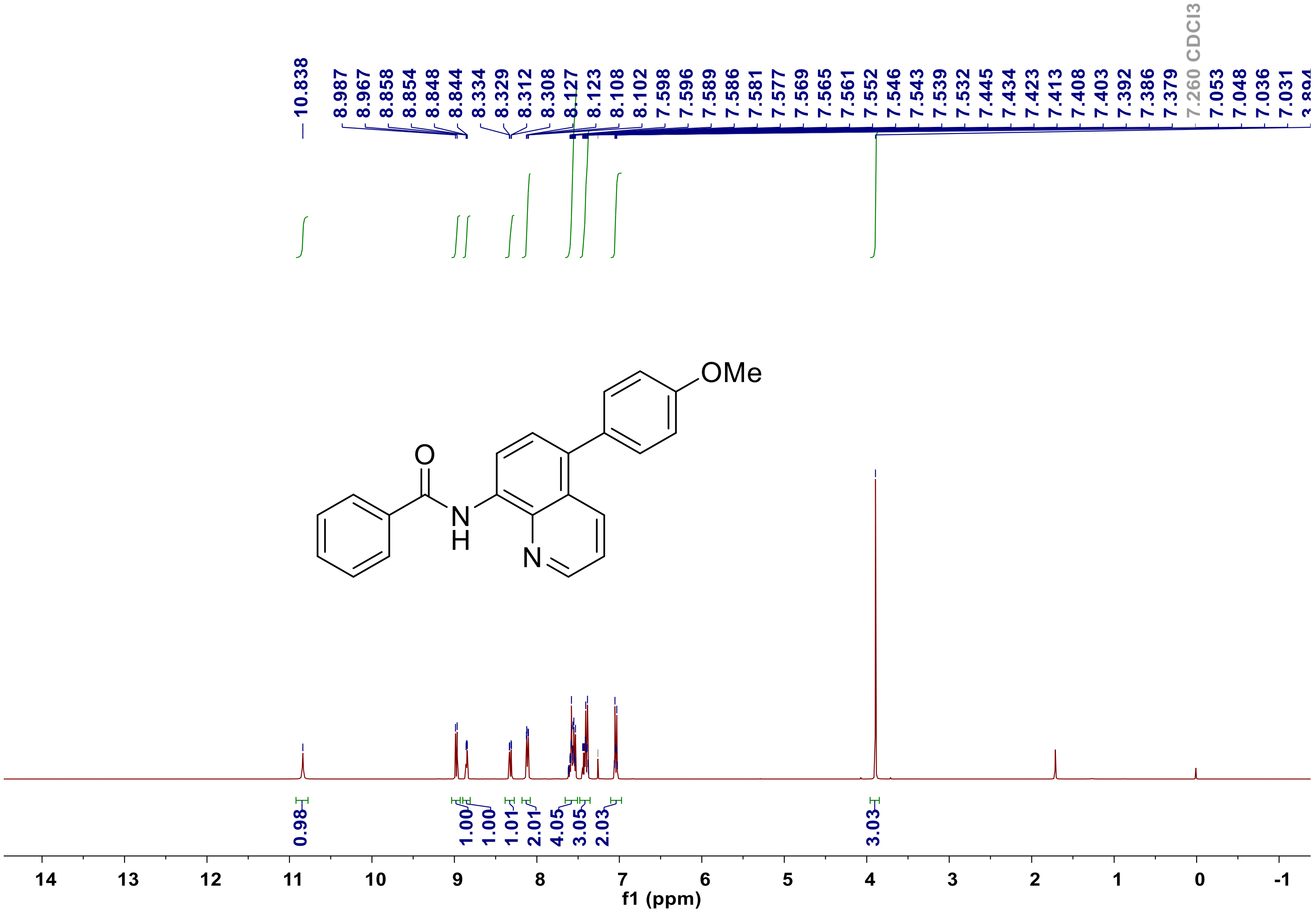

10.838
8.987
8.967
8.858
8.854
8.848
8.844
8.334
8.329
8.312
8.308
8.127
8.123
8.108
8.102
7.598
7.596
7.589
7.586
7.581
7.577
7.569
7.565
7.561
7.552
7.546
7.543
7.539
7.532
7.445
7.434
7.423
7.413
7.408
7.403
7.392
7.386
7.379
7.260 CDCl3
7.053
7.048
7.036
7.031
OMe
O
N
H
N
0.98
1.00
1.00
1.01
2.01
4.05
3.05
2.03
3.03
14
13
12
11
10
9
8
7
6
5
4
3
2
1
0
-1
f1 (ppm)

^{13}C NMR of **1h-1**

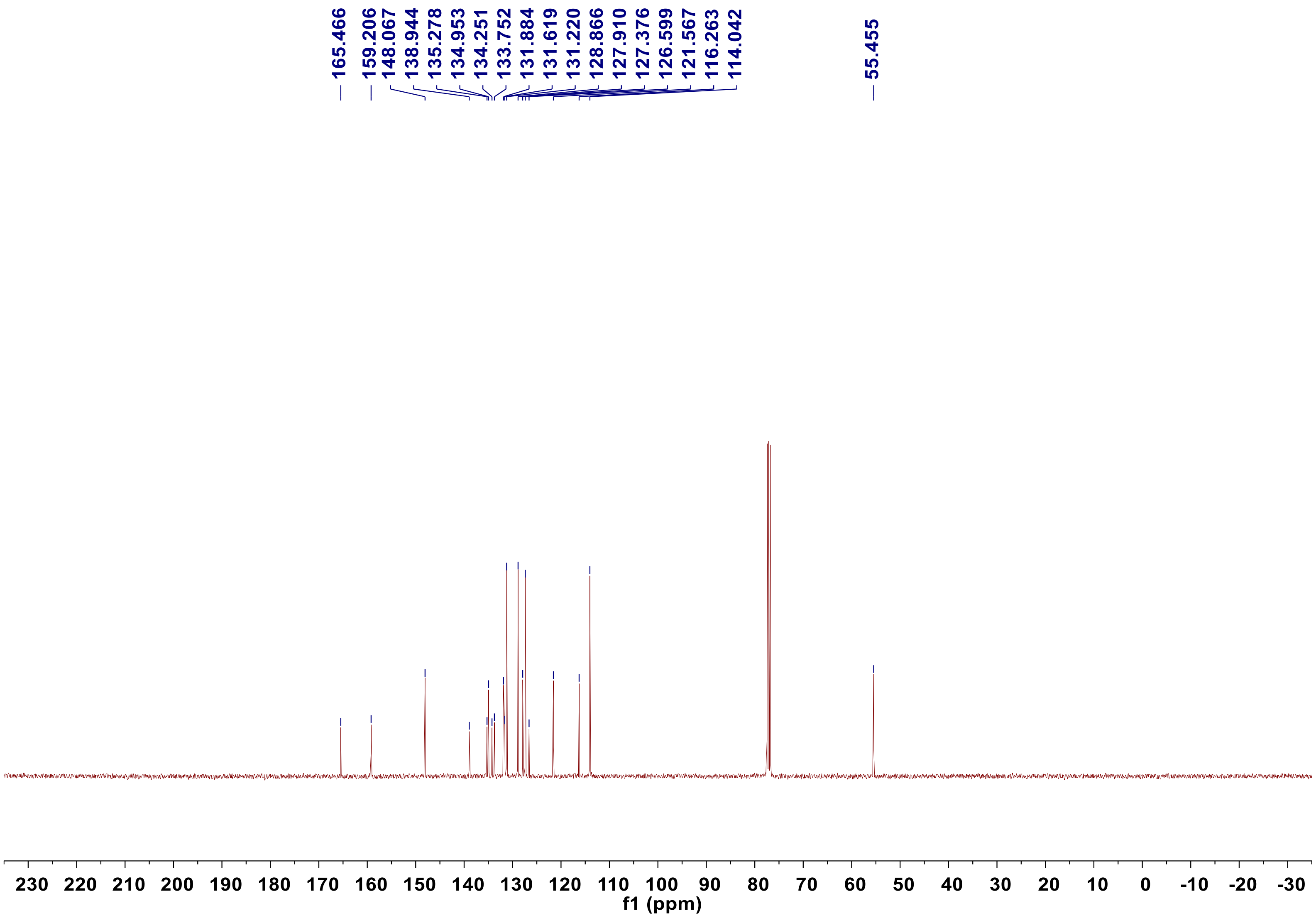

^{1}H NMR of **1i-1**

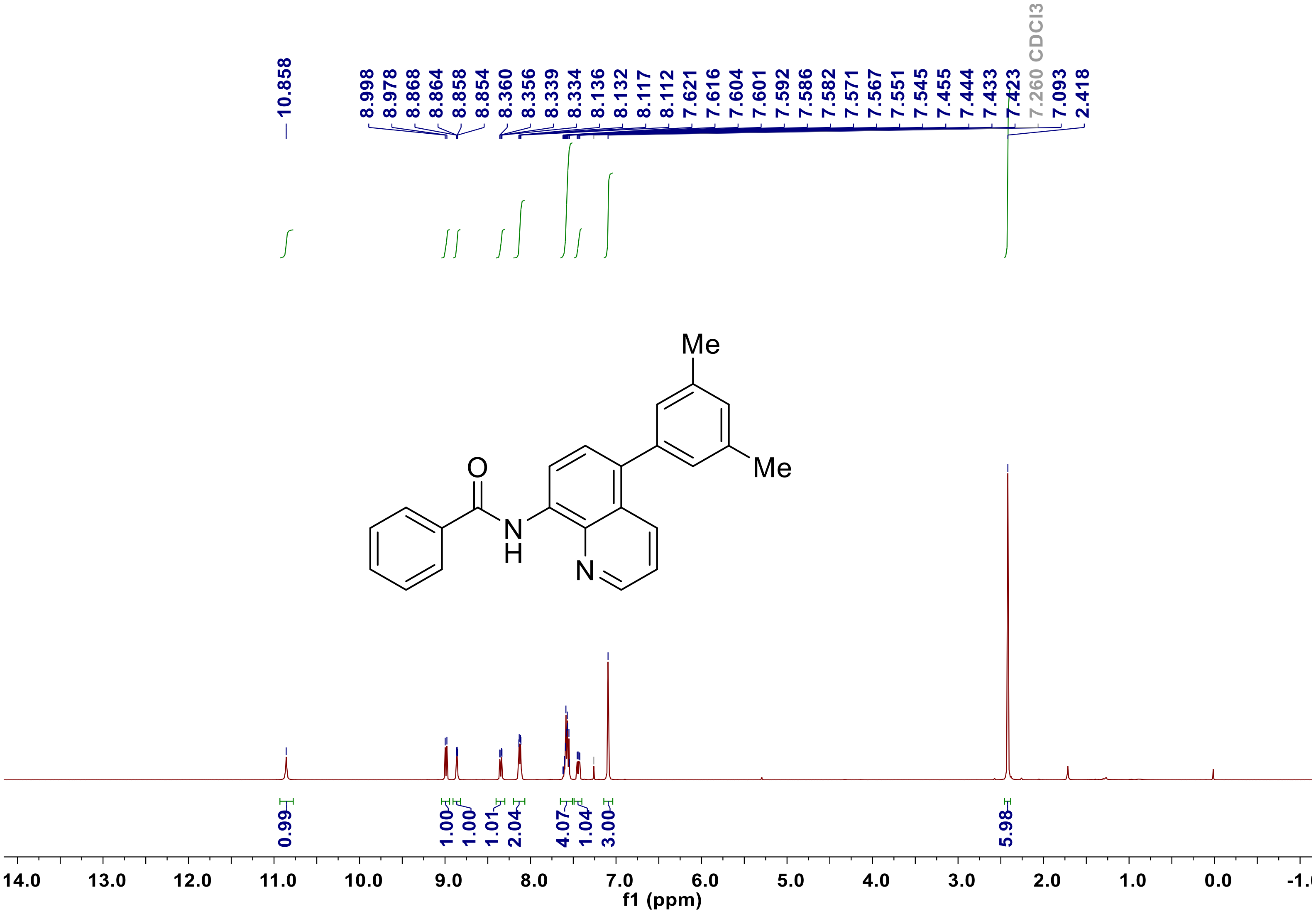

10.858
8.998
8.978
8.868
8.864
8.858
8.854
8.360
8.356
8.339
8.334
8.136
8.132
8.117
8.112
7.621
7.616
7.604
7.601
7.592
7.586
7.582
7.571
7.567
7.551
7.545
7.455
7.444
7.433
7.423
7.260 CDCl3
7.093
2.418
Me
Me
O
N
H
N
0.99
1.00
1.00
1.01
2.04
4.07
1.04
3.00
5.98
14.0
13.0
12.0
11.0
10.0
9.0
8.0
7.0
6.0
5.0
4.0
3.0
2.0
1.0
0.0
f1 (ppm)

^{13}C NMR of **1i-1**

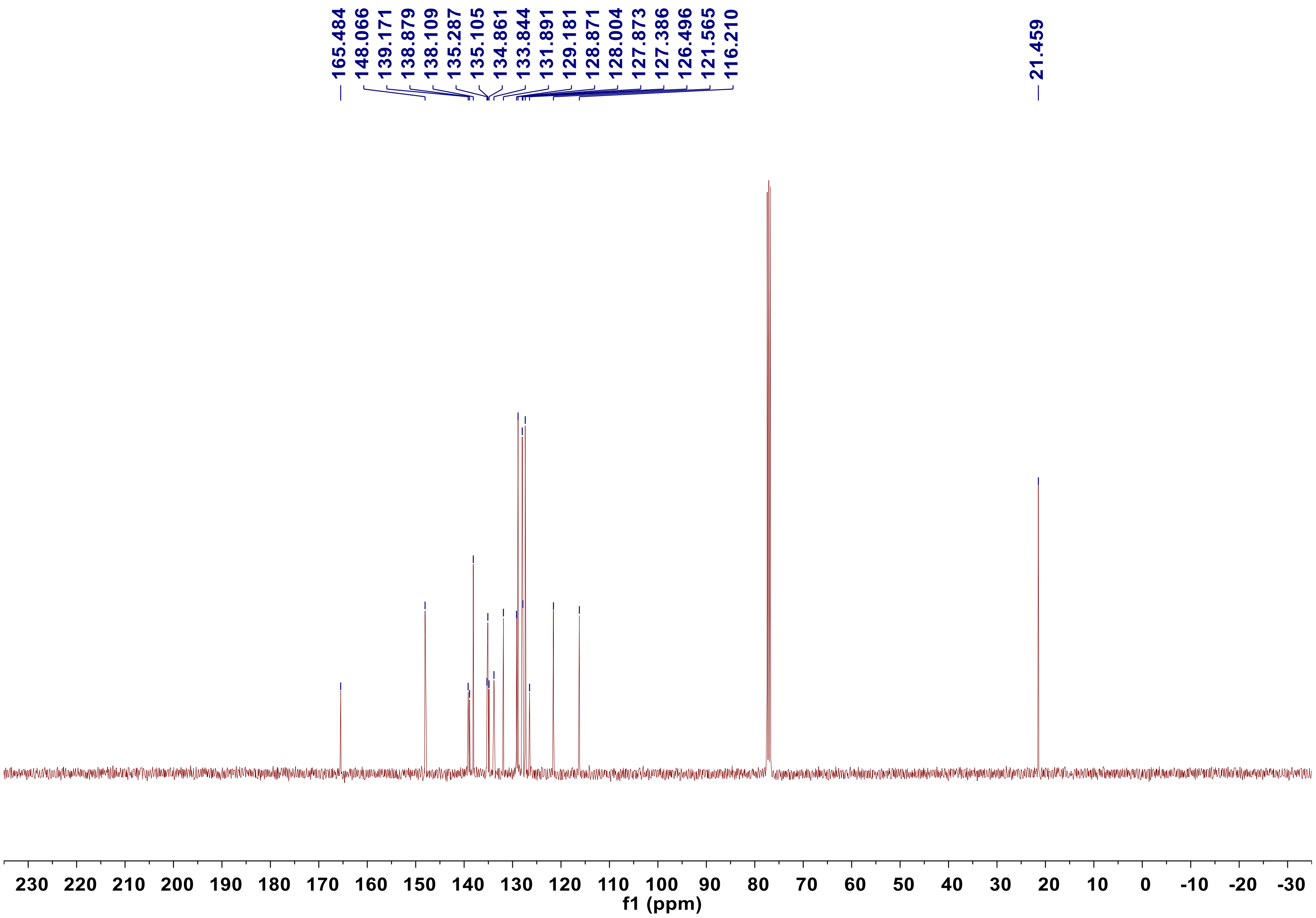

^{1}H NMR of **1j-1**

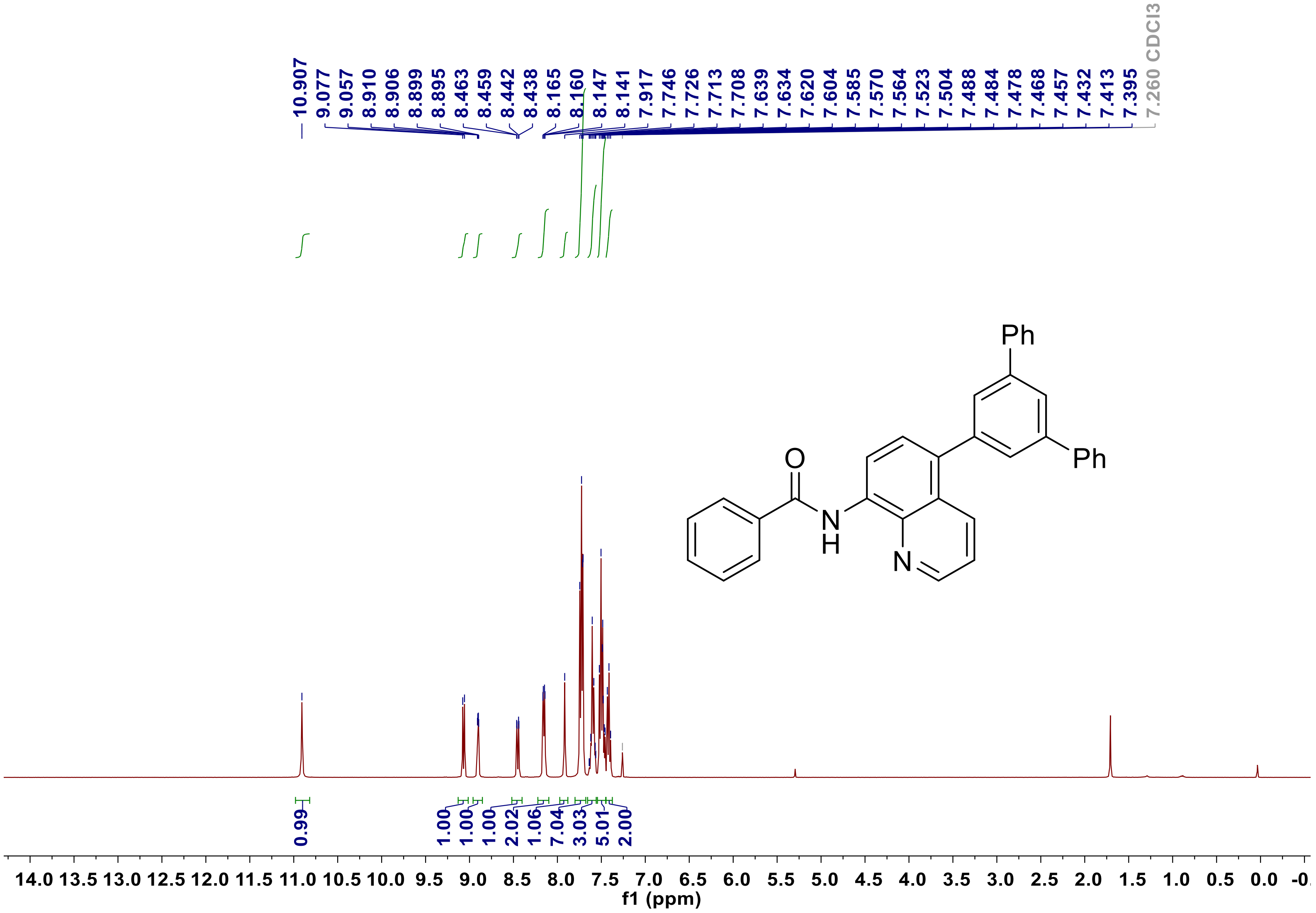

10.907
9.077
9.057
8.910
8.906
8.899
8.895
8.463
8.459
8.442
8.438
8.165
8.160
8.147
8.141
7.917
7.746
7.726
7.713
7.708
7.639
7.634
7.620
7.604
7.585
7.570
7.564
7.523
7.504
7.488
7.484
7.478
7.468
7.457
7.432
7.413
7.395
7.260 CDCl3
0.99
1.00
1.00
1.00
2.02
1.06
7.04
3.03
5.01
2.00
14.0 13.5 13.0 12.5 12.0 11.5 11.0 10.5 10.0 9.5 9.0 8.5 8.0 7.5 7.0 6.5 6.0 5.5 5.0 4.5 4.0 3.5 3.0 2.5 2.0 1.5 1.0 0.5 0.0
f1 (ppm)
O
N
H
N
Ph
Ph

^{13}C NMR of **1j-1**

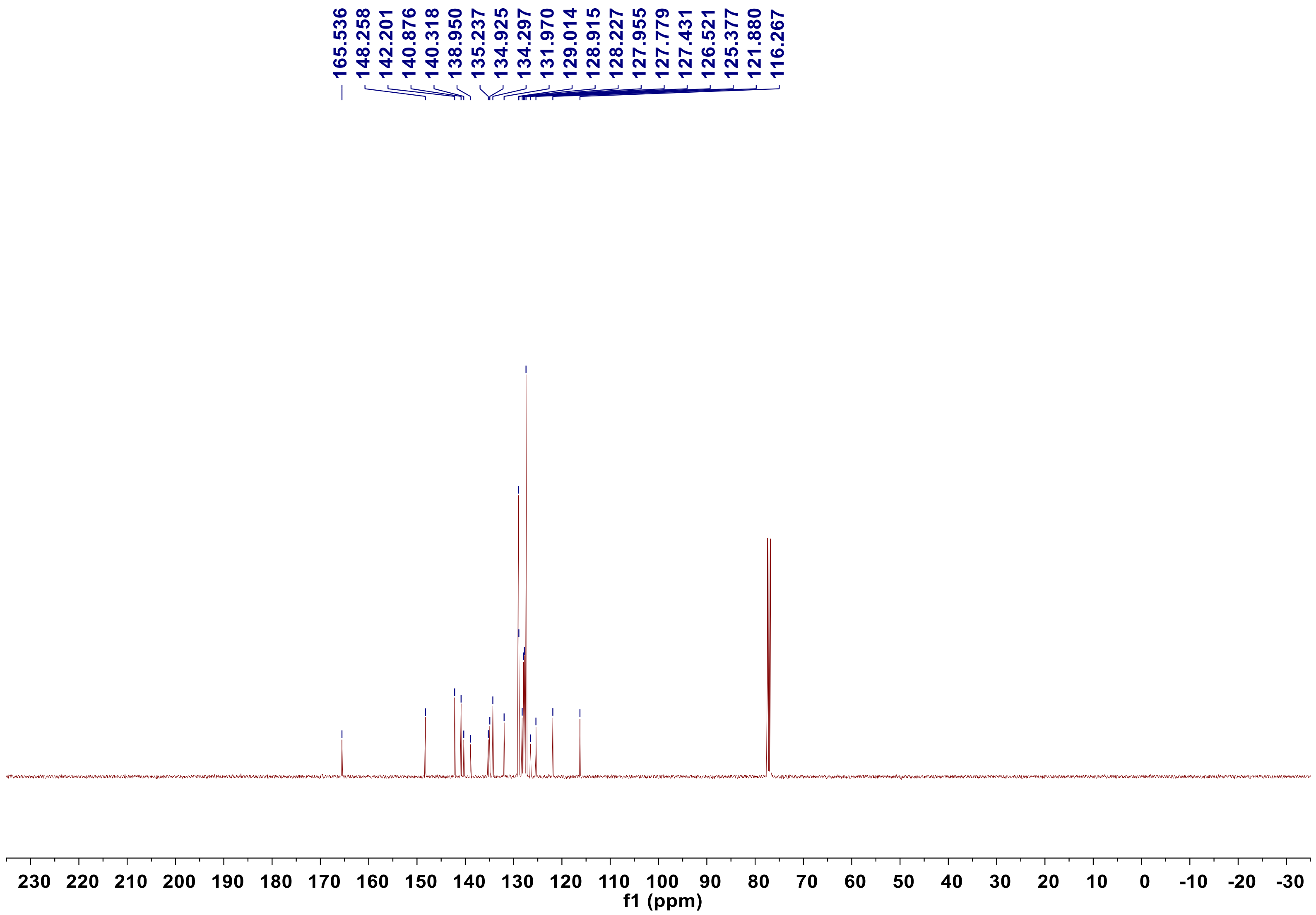

^{1}H NMR of **NQ**

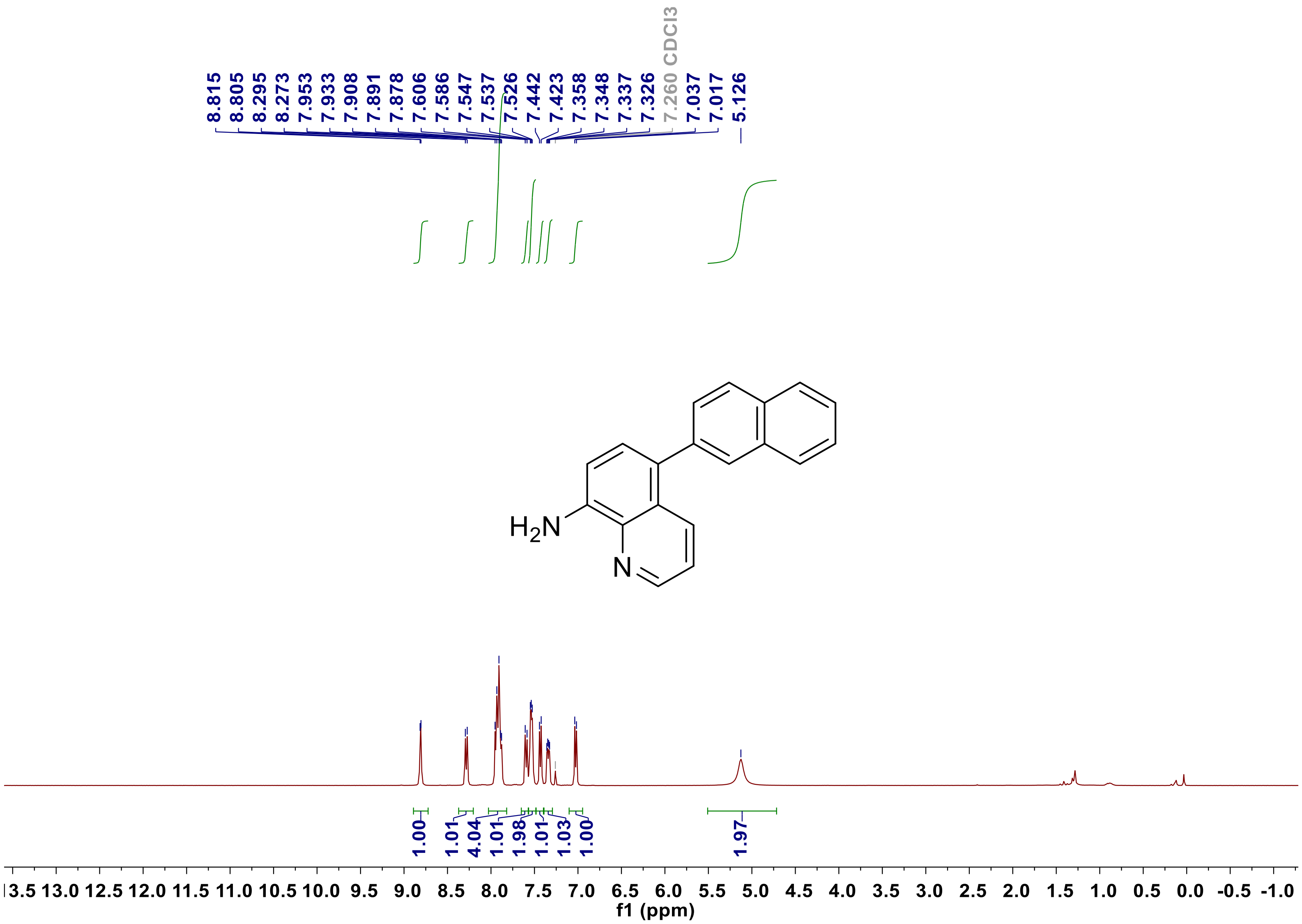
8.815 8.805 8.295 8.273 7.953 7.933 7.908 7.891 7.878 7.606 7.586 7.547 7.537 7.526 7.442 7.423 7.358 7.348 7.337 7.326 7.260 CDCl3 7.037 7.017 5.126
H2N
N
1.00 1.01 4.04 1.01 1.98 1.01 1.03 1.00 1.97
13.5 13.0 12.5 12.0 11.5 11.0 10.5 10.0 9.5 9.0 8.5 8.0 7.5 7.0 6.5 6.0 5.5 5.0 4.5 4.0 3.5 3.0 2.5 2.0 1.5 1.0 0.5 0.0 -0.5 -1.0
f1 (ppm)

^{13}C NMR of **NQ**

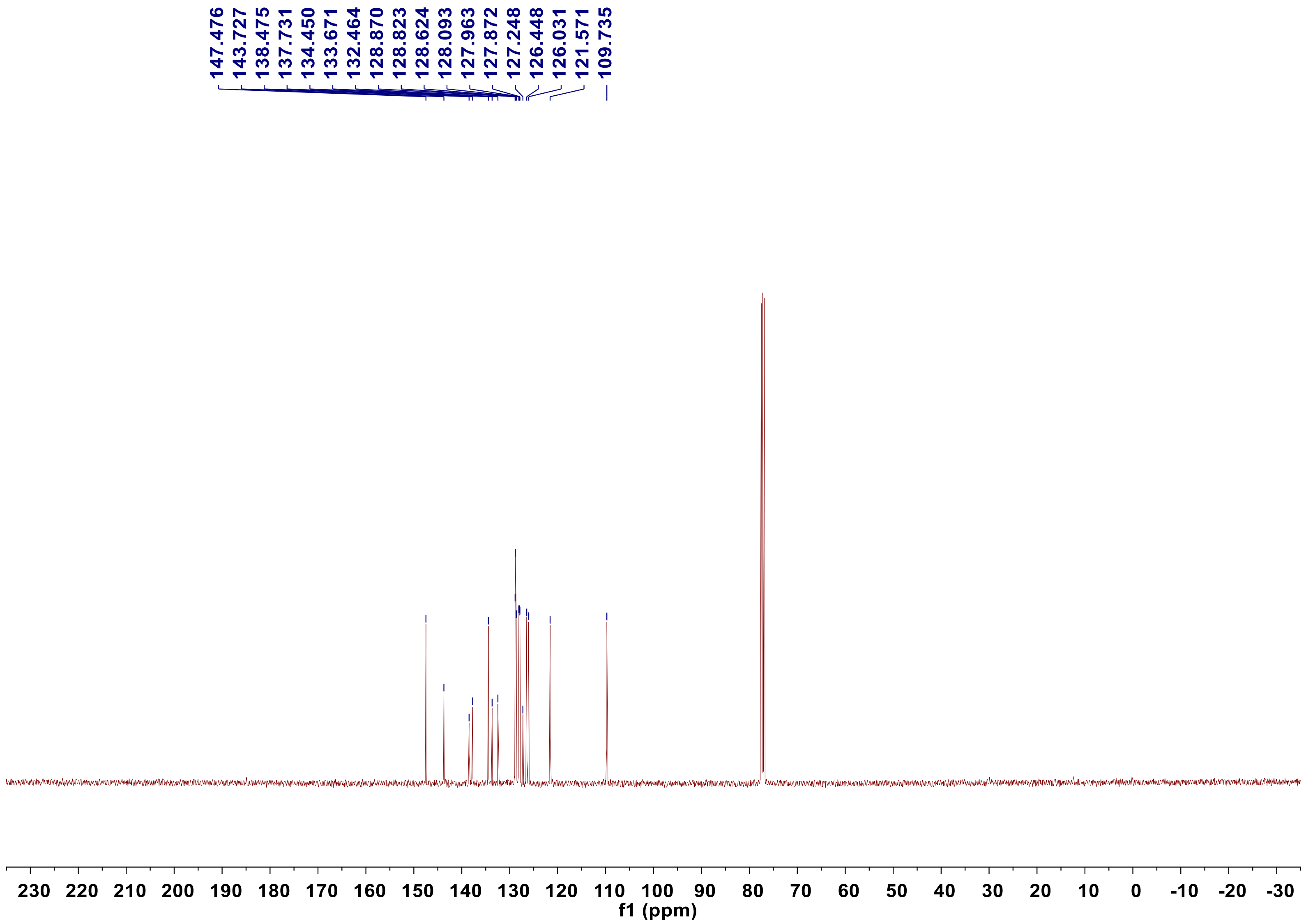
147.476
143.727
138.475
137.731
134.450
133.671
132.464
128.870
128.823
128.624
128.093
127.963
127.872
127.248
126.448
126.031
121.571
109.735
230 220 210 200 190 180 170 160 150 140 130 120 110 100 90 80 70 60 50 40 30 20 10 0 -10 -20 -30
f1 (ppm)

^{1}H NMR of **1g-2**

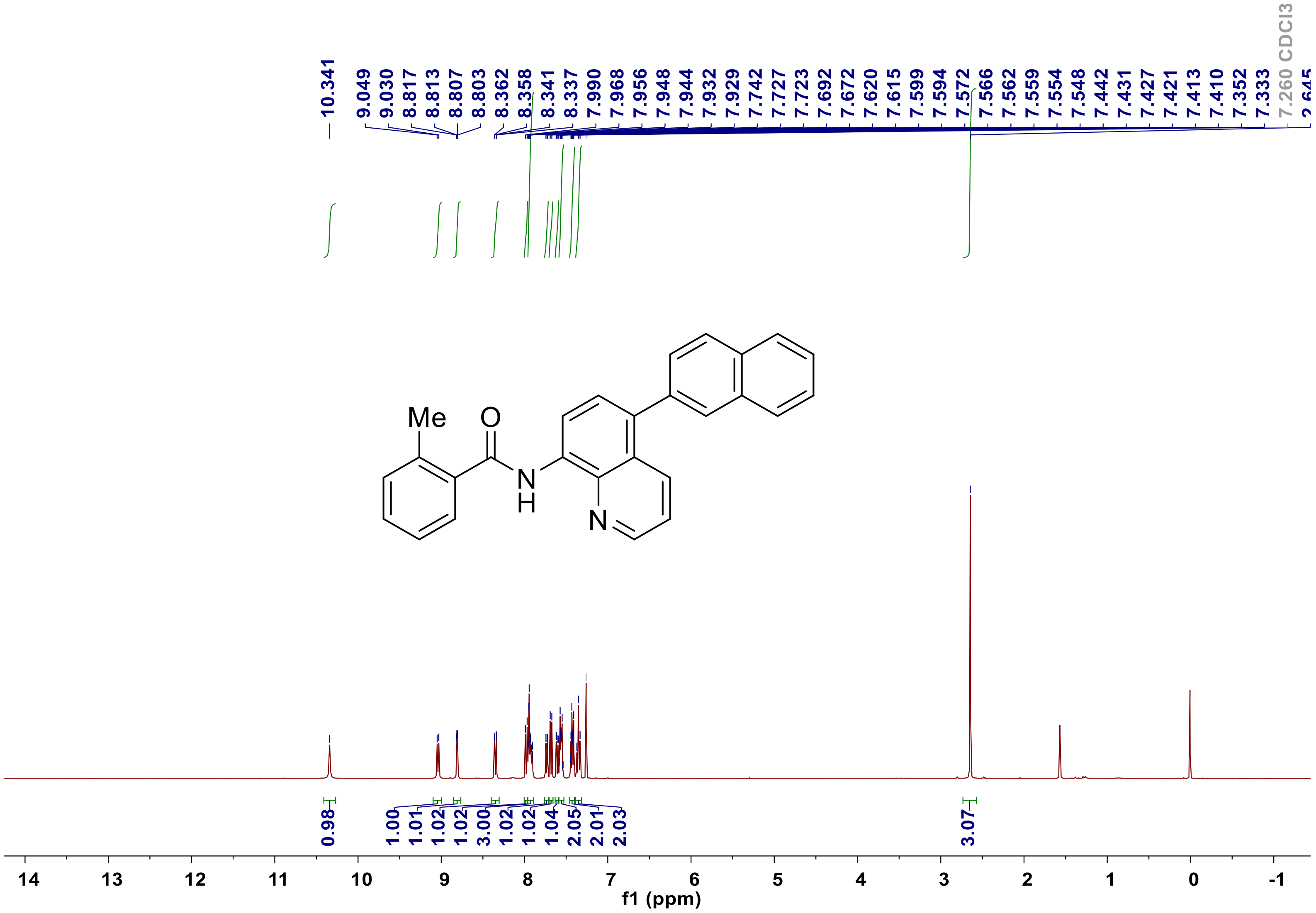

10.341
9.049
9.030
8.817
8.813
8.807
8.803
8.362
8.358
8.341
8.337
7.990
7.968
7.956
7.948
7.944
7.932
7.929
7.742
7.727
7.723
7.692
7.672
7.620
7.615
7.599
7.594
7.572
7.566
7.562
7.559
7.554
7.548
7.442
7.431
7.427
7.421
7.413
7.410
7.352
7.333
7.260 CDCl3
Me
O
N
H
N
0.98
1.00
1.01
1.02
1.02
3.00
1.02
1.02
1.04
2.05
2.01
2.03
3.07
f1 (ppm)

^{13}C NMR of **1g-2**

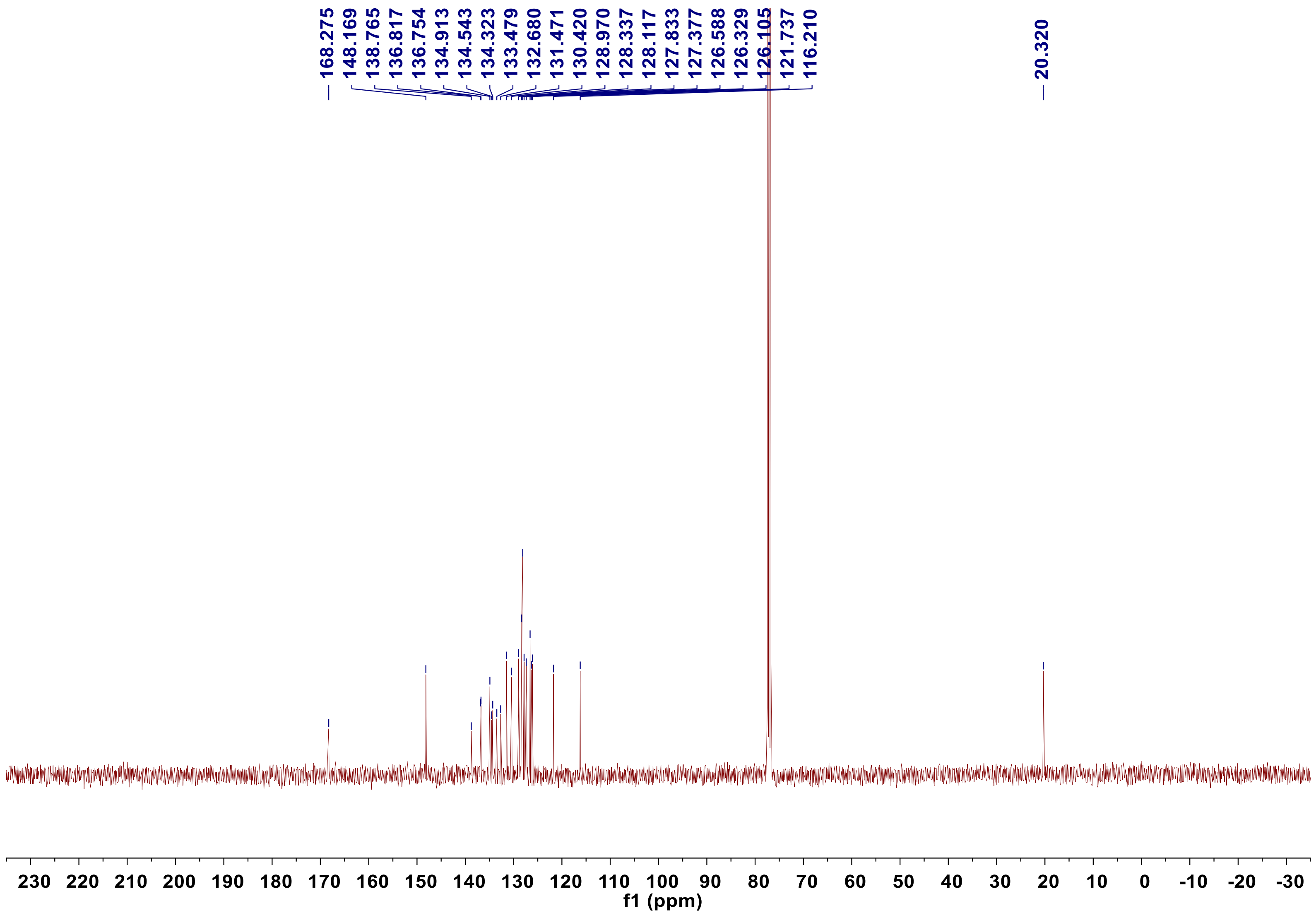

^{1}H NMR of **1g-3**

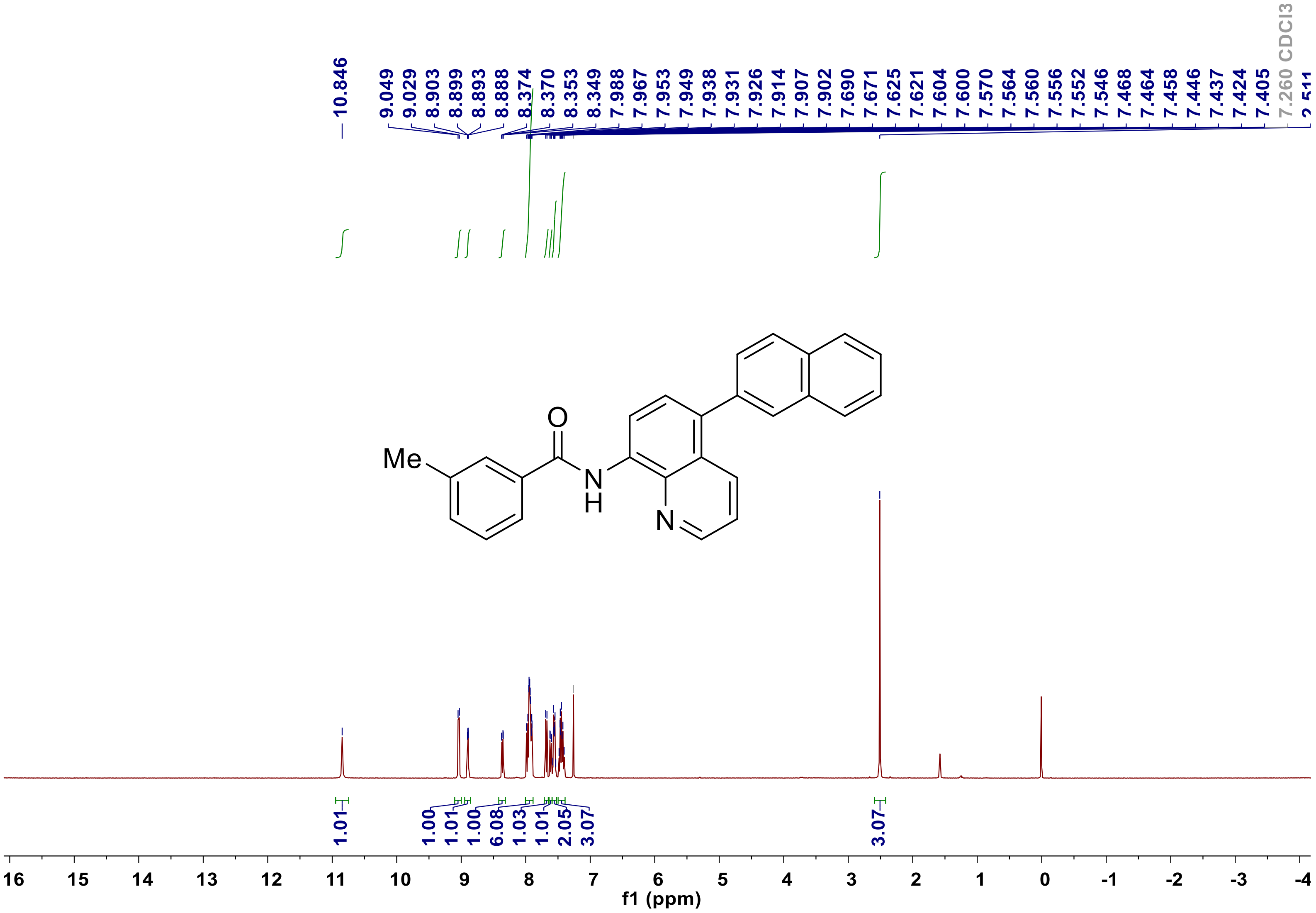

^{13}C NMR of **1g-3**

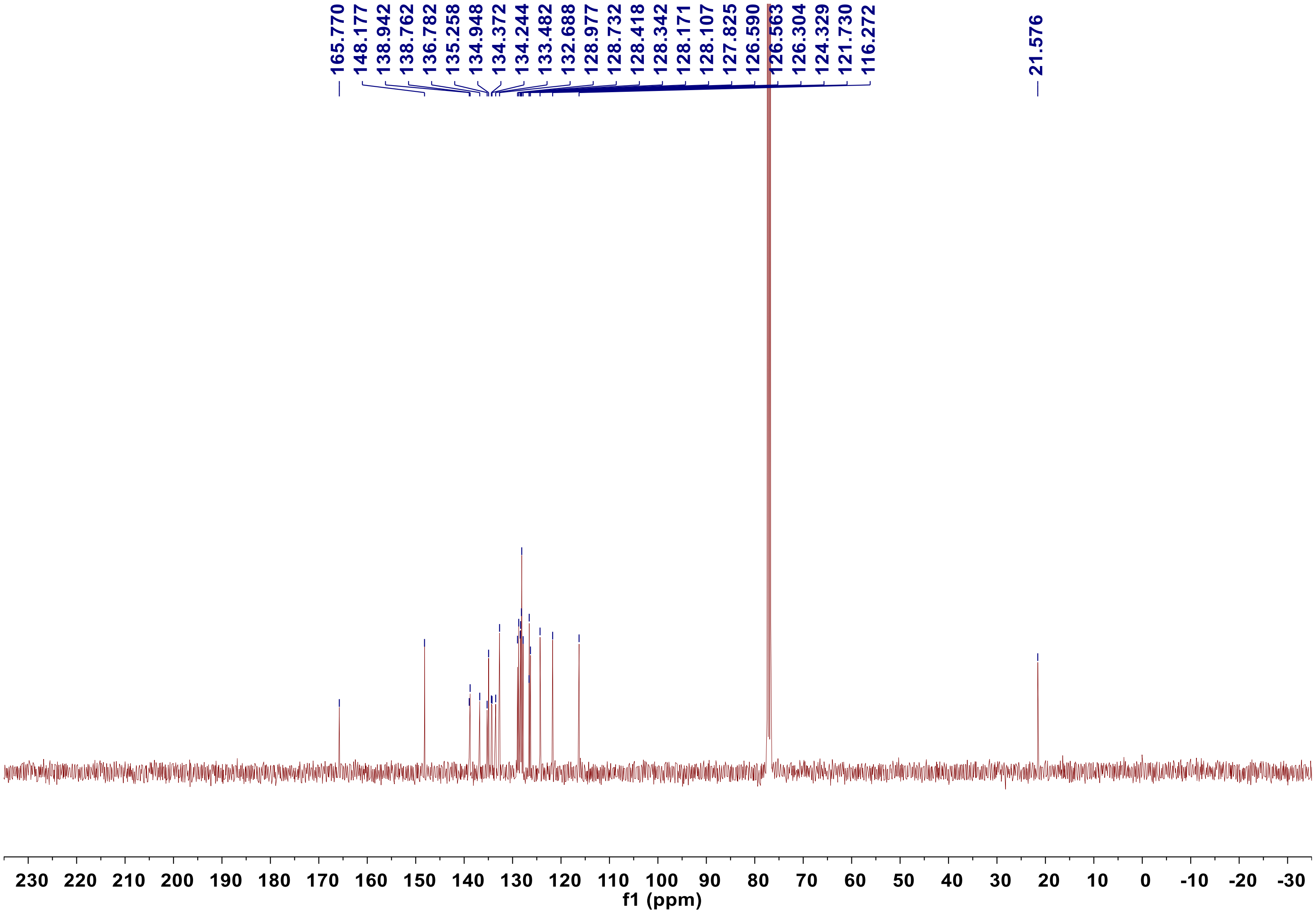

^{1}H NMR of **1g-4**

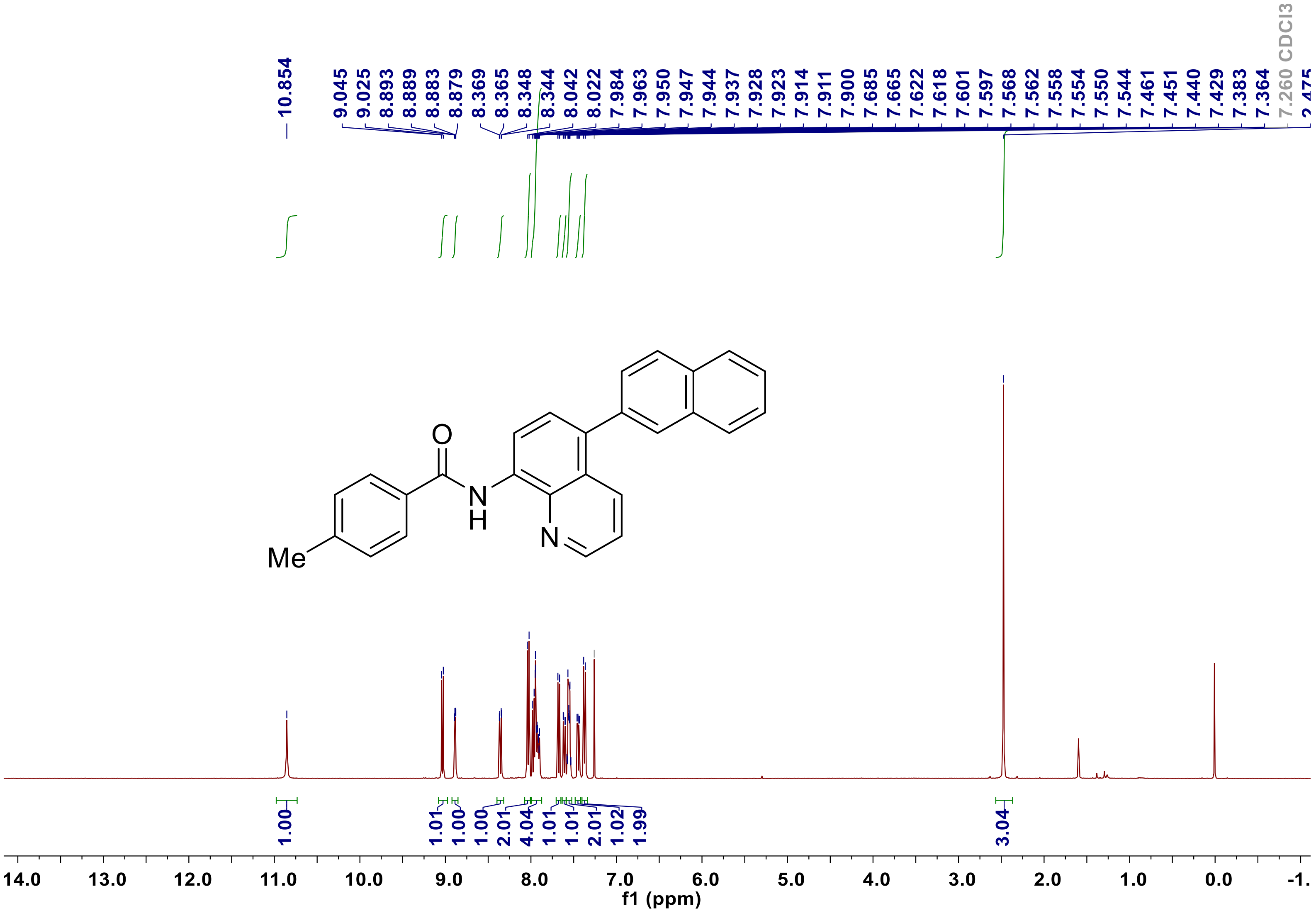

10.854
9.045
9.025
8.893
8.889
8.883
8.879
8.369
8.365
8.348
8.344
8.042
8.022
7.984
7.963
7.950
7.947
7.944
7.937
7.928
7.923
7.914
7.911
7.900
7.685
7.665
7.622
7.618
7.601
7.597
7.568
7.562
7.558
7.554
7.550
7.544
7.461
7.451
7.440
7.429
7.383
7.364
7.260 CDCl3
1.00
1.01
1.00
1.00
2.01
4.04
1.01
1.01
2.01
1.02
1.99
3.04
O
N
H
N
Me
14.0
13.0
12.0
11.0
10.0
9.0
8.0
7.0
6.0
5.0
4.0
3.0
2.0
1.0
0.0
f1 (ppm)

^{13}C NMR of **1g-4**

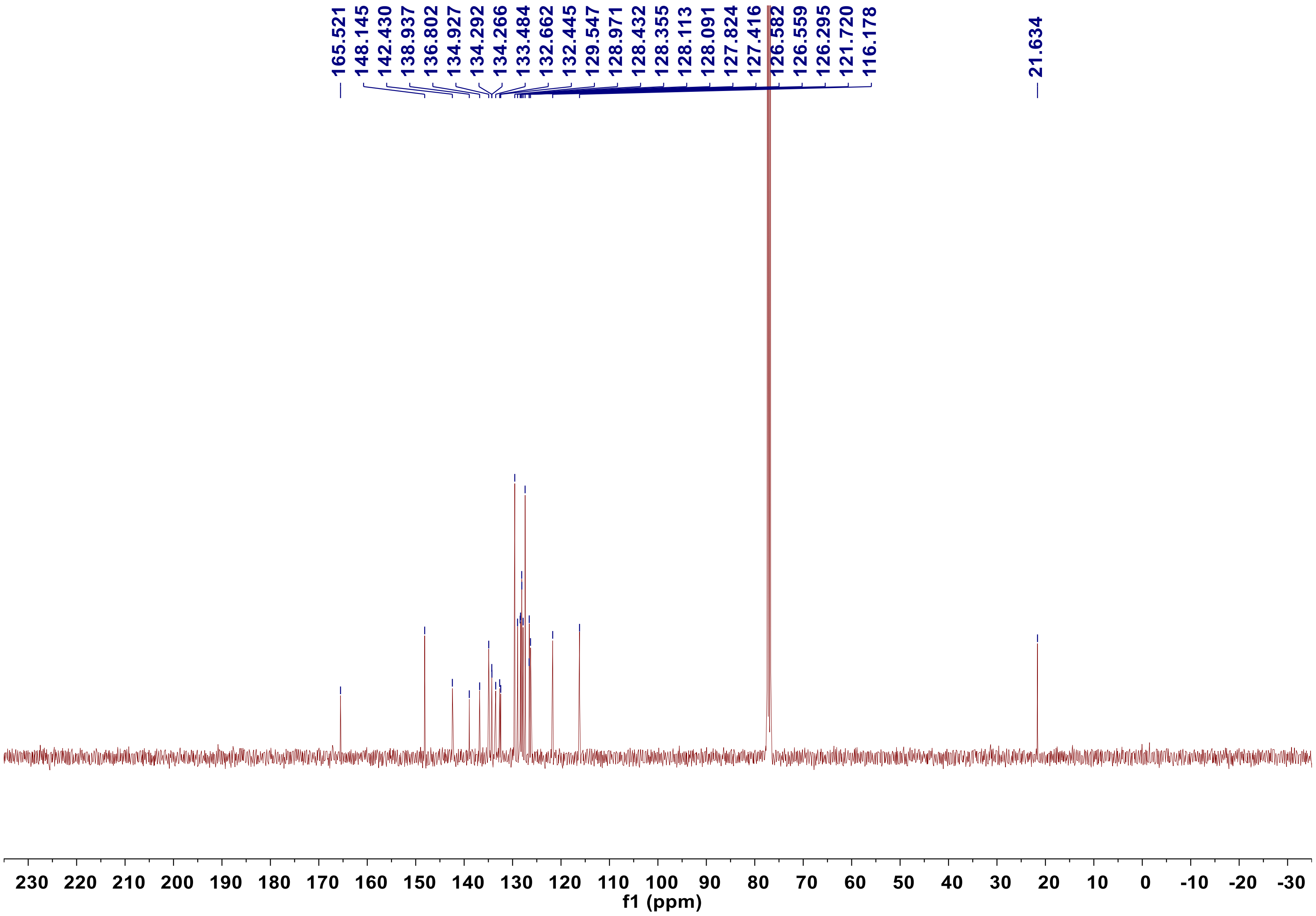

^{1}H NMR of **1g-5**

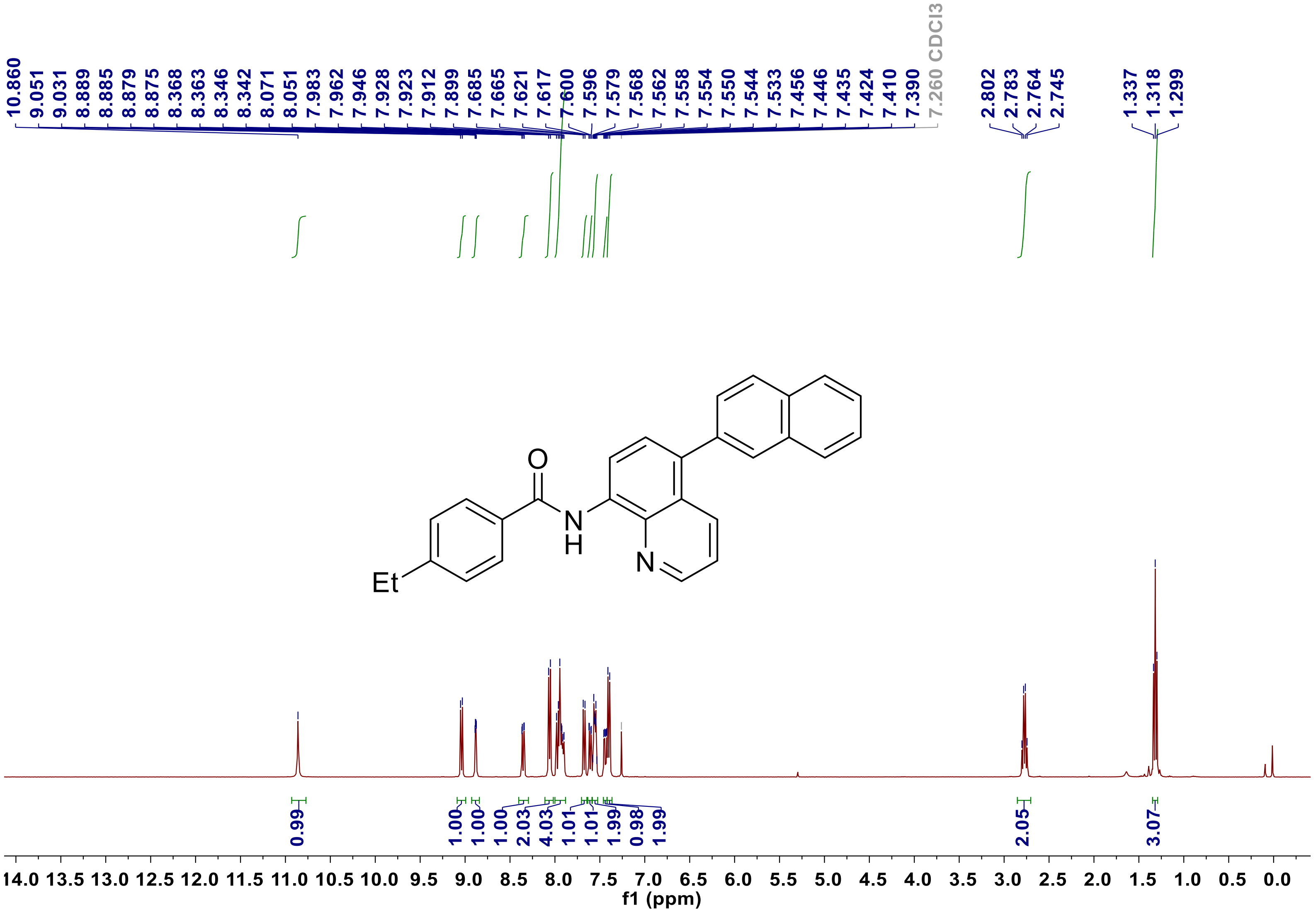
10.860
9.051
9.031
8.889
8.885
8.879
8.875
8.368
8.363
8.346
8.342
8.071
8.051
7.983
7.962
7.946
7.928
7.923
7.912
7.899
7.685
7.665
7.621
7.617
7.600
7.596
7.579
7.568
7.562
7.558
7.554
7.550
7.544
7.533
7.456
7.446
7.435
7.424
7.410
7.390
7.260 CDCl3
2.802
2.783
2.764
2.745
1.337
1.318
1.299
O
N
H
N
Et
0.99
1.00
1.00
1.00
2.03
4.03
1.01
1.01
1.99
0.98
1.99
2.05
3.07
14.0 13.5 13.0 12.5 12.0 11.5 11.0 10.5 10.0 9.5 9.0 8.5 8.0 7.5 7.0 6.5 6.0 5.5 5.0 4.5 4.0 3.5 3.0 2.5 2.0 1.5 1.0 0.5 0.0
f1 (ppm)

^{13}C NMR of **1g-5**

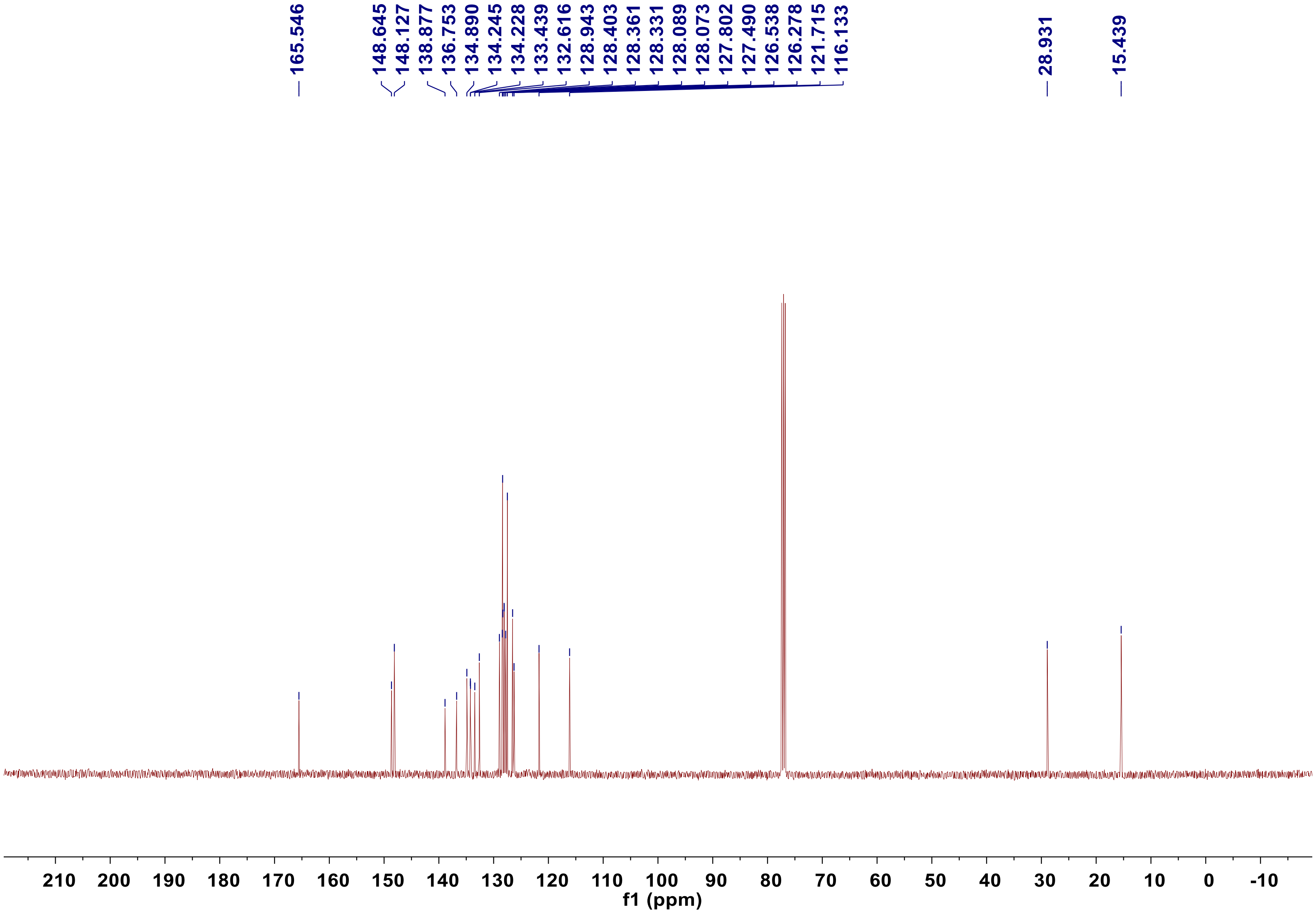

^{1}H NMR of **1g-6**

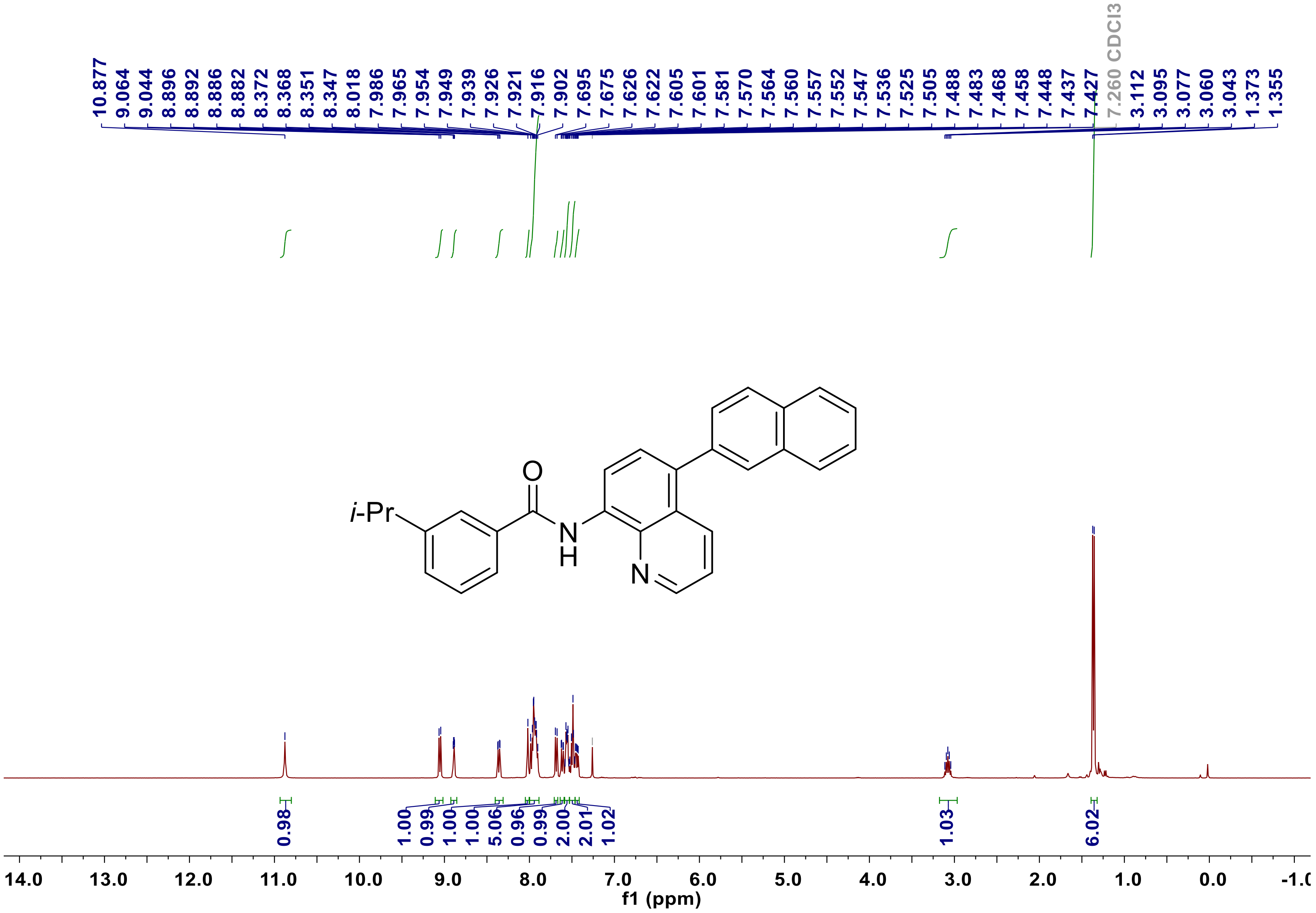

10.877 9.064 9.044 8.896 8.892 8.886 8.882 8.372 8.368 8.351 8.347 8.018 7.986 7.965 7.954 7.949 7.939 7.926 7.921 7.916 7.902 7.695 7.675 7.626 7.622 7.605 7.601 7.581 7.570 7.564 7.560 7.557 7.552 7.547 7.536 7.525 7.505 7.488 7.483 7.468 7.458 7.448 7.437 7.427
7.260 CDCl3
3.112 3.095 3.077 3.060 3.043 1.373 1.355
i-Pr
O
N
H
N
0.98 1.00 0.99 1.00 1.00 5.06 0.96 0.99 2.00 2.01 1.02 1.03 6.02
14.0 13.0 12.0 11.0 10.0 9.0 8.0 7.0 6.0 5.0 4.0 3.0 2.0 1.0 0.0
f1 (ppm)

^{13}C NMR of **1g-6**

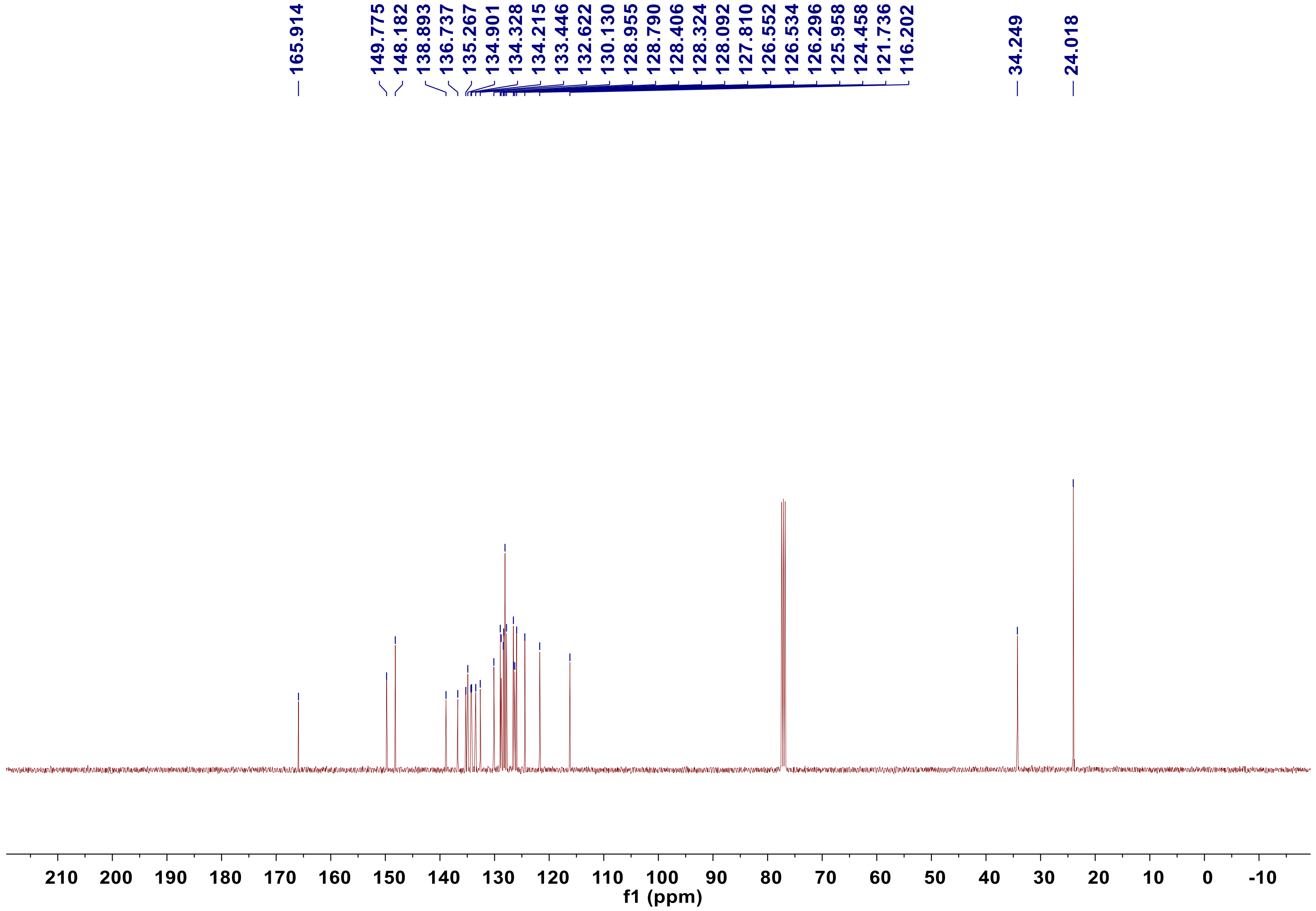

^{1}H NMR of **1g-7**

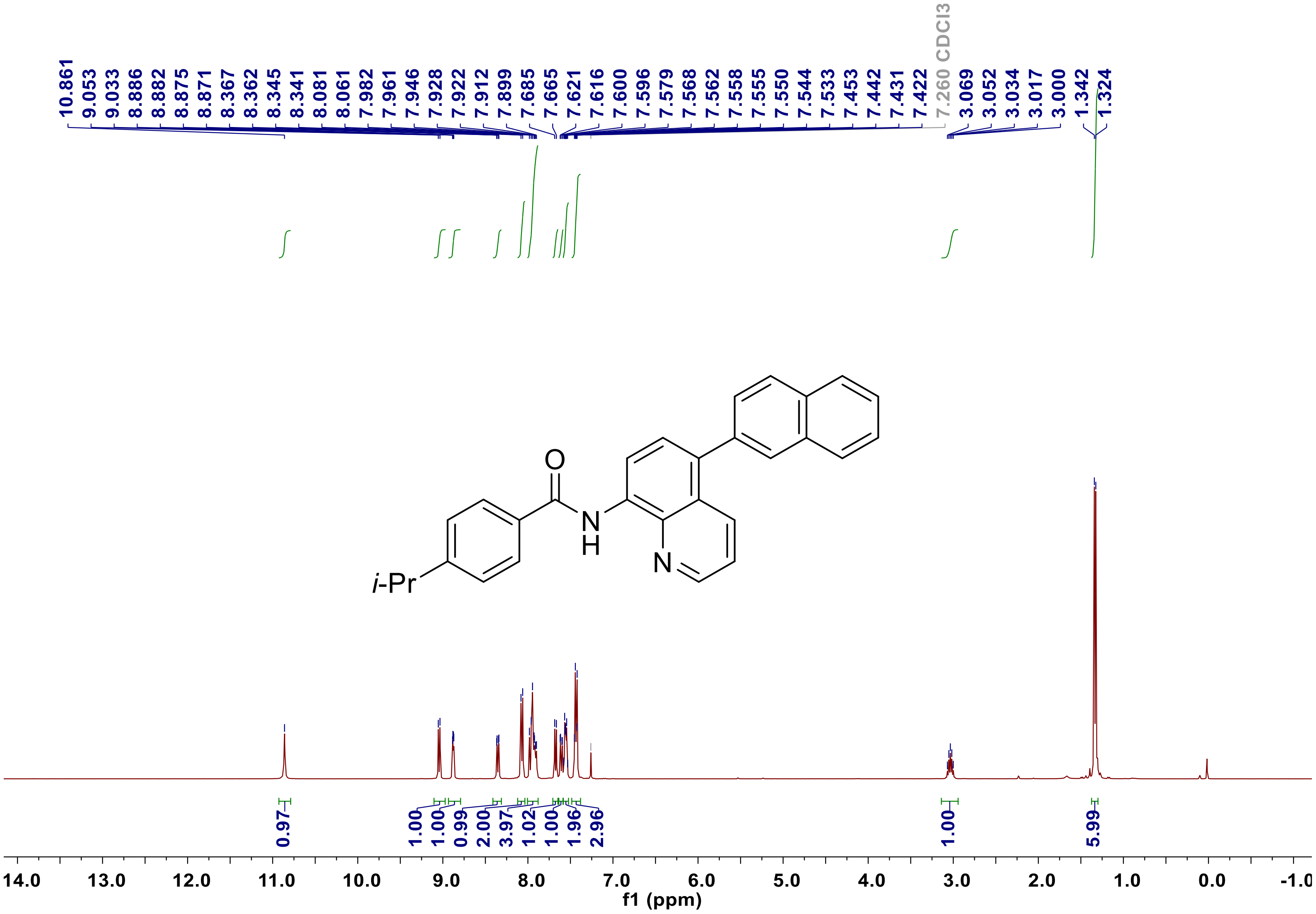
10.861
9.053
9.033
8.886
8.882
8.875
8.871
8.367
8.362
8.345
8.341
8.081
8.061
7.982
7.961
7.946
7.928
7.922
7.912
7.899
7.685
7.665
7.621
7.616
7.600
7.596
7.579
7.568
7.562
7.558
7.555
7.550
7.544
7.533
7.453
7.442
7.431
7.422
7.260 CDCl3
3.069
3.052
3.034
3.017
3.000
1.342
1.324
O
N
H
N
i-Pr
0.97
1.00
1.00
0.99
2.00
3.97
1.02
1.00
1.96
2.96
1.00
5.99
14.0
13.0
12.0
11.0
10.0
9.0
8.0
7.0
6.0
5.0
4.0
3.0
2.0
1.0
0.0
-1.0
f1 (ppm)

^{13}C NMR of **1g-7**

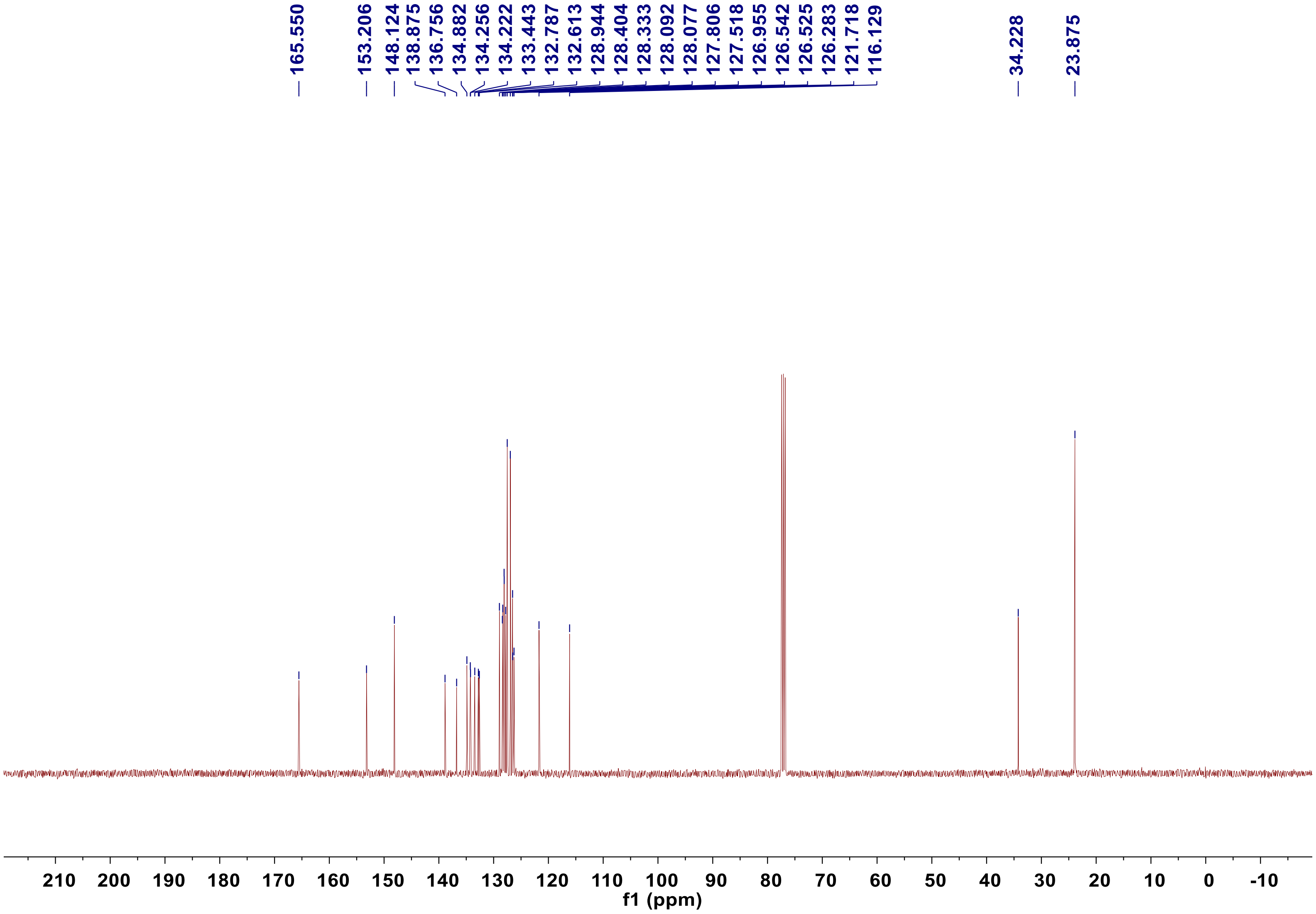

^{1}H NMR of **1g-8**

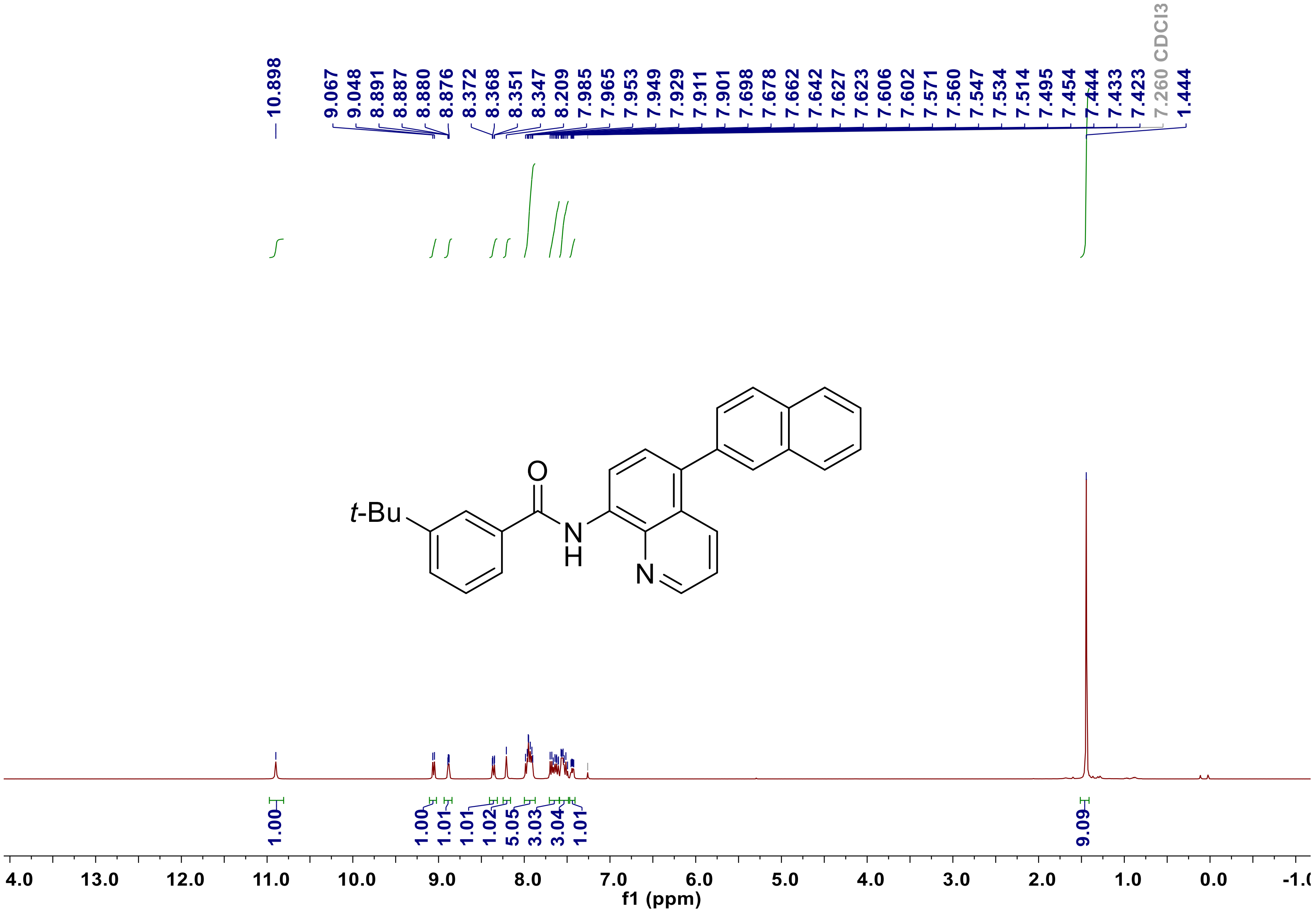

10.898
9.067
9.048
8.891
8.887
8.880
8.876
8.372
8.368
8.351
8.347
8.209
7.985
7.965
7.953
7.949
7.929
7.911
7.901
7.698
7.678
7.662
7.642
7.627
7.623
7.606
7.602
7.571
7.560
7.547
7.534
7.514
7.495
7.454
7.444
7.433
7.423
7.260 CDCl3
1.444
O
t-Bu
N
H
N
1.00
1.00
1.01
1.01
1.02
5.05
3.03
3.04
1.01
9.09
14.0
13.0
12.0
11.0
10.0
9.0
8.0
7.0
6.0
5.0
4.0
3.0
2.0
1.0
0.0
-1.0
f1 (ppm)

^{13}C NMR of **1g-8**

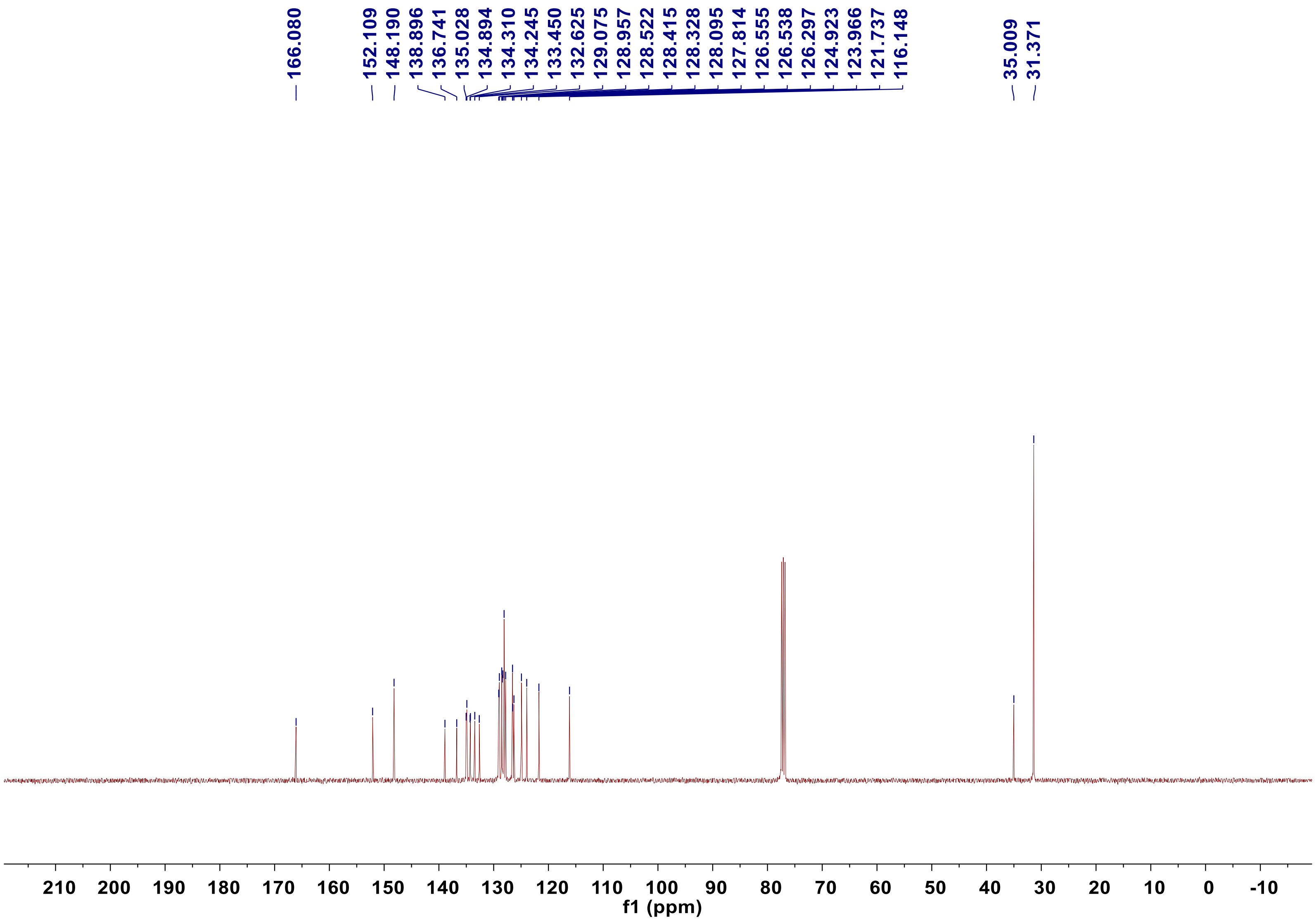

^{1}H NMR of **1g-9**

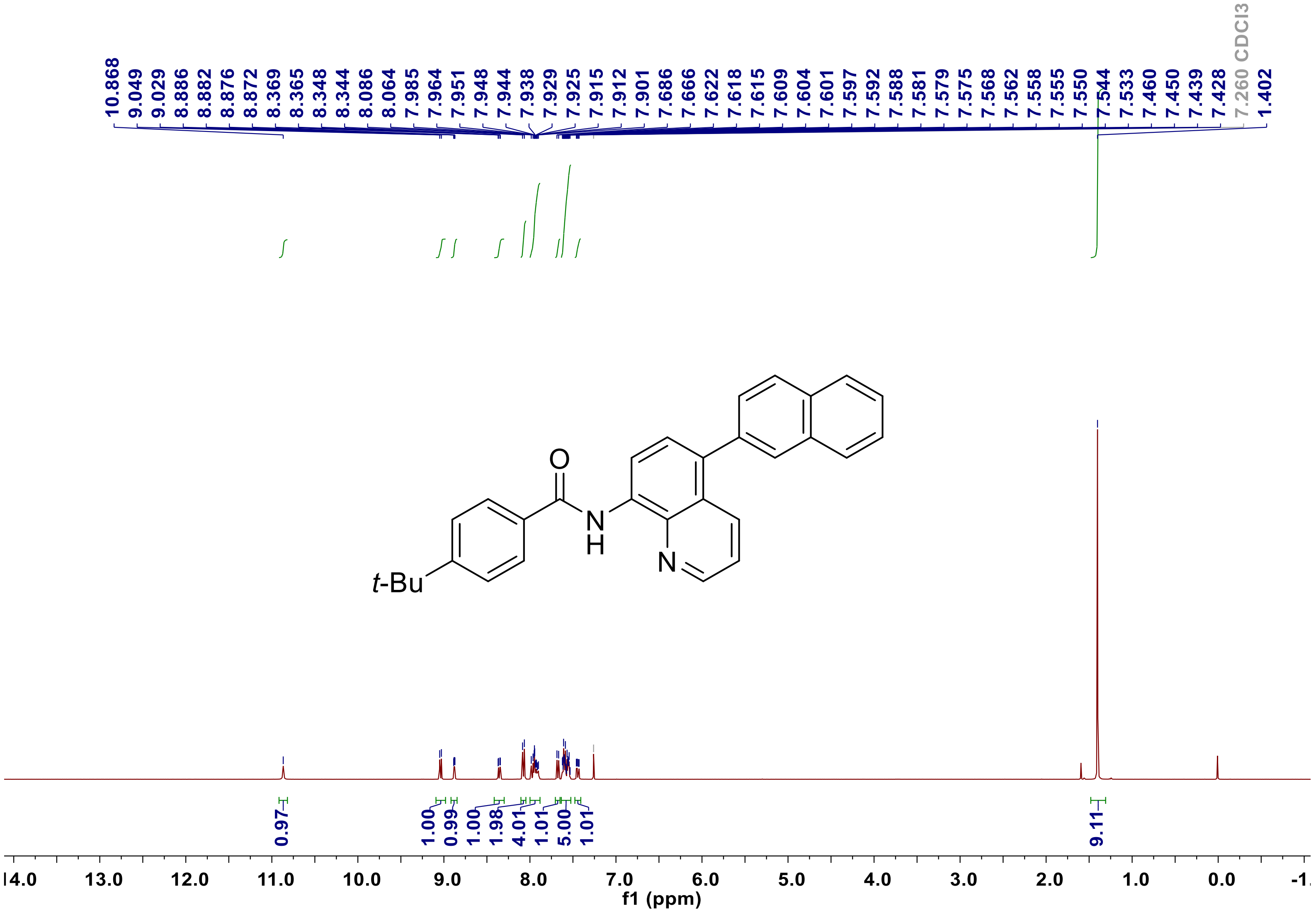

10.868
9.049
9.029
8.886
8.882
8.876
8.872
8.369
8.365
8.348
8.344
8.086
8.064
7.985
7.964
7.951
7.948
7.944
7.938
7.929
7.925
7.915
7.912
7.901
7.686
7.666
7.622
7.618
7.615
7.609
7.604
7.601
7.597
7.592
7.588
7.581
7.579
7.575
7.568
7.562
7.558
7.555
7.550
7.544
7.533
7.460
7.450
7.439
7.428
7.260 CDCl3
1.402
O
N
H
N
t-Bu
0.97
1.00
0.99
1.00
1.98
4.01
1.01
5.00
1.01
9.11
14.0
13.0
12.0
11.0
10.0
9.0
8.0
7.0
6.0
5.0
4.0
3.0
2.0
1.0
0.0
f1 (ppm)

^{13}C NMR of **1g-9**

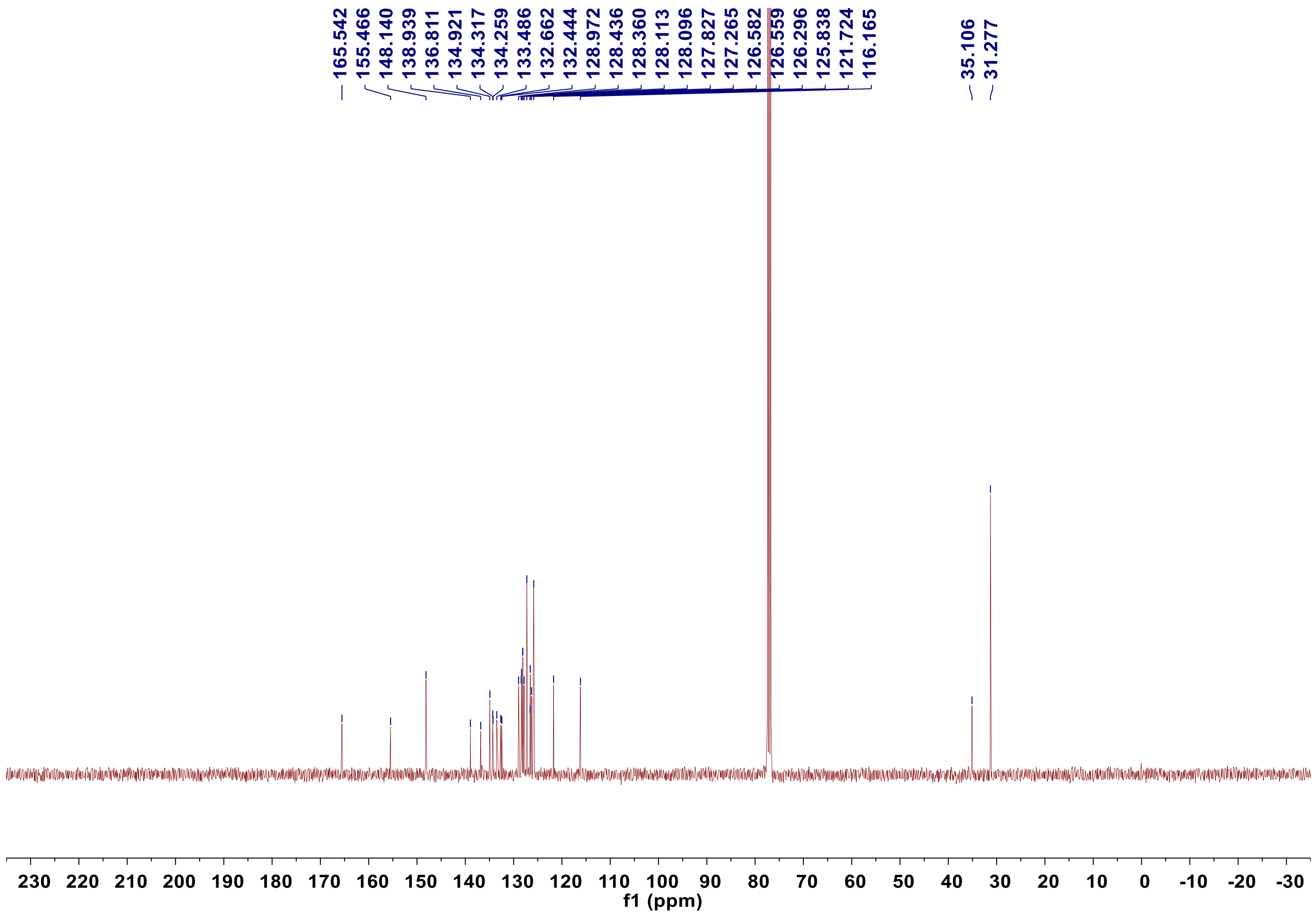

^{1}H NMR of **1g-10**

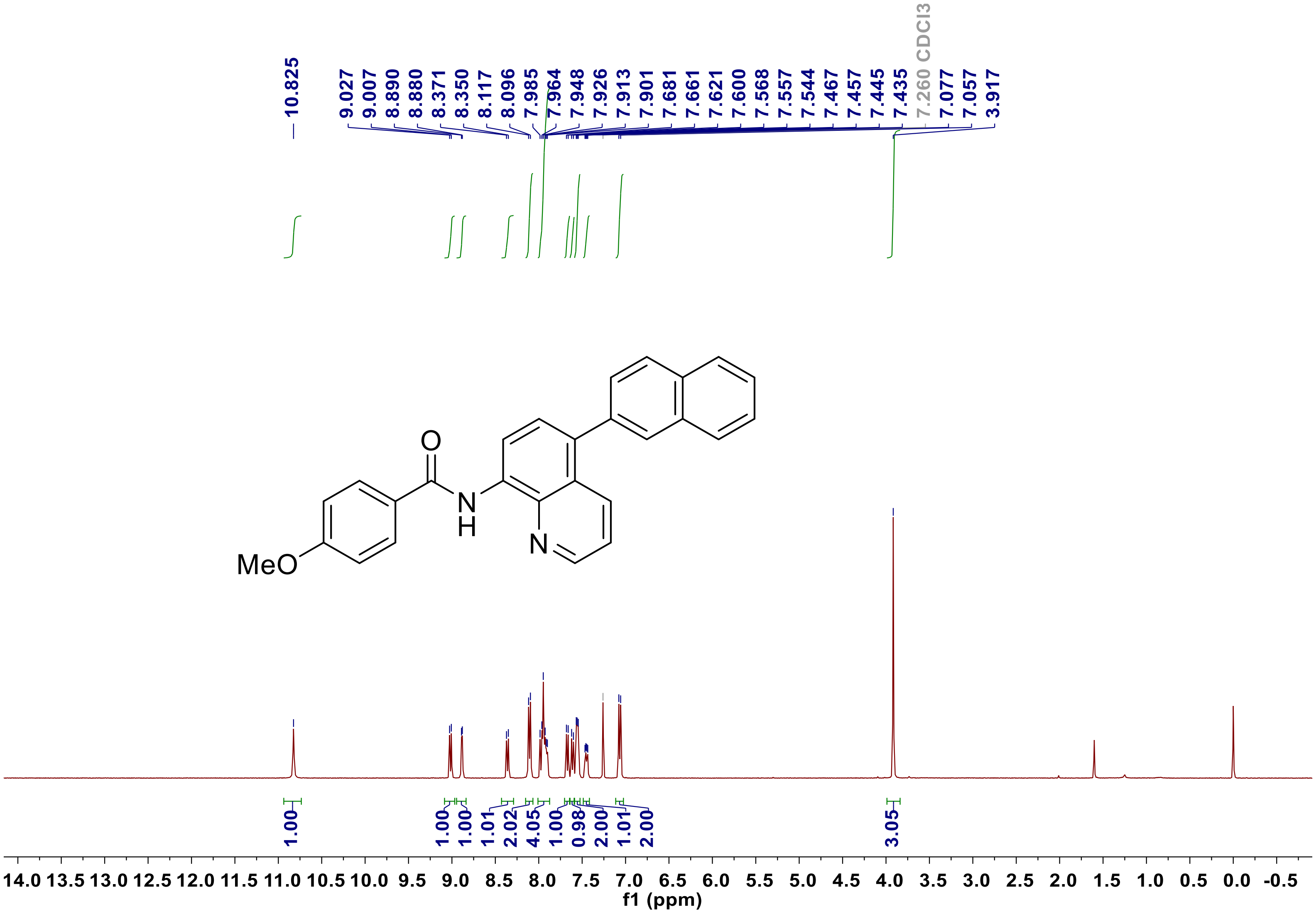

^{13}C NMR of **1g-10**

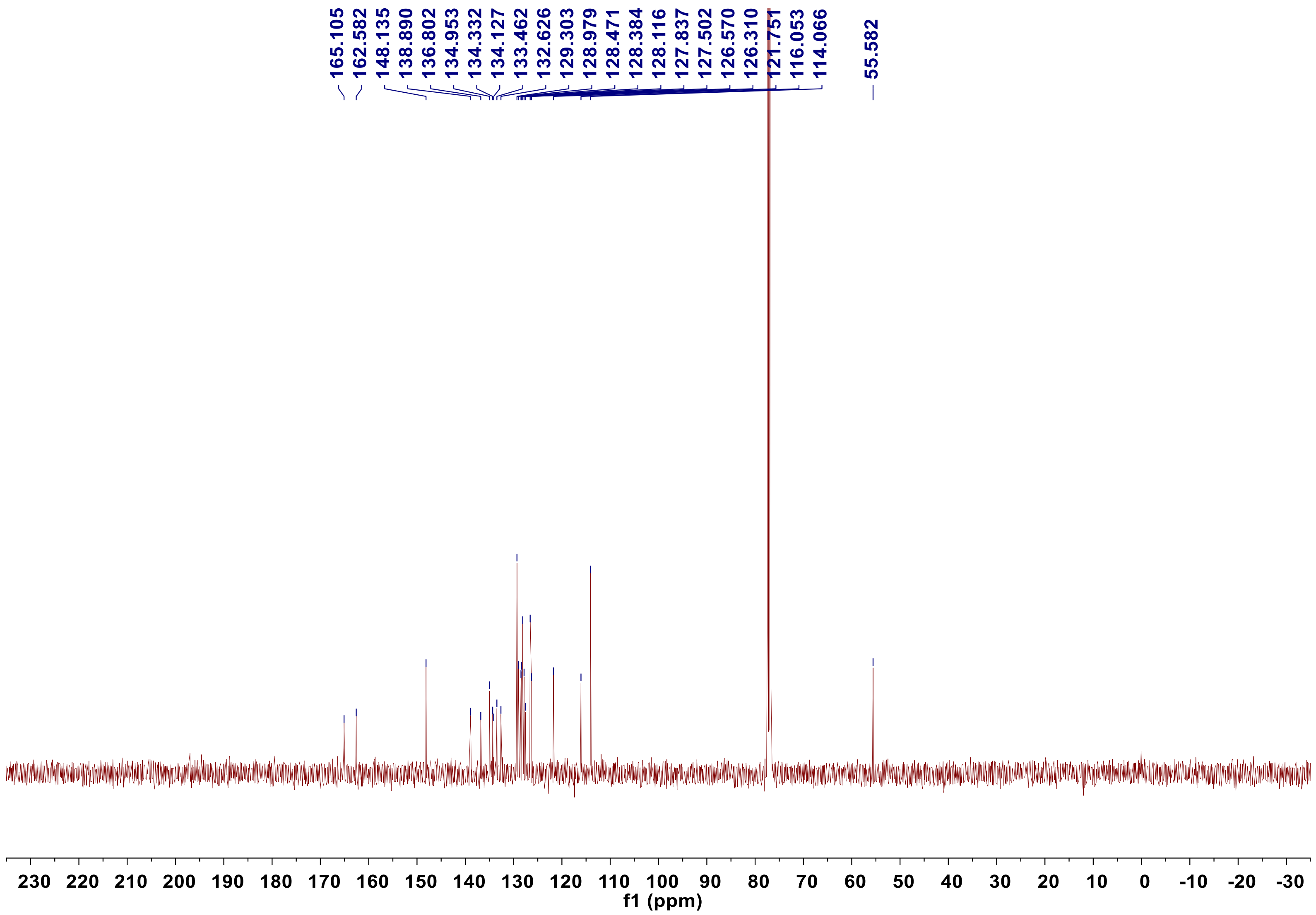

^{1}H NMR of **1g-11**

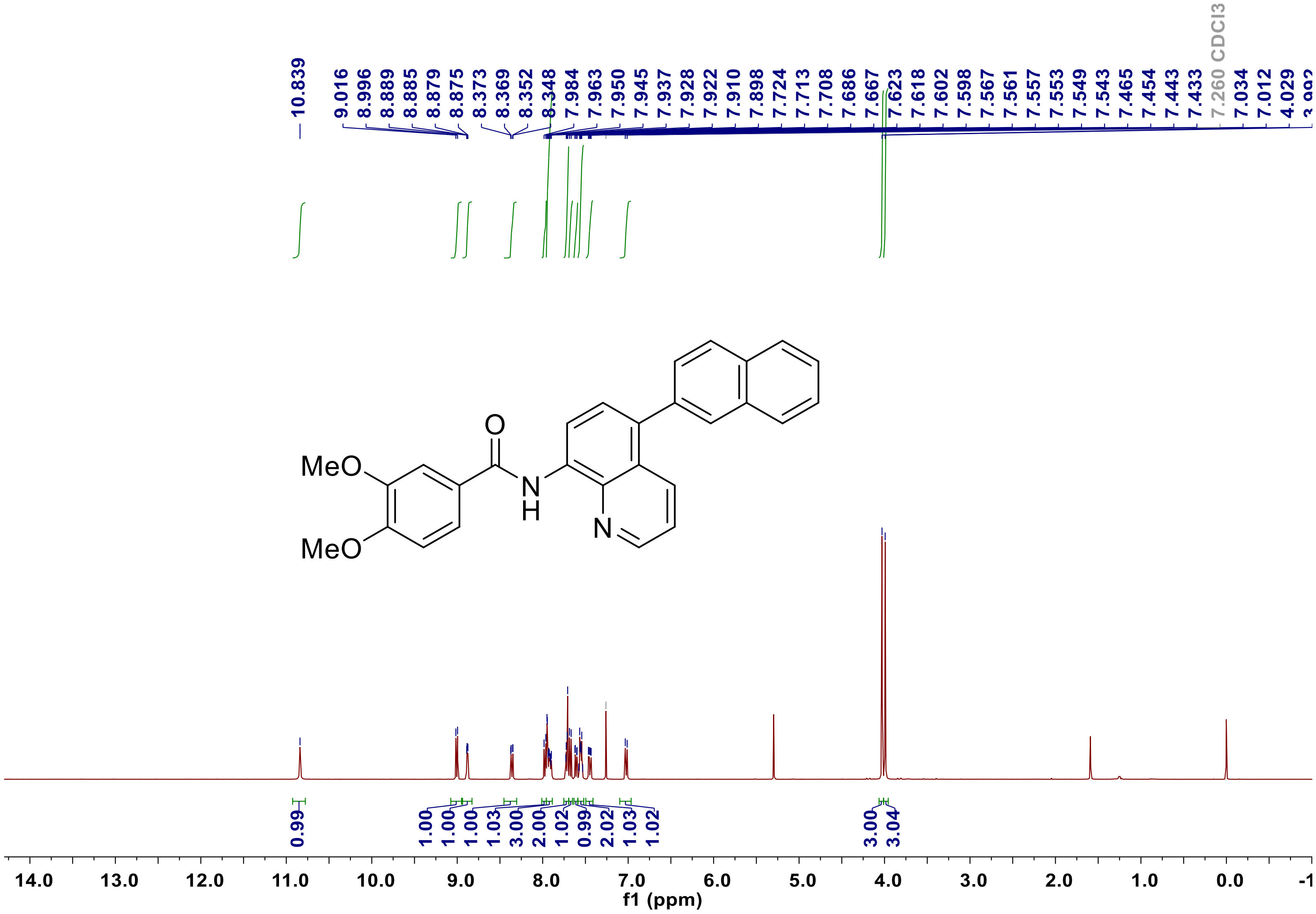

^{13}C NMR of **1g-11**

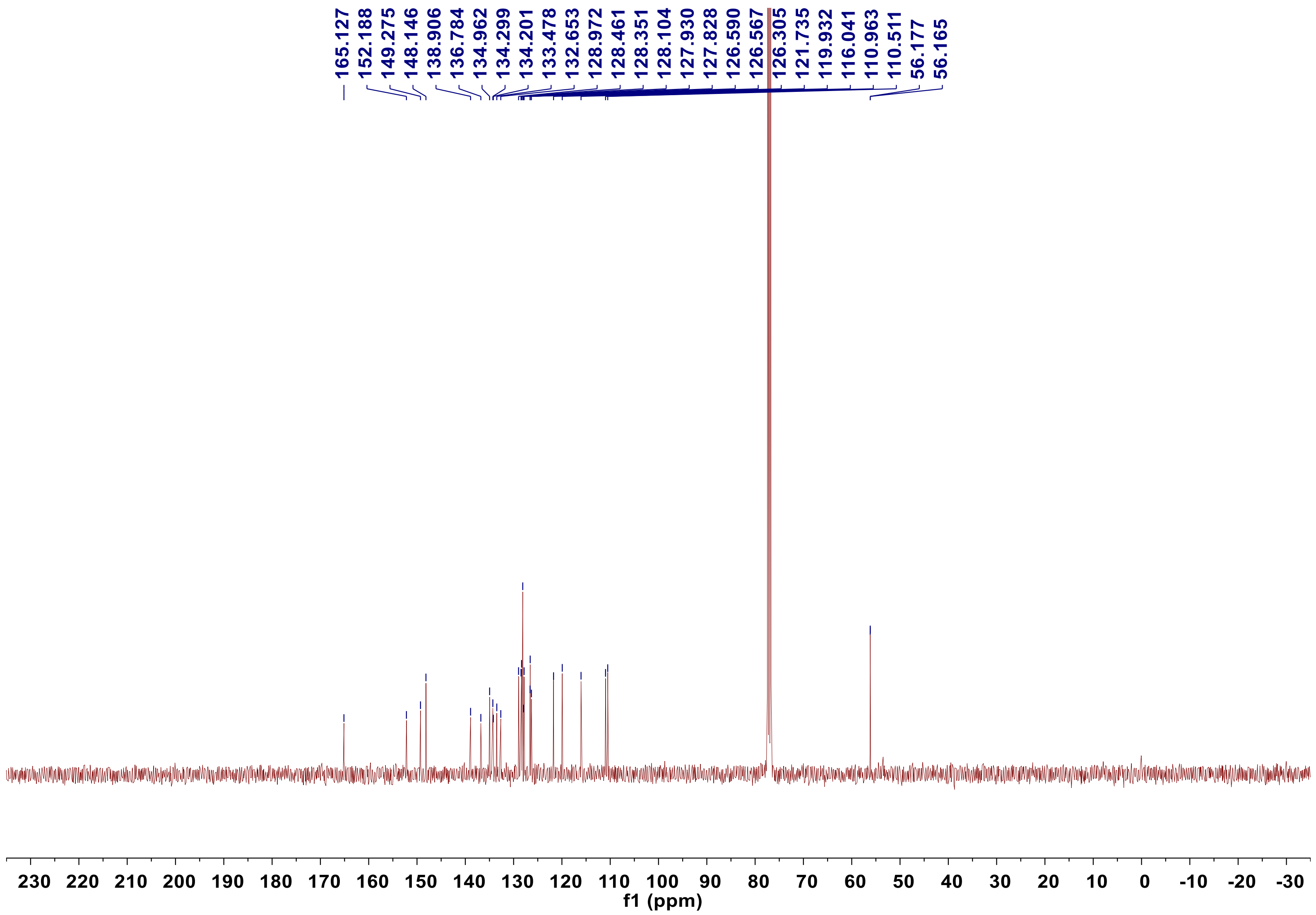

^{1}H NMR of **1g-12**

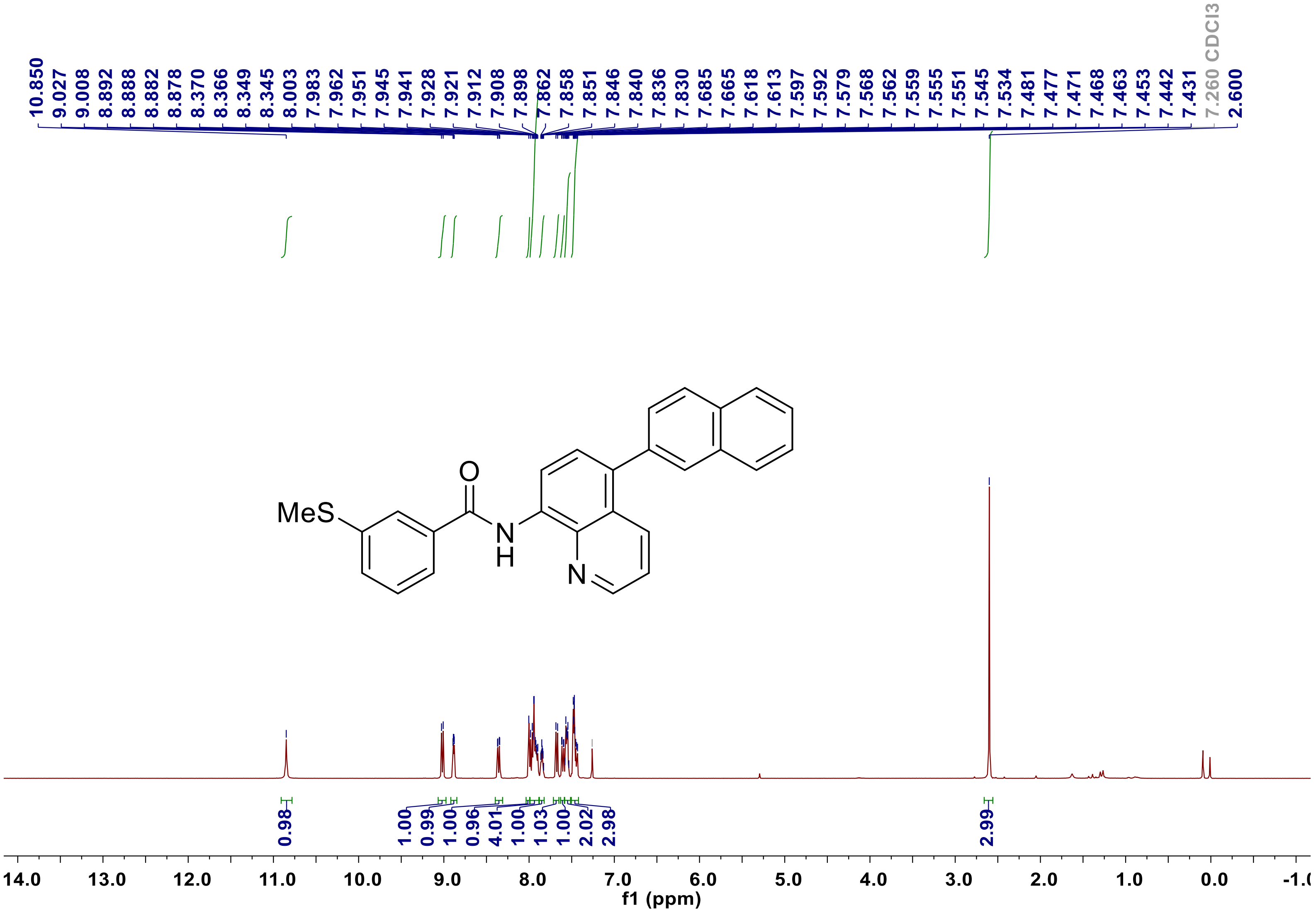

^{13}C NMR of **1g-12**

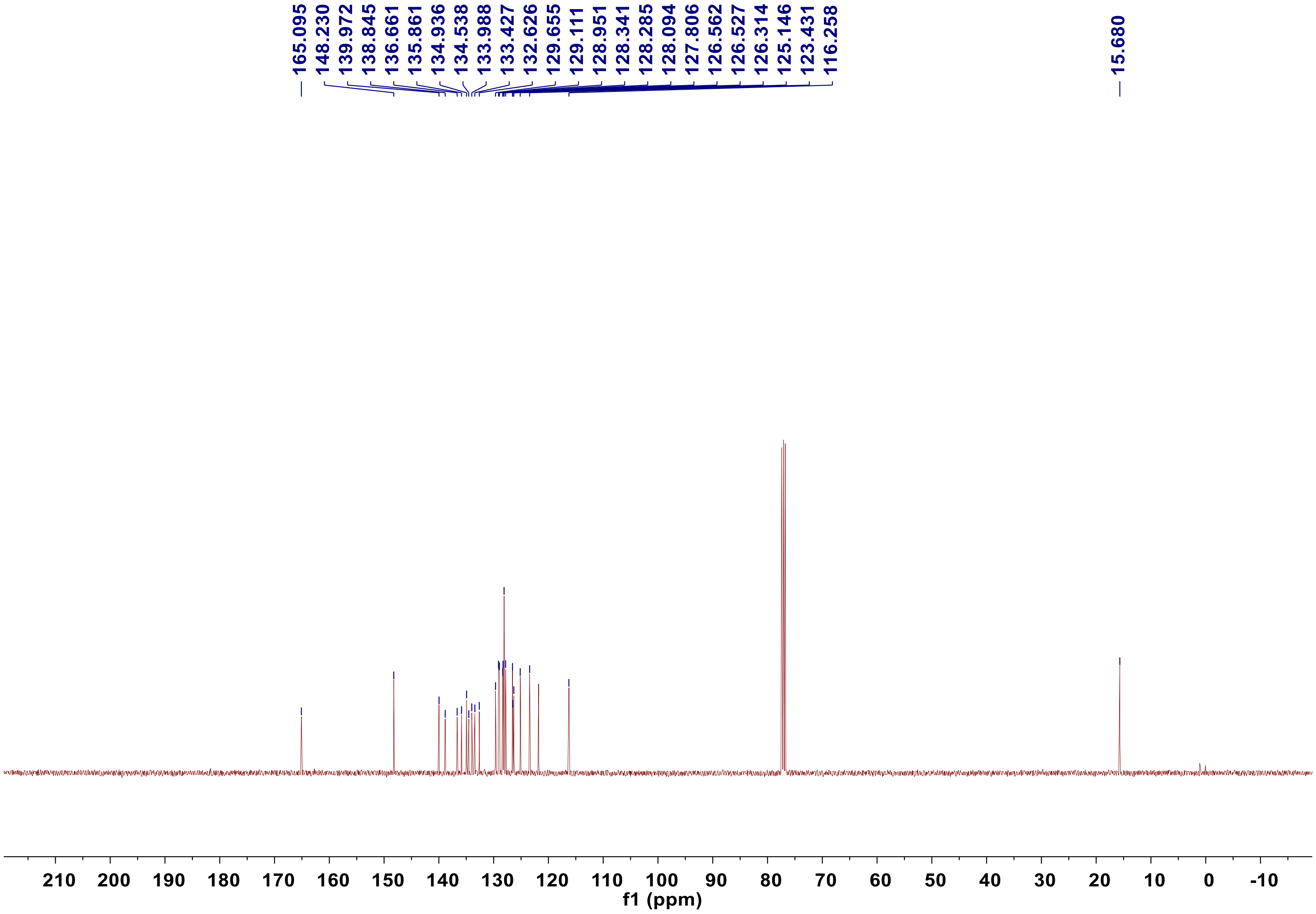

^{1}H NMR of **1g-13**

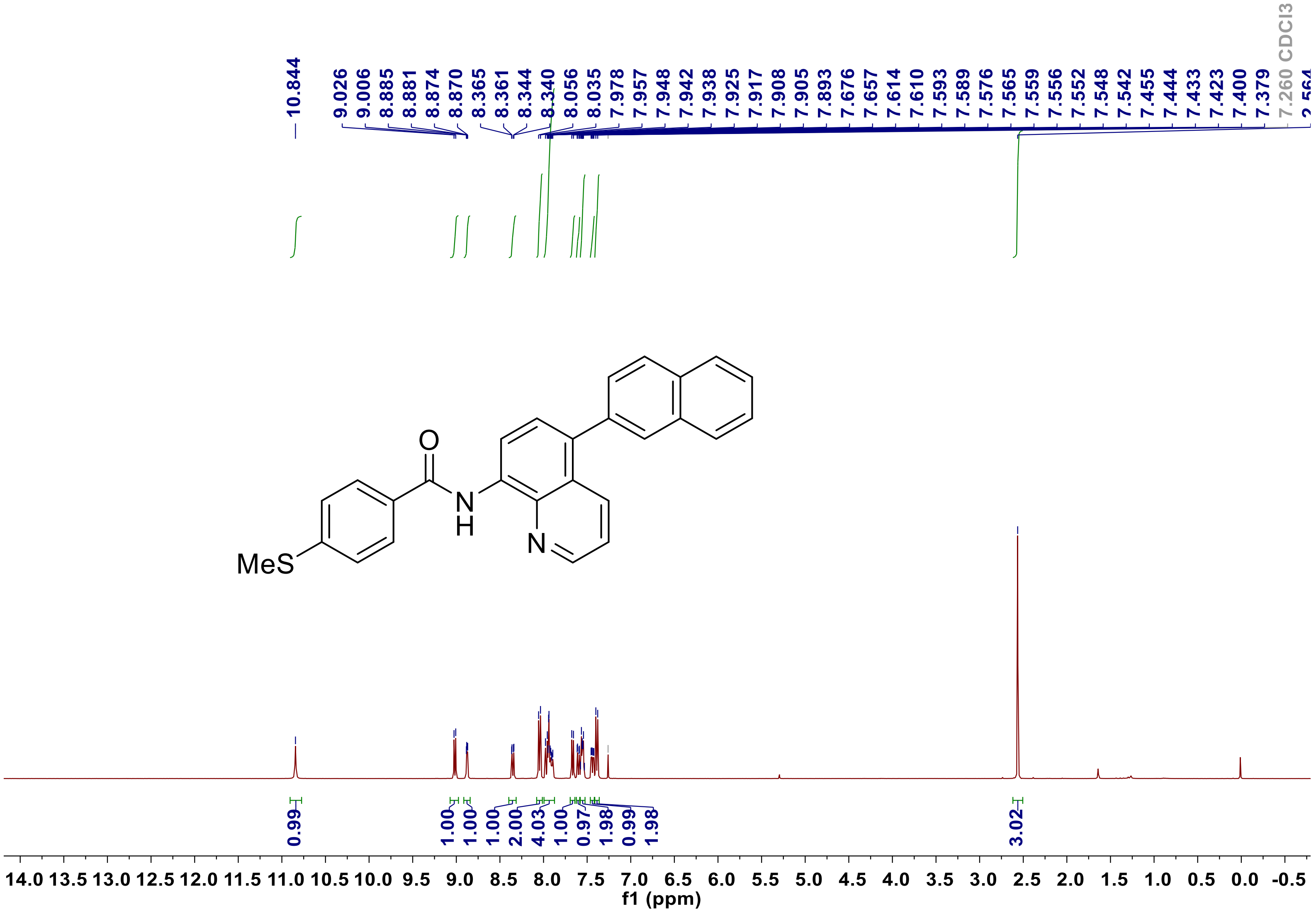

^{13}C NMR of **1g-13**

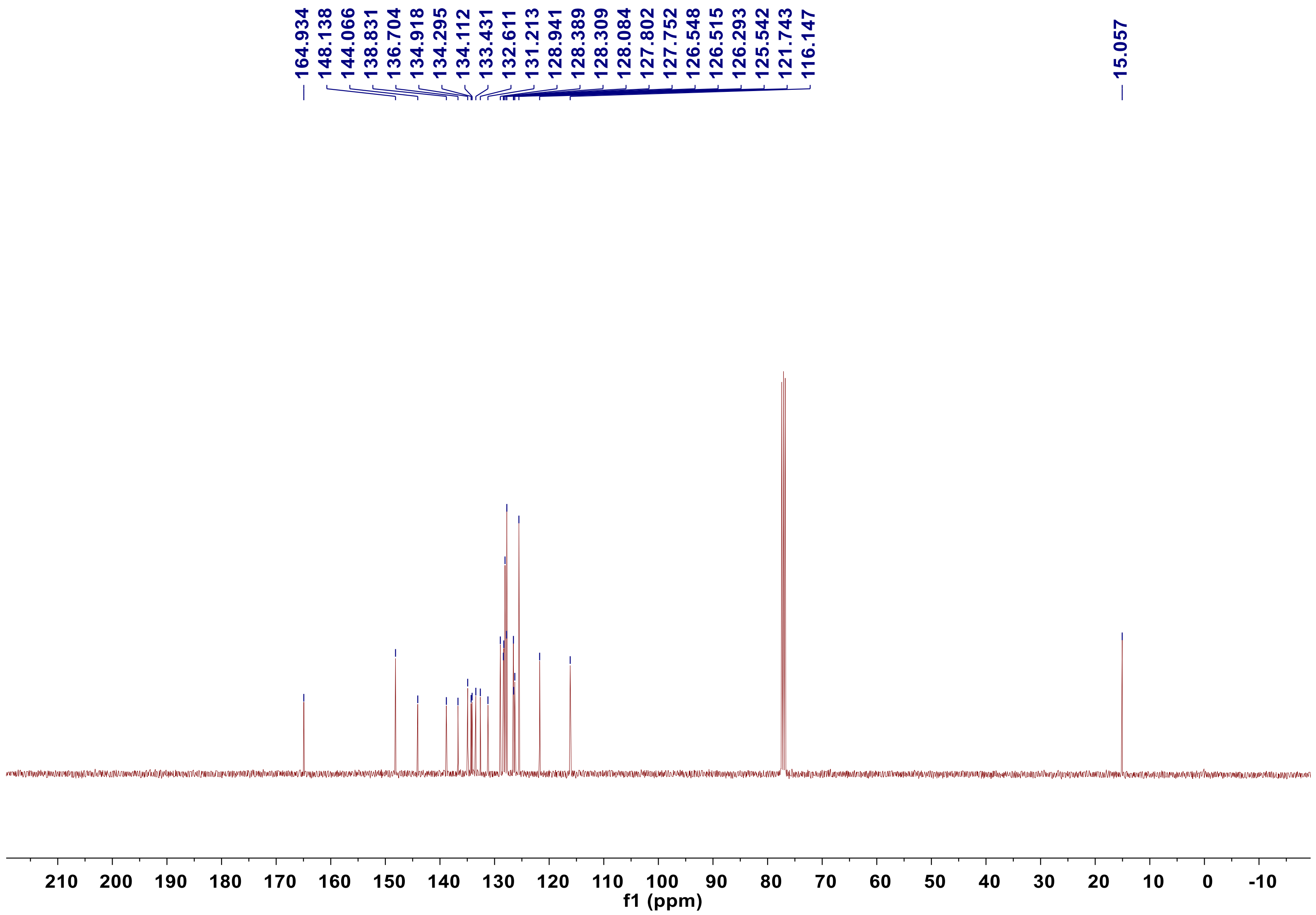

^{1}H NMR of **1g-14**

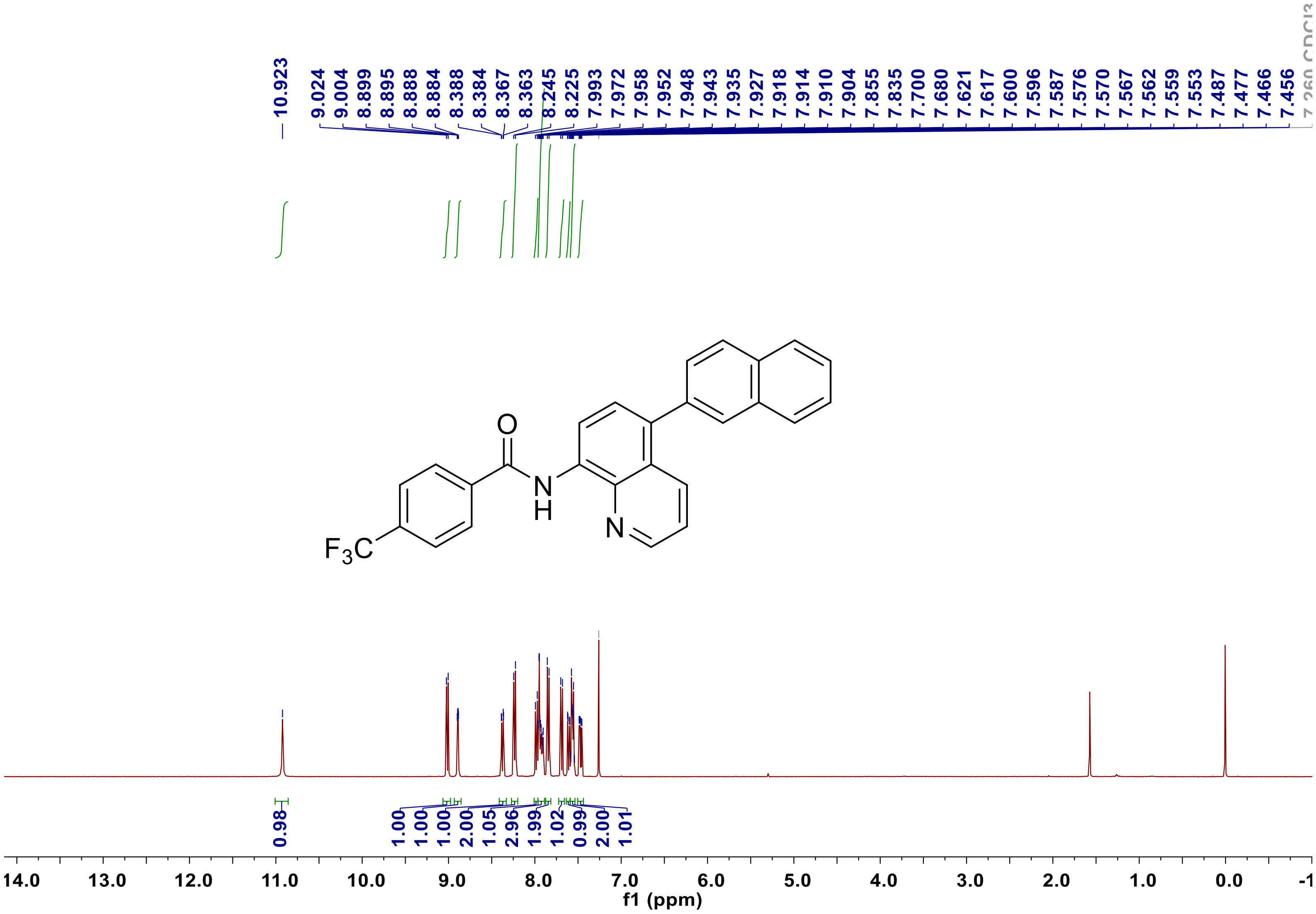

^{19}F NMR of **1g-14**

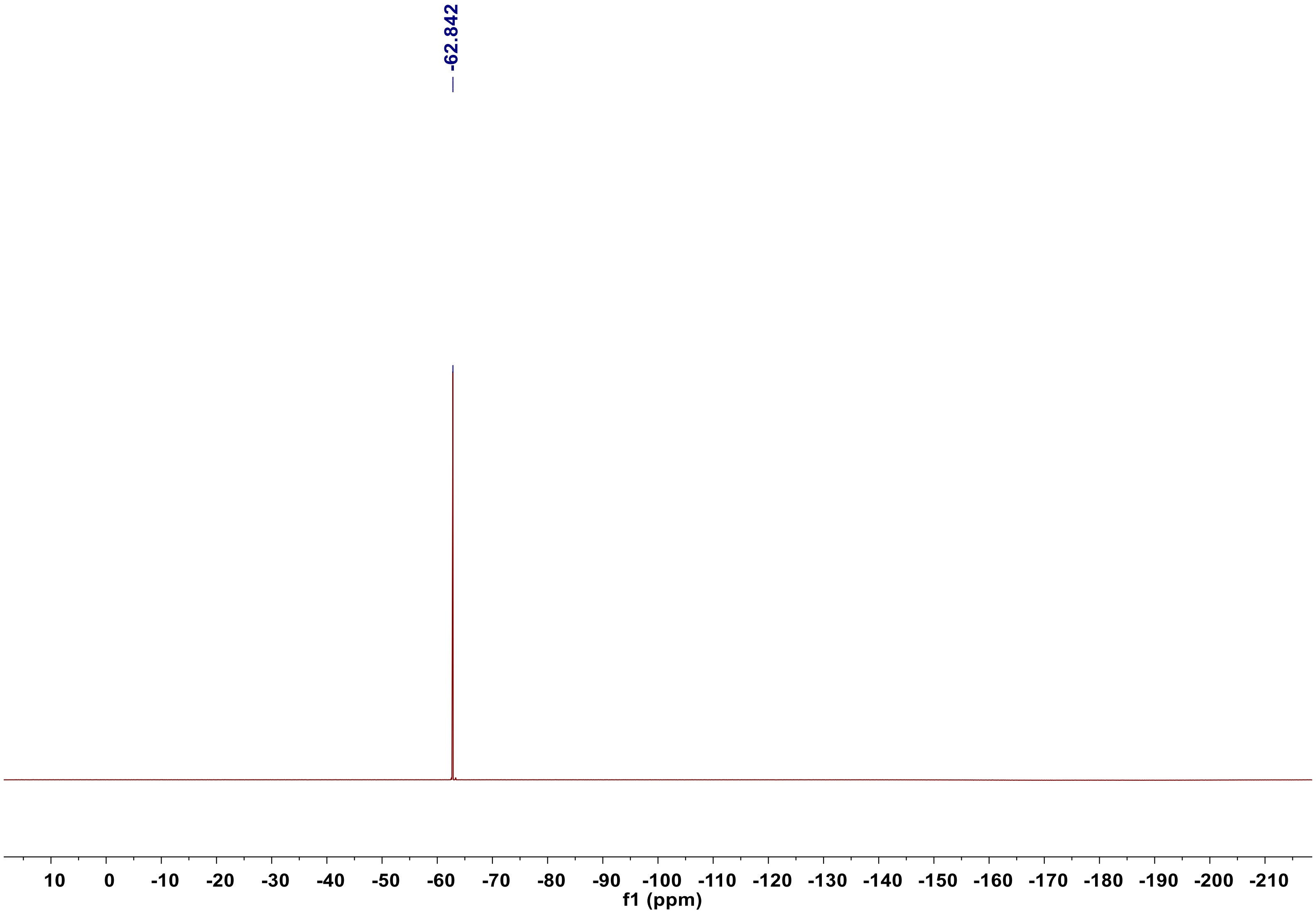

^{13}C NMR of **1g-14**

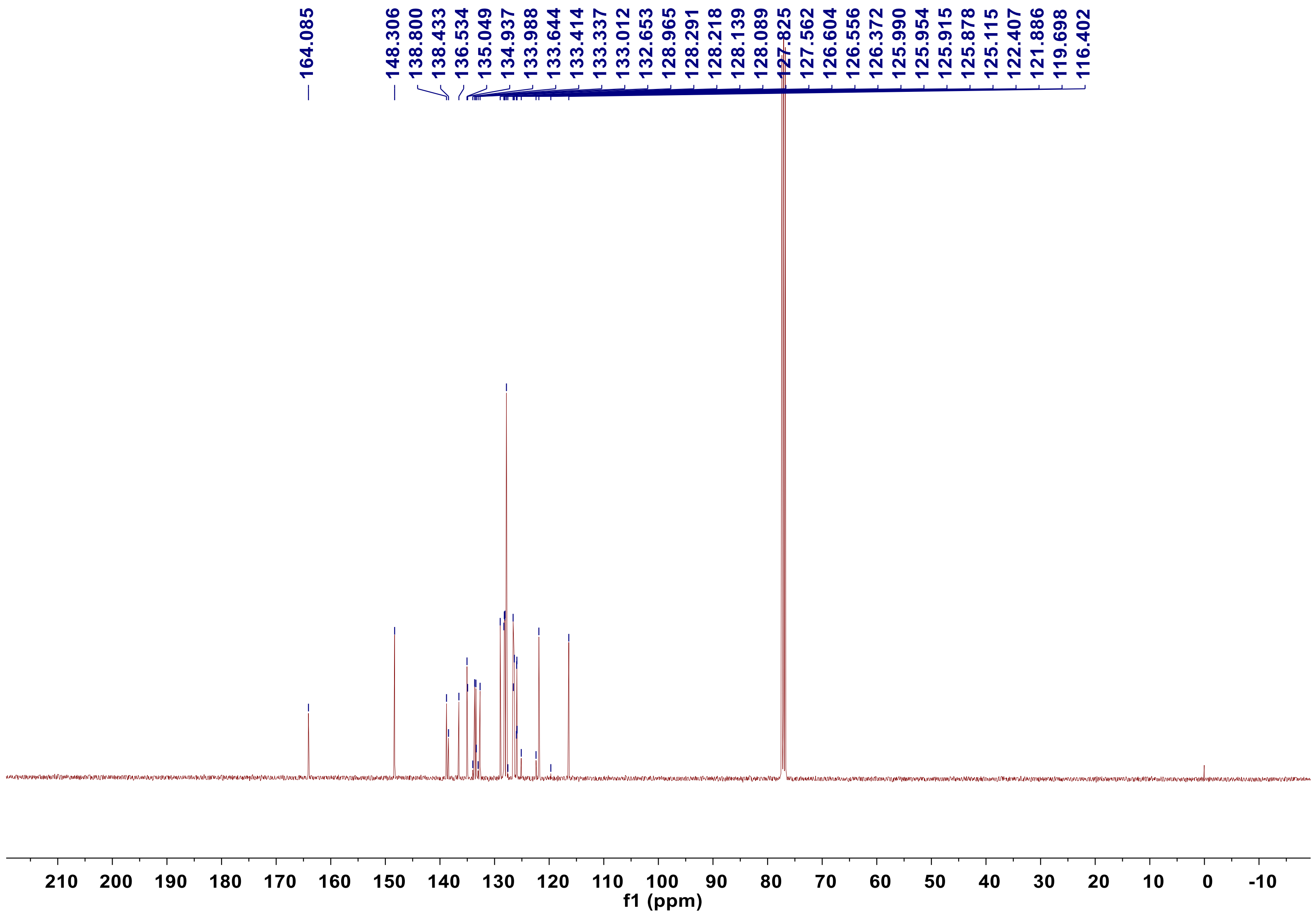

^{1}H NMR of **1g-15**

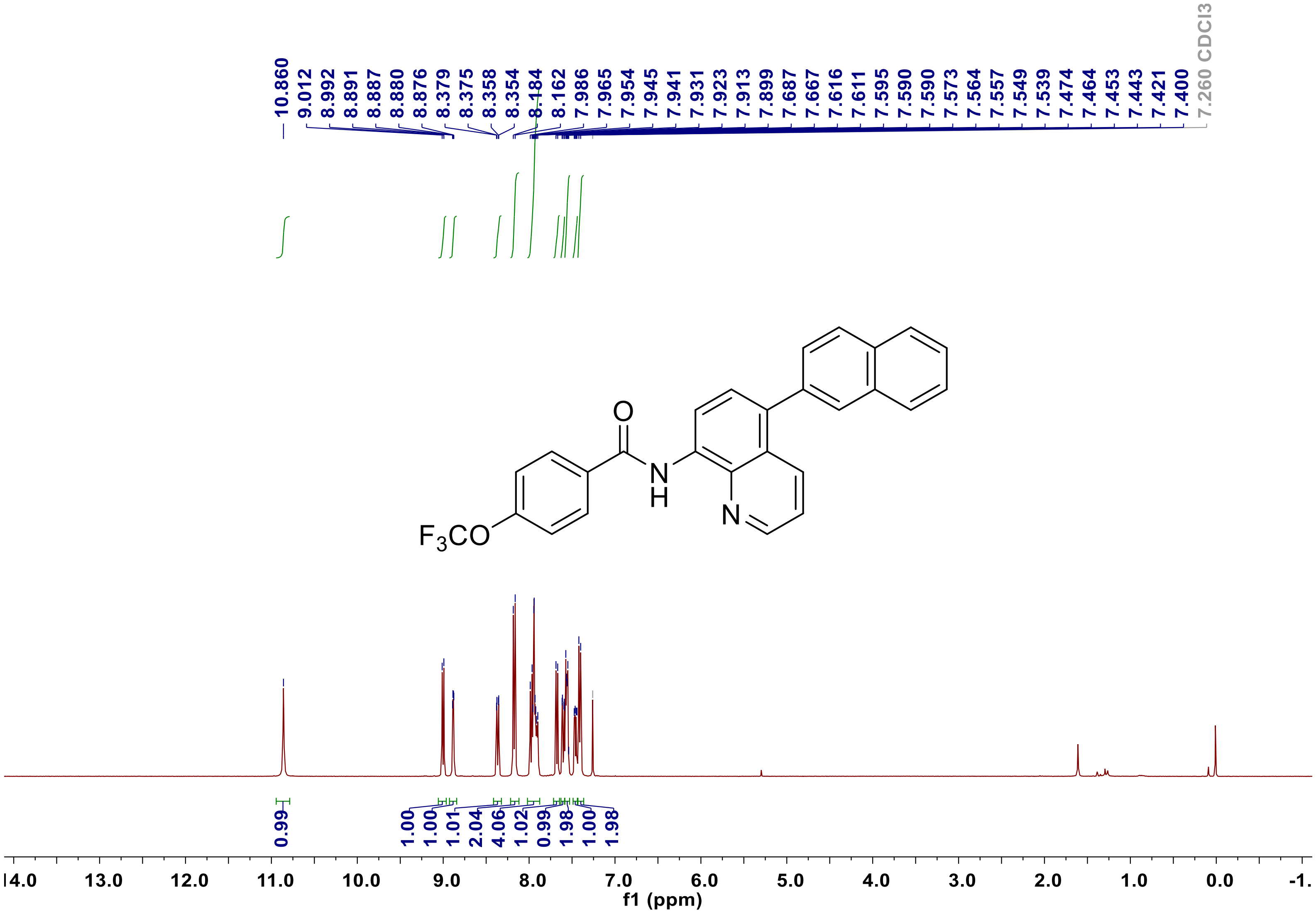

^{19}F NMR of **1g-15**

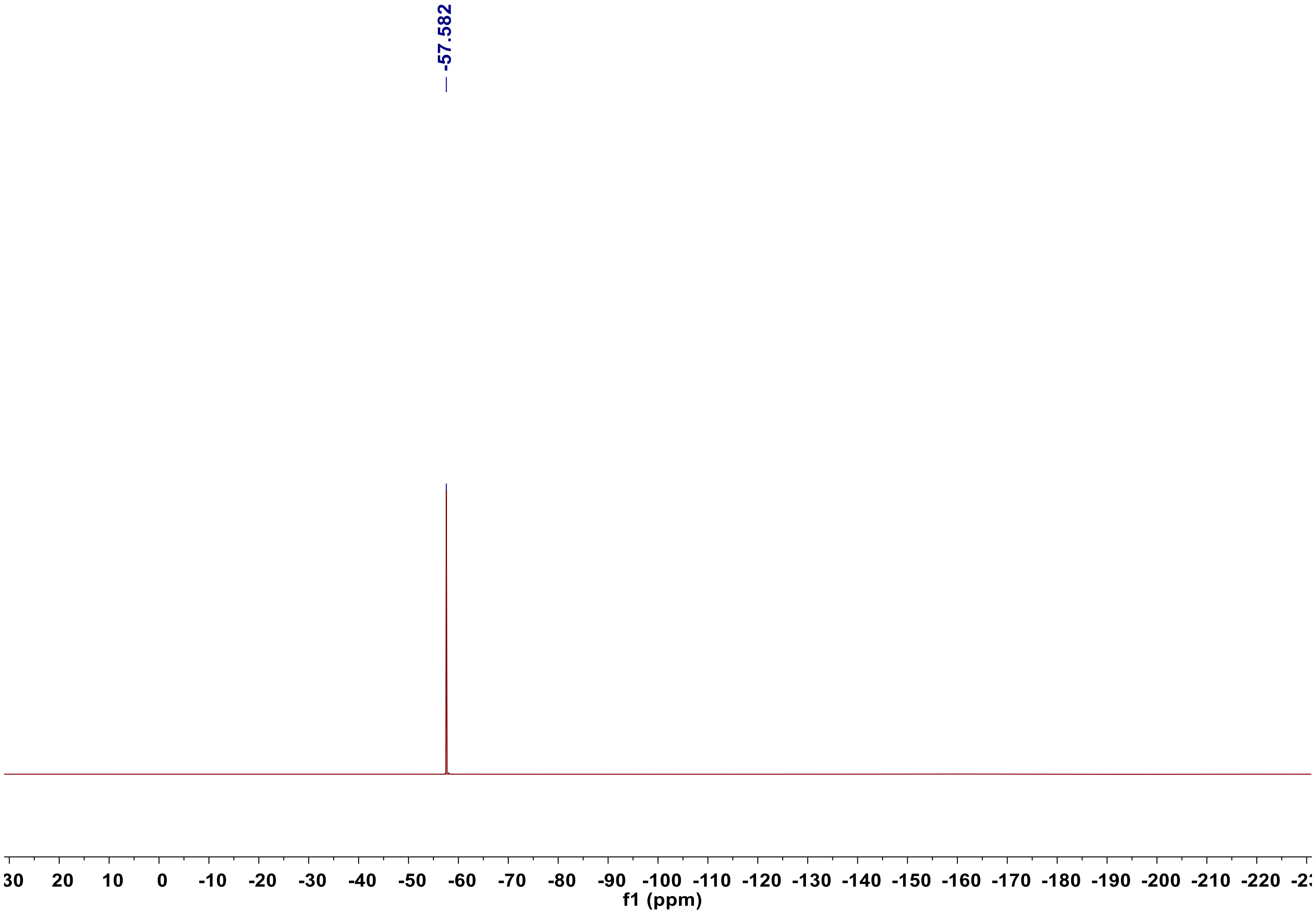

^{13}C NMR of **1g-15**

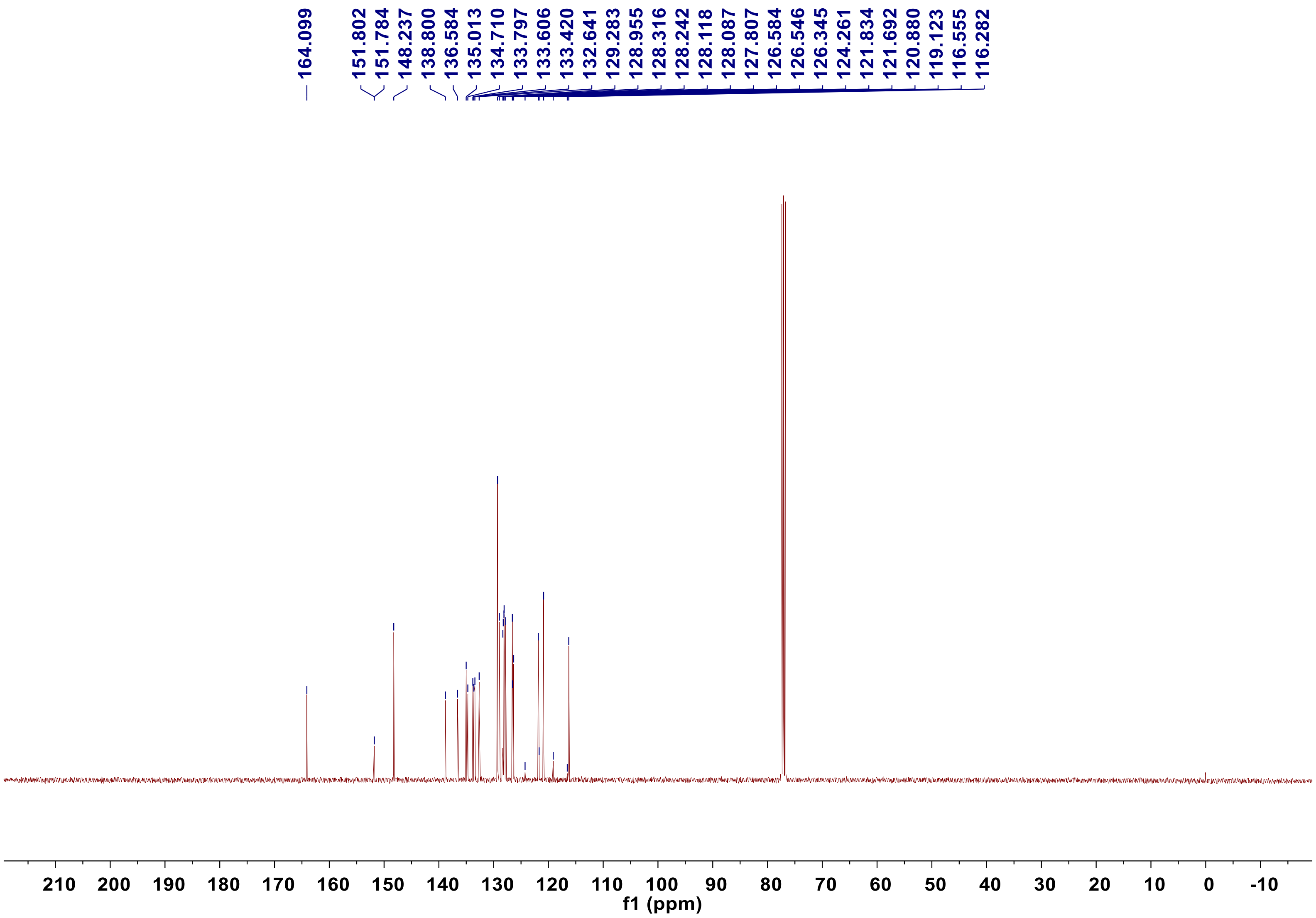

^{1}H NMR of **1g-16**

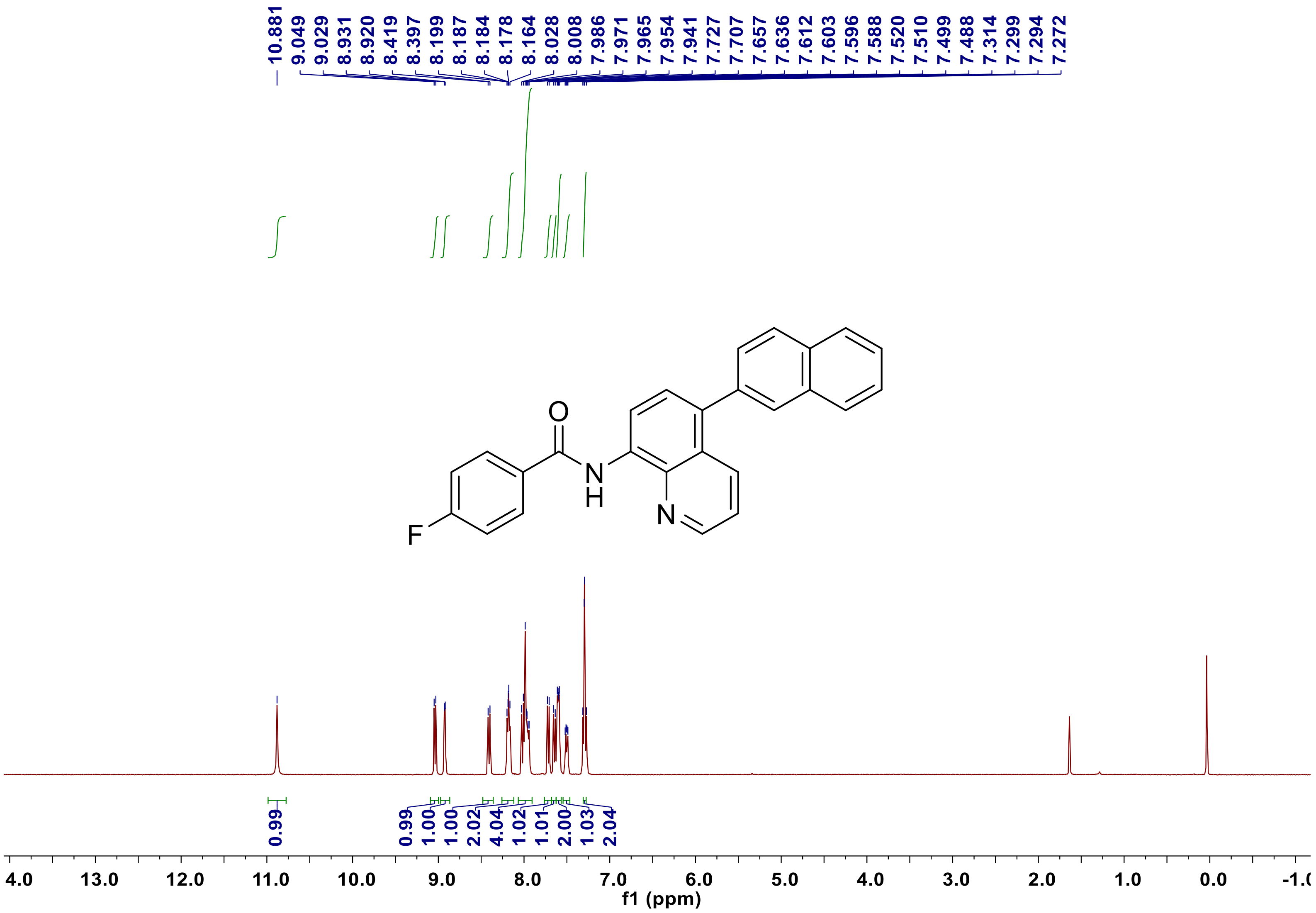

^{19}F NMR of **1g-16**

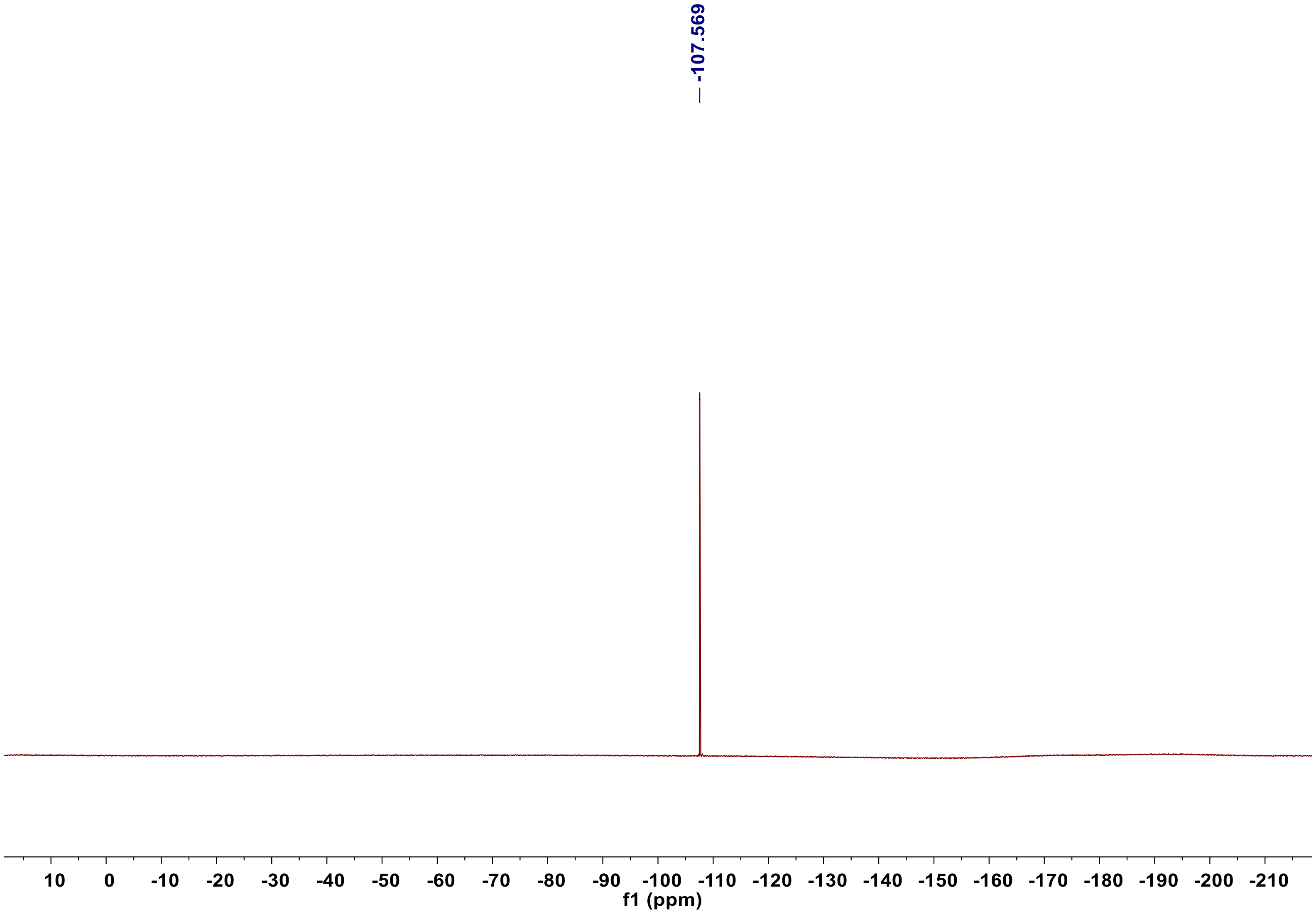

^{13}C NMR of **1g-16**

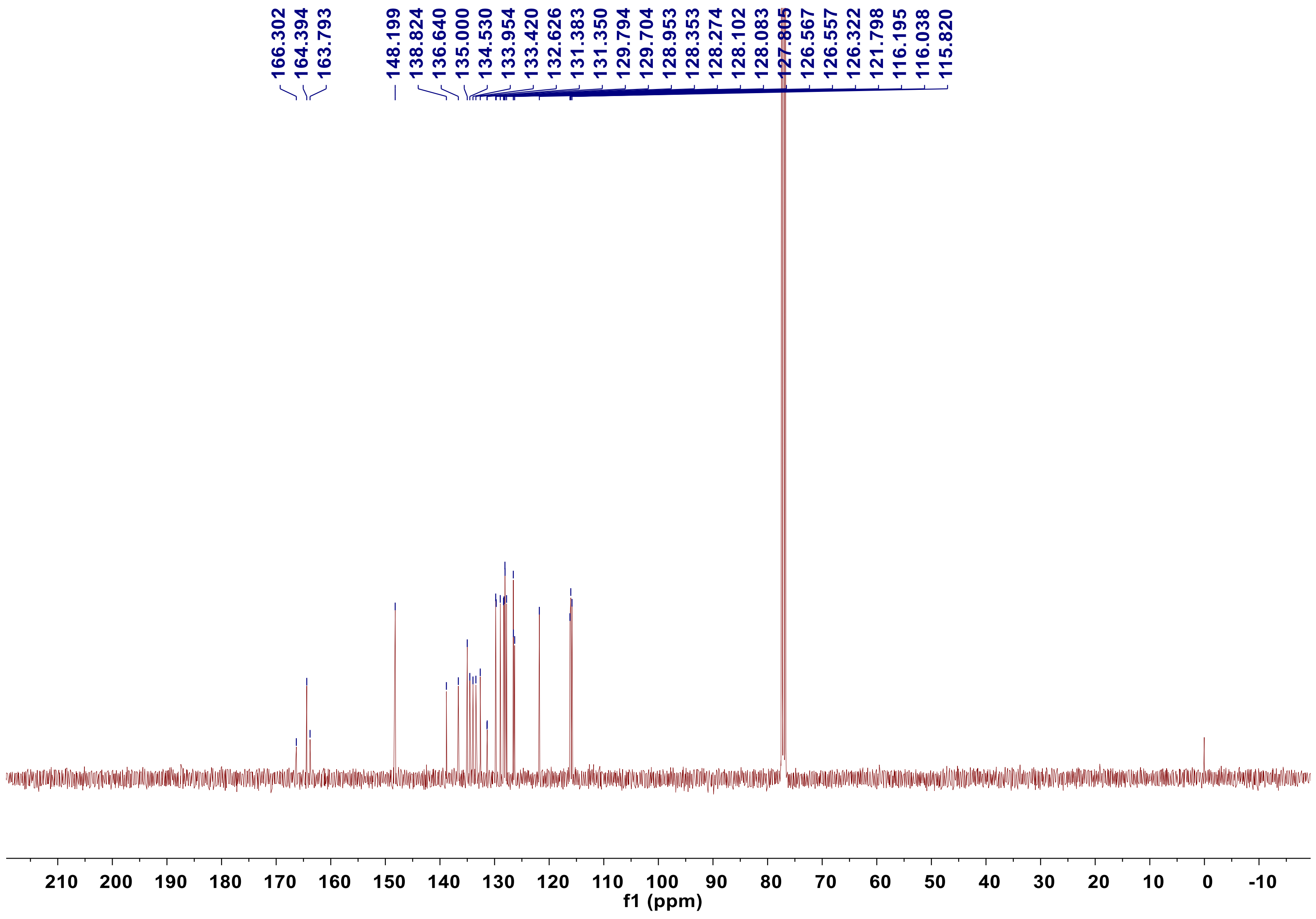

^{1}H NMR of **1g-17**

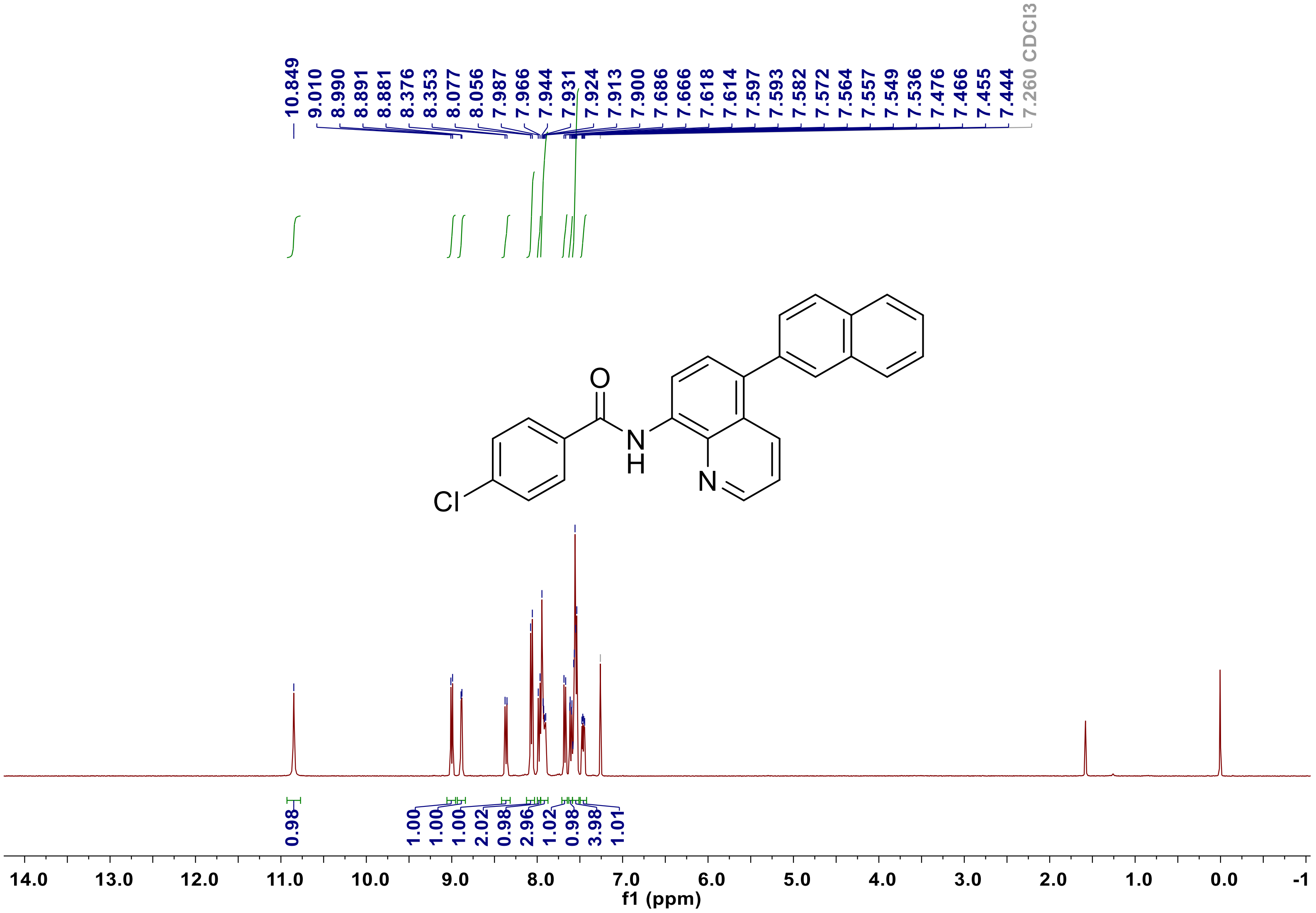

^{13}C NMR of **1g-17**

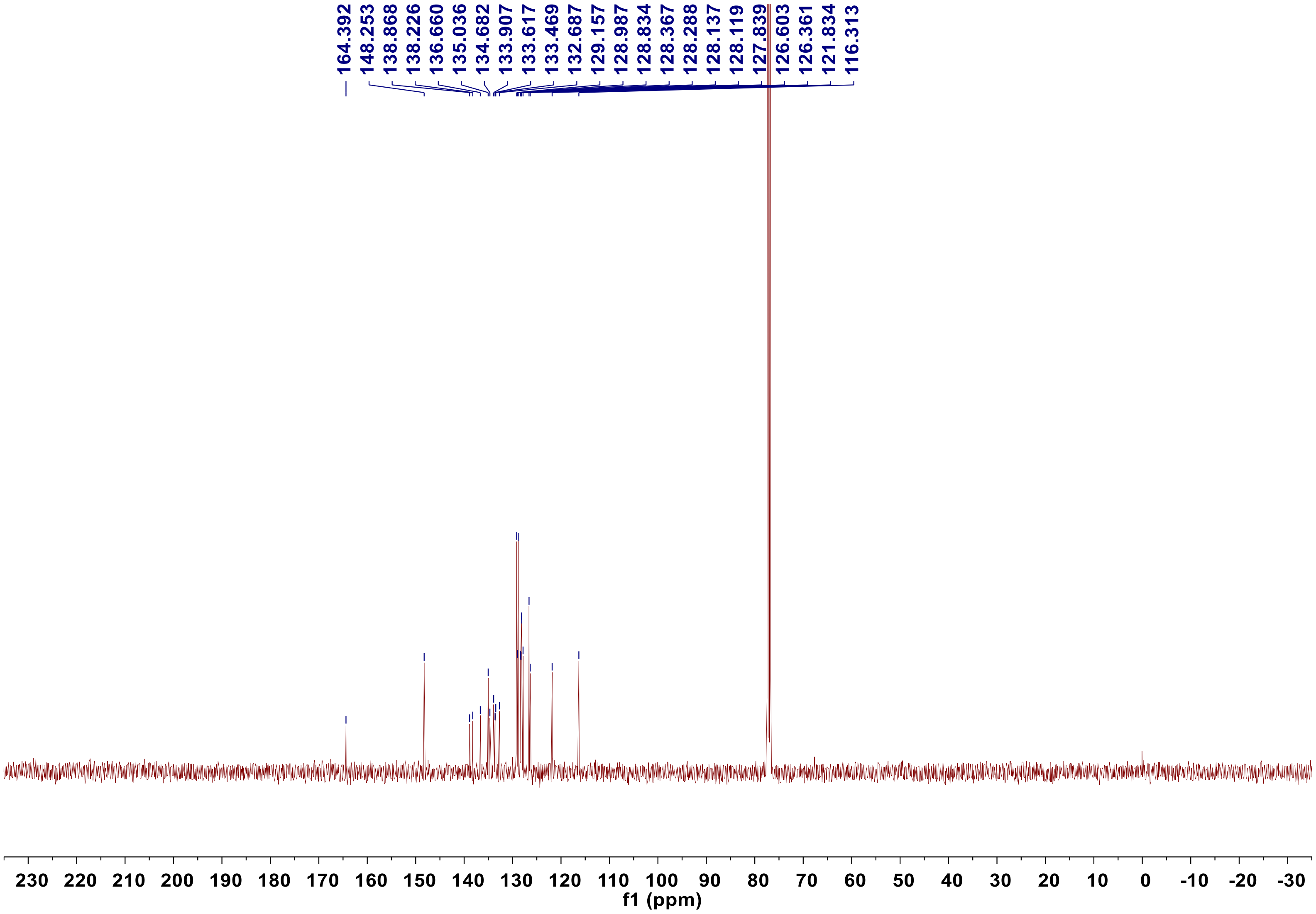

¹H NMR of **1g-18**

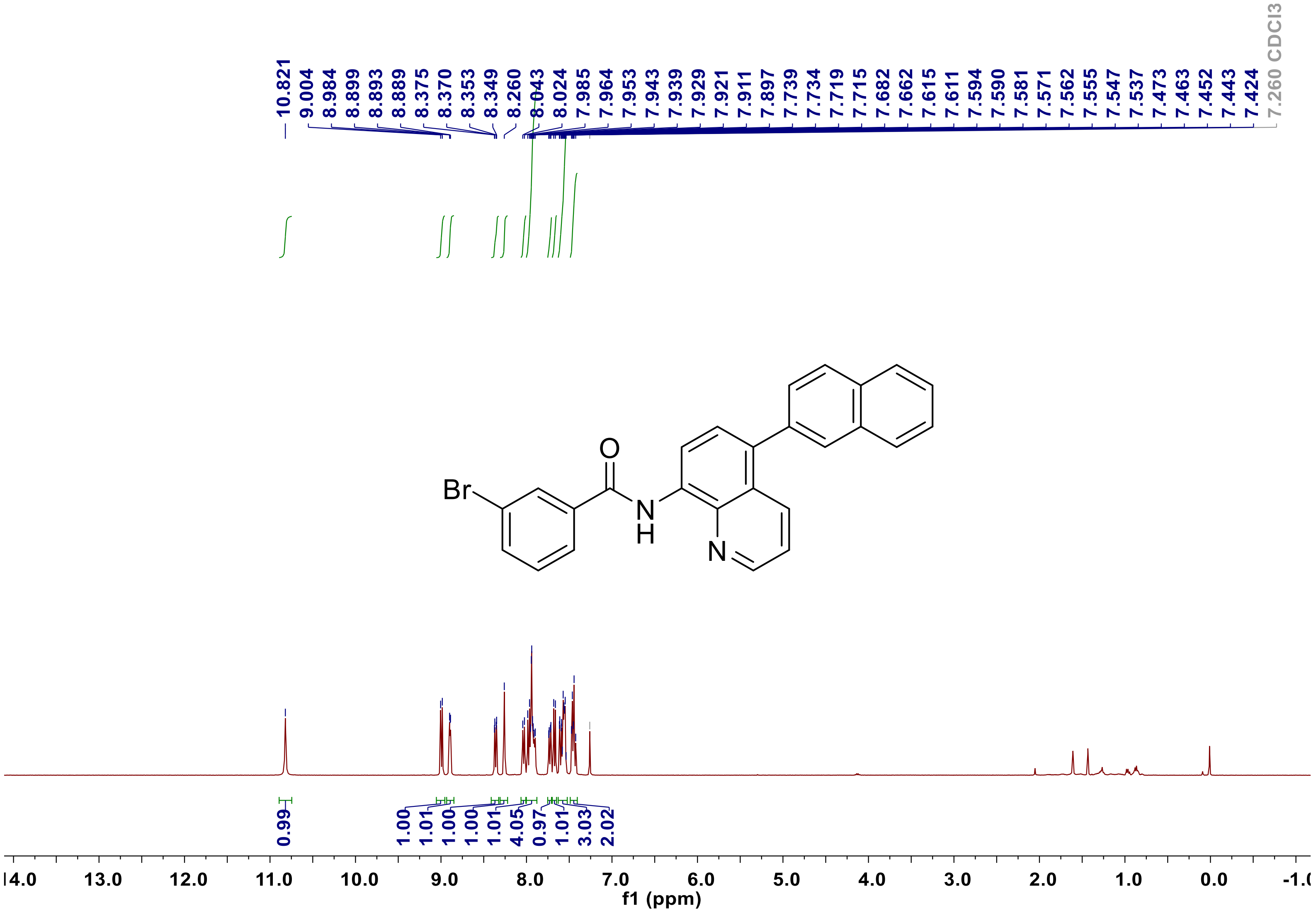

^{13}C NMR of **1g-18**

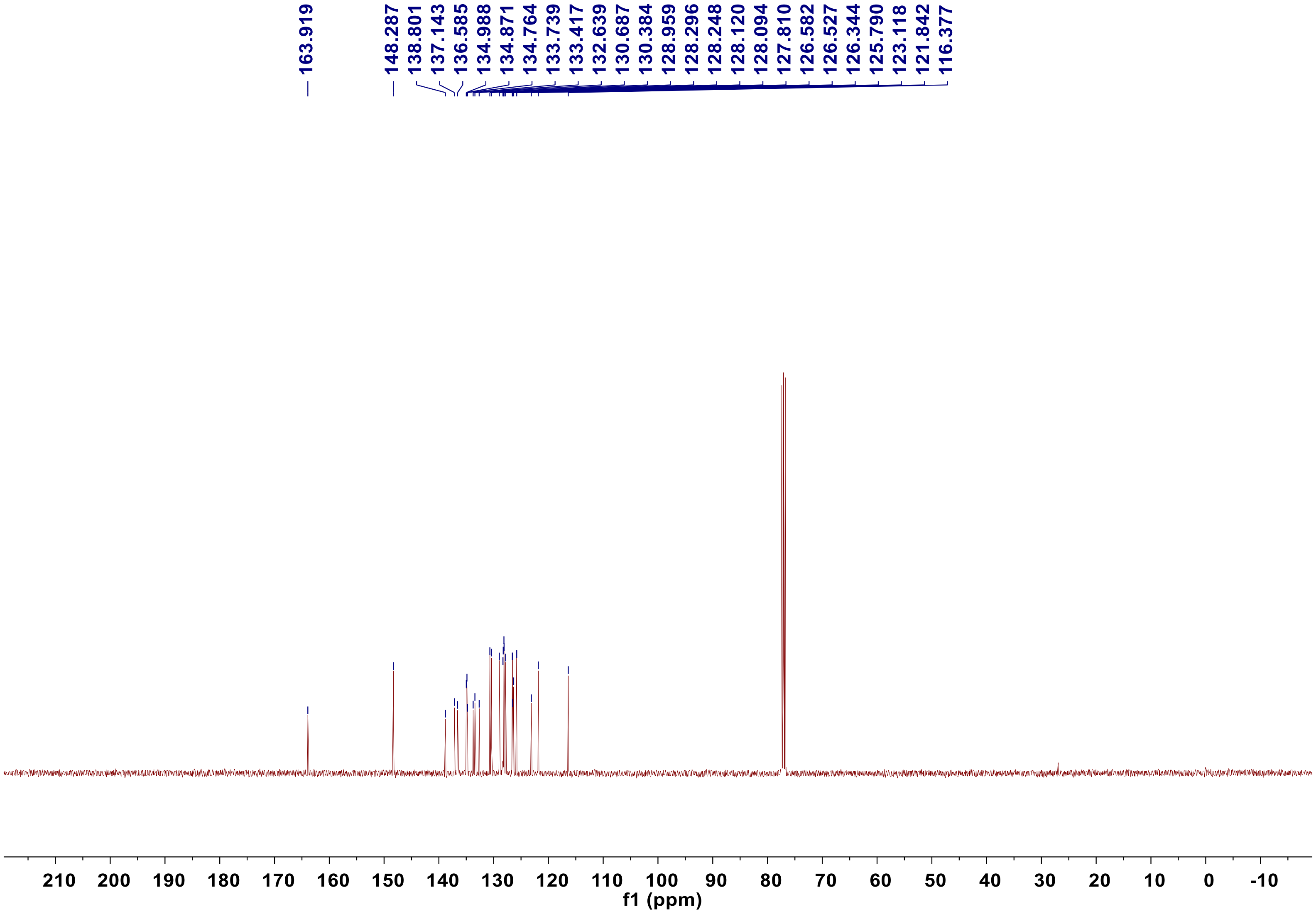

^{1}H NMR of **1g-19**

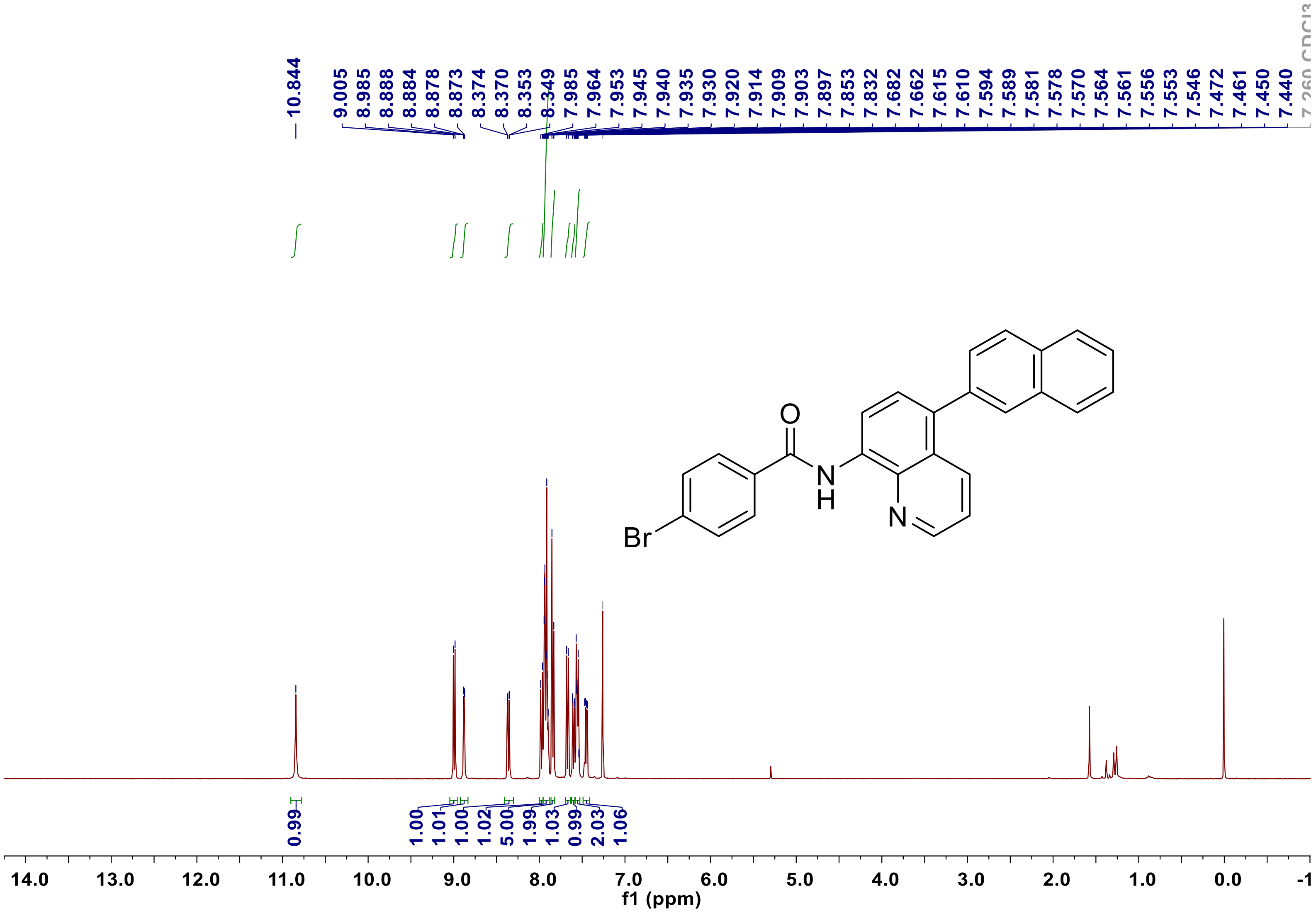

^{13}C NMR of **1g-19**

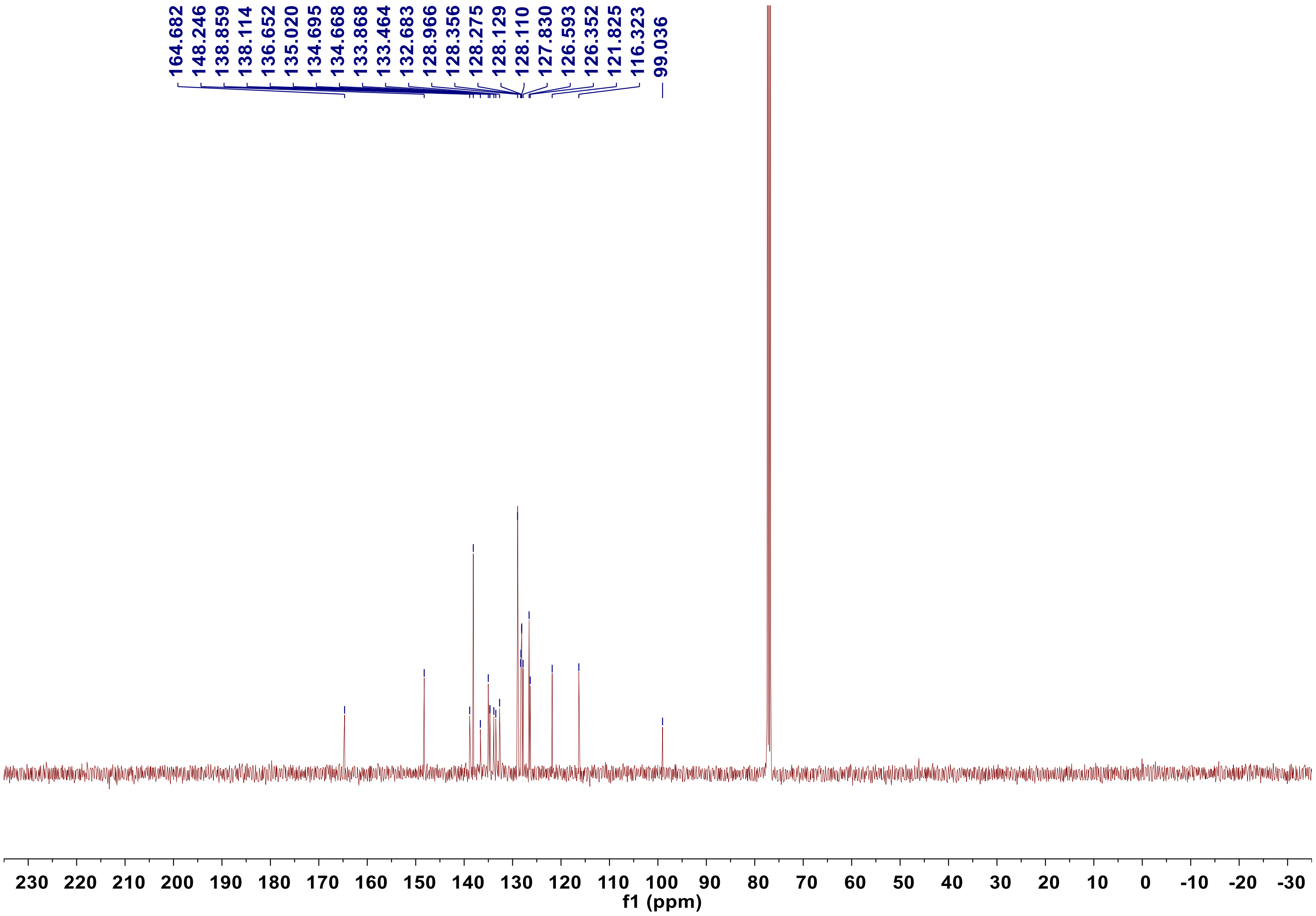

^{1}H NMR of **1g-20**

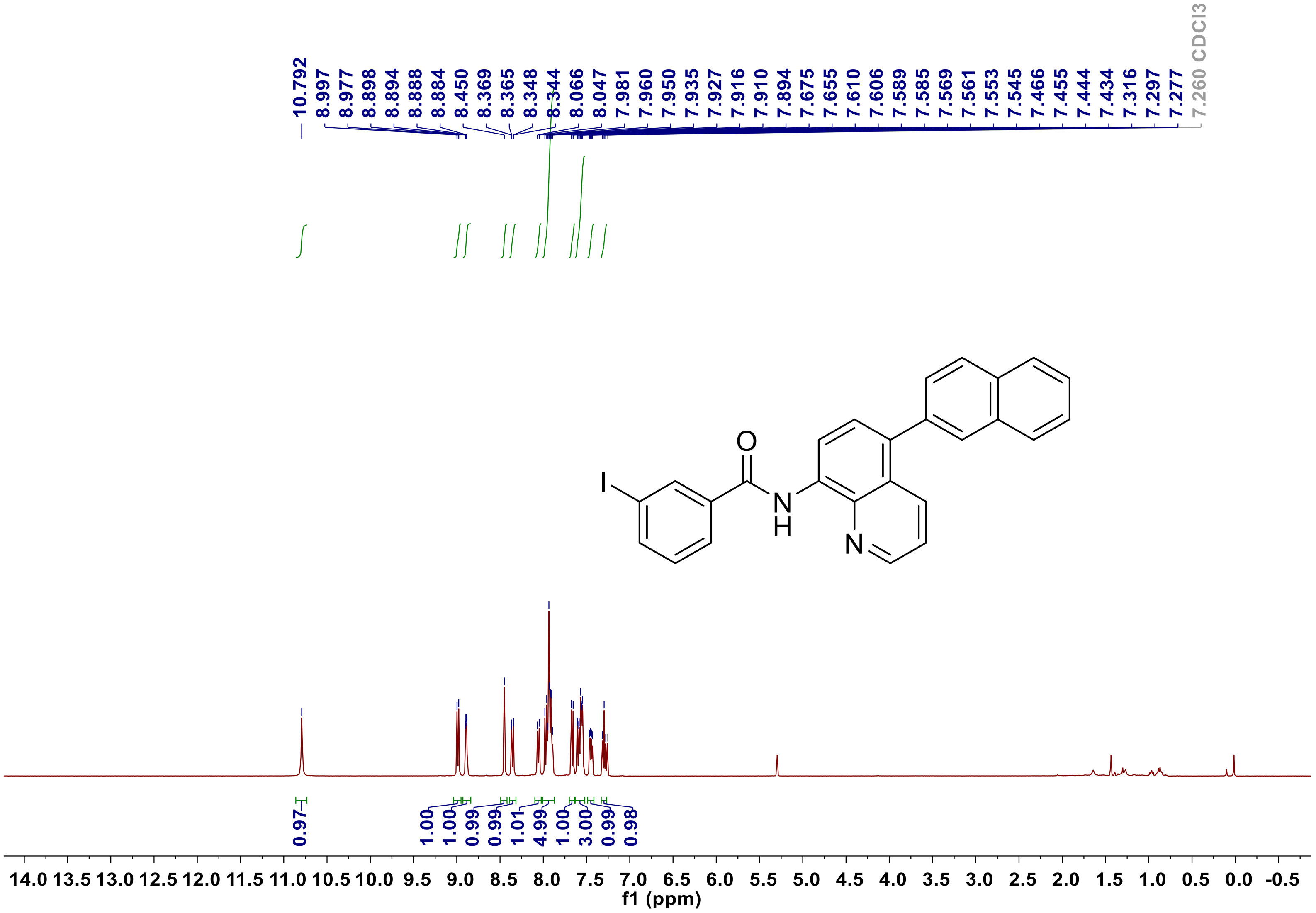

^{13}C NMR of **1g-20**

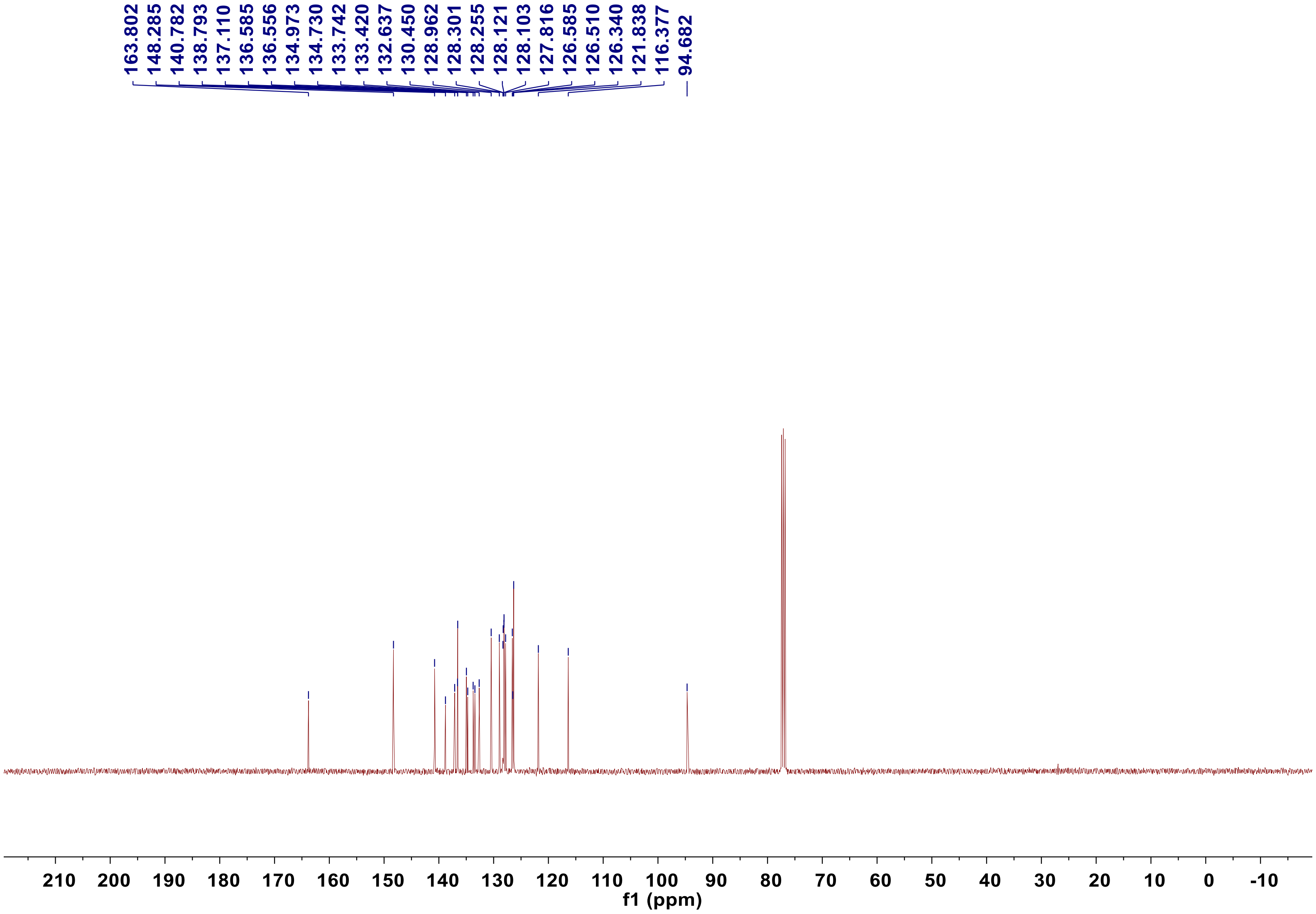

^{1}H NMR of **1g-21**

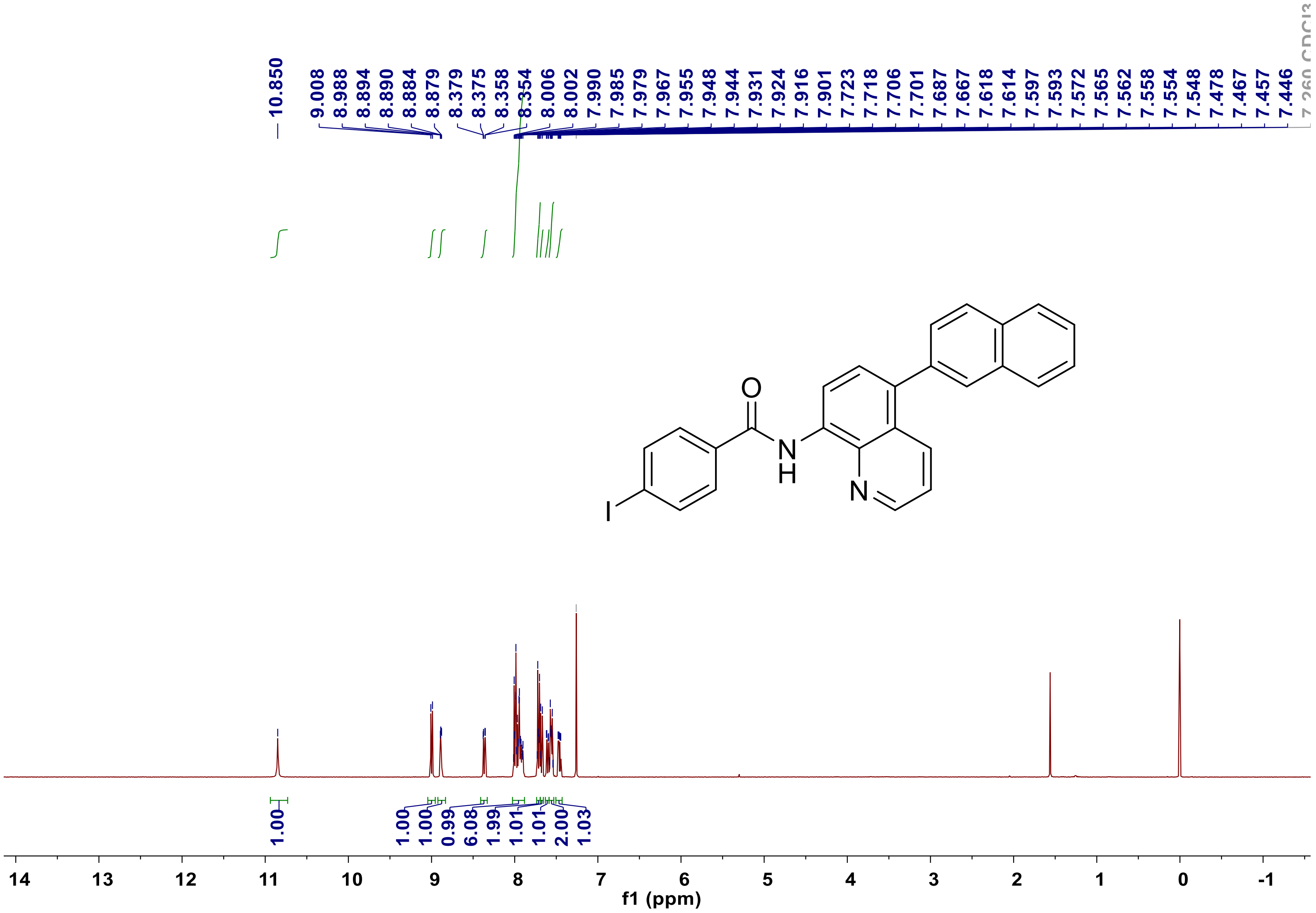

^{13}C NMR of **1g-21**

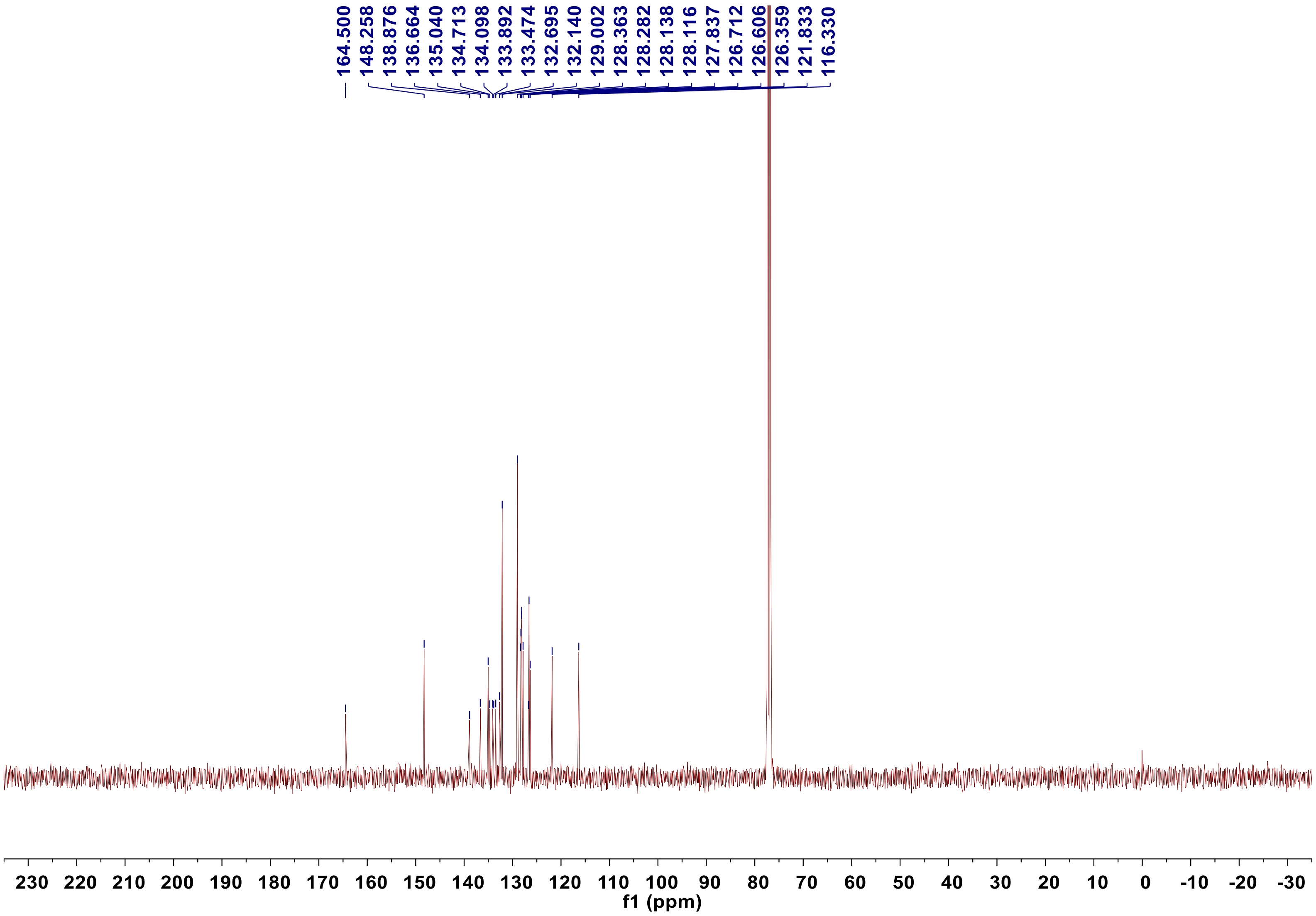

^{1}H NMR of **1g-22**

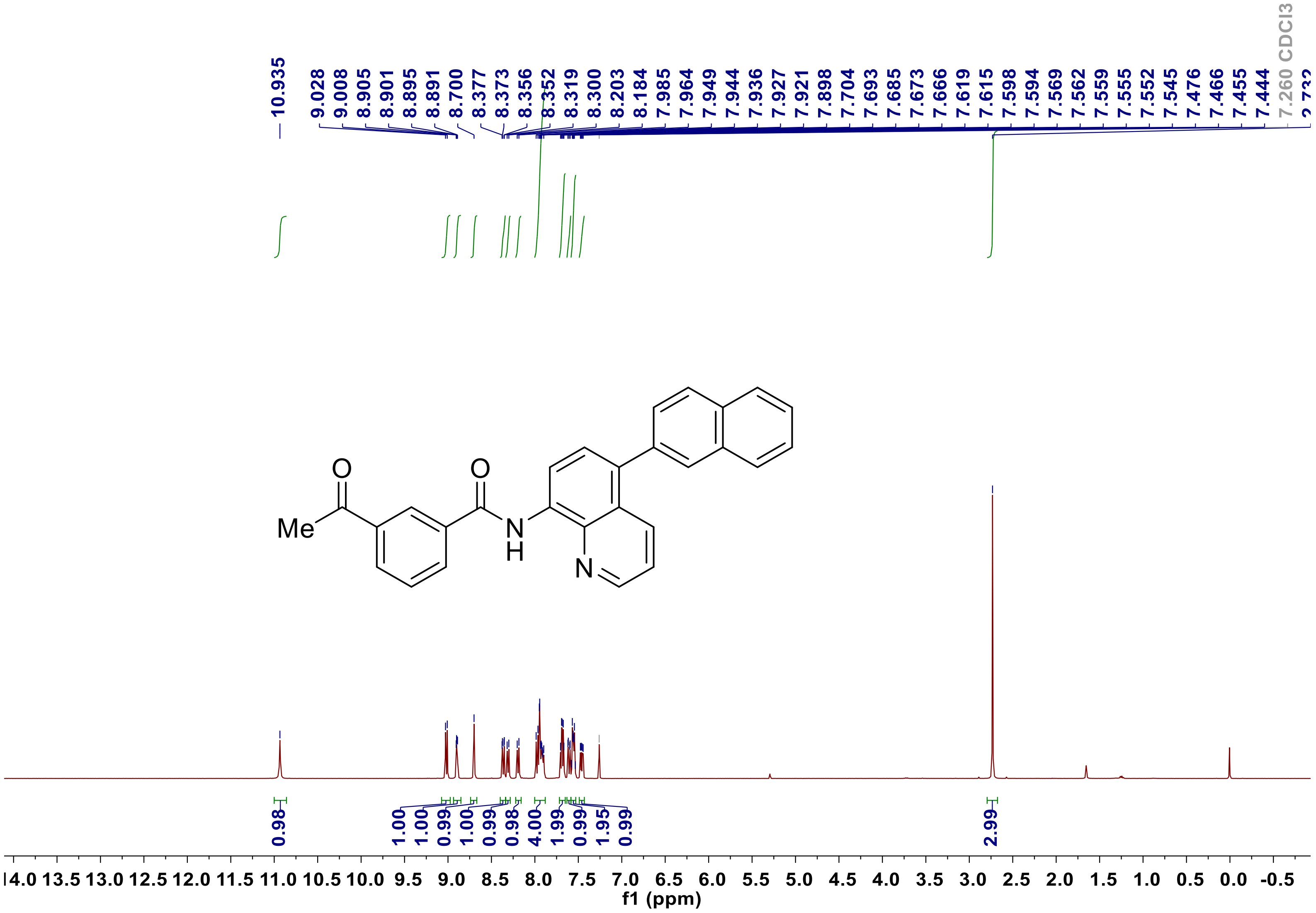

^{13}C NMR of **1g-22**

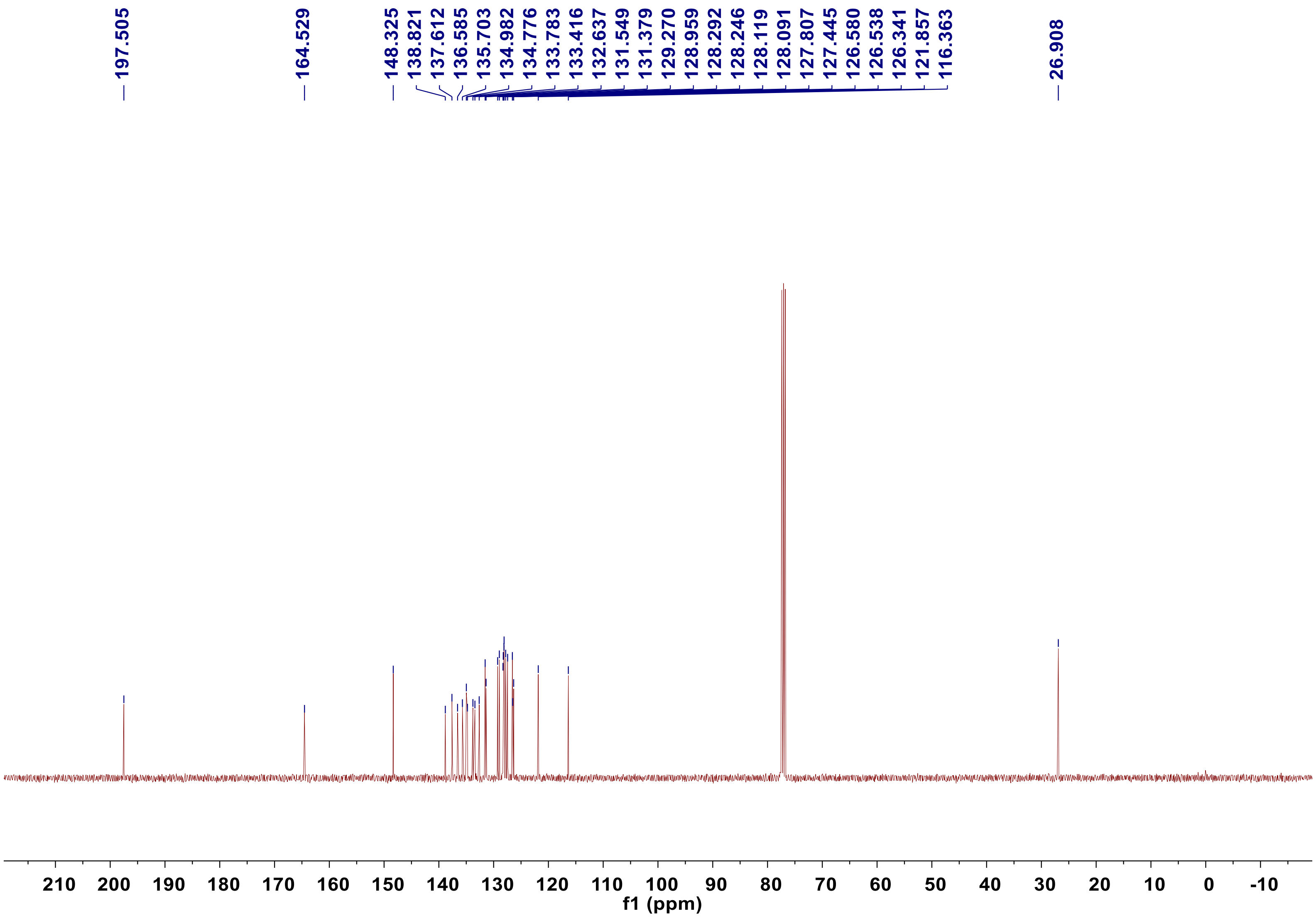

^{1}H NMR of **1g-23**

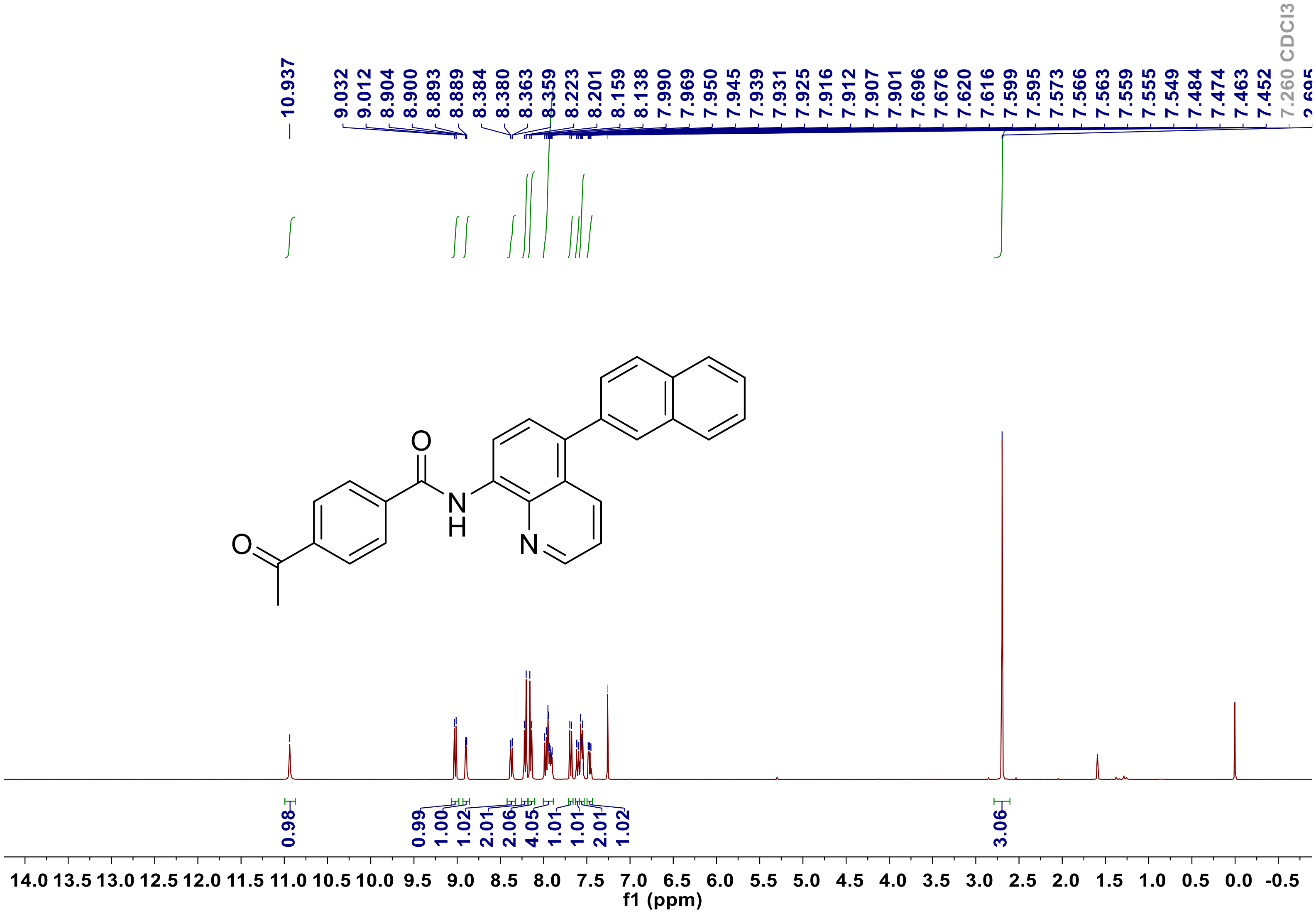

^{13}C NMR of **1g-23**

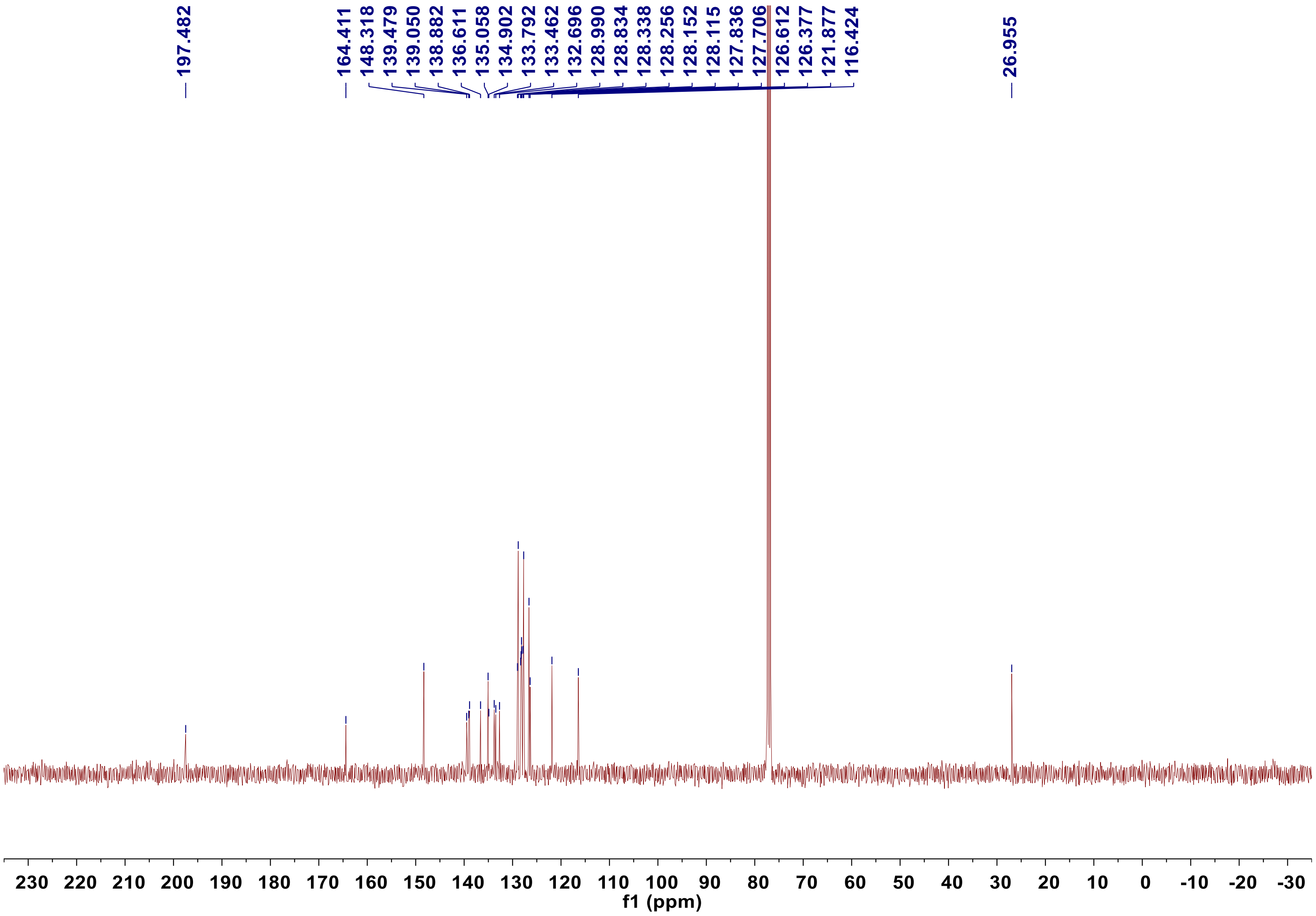

^{1}H NMR of **1g-24**

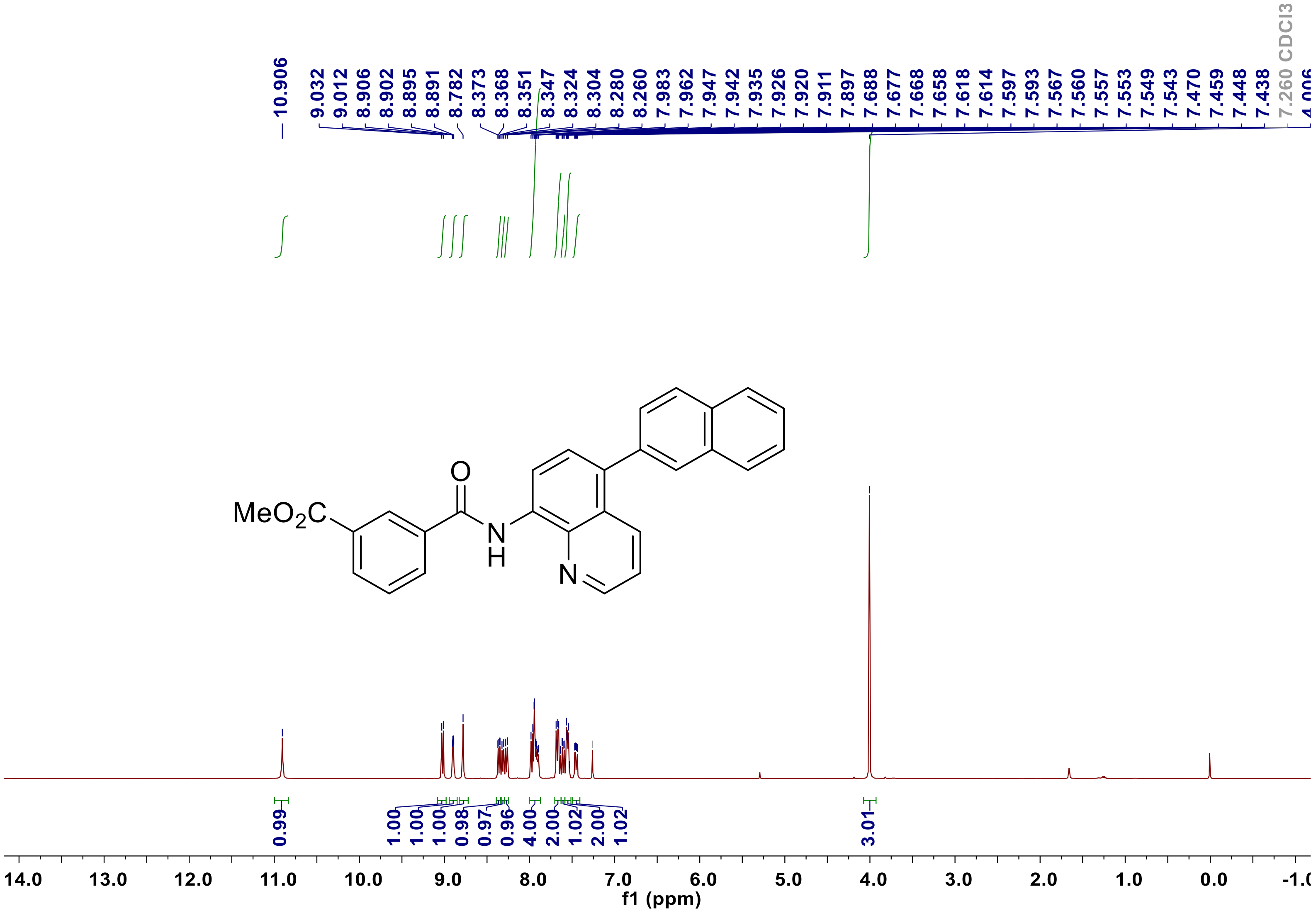

^{13}C NMR of **1g-24**

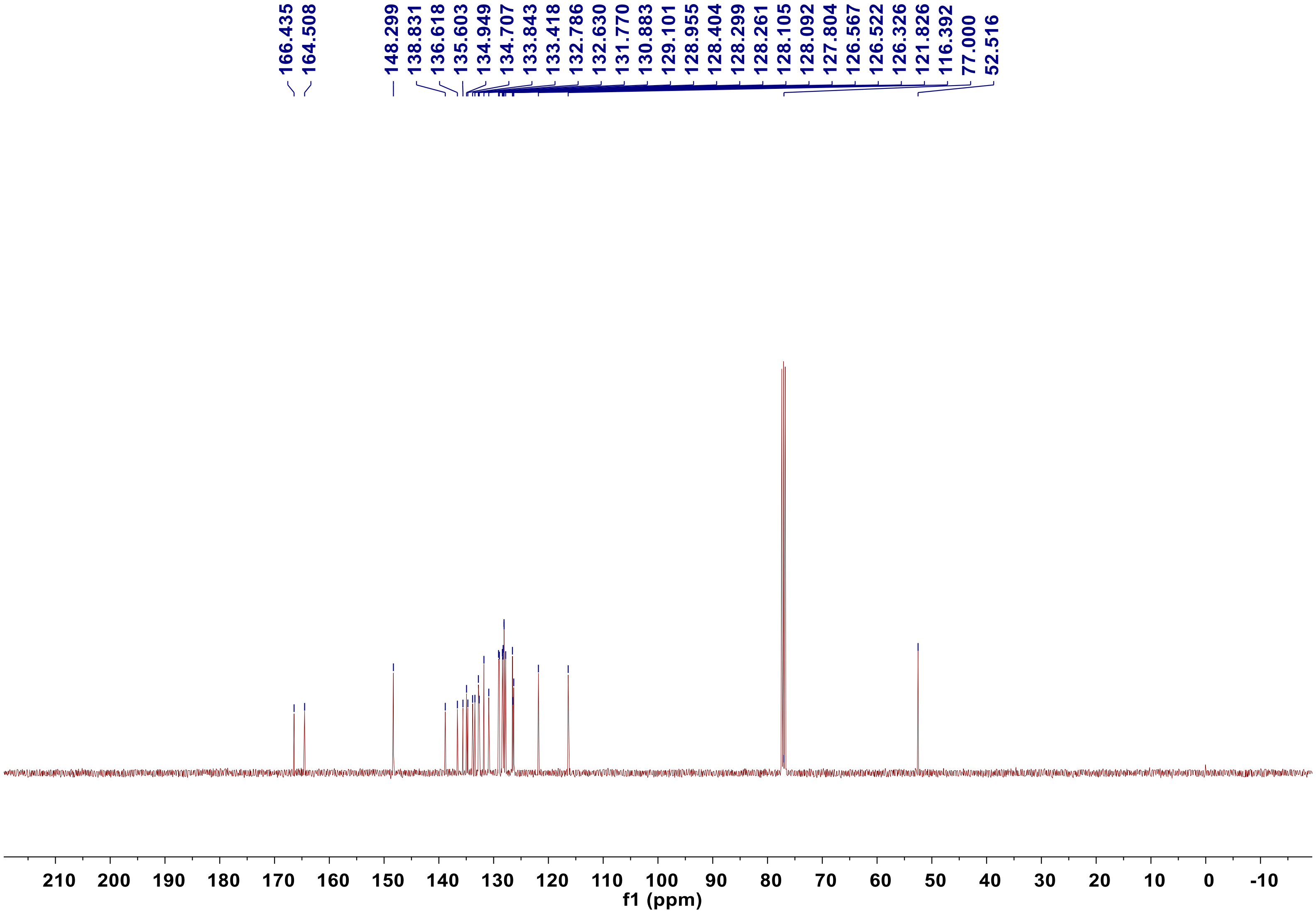

^{1}H NMR of **1g-25**

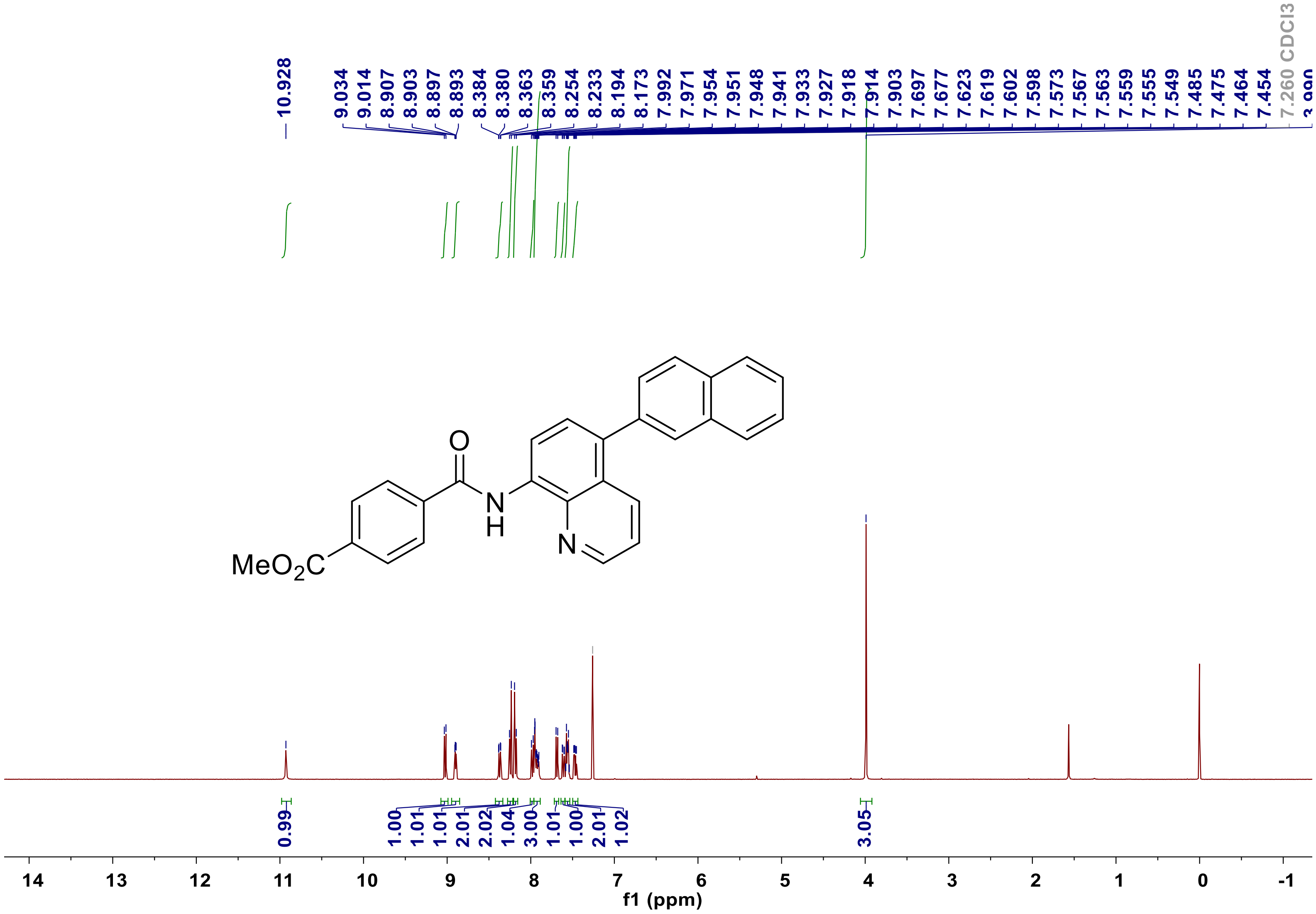

^{13}C NMR of **1g-25**

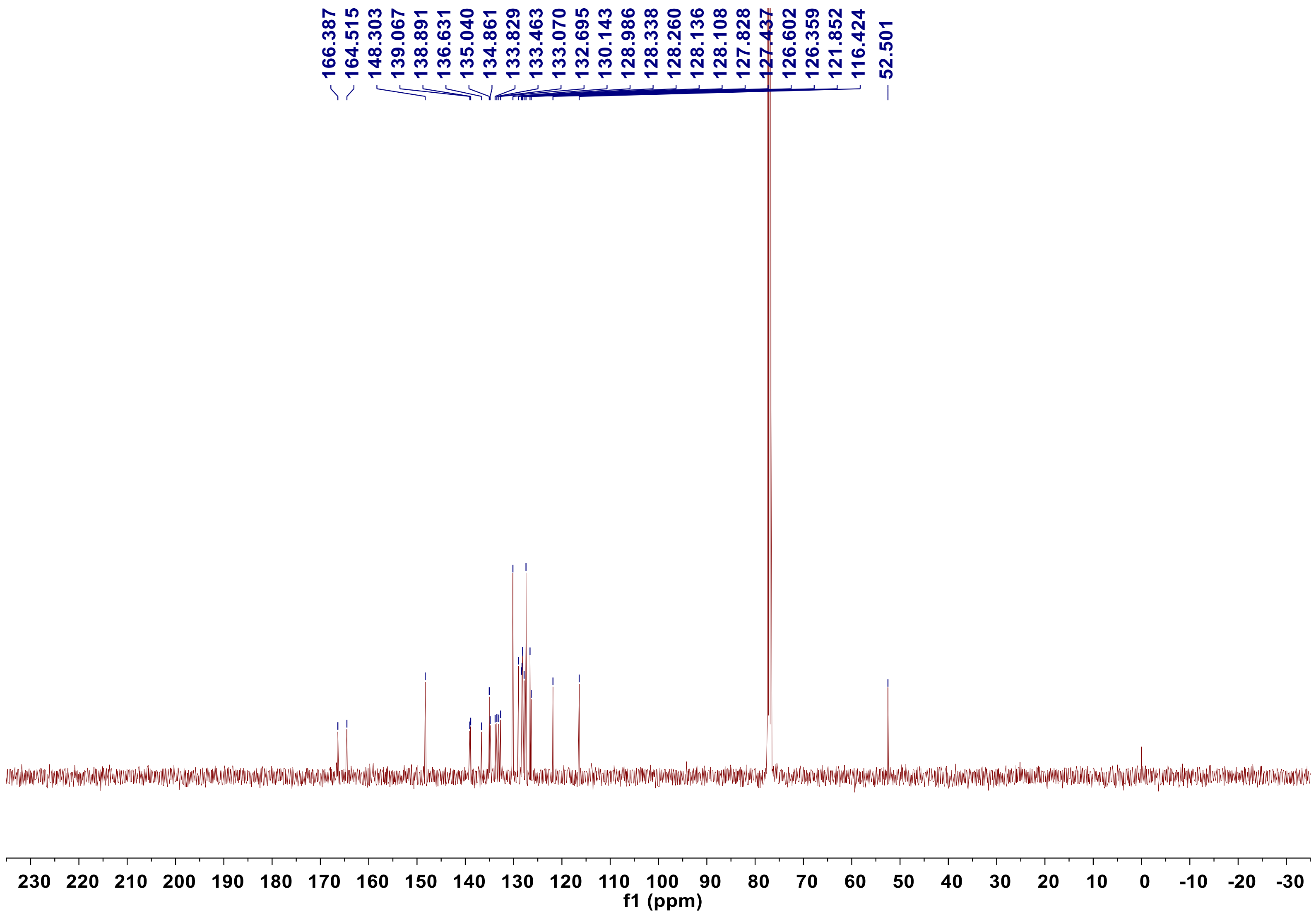

^{1}H NMR of **1g-26**

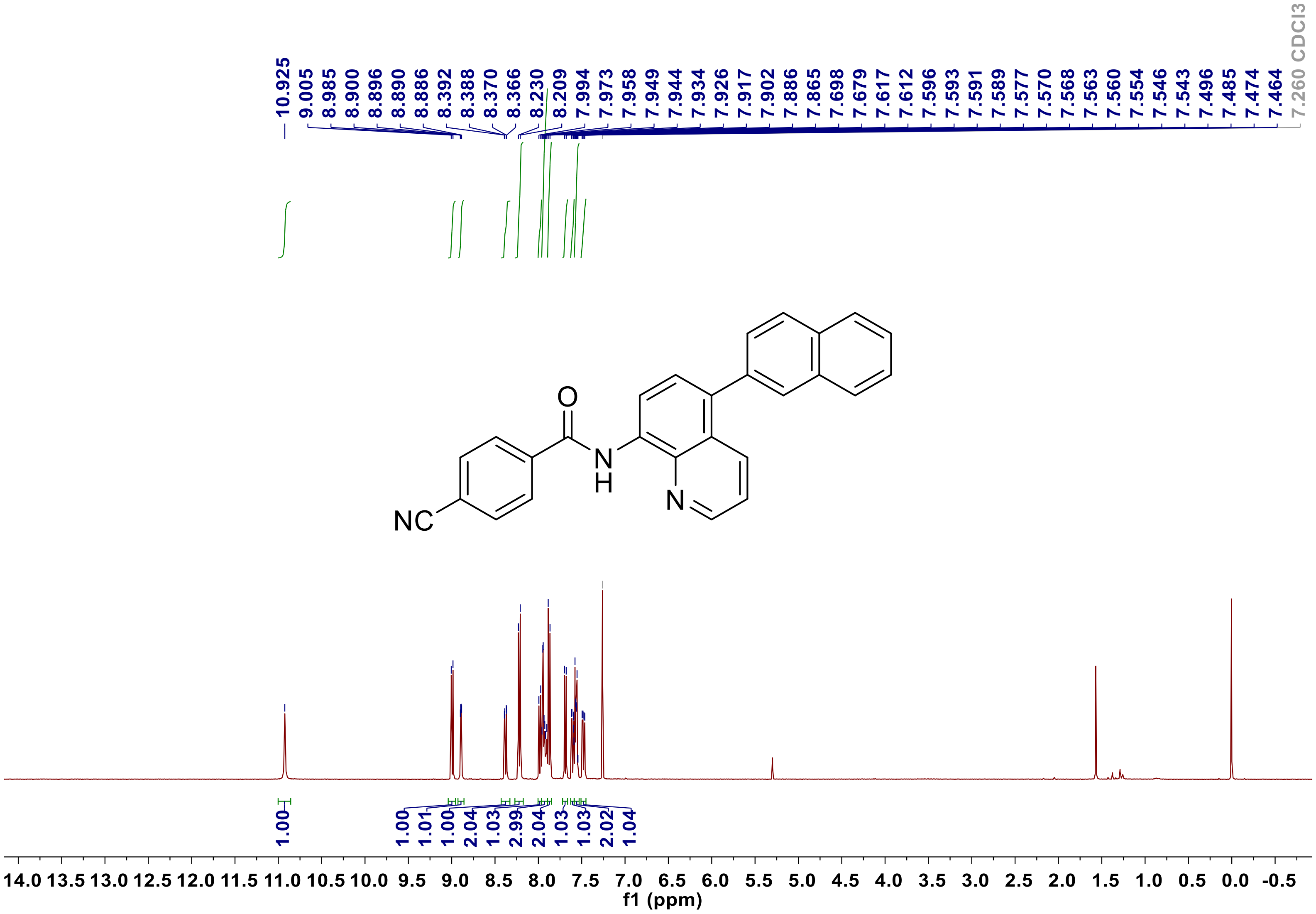

^{13}C NMR of **1g-26**

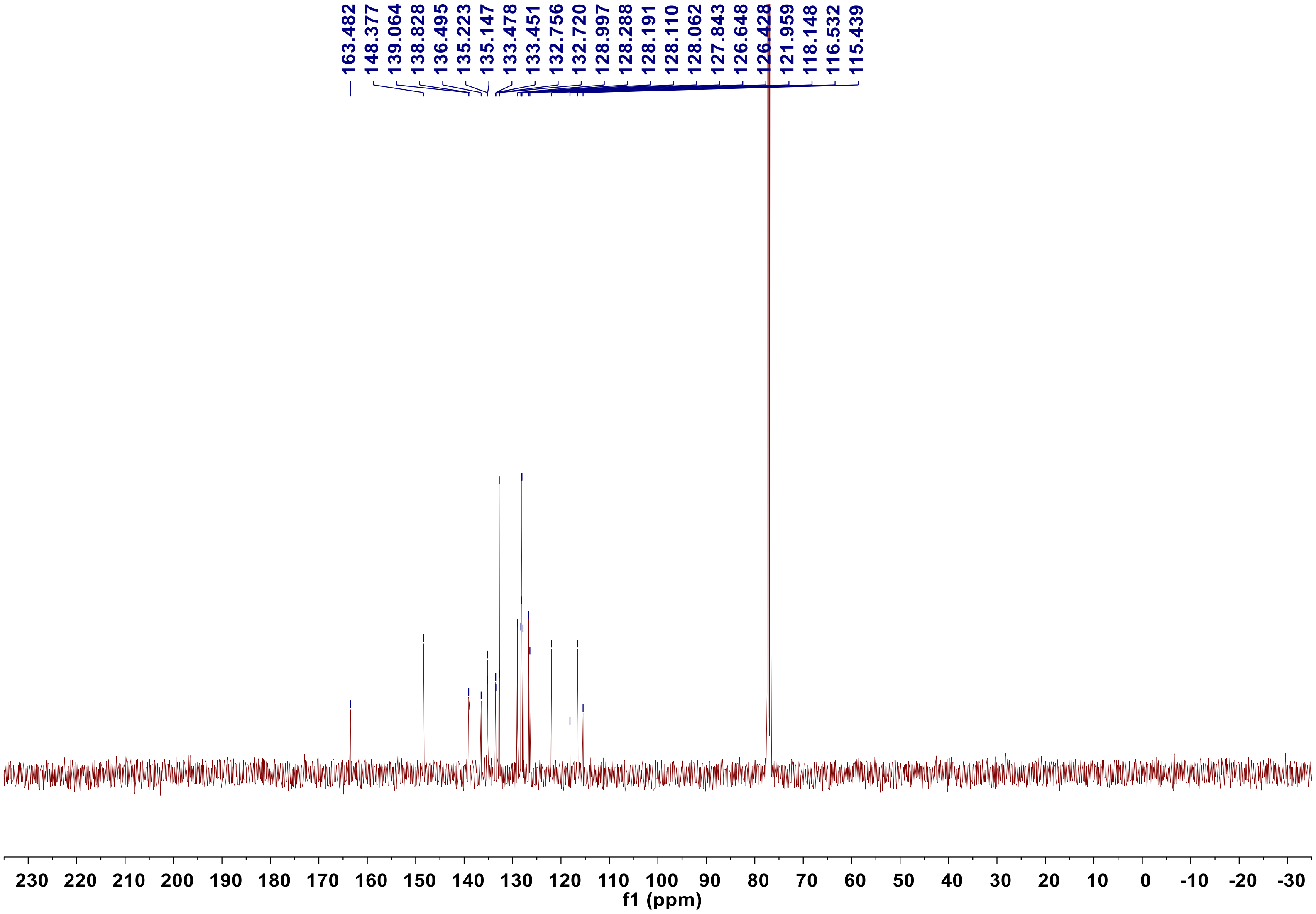

^{1}H NMR of **1g-27**

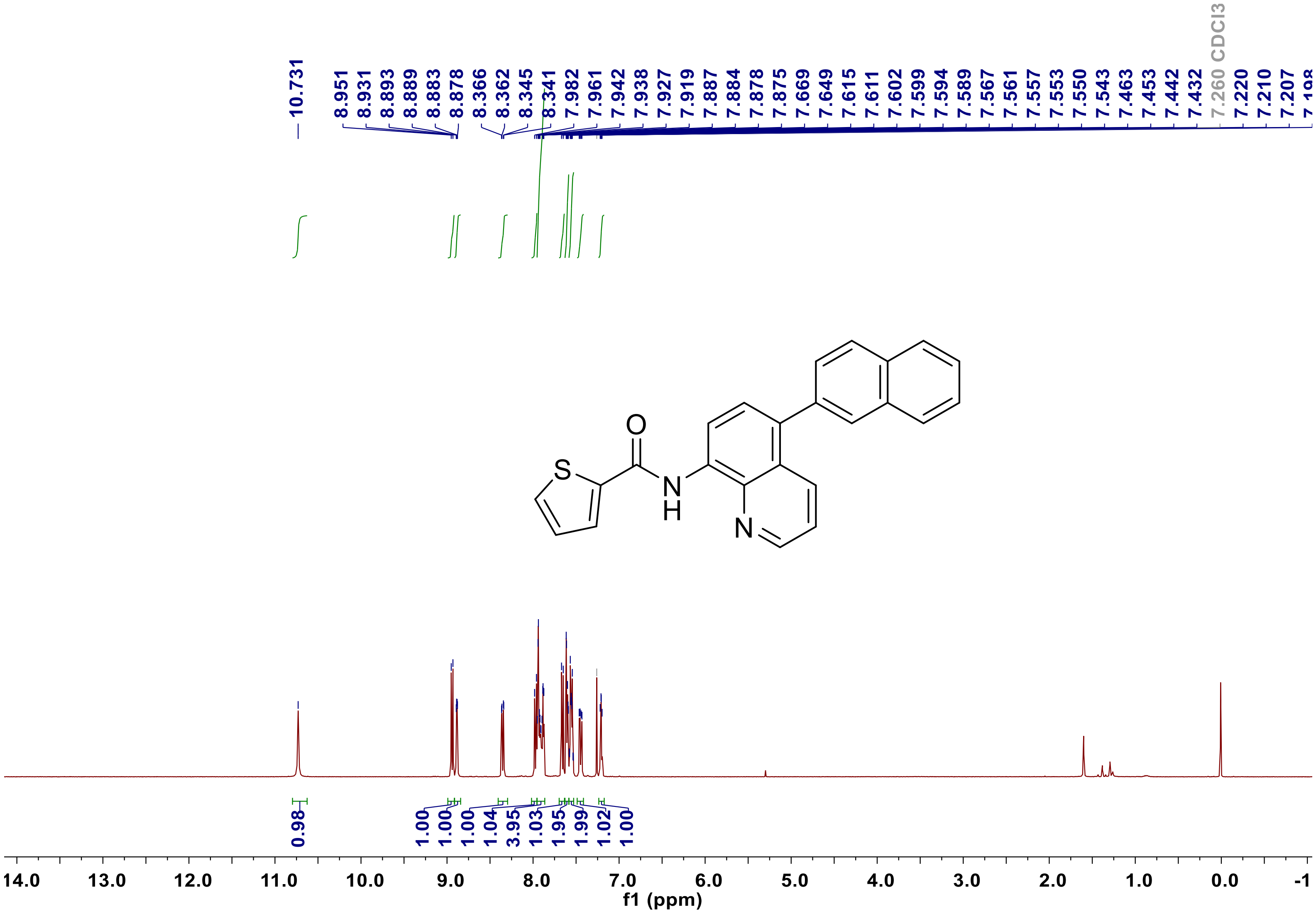

^{13}C NMR of **1g-27**

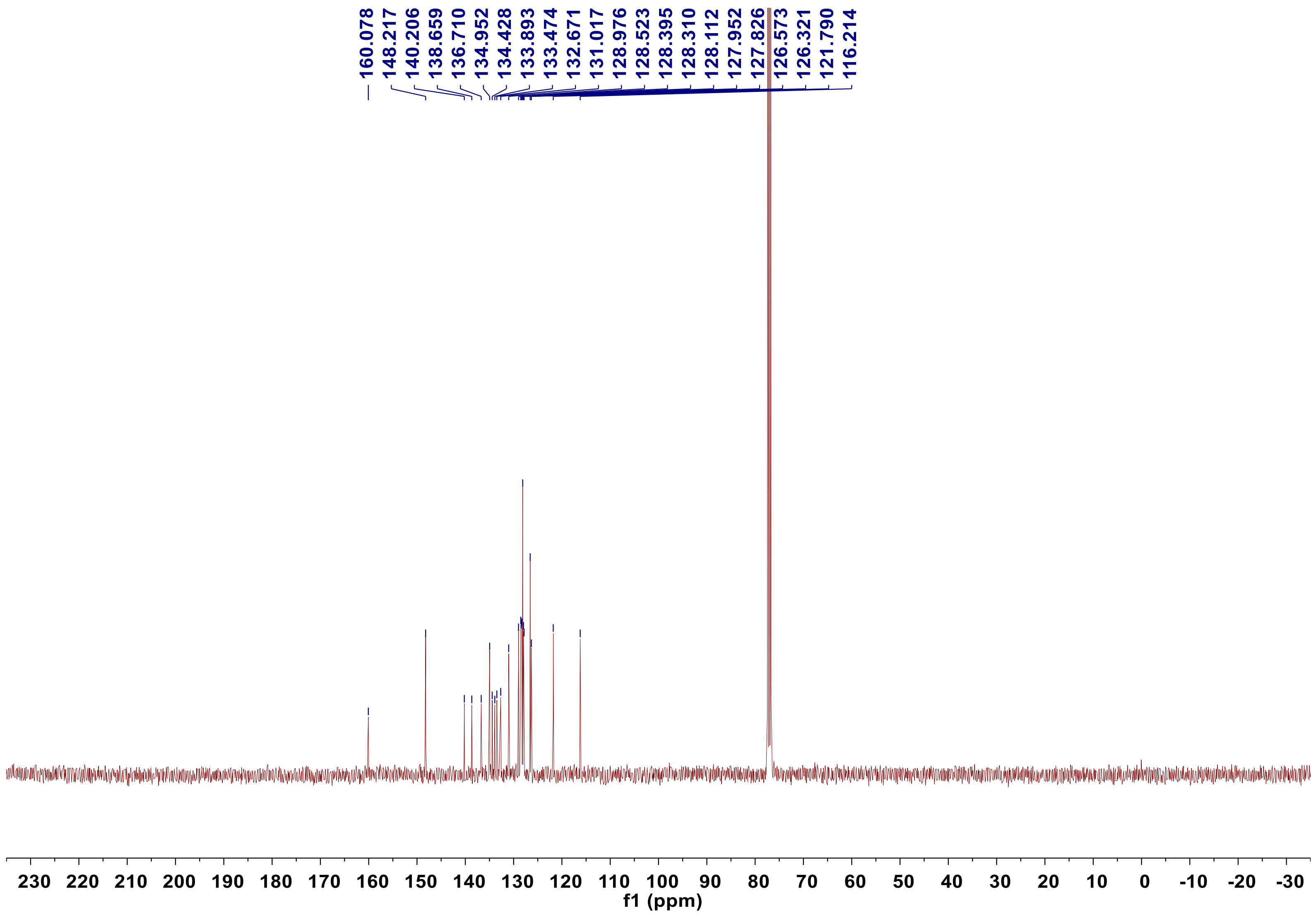

^{1}H NMR of **1g-28**

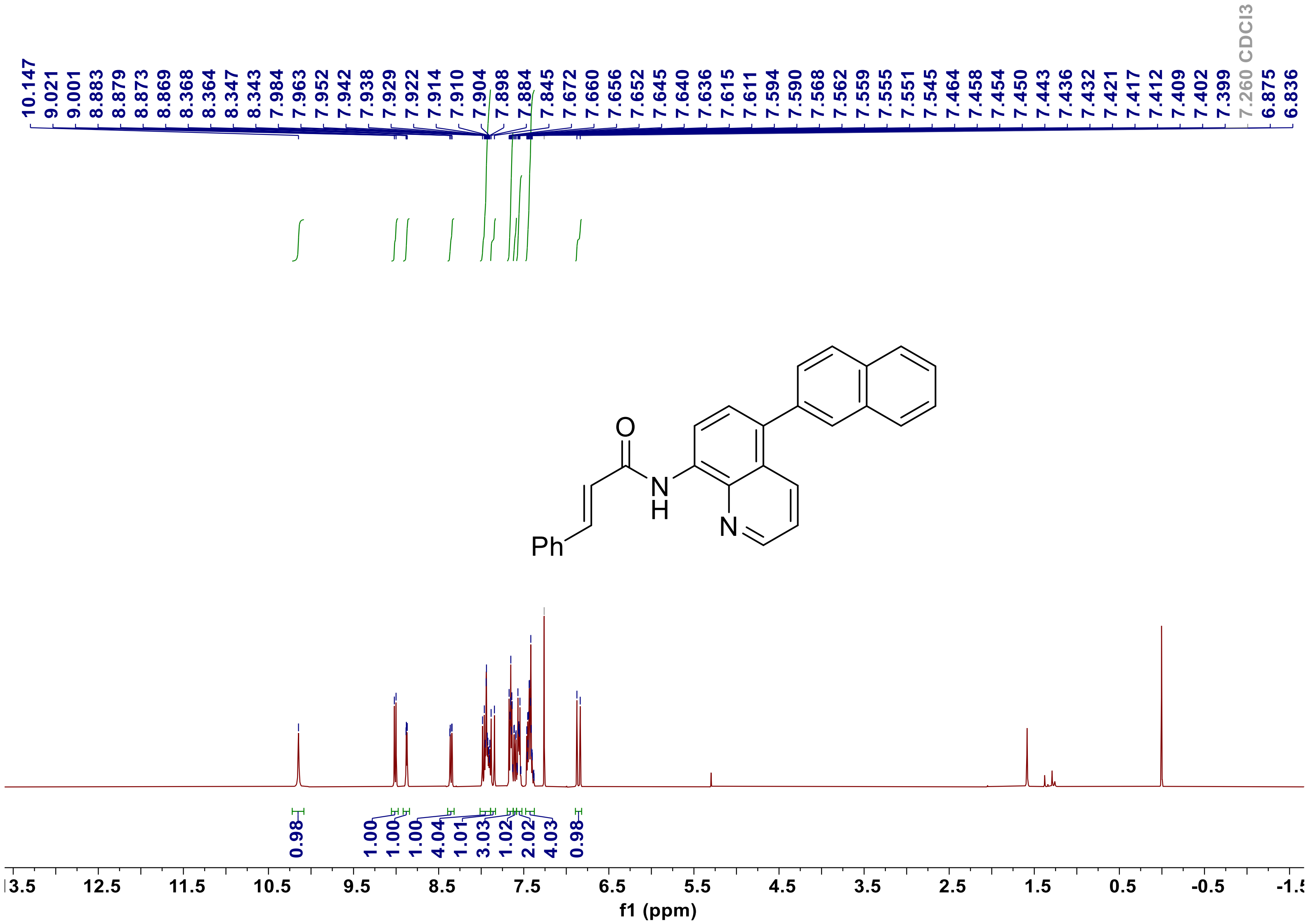

^{13}C NMR of **1g-28**

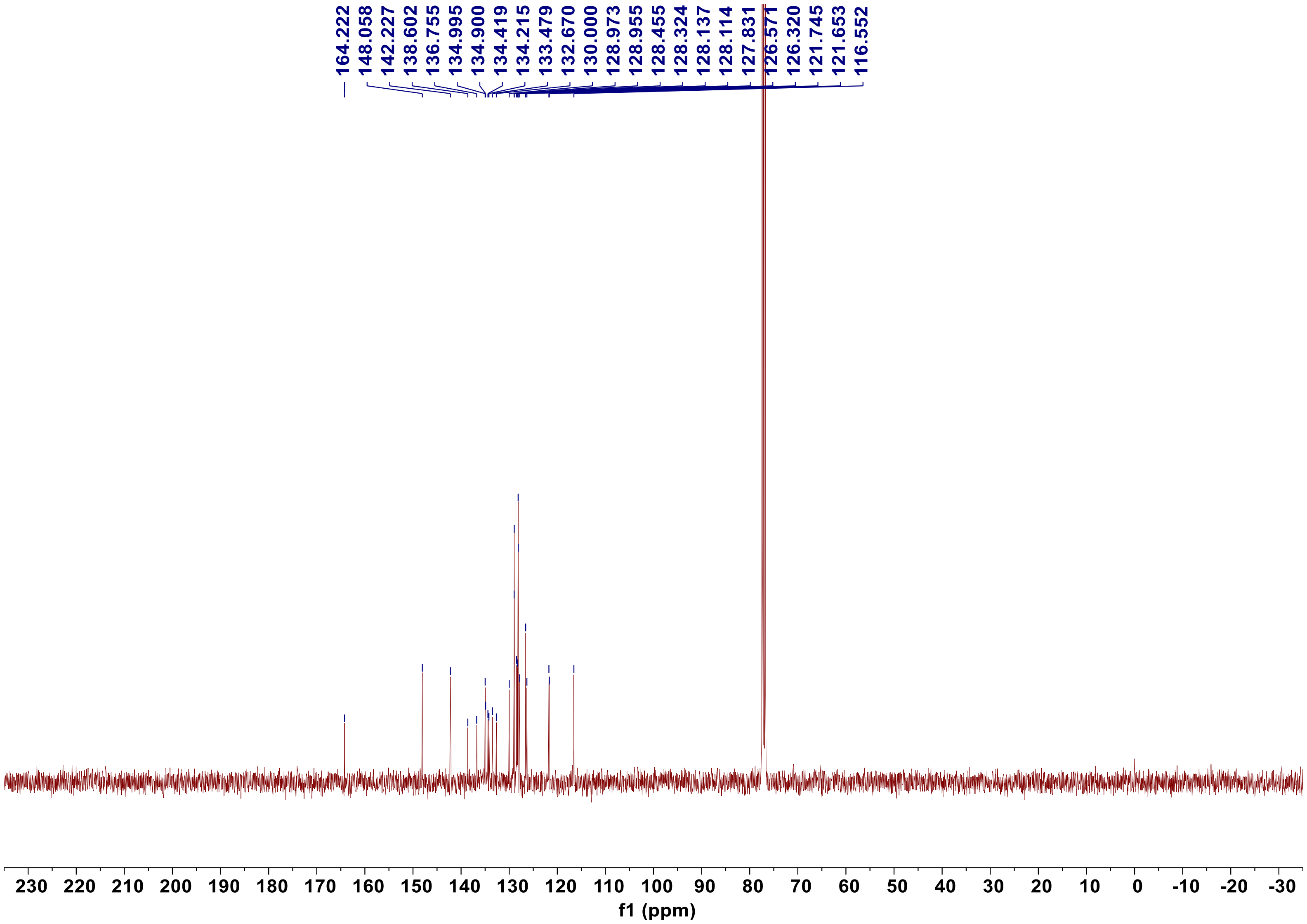

^{1}H NMR of (*S*)-**L6**

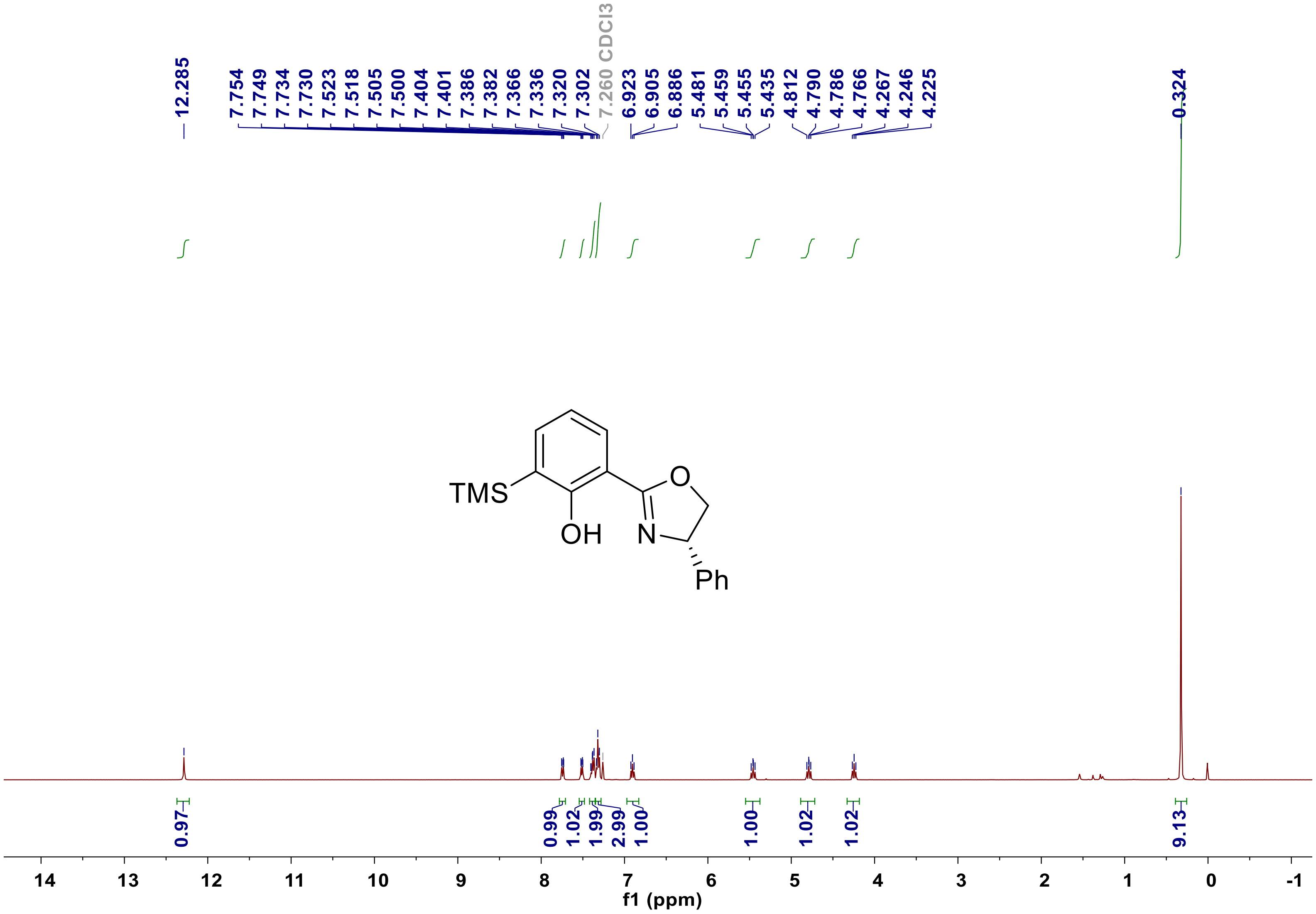

12.285
7.754
7.749
7.734
7.730
7.523
7.518
7.505
7.500
7.404
7.401
7.386
7.382
7.366
7.336
7.320
7.302
7.260 CDCl3
6.923
6.905
6.886
5.481
5.459
5.455
5.435
4.812
4.790
4.786
4.766
4.267
4.246
4.225
0.324
TMS
OH
O
N
Ph
0.97
0.99
1.02
1.99
2.99
1.00
1.00
1.02
1.02
9.13
14
13
12
11
10
9
8
7
6
5
4
3
2
1
0
-1
f1 (ppm)

^{13}C NMR of (*S*)-**L6**

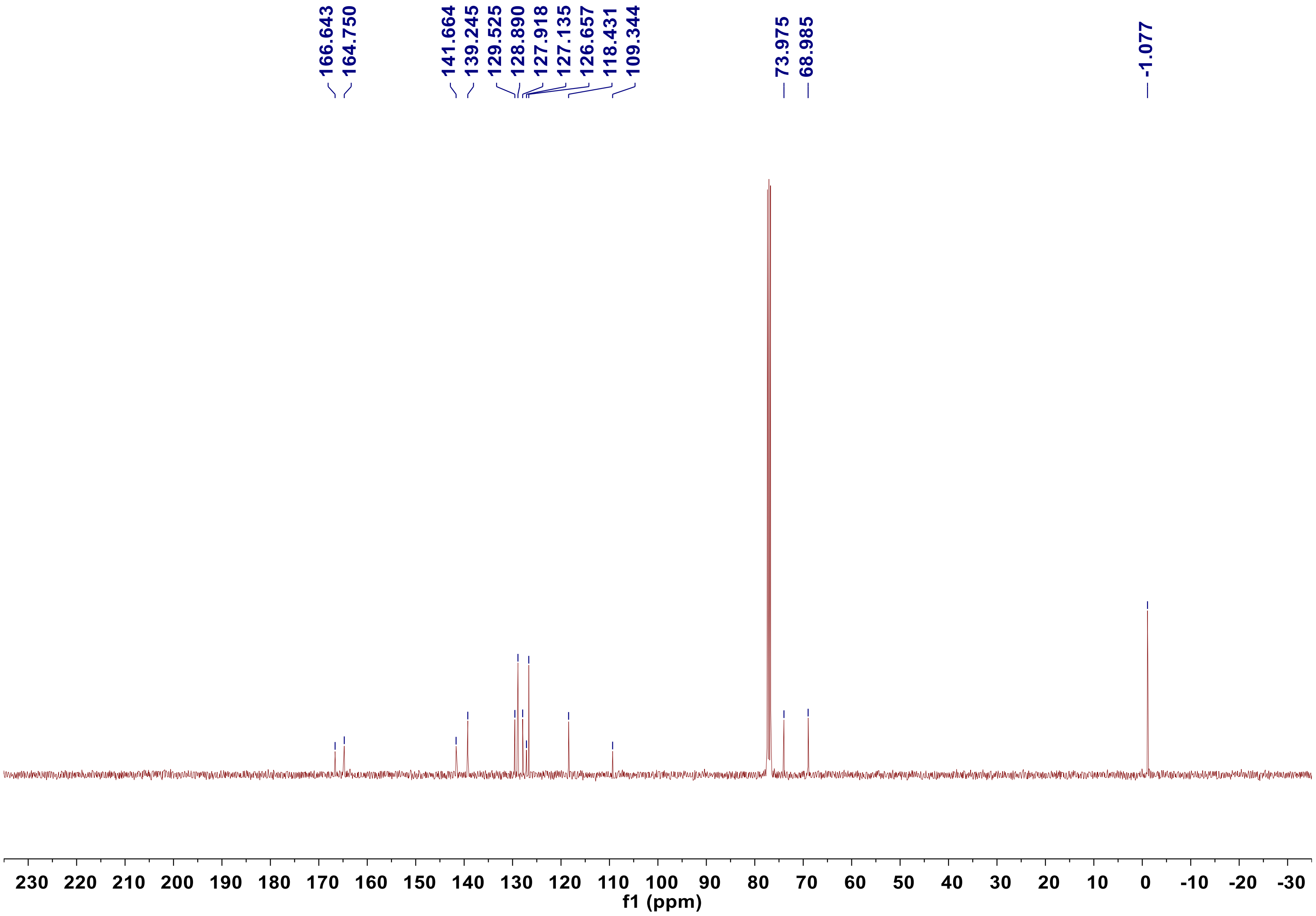

^{1}H NMR of (*S*)-**L7**

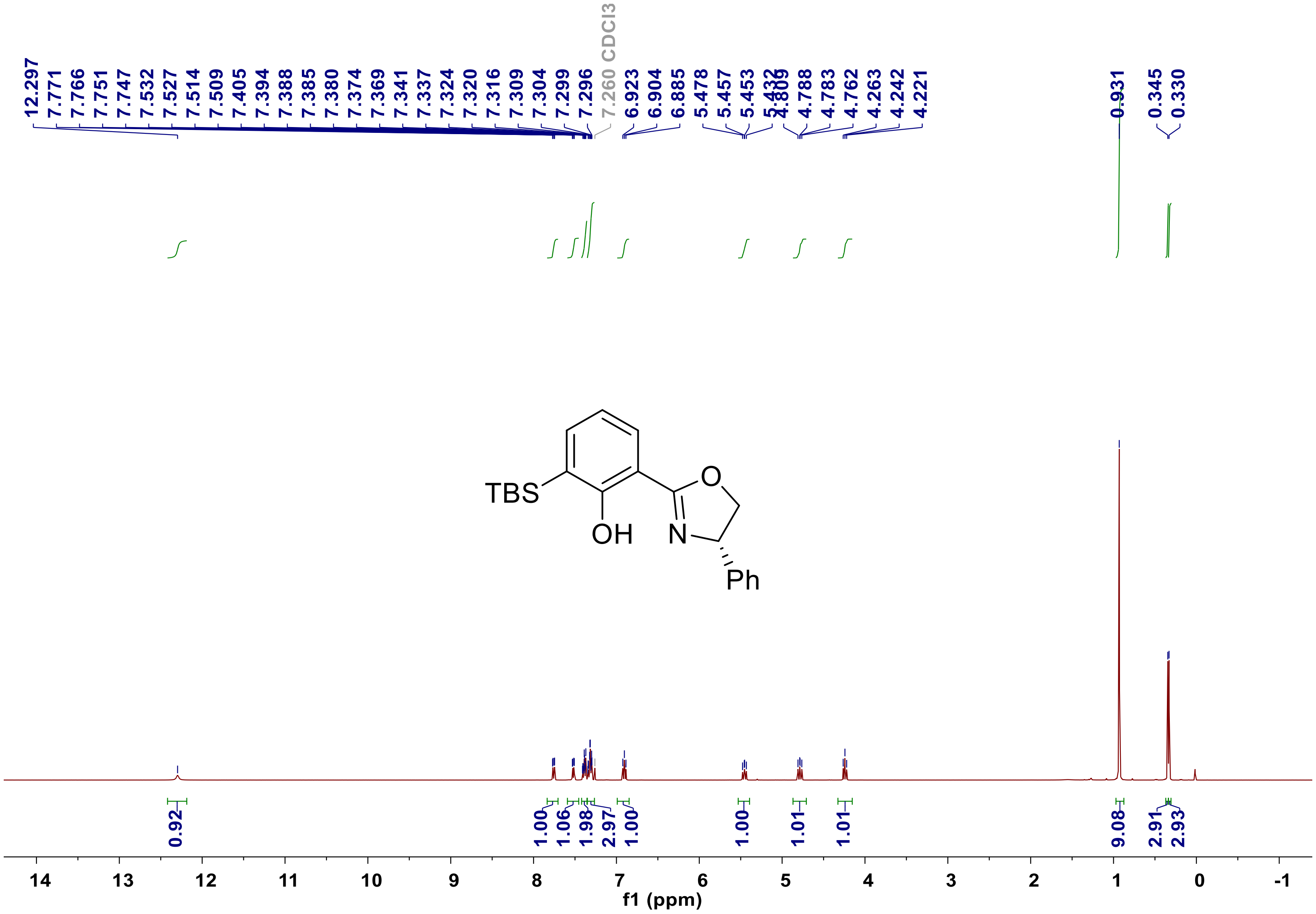
12.297
7.771
7.766
7.751
7.747
7.532
7.527
7.514
7.509
7.405
7.394
7.388
7.385
7.380
7.374
7.369
7.341
7.337
7.324
7.320
7.316
7.309
7.304
7.299
7.296
7.260 CDCl3
6.923
6.904
6.885
5.478
5.457
5.453
5.432
4.809
4.788
4.783
4.762
4.263
4.242
4.221
0.931
0.345
0.330
TBS
OH
O
N
Ph
0.92
1.00
1.06
1.98
2.97
1.00
1.00
1.01
1.01
9.08
2.91
2.93
f1 (ppm)

^{13}C NMR of (*S*)-**L7**

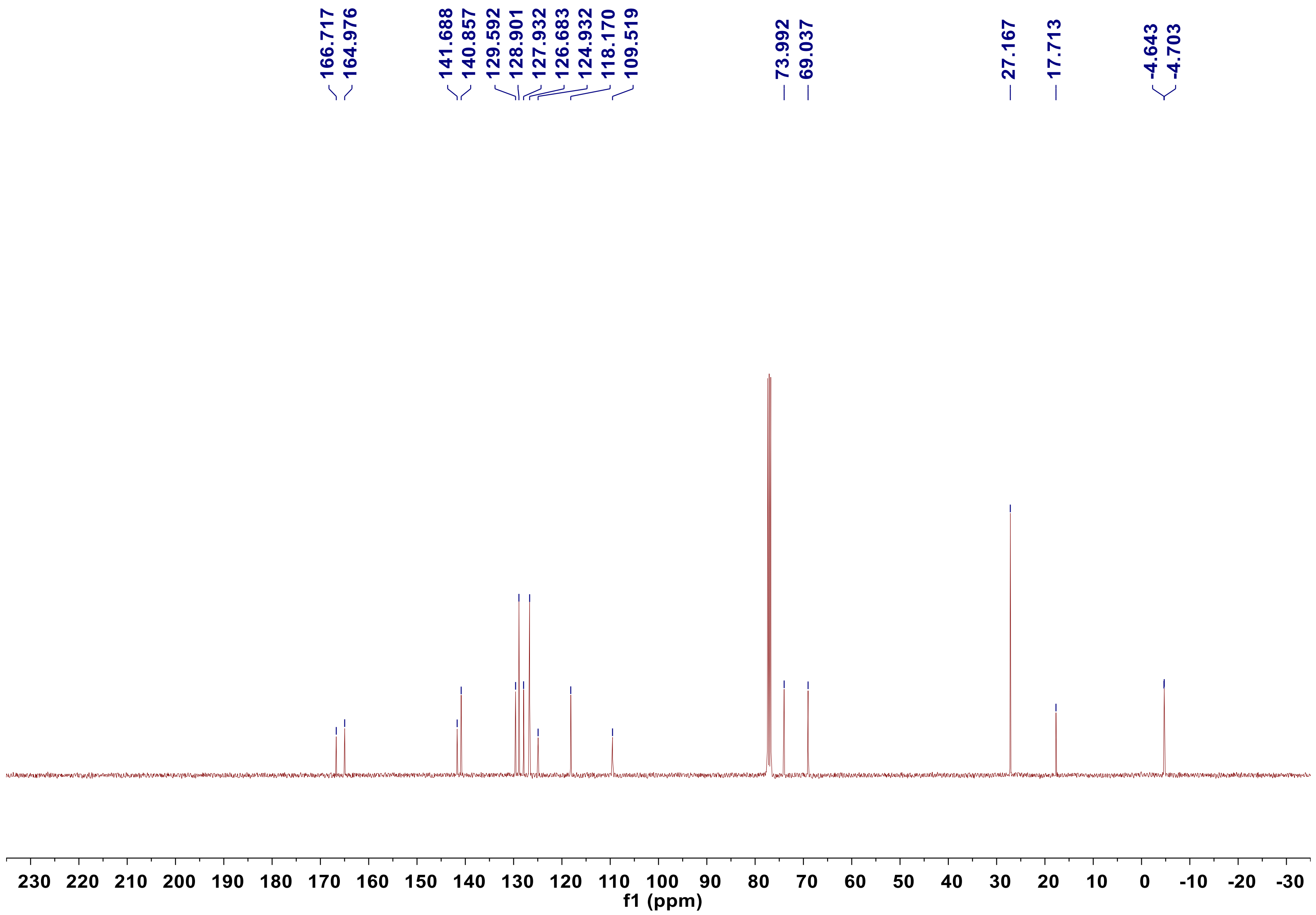

^{1}H NMR of (*S*)-**L8**

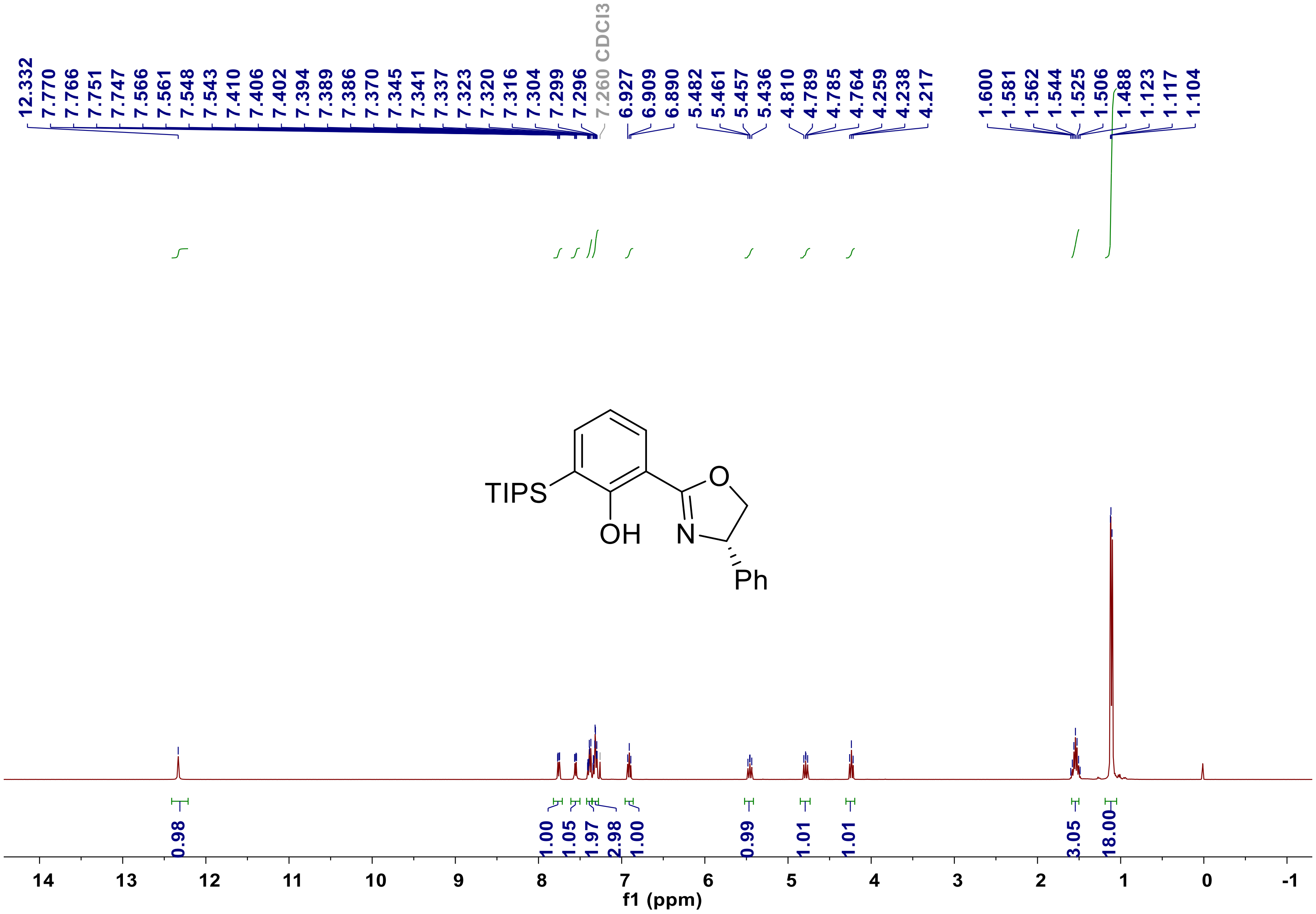

12.332
7.770
7.766
7.751
7.747
7.566
7.561
7.548
7.543
7.410
7.406
7.402
7.394
7.389
7.386
7.370
7.345
7.341
7.337
7.323
7.320
7.316
7.304
7.299
7.296
7.260 CDCl3
6.927
6.909
6.890
5.482
5.461
5.457
5.436
4.810
4.789
4.785
4.764
4.259
4.238
4.217
1.600
1.581
1.562
1.544
1.525
1.506
1.488
1.123
1.117
1.104
TIPS
OH
O
N
Ph
0.98
1.00
1.05
1.97
2.98
1.00
0.99
1.01
1.01
3.05
18.00
14
13
12
11
10
9
8
7
6
5
4
3
2
1
0
-1
f1 (ppm)

^{13}C NMR of (*S*)-**L8**

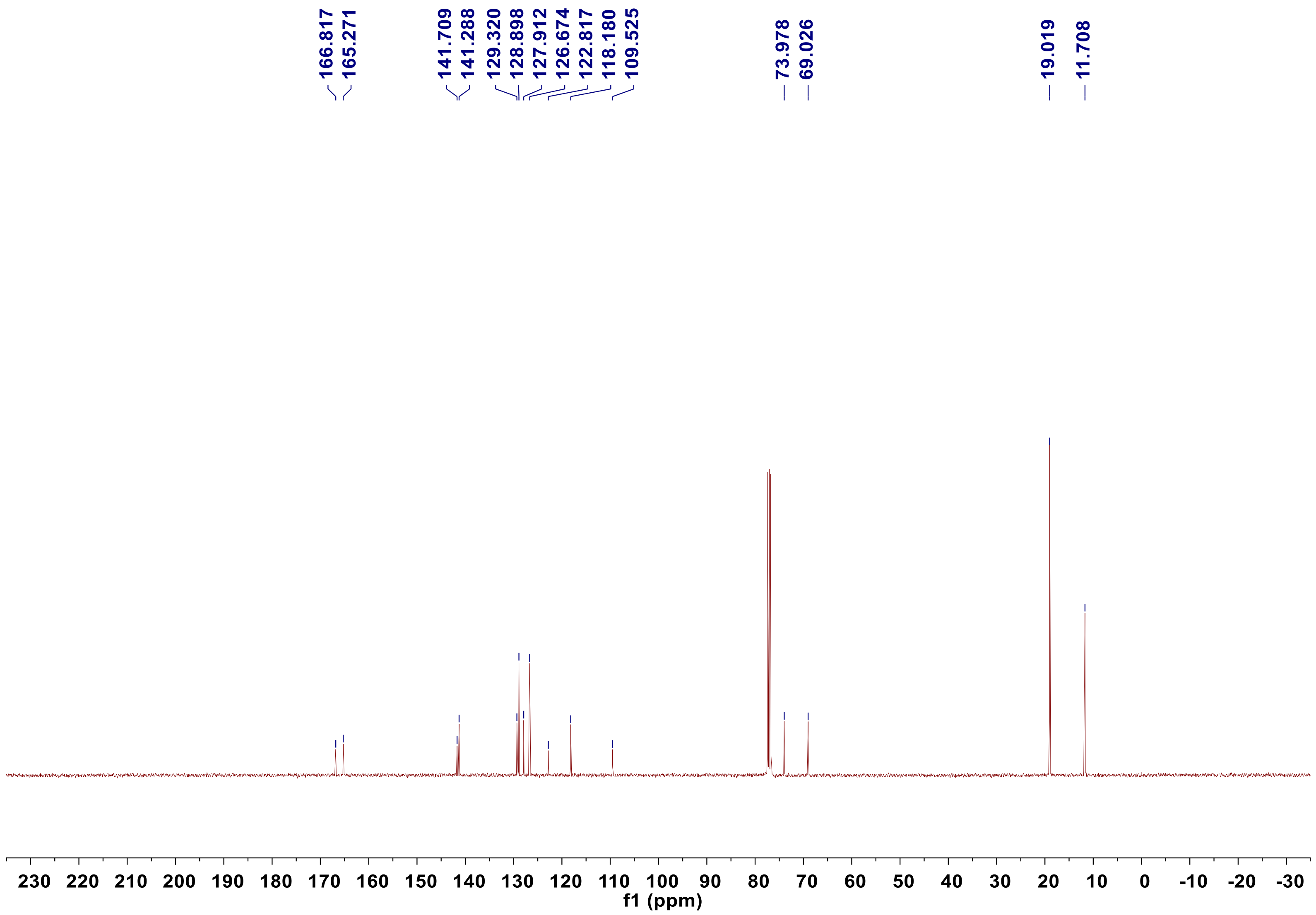

^{1}H NMR of **3a-1**

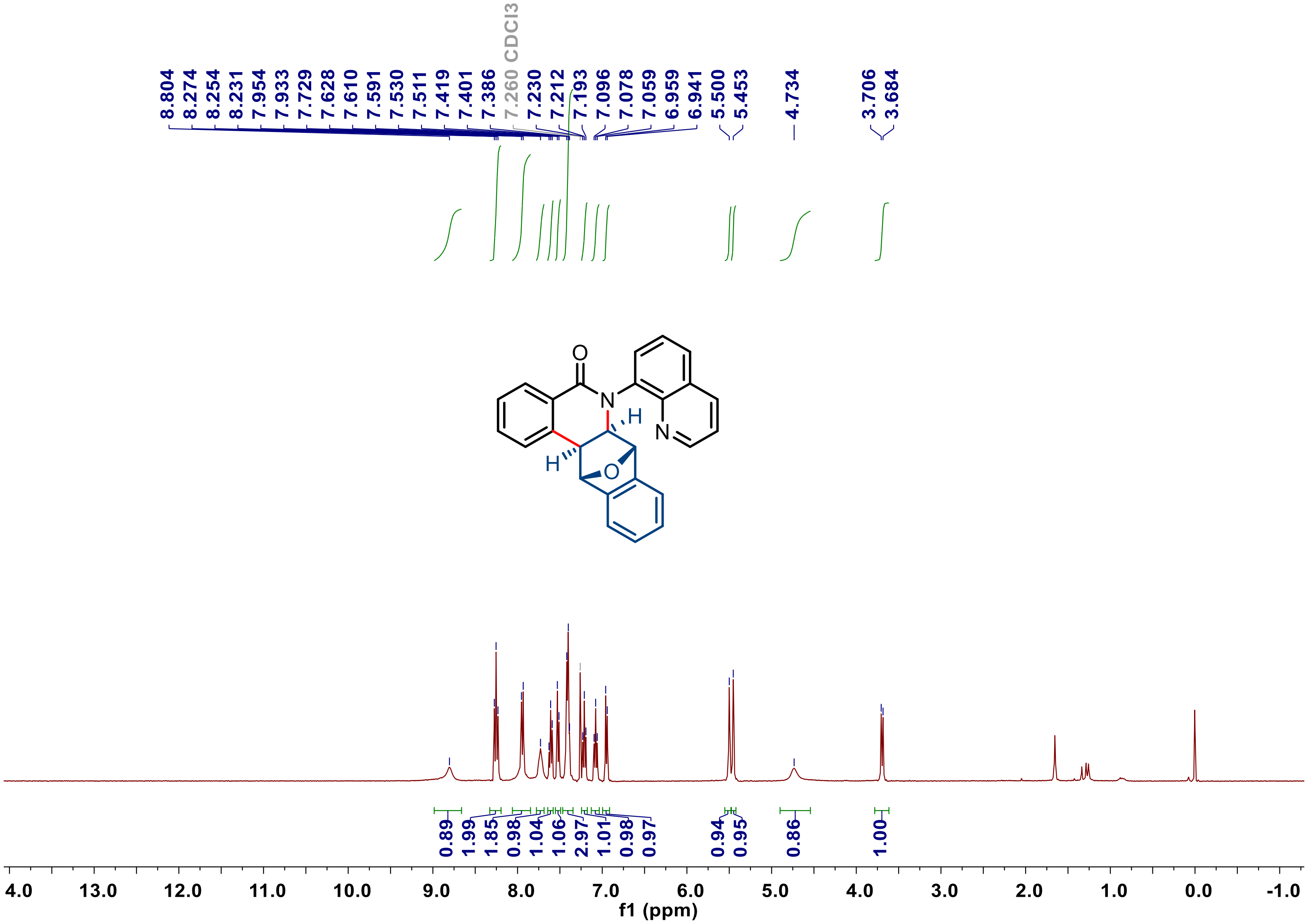

8.804
8.274
8.254
8.231
7.954
7.933
7.729
7.628
7.610
7.591
7.530
7.511
7.419
7.401
7.386
7.260 CDCl3
7.230
7.212
7.193
7.096
7.078
7.059
6.959
6.941
5.500
5.453
4.734
3.706
3.684
O
N
H
N
H
O
0.89
1.99
1.85
0.98
1.04
1.06
2.97
1.01
0.98
0.97
0.94
0.95
0.86
1.00
4.0
13.0
12.0
11.0
10.0
9.0
8.0
7.0
6.0
5.0
4.0
3.0
2.0
1.0
0.0
-1.0
f1 (ppm)

^{13}C NMR of **3a-1**

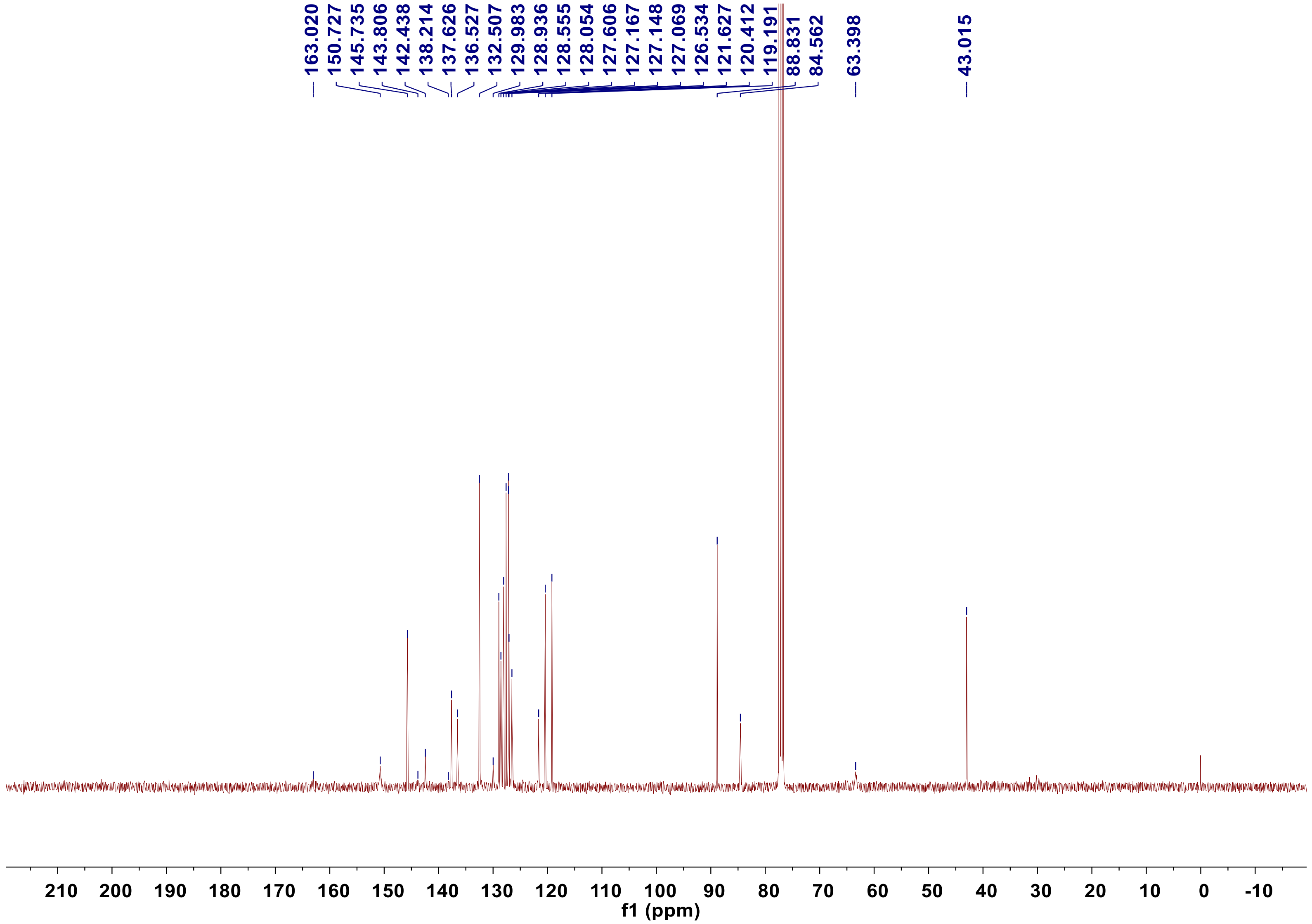

^{1}H NMR of **3b-1**

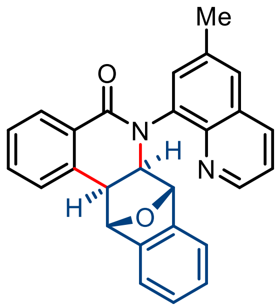

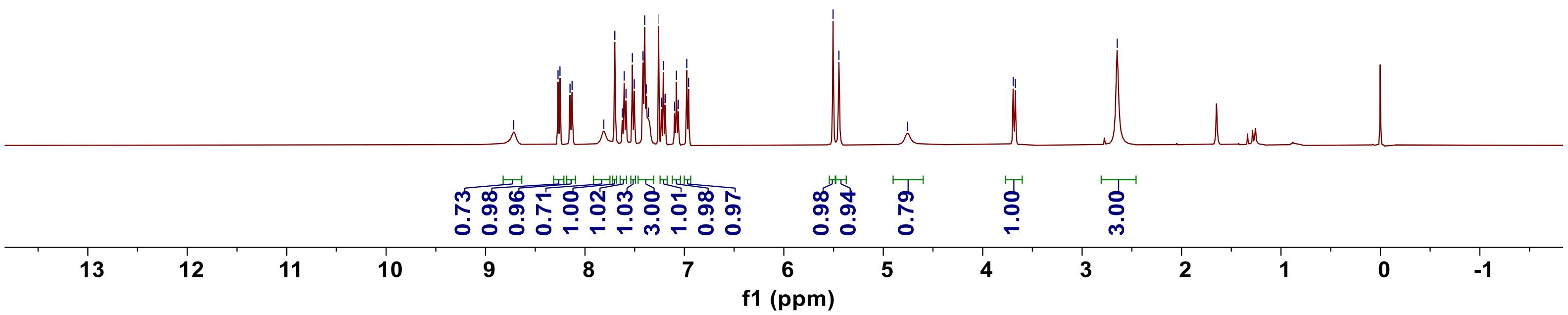
8.717
8.271
8.251
8.150
8.130
7.811
7.700
7.625
7.606
7.587
7.524
7.504
7.417
7.399
7.384
7.361
7.260 CDCl3
7.231
7.212
7.194
7.099
7.080
7.062
6.976
6.958
5.505
5.446
4.755
3.693
3.672
2.647
0.73
0.98
0.96
0.71
1.00
1.02
1.03
3.00
1.01
0.98
0.97
0.98
0.94
0.79
1.00
3.00
13 12 11 10 9 8 7 6 5 4 3 2 1 0 -1
f1 (ppm)

^{13}C NMR of **3b-1**

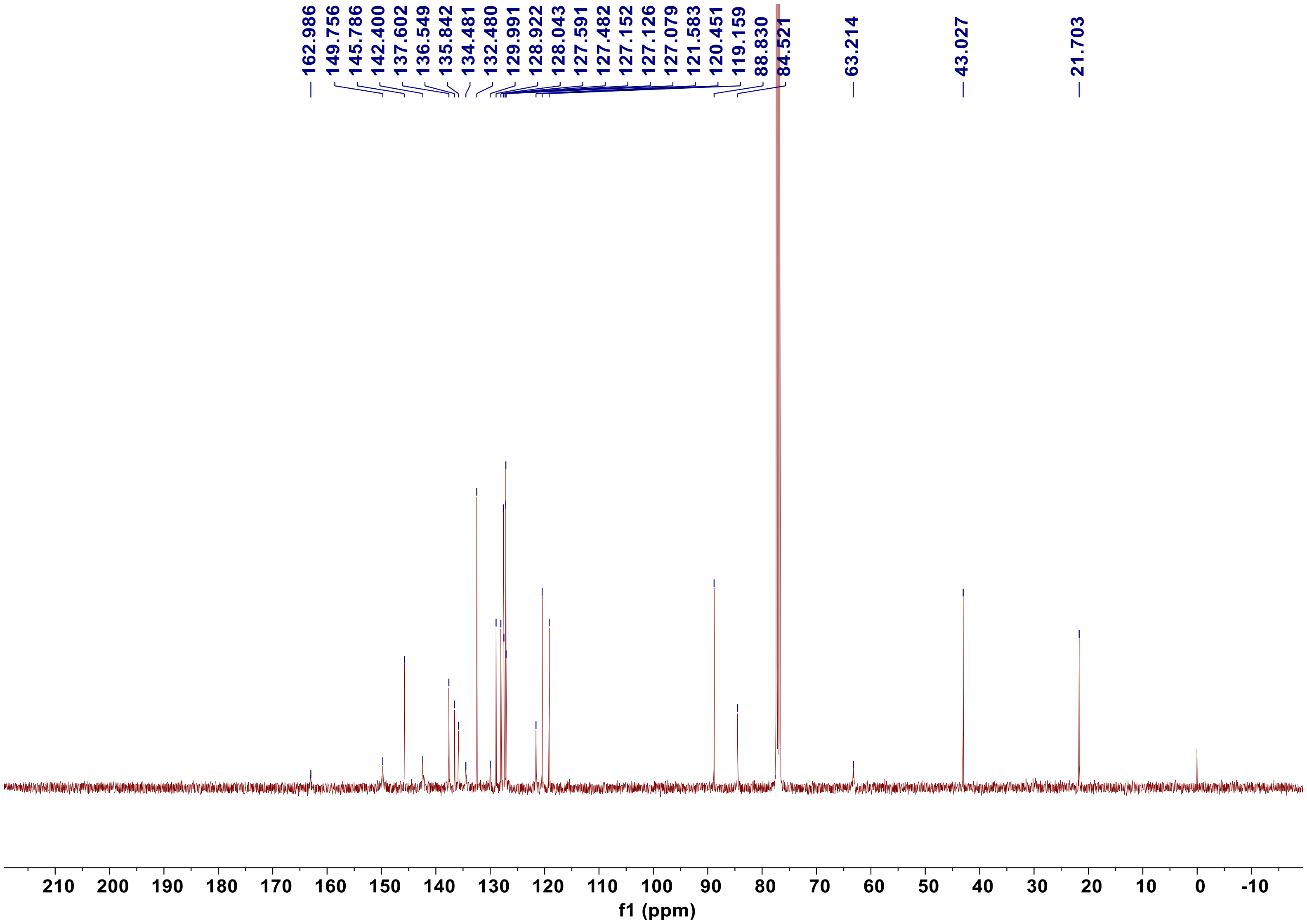

^{1}H NMR of **3c-1**

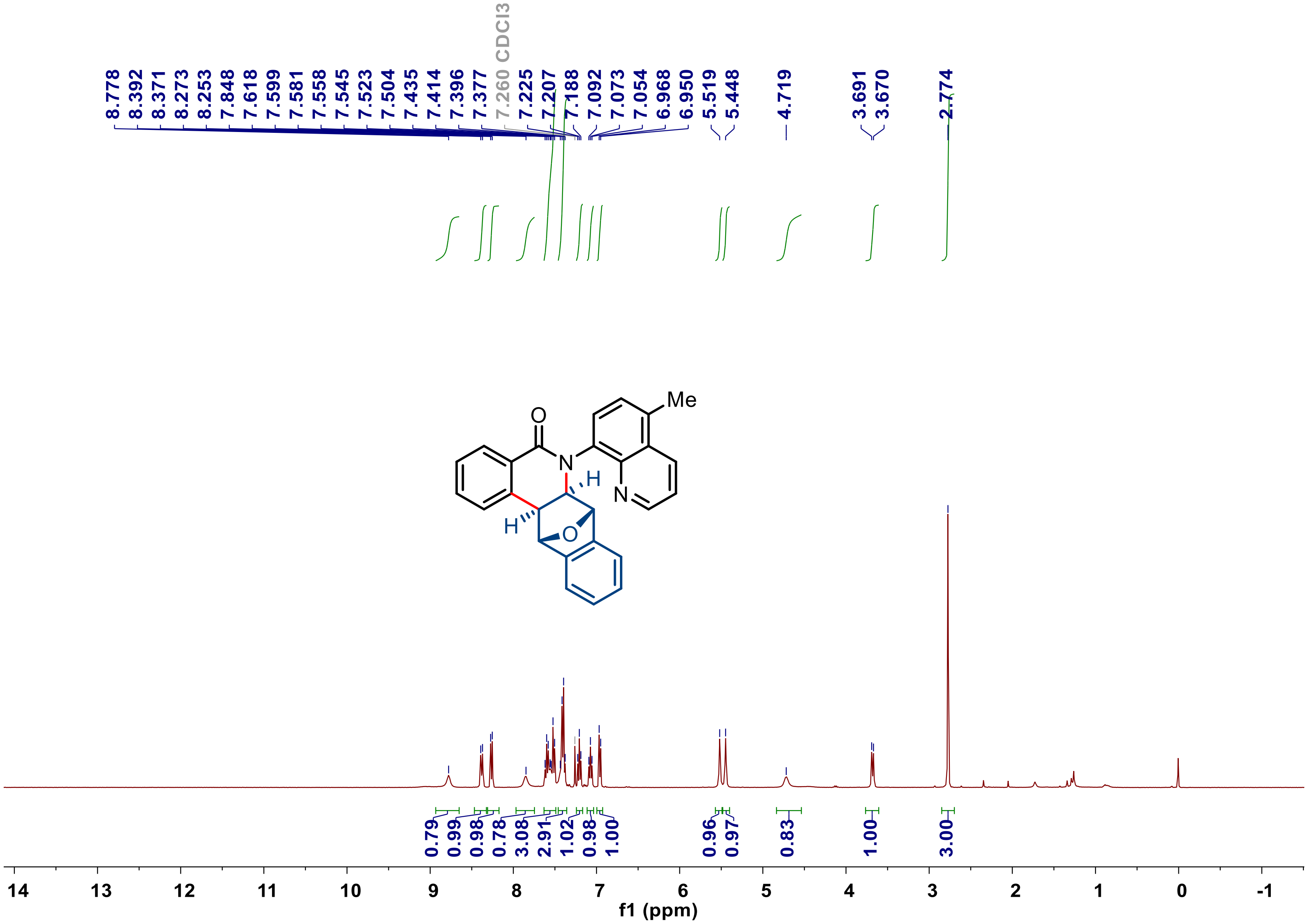
8.778
8.392
8.371
8.273
8.253
7.848
7.618
7.599
7.581
7.558
7.545
7.523
7.504
7.435
7.414
7.396
7.377
7.260 CDCl3
7.225
7.207
7.188
7.092
7.073
7.054
6.968
6.950
5.519
5.448
4.719
3.691
3.670
2.774
O
N
Me
H
N
H
O
0.79
0.99
0.98
0.78
3.08
2.91
1.02
0.98
1.00
0.96
0.97
0.83
1.00
3.00
14
13
12
11
10
9
8
7
6
5
4
3
2
1
0
-1
f1 (ppm)

^{13}C NMR of **3c-1**

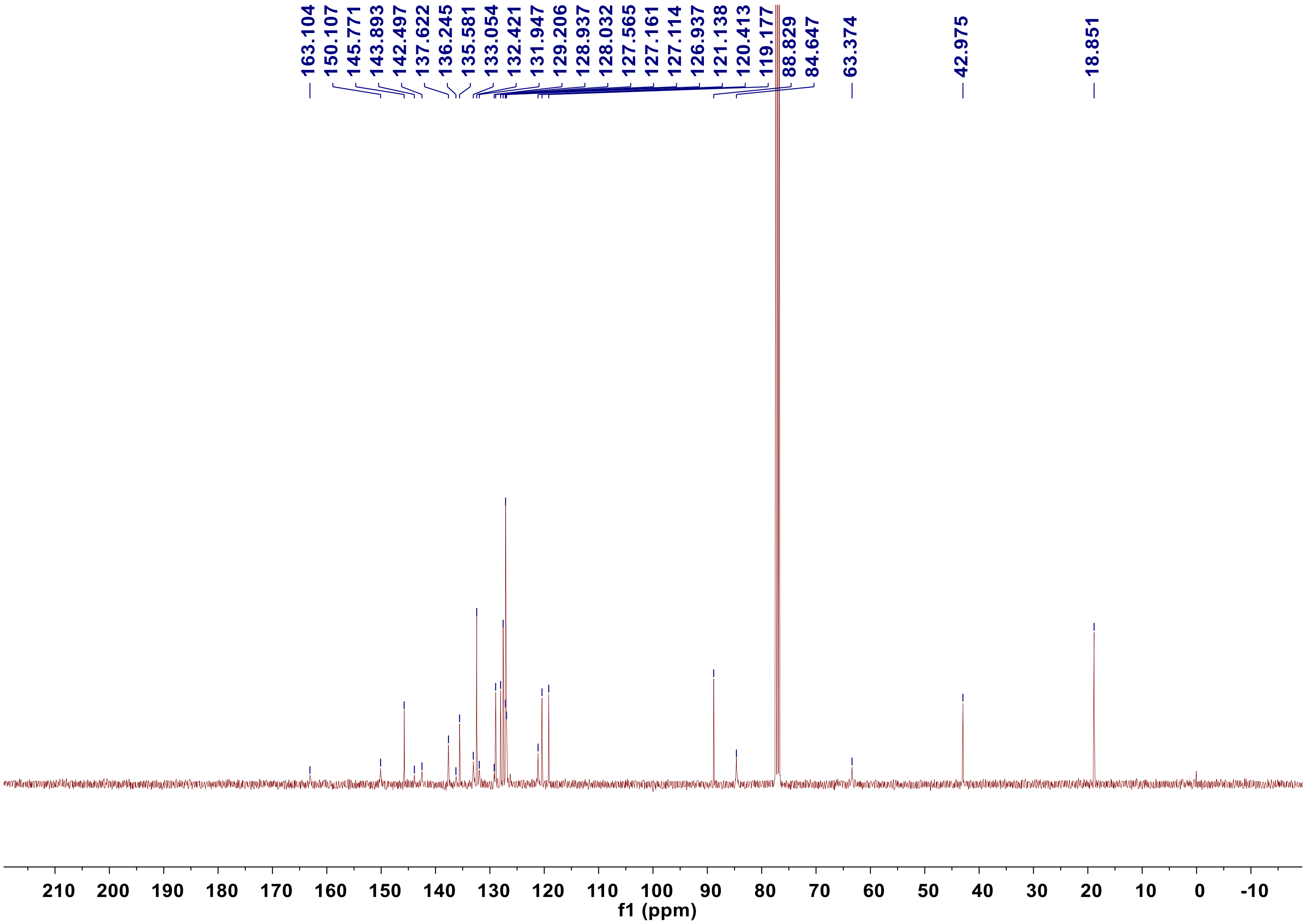

^{1}H NMR of **3d-1**

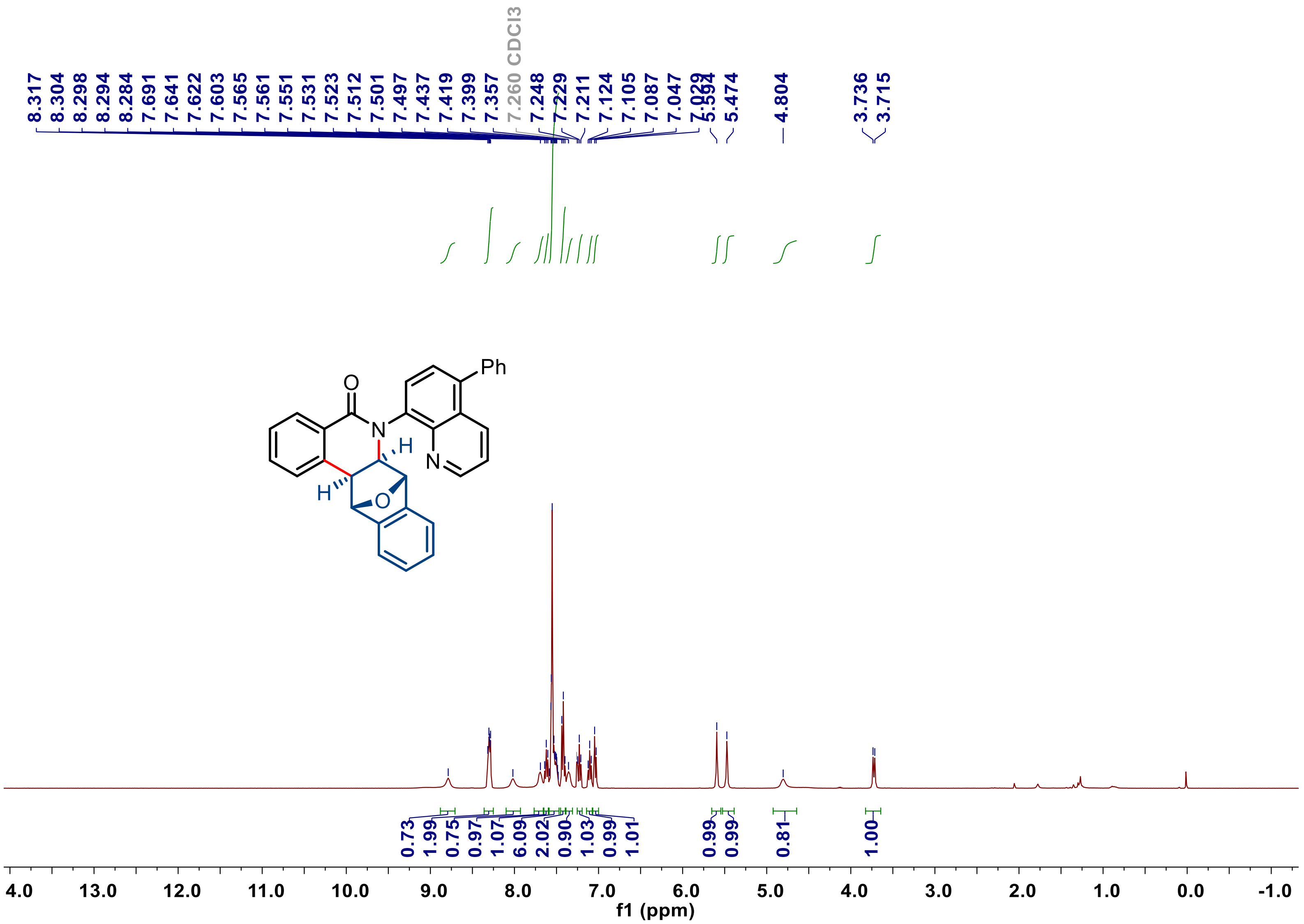
8.317
8.304
8.298
8.294
8.284
7.691
7.641
7.622
7.603
7.565
7.561
7.551
7.531
7.523
7.512
7.501
7.497
7.437
7.419
7.399
7.357
7.260 CDCl3
7.248
7.229
7.211
7.124
7.105
7.087
7.047
7.029
5.594
5.474
4.804
3.736
3.715
Ph
O
N
H
N
H
O
0.73
1.99
0.75
0.97
1.07
6.09
2.02
0.90
1.03
0.99
1.01
0.99
0.99
0.81
1.00
f1 (ppm)

^{13}C NMR of **3d-1**

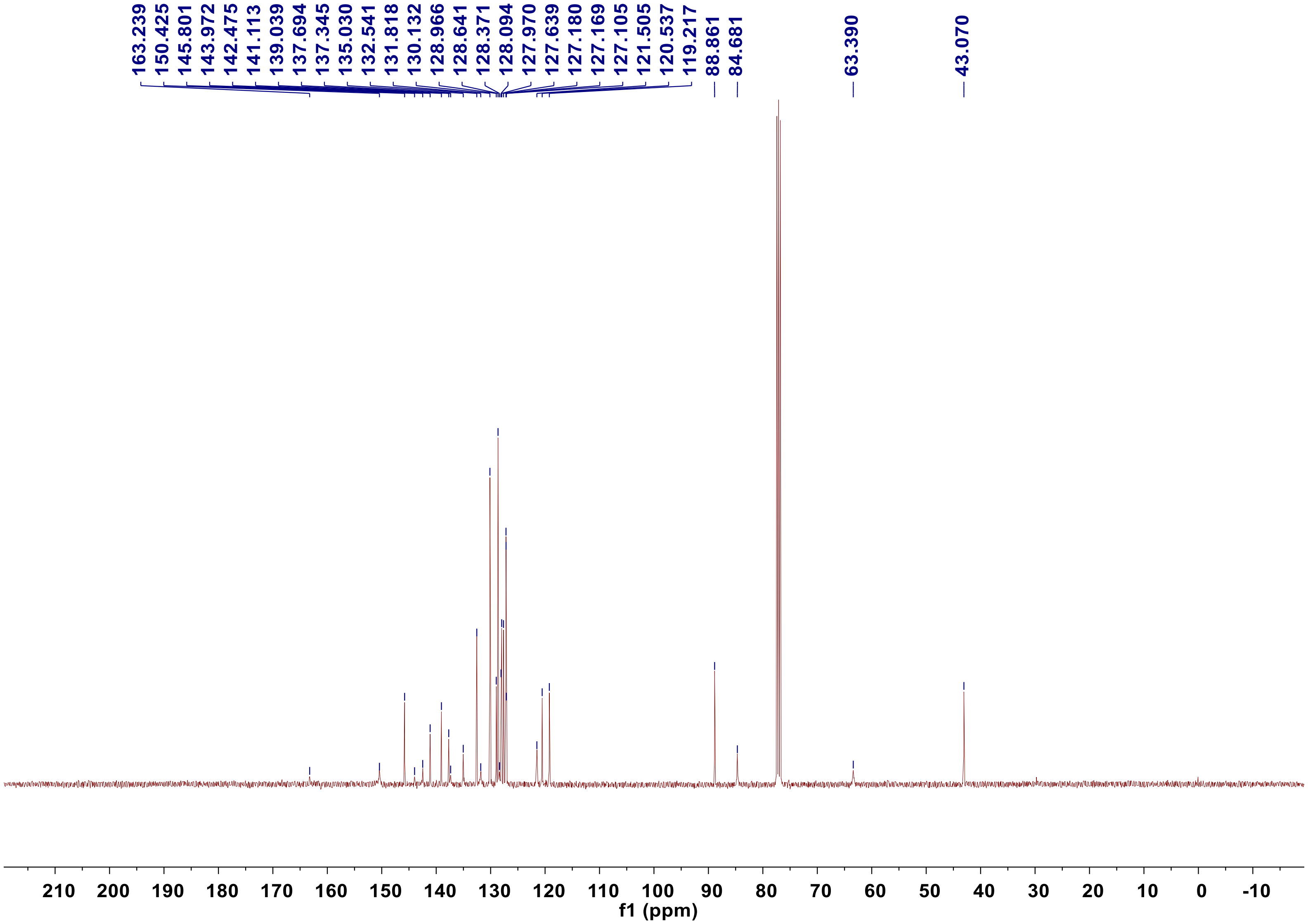

^{1}H NMR of **3e-1**

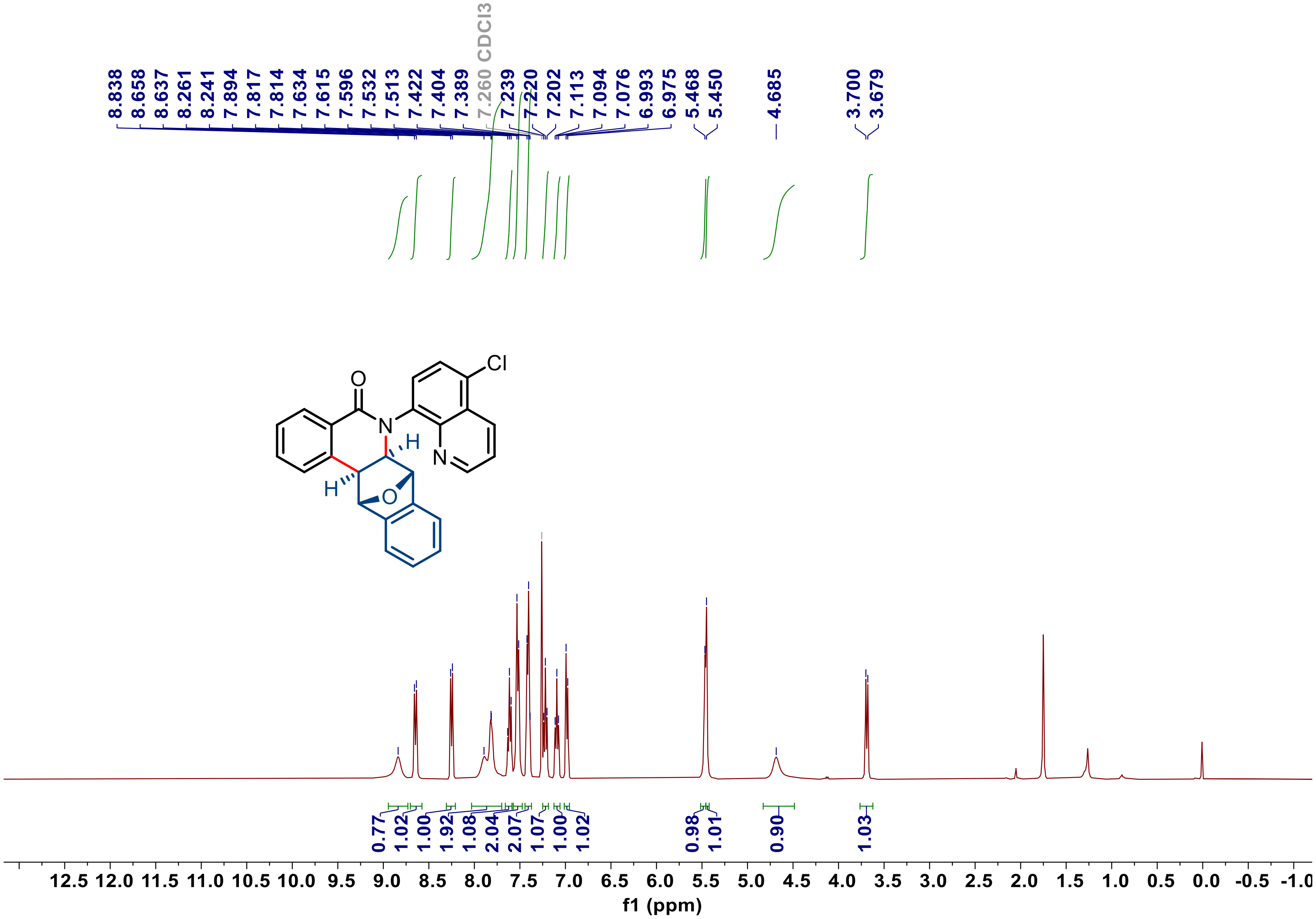
8.838
8.658
8.637
8.261
8.241
7.894
7.817
7.814
7.634
7.615
7.596
7.532
7.513
7.422
7.404
7.389
7.260 CDCl3
7.239
7.220
7.202
7.113
7.094
7.076
6.993
6.975
5.468
5.450
4.685
3.700
3.679
O
N
H
Cl
N
H
O
0.77
1.02
1.00
1.92
1.08
2.04
2.07
1.07
1.00
1.02
0.98
1.01
0.90
1.03
12.5 12.0 11.5 11.0 10.5 10.0 9.5 9.0 8.5 8.0 7.5 7.0 6.5 6.0 5.5 5.0 4.5 4.0 3.5 3.0 2.5 2.0 1.5 1.0 0.5 0.0 -0.5 -1.0
f1 (ppm)

^{13}C NMR of **3e-1**

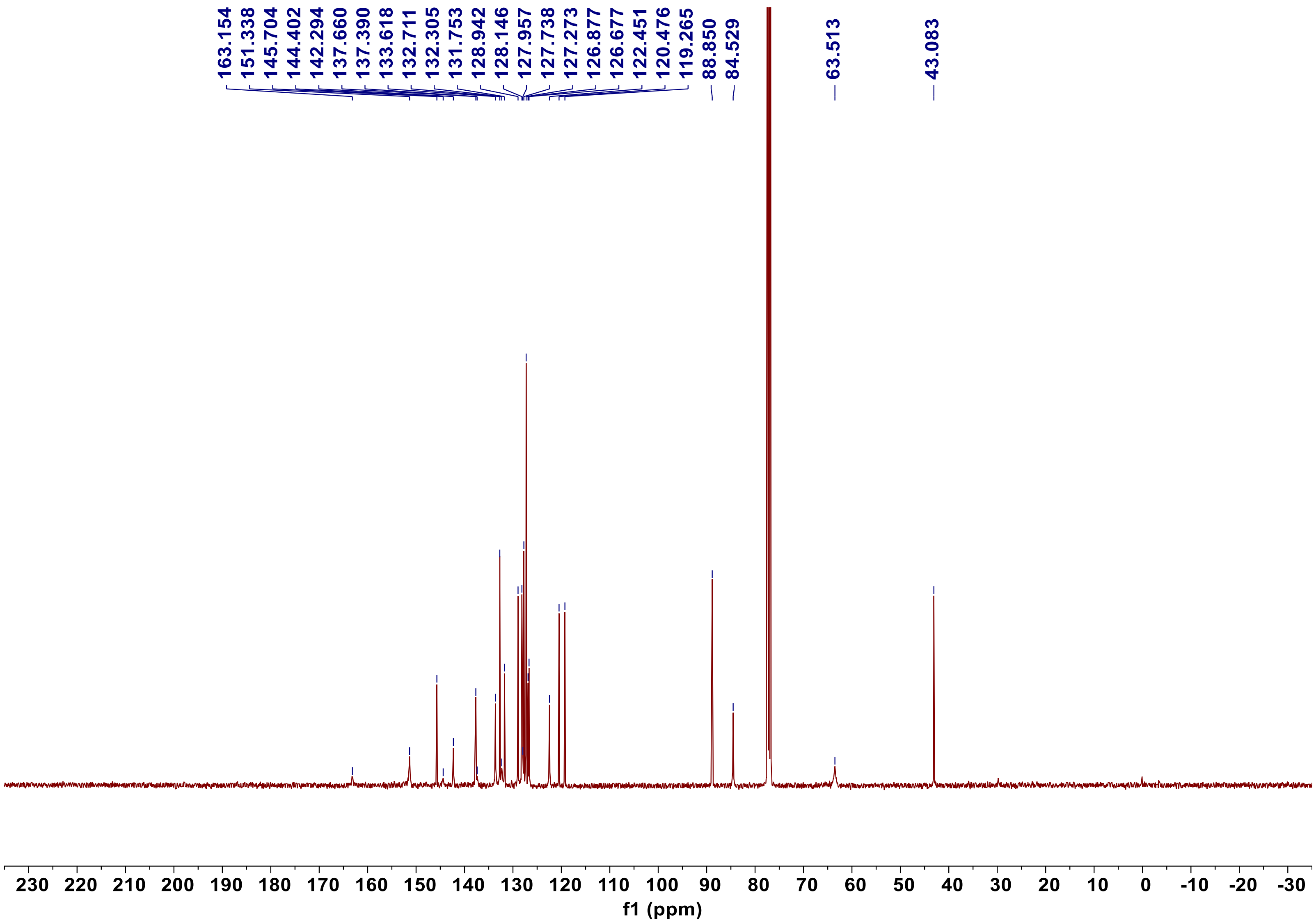

^{1}H NMR of **3f-1**

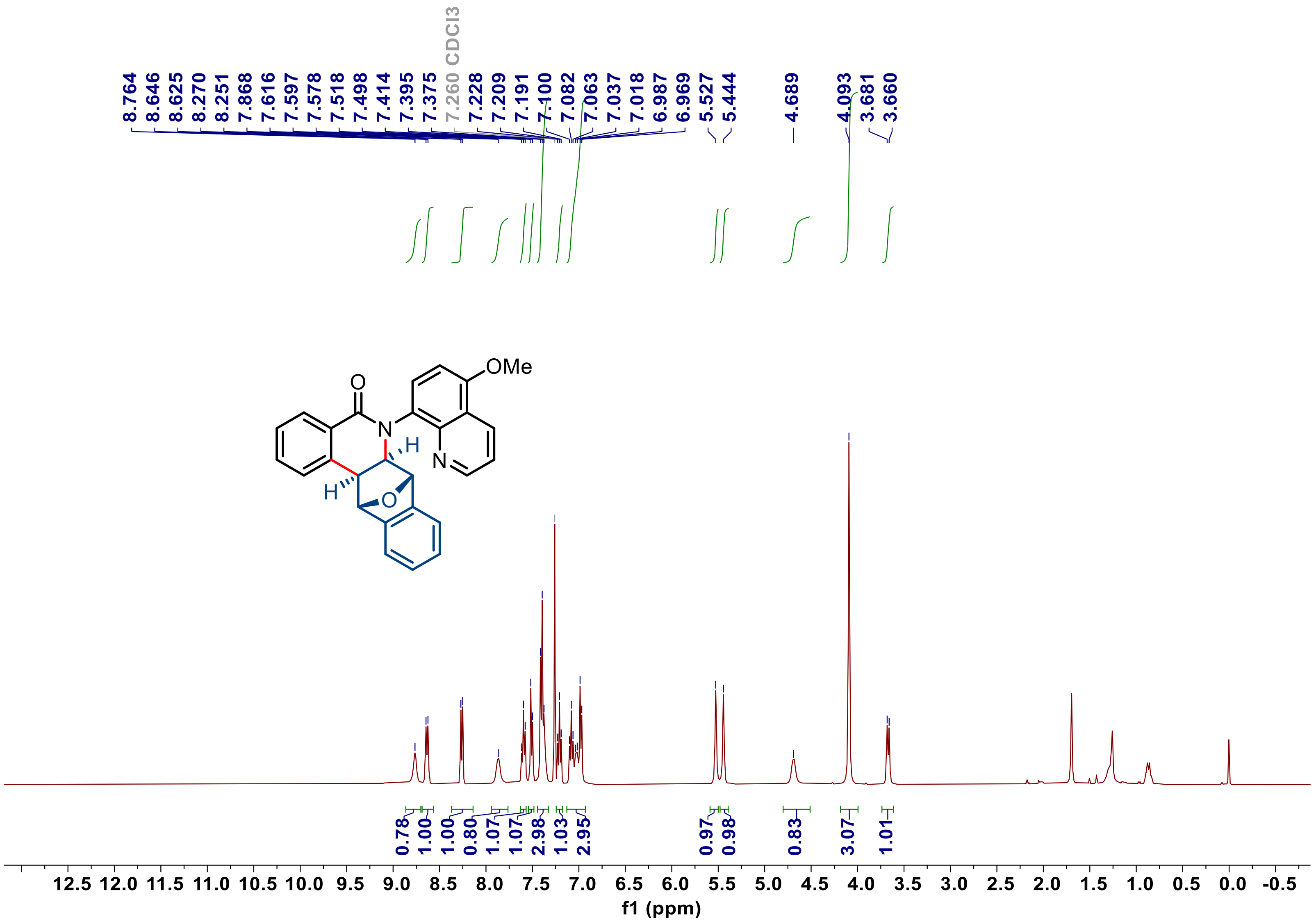

8.764
8.646
8.625
8.270
8.251
7.868
7.616
7.597
7.578
7.518
7.498
7.414
7.395
7.375
7.260 CDCl3
7.228
7.209
7.191
7.100
7.082
7.063
7.037
7.018
6.987
6.969
5.527
5.444
4.689
4.093
3.681
3.660
OMe
O
N
H
N
H
O
0.78
1.00
1.00
0.80
1.07
1.07
2.98
1.03
2.95
0.97
0.98
0.83
3.07
1.01
12.5 12.0 11.5 11.0 10.5 10.0 9.5 9.0 8.5 8.0 7.5 7.0 6.5 6.0 5.5 5.0 4.5 4.0 3.5 3.0 2.5 2.0 1.5 1.0 0.5 0.0 -0.5
f1 (ppm)

^{13}C NMR of **3f-1**

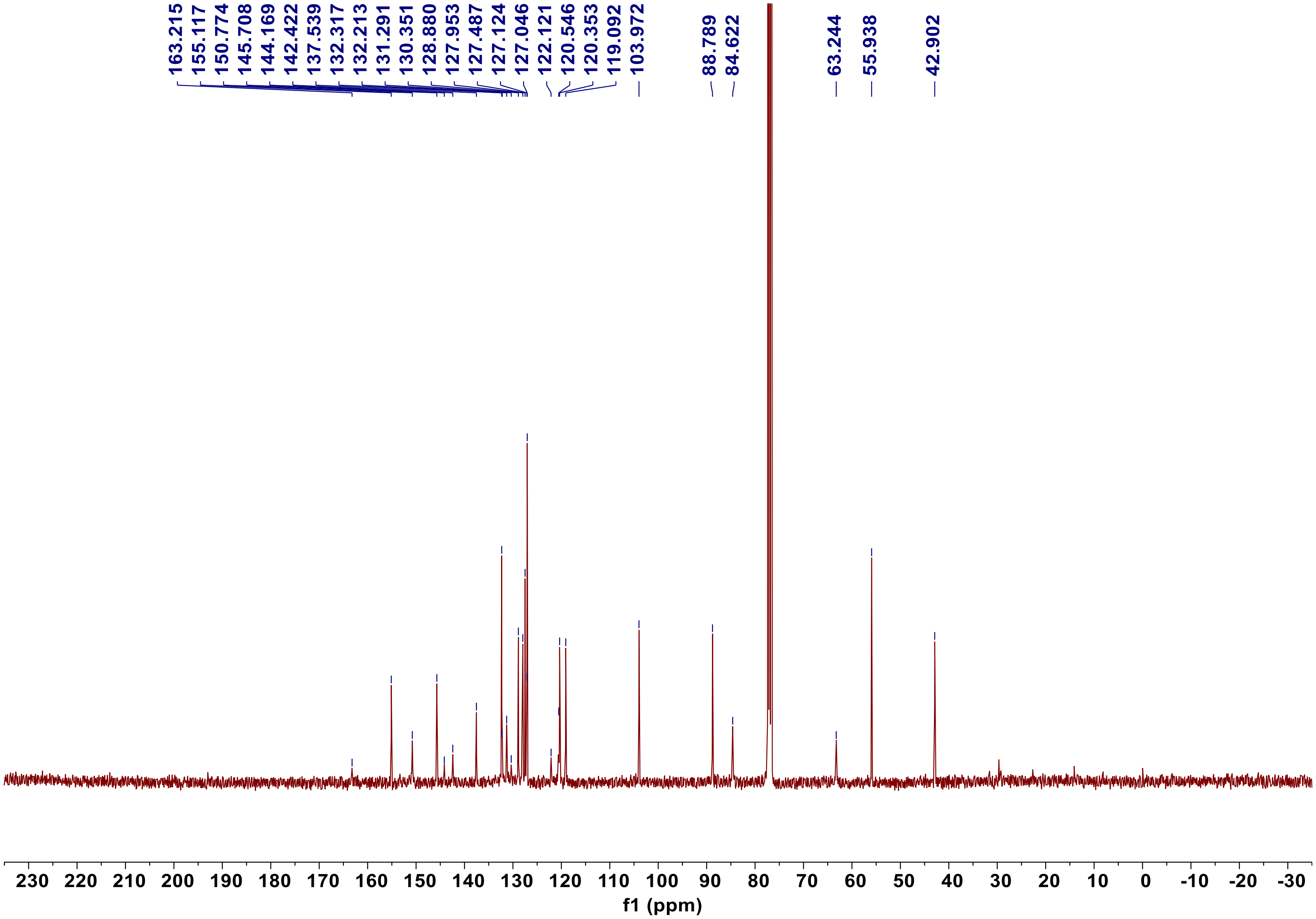

^{1}H NMR of **3g-1**

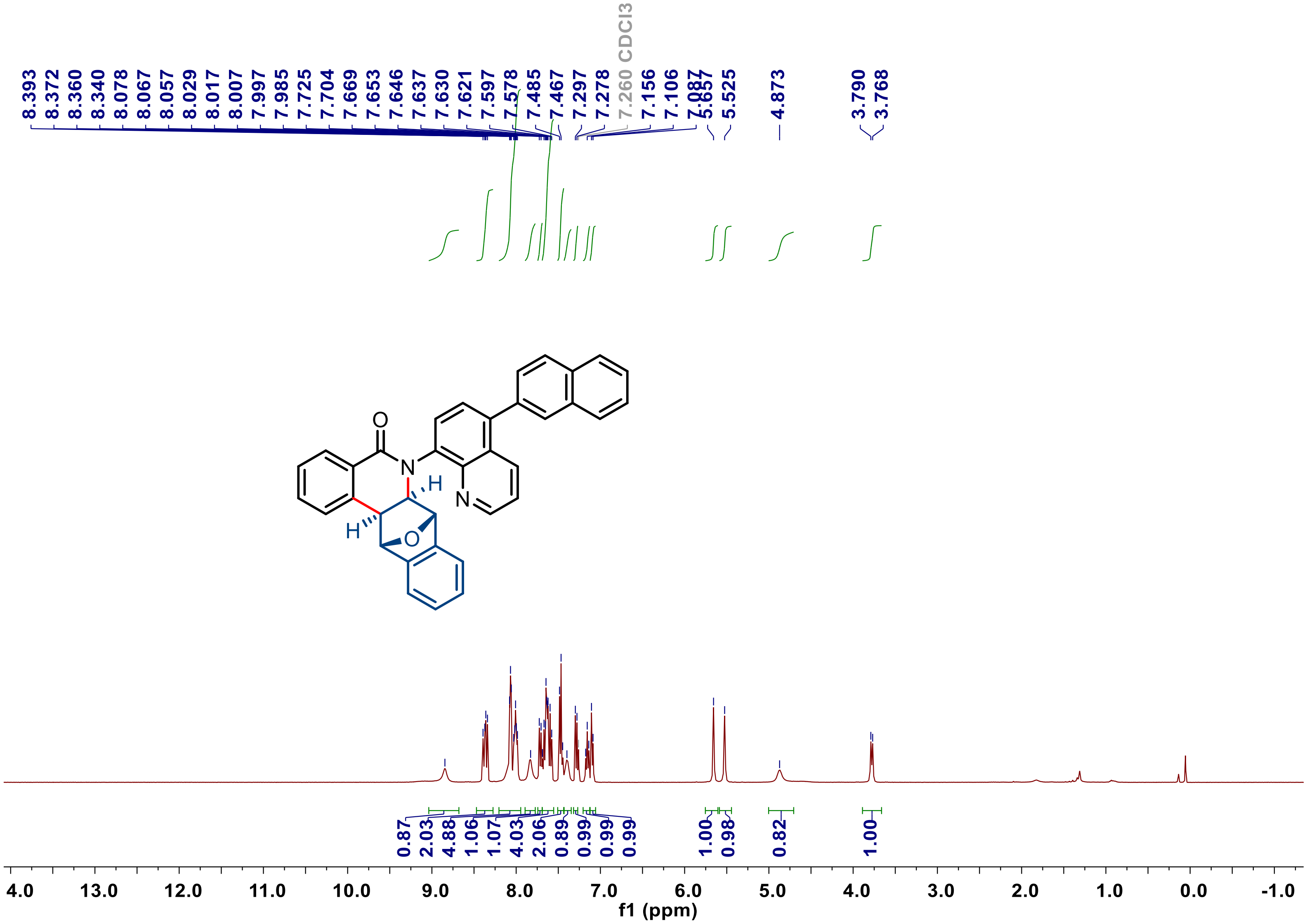

8.393
8.372
8.360
8.340
8.078
8.067
8.057
8.029
8.017
8.007
7.997
7.985
7.725
7.704
7.669
7.653
7.646
7.637
7.630
7.621
7.597
7.578
7.485
7.467
7.297
7.278
7.260 CDCl3
7.156
7.106
7.087
5.657
5.525
4.873
3.790
3.768
O
N
H
N
H
O
0.87
2.03
4.88
1.06
1.07
4.03
2.06
0.89
0.99
0.99
0.99
1.00
0.98
0.82
1.00
4.0 13.0 12.0 11.0 10.0 9.0 8.0 7.0 6.0 5.0 4.0 3.0 2.0 1.0 0.0 -1.0
f1 (ppm)

^{13}C NMR of **3g-1**

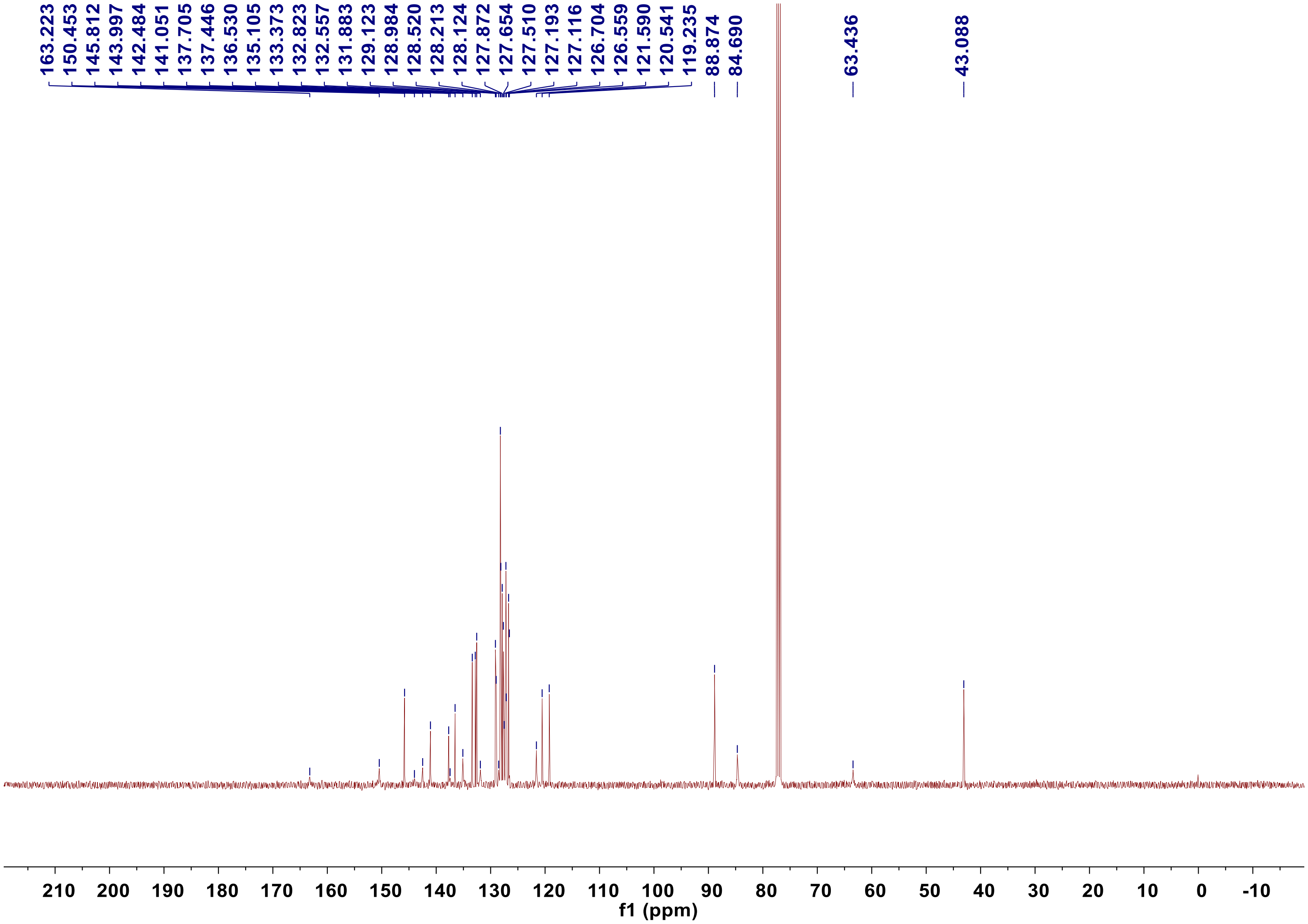

^{1}H NMR of **3h-1**

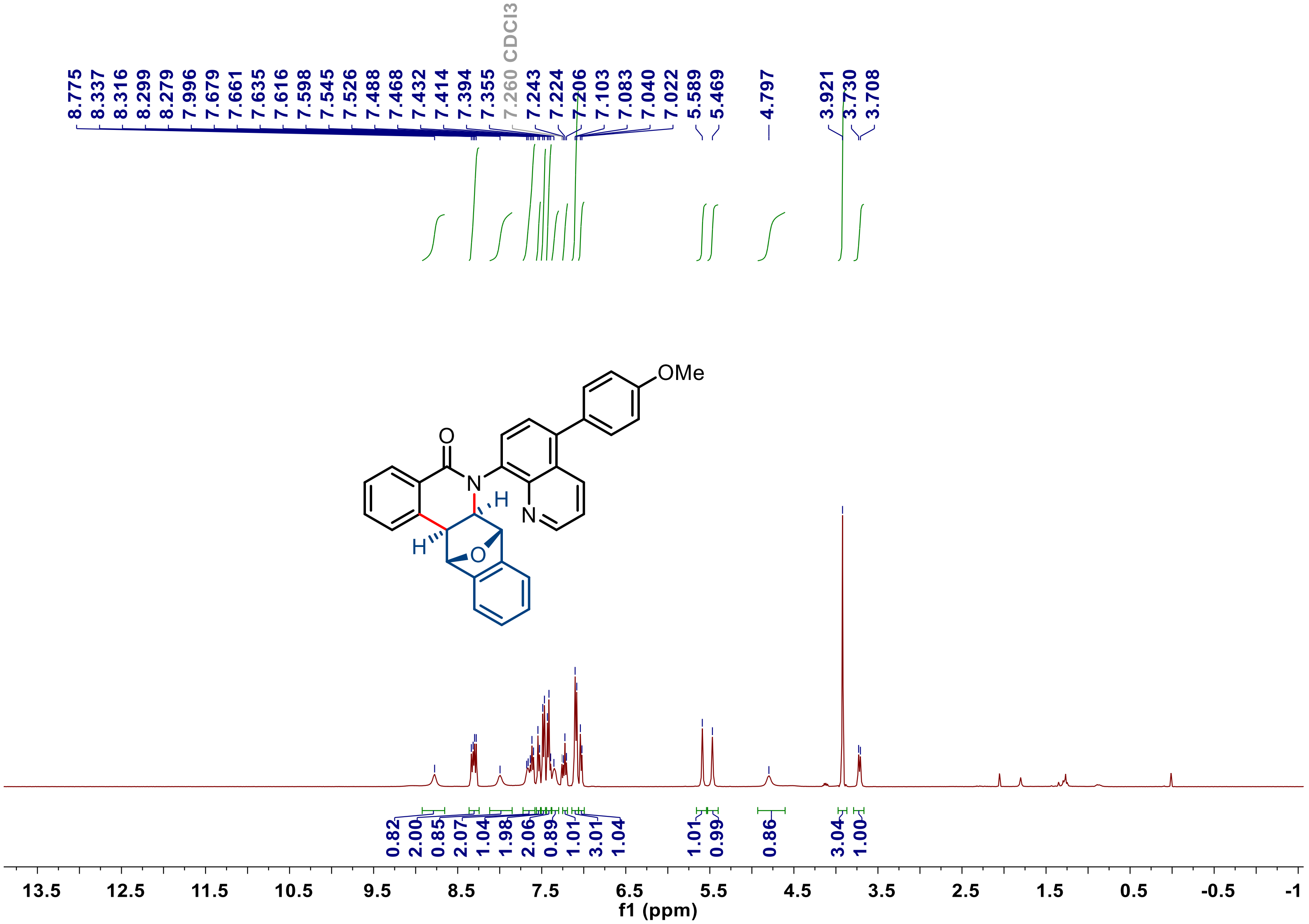
8.775
8.337
8.316
8.299
8.279
7.996
7.679
7.661
7.635
7.616
7.598
7.545
7.526
7.488
7.468
7.432
7.414
7.394
7.355
7.260 CDCl3
7.243
7.224
7.206
7.103
7.083
7.040
7.022
5.589
5.469
4.797
3.921
3.730
3.708
OMe
O
N
H
N
H
O
0.82
2.00
0.85
2.07
1.04
1.98
2.06
0.89
1.01
3.01
1.04
1.01
0.99
0.86
3.04
1.00
13.5
12.5
11.5
10.5
9.5
8.5
7.5
6.5
5.5
4.5
3.5
2.5
1.5
0.5
-0.5
-1
f1 (ppm)

^{13}C NMR of **3h-1**

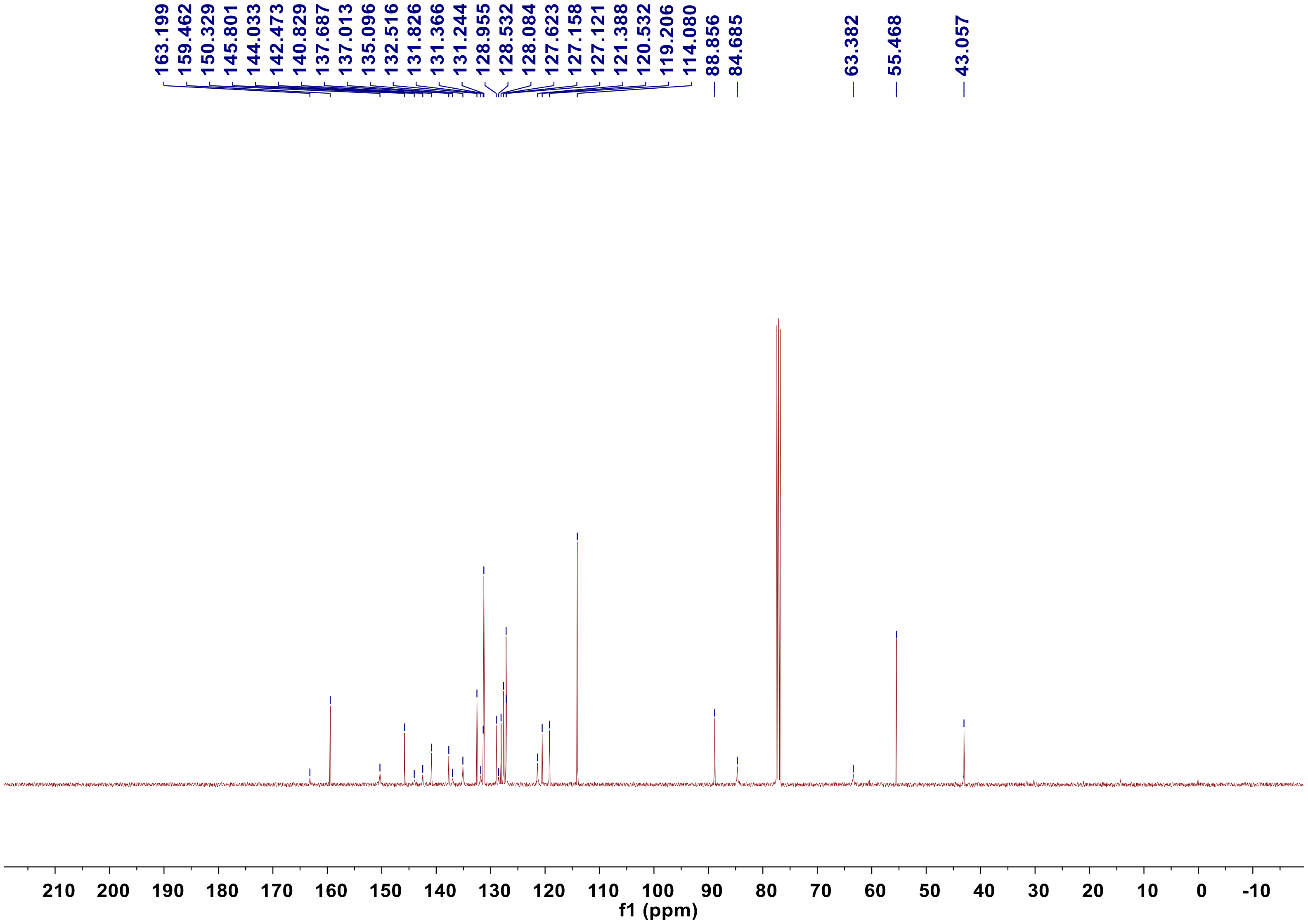

^{1}H NMR of **3i-1**

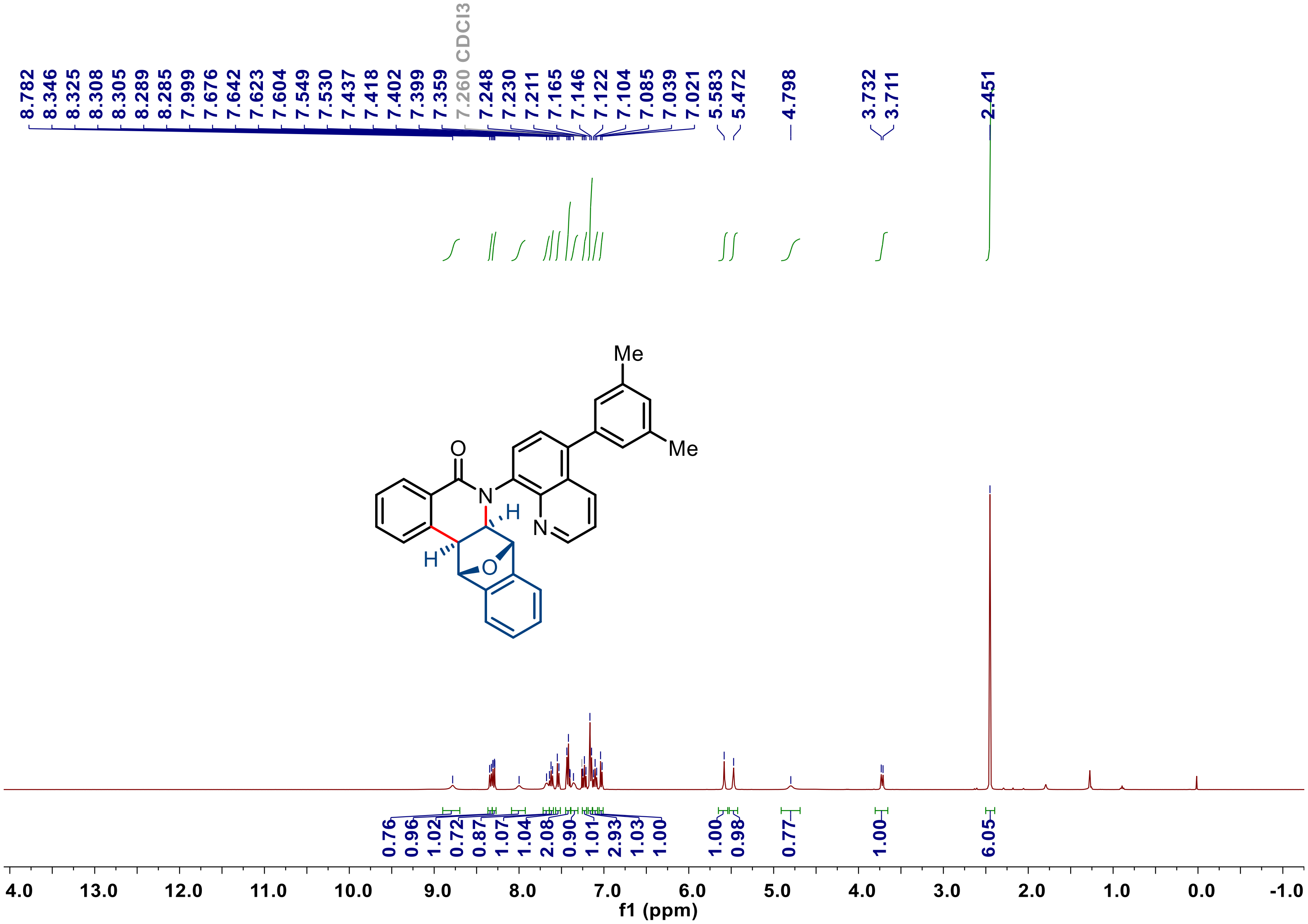
8.782 8.346 8.325 8.308 8.305 8.289 8.285 7.999 7.676 7.642 7.623 7.604 7.549 7.530 7.437 7.418 7.402 7.399 7.359 7.260 CDCl3 7.248 7.230 7.211 7.165 7.146 7.122 7.104 7.085 7.039 7.021 5.583 5.472 4.798 3.732 3.711 2.451
Me
Me
O
N
H
N
H
O
0.76 0.96 1.02 0.72 0.87 1.07 1.04 2.08 0.90 1.01 2.93 1.03 1.00 1.00 0.98 0.77 1.00 6.05
4.0 13.0 12.0 11.0 10.0 9.0 8.0 7.0 6.0 5.0 4.0 3.0 2.0 1.0 0.0 -1.0
f1 (ppm)

^{13}C NMR of **3i-1**

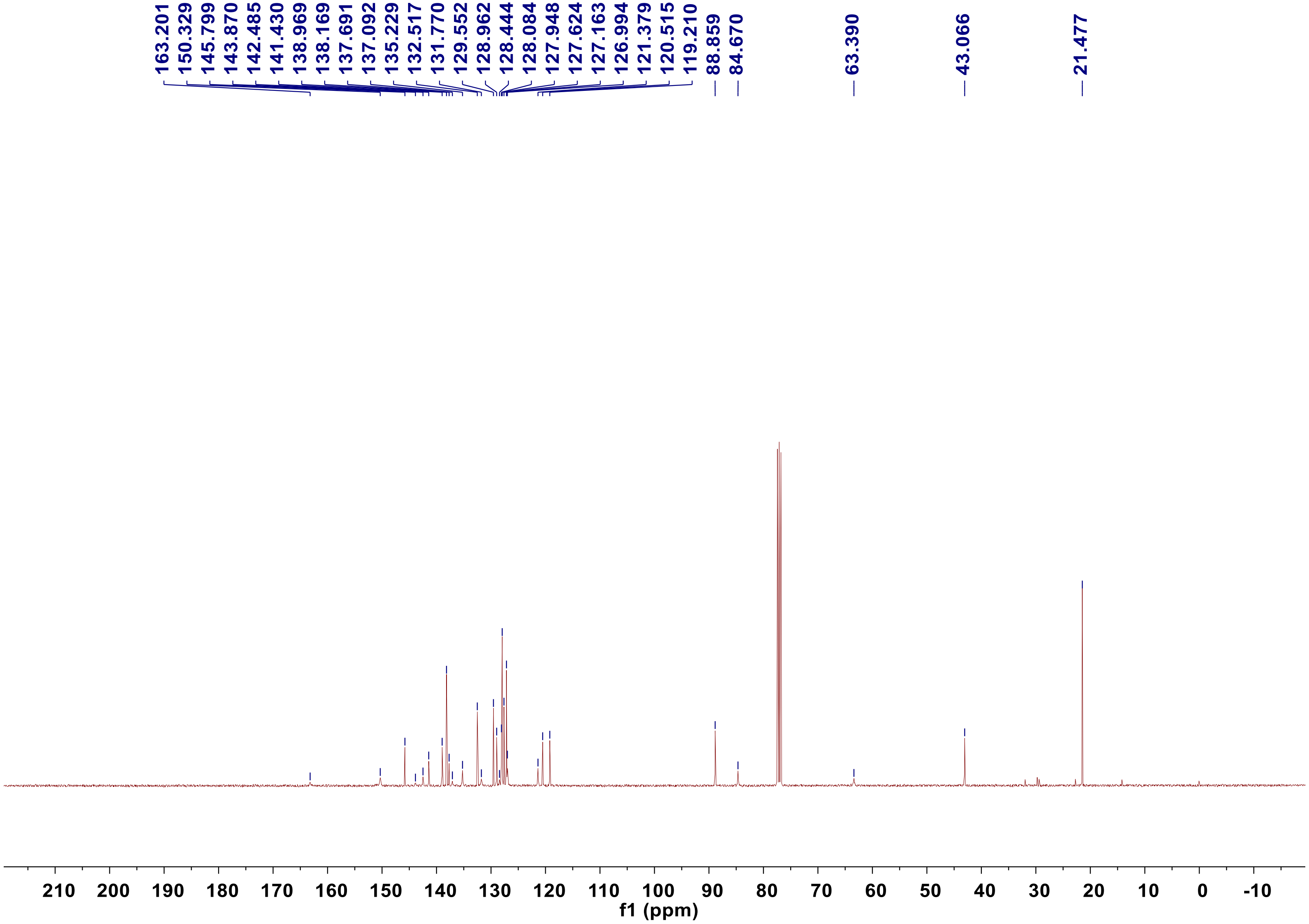

^{1}H NMR of **3j-1**

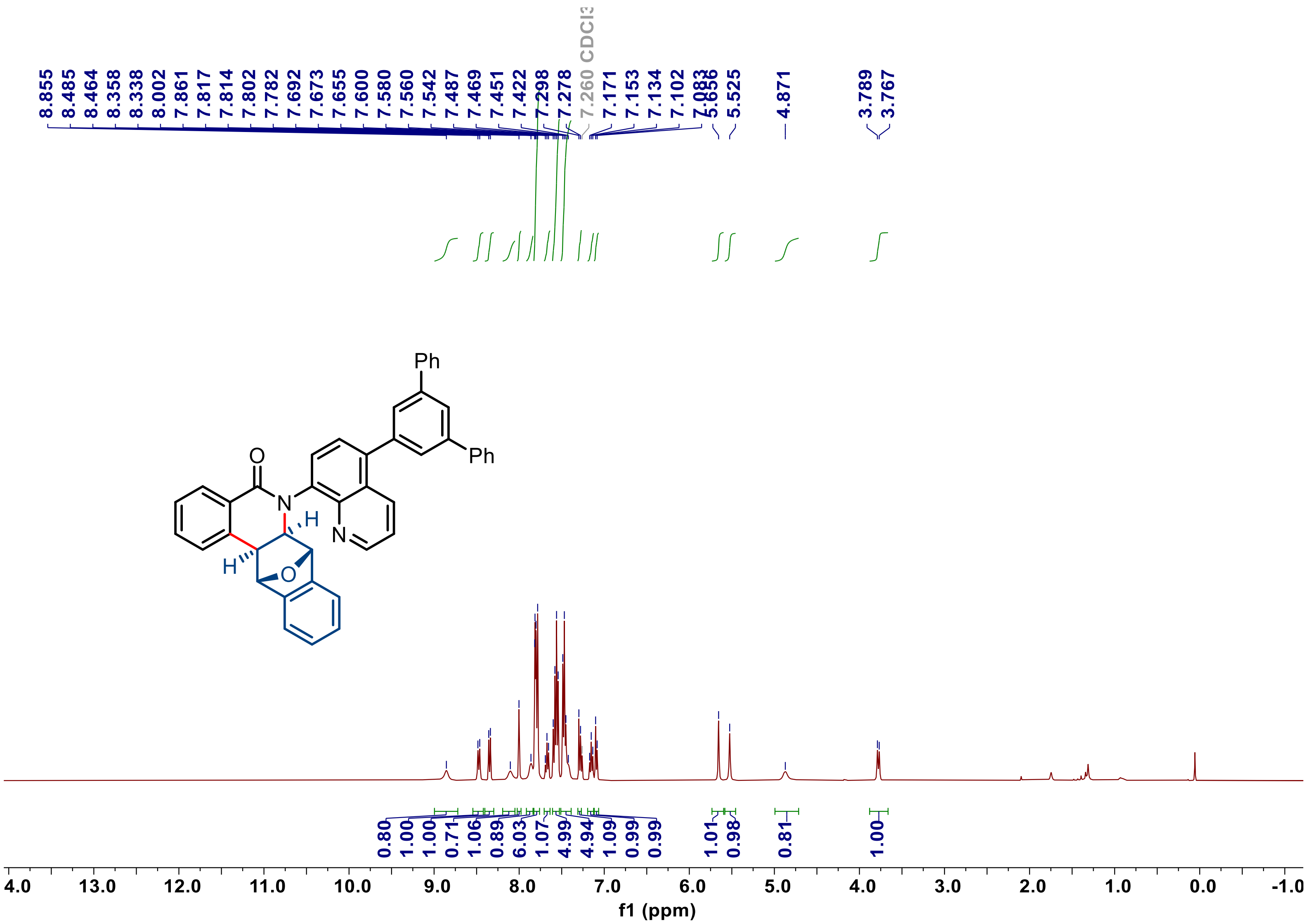
8.855
8.485
8.464
8.358
8.338
8.002
7.861
7.817
7.814
7.802
7.782
7.692
7.673
7.655
7.600
7.580
7.560
7.542
7.487
7.469
7.451
7.422
7.298
7.278
7.260 CDCl3
7.171
7.153
7.134
7.102
7.083
5.656
5.525
4.871
3.789
3.767
Ph
Ph
O
N
H
N
H
O
0.80
1.00
1.00
0.71
1.06
0.89
6.03
1.07
4.99
4.94
1.09
0.99
0.99
1.01
0.98
0.81
1.00
4.0
13.0
12.0
11.0
10.0
9.0
8.0
7.0
6.0
5.0
4.0
3.0
2.0
1.0
0.0
-1.0
f1 (ppm)

^{13}C NMR of **3j-1**

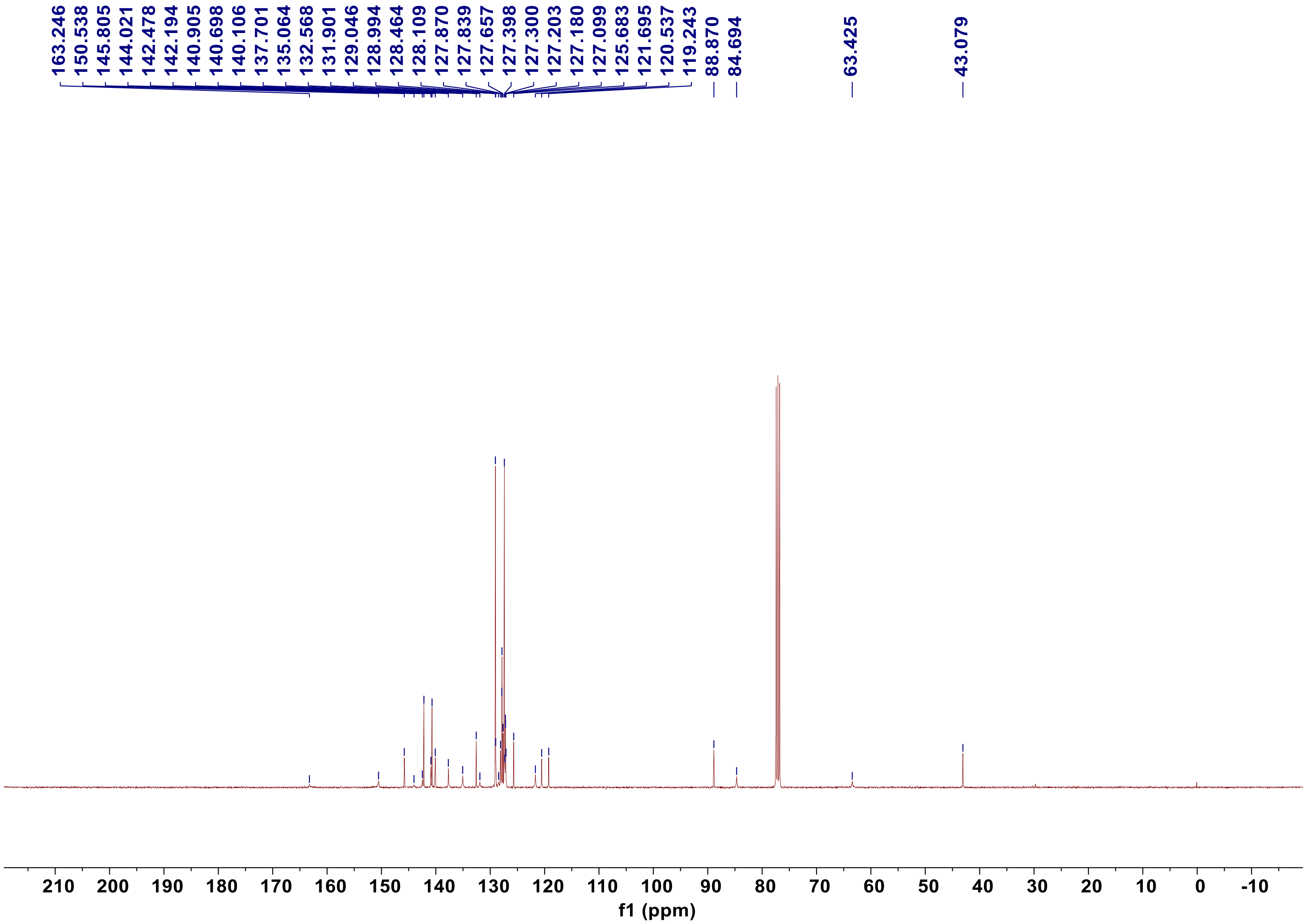

^{1}H NMR of **3g-2**

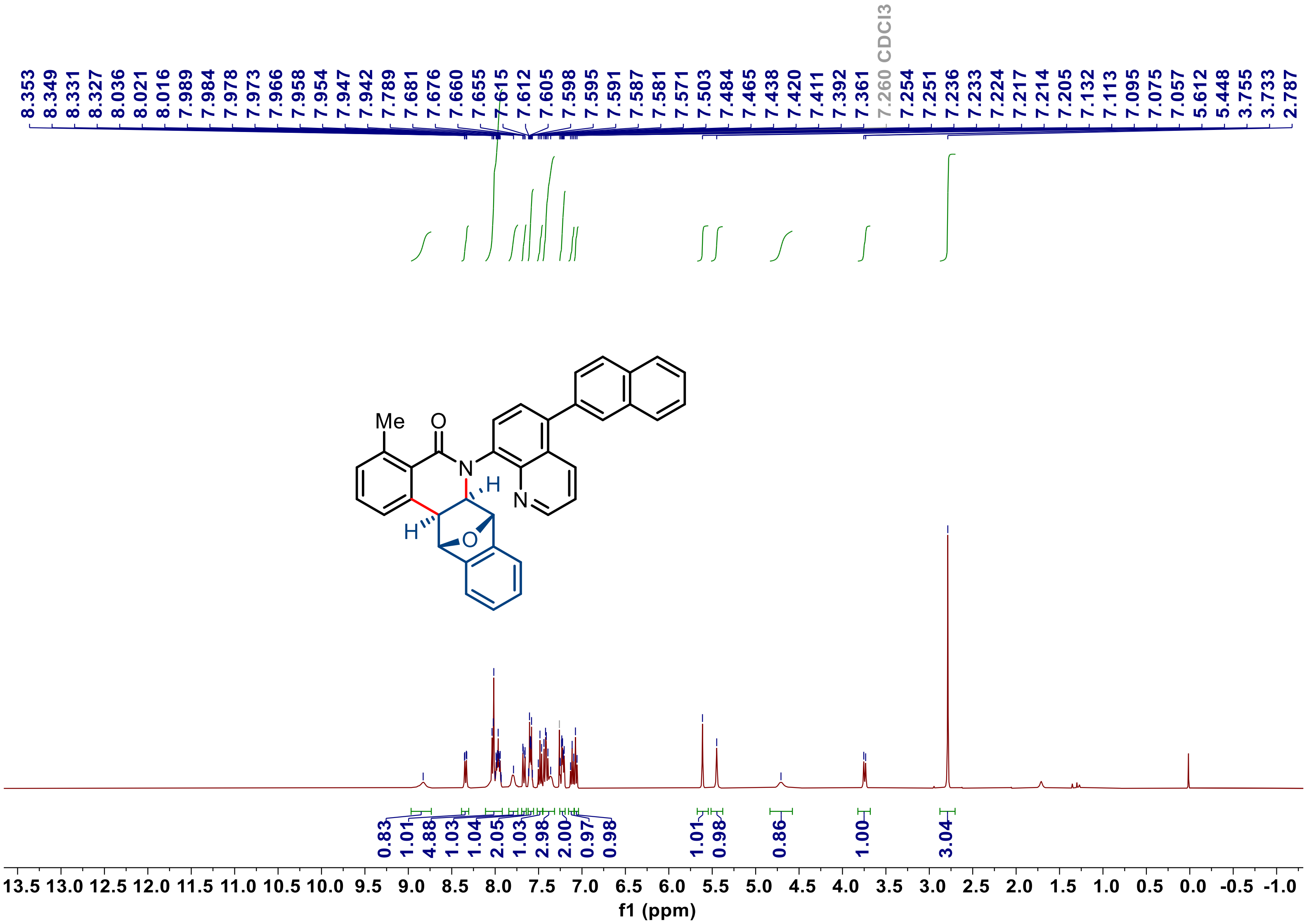

8.353
8.349
8.331
8.327
8.036
8.021
8.016
7.989
7.984
7.978
7.973
7.966
7.958
7.954
7.947
7.942
7.789
7.681
7.676
7.660
7.655
7.615
7.612
7.605
7.598
7.595
7.591
7.587
7.581
7.571
7.503
7.484
7.465
7.438
7.420
7.411
7.392
7.361
7.260 CDCl3
7.254
7.251
7.236
7.233
7.224
7.217
7.214
7.205
7.132
7.113
7.095
7.075
7.057
5.612
5.448
3.755
3.733
2.787
Me
O
N
H
N
H
O
0.83
1.01
4.88
1.03
1.04
2.05
1.03
2.98
2.00
0.97
0.98
1.01
0.98
0.86
1.00
3.04
13.5 13.0 12.5 12.0 11.5 11.0 10.5 10.0 9.5 9.0 8.5 8.0 7.5 7.0 6.5 6.0 5.5 5.0 4.5 4.0 3.5 3.0 2.5 2.0 1.5 1.0 0.5 0.0 -0.5 -1.0
f1 (ppm)

^{13}C NMR of **3g-2**

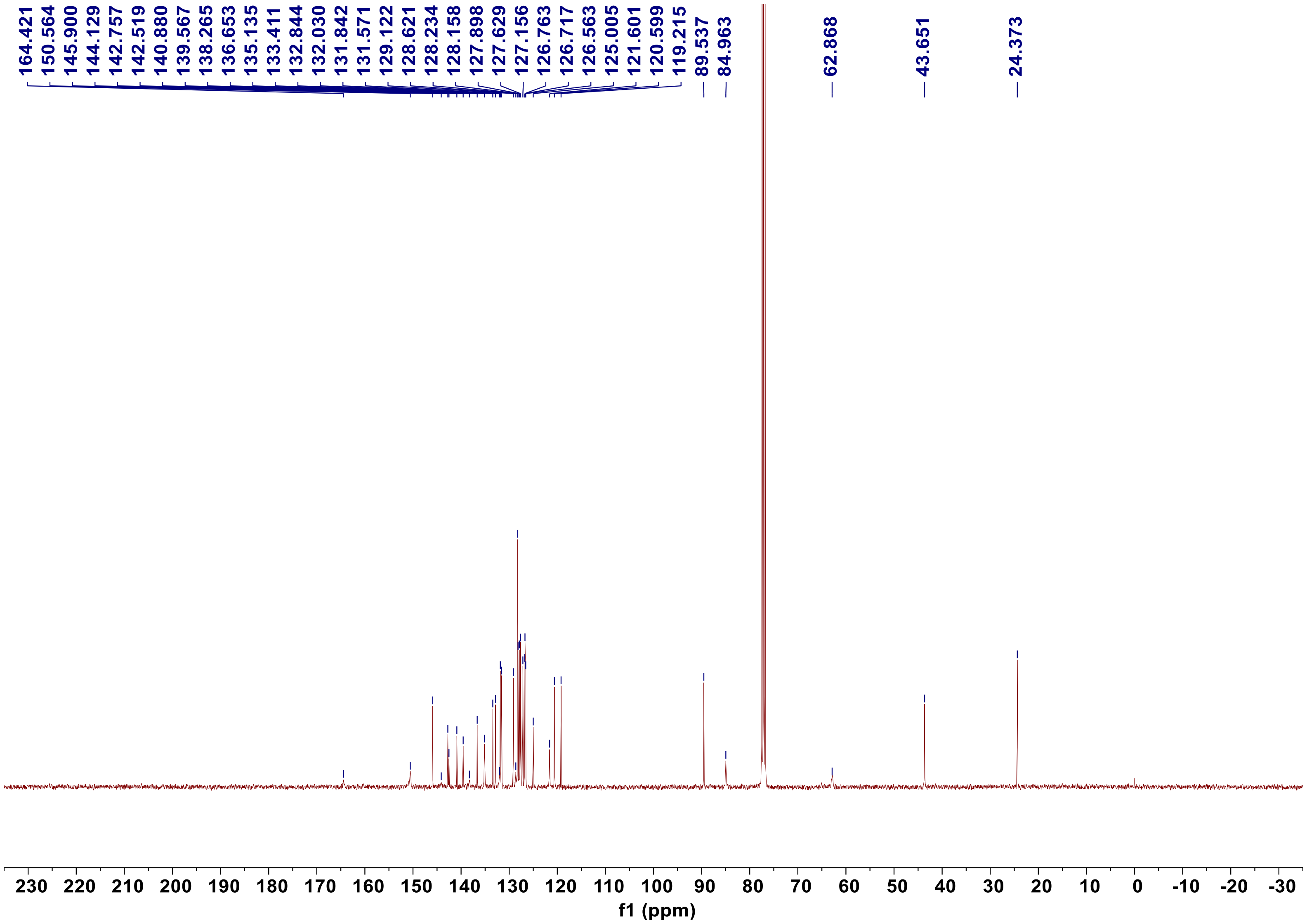

^{1}H NMR of **3g-3**

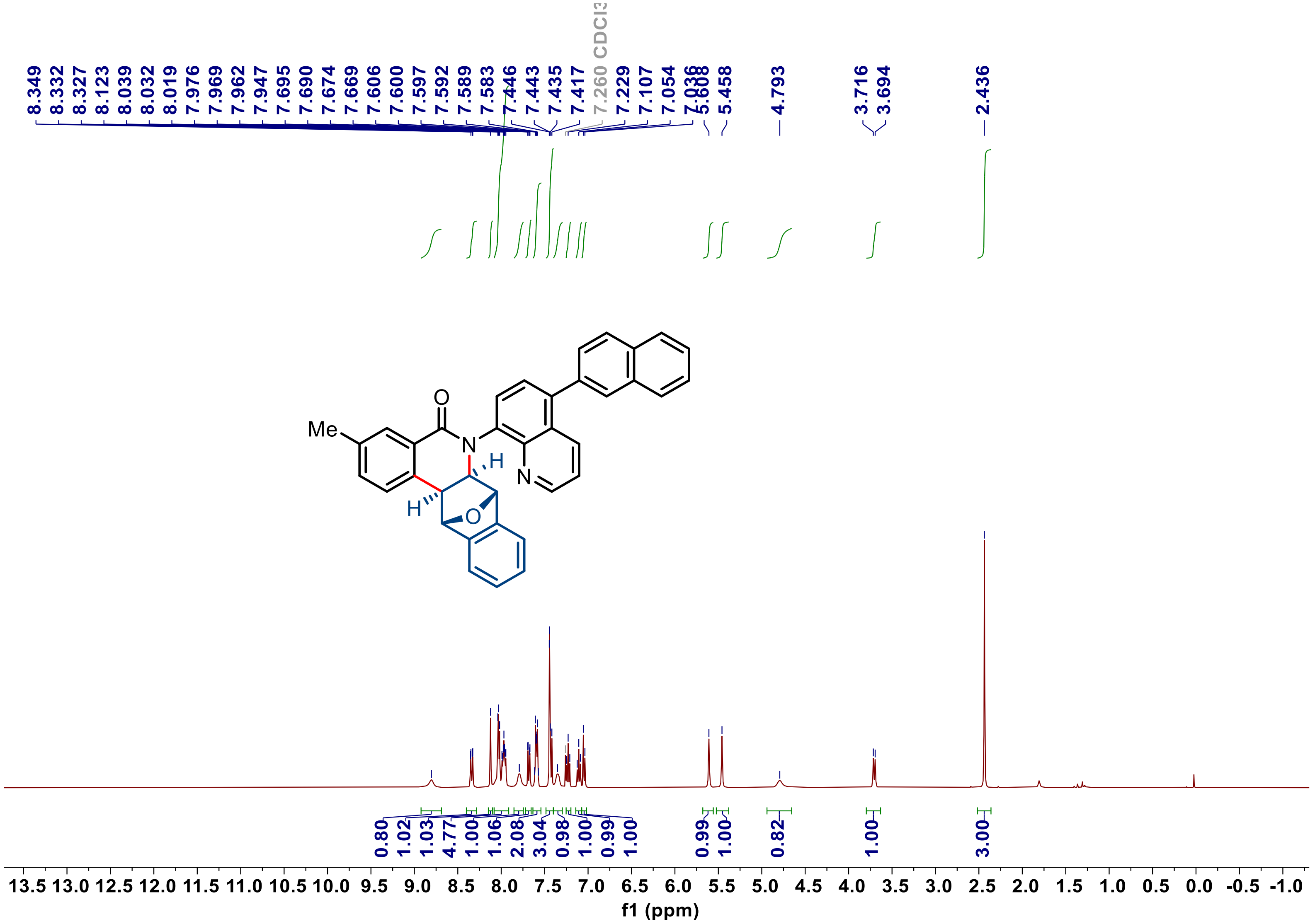
8.349
8.332
8.327
8.123
8.039
8.032
8.019
7.976
7.969
7.962
7.947
7.695
7.690
7.674
7.669
7.606
7.600
7.597
7.592
7.589
7.583
7.446
7.443
7.435
7.417
7.260 CDCl3
7.229
7.107
7.054
7.036
5.608
5.458
4.793
3.716
3.694
2.436
Me
O
N
H
N
H
O
0.80
1.02
1.03
4.77
1.00
1.06
2.08
3.04
0.98
1.00
0.99
1.00
0.99
1.00
0.82
1.00
3.00
13.5 13.0 12.5 12.0 11.5 11.0 10.5 10.0 9.5 9.0 8.5 8.0 7.5 7.0 6.5 6.0 5.5 5.0 4.5 4.0 3.5 3.0 2.5 2.0 1.5 1.0 0.5 0.0 -0.5 -1.0
f1 (ppm)

^{13}C NMR of **3g-3**

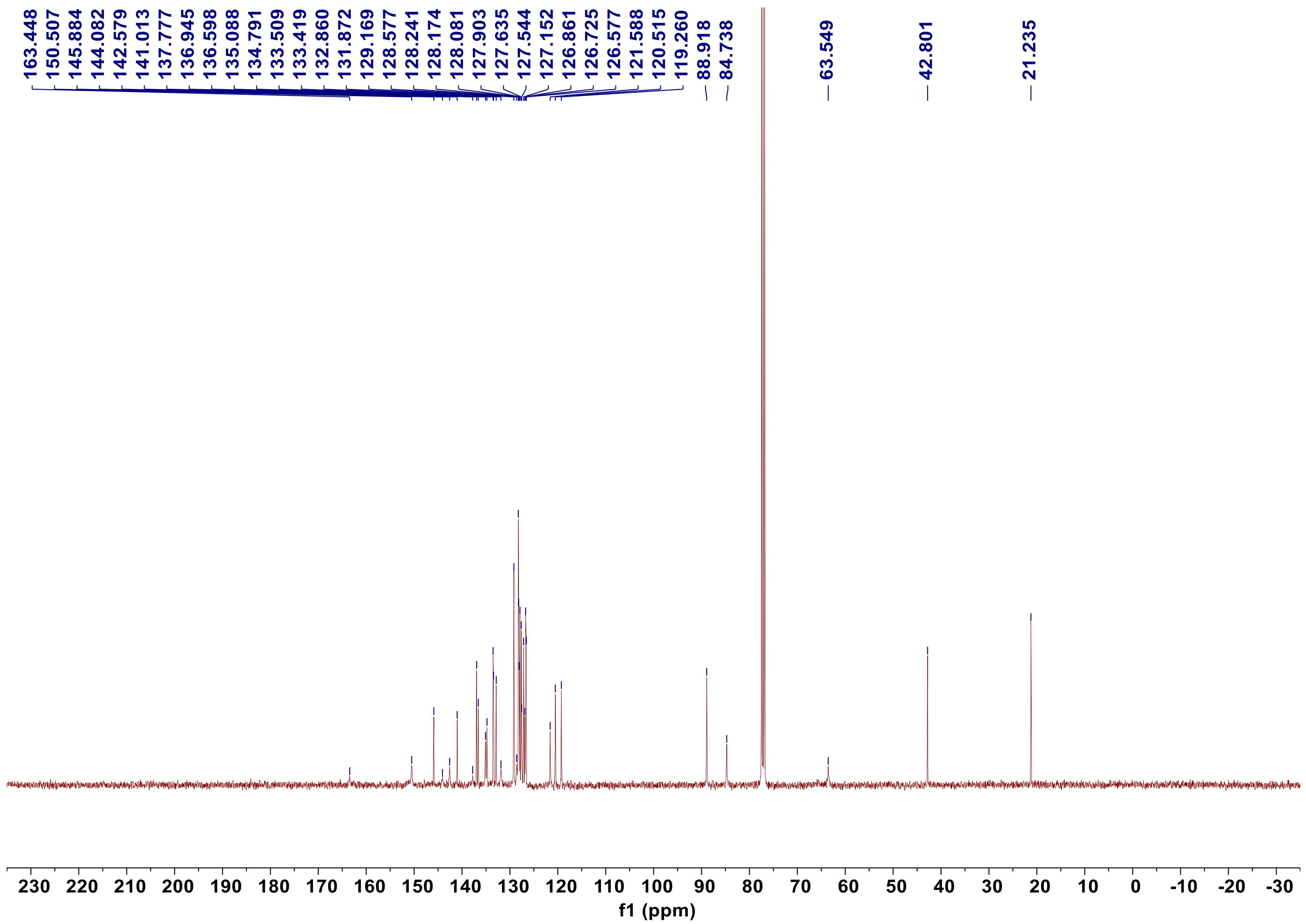

^{1}H NMR of **3g-4**

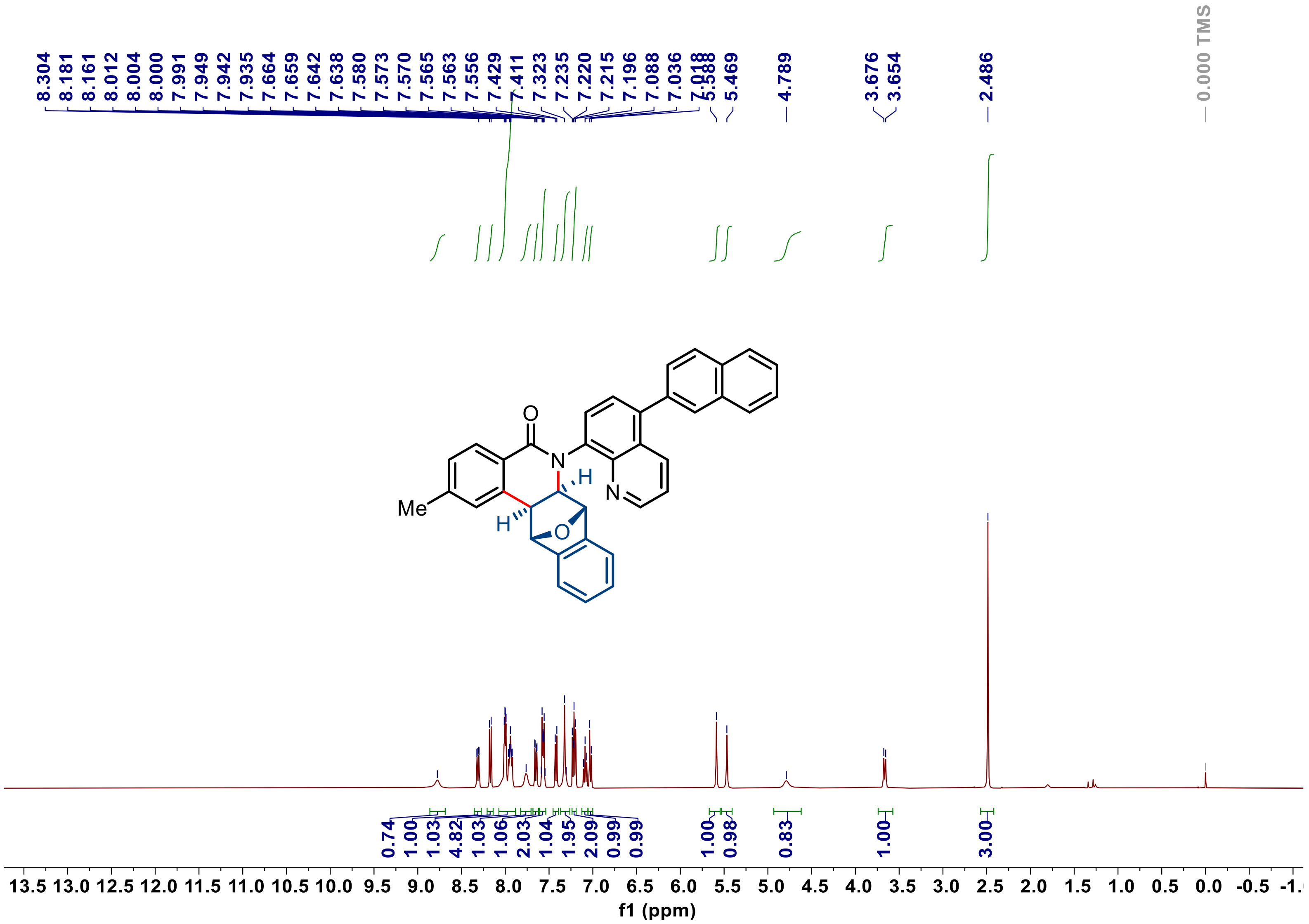

^{13}C NMR of **3g-4**

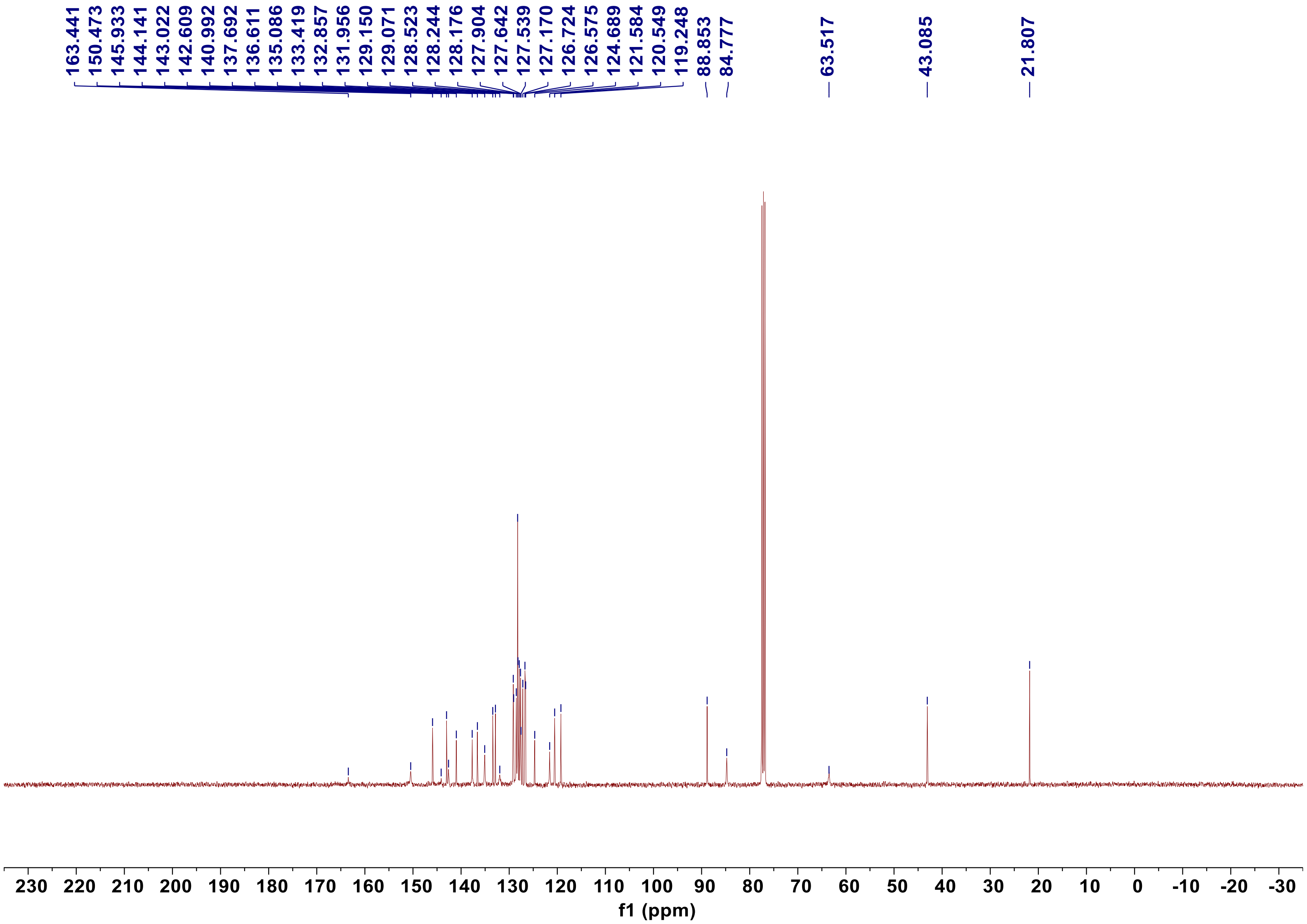

^{1}H NMR of **3g-5**

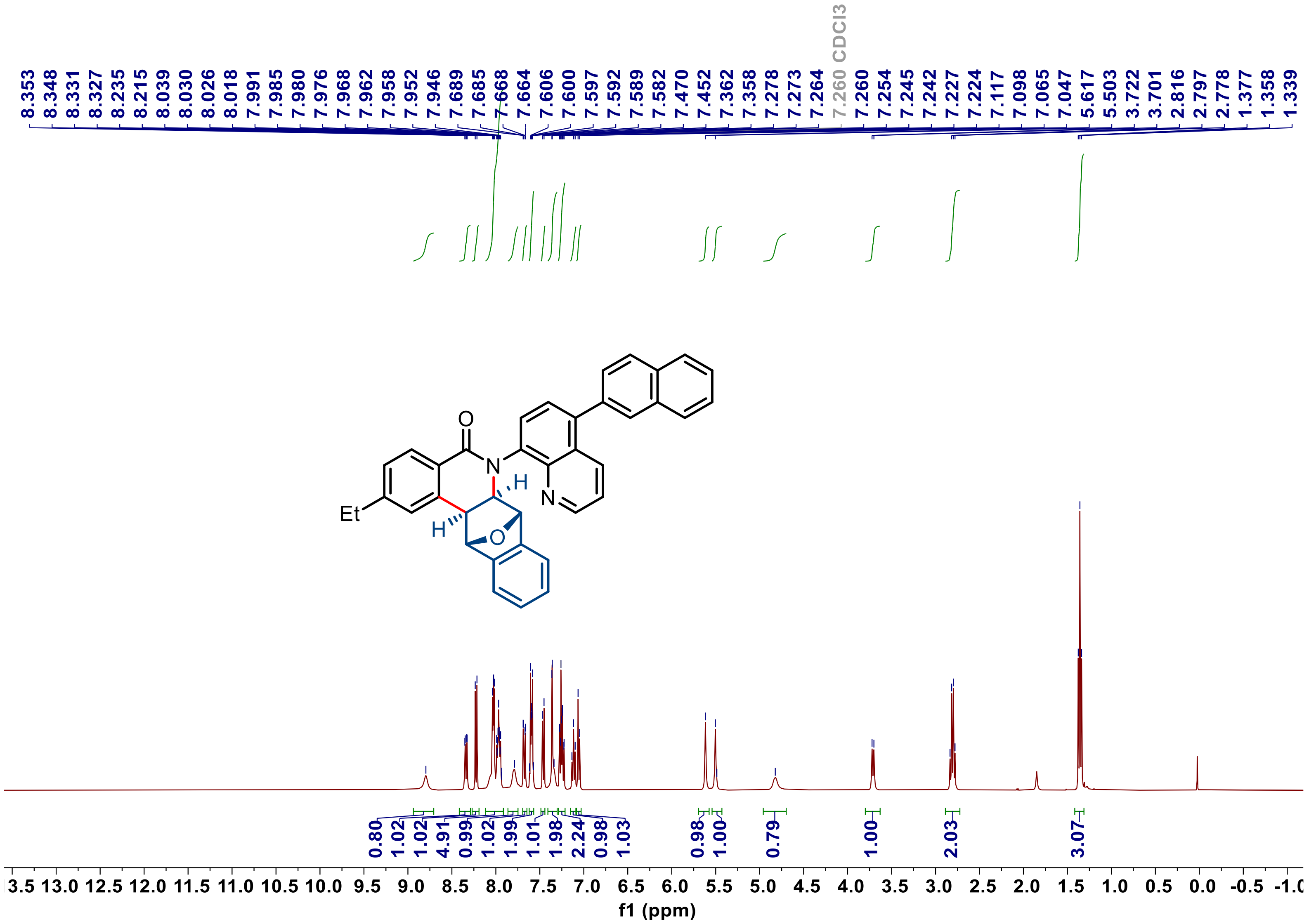
8.353
8.348
8.331
8.327
8.235
8.215
8.039
8.030
8.026
8.018
7.991
7.985
7.980
7.976
7.968
7.962
7.958
7.952
7.946
7.689
7.685
7.668
7.664
7.606
7.600
7.597
7.592
7.589
7.582
7.470
7.452
7.362
7.358
7.278
7.273
7.264
7.260 CDCl3
7.260
7.254
7.245
7.242
7.227
7.224
7.117
7.098
7.065
7.047
5.617
5.503
3.722
3.701
2.816
2.797
2.778
1.377
1.358
1.339
O
N
H
N
Et
H
O
0.80
1.02
1.02
4.91
0.99
1.02
1.99
1.01
1.98
2.24
0.98
1.03
0.98
1.00
0.79
1.00
2.03
3.07
13.5 13.0 12.5 12.0 11.5 11.0 10.5 10.0 9.5 9.0 8.5 8.0 7.5 7.0 6.5 6.0 5.5 5.0 4.5 4.0 3.5 3.0 2.5 2.0 1.5 1.0 0.5 0.0 -0.5 -1.0
f1 (ppm)

^{13}C NMR of **3g-5**

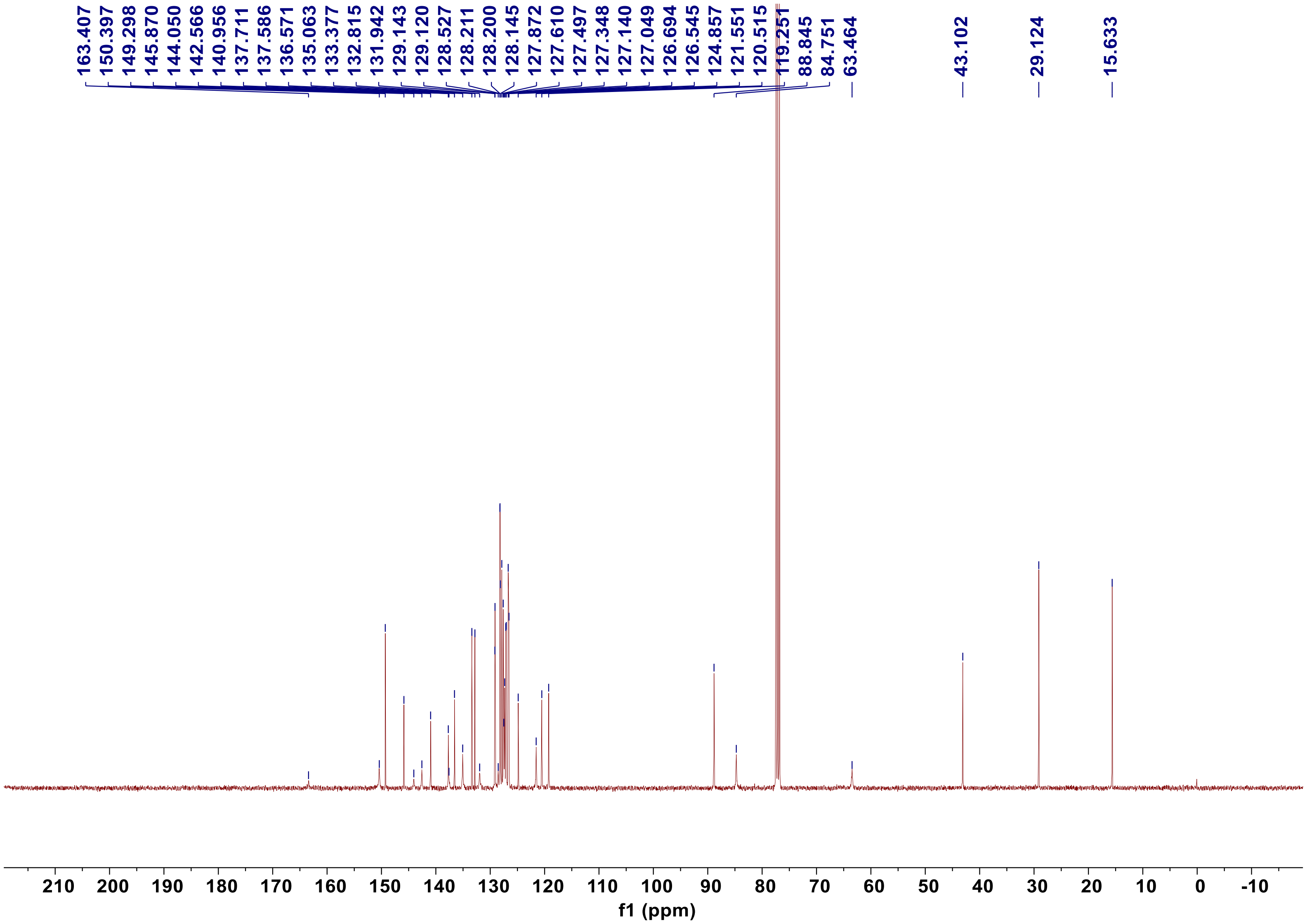

^{1}H NMR of **3g-6**

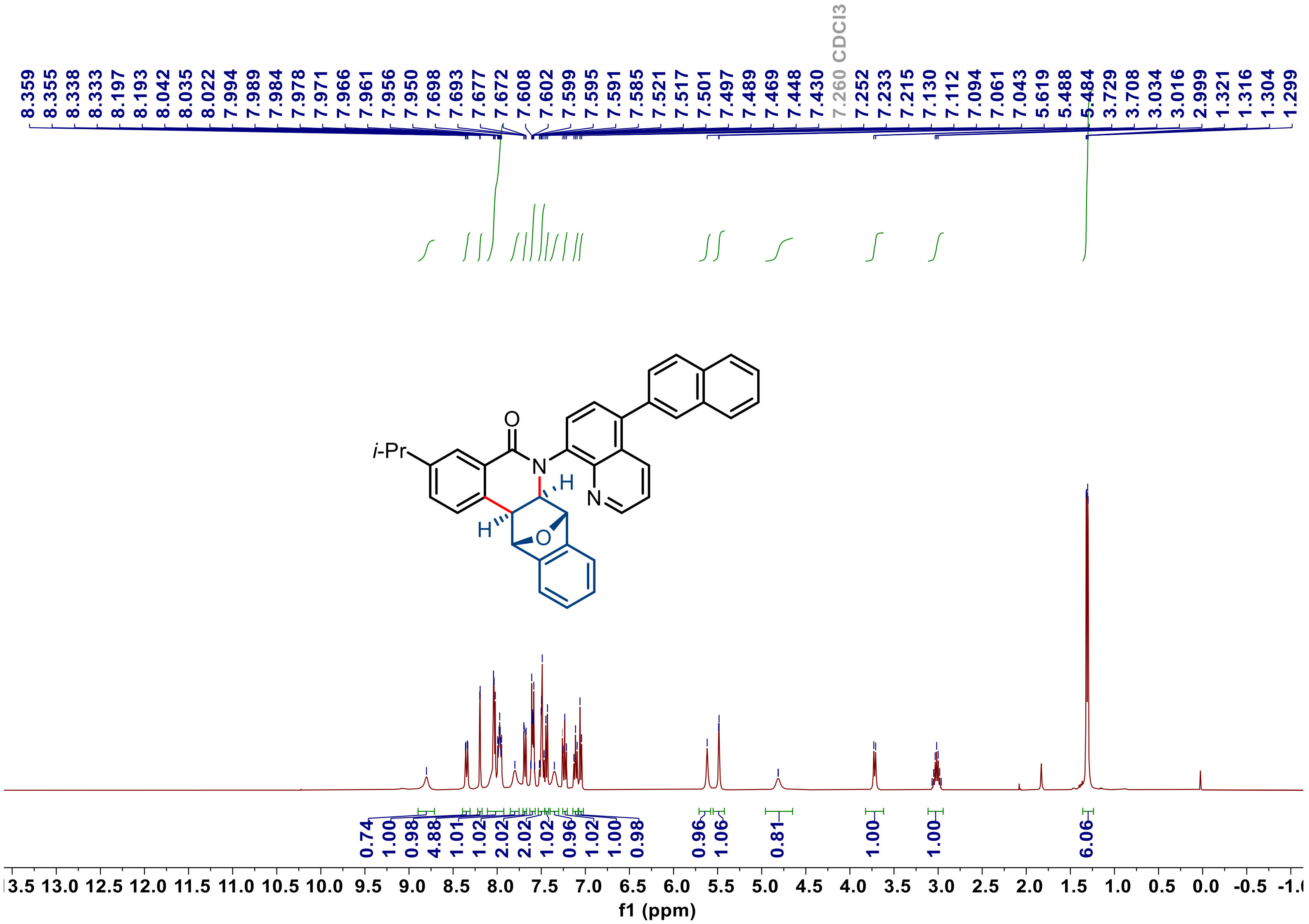
8.359 8.355 8.338 8.333 8.197 8.193 8.042 8.035 8.022 7.994 7.989 7.984 7.978 7.971 7.966 7.961 7.956 7.950 7.698 7.693 7.677 7.672 7.608 7.602 7.599 7.595 7.591 7.585 7.521 7.517 7.501 7.497 7.489 7.469 7.448 7.430 7.260 CDCl3 7.252 7.233 7.215 7.130 7.112 7.094 7.061 7.043 5.619 5.488 5.484 3.729 3.708 3.034 3.016 2.999 1.321 1.316 1.304 1.299
i-Pr
O
N
H
N
O
0.74 1.00 0.98 4.88 1.01 1.02 2.02 2.02 1.02 0.96 1.02 1.00 0.98 0.96 1.06 0.81 1.00 1.00 6.06
13.5 13.0 12.5 12.0 11.5 11.0 10.5 10.0 9.5 9.0 8.5 8.0 7.5 7.0 6.5 6.0 5.5 5.0 4.5 4.0 3.5 3.0 2.5 2.0 1.5 1.0 0.5 0.0 -0.5 -1.0
f1 (ppm)

^{13}C NMR of **3g-6**

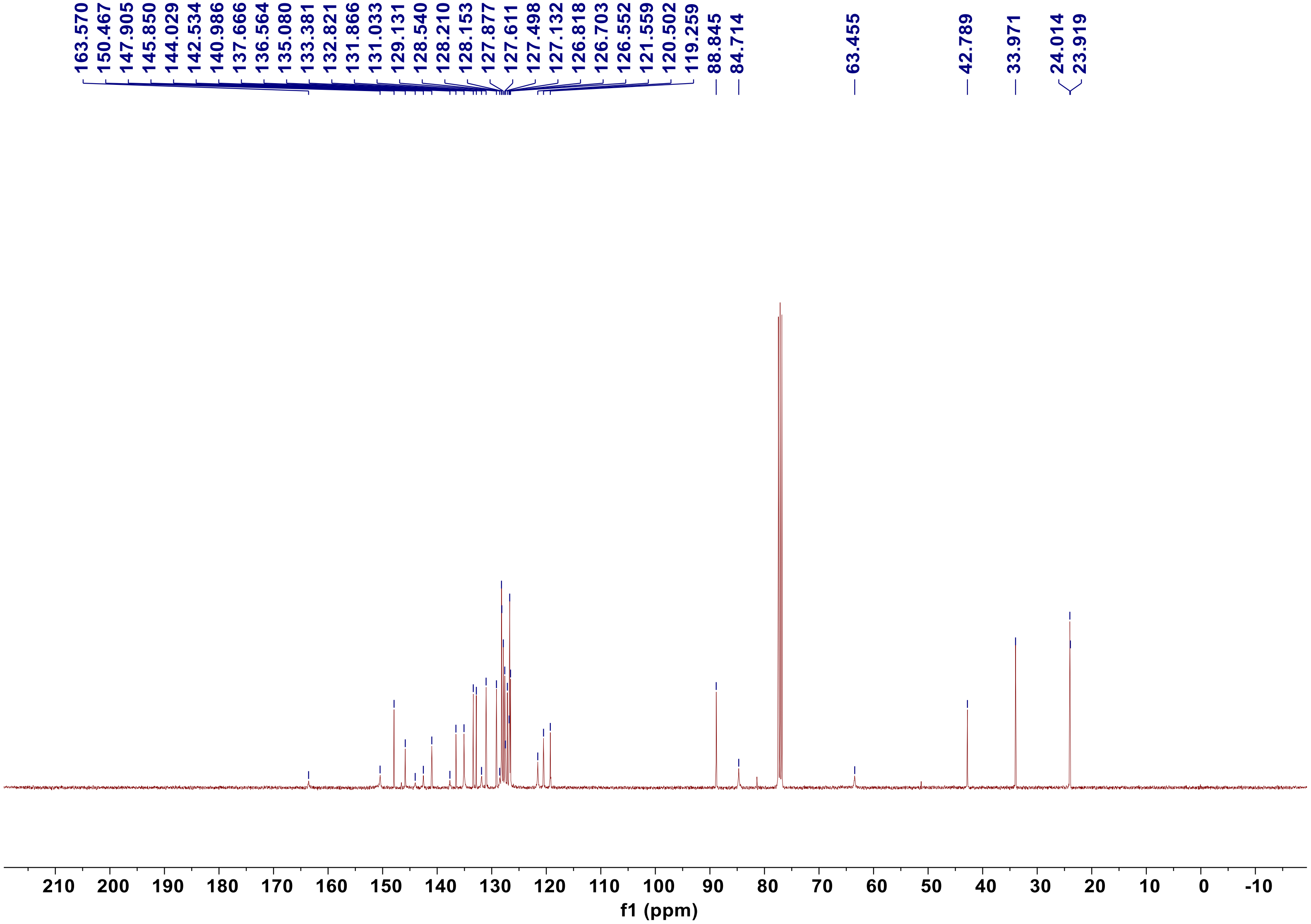

^{1}H NMR of **3g-7**

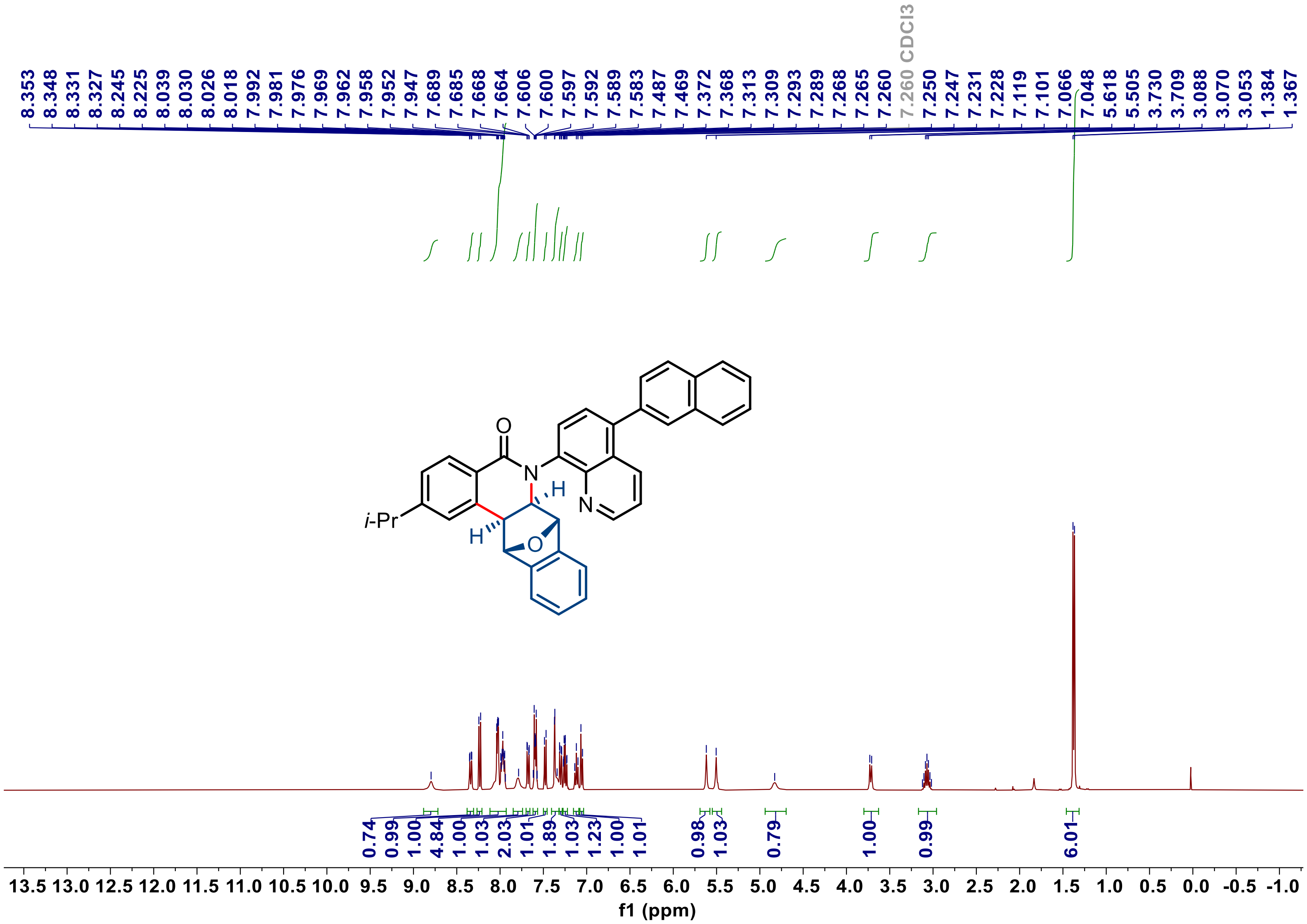
8.353 8.348 8.331 8.327 8.245 8.225 8.039 8.030 8.026 8.018 7.992 7.981 7.976 7.969 7.962 7.958 7.952 7.947 7.689 7.685 7.668 7.664 7.606 7.600 7.597 7.592 7.589 7.583 7.487 7.469 7.372 7.368 7.313 7.309 7.293 7.289 7.268 7.265 7.260 7.260 CDCl3 7.250 7.247 7.231 7.228 7.119 7.101 7.066 7.048 5.618 5.505 3.730 3.709 3.088 3.070 3.053 1.384 1.367
0.74 0.99 1.00 4.84 1.00 1.03 2.03 1.01 1.89 1.03 1.23 1.00 1.01 0.98 1.03 0.79 1.00 0.99 6.01
13.5 13.0 12.5 12.0 11.5 11.0 10.5 10.0 9.5 9.0 8.5 8.0 7.5 7.0 6.5 6.0 5.5 5.0 4.5 4.0 3.5 3.0 2.5 2.0 1.5 1.0 0.5 0.0 -0.5 -1.0
f1 (ppm)
i-Pr
O
N
H
N
H
O

^{13}C NMR of **3g-7**

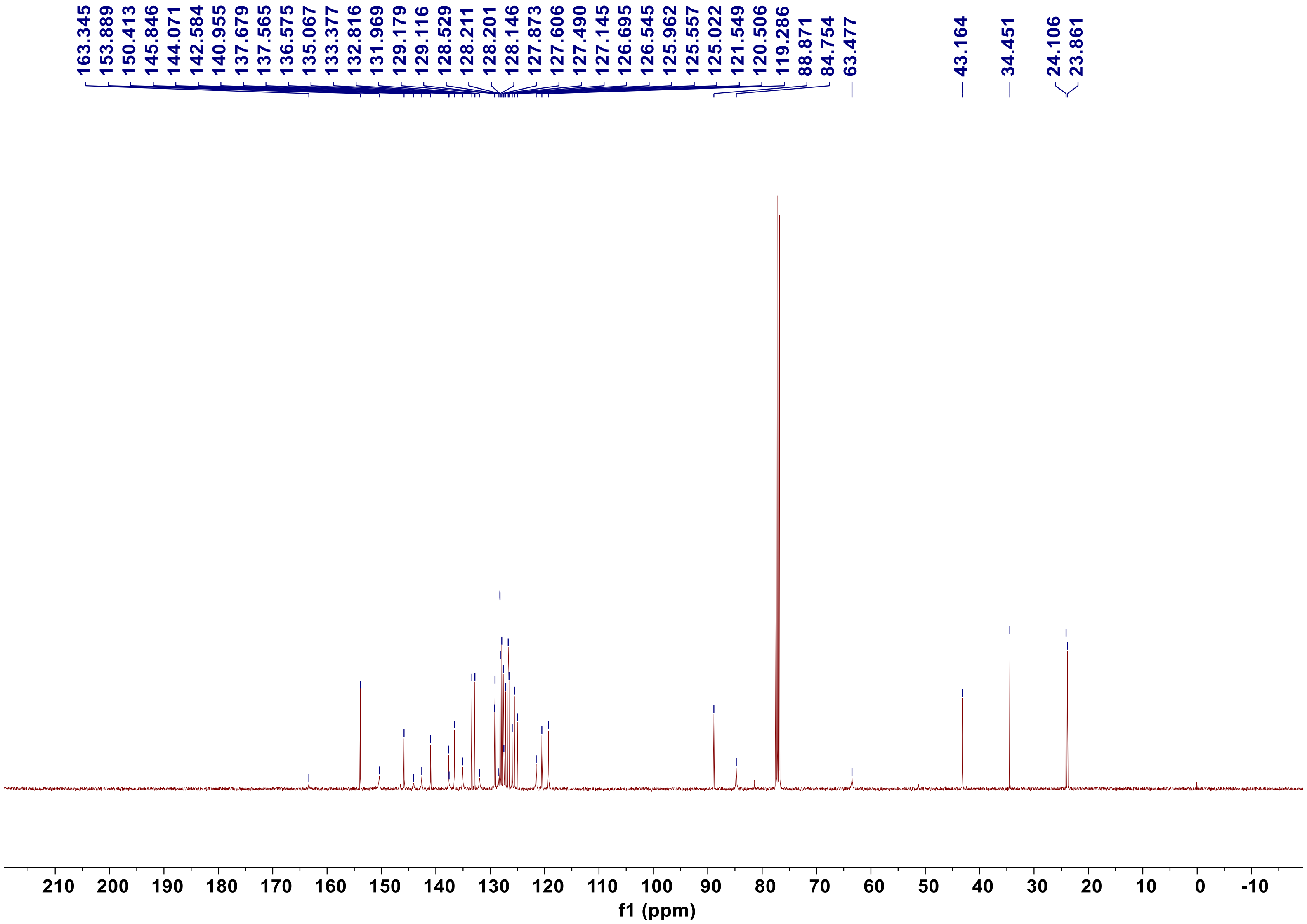

^{1}H NMR of **3g-8**

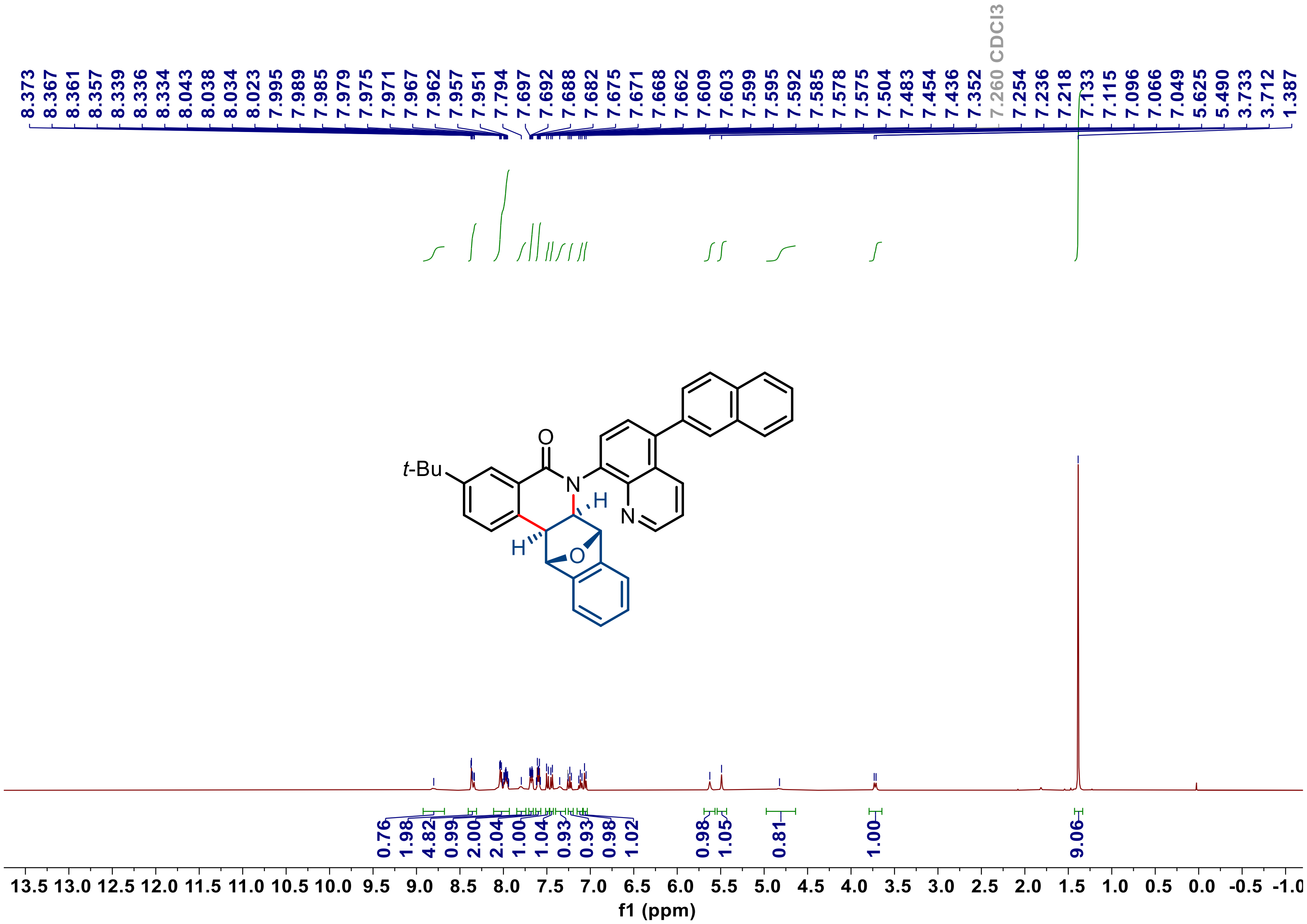

t-Bu
O
N
H
H
O
N
8.373 8.367 8.361 8.357 8.339 8.336 8.334 8.043 8.038 8.034 8.023 7.995 7.989 7.985 7.979 7.975 7.971 7.967 7.962 7.957 7.951 7.794 7.697 7.692 7.688 7.682 7.675 7.671 7.668 7.662 7.609 7.603 7.599 7.595 7.592 7.585 7.578 7.575 7.504 7.483 7.454 7.436 7.352 7.260 CDCl3 7.254 7.236 7.218 7.133 7.115 7.096 7.066 7.049 5.625 5.490 3.733 3.712 1.387
0.76 1.98 4.82 0.99 2.00 2.04 1.00 1.04 0.93 0.93 0.98 1.02 0.98 1.05 0.81 1.00 9.06
13.5 13.0 12.5 12.0 11.5 11.0 10.5 10.0 9.5 9.0 8.5 8.0 7.5 7.0 6.5 6.0 5.5 5.0 4.5 4.0 3.5 3.0 2.5 2.0 1.5 1.0 0.5 0.0 -0.5 -1.0
f1 (ppm)

^{13}C NMR of **3g-8**

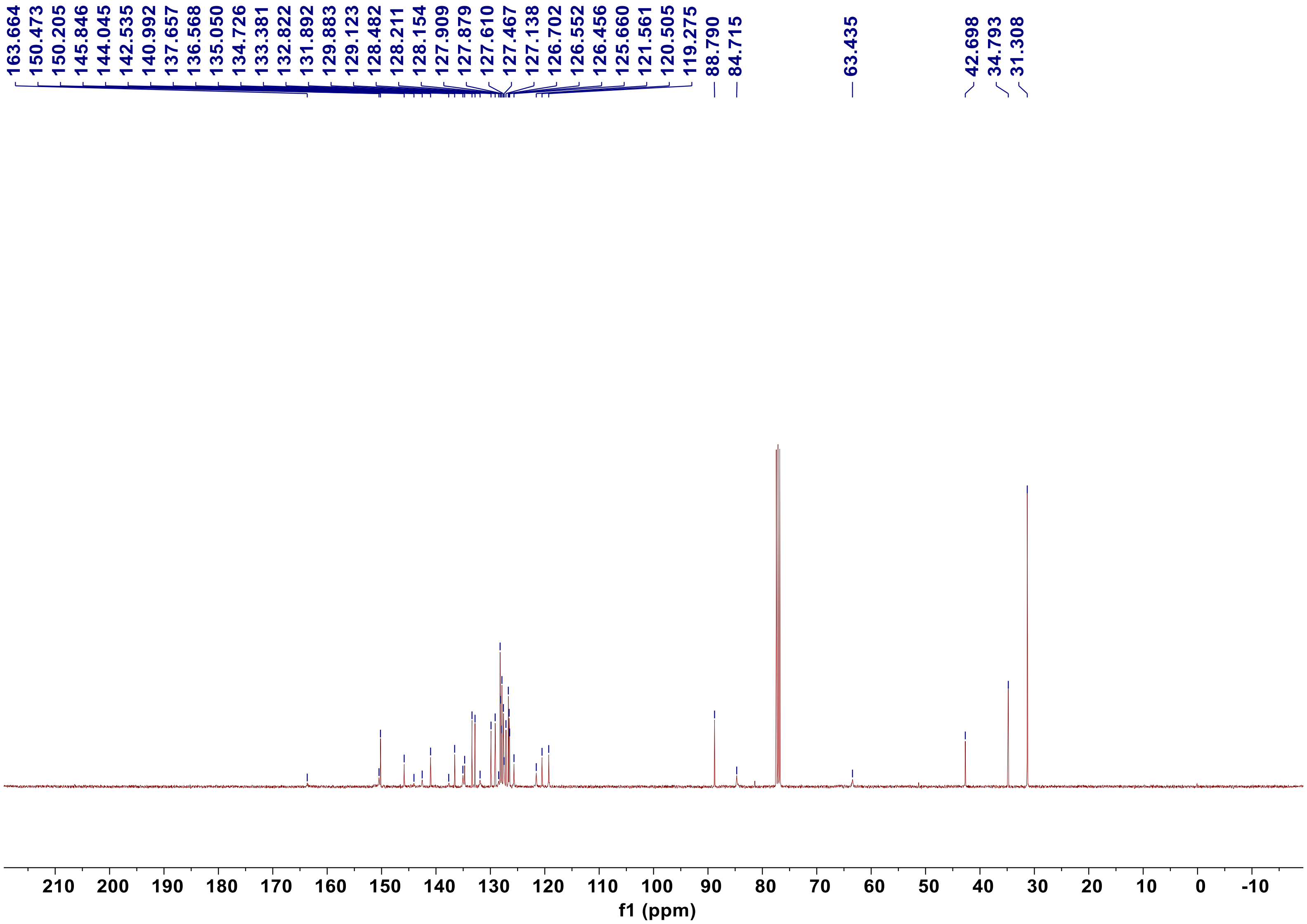

^{1}H NMR of **3g-9**

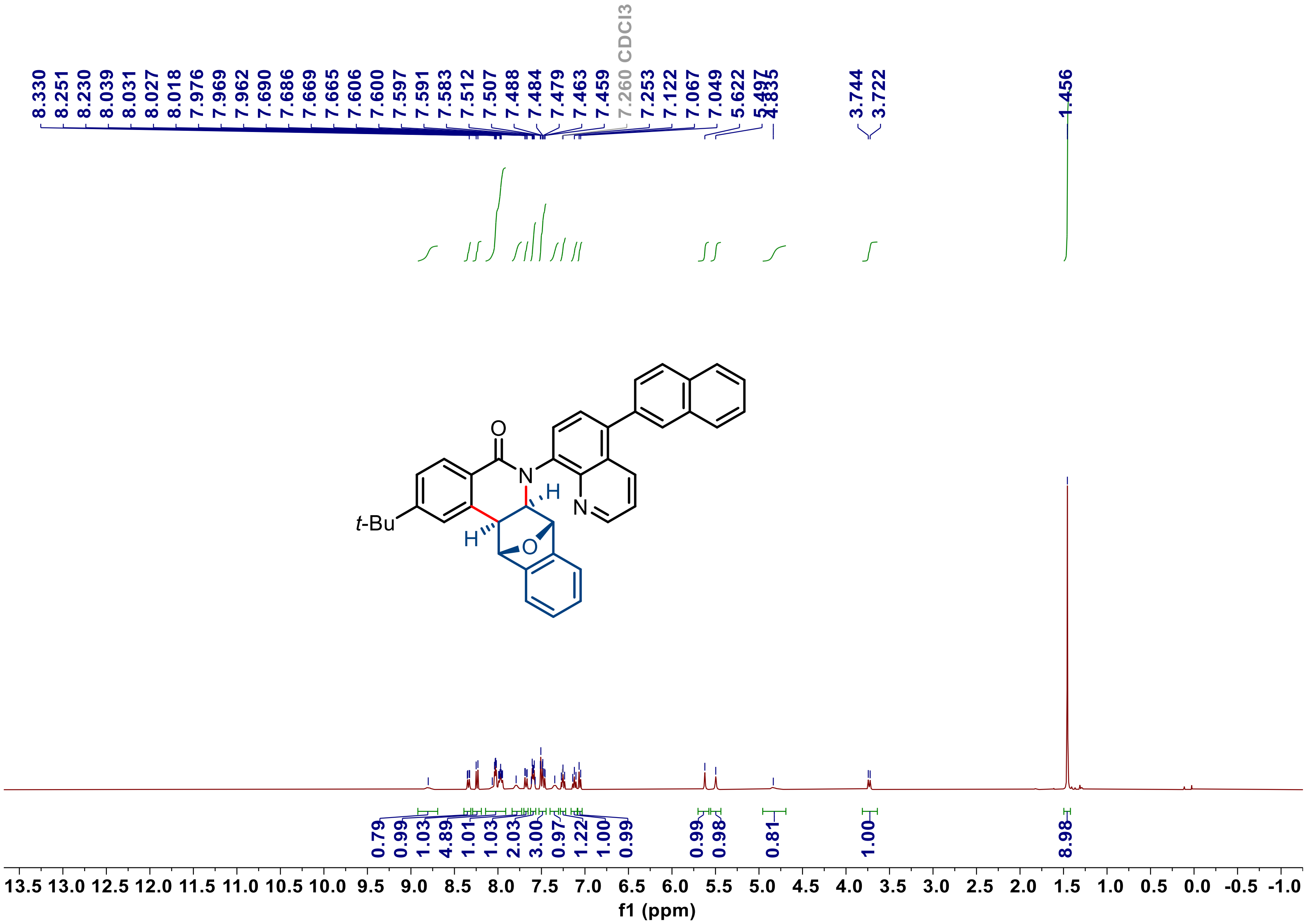
8.330
8.251
8.230
8.039
8.031
8.027
8.018
7.976
7.969
7.962
7.690
7.686
7.669
7.665
7.606
7.600
7.597
7.591
7.583
7.512
7.507
7.488
7.484
7.479
7.463
7.459
7.260 CDCl3
7.253
7.122
7.067
7.049
5.622
5.497
4.835
3.744
3.722
1.456
O
N
H
t-Bu
H
O
N
0.79
0.99
1.03
4.89
1.01
1.03
2.03
3.00
0.97
1.22
1.00
0.99
0.99
0.98
0.81
1.00
8.98
13.5 13.0 12.5 12.0 11.5 11.0 10.5 10.0 9.5 9.0 8.5 8.0 7.5 7.0 6.5 6.0 5.5 5.0 4.5 4.0 3.5 3.0 2.5 2.0 1.5 1.0 0.5 0.0 -0.5 -1.0
f1 (ppm)

^{13}C NMR of **3g-9**

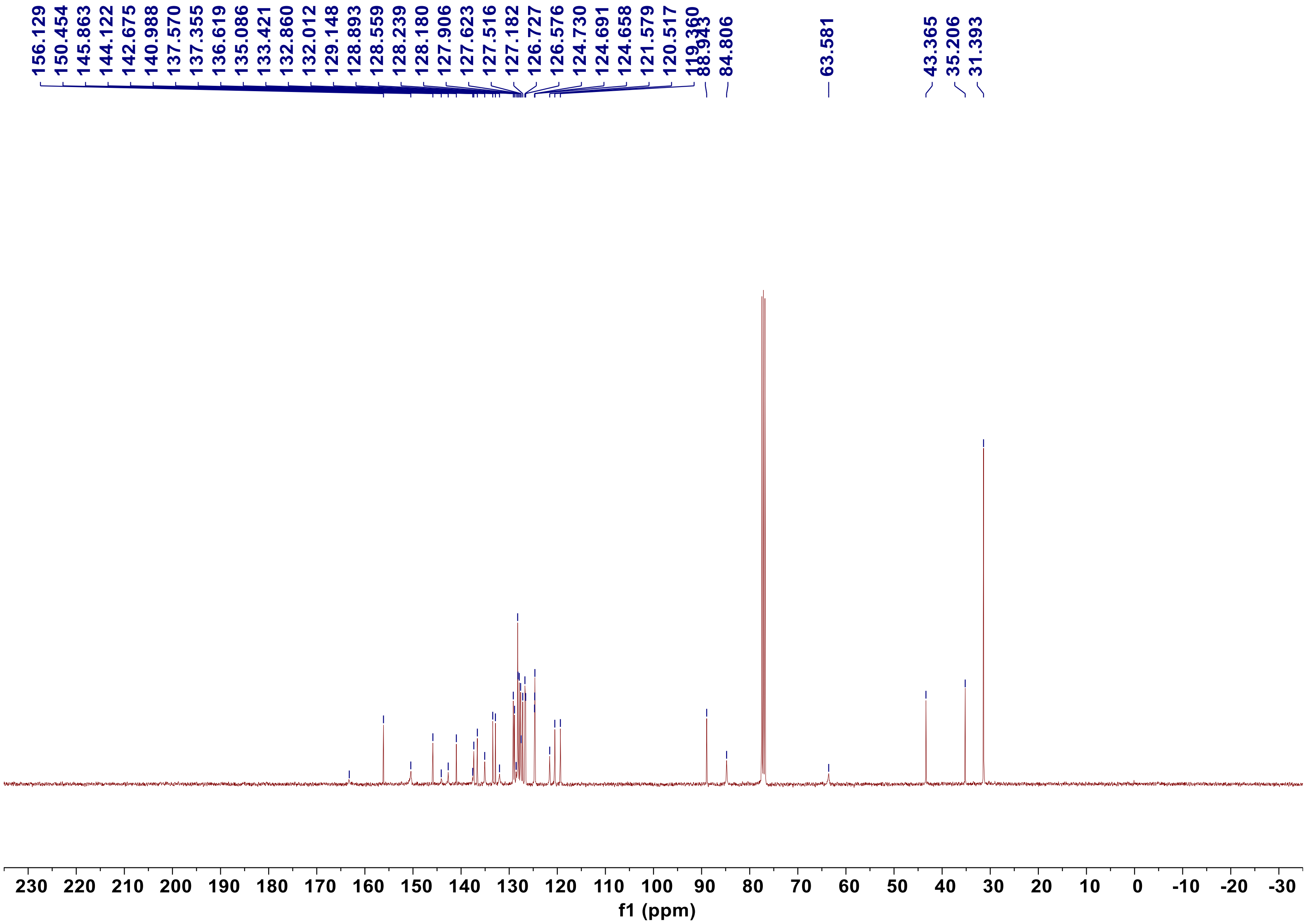

^{1}H NMR of **3g-10**

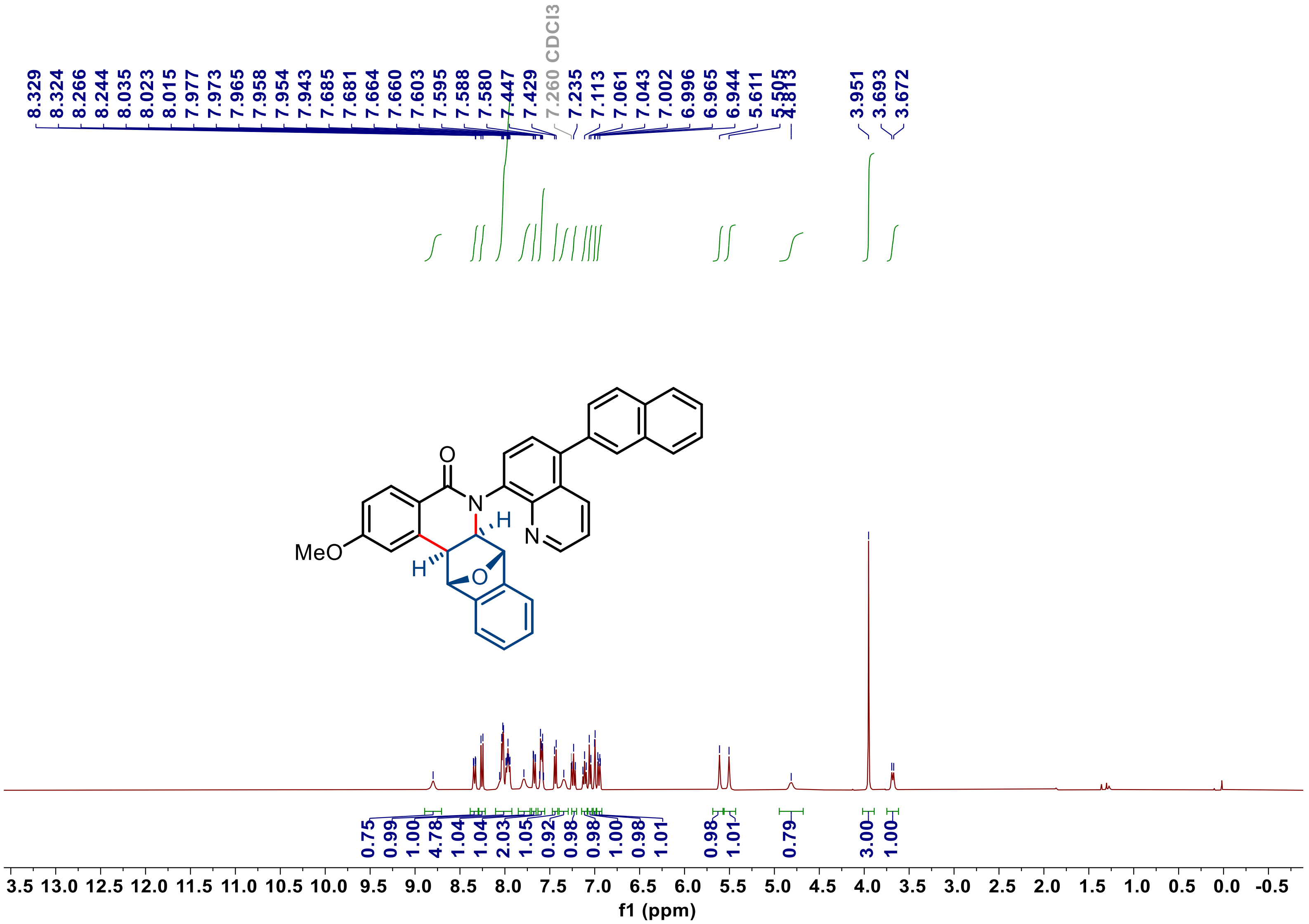

^{13}C NMR of **3g-10**

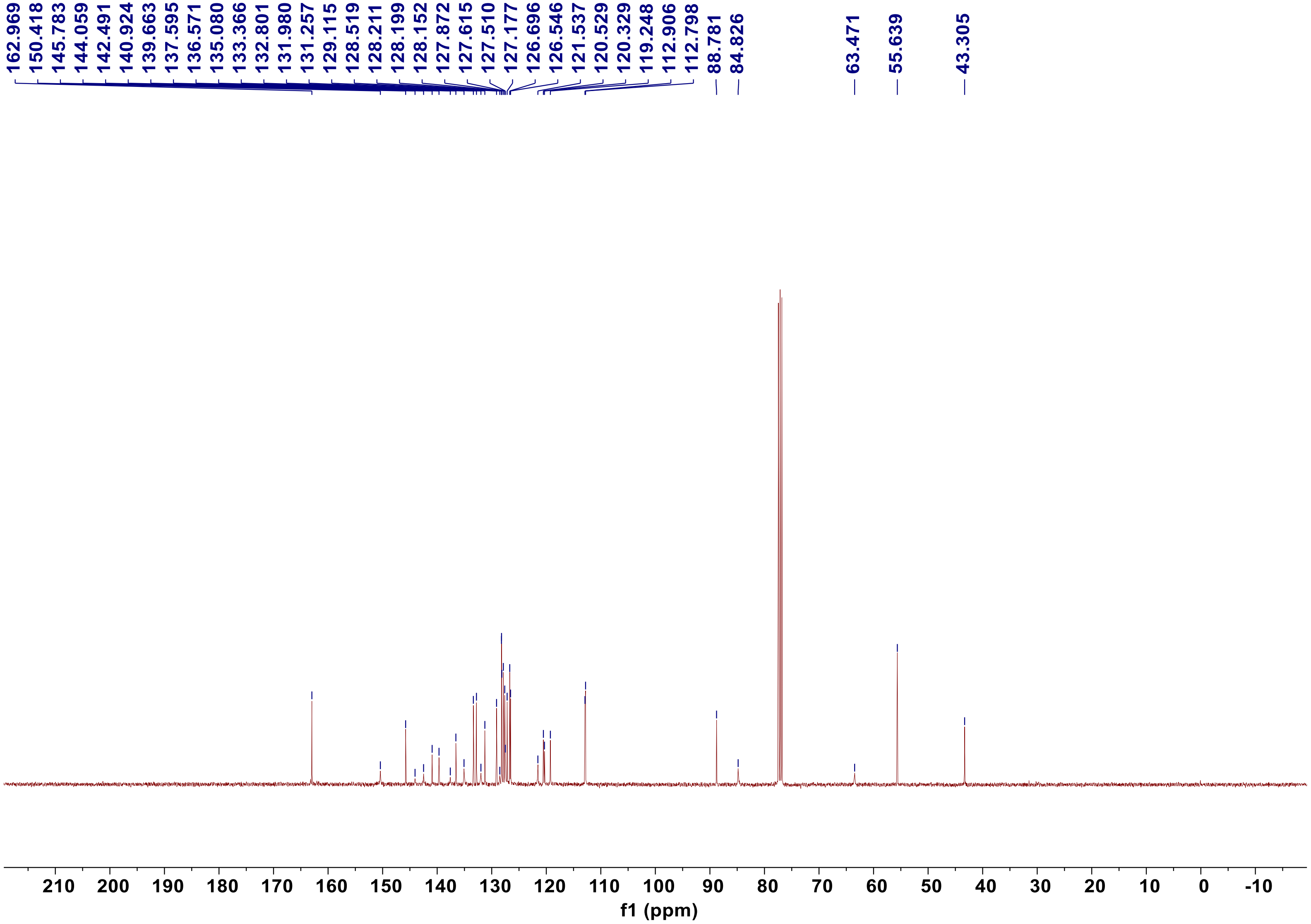

^{1}H NMR of **3g-11**

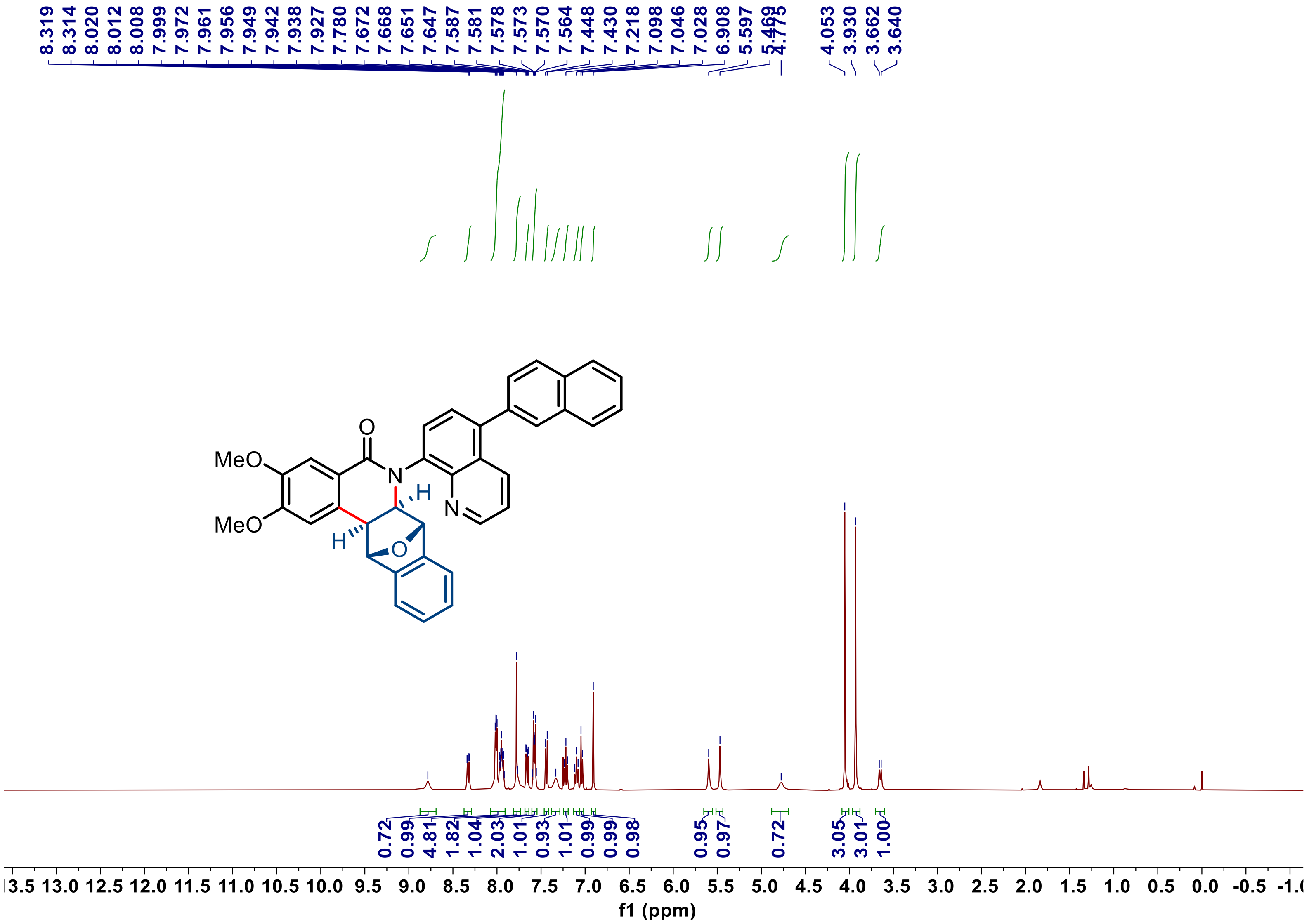

^{13}C NMR of **3g-11**

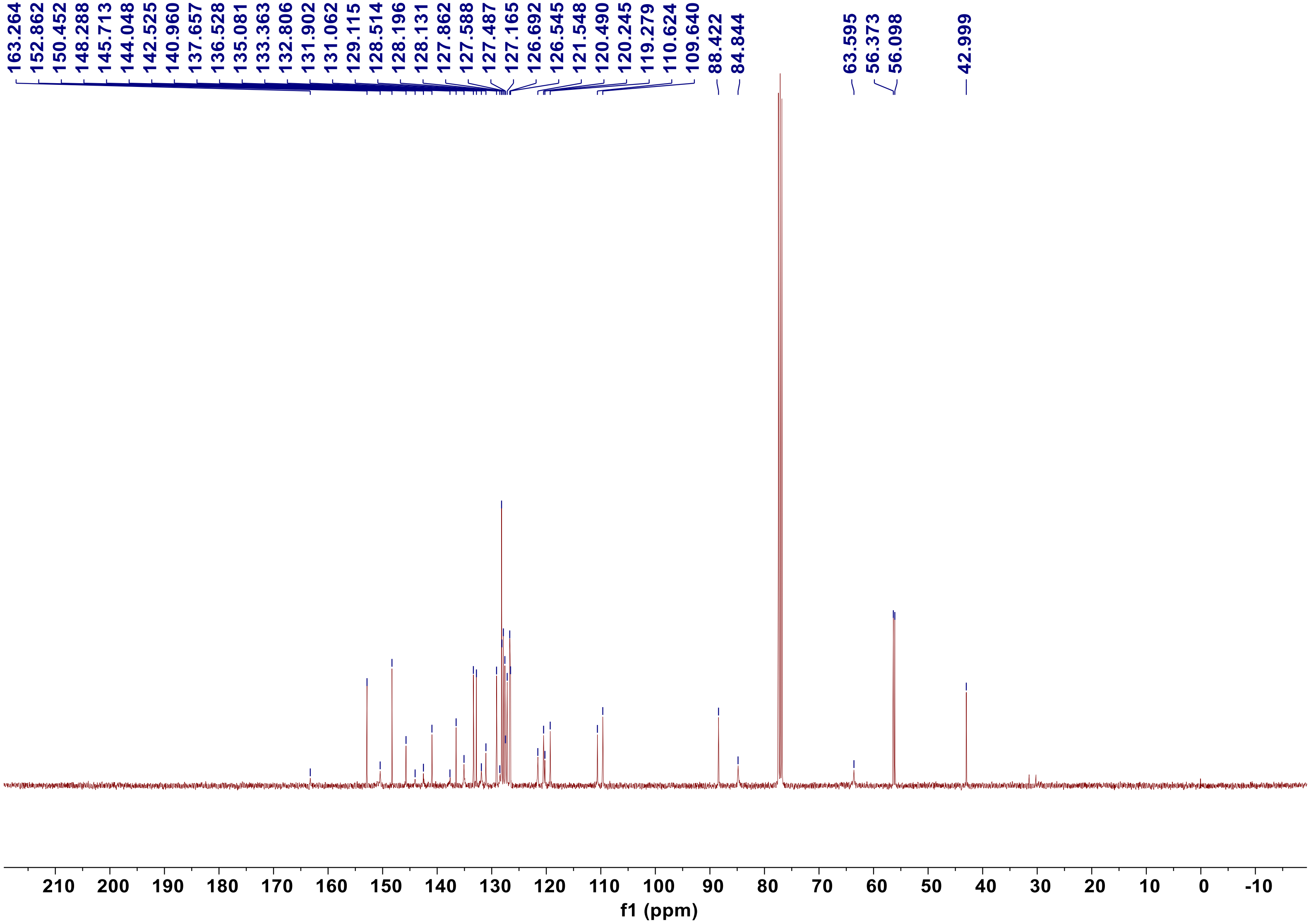

^{1}H NMR of **3g-12**

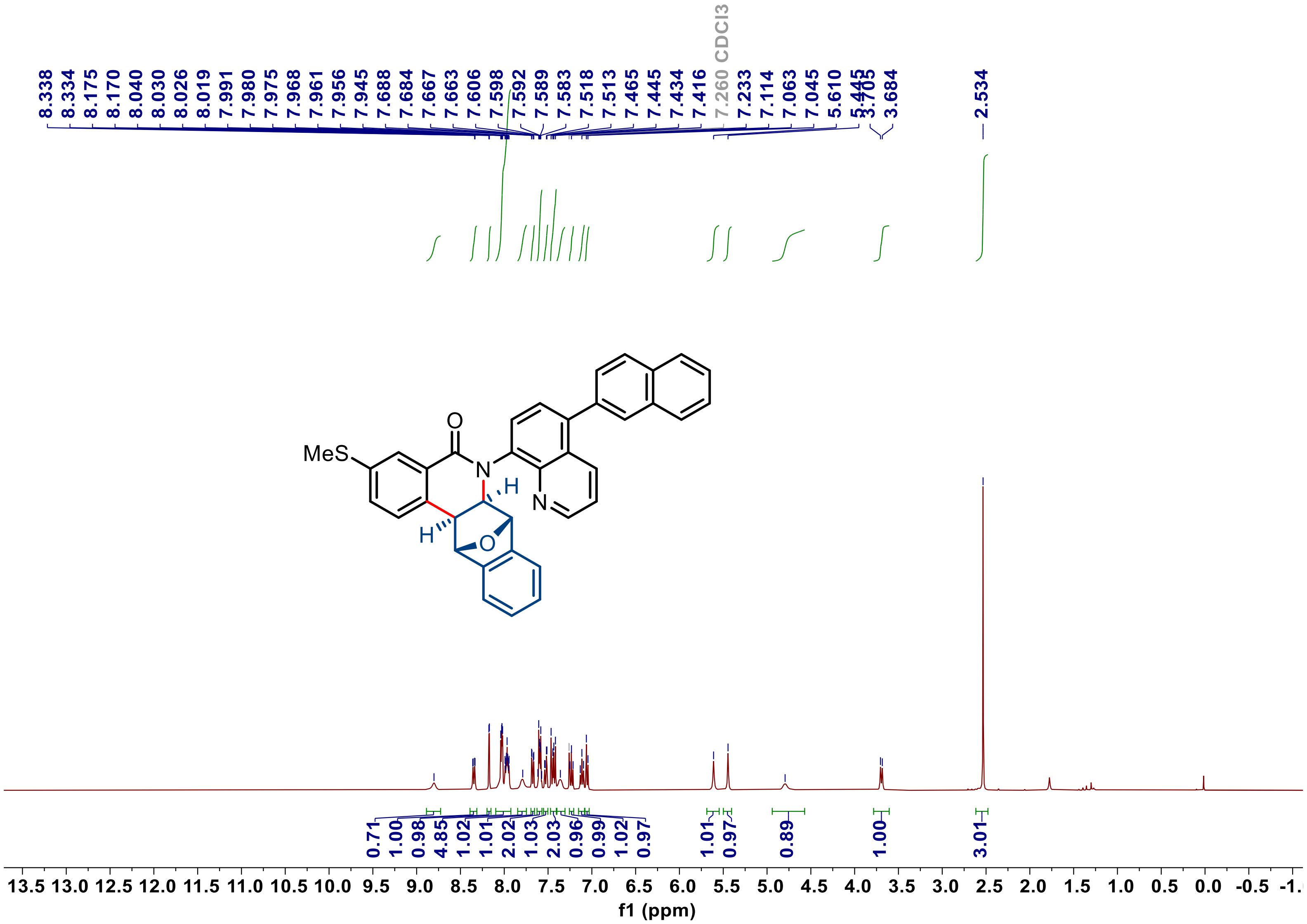

^{13}C NMR of **3g-12**

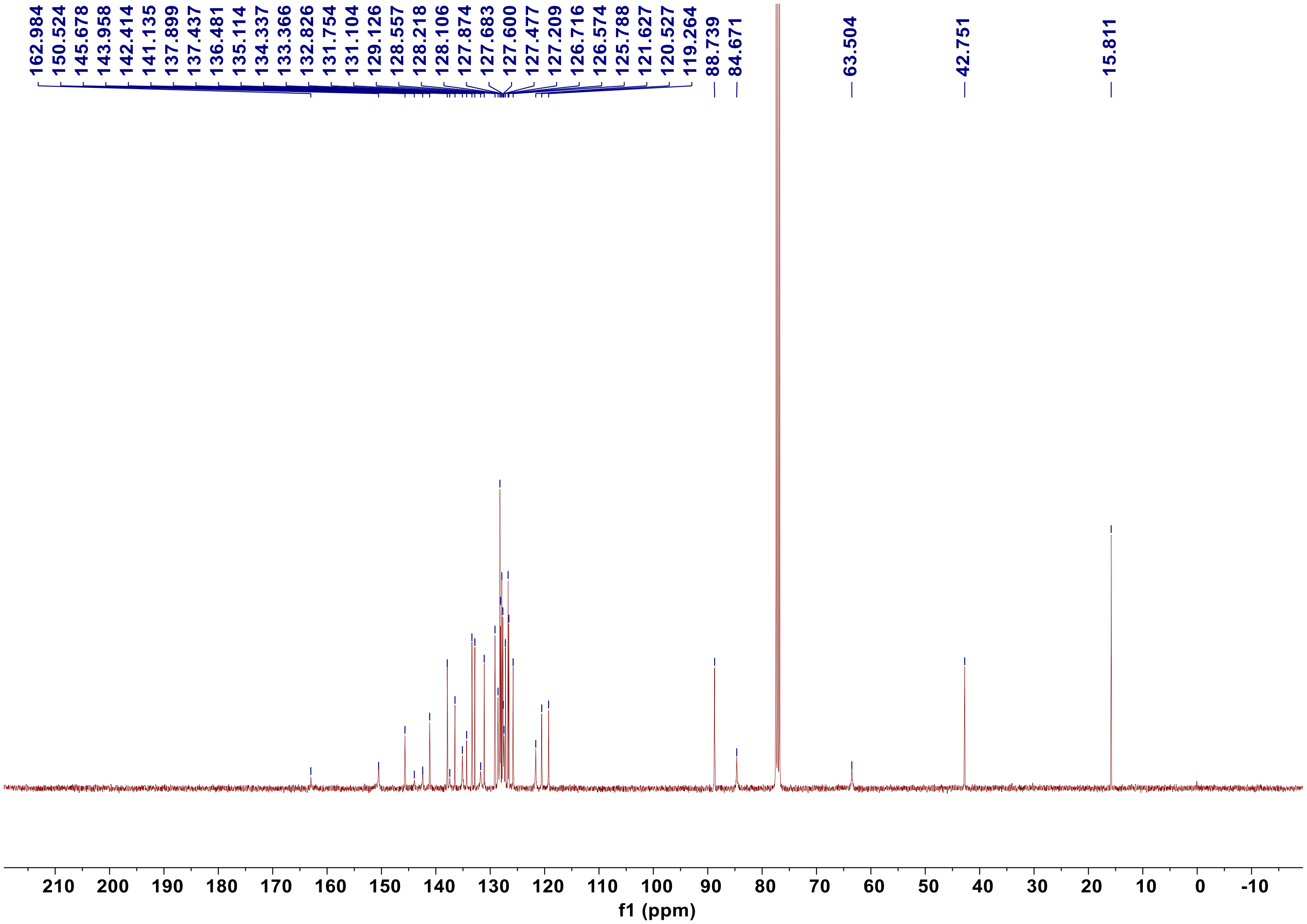

^{1}H NMR of **3g-13**

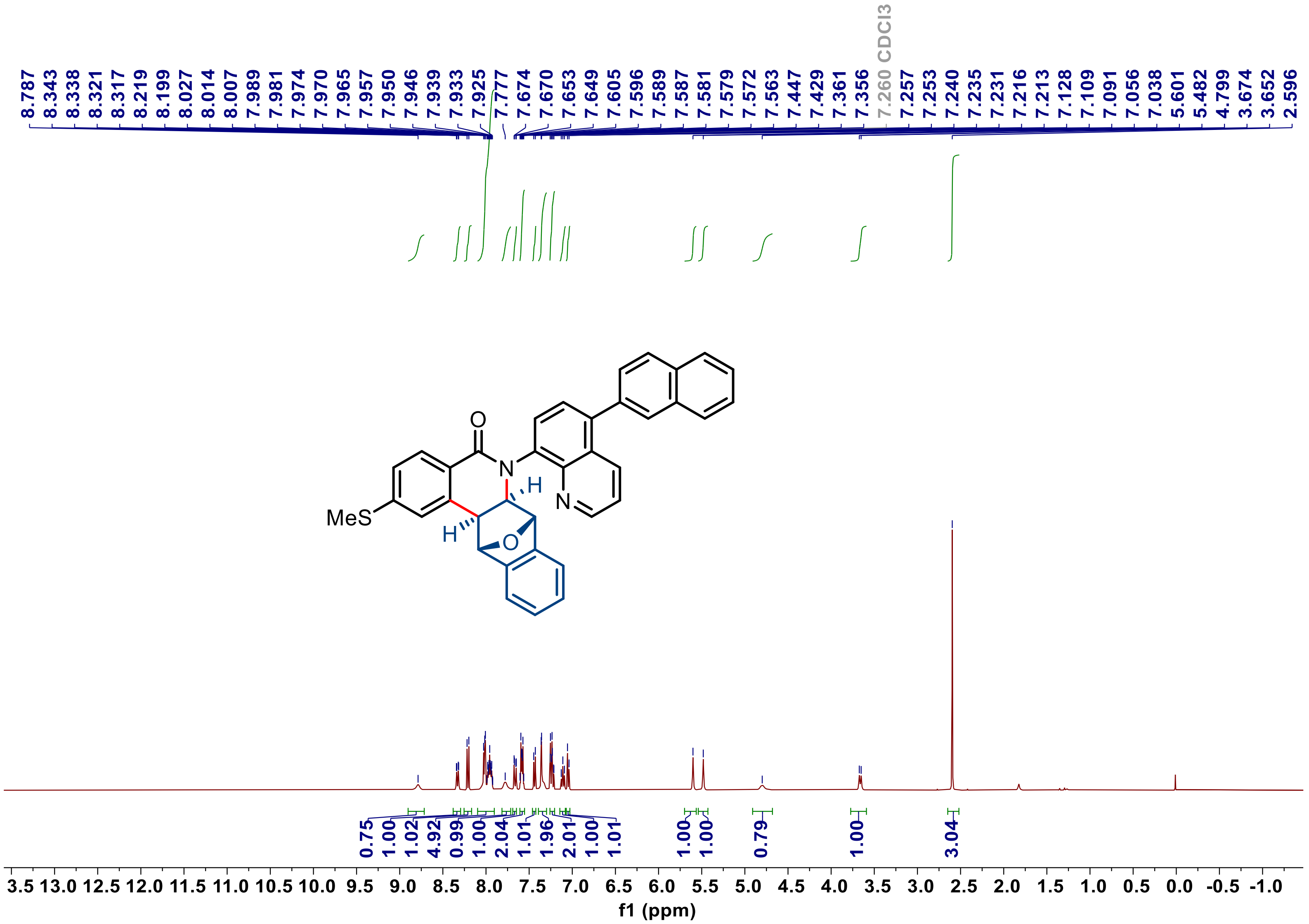

^{13}C NMR of **3g-13**

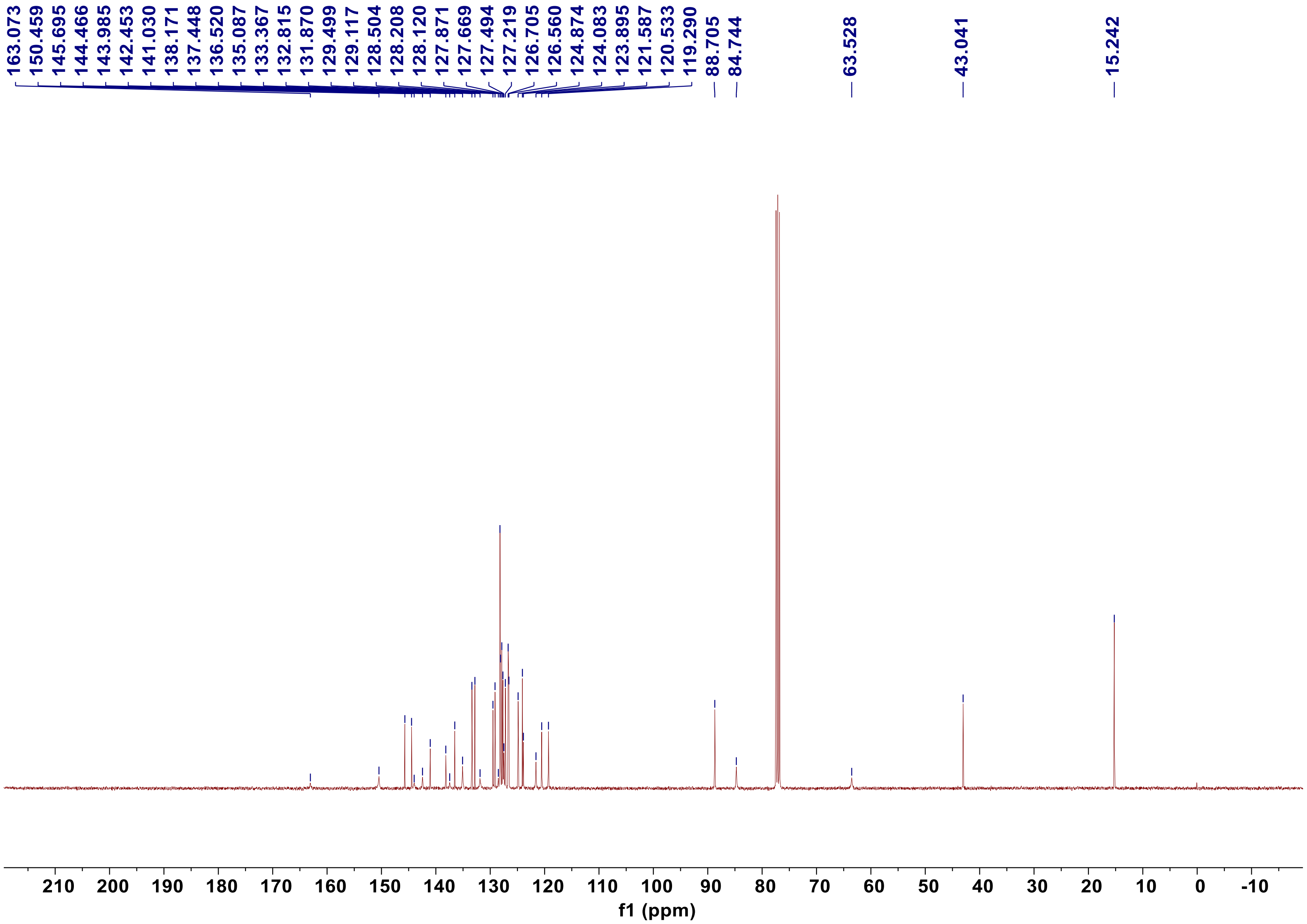

^{1}H NMR of **3g-14**

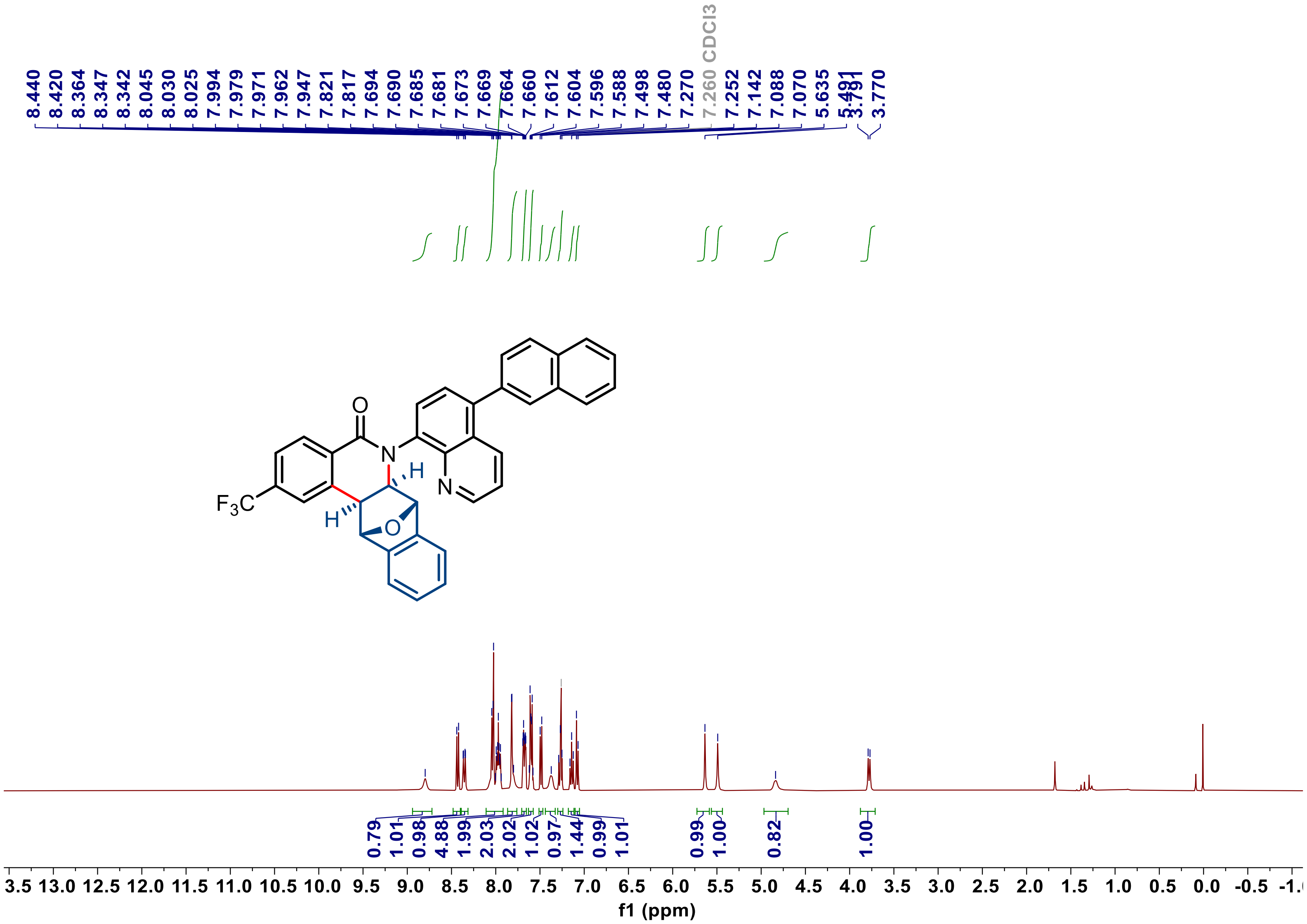

^{19}F NMR of **3g-14**

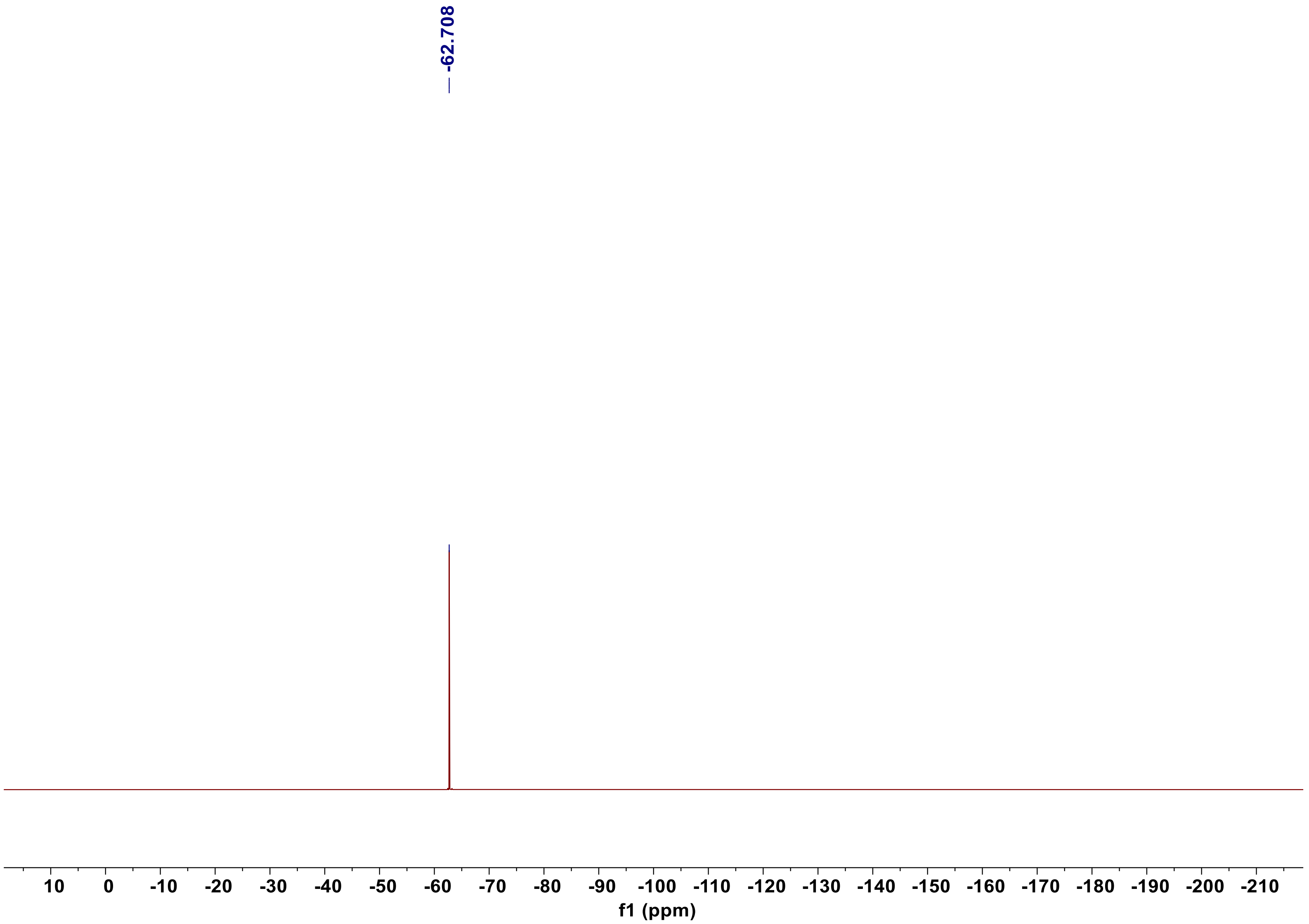

^{13}C NMR of **3g-14**

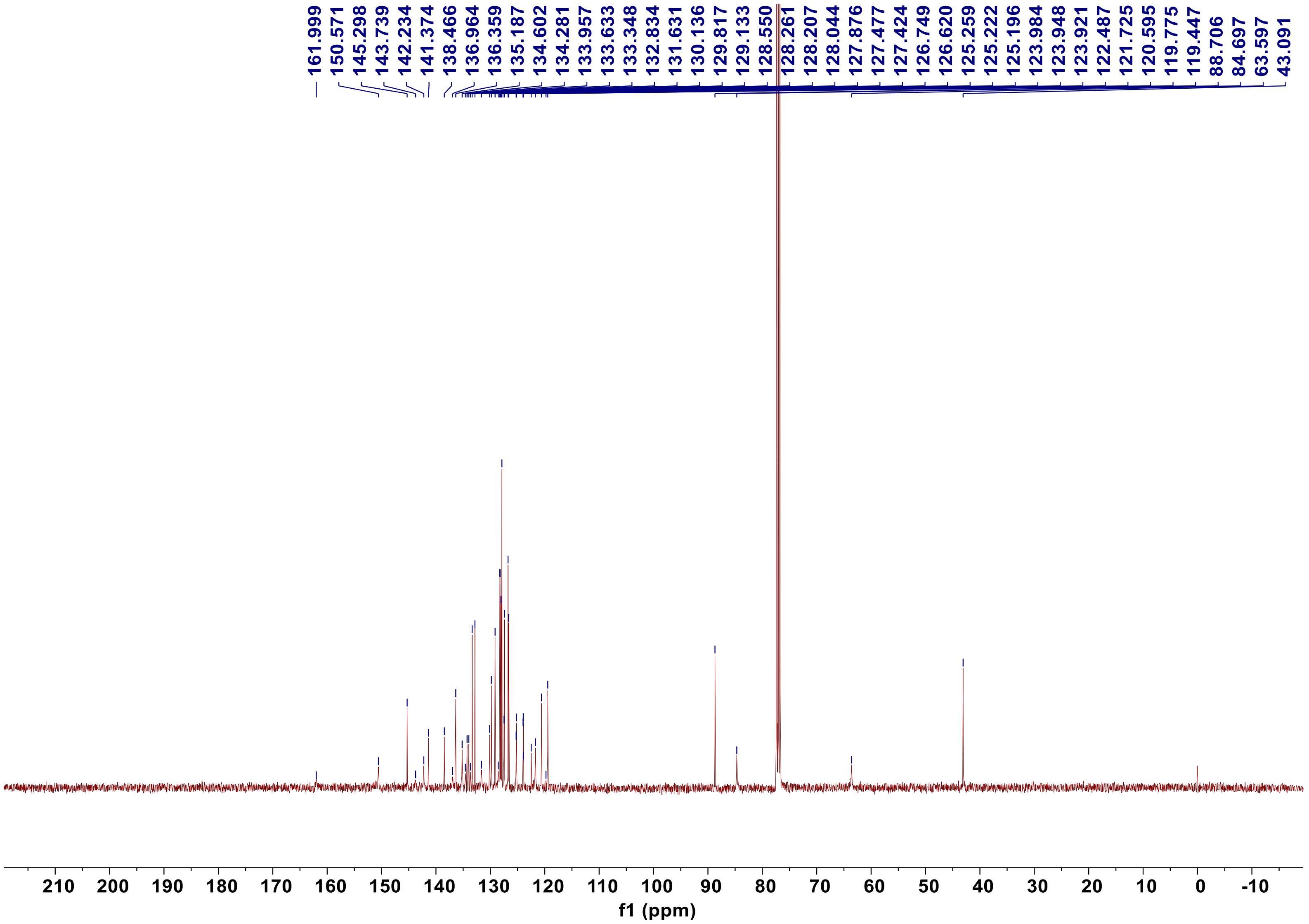

^{1}H NMR of **3g-15**

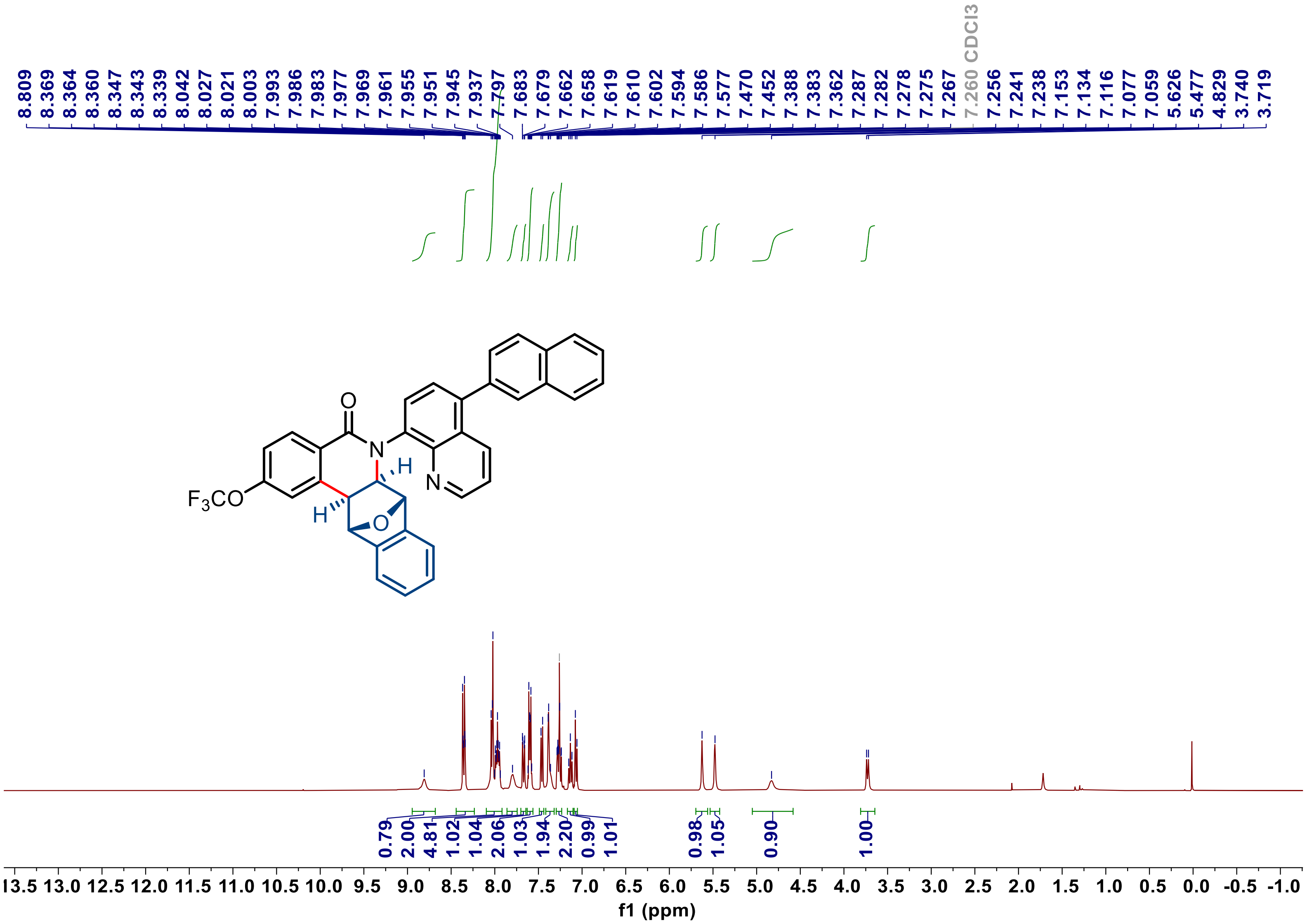

^{19}F NMR of **3g-15**

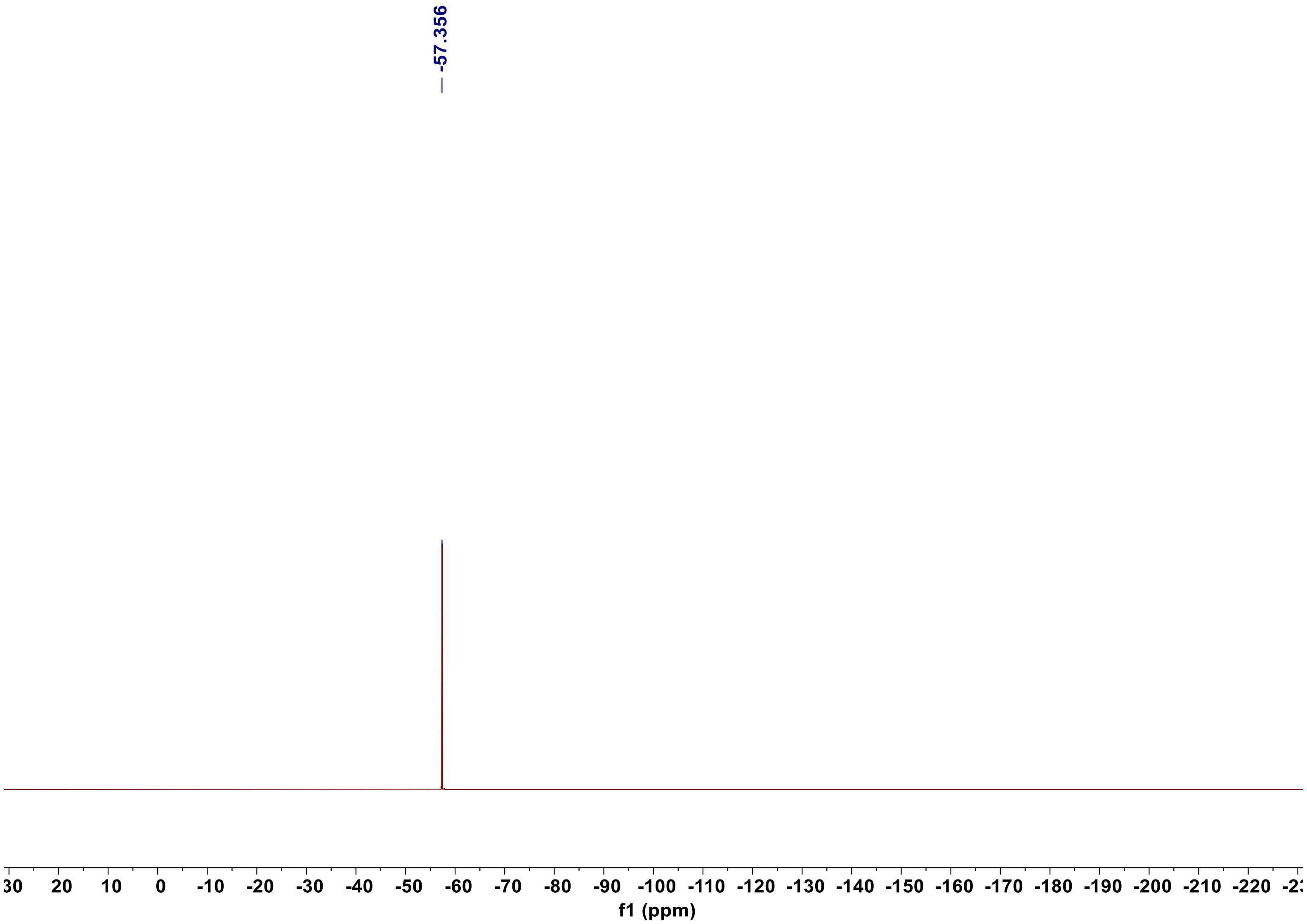

^{13}C NMR of **3g-15**

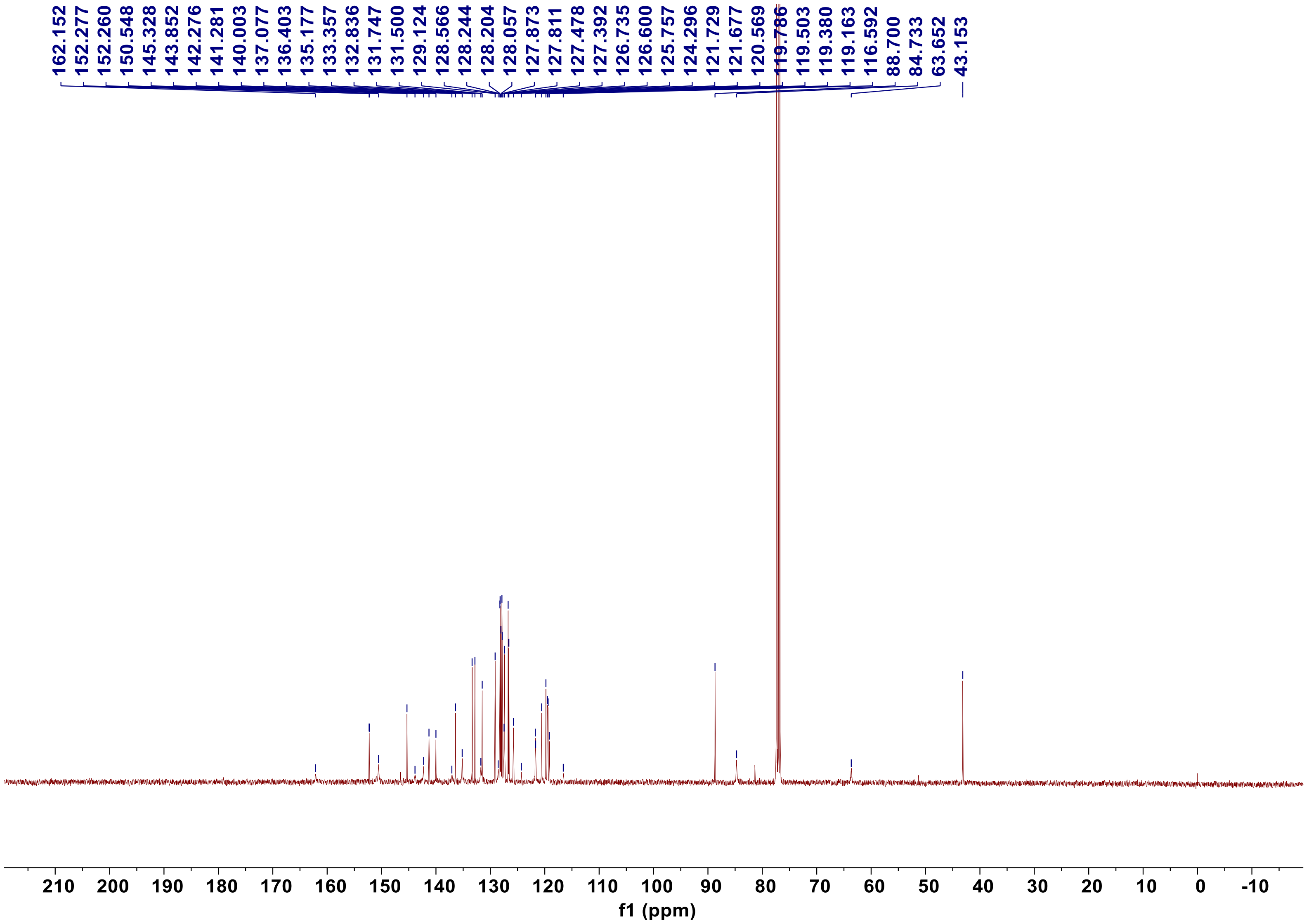

^{1}H NMR of **3g-16**

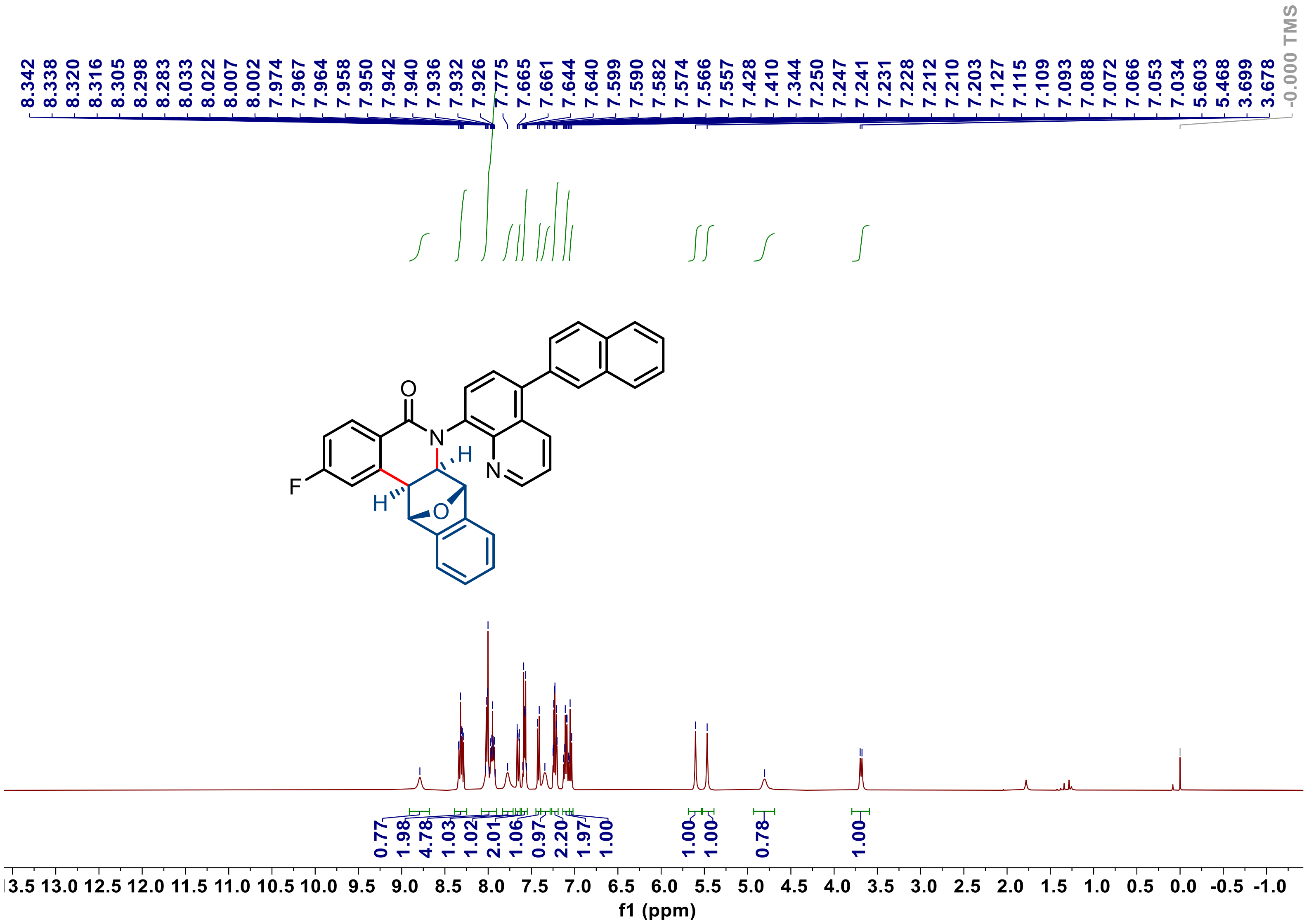

^{19}F NMR of **3g-16**

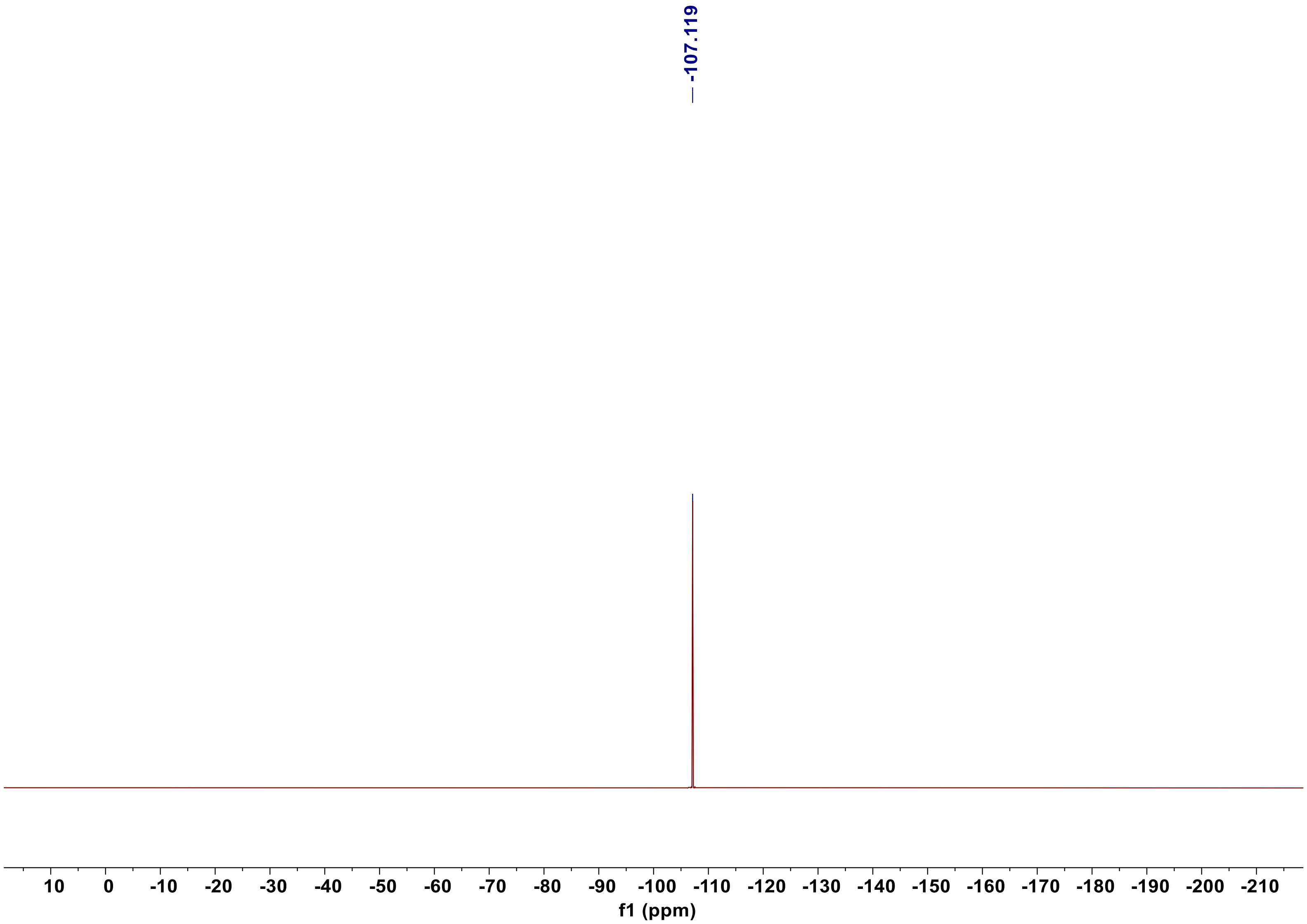

^{13}C NMR of **3g-16**

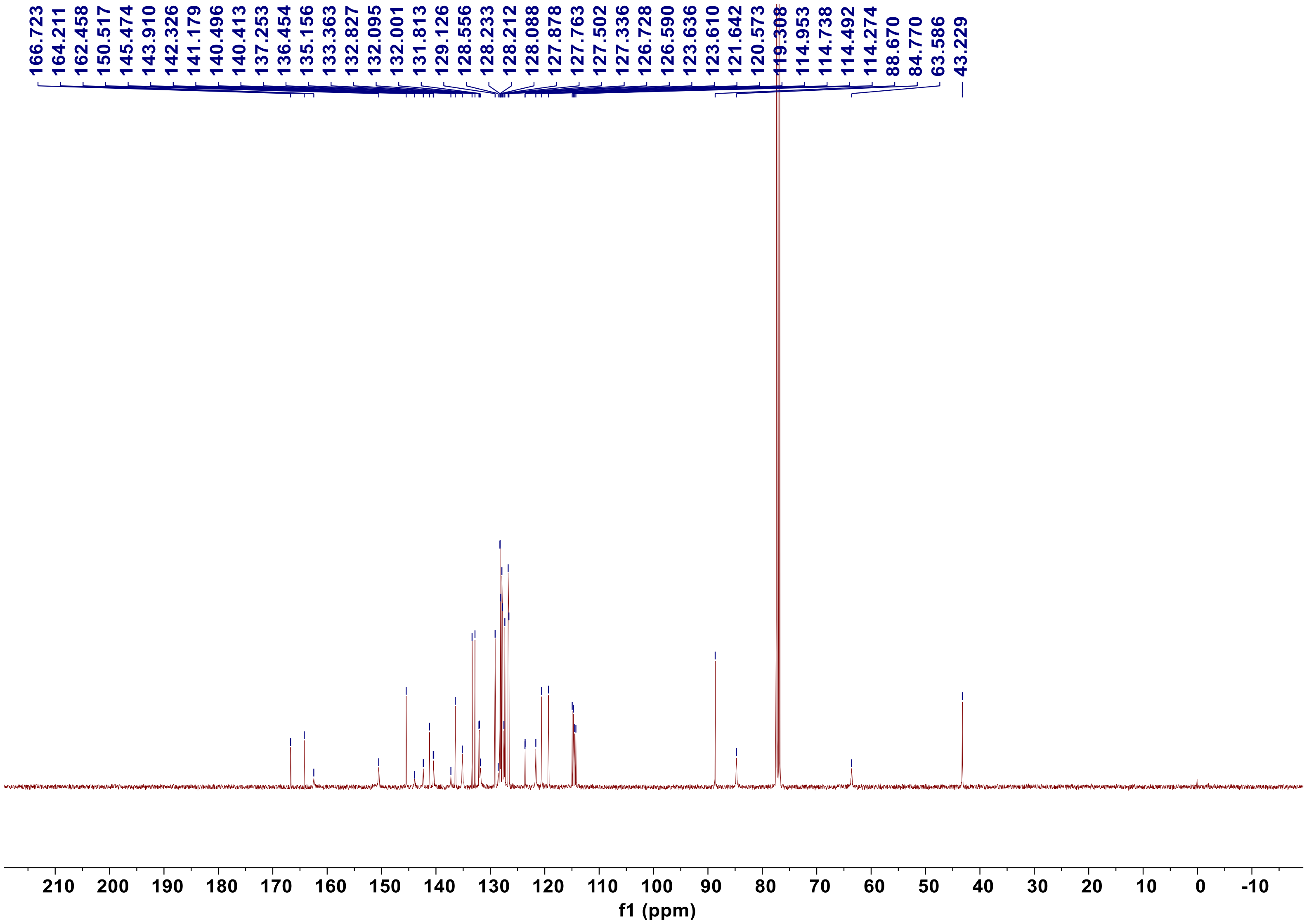

^{1}H NMR of **3g-17**

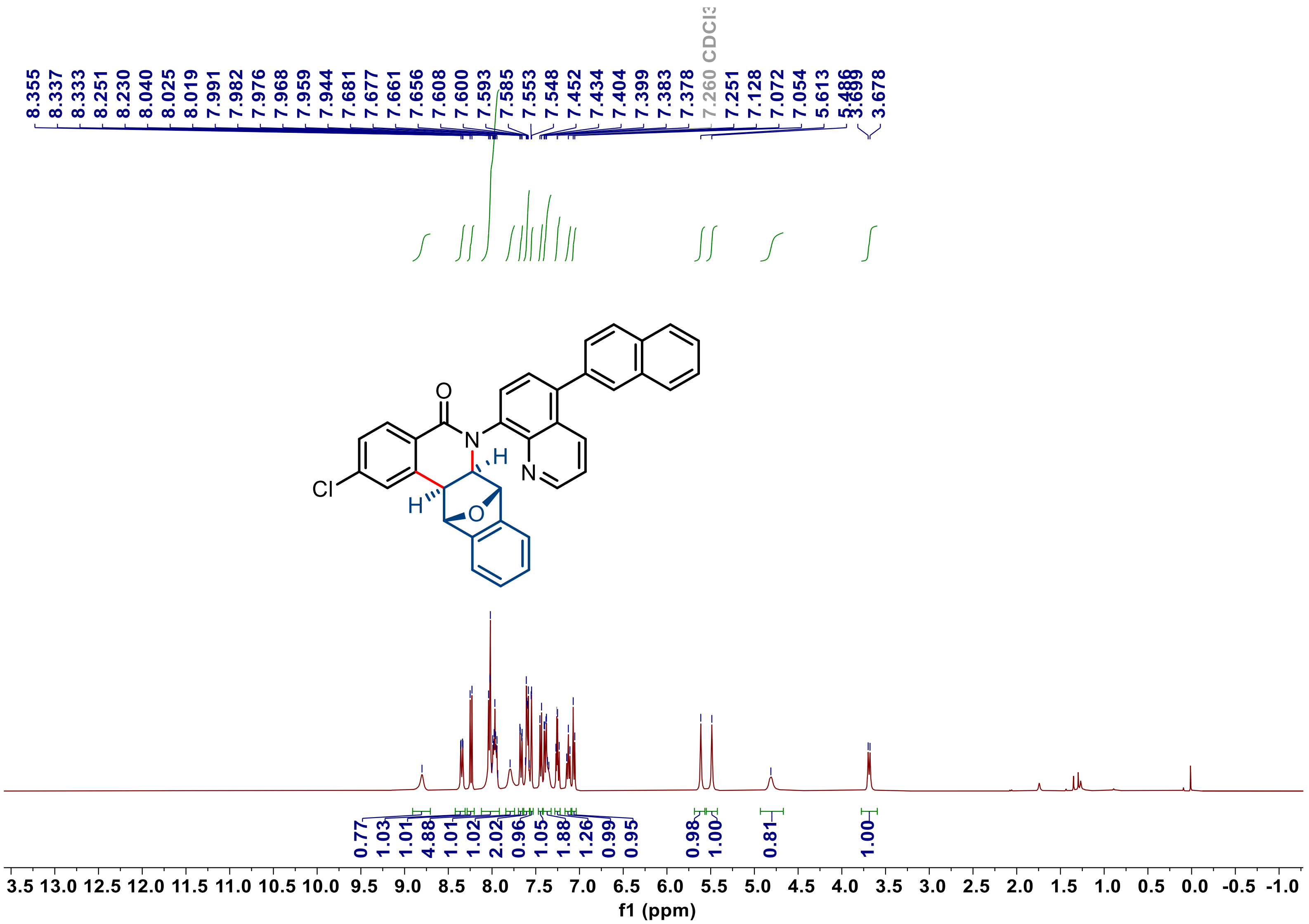

^{13}C NMR of **3g-17**

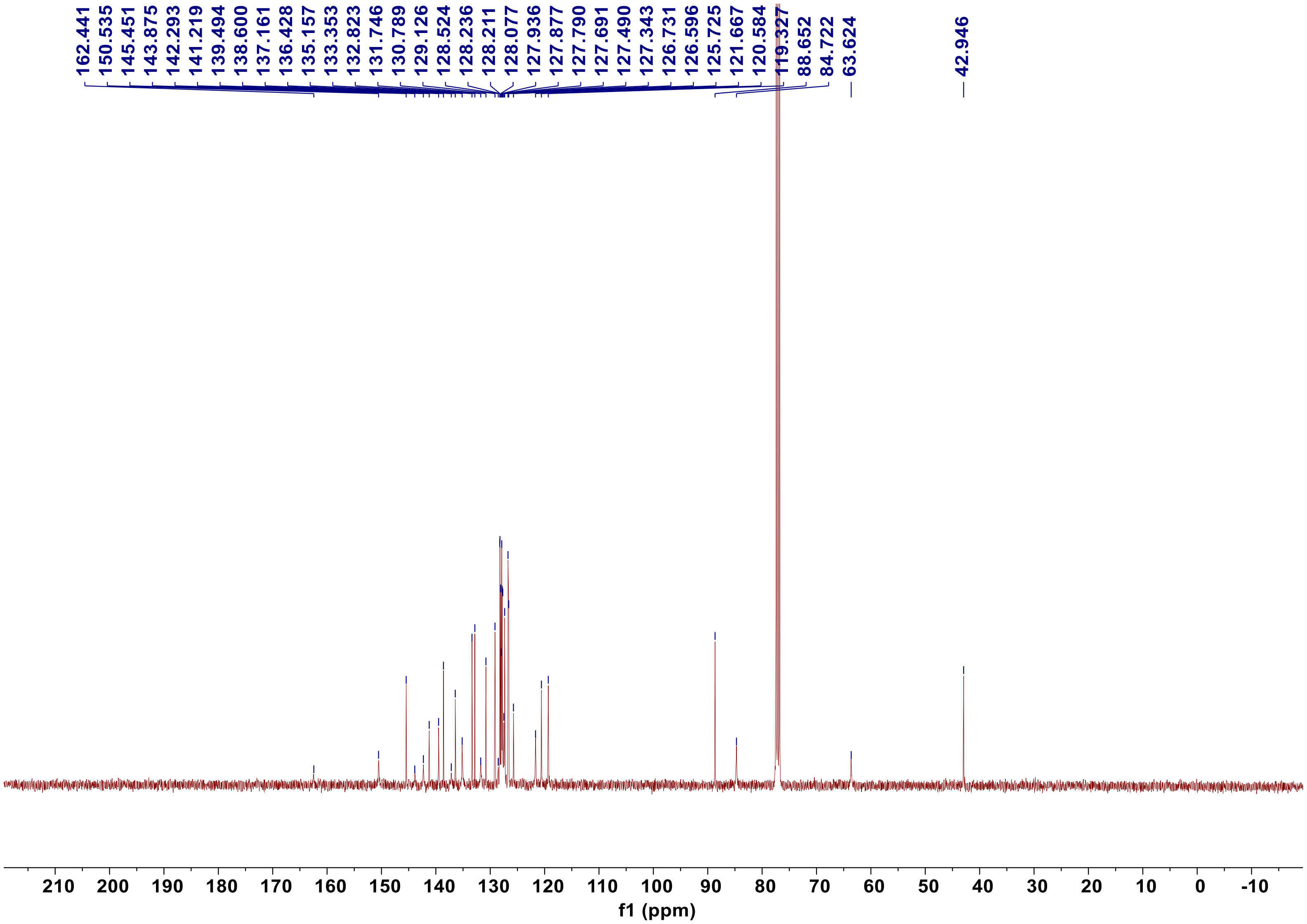

^{1}H NMR of **3g-18**

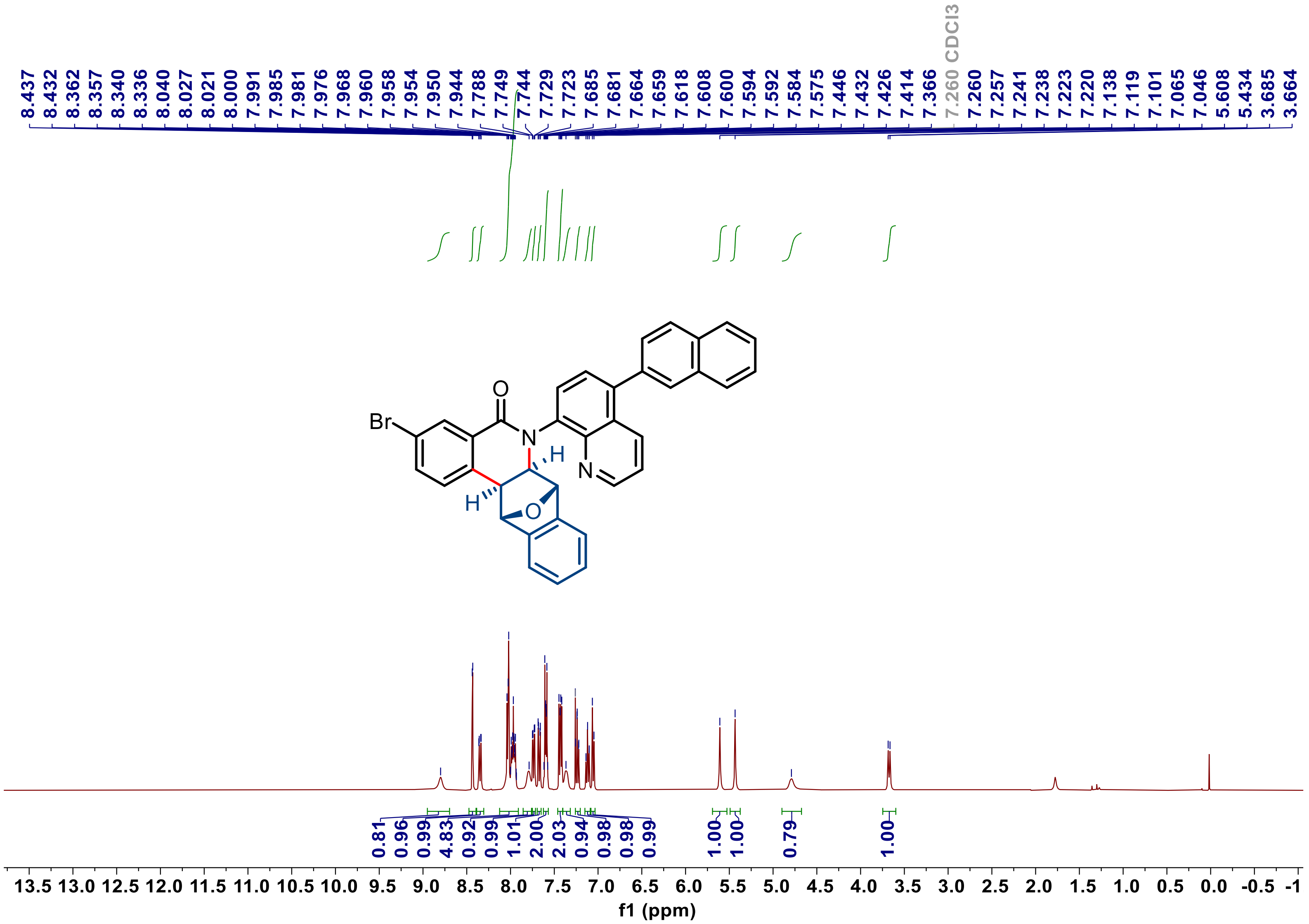

^{13}C NMR of **3g-18**

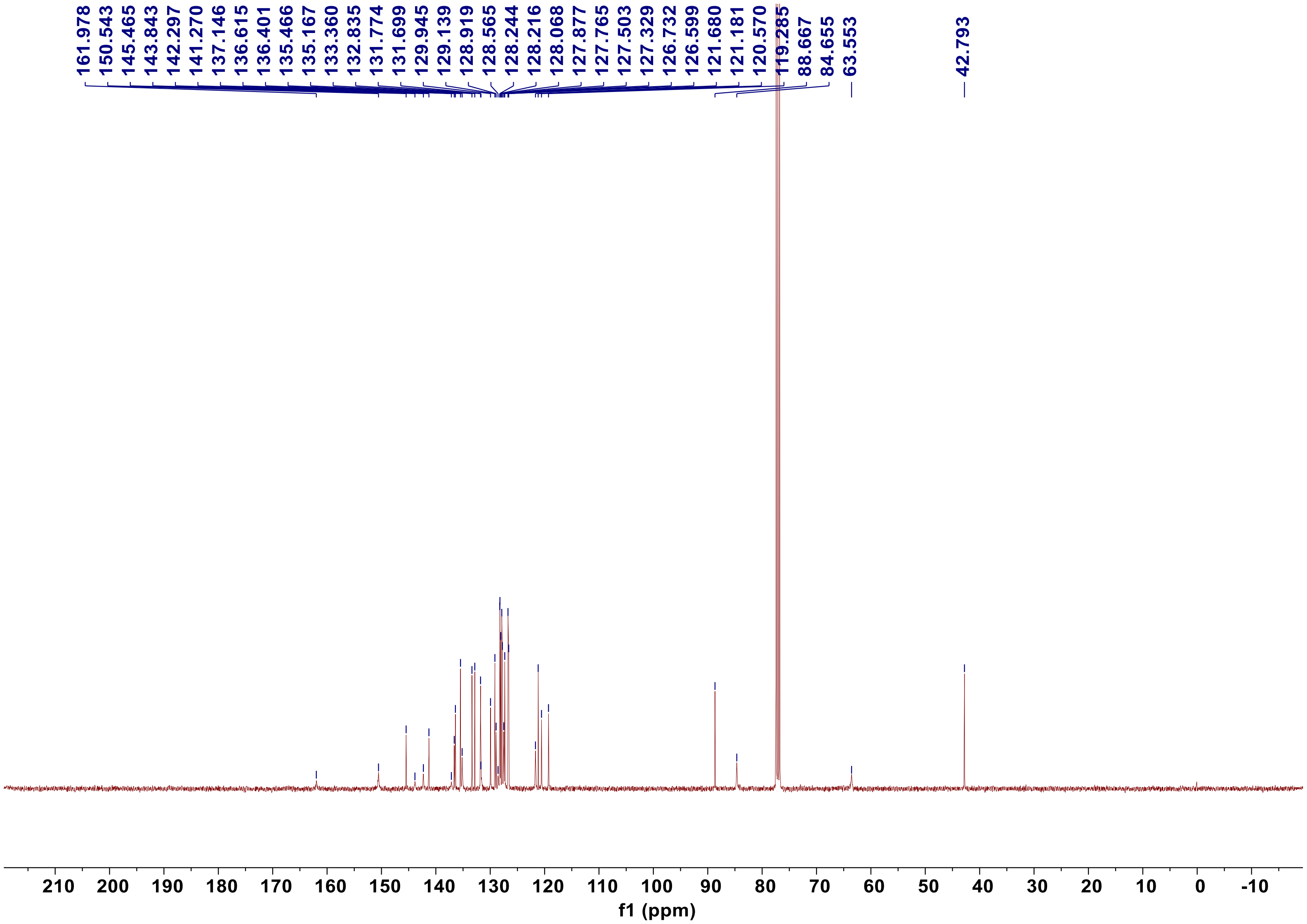

^{1}H NMR of **3g-19**

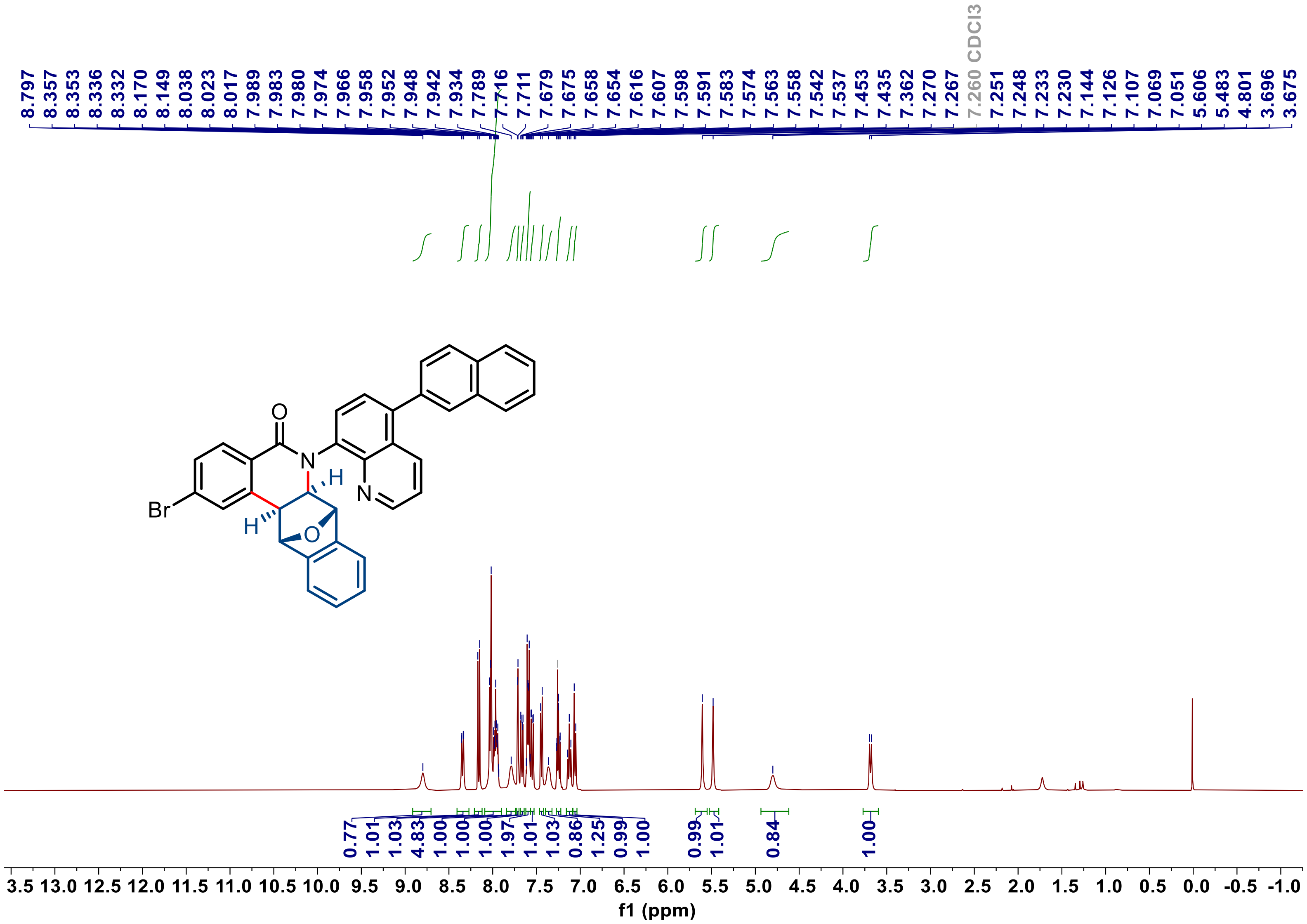

^{13}C NMR of **3g-19**

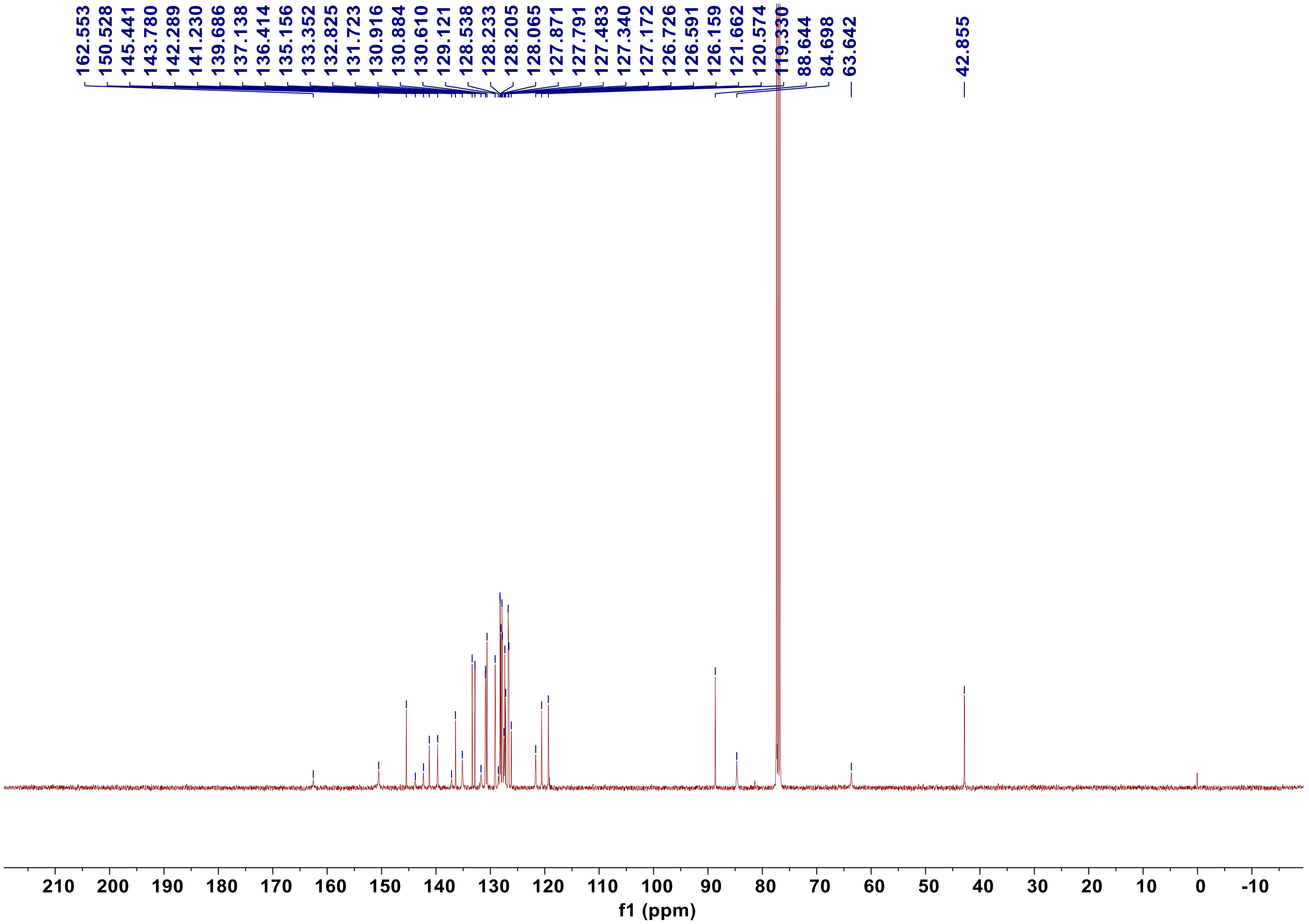

^{1}H NMR of **3g-20**

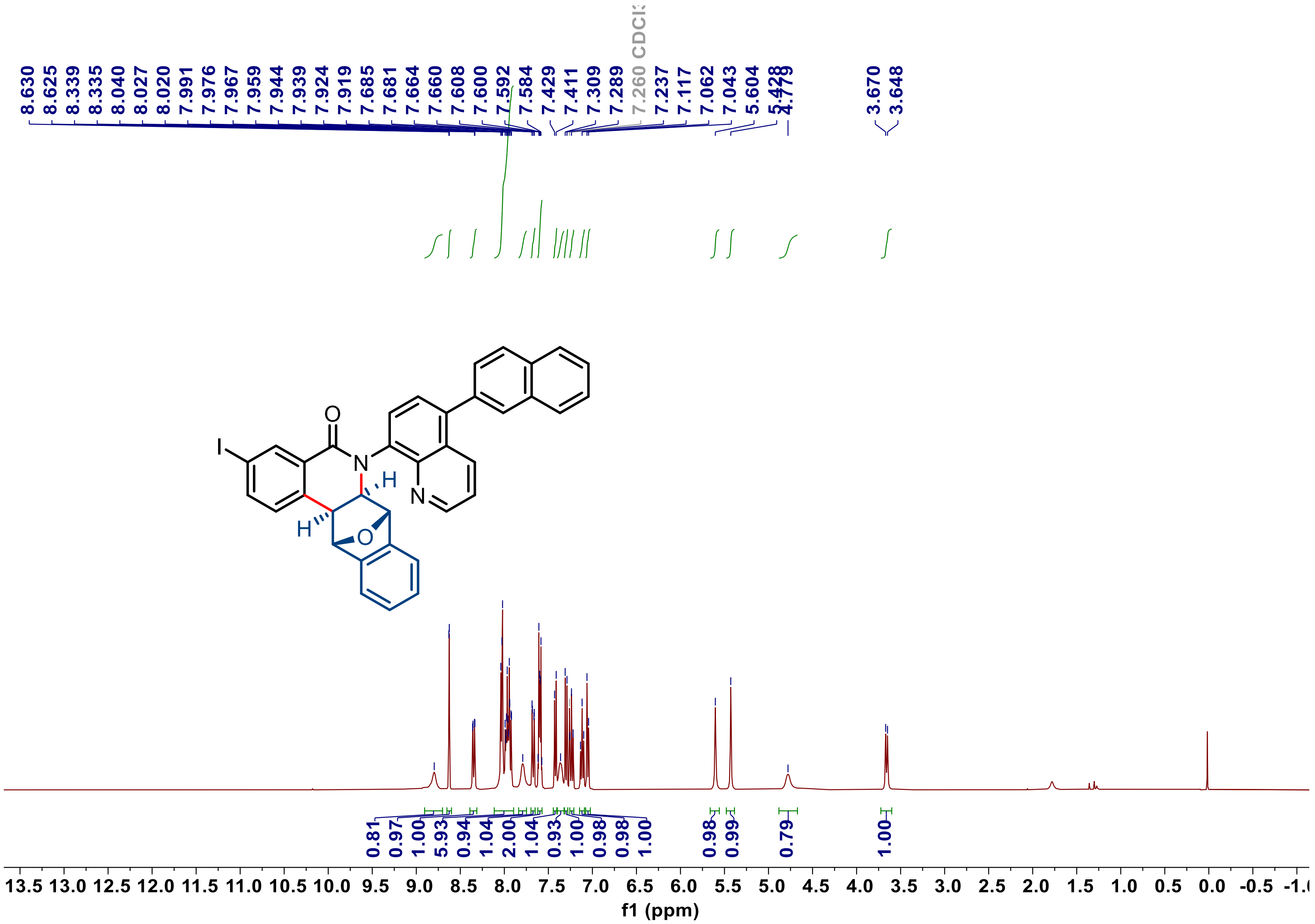

^{13}C NMR of **3g-20**

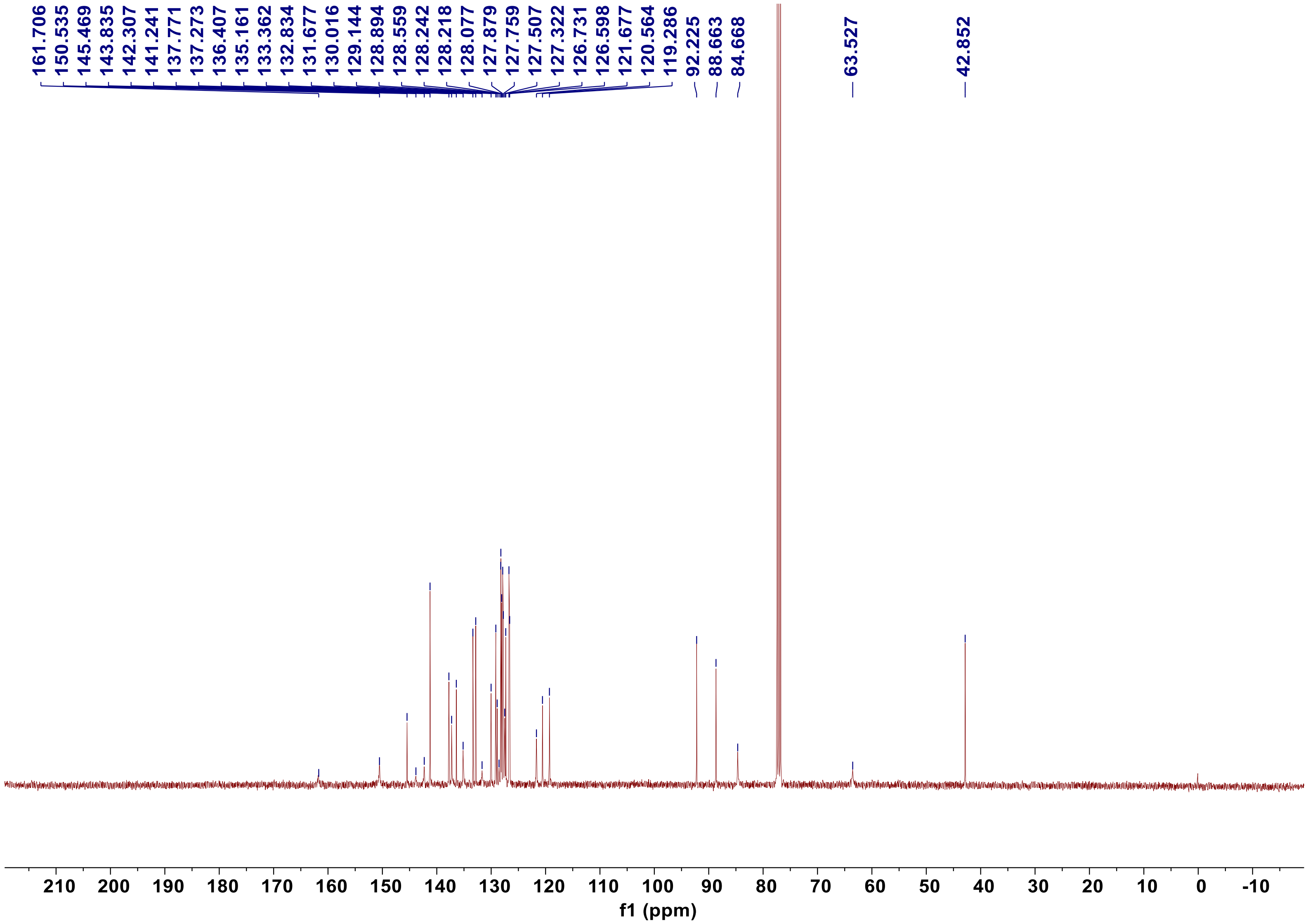

^{1}H NMR of **3g-21**

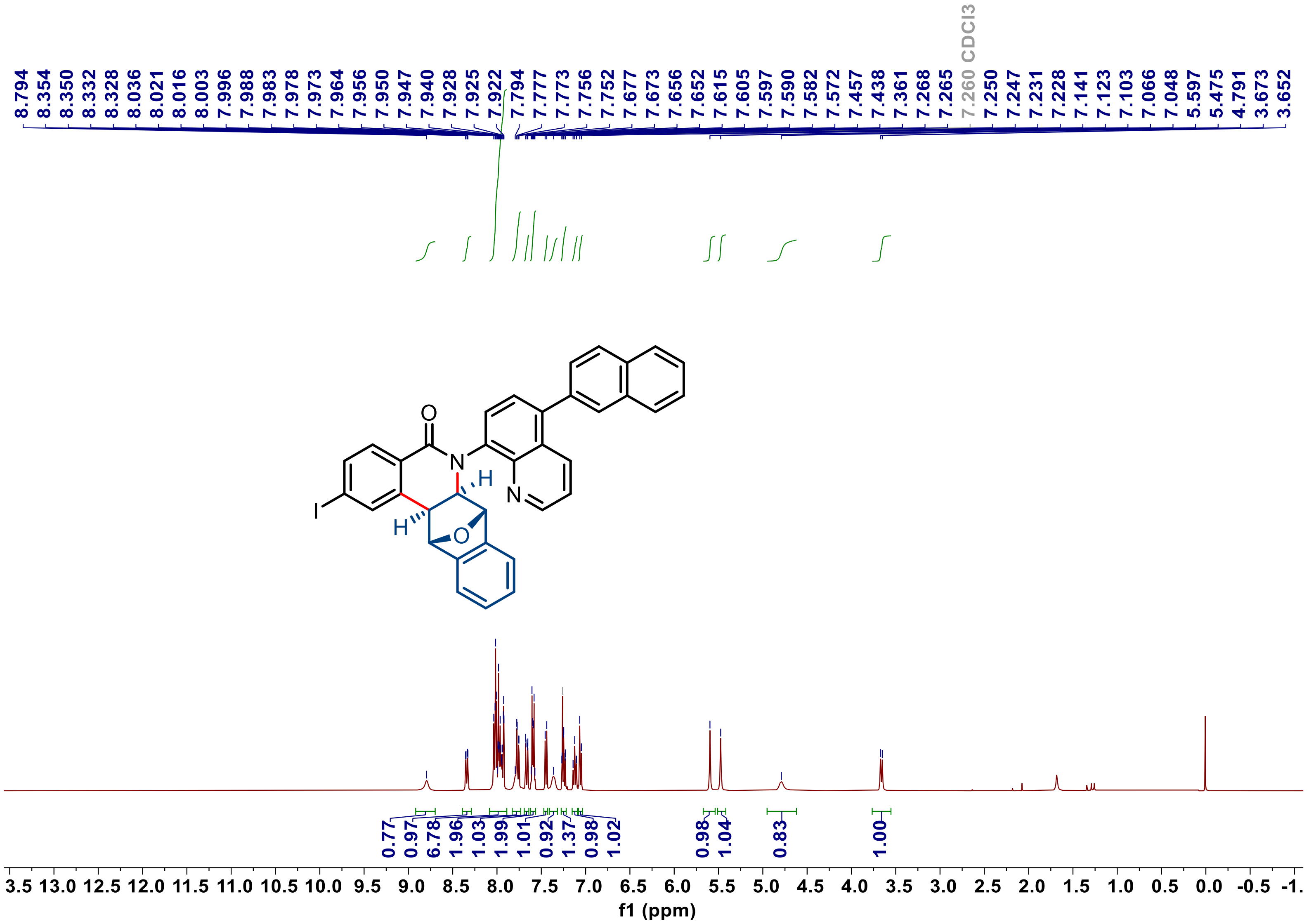

^{13}C NMR of **3g-21**

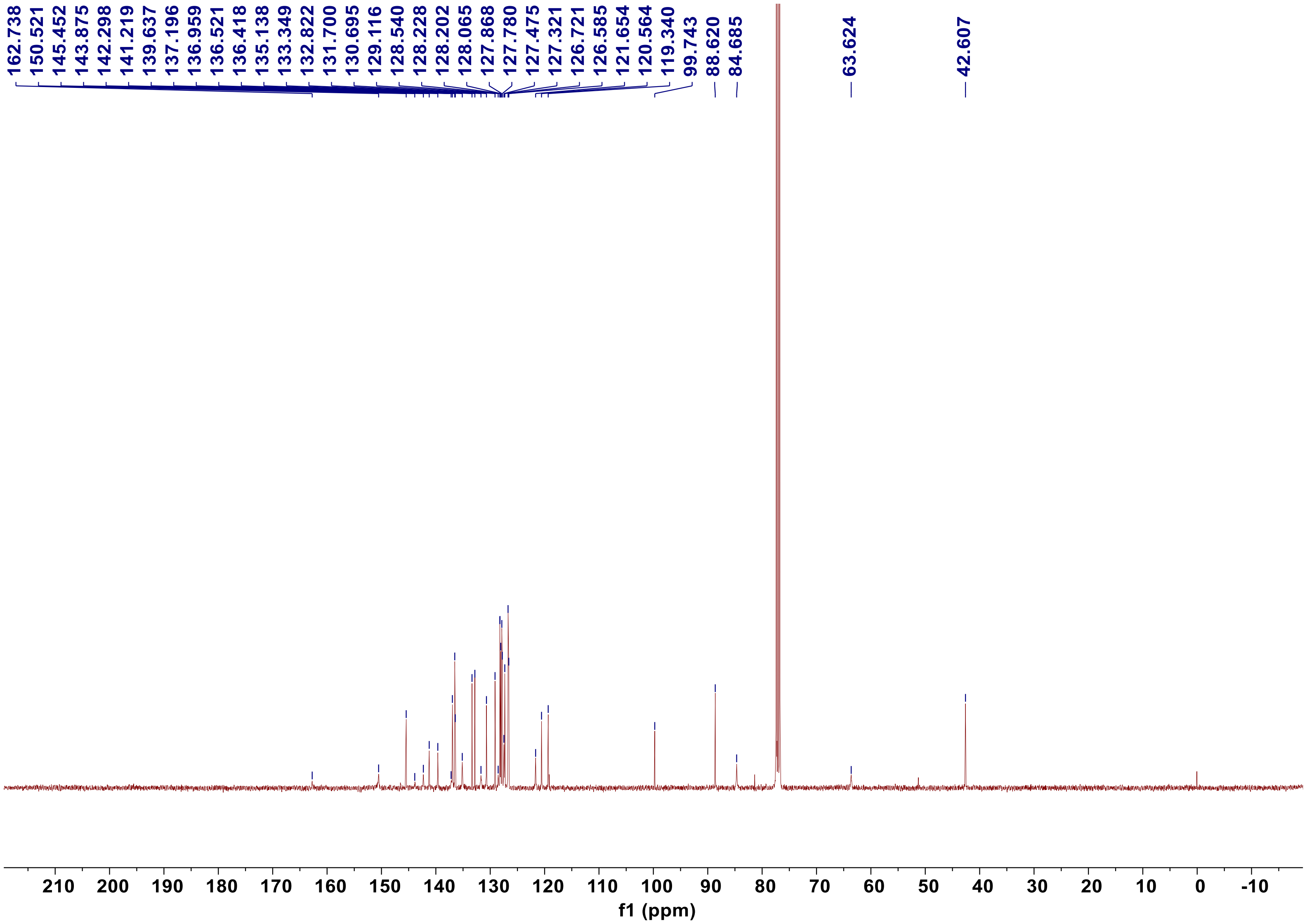

^{1}H NMR of **3g-22**

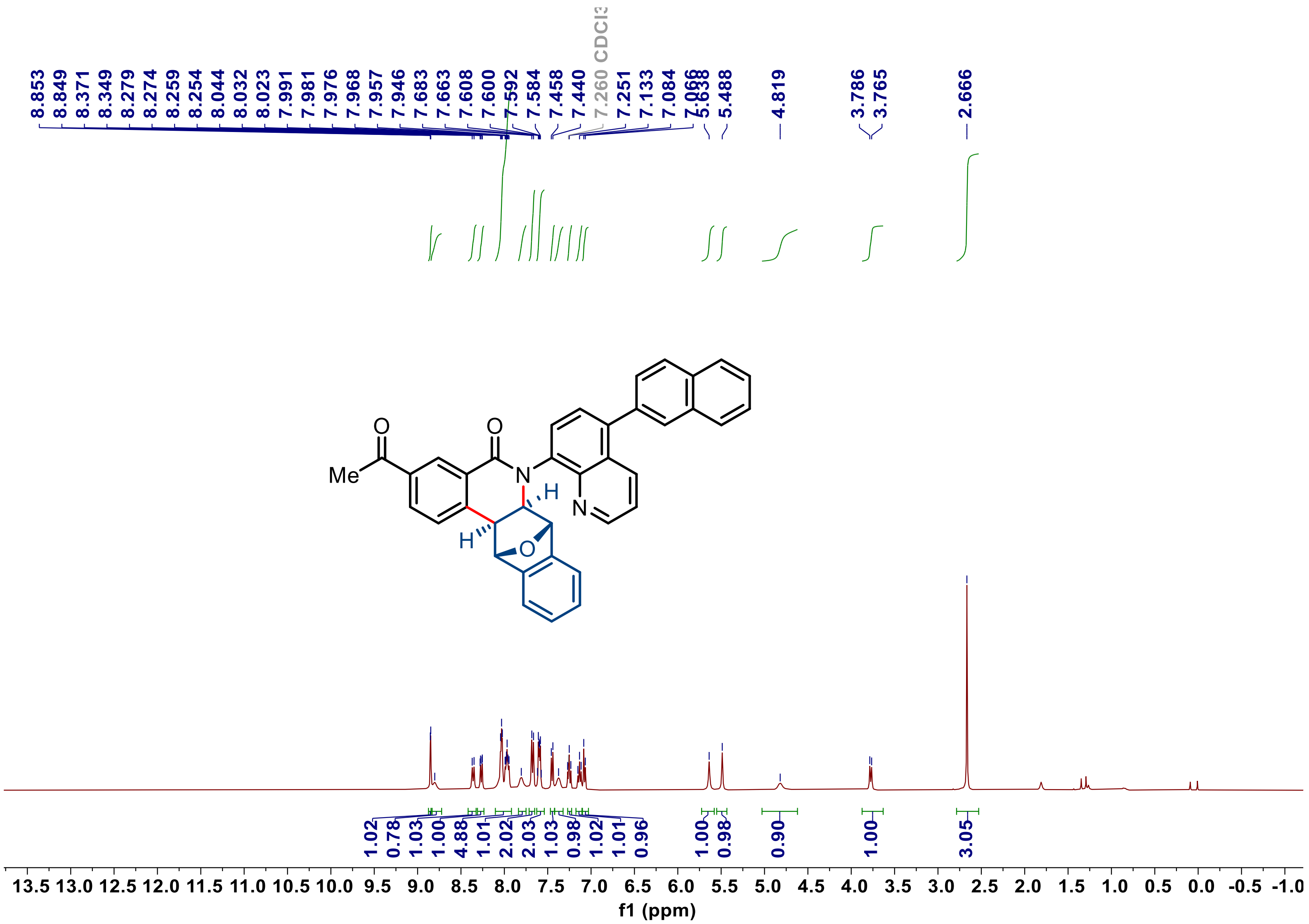

^{13}C NMR of **3g-22**

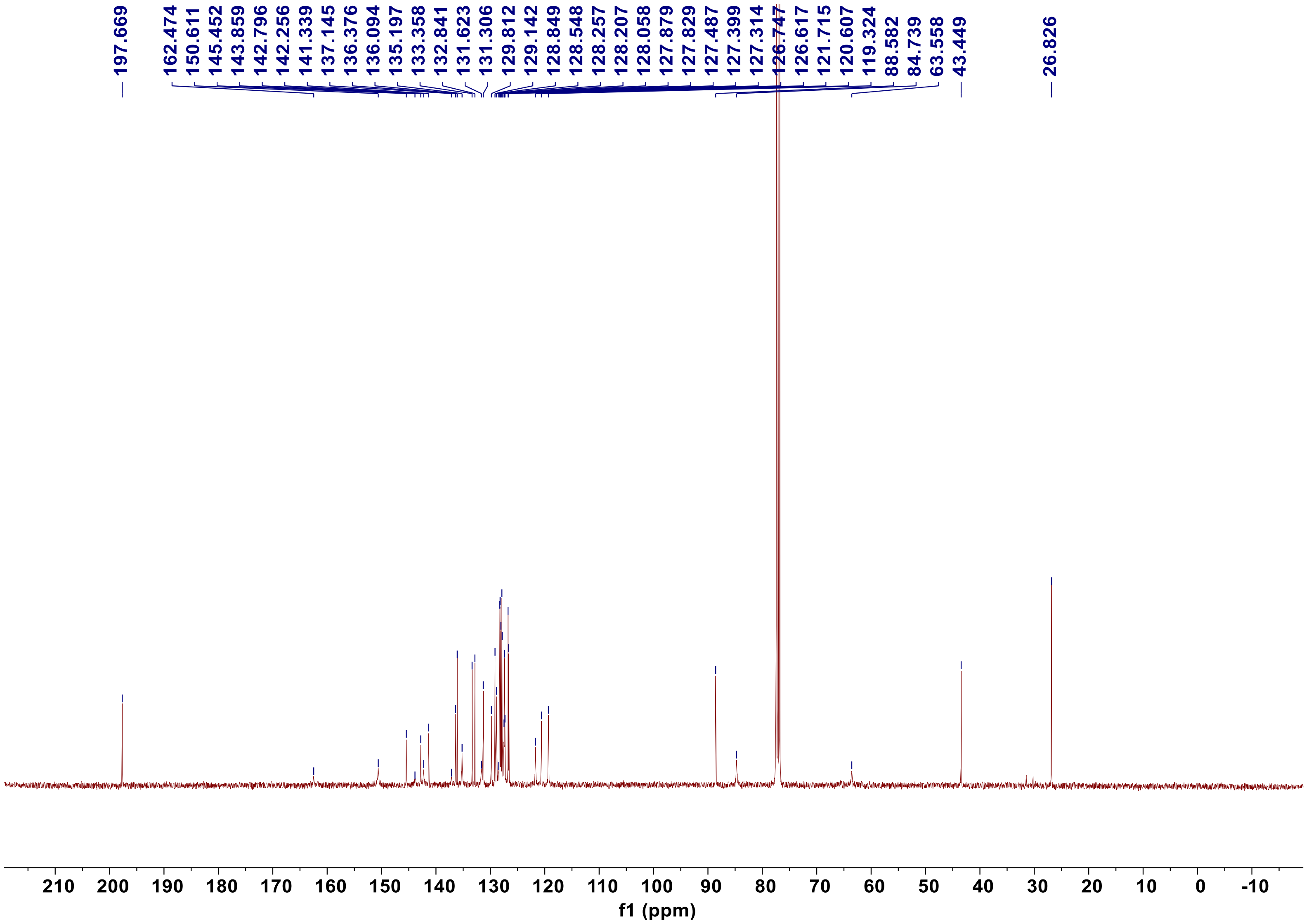

^{1}H NMR of **3g-23**

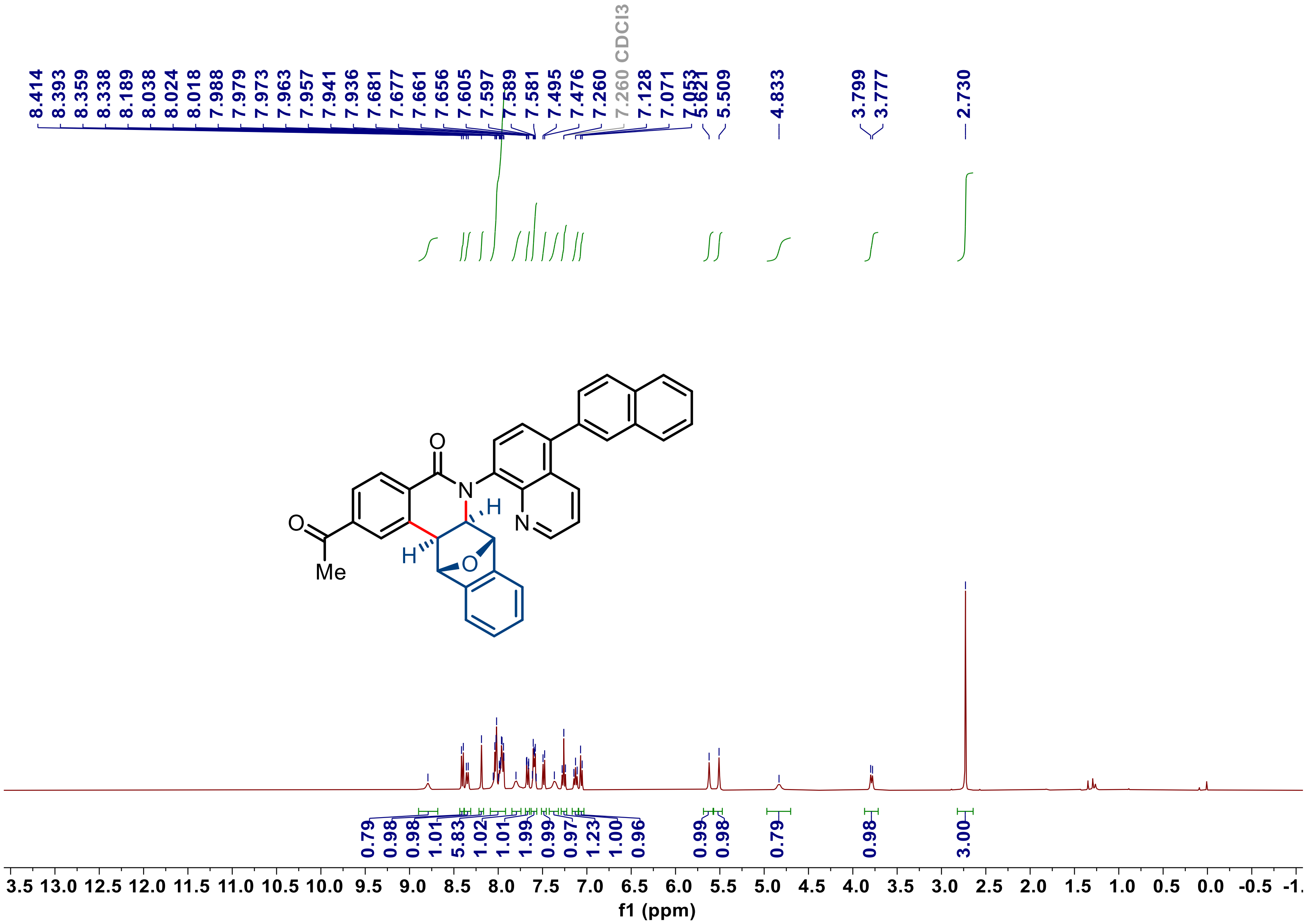

^{13}C NMR of **3g-23**

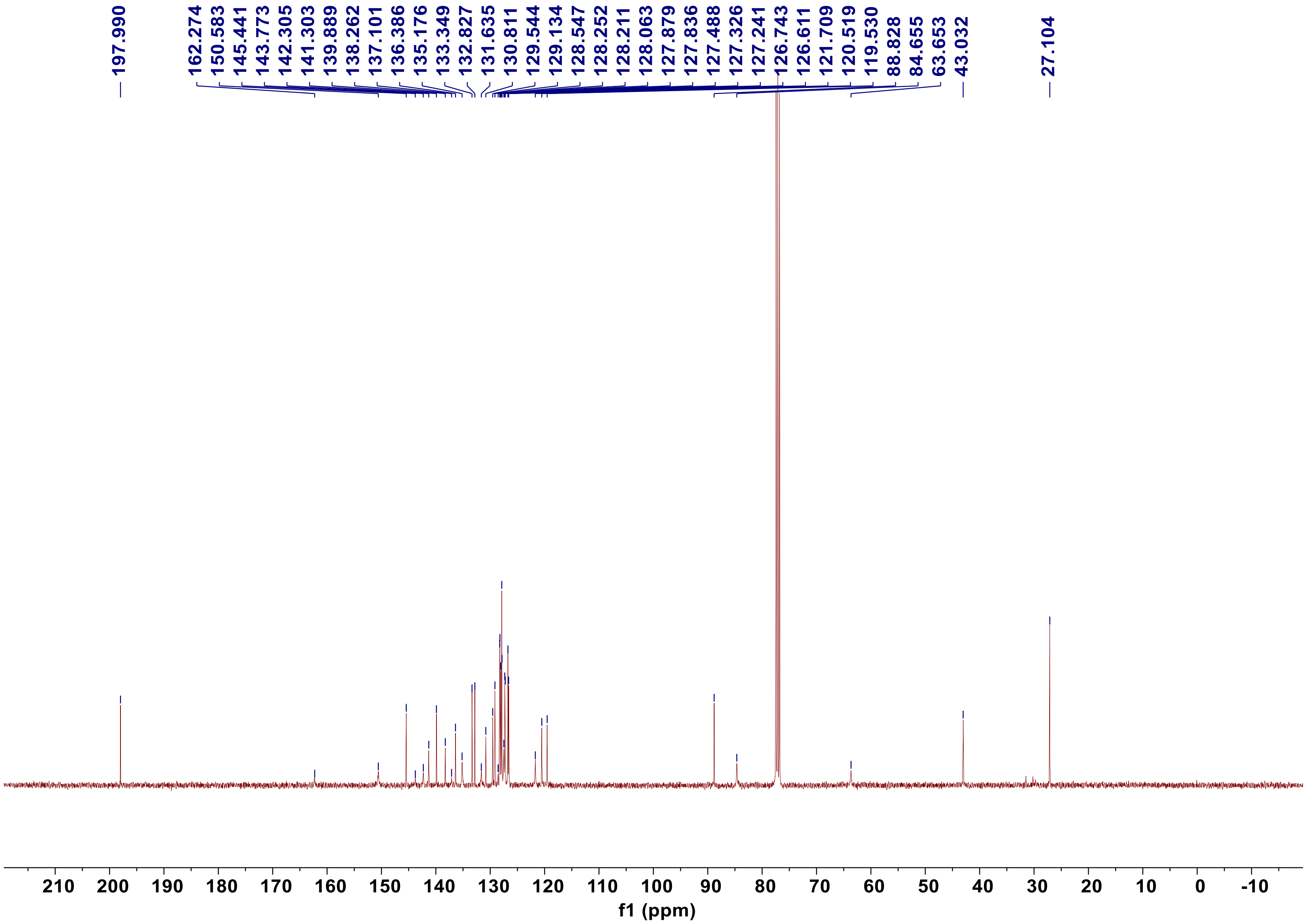

^{1}H NMR of **3g-24**

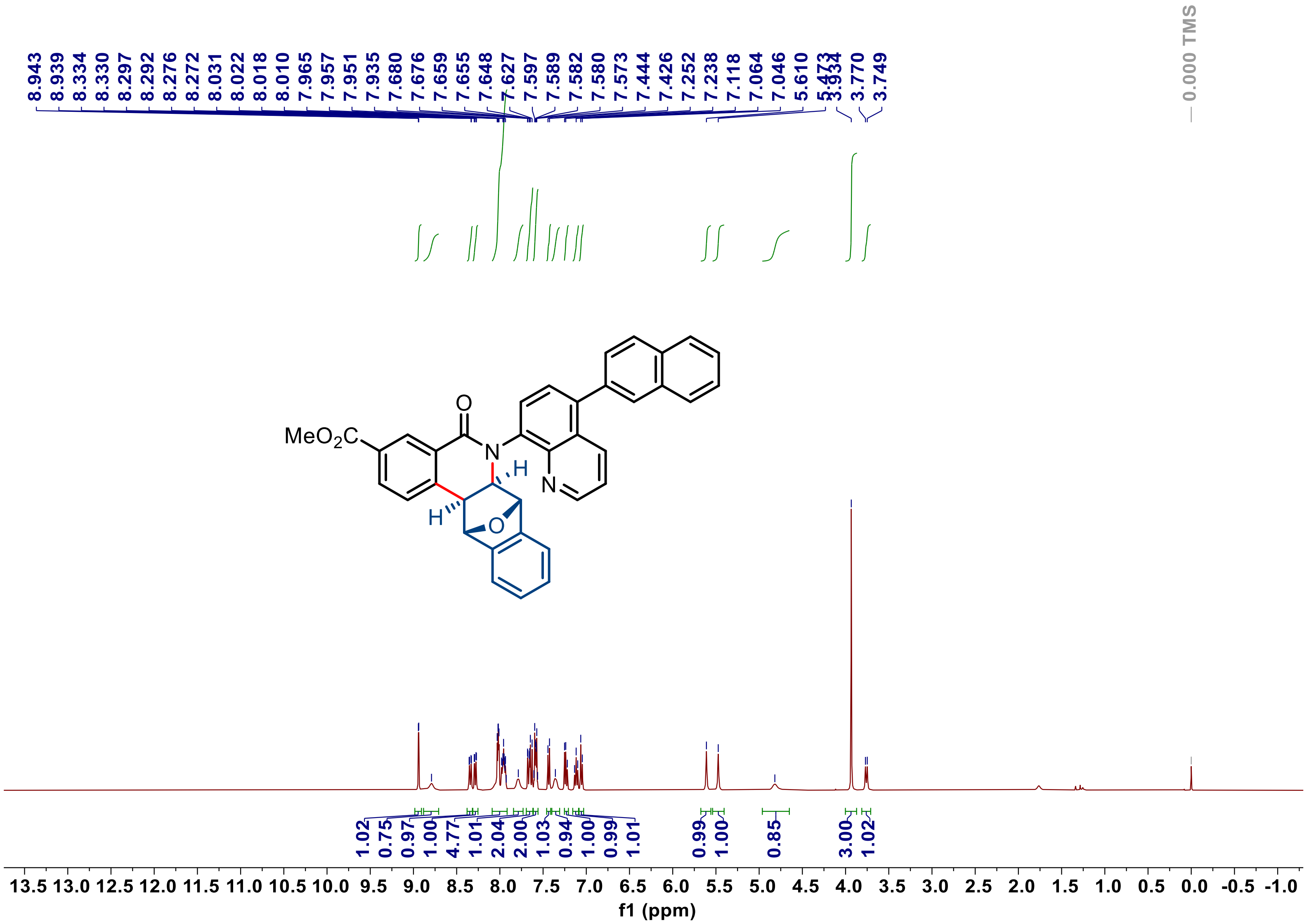

^{13}C NMR of **3g-24**

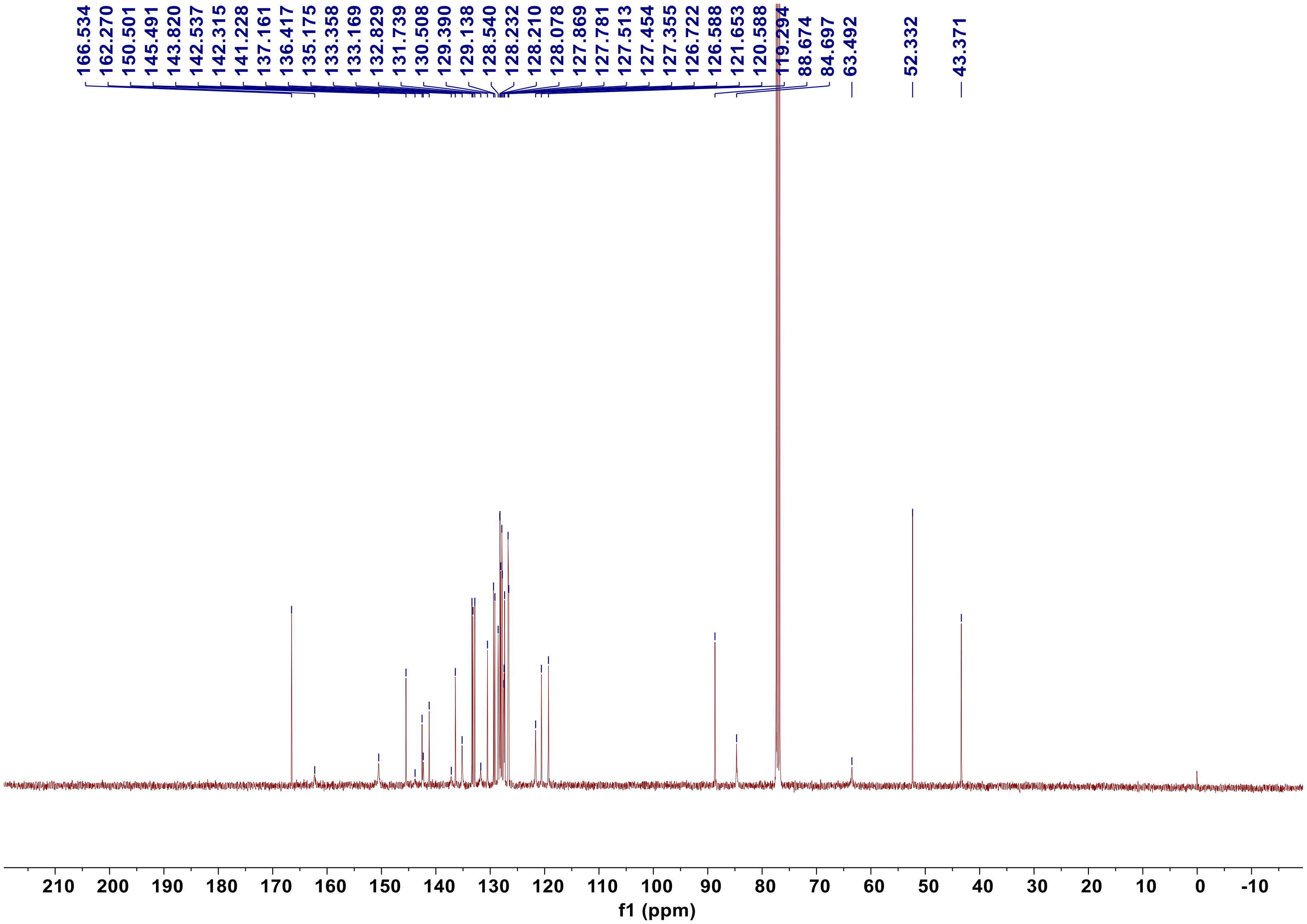

^{1}H NMR of **3g-25**

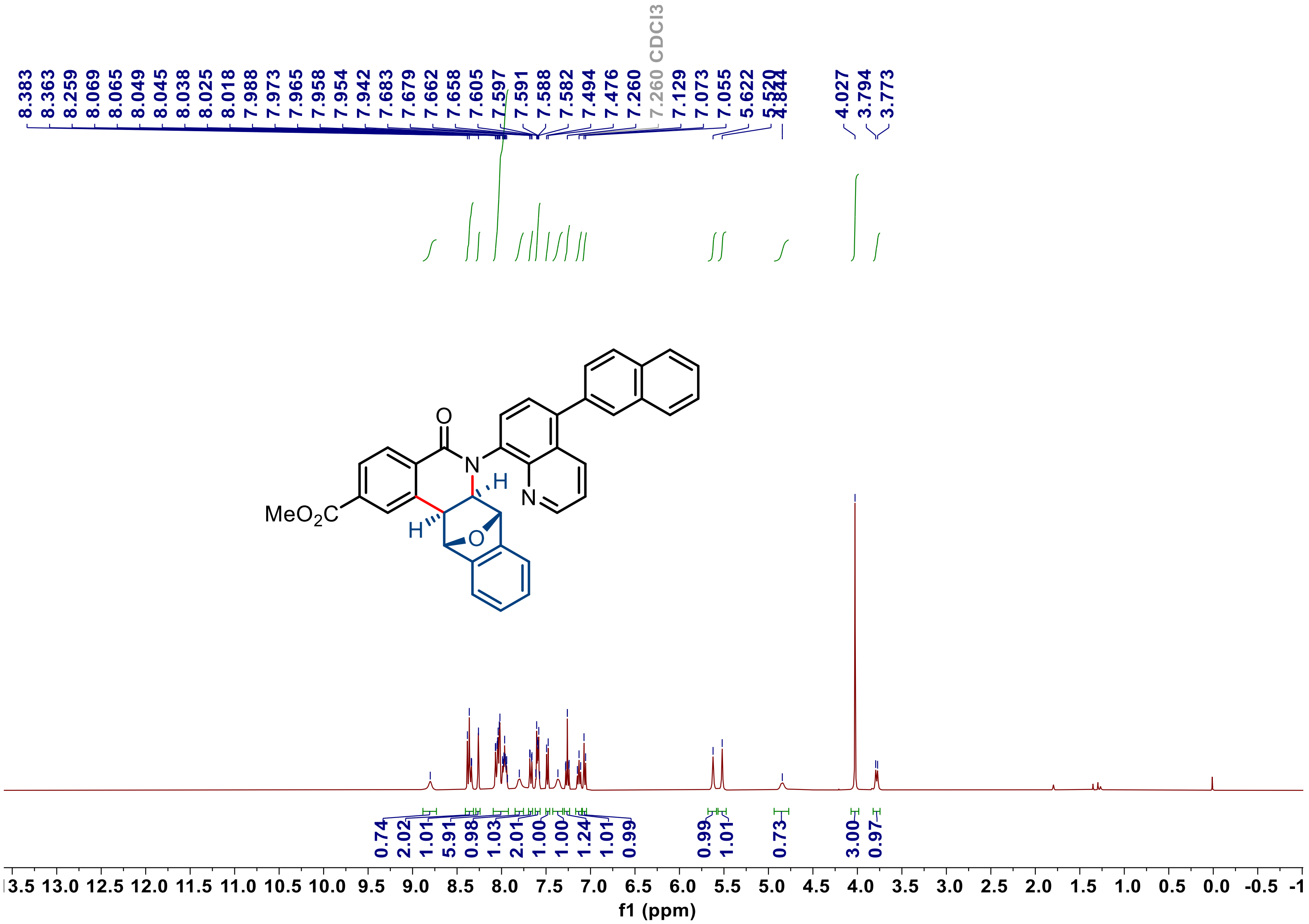

^{13}C NMR of **3g-25**

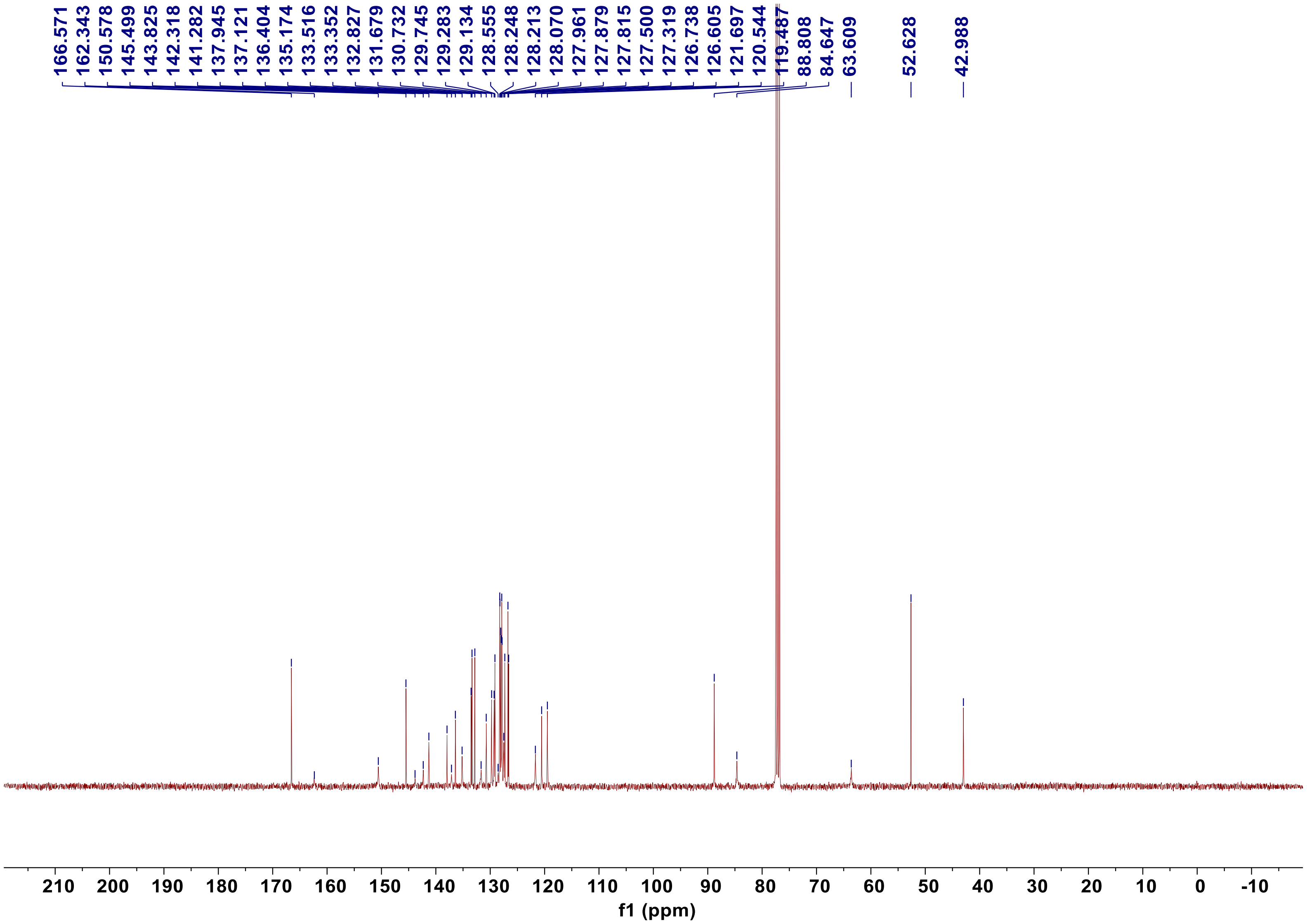

^{1}H NMR of **3g-26**

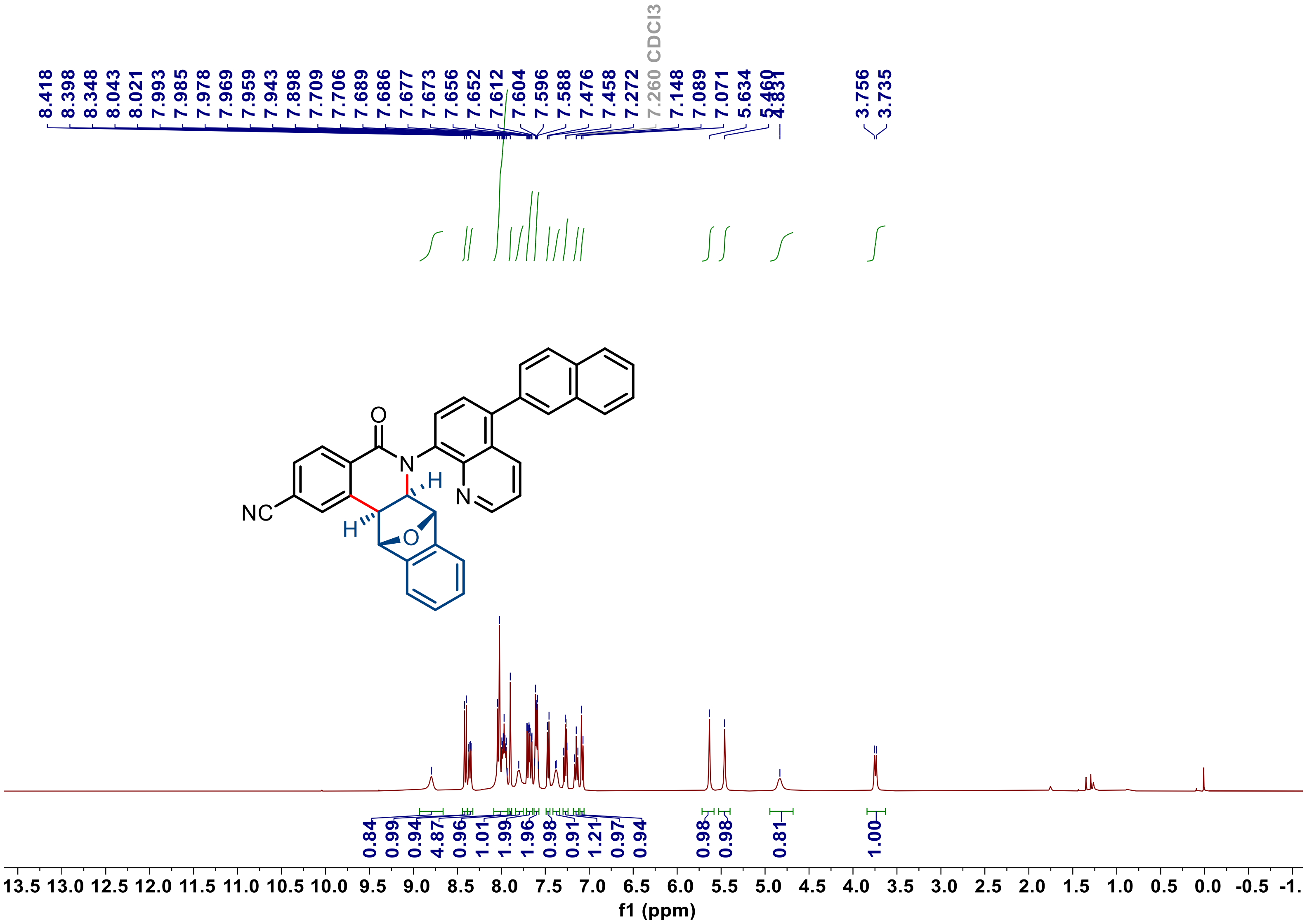

^{13}C NMR of **3g-26**

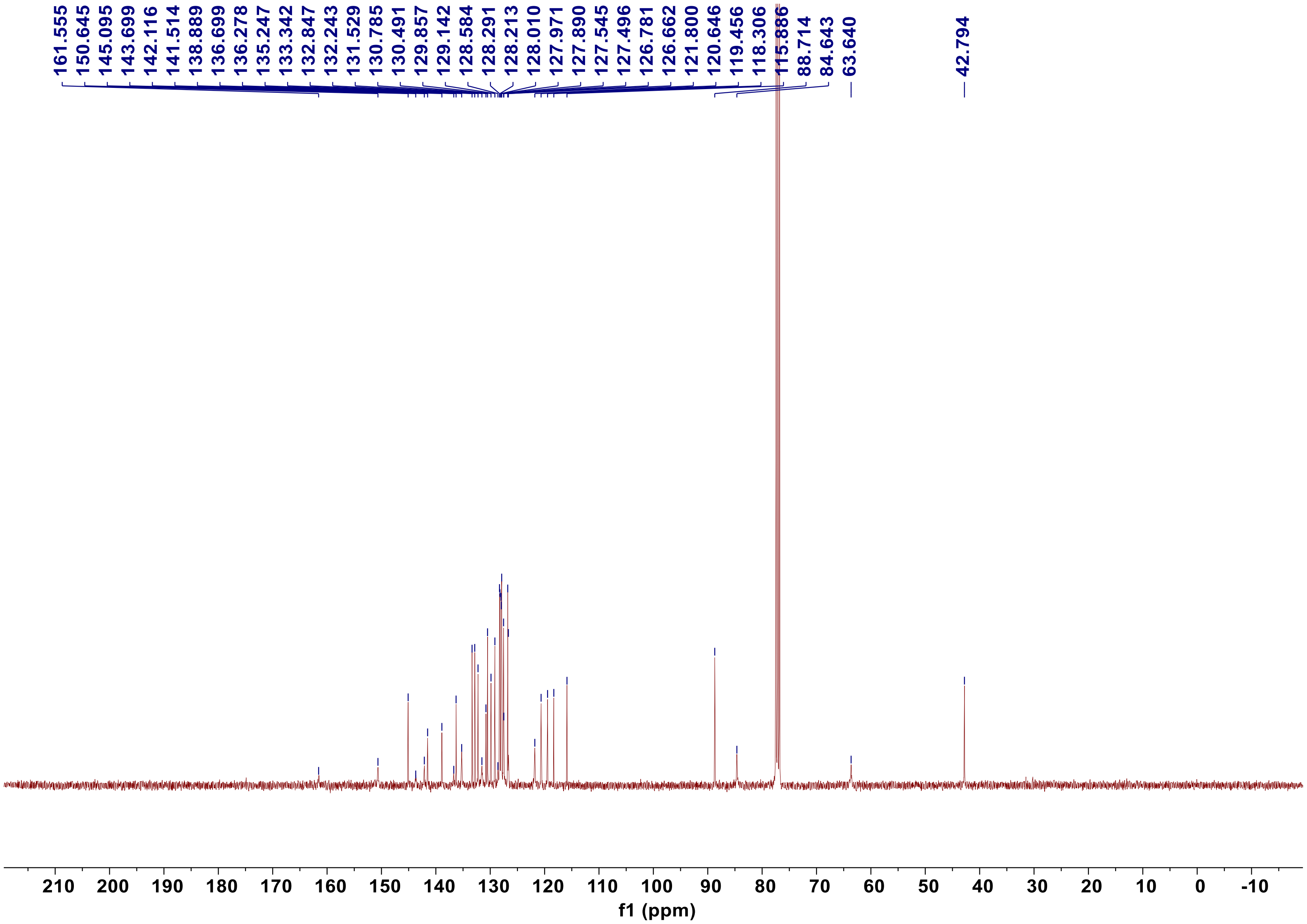

^{1}H NMR of **3g-27**

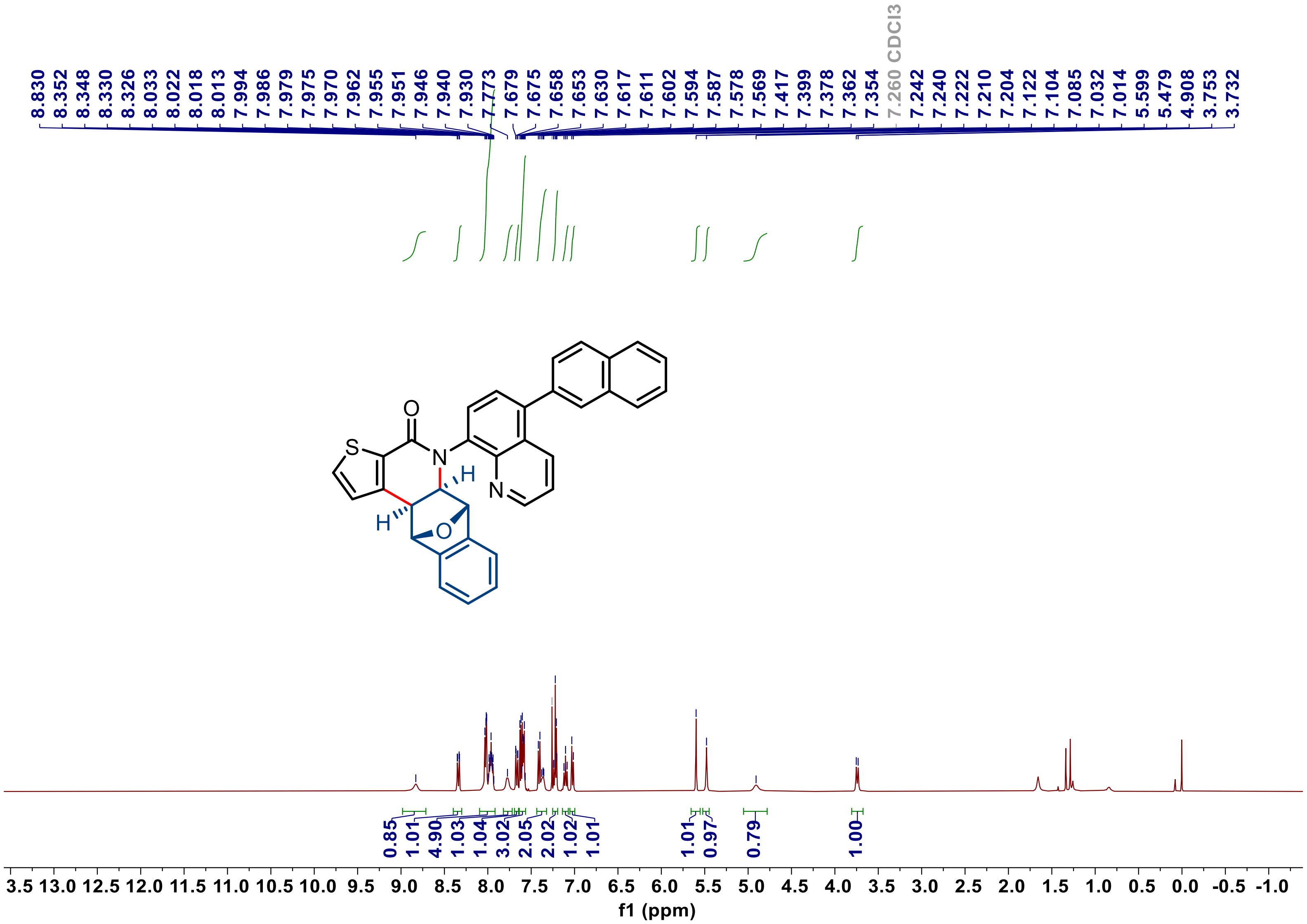

^{13}C NMR of **3g-27**

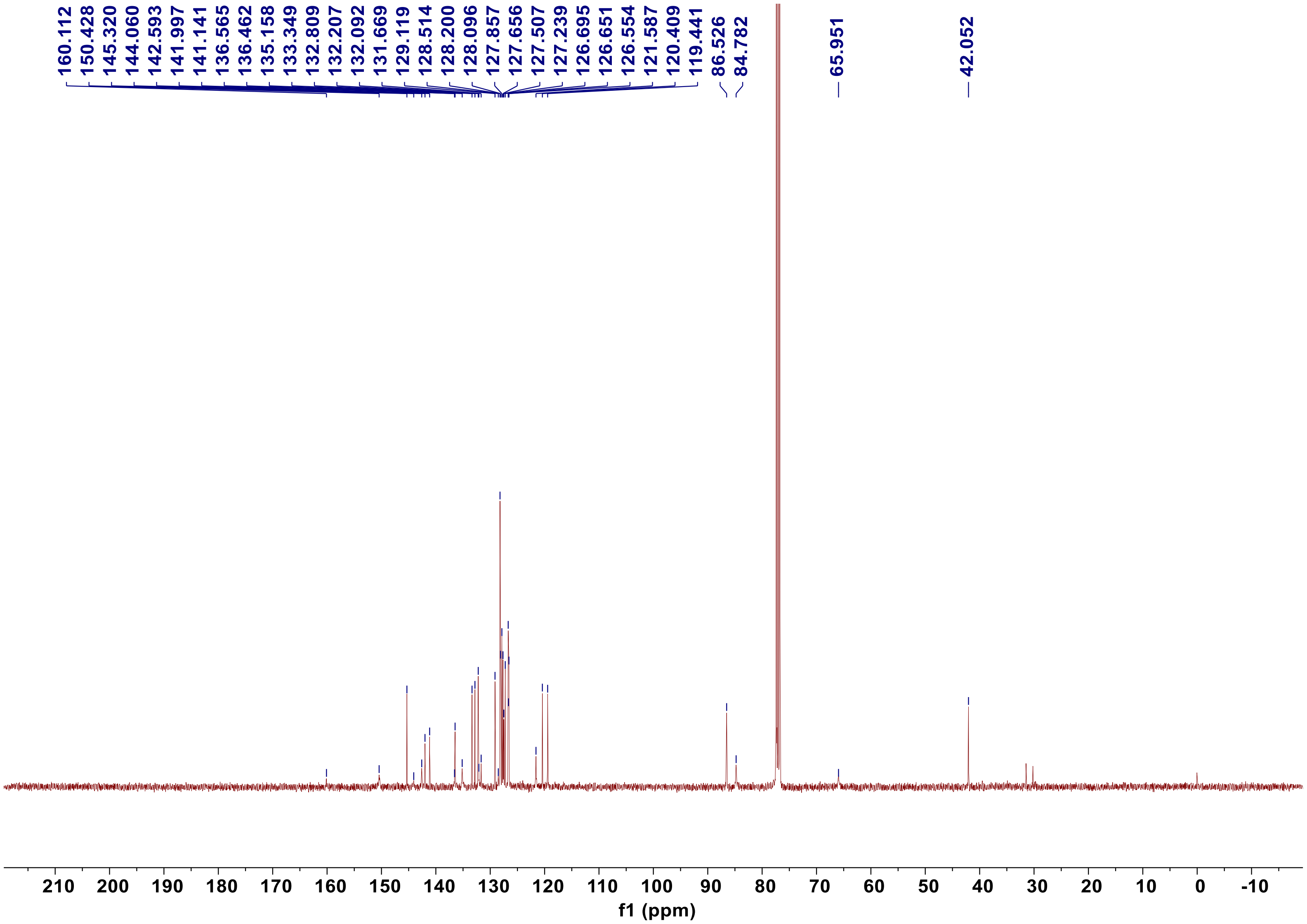

^{1}H NMR of **4g-1**

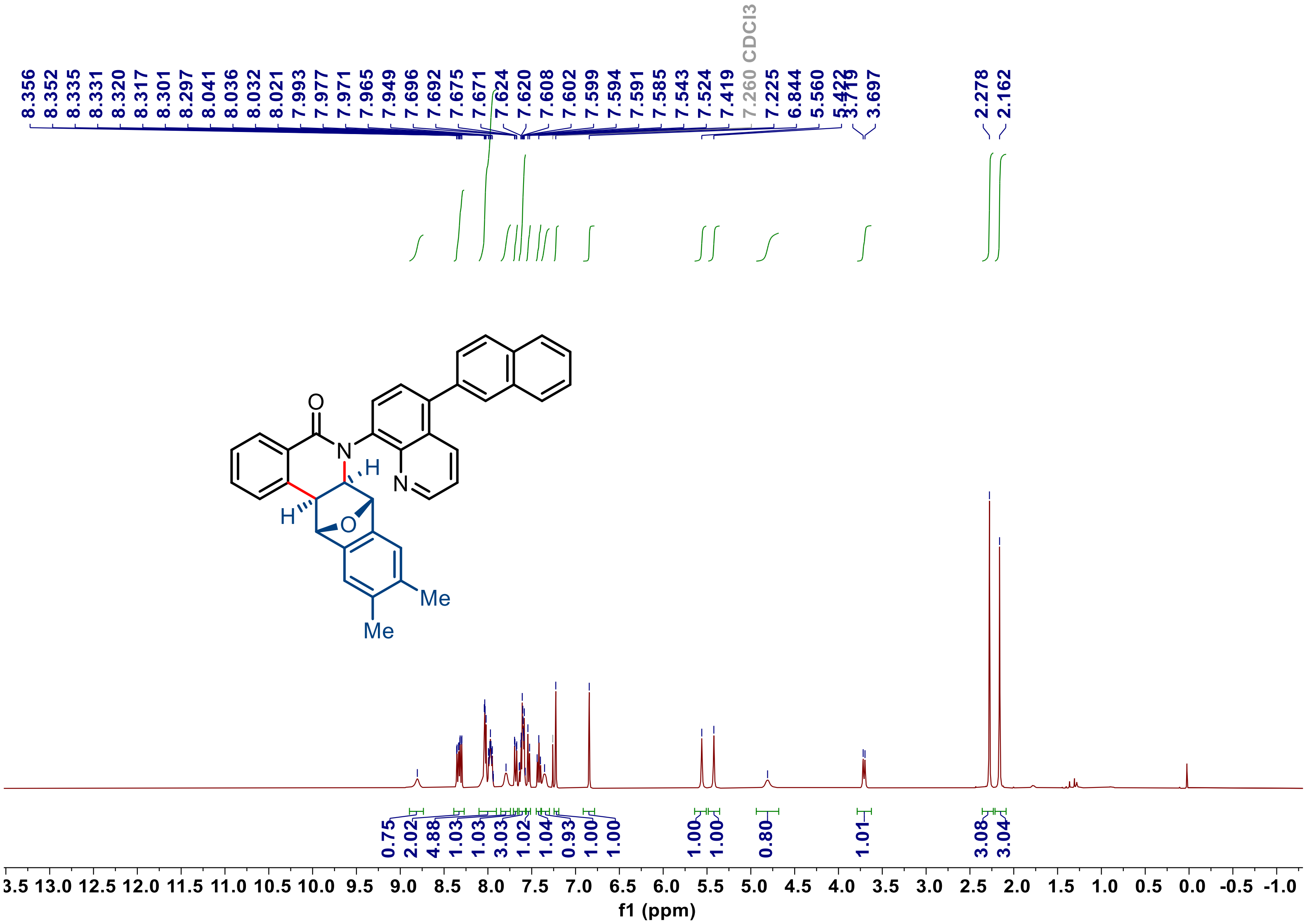

^{13}C NMR of **4g-1**

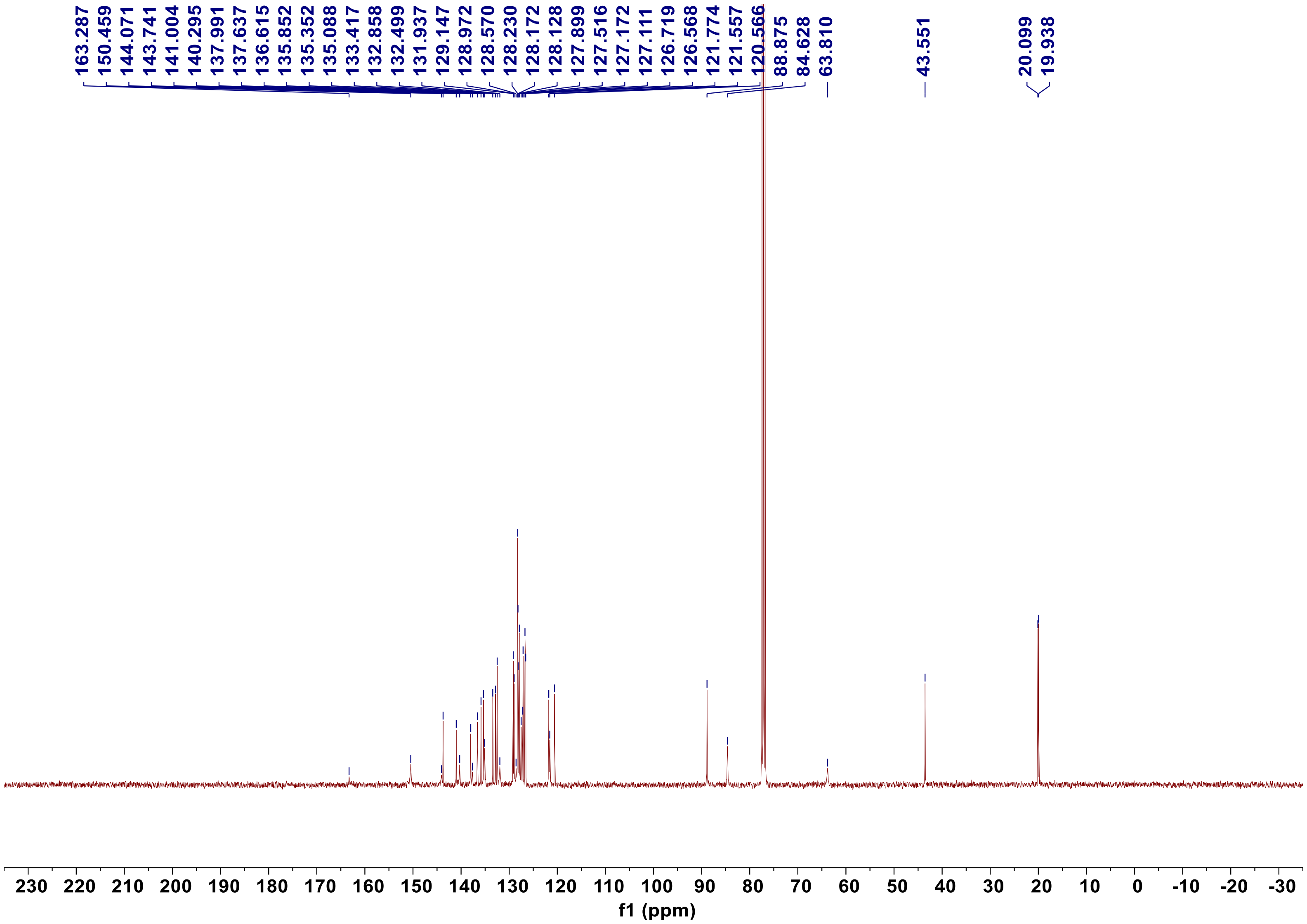

^{1}H NMR of **5g-1**

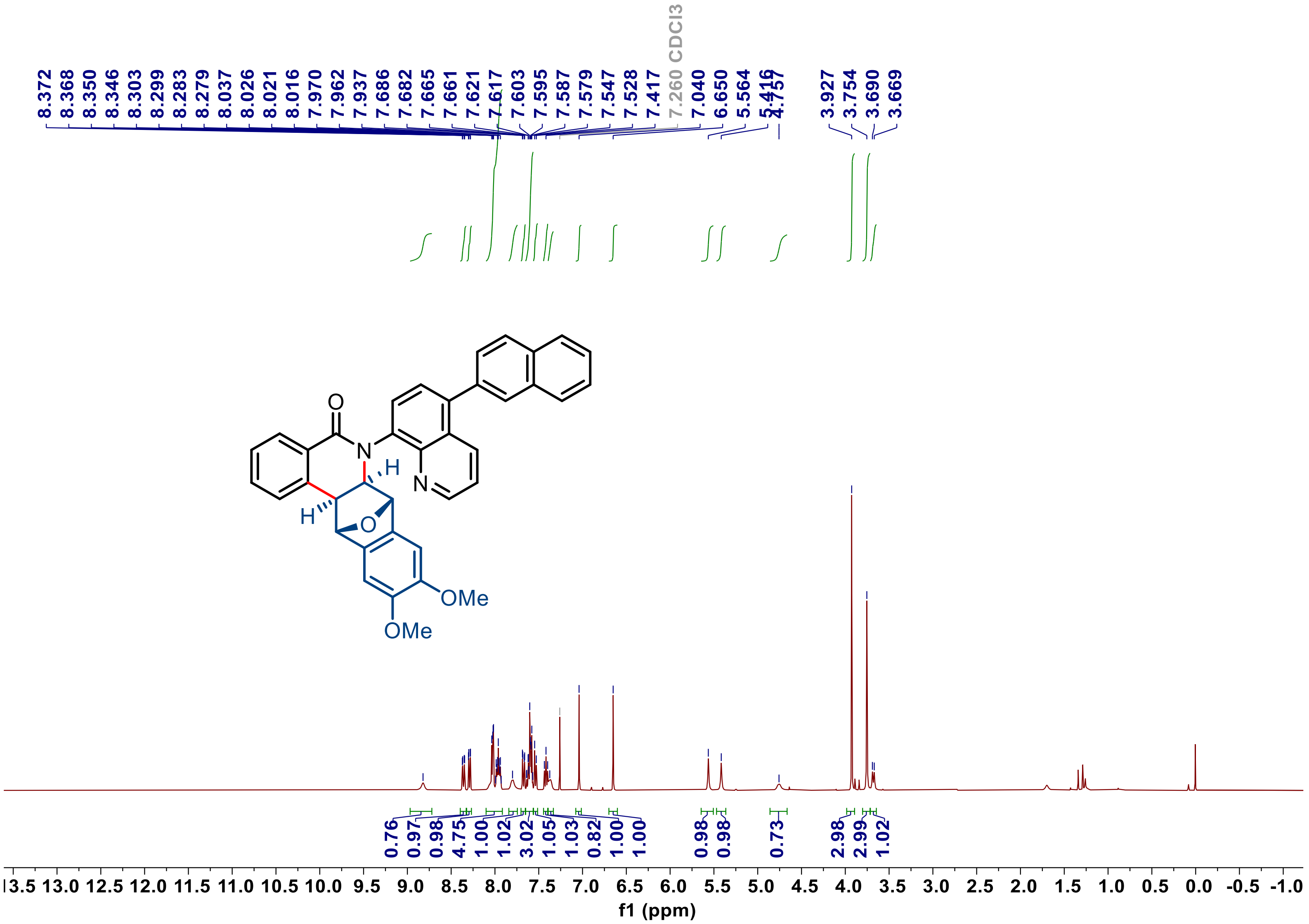
8.372
8.368
8.350
8.346
8.303
8.299
8.283
8.279
8.037
8.026
8.021
8.016
7.970
7.962
7.937
7.686
7.682
7.665
7.661
7.621
7.617
7.603
7.595
7.587
7.579
7.547
7.528
7.417
7.260 CDCl3
7.040
6.650
5.564
5.416
4.757
3.927
3.754
3.690
3.669
O
N
H
N
H
O
OMe
OMe
0.76
0.97
0.98
4.75
1.00
1.02
3.02
1.05
1.03
0.82
1.00
1.00
0.98
0.98
0.73
2.98
2.99
1.02
13.5 13.0 12.5 12.0 11.5 11.0 10.5 10.0 9.5 9.0 8.5 8.0 7.5 7.0 6.5 6.0 5.5 5.0 4.5 4.0 3.5 3.0 2.5 2.0 1.5 1.0 0.5 0.0 -0.5 -1.0
f1 (ppm)

^{13}C NMR of **5g-1**

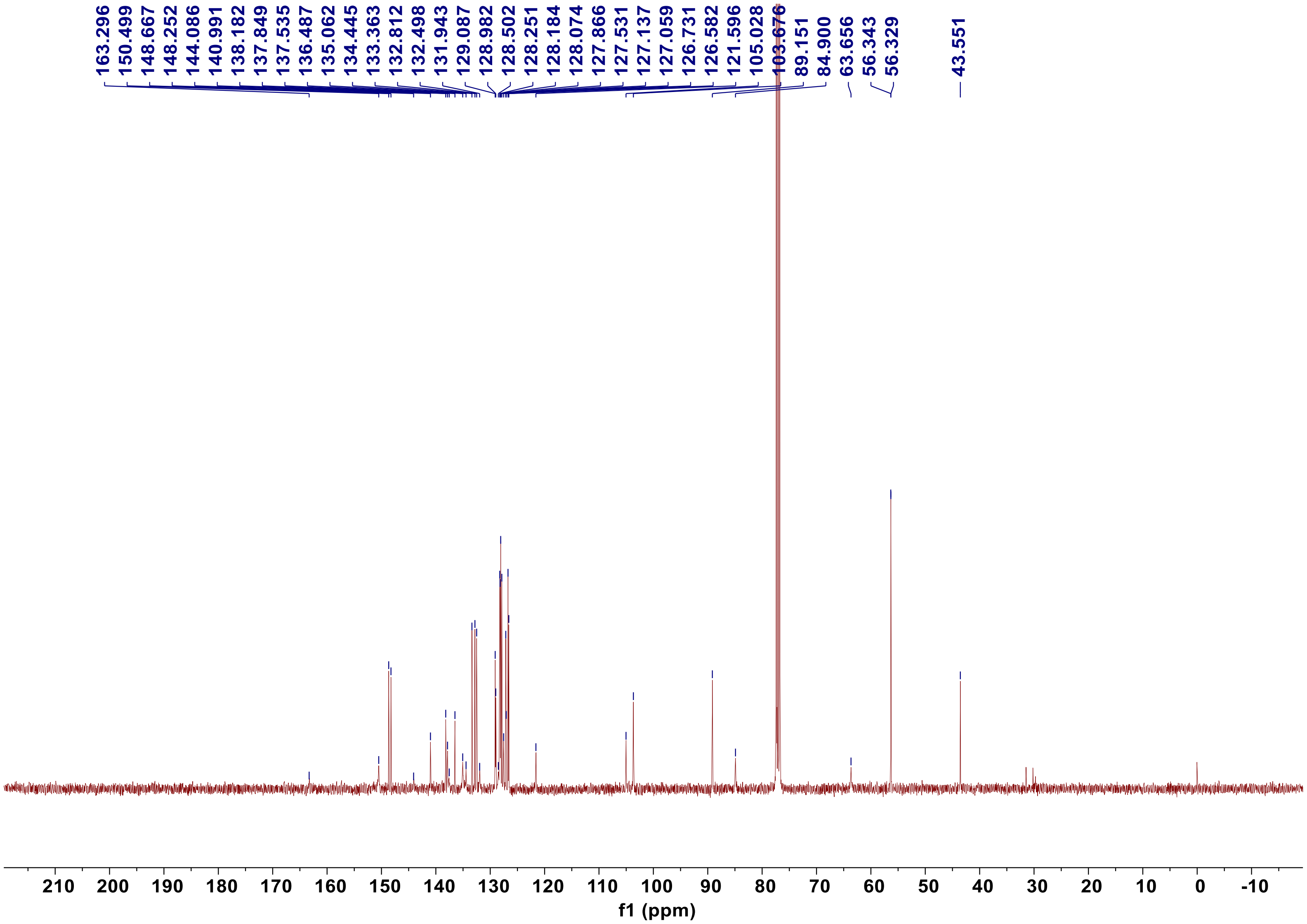

^{1}H NMR of **6g-1**

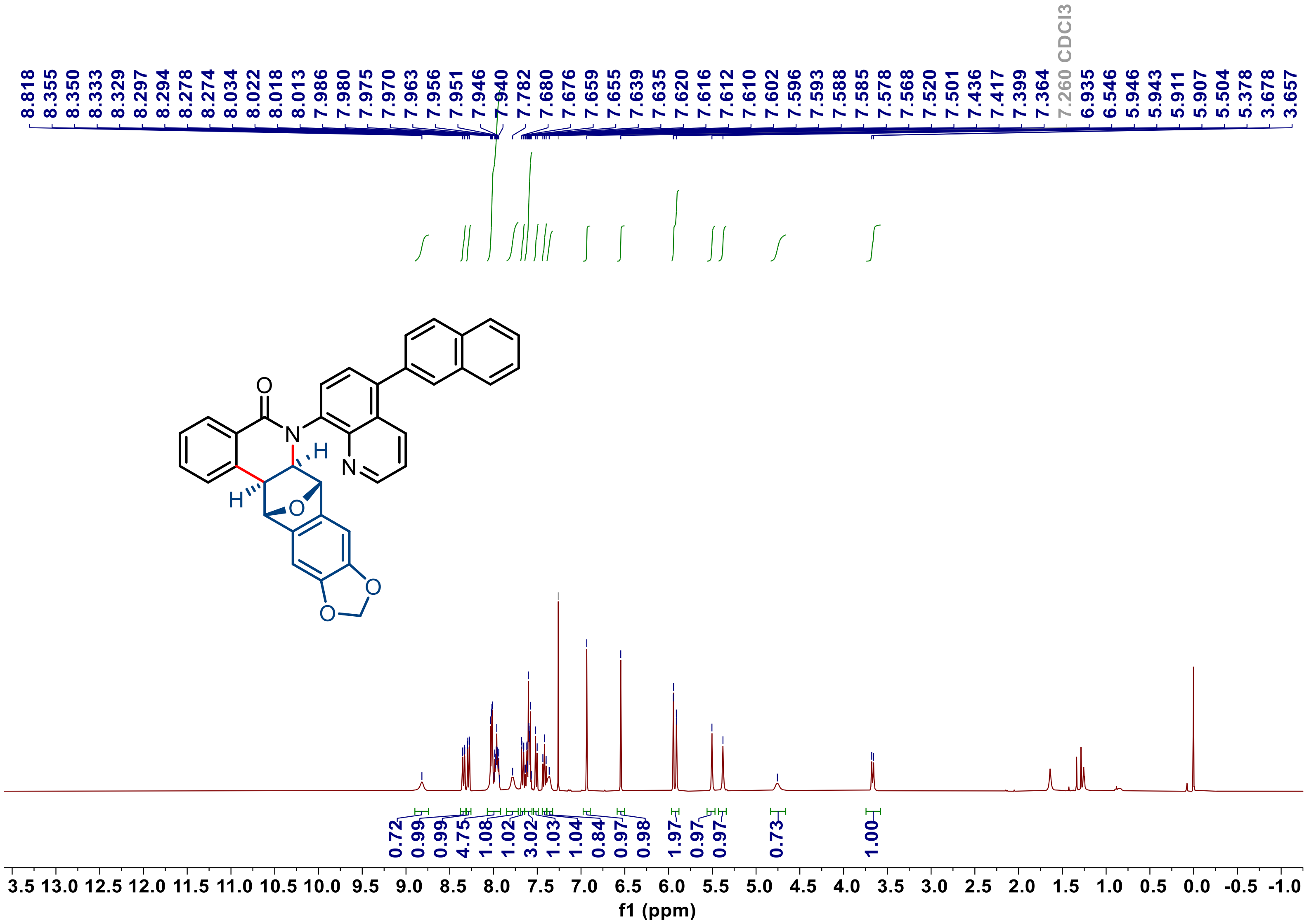
8.818
8.355
8.350
8.333
8.329
8.297
8.294
8.278
8.274
8.034
8.022
8.018
8.013
7.986
7.980
7.975
7.970
7.963
7.956
7.951
7.946
7.940
7.782
7.680
7.676
7.659
7.655
7.639
7.635
7.620
7.616
7.612
7.610
7.602
7.596
7.593
7.588
7.585
7.578
7.568
7.520
7.501
7.436
7.417
7.399
7.364
7.260 CDCl3
6.935
6.546
5.946
5.943
5.911
5.907
5.504
5.378
3.678
3.657
0.72
0.99
0.99
4.75
1.08
1.02
3.02
1.03
1.04
0.84
0.97
0.98
1.97
0.97
0.97
0.73
1.00
13.5 13.0 12.5 12.0 11.5 11.0 10.5 10.0 9.5 9.0 8.5 8.0 7.5 7.0 6.5 6.0 5.5 5.0 4.5 4.0 3.5 3.0 2.5 2.0 1.5 1.0 0.5 0.0 -0.5 -1.0
f1 (ppm)

^{13}C NMR of **6g-1**

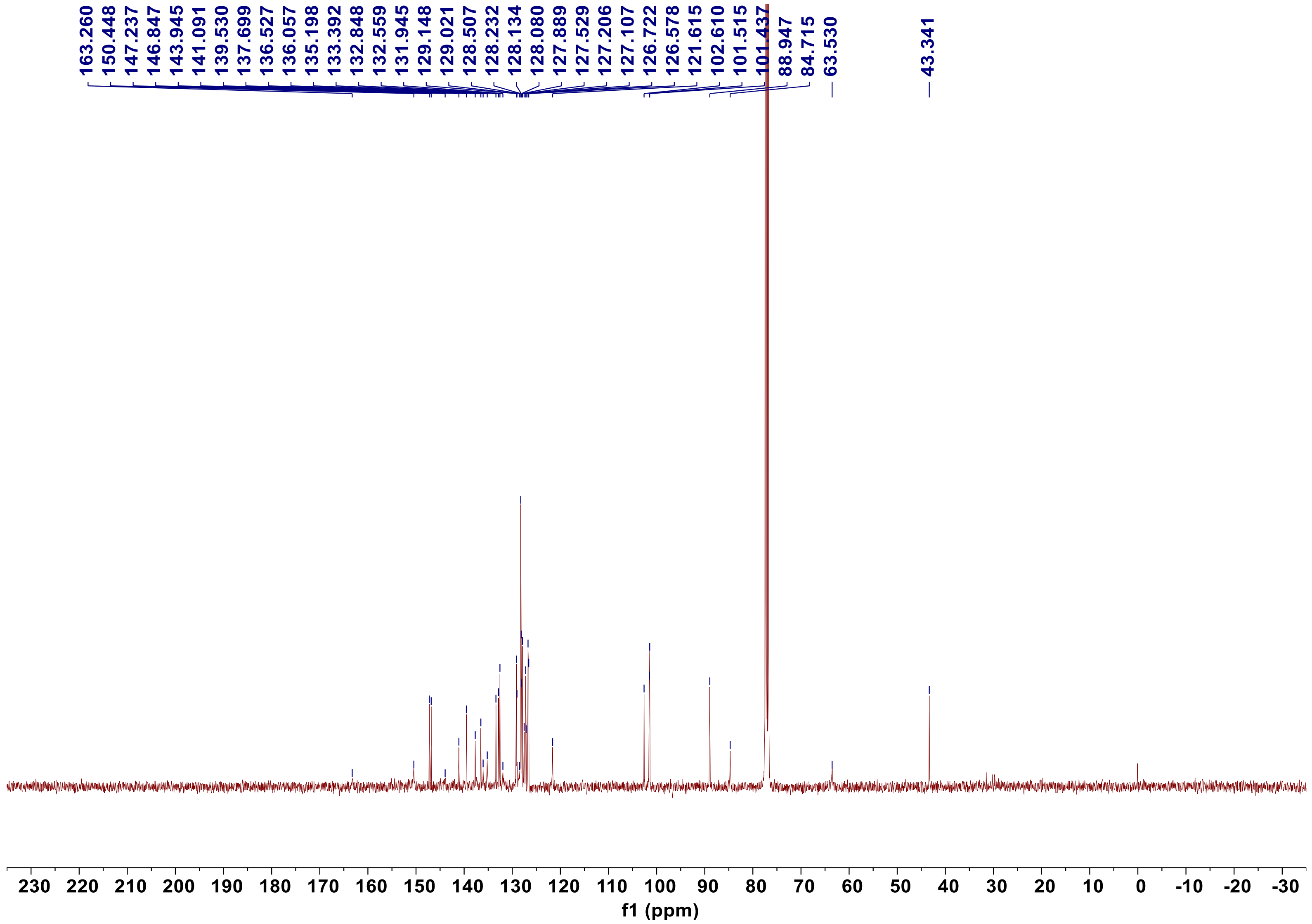

^{1}H NMR of **7g-1**

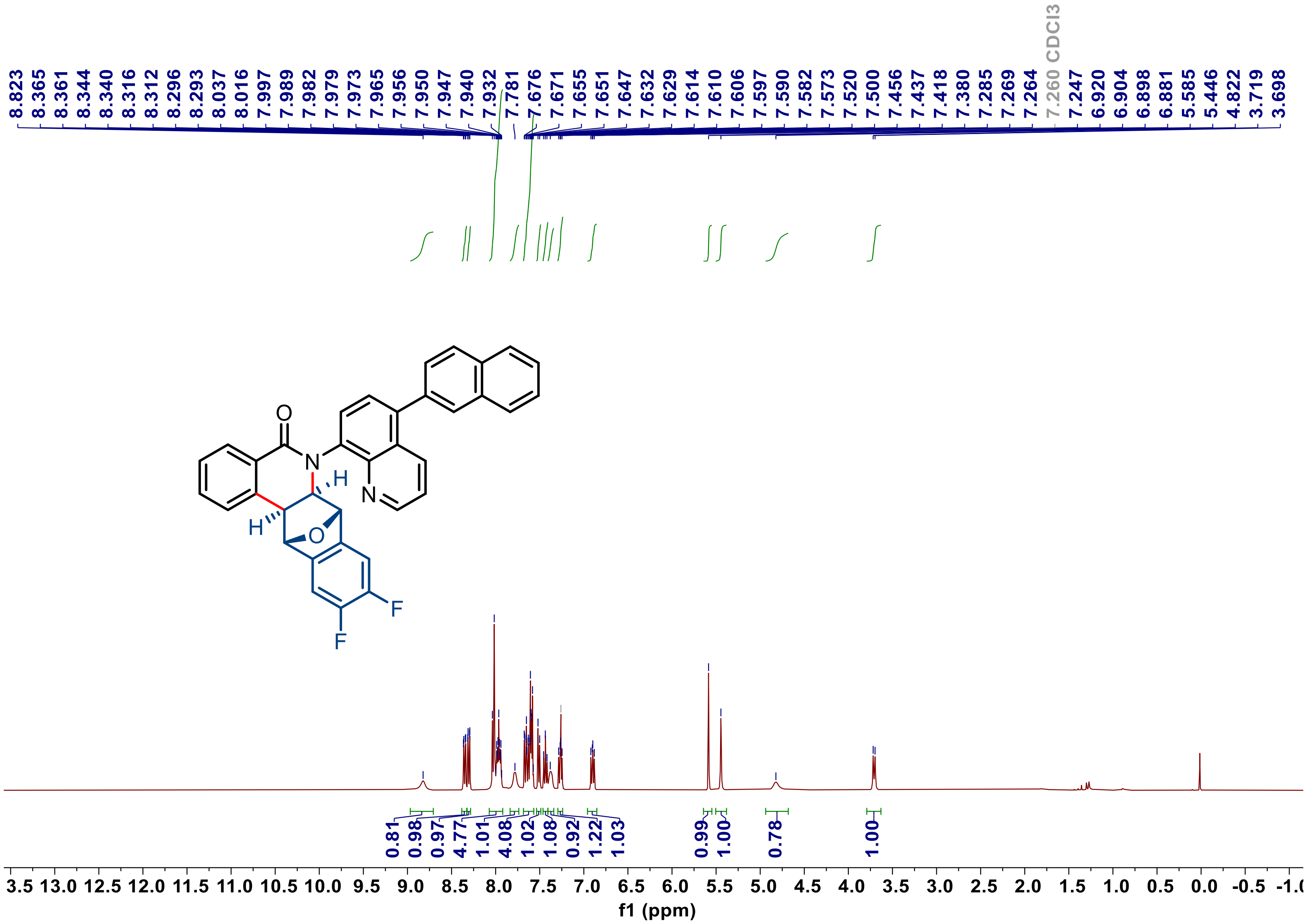
8.823
8.365
8.361
8.344
8.340
8.316
8.312
8.296
8.293
8.037
8.016
7.997
7.989
7.982
7.979
7.973
7.965
7.956
7.950
7.947
7.940
7.932
7.781
7.676
7.671
7.655
7.651
7.647
7.632
7.629
7.614
7.610
7.606
7.597
7.590
7.582
7.573
7.520
7.500
7.456
7.437
7.418
7.380
7.285
7.269
7.264
7.260 CDCl3
7.247
6.920
6.904
6.898
6.881
5.585
5.446
4.822
3.719
3.698
O
N
H
N
H
O
F
F
0.81
0.98
0.97
4.77
1.01
4.08
1.02
1.08
0.92
1.22
1.03
0.99
1.00
0.78
1.00
f1 (ppm)

^{19}F NMR of **7g-1**

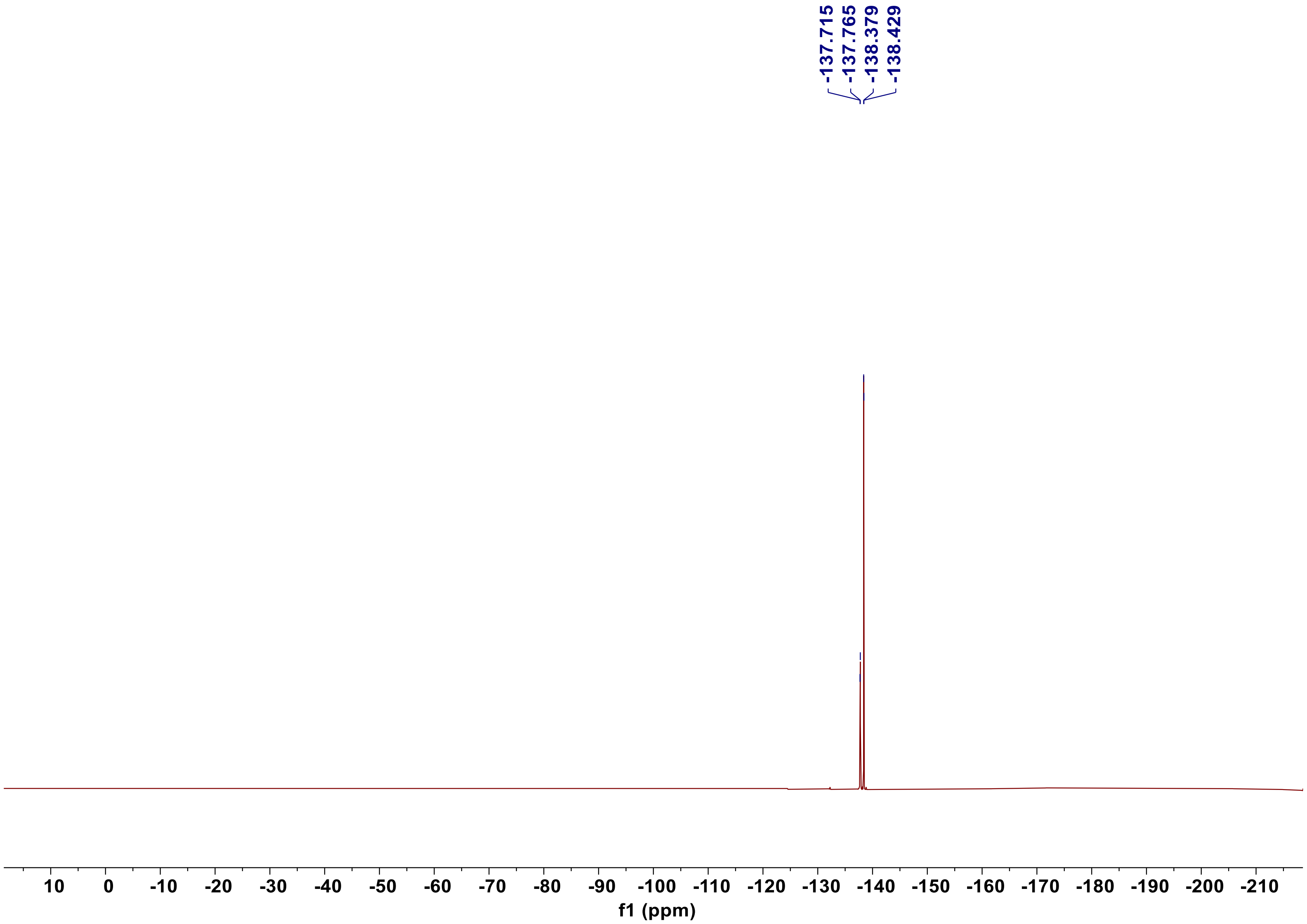

^{13}C NMR of **7g-1**

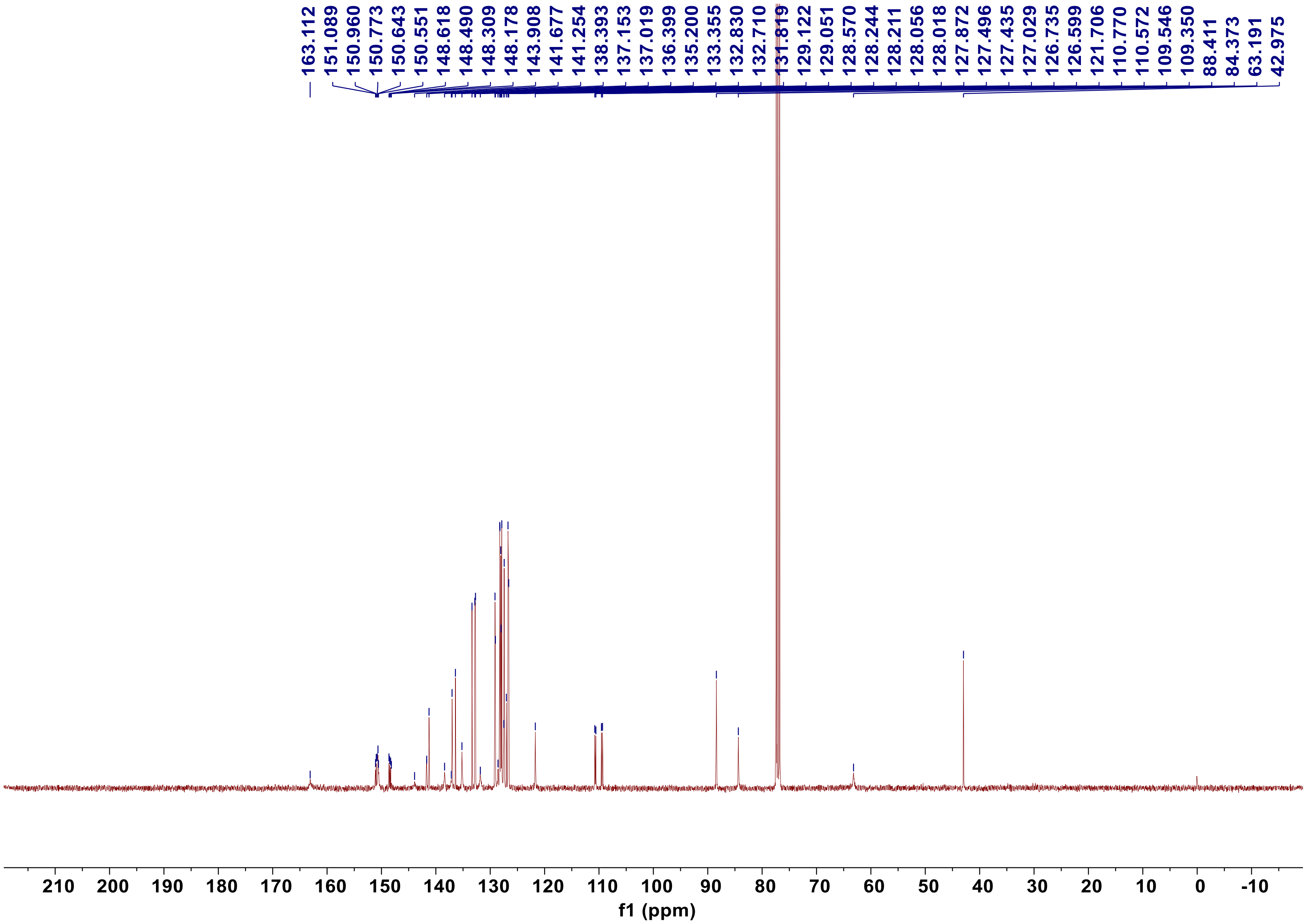

^{1}H NMR of **8g-1**

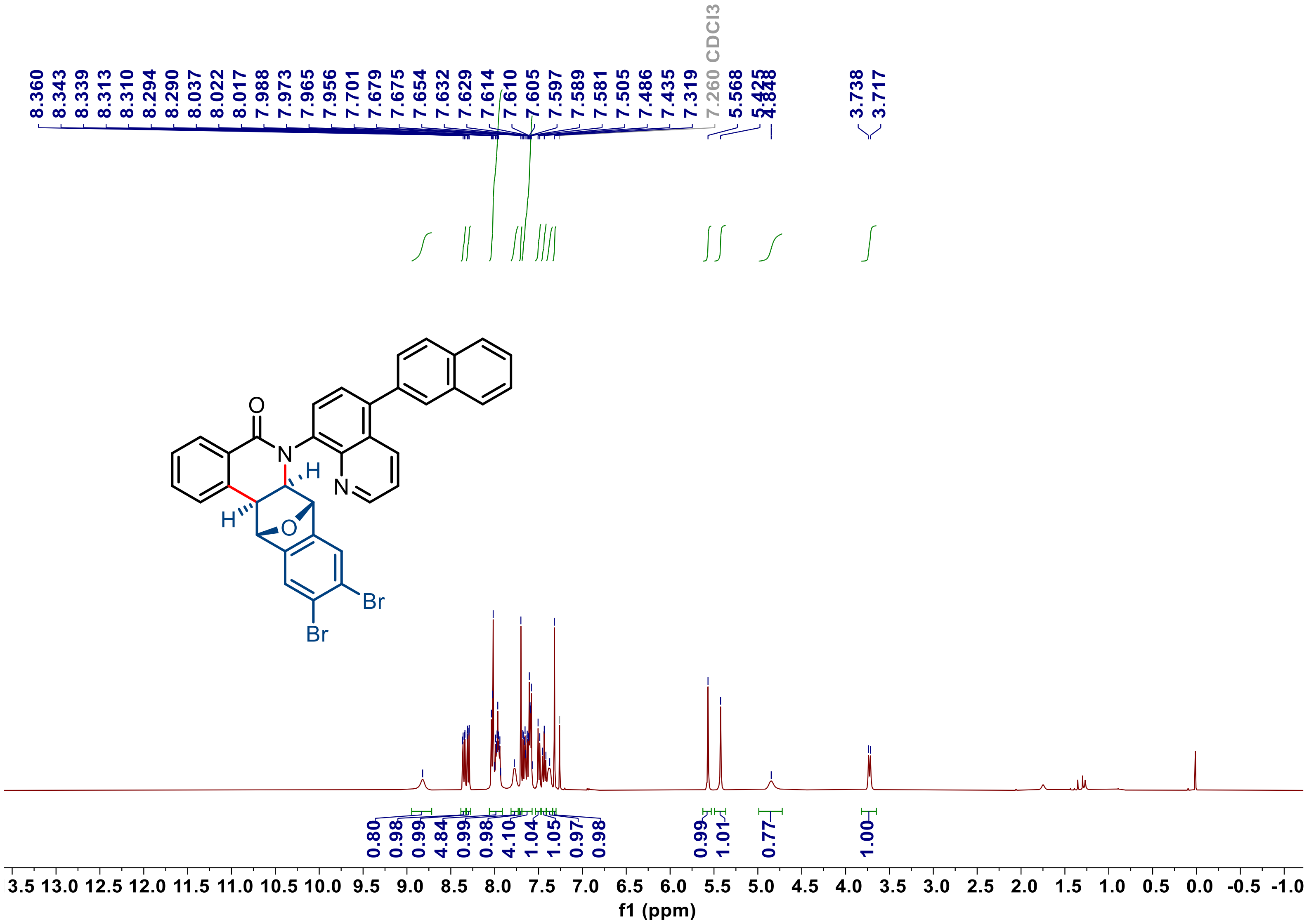
8.360
8.343
8.339
8.313
8.310
8.294
8.290
8.037
8.022
8.017
7.988
7.973
7.965
7.956
7.701
7.679
7.675
7.654
7.632
7.629
7.614
7.610
7.605
7.597
7.589
7.581
7.505
7.486
7.435
7.319
7.260 CDCl3
5.568
5.425
4.848
3.738
3.717
O
N
H
H
N
O
Br
Br
0.80
0.98
0.99
4.84
0.99
0.98
4.10
1.04
1.05
0.97
0.98
0.99
1.01
0.77
1.00
13.5 13.0 12.5 12.0 11.5 11.0 10.5 10.0 9.5 9.0 8.5 8.0 7.5 7.0 6.5 6.0 5.5 5.0 4.5 4.0 3.5 3.0 2.5 2.0 1.5 1.0 0.5 0.0 -0.5 -1.0
f1 (ppm)

^{13}C NMR of **8g-1**

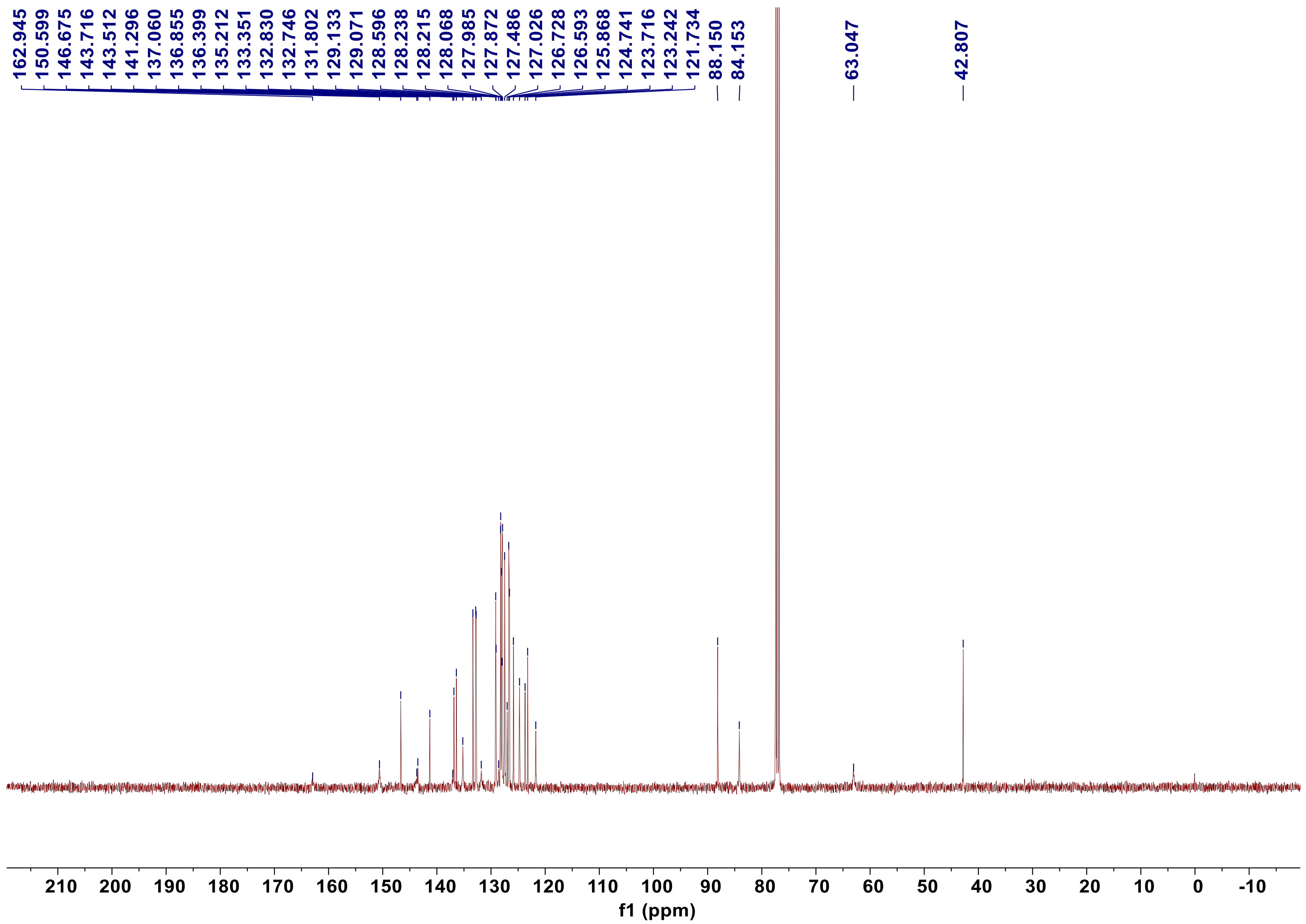

^{1}H NMR of **9g-1**

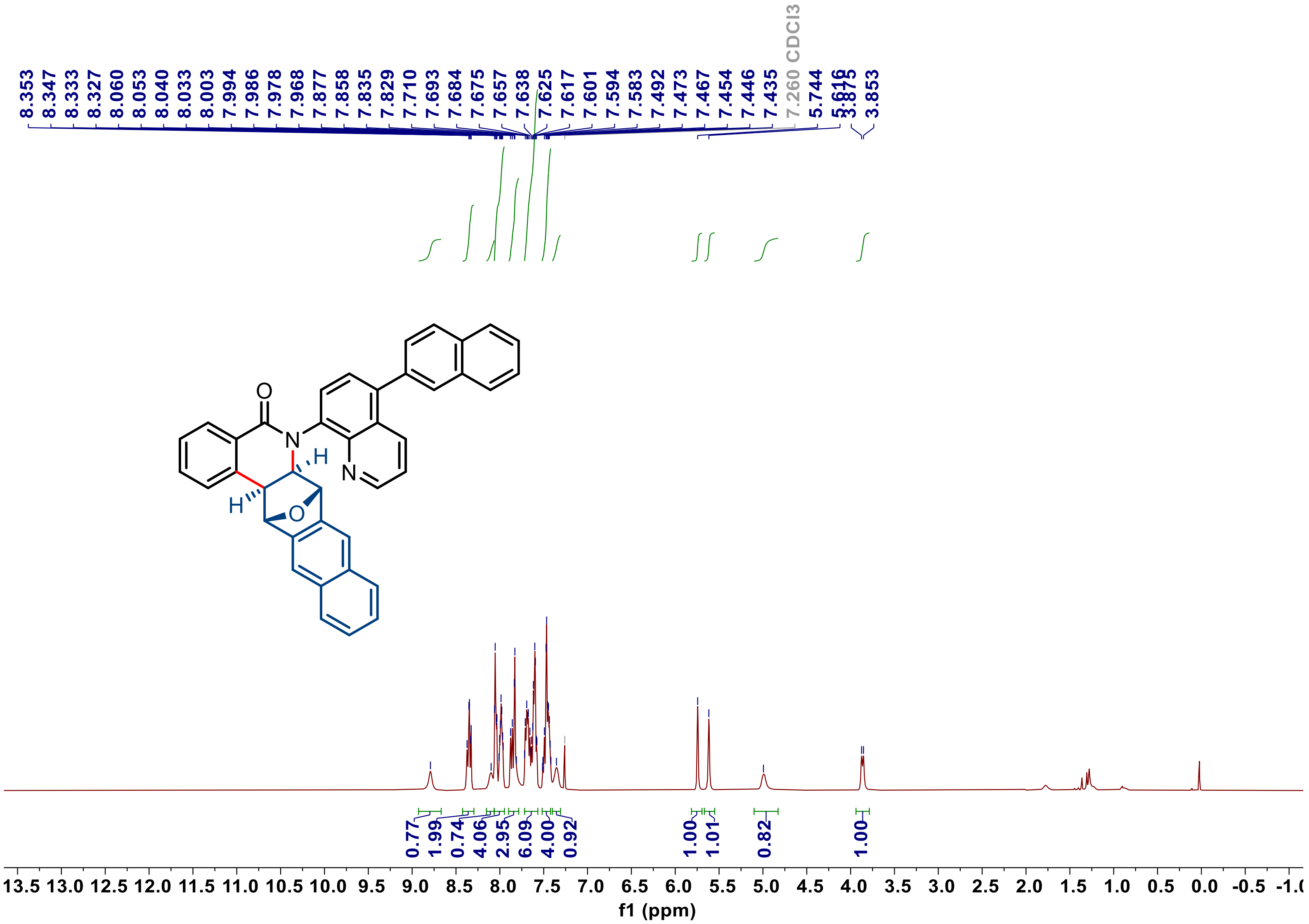
8.353
8.347
8.333
8.327
8.060
8.053
8.040
8.033
8.003
7.994
7.986
7.978
7.968
7.877
7.858
7.835
7.829
7.710
7.693
7.684
7.675
7.657
7.638
7.625
7.617
7.601
7.594
7.583
7.492
7.473
7.467
7.454
7.446
7.435
7.260 CDCl3
5.744
5.616
3.875
3.853
O
N
H
N
H
O
0.77
1.99
0.74
4.06
2.95
6.09
4.00
0.92
1.00
1.01
0.82
1.00
13.5 13.0 12.5 12.0 11.5 11.0 10.5 10.0 9.5 9.0 8.5 8.0 7.5 7.0 6.5 6.0 5.5 5.0 4.5 4.0 3.5 3.0 2.5 2.0 1.5 1.0 0.5 0.0 -0.5
f1 (ppm)

^{13}C NMR of **9g-1**

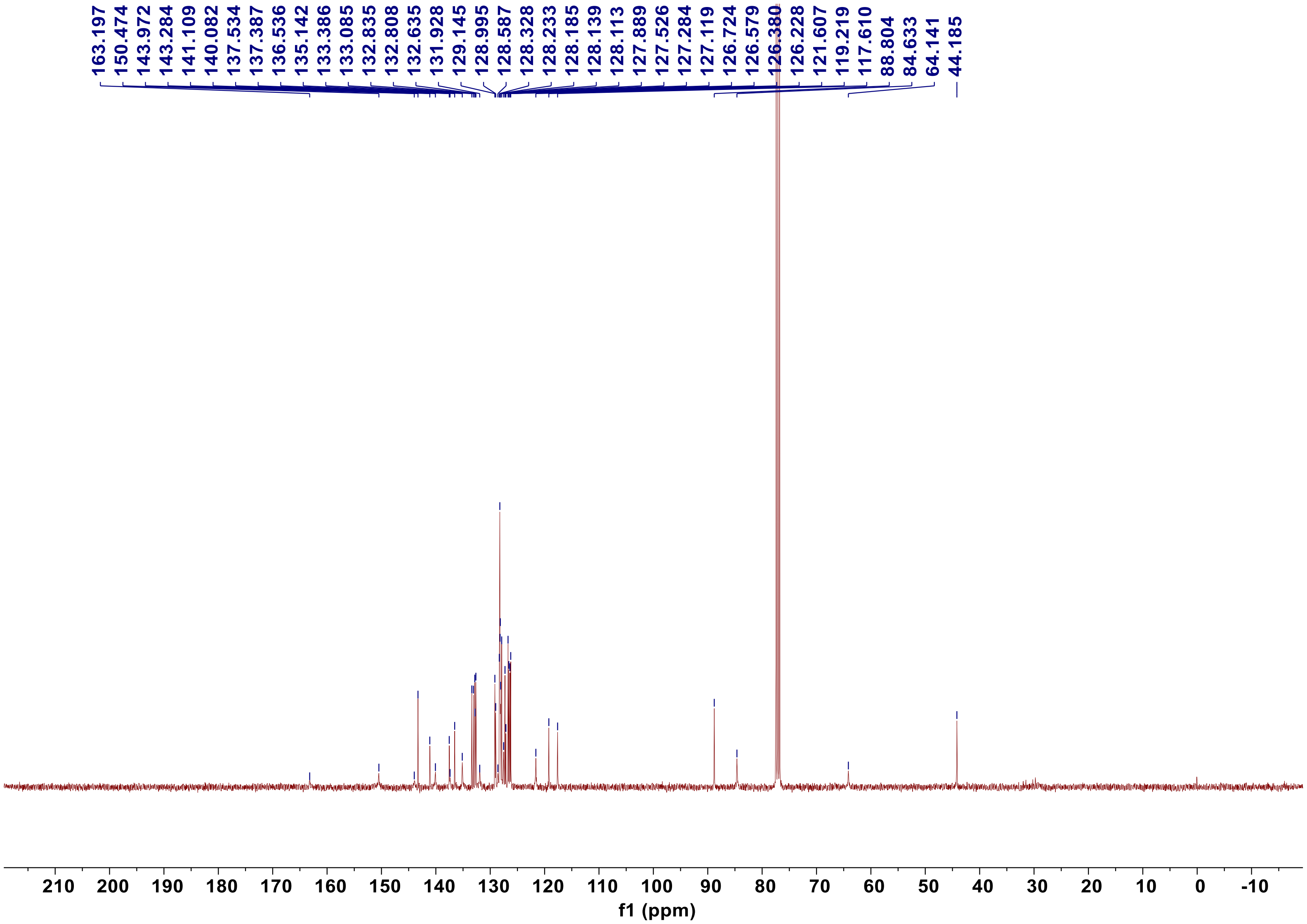

^{1}H NMR of **10**

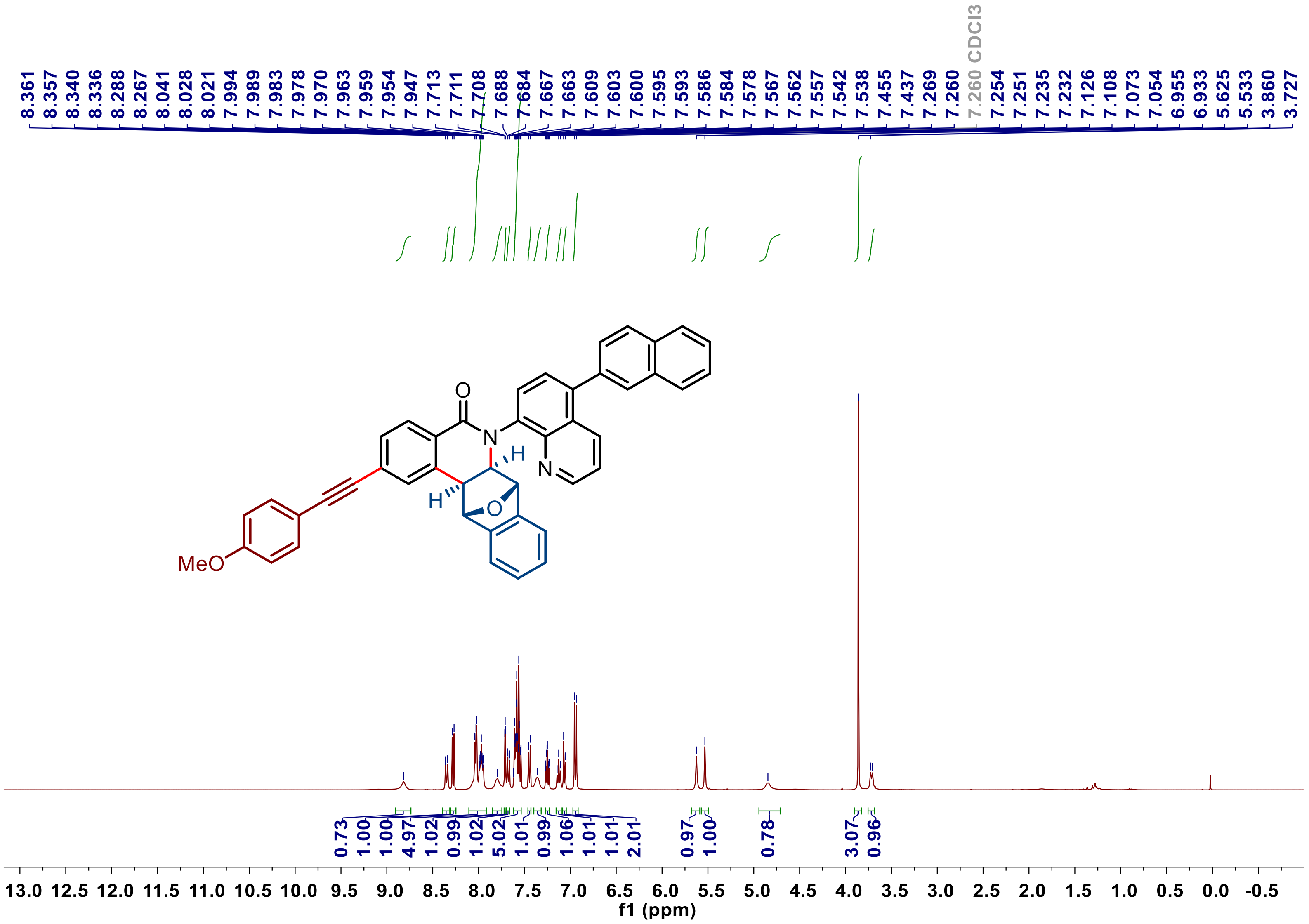

^{13}C NMR of **10**

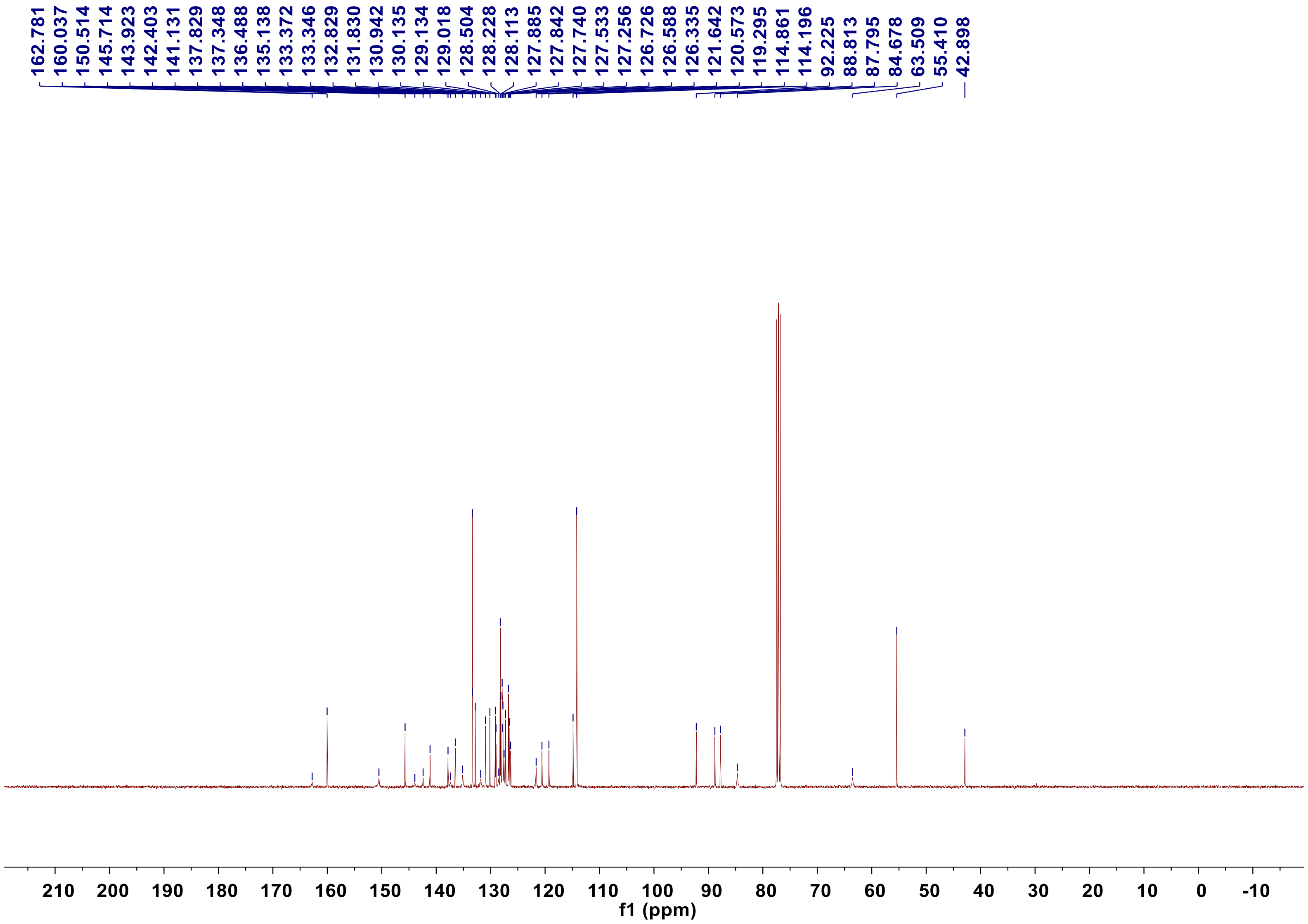
162.781
160.037
150.514
145.714
143.923
142.403
141.131
137.829
137.348
136.488
135.138
133.372
133.346
132.829
131.830
130.942
130.135
129.134
129.018
128.504
128.228
128.113
127.885
127.842
127.740
127.533
127.256
126.726
126.588
126.335
121.642
120.573
119.295
114.861
114.196
92.225
88.813
87.795
84.678
63.509
55.410
42.898
210 200 190 180 170 160 150 140 130 120 110 100 90 80 70 60 50 40 30 20 10 0 -10
f1 (ppm)

^{1}H NMR of **11**

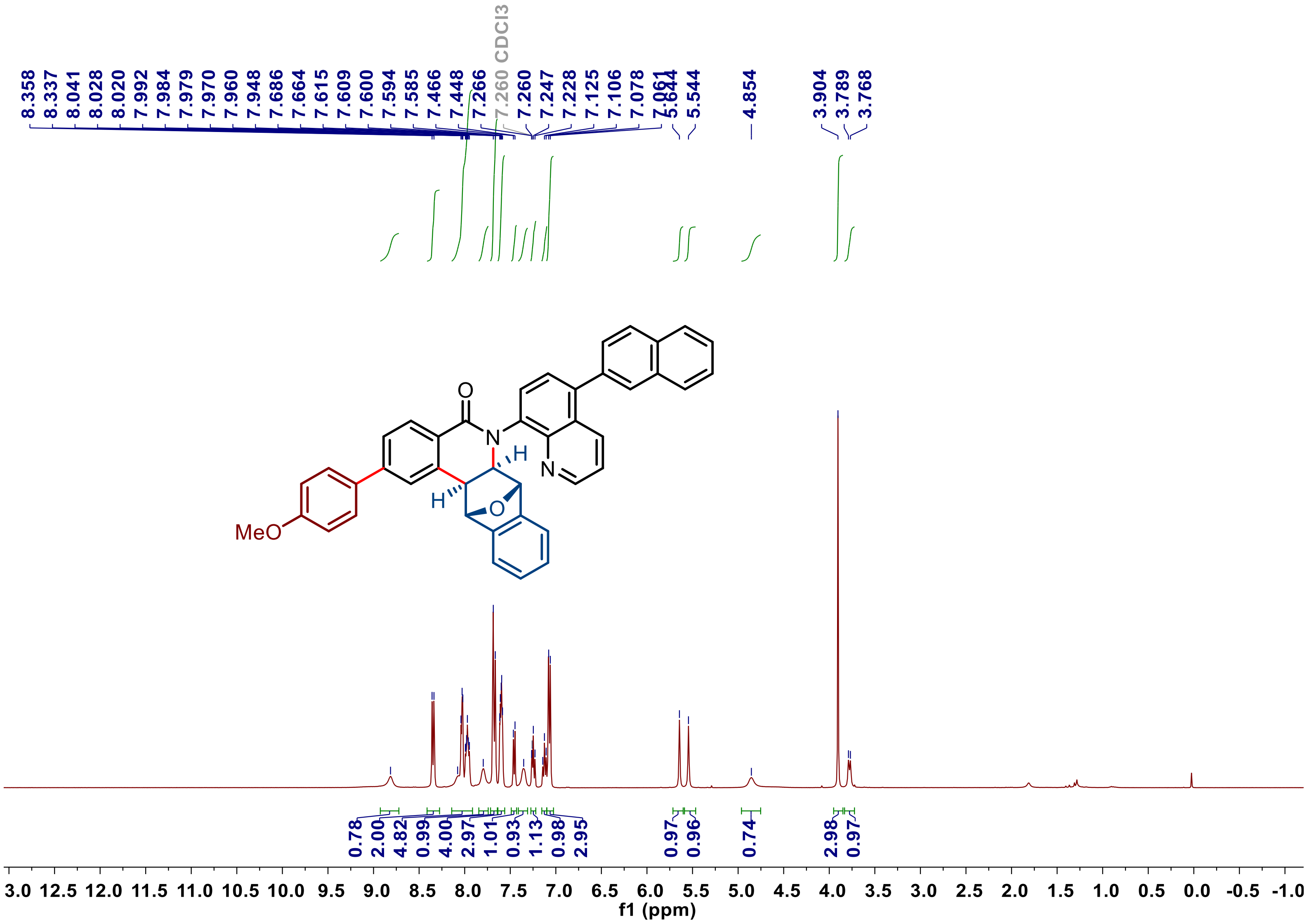

8.358
8.337
8.041
8.028
8.020
7.992
7.984
7.979
7.970
7.960
7.948
7.686
7.664
7.615
7.609
7.600
7.594
7.585
7.466
7.448
7.266
7.260 CDCl3
7.260
7.247
7.228
7.125
7.106
7.078
7.061
5.644
5.544
4.854
3.904
3.789
3.768
0.78
2.00
4.82
0.99
4.00
2.97
1.01
0.93
1.13
0.98
2.95
0.97
0.96
0.74
2.98
0.97
O
N
H
H
O
N
MeO
13.0 12.5 12.0 11.5 11.0 10.5 10.0 9.5 9.0 8.5 8.0 7.5 7.0 6.5 6.0 5.5 5.0 4.5 4.0 3.5 3.0 2.5 2.0 1.5 1.0 0.5 0.0 -0.5 -1.0
f1 (ppm)

^{13}C NMR of **11**

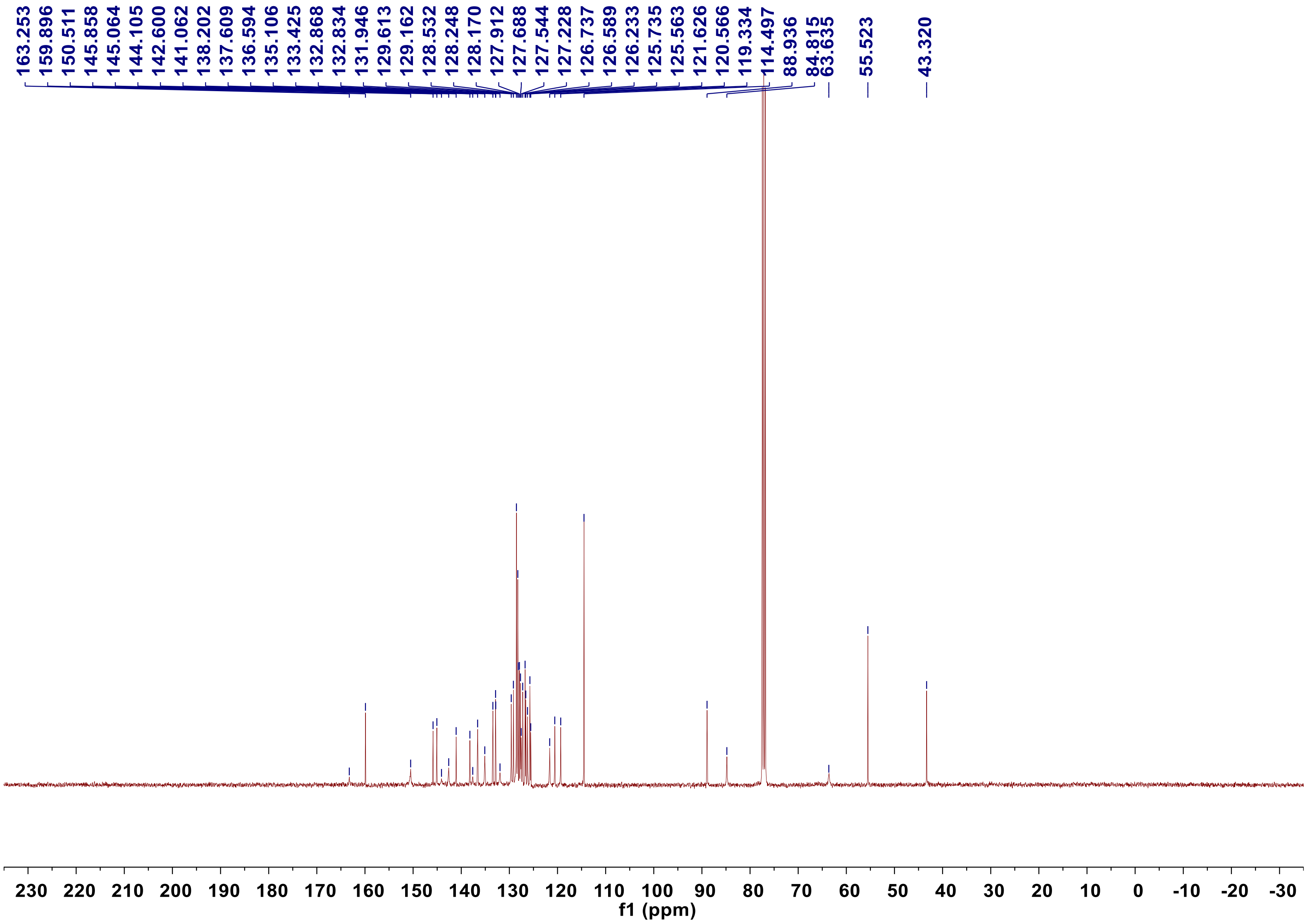
163.253
159.896
150.511
145.858
145.064
144.105
142.600
141.062
138.202
137.609
136.594
135.106
133.425
132.868
132.834
131.946
129.613
129.162
128.532
128.248
128.170
127.912
127.688
127.544
127.228
126.737
126.589
126.233
125.735
125.563
121.626
120.566
119.334
114.497
88.936
84.815
63.635
55.523
43.320
230 220 210 200 190 180 170 160 150 140 130 120 110 100 90 80 70 60 50 40 30 20 10 0 -10 -20 -30
f1 (ppm)

^{1}H NMR of **12**

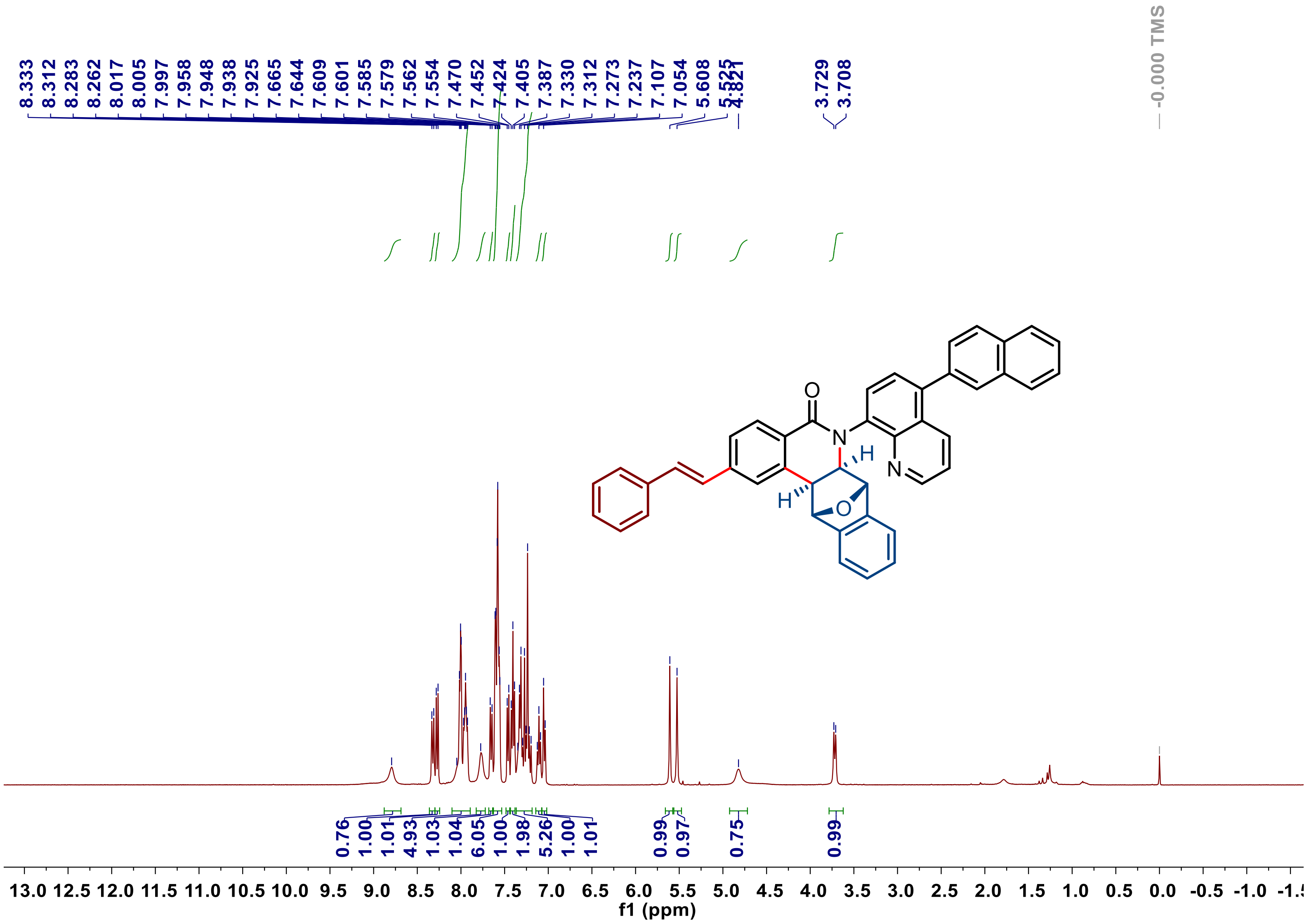

^{13}C NMR of **12**

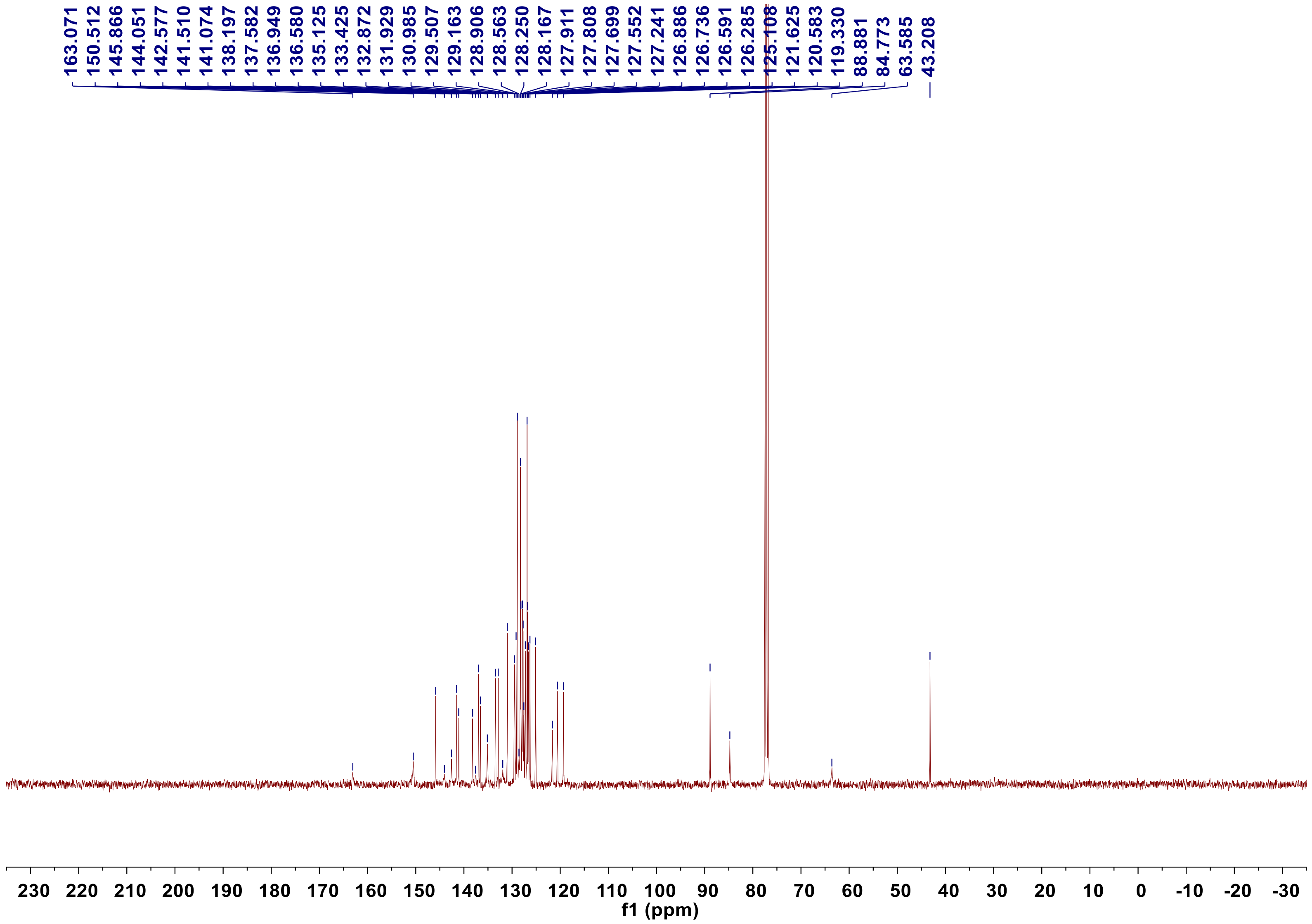
163.071
150.512
145.866
144.051
142.577
141.510
141.074
138.197
137.582
136.949
136.580
135.125
133.425
132.872
131.929
130.985
129.507
129.163
128.906
128.563
128.250
128.167
127.911
127.808
127.699
127.552
127.241
126.886
126.736
126.591
126.285
125.108
121.625
120.583
119.330
88.881
84.773
63.585
43.208
230 220 210 200 190 180 170 160 150 140 130 120 110 100 90 80 70 60 50 40 30 20 10 0 -10 -20 -30
f1 (ppm)

^{1}H NMR of **C-Ni(II)**

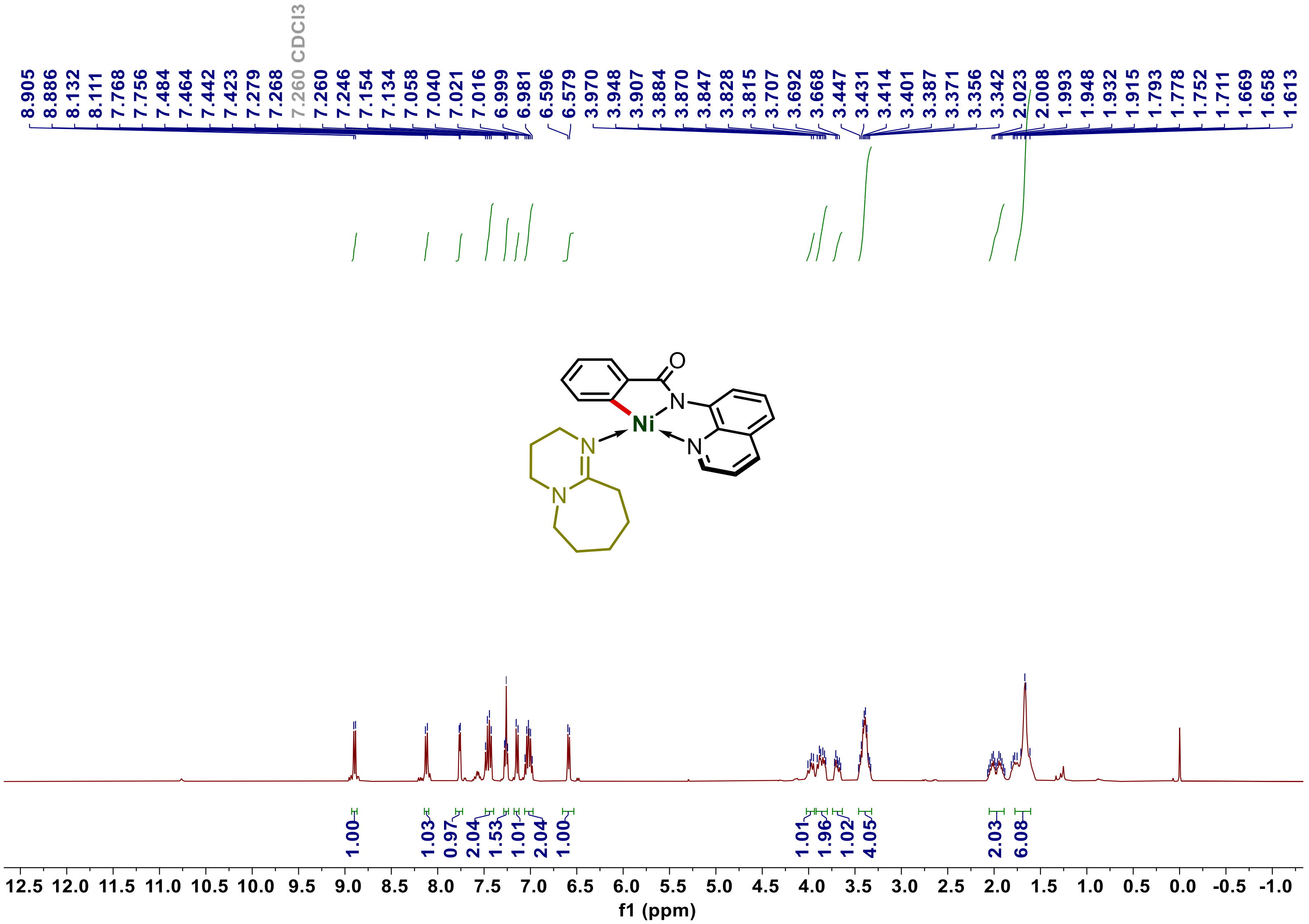

^{13}C NMR of **C-Ni(II)**

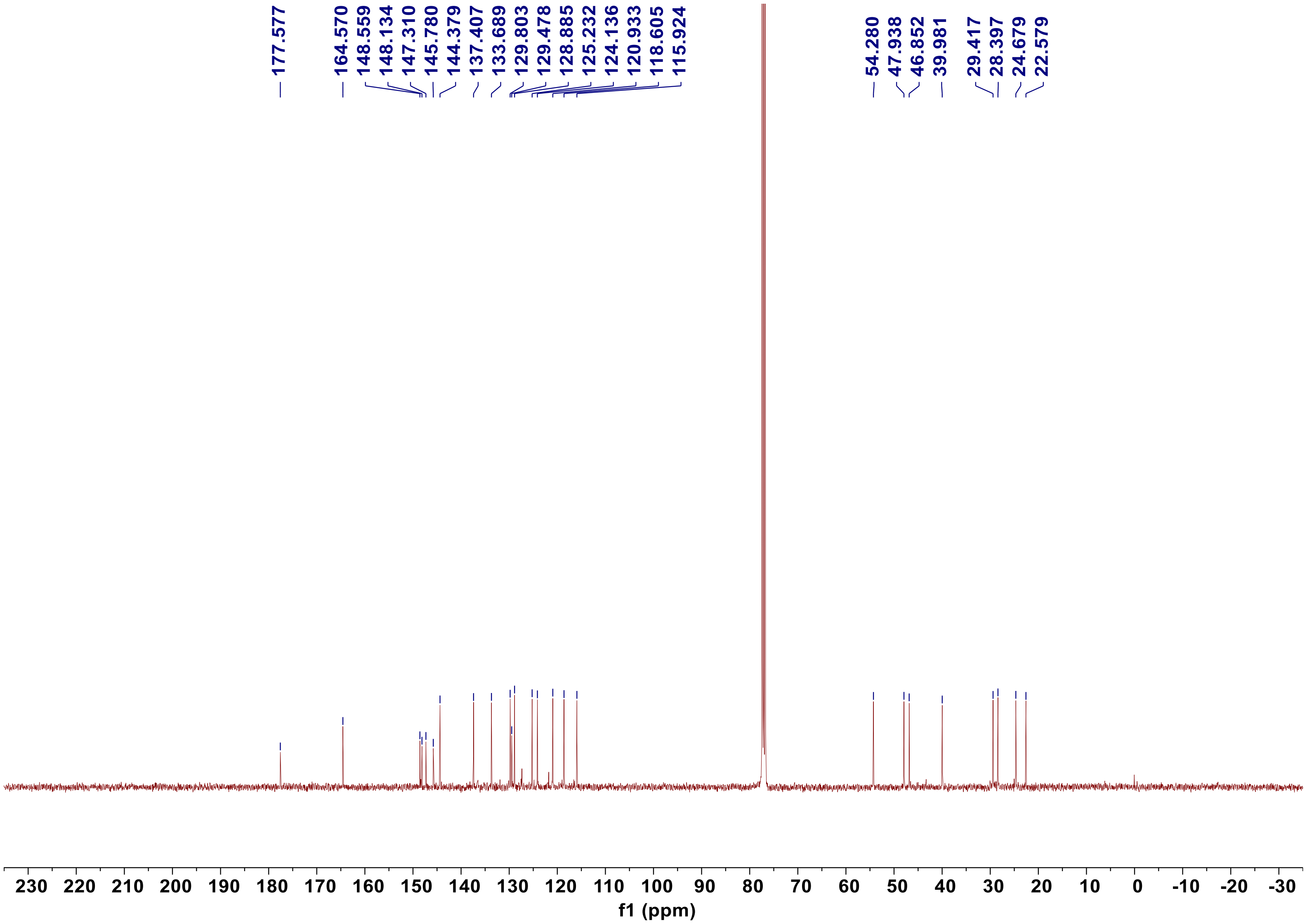

Supplement: Supplementary file 1 — oc4c02049_si_001.pdf [file oc4c02049_si_001.pdf]
